# ﻿ANNiKEY Linear – diagnoses, descriptions, and a single-access identification key to Annelida family-level taxa

**DOI:** 10.3897/zookeys.1247.137606

**Published:** 2025-07-31

**Authors:** Christopher J. Glasby, Olga Biriukova, Patrick Martin, Geoffrey R. Dyne, Serge Utevsky, Robin S. Wilson

**Affiliations:** 1 Museum and Art Gallery Northern Territory, PO Box 4646, Darwin NT 0801, Australia Museum and Art Gallery Northern Territory Darwin Australia; 2 Australian Museum Research Institute, Australian Museum, 1 William Street, Sydney, NSW 2010, Australia Australian Museum Research Institute, Australian Museum Sydney Australia; 3 Natural Sciences Department, Tāmaki Paenga Hira – Auckland Museum, Parnell, Auckland 1010, New Zealand Tāmaki Paenga Hira – Auckland Museum Auckland New Zealand; 4 Royal Belgium Institute of Natural Sciences, Rue Vautier 29, 1000 Bruxelles, Belgium Royal Belgium Institute of Natural Sciences Bruxelles Belgium; 5 Australian National Insect Collection, Black Mountain Laboratories, GPO Box 1700, ACT 2601, Canberra, Australia Australian National Insect Collection, Black Mountain Laboratories Canberra Australia; 6 V. N. Karazin Kharkiv National University, Svobody Sq. 4, 61022, Kharkiv, Ukraine V. N. Karazin Kharkiv National University Kharkiv Ukraine; 7 Sciences Department, Museums Victoria Research Institute, Museums Victoria, GPO Box 666, Melbourne, Victoria 3001, Australia Museums Victoria Research Institute, Museums Victoria Melbourne Australia

**Keywords:** Annelid, ANNiKEY, computer taxonomy, DELTA, diagnosis, interactive key, linear key, natural language descriptions, taxonomic verification

## Abstract

Phylum Annelida are ubiquitous metazoans found in almost every terrestrial and aquatic habitat on Earth. Historically, taxonomic studies on the phylum have been focused largely on its majorgroups, polychaetes, oligochaetes and leeches, so that while family-level keys for each group are available, no single-source identification guide exists to the world’s annelid families. Here, the first illustrated linear key to annelid families is provided and family-level descriptions and diagnoses that distinguish individuals of each family from those of other families in the phylum are updated. This information is generated from an annelid DELTA database of 334 characters and 166 mostly family-level taxa. A link is provided to downloadable software (ANNiKEY Interactive) allowing the same data to be interrogated using the open-source DELTA program Intkey, which enables both interactive identification and taxonomic query functionality. For each family-level taxon, a diagnosis, full description, links to taxonomic data at the World Register of Marine Species, illustrations of diagnostic features, and a summary of the recent literature, including a list of published keys to genera and species are provided.

## ﻿﻿Introduction

Annelida is a large phylum with approximately 20,000 species (Rouse et al. 2022; World Register of Marine Species (WoRMS) 2025) and is found in almost every terrestrial and aquatic habitat on Earth. For most of the last 170 years, Annelida have been divided into three majorclasses, Polychaeta (bristleworms), Oligochaeta (earthworms and allies) and Hirudinea (leeches). A fourth class, Archiannelida, containing a mix of minute polychaete-like annelids (Hermans 1969), now known to be unrelated, was rejected long ago by Fauchald (1977) but not finally abandoned until recent more taxon-specific phylogenomic studies firmly established their links with polychaete families containing large-bodied species (Andrade et al. 2015, and references therein). During the last century, Oligochaeta and Hirudinea were relegated to subclasses under class Clitellata Michaelsen, 1919 in recognition of a majorshared reproductive feature, the clitellum. Today, largely as a result of molecular studies, Clitellata is known to be deeply embedded within Polychaeta, which also includes the former phyla Sipuncula, Echiura, Pogonophora, and Vestimentifera (= Siboglinidae), meaning that ‘Polychaeta’ and ‘Annelida’ are almost one and the same concepts (Rouse et al. 2022, and references therein). However, for the purposes of this review the term ‘polychaete’ is retained as an informal name for the largely marine non-clitellate worms (excluding echiurans and sipunculans) around which particular skills for their study and literature have developed.

Despite these advances, the original taxonomic arrangement of Annelida has more or less set the boundaries for present systematic studies: taxonomists tend to specialize in one of the three majorclasses/subclasses and very few workers published on more than one class of annelid. This prevailing class-focused taxonomy has largely been detrimental for annelid systematics: firstly, detailed morphological comparisons between classes and orders of Annelida have been few, which has potentially hindered phylogenetic studies; and secondly, different morphological terms and segment numbering systems were developed for each of the majorgroups (for a review on how this impacted knowledge of Siboglinidae, see Rouse et al. 2022). The present study uses, where possible, a common set of characters including a reconciled segment numbering system, to review the morphology of Annelida.

In the last ten years or so, annelid systematics has seen several important contributions that have greatly influenced higher-level taxon concepts (Weigert et al. 2014; Tessler et al. 2018; Erséus et al. 2020; Schmelz et al. 2021; Rouse et al. 2022). With these advancements in our understanding of annelid phylogeny, it was considered important to provide an up-to-date description of each of the family concepts, using a standard set of morphological characters, including both external and internal features. Currently, there is a marked disparity in the type of characters used across the group, with internal characters being heavily utilized for clitellates and external characters for most groups of polychaetes, though this was not always the case. Studies on the internal anatomy of polychaetes were common in the late 1800s and early 1900s and this knowledge has been masterfully captured in Rouse et al. (2022). This review serves not only to incorporate revised morphological data into formal higher-taxon descriptions but also to highlight character data currently missing from these descriptions. Further, as far as we know there is no single key for all annelid families, although keys to families of the majorgroups exist (e.g., Fauchald 1977; Brinkhurst and Gelder 2001; Glasby and Fauchald 2003; Govedich et al. 2009; Gil 2011; Thorp et al. 2019). A single key for all annelids will facilitate the identification of families showing intermediate characteristics, such as Acanthobdellidae, Aeolosomatidae, and Hrabeiellidae.

Despite the ecological and phylogenetic importance of Annelida and recent taxonomic progress, the identification of annelid specimens remains a challenge, especially to non-specialists. The need for identification tools is widespread, not least from those conducting molecular systematics studies to resolve problematic taxa. Microdrile oligochaetes are ubiquitous in a broad range of aquatic habitats on land and in the ocean, but outside Europe and North America they are poorly known and mostly neglected for environmental studies because of the perceived difficulty in identifying species. For megadrile oligochaetes (Metagynophora *sensu* Jamieson, 1988), there are a significant number of exotic/peregrine species that have been deliberately or accidentally introduced around the globe, and these can often be detected at the family level if such families are known to not be a part of the endemic fauna. Therefore, for oligochaetes in general, accurate family-level identification may be beneficial for ecological and environmental studies.

Likewise, polychaete species identification also remains a challenge, particularly in many parts of the Southern Hemisphere. However, because of their ubiquity in marine and coastal sediments, polychaetes are often only identified to family in ecological and environmental studies for cost-benefit reasons – family-level identification is more easily achieved and sufficient in order to detect compositional and/or abundance differences in benthic assemblages as a result of environmental disturbance (see POLiKEY web page; https://www.dcceew.gov.au/science-research/abrs/online-resources/polikey). Family-level identification of polychaetes typically still relies on the family concepts of Fauchald (1977), limiting their ecological value due to intra-family variation of feeding modes at that level (Fauchald and Jumars 1979). Our adoption of polychaete subfamilies in the present study aligns with the subfamily ‘morphotypes’ documented in the revised ‘Diet of Worms’ paper (Jumars et al. 2015) and should allow studies using family-level identification to collect data that are more ecologically meaningful.

For these reasons, we provide this review with the aim of providing:

1. an updated family-level diagnoses and descriptions (Suppl. material 1) and a summary of the recent taxonomic literature, especially those containing keys to genera and species.
2. an illustrated key to family-level taxa including a linear key (herein) and an online key (ANNiKEY Interactive; Glasby et al. 2025a).
3. an annotated morphological character list and NEXUS file for phylogenetic analysis (Suppl. materials 2, 3).

## ﻿﻿Methods

### ﻿﻿Taxonomic software, concepts, and outputs

We used the software DELTA (Descriptive Language for Taxonomy) (Dallwitz 1974, 1980; Dallwitz et al. 1993; Dallwitz and Paine 2015) to create and manage taxonomic data to support objective and quantitative description and discrimination of annelid taxa. Version history and availability of the programs are provided in Wilson et al. (2023). Taxa were scored primarily using the literature (both primary and compendia). The principal outputs of DELTA included in this paper are a linear key (herein), an online key (ANNiKEY Interactive; Glasby et al. 2025a), and taxon treatments, including the common name for the family, a Life Sciences Identifier (LSID) which links to taxonomic information in the World Register of Marine Species (WoRMS 2025), a DELTA diagnosis for each taxon, natural language descriptions (Suppl. material 1) and Remarks, which includes, for the marine taxa (mainly polychaetes), an estimation of its distribution based on visualization of the Global Biodiversity Information Facility (GBIF) maps generated by taxon occurrence searches (GBIF.org 2023). An annotated character list of the morphological characters and a NEXUS file are also included in Suppl. materials 2, 3, respectively.

Taxon diagnoses are ‘minimal diagnoses’, generated through DELTA Intkey, which are a list of those characters which alone are sufficient to distinguish individuals of the family from all other families in their taxonomic group (i.e., polychaete, echiuran, sipunculan, leech, megadrile and microdrile), and to verify identifications by detecting errors that may have been made while using the key. DELTA’s diagnostic levels provide cumulative redundancy so that if Diagnostic Level 3 (the maximum level we assessed) is achieved then the given diagnosis differs from all other taxa (of the majorgroup) by at least three characters from every other taxon. Thus, where diagnostic Level 2 is achieved those taxa differ by at least two characters. Diagnostic Level 1 indicates a difference of only one character from the other taxa of the majorgroup, i.e., the family as coded is not very distinct. Level 0 families are undiagnosable using the present dataset (i.e., not separable from one or more other families), although we still provide characters that are typical of the group. In the case of Level 0, the reader is referred to the full description (Suppl. material 1).

Other identification and diagnostic functions are available in ANNiKEY Interactive. Additional identification power is enabled when interrogated using the Intkey software, since during an identification, Intkey can also display diagnostic characters, and in that situation, the diagnostic characters will only include characters not already used during that interactive identification session. In addition to diagnosing error detection, ANNiKEY Interactive offers two other forms of identification confirmation: descriptions (same as in Suppl. material 1) and taxon images (a greater range of images than in this paper). Traditional paper-based linear keys rarely provide this facility, since the author has selectively chosen one or two characters believed to be the best for binary decision-making. The linear key provided here thus has a lower level of self-checking (fewer taxon images); nevertheless, diagnoses, full descriptions (Suppl. material 1), and family-level ‘plates’ are provided here, and access to higher magnification versions of the plates are available as part of the downloadable ANNiKEY Interactive repository (Glasby et al. 2025a).

Our Intkey files are available as a separate download (ANNiKEY Interactive; Glasby et al. 2025a) and require prior installation of the (recommended) Open DELTA software (https://github.com/AtlasOfLivingAustralia/open-delta) for all operating systems or the original DELTA programs (Windows only; Dallwitz 2020). This paper serves as an alternative for those unable to install the DELTA software. ANNiKEY Interactive, however, is more informative and more powerful as it includes illustrated morphological characters and selected biological and environmental traits; although not comprehensive, the traits allow the user to identify subgroups selected by buttons on a toolbar, thus greatly facilitating identification by limiting available taxa (e.g., only freshwater annelids, or only annelids formerly belonging to the phylum Echiura).

Finally, we provide a NEXUS file for phylogenetic analysis, which contains 156 taxa and 274 characters (Suppl. material 3). We excluded redundant taxa (i.e., when subfamilies were included because of evidence of monophyly, the parent family was excluded) including two tribes (Polycirrini and Alciopini), which, if included, rendered their respective higher taxa non-monophyletic. The characters included both binary and unordered multistate ones (text-based, integer and real number characters were excluded, as per Wilson et al. 2023) and thus represented a subset of the 288 morphological characters used to score annelid family-level taxa (see below; Suppl. material 1). The NEXUS file was generated by DELTA from the same DELTA database that was used for the diagnoses and descriptions. As preparation of the NEXUS file required trimming characters and states to 30 characters or less, we included the full character list at the end of the NEXUS file. The file is readable with all currently available phylogenetic software as is or with minor format editing.

### ﻿﻿Taxa

A total of 166 taxa are included in the key, mostly representing family-level, including polychaetes (Polychaeta; 101 families/subfamilies and 1 order), leeches (Hirudinea; 17), megadrile oligochaetes (Crassiclitellata; 20), microdrile oligochaetes (18), Sipuncula (6) and Echiura (5). The atypical polychaete, Myzostomida, symbionts of echinoderms, is the only order-level taxon scored, as explained below. Crassiclitellata has had, arguably, the most changes in family-level taxonomy. We largely follow Misirlioğlu et al. (2023) for this group, except for the exclusion of the monotypic Arecoidae James, Csuzdi & Brown, 2023, the monogeneric Diporodrilidae Bouché, 1970 and Kazimierzidae Nxele & Plisko, 2016, and the monotypic Komarekionidae Gates, 1974. Although all of these families are accepted in WoRMS (2025), their members are rarely collected, and all appear to need more scrutiny to confirm their familial ranking, especially from a phylogenomic viewpoint.

Family-level concepts for all non-clitellate annelids follow WoRMS (2025), which is largely based on Rouse and Pleijel (2001), and updated using Rouse et al. (2022); however, note that our non-clitellate family count is much higher than in Rouse et al. (2022) because we have included families comprising only a single genus unlike Rouse et al. (2022) who abandoned the family name of singletons arguing that they were taxonomically redundant. Families of Sipuncula follow Schulze et al. (2019); Echiura has been demoted to a family (Thalassematidae) of Polychaeta following Goto et al. (2020), but we have maintained the traditional five family concepts as in Edmonds (2000) and WoRMS (2025), which are here treated as subfamilies. The echinoderm symbionts Myzostomida (Polychaeta) are treated here at the order level following Rouse et al. (2022), as most of the 6–8 families (depending on authority) are only represented by a single or a few species. Although the order is far from being uniform morphologically, it is nevertheless diagnosable at DELTA Level 3; literature having keys to the families of Myzostomida are provided under that name in the section, Taxonomic accounts. Finally, we include in the dataset two tribes, Alciopini and Polycirinni, representing a former family (Alciopidae) and subfamily (Terebellidae, Polycirrinae), which have been taxonomically demoted following recent phylogenetic analyses (Rouse et al. (2022) and references therein). The complete list of family-level taxa and their higher classification is provided in Table 1.

**Table 1.** Family-level taxa (and common name used in this study) dealt with in this study, arranged taxonomically by class, subclass and order based on the clade-based classification of Schmelz et al. (2021) (Oligochaeta), Tessler et al. (2018) (Hirudinea including acanthobdellids) and Rouse et al. (2022) (Polychaeta including echiurans and sipunculans). Note that Schmelz et al. (2021) and Tessler et al. (2018) disagree with respect to the placement of the ‘true’ leech families – the former place them in order Lumbriculida, whereas the latter place them in order Hirudinida; we have followed the latter.

Class	Subclass	Order-level name	Family-level name	Common name
**Chaetopteriformia**	Chaetopteriformia	Chaetopteriformia Fauchald, 1977	Chaetopteridae	polychaete
**Chaetopteriformia**	Chaetopteriformia	Chaetopteriformia Fauchald, 1977	Apistobranchidae	polychaete
**Chaetopteriformia**	Chaetopteriformia	Chaetopteriformia Fauchald, 1977	Psammodrilidae	polychaete
**Clitellata**	Hirudinea	Acanthobdellida Livanow, 1905	Acanthobdellidae	leech
**Clitellata**	Hirudinea	Branchiobdellida Holt, 1965	Branchiobdellidae	leech
**Clitellata**	Hirudinea	Hirudinida Siddall et al., 2000	Americobdellidae	leech
**Clitellata**	Hirudinea	Hirudinida Siddall et al., 2000	Cyclobdellidae	leech
**Clitellata**	Hirudinea	Hirudinida Siddall et al., 2000	Cylicobdellidae	leech
**Clitellata**	Hirudinea	Hirudinida Siddall et al., 2000	Erpobdellidae	leech
**Clitellata**	Hirudinea	Hirudinida Siddall et al., 2000	Gastrostomobdellidae	leech
**Clitellata**	Hirudinea	Hirudinida Siddall et al., 2000	Glossiphoniidae	leech
**Clitellata**	Hirudinea	Hirudinida Siddall et al., 2000	Haemadipsidae	leech
**Clitellata**	Hirudinea	Hirudinida Siddall et al., 2000	Hirudinidae	leech
**Clitellata**	Hirudinea	Hirudinida Siddall et al., 2000	Orobdellidae	leech
**Clitellata**	Hirudinea	Hirudinida Siddall et al., 2000	Ozobranchidae	leech
**Clitellata**	Hirudinea	Hirudinida Siddall et al., 2000	Praobdellidae	leech
**Clitellata**	Hirudinea	Hirudinida Siddall et al., 2000	Salifidae	leech
**Clitellata**	Hirudinea	Hirudinida Siddall et al., 2000	Semiscolecidae	leech
**Clitellata**	Hirudinea	Hirudinida Siddall et al., 2000	Xerobdellidae	leech
**Clitellata**	Hirudinea	Hirudinida Siddall et al., 2000	Piscicolidae	leech
**Clitellata**	Oligochaeta	Alluroidida Timm & Martin, 2015	Alluroididae	microdrile
**Clitellata**	Oligochaeta	Alluroidida Timm & Martin, 2015	Syngenodrilidae	microdrile
**Clitellata**	Oligochaeta	Capilloventrida Timm, 2021	Capilloventridae	microdrile
**Clitellata**	Oligochaeta	Crassiclitellata Jamieson, 1988	Acanthodrilidae	megadrile
**Clitellata**	Oligochaeta	Crassiclitellata Jamieson, 1988	Almidae	megadrile
**Clitellata**	Oligochaeta	Crassiclitellata Jamieson, 1988	Biwadrilidae	megadrile
**Clitellata**	Oligochaeta	Crassiclitellata Jamieson, 1988	Criodrilidae	megadrile
**Clitellata**	Oligochaeta	Crassiclitellata Jamieson, 1988	Eudrilidae	megadrile
**Clitellata**	Oligochaeta	Crassiclitellata Jamieson, 1988	Glossoscolecidae	megadrile
**Clitellata**	Oligochaeta	Crassiclitellata Jamieson, 1988	Hormogastridae	megadrile
**Clitellata**	Oligochaeta	Crassiclitellata Jamieson, 1988	Kynotidae	megadrile
**Clitellata**	Oligochaeta	Crassiclitellata Jamieson, 1988	Lumbricidae	megadrile
**Clitellata**	Oligochaeta	Crassiclitellata Jamieson, 1988	Lutodrilidae	megadrile
**Clitellata**	Oligochaeta	Crassiclitellata Jamieson, 1988	Megascolecidae	megadrile
**Clitellata**	Oligochaeta	Crassiclitellata Jamieson, 1988	Microchaetidae	megadrile
**Clitellata**	Oligochaeta	Crassiclitellata Jamieson, 1988	Ocnerodrilidae	megadrile
**Clitellata**	Oligochaeta	Crassiclitellata Jamieson, 1988	Rhinodrilidae	megadrile
**Clitellata**	Oligochaeta	Crassiclitellata Jamieson, 1988	Sparganophilidae	megadrile
**Clitellata**	Oligochaeta	Crassiclitellata Jamieson, 1988	Tritogeniidae	megadrile
**Clitellata**	Oligochaeta	Crassiclitellata Jamieson, 1988	Tumakidae	megadrile
**Clitellata**	Oligochaeta	Enchytraeida Kasprzak, 1984	Enchytraeidae	microdrile
**Clitellata**	Oligochaeta	Enchytraeida Kasprzak, 1984	Propappidae	microdrile
**Clitellata**	Oligochaeta	Haplotaxida Brinkhurst & Jamieson, 1971	Haplotaxidae	microdrile
**Clitellata**	Oligochaeta	Lumbriculida Brinkhurst & Jamieson, 1971	Dorydrilidae	microdrile
**Clitellata**	Oligochaeta	Lumbriculida Brinkhurst & Jamieson, 1971	Lumbriculidae	microdrile
**Clitellata**	Oligochaeta	Moniligastrida Brinkhurst & Jamieson, 1971	Moniligastridae	megadrile
**Clitellata**	Oligochaeta	Narapida Timm, 2021	Narapidae	microdrile
**Clitellata**	Oligochaeta	Oligochaeta incertae sedis	Aeolosomatidae	microdrile
**Clitellata**	Oligochaeta	Oligochaeta incertae sedis	Hrabeiellidae	microdrile
**Clitellata**	Oligochaeta	Parvidrilida Timm, 2021	Parvidrilidae	microdrile
**Clitellata**	Oligochaeta	Randiellida Jamieson, 1988	Randiellidae	microdrile
**Clitellata**	Oligochaeta	Tubificida Jamieson, 1978	Phreodrilidae	microdrile
**Clitellata**	Oligochaeta	Tubificida Jamieson, 1978	Naididae sensu lato	microdrile
**Clitellata**	Oligochaeta	Tubificida Jamieson, 1978	Naididae, Naidinae	microdrile
**Clitellata**	Oligochaeta	Tubificida Jamieson, 1978	Naididae, Opistocystinae	microdrile
**Clitellata**	Oligochaeta	Tubificida Jamieson, 1978	Naididae, Pristininae	microdrile
**Clitellata**	Oligochaeta	Tubificida Jamieson, 1978	Naididae, Tubificinae	microdrile
**Clitellata**	Oligochaeta incertae sedis	Haplotaxida Brinkhurst & Jamieson, 1971	Tiguassidae	microdrile
**Oweniida**	Oweniida	Oweniida Dales, 1962	Magelonidae	polychaete
**Oweniida**	Oweniida	Oweniida Dales, 1962	Oweniidae	polychaete
**Polychaeta**	Errantia	Aciculata incertae sedis	Nerillidae	polychaete
**Polychaeta**	Errantia	Aciculata incertae sedis	Spintheridae	polychaete
**Polychaeta**	Errantia	Aciculata incertae sedis	Aberrantidae	polychaete
**Polychaeta**	Errantia	Aciculata incertae sedis	Myzostomida	polychaete
**Polychaeta**	Errantia	Eunicida Dales, 1962	Dorvilleidae	polychaete
**Polychaeta**	Errantia	Eunicida Dales, 1962	Eunicidae	polychaete
**Polychaeta**	Errantia	Eunicida Dales, 1962	Hartmaniellidae	polychaete
**Polychaeta**	Errantia	Eunicida Dales, 1962	Histriobdellidae	polychaete
**Polychaeta**	Errantia	Eunicida Dales, 1962	Lumbrineridae	polychaete
**Polychaeta**	Errantia	Eunicida Dales, 1962	Oenonidae	polychaete
**Polychaeta**	Errantia	Eunicida Dales, 1962	Onuphidae	polychaete
**Polychaeta**	Errantia	Phyllodocida Dales, 1962	Acoetidae	polychaete
**Polychaeta**	Errantia	Phyllodocida Dales, 1962	Aphroditidae	polychaete
**Polychaeta**	Errantia	Phyllodocida Dales, 1962	Eulepethidae	polychaete
**Polychaeta**	Errantia	Phyllodocida Dales, 1962	Glyceridae	polychaete
**Polychaeta**	Errantia	Phyllodocida Dales, 1962	Goniadidae	polychaete
**Polychaeta**	Errantia	Phyllodocida Dales, 1962	Hesionidae	polychaete
**Polychaeta**	Errantia	Phyllodocida Dales, 1962	Iospilidae	polychaete
**Polychaeta**	Errantia	Phyllodocida Dales, 1962	Iphionidae	polychaete
**Polychaeta**	Errantia	Phyllodocida Dales, 1962	Lacydoniidae	polychaete
**Polychaeta**	Errantia	Phyllodocida Dales, 1962	Lopadorrhynchidae	polychaete
**Polychaeta**	Errantia	Phyllodocida Dales, 1962	Microphthalmidae	polychaete
**Polychaeta**	Errantia	Phyllodocida Dales, 1962	Nephtyidae	polychaete
**Polychaeta**	Errantia	Phyllodocida Dales, 1962	Nereididae	polychaete
**Polychaeta**	Errantia	Phyllodocida Dales, 1962	Paralacydoniidae	polychaete
**Polychaeta**	Errantia	Phyllodocida Dales, 1962	Pilargidae	polychaete
**Polychaeta**	Errantia	Phyllodocida Dales, 1962	Polynoidae	polychaete
**Polychaeta**	Errantia	Phyllodocida Dales, 1962	Pontodoridae	polychaete
**Polychaeta**	Errantia	Phyllodocida Dales, 1962	Sphaerodoridae	polychaete
**Polychaeta**	Errantia	Phyllodocida Dales, 1962	Syllidae	polychaete
**Polychaeta**	Errantia	Phyllodocida Dales, 1962	Tomopteridae	polychaete
**Polychaeta**	Errantia	Phyllodocida Dales, 1962	Typhloscolecidae	polychaete
**Polychaeta**	Errantia	Phyllodocida Dales, 1962	Yndolaciidae	polychaete
**Polychaeta**	Errantia	Phyllodocida Dales, 1962	Chrysopetalidae, Calamyzinae	polychaete
**Polychaeta**	Errantia	Phyllodocida Dales, 1962	Chrysopetalidae sensu lato	polychaete
**Polychaeta**	Errantia	Phyllodocida Dales, 1962	Chrysopetalidae, Chrysopetalinae	polychaete
**Polychaeta**	Errantia	Phyllodocida Dales, 1962	Chrysopetalidae, Dysponetinae	polychaete
**Polychaeta**	Errantia	Phyllodocida Dales, 1962	Sigalionidae, Pelogeniinae	polychaete
**Polychaeta**	Errantia	Phyllodocida Dales, 1962	Sigalionidae, Pholoinae	polychaete
**Polychaeta**	Errantia	Phyllodocida Dales, 1962	Sigalionidae, Pisioninae	polychaete
**Polychaeta**	Errantia	Phyllodocida Dales, 1962	Sigalionidae sensu lato	polychaete
**Polychaeta**	Errantia	Phyllodocida Dales, 1962	Sigalionidae, Sigalioninae	polychaete
**Polychaeta**	Errantia	Phyllodocida Dales, 1962	Sigalionidae, Sthenelanellinae	polychaete
**Polychaeta**	Errantia	Phyllodocida Dales, 1962	Phyllodocidae, Eteoninae, Alciopini	polychaete
**Polychaeta**	Errantia	Phyllodocida Dales, 1962	Phyllodocidae	polychaete
**Polychaeta**	Errantia	Protodriliformia Struck et al., 2015	Polygordiidae	polychaete
**Polychaeta**	Errantia	Protodrilida, Pettibone, 1982	Protodrilidae	polychaete
**Polychaeta**	Errantia	Protodrilida Pettibone, 1982	Protodriloididae	polychaete
**Polychaeta**	Errantia	Protodrilida Pettibone, 1982	Saccocirridae	polychaete
**Polychaeta**	Polychaeta incertae sedis	Amphinomida Lamarck, 1818	Amphinomidae	polychaete
**Polychaeta**	Polychaeta incertae sedis	Amphinomida Lamarck, 1818	Euphrosinidae	polychaete
**Polychaeta**	Polychaeta incertae sedis	Sipuncula Stephen, 1964	Sipuncula, Aspidosiphonidae	sipunculan
**Polychaeta**	Polychaeta incertae sedis	Sipuncula Stephen, 1964	Sipuncula, Golfingiidae	sipunculan
**Polychaeta**	Polychaeta incertae sedis	Sipuncula Stephen, 1964	Sipuncula, Phascolosomatidae	sipunculan
**Polychaeta**	Polychaeta incertae sedis	Sipuncula Stephen, 1964	Sipuncula, Siphonosomatidae	sipunculan
**Polychaeta**	Polychaeta incertae sedis	Sipuncula Stephen, 1964	Sipuncula, Sipunculidae	sipunculan
**Polychaeta**	Polychaeta incertae sedis	Sipuncula Stephen, 1964	Sipuncula, Antillesomatidae	sipunculan
**Polychaeta**	Polychaeta incertae sedis	Sipuncula Stephen, 1964	Sipuncula, sensu lato	sipunculan
**Polychaeta**	Sedentaria	Capitellida Dales, 1962	Capitellidae	polychaete
**Polychaeta**	Sedentaria	Capitellida Dales, 1962	Opheliidae	polychaete
**Polychaeta**	Sedentaria	Capitellida Dales, 1962	Thalassematidae, Bonelliinae	echiuran
**Polychaeta**	Sedentaria	Capitellida Dales, 1962	Thalassematidae, Echiurinae	echiuran
**Polychaeta**	Sedentaria	Capitellida Dales, 1962	Thalassematidae, Ikedinae	echiuran
**Polychaeta**	Sedentaria	Capitellida Dales, 1962	Thalassematidae sensu lato	echiuran
**Polychaeta**	Sedentaria	Capitellida Dales, 1962	Thalassematidae, Thalassematinae	echiuran
**Polychaeta**	Sedentaria	Capitellida Dales, 1962	Thalassematidae, Urechinae	echiuran
**Polychaeta**	Sedentaria	Cirratulida Dales, 1962	Acrocirridae	polychaete
**Polychaeta**	Sedentaria	Cirratulida Dales, 1962	Cirratulidae	polychaete
**Polychaeta**	Sedentaria	Cirratulida Dales, 1962	Ctenodrilidae	polychaete
**Polychaeta**	Sedentaria	Cirratulida Dales, 1962	Flabelligeridae	polychaete
**Polychaeta**	Sedentaria	Cirratulida Dales, 1962	Longosomatidae	polychaete
**Polychaeta**	Sedentaria	Dinophiliformia Martin-Duran et al., 2021	Dinophilidae	polychaete
**Polychaeta**	Sedentaria	Dinophiliformia Martin-Duran et al., 2021	Lobatocerebridae	polychaete
**Polychaeta**	Sedentaria	Orbiniida Fauchald, 1977	Orbiniidae	polychaete
**Polychaeta**	Sedentaria	Orbiniida Fauchald, 1977	Parergodrilidae	polychaete
**Polychaeta**	Sedentaria	Sabellida Dales, 1962	Fabriciidae	polychaete
**Polychaeta**	Sedentaria	Sabellida Dales, 1962	Sabellidae	polychaete
**Polychaeta**	Sedentaria	Sabellida Dales, 1962	Serpulidae	polychaete
**Polychaeta**	Sedentaria	Sedentaria incertae sedis	Cossuridae	polychaete
**Polychaeta**	Sedentaria	Sedentaria incertae sedis	Diurodrilidae	polychaete
**Polychaeta**	Sedentaria	Spionida Dales, 1962	Poecilochaetidae	polychaete
**Polychaeta**	Sedentaria	Spionida Dales, 1962	Sabellariidae	polychaete
**Polychaeta**	Sedentaria	Spionida Dales, 1962	Spionidae	polychaete
**Polychaeta**	Sedentaria	Spionida Dales, 1962	Trochochaetidae	polychaete
**Polychaeta**	Sedentaria	Spionida Dales, 1962	Uncispionidae	polychaete
**Polychaeta**	Sedentaria	Sternaspida Fauchald, 1977	Fauveliopsidae	polychaete
**Polychaeta**	Sedentaria	Sternaspida Fauchald, 1977	Paraonidae	polychaete
**Polychaeta**	Sedentaria	Sternaspida Fauchald, 1977	Sternaspidae	polychaete
**Polychaeta**	Sedentaria	Sternaspida Fauchald, 1977	Siboglinidae, Frenulata	polychaete
**Polychaeta**	Sedentaria	Sternaspida Fauchald, 1977	Siboglinidae, Osedax	polychaete
**Polychaeta**	Sedentaria	Sternaspida Fauchald, 1977	Siboglinidae, Sclerolinum	polychaete
**Polychaeta**	Sedentaria	Sternaspida Fauchald, 1977	Siboglinidae sensu lato	polychaete
**Polychaeta**	Sedentaria	Sternaspida Fauchald, 1977	Siboglinidae, Vestimentifera	polychaete
**Polychaeta**	Sedentaria	Terebellida Dales, 1962	Alvinellidae	polychaete
**Polychaeta**	Sedentaria	Terebellida Dales, 1962	Ampharetidae	polychaete
**Polychaeta**	Sedentaria	Terebellida Dales, 1962	Arenicolidae	polychaete
**Polychaeta**	Sedentaria	Terebellida Dales, 1962	Maldanidae	polychaete
**Polychaeta**	Sedentaria	Terebellida Dales, 1962	Melinnidae	polychaete
**Polychaeta**	Sedentaria	Terebellida Dales, 1962	Pectinariidae	polychaete
**Polychaeta**	Sedentaria	Terebellida Dales, 1962	Trichobranchidae	polychaete
**Polychaeta**	Sedentaria	Terebellida Dales, 1962	Terebellidae, Terebellinae, Polycirrini	polychaete
**Polychaeta**	Sedentaria	Terebellida Dales, 1962	Scalibregmatidae, Scalibregmatinae	polychaete
**Polychaeta**	Sedentaria	Terebellida Dales, 1962	Terebellidae sensu lato	polychaete
**Polychaeta**	Sedentaria	Terebellida Dales, 1962	Terebellidae, Terebellinae ex Polycirrini	polychaete
**Polychaeta**	Sedentaria	Terebellida Dales, 1962	Terebellidae, Thelepodinae	polychaete
**Polychaeta**	Sedentaria	Terebellida Dales, 1962	Scalibregmatidae, Travisia	polychaete

### ﻿﻿Morphological characters and other traits

Morphological characters and character states (two or more) are described and illustrated in ANNiKEY Interactive; a text only version is provided in Suppl. material 2. The commonly used terms ‘character’ and ’character state’ have been referred to as ‘subject’ and ’predicate’ by a few polychaete annelid authors (e.g., Fitzhugh 2006; Nogueira et al. 2013), but we prefer to use the former nomenclature as used in DELTA. We refer to each character and state combination as a feature, as per DELTA, which appears to be the same concept as Fitzhugh’s (2006) ‘observation statement’. Features may or may not be homologous: the operation of the Intkey does not depend on the identified features being homologous between taxa. Thus, in the keys we have used a combination of binary and multistate characters and putatively homologous and non-homologous features, which provide optimal choice strategies that minimize the joint cost of errors and effort. We identified 288 morphological characters to score annelid family-level taxa (Suppl. material 2). Many characters are annotated with tips on how they can be used; the same characters are also illustrated in ANNiKEY Interactive. For both the linear and interactive keys, we used the default algorithm in DELTA commands TOKEY and TOINT, respectively, to select the most effective characters to use to differentiate taxa. The default algorithm favors keys with fewer couplets (shortest), and no penalty by the DELTA parameters was imposed for taxa to come out more than once in the key – this simply reflects family-level variability in the characters selected (the alternative is for taxa to appear only once and their features to be qualified as ‘usually’ or ‘rarely’). Further, the position of family taxa in the key does not necessarily reflect their phylogenetic proximity. Characters (ch.) considered difficult for the user to observe (under light microscopy) or interpret were largely excluded from the diagnosis (but included in other outputs including the full description and NEXUS file); for example, the musculature of the foregut (ch. 138), which can only be determined by histological sectioning, presence of microscopic lateral organs in polychaetes (ch. 191) and certain characters related to the segmental organs (ducts serving for both excretion/osmoregulation and gamete release; chs 291–299); the latter were assessed individually depending on the majorgroup as these characters are useful in identifying some annelids, particularly megadrile oligochaetes, but difficult or impossible to observe in small-bodied microdriles. Characters related to segmental organs and other internal characters were all included in the NEXUS file (Suppl. material 3) as they are often considered phylogenetically important (e.g., Bartolomaeus 1999), and they also appear in the full descriptions to make them as inclusive as possible.

For the linear key to be user-friendly and ‘familiar’ to annelid biologists, we manually constructed the first ‘couplet’ which leads to the majorannelid groups, then used the DELTA TOKEY command to generate keys within each of the majorgroups. We used minor character weighting (0–10, where 10 is the maximum weight) to force particular characters to appear early in their respective majorgroup sections and restrict others deemed difficult to interpret or visualize, from appearing in a couplet by themselves (Table 2). For example, characters 43–47, presence and forms of secondary annulation are important for leech identification but are misleading, or at least not well known, for other clitellates so was down-weighted for microdriles and megadriles.

**Table 2.** Character weights (= DELTA reliability scores; 0–10; 0 = not reliable; 10 = maximally reliable) for morphological characters for each of the majorannelid groups. Characters not mentioned were unweighted (5). Refer to Suppl. material 2 for the character list.

Informal taxon	Character reliability score
**Polychaetes, echiurans and sipunculans**	133,4 171,4 191,4 288,3
**Leeches**	42,10 288,2
**Megadriles**	43–47,2 70,3 223,8 265–267,8 317,8
**Microdriles**	43–47,2

For users unfamiliar with the differences between majorannelid groups or attempting to identify a morphological outlier taxon (e.g., an acanthobdellid leech which lacks the typical leech anterior sucker; or a polychaete or microdrile oligochaete lacking chaetae), we recommend using the online ANNiKEY Interactive rather than the linear key. ANNiKEY Interactive allows multiple access to morphological characters (character choice is left to the user), and as mentioned above, by being able to filter the starting taxa for an identification based on non-morphological characters – for example, environment (marine, freshwater, terrestrial) and biogeographic region (oligochaetes only), identification can often be used to reduce the number of possible family taxa. This is particularly useful for distinguishing between megadrile and microdrile oligochaetes, as they often display both environmental and geographic fidelity, although the high numbers of introduced species may be a complicating factor for some groups.

### ﻿﻿Morphological conventions, standards, and terminology

Most annelids have serially repeated units that comprise the body of a worm, which are often separated internally by septa. Unfortunately, how they have been designated and counted in Annelida has differed depending on the group. In polychaetes, Arabic numbers have mostly been used (S1, S2, S2 …; see exception below), but in leeches and oligochaetes roman numerals have been preferred (SI, SII, SIII ...). In polychaetes segment numbering starts after the presegmental prostomium and peristomium; in oligochaetes the peristomium is counted as the first segment, while in leeches the prostomium and peristomium have been counted as the first two segments. In the present study (and ANNiKEY Interactive), we have maintained, where possible, the historical numbering sequence of each group in order to facilitate comparisons with previous studies, i.e., the segment after the peristomium is referred to as segment 1 in polychaetes, segment II in oligochaetes, and segment III in leeches. Counting body segments is straightforward in polychaetes and oligochaetes that bear parapodia and/or chaetae; however, segment counting can be difficult when parapodia and chaetae are lacking (e.g., in leeches) or when there are pseudoannulations (most obvious in leeches, but also in other annelids) which resemble the annulations that mark true segments. Fortunately, all typical leeches have 34 segments (I–XXXIV; i.e., counting the preoral prostomium and peristomium as segments): six segments (I–VI) constitute the head, seven (XXVIII–XXXIV) constitute the caudal sucker, and the intervening 21 segments (VII–XXVII) constitute the midbody (after Sawyer 1986: 54–66). Counting segments in the leech-like Acanthobdellidae and Branchiobdellidae is similar (prostomium and peristomium counted as segments), but they have fewer segments than true leeches.

Unsurprisingly, there is an even greater diversity of morphological terminologies used between the majorannelid groups, with many examples of the same or very similar features of annelids being called by different names (e.g., capillary chaetae vs hair chaetae; hooks vs crotchets, and buccal tentacles vs oral filaments vs palps). The character list in Suppl. material 2 attempts to clarify some of these ‘terminological synonyms’, but for a more complete list, and for ease of searching for a particular term, the reader is referred to the companion ANNiKEY Online (Glasby et al. 2025b), which provides an illustrated synthesis of annelid morphological terms, including definitions, terms by character-type, terms by annelid group, and suggested abbreviations. The Glossary is also accessible within ANNiKEY Interactive.

## ﻿﻿Results

﻿This key is designed to work for adult specimens and, in the case of oligochaetes, sexually mature adults. Given the limitations of linear keys (discussed above), especially concerning taxon position in the key (the chance of an incorrect identification increases the further the taxon is toward the end of the key), we recommend that after reaching a family-level determination using the key below, the next step should be to compare the specimen at hand with the identified family in the Taxonomic accounts section; if specimen and description match, the user can have increased confidence in the identification.

### ﻿﻿Key to Annelida family-level taxa of the World

**Table:** 

1	Segmentation present; anus positioned at posterior end; anterior and posterior suckers absent; feeding appendages, when present, short proboscis or tentacular; clitellum absent; many chaetae (very rarely absent) [mostly marine; rarely freshwater or terrestrial]	**polychaetes (Polychaeta) 2**
–	Segmentation absent; anus positioned at posterior end; anterior and posterior suckers absent; single, long anterior feeding appendage present (ribbon-like); clitellum absent; chaetae, if present, few [marine]	**echiurans (Thalassematidae) 87**
–	Segmentation absent; anus positioned near anterior end; anterior and posterior suckers absent; single, long anterior feeding appendage present (robust proboscis); clitellum absent; chaetae absent [marine]	**sipunculans (Sipuncula) 90**
–	Segmentation present; anus positioned near posterior end (dorsal to the posterior sucker); anterior and posterior suckers present (anterior sucker rarely absent and replaced by adhesive mouth parts or chaetae); feeding appendages, when present, as short proboscis; clitellum present; chaetae absent (rarely present in first few segments) [terrestrial, freshwater, marine]	**leeches (Hirudinea) 94**
–	Segmentation present; anus positioned near posterior end; anterior and/or posterior suckers absent; feeding appendages absent; clitellum present, thick; chaetae usually few per segment (rarely more numerous), short, stout [mostly terrestrial]	**megadrile oligochaetes or earthworms (Crassiclitellata and Moniligastrida) 110**
–	Segmentation present; anus positioned near posterior end; anterior and/or posterior suckers absent; feeding appendages absent; clitellum present, thin; chaetae few, include both long hairs and/or short stout ones (rarely chaetae absent) [mostly freshwater or marine]	**microdrile oligochaetes and oligochaetoid polychaetes 130**
2(1)	Tentacular cirri absent	**3**
–	Tentacular cirri present	**63**
3(2)	Body shape elongate, more-or-less equal width along entire length; segmentation present (rarely absent)	**4**
–	Body shape widest anteriorly and tapering posteriorly; segmentation present	**44**
–	Body shape sausage- or grub-shaped; segmentation present (rarely absent)	**56**
–	Body shape ovate to elliptical; segmentation present (most easily visible on underside)	**62**
–	Body shape circular, flattened (Fig. 69D); segmentation absent (Fig. 69A, E–G); commensal on echinoderms	**Myzostomida**
–	Body shape peanut-shaped (spherical when contracted) (Fig. 131A); segmentation present	**Sternaspidae**
4(3)	Body regionalization absent	**5**
–	Body regionalization present, either two (e.g., thorax and abdomen) or three regions, usually demarcated by structural differences in parapodia along body	**29**
5(4)	Ventral cirri absent	**6**
–	Ventral cirri present	**22**
6(5)	Palps absent	**7**
–	Palps present	**13**
7(6)	Body segmentation present; nuchal organs present; parapodia present; chaetae present	**8**
–	Body segmentation absent; nuchal organs absent; parapodia absent; chaetae absent	**12**
8(7)	Prostomium conical, tapering to slender tip (palpode), otherwise head appendages absent (Fig. 81A)	**Opheliidae**
–	Prostomium rounded to oval, head appendages present (Fig. 29B)	**Dorvilleidae**
–	Prostomium narrow, keel- or ridge-shaped, often with a rimmed bordered (Fig. 63B); head appendages absent	**Maldanidae**
–	Prostomium bluntly conical (typical); head appendages present or absent	**9**
9(8)	1^st^ chaetiger with notochaetae only; single long mid-dorsal filament (branchia) on anterior chaetiger (Fig. 24A)	**Cossuridae**
–	1^st^ chaetiger with neurochaetae only (Fig. 60A); mid-dorsal branchia absent	**Lumbrineridae**
–	1^st^ chaetiger with both notochaetae and neurochaetae; mid-dorsal branchia absent	**10**
10(9)	Head not retractable; parapodia with interramal papilla absent	**11**
–	Head retractable into anterior segments; parapodia with interramal papilla present (Fig. 38D, E)	**Fauveliopsidae**
11(10)	Eyes on head absent; pharynx dorsolateral ciliated folds absent; biramous parapodia absent or very low; dorsal cirri absent	**Ctenodrilidae**
–	Eyes on head present; pharynx dorsolateral ciliated folds present; biramous parapodia prominent; dorsal cirri present	**Oenonidae**
12(7)	Body pigmentation absent; anus positioned at posterior body; pygidium present	**Diurodrilidae**
–	Body pigmentation present; anus subterminal; pygidium absent	**Lobatocerebridae**
13(6)	Prostomium conical, tapering to slender tip; branchiae present, arise from lateral or dorsal body anywhere along body	**14**
–	Prostomium bluntly conical; branchiae present or absent	**15**
–	Prostomium triangular to trapezoidal (narrow end posteriorly); branchiae present anteriorly or on midbody	**19**
–	Prostomium rounded to oval; branchiae present or absent	**20**
–	Prostomium narrow, keel- or ridge-shaped (Fig. 5B, pr); branchiae present (but easily detached in preserved specimen; Fig. 5A–C)	**Acrocirridae**
–	Prostomium T-shaped, wide end anteriorly (Fig. 130A); branchiae present (Fig. 130A, B, D)	**Spionidae**
–	Prostomium flattened, shovel-shaped (Fig. 62B, pr); branchiae absent	**Magelonidae**
14(13)	Head lobe-like without appendages; nuchal organs paired low projections from posterolateral prostomium; peristomium a single ring (Fig. 23A); pygidial appendages absent (Fig. 23C)	**Cirratulidae**
–	Head bearing appendages; nuchal organs single antenna-like projection from posterior prostomium; peristomium collar-like (Fig. 130A); pygidial appendages present	**Spionidae**
15(13)	Palps anterodorsal; obvious biramous parapodia	**16**
–	Palps anteroventral; parapodia uniramous (reduced) or absent	**17**
–	Palps frontal; parapodia absent	**18**
16(15)	Epidermis more-or-less smooth; head not retractable, without appendages; numerous, unpaired eyes	**Cirratulidae**
–	Epidermis papillated; head retractable into anterior segments, bearing appendages; two pairs of eyes	**Flabelligeridae**
17(15)	Parapodia present; 1^st^ and 2^nd^ segments chaetous (as are all following segments) (Fig. 109); dorsal body surface smooth	**Saccocirridae**
–	Parapodia absent; 1^st^ and 2^nd^ segments achaetous (as are all following segments) (Fig. 102A–C); dorsal body surface ciliated	**Protodrilidae**
18(15)	Epidermal glands absent (Fig. 97A); pygidium simple lobe although subterminally inflated and appearing bulb-shaped (bulb adorned with a ring of papilla-sized adhesive glands) (Fig. 97A, C); macrobiotic size (2.0–200 mm)	**Polygordiidae**
–	Epidermal glands present (Fig. 103A) pygidium bilobed (Fig. 103A); meiobiotic size (0.2–2.0 mm)	**Protodriloididae**
19(13)	Parapodia of 1^st^ chaetiger similar in length or slightly shorter than subsequent parapodia, more-or-less laterally directed and free from head; chaetae of 1^st^ chaetiger similar in orientation, length, and thickness to other chaetae; nuchal organs single antenna-like projection from posterior prostomium (caruncle) (Fig. 130A)	**Spionidae**
–	Parapodia of 1^st^ chaetiger very elongated, anteriorly directed and wrapping around head; chaetae of 1^st^ chaetiger slender and elongate, forming cage (or basket) around head (Fig. 149A, C); nuchal organs paired low projections from posterolateral prostomium (Fig. 149A)	**Uncispionidae**
20(13)	Epidermis more-or-less smooth; head not retractable; 2^nd^ segment chaetous; circulatory system present	**21**
–	Epidermis papillated; head retractable into anterior segments (Fig. 5C); 2^nd^ segment achaetous; circulatory system absent	**Acrocirridae**
21(20)	Prostomial antenna median only; facial tubercle present (Fig. 96A); palps anterodorsal (Fig. 96A); 1^st^ chaetiger with both notochaetae and neurochaetae	**Poecilochaetidae**
–	Prostomial antennae paired, lateral (Fig. 29B); facial tubercle absent; palps anteroventral (Fig. 29B); 1^st^ chaetiger with neurochaetae only	**Dorvilleidae**
22(5)	Prostomium elongate-conical, tapering to slender tip; often annulated with four identical small cirriform projections (palps and antennae) arising distally	**23**
–	Prostomium bluntly conical, not annulated, antennae and/or palps present, arising distally or from near base of prostomium	**24**
–	Prostomium rounded to oval, not annulated, antennae and/or palps present arising from near base of prostomium	**25**
–	Prostomium triangular to trapezoidal (narrow end posteriorly) (Fig. 11B); not annulated, antennae and/or palps present arising from near base of prostomium (Fig. 11B)	**Amphinomidae**
23(22)	Pharynx bearing two pairs of jaws (Fig. 42A, B)	**Glyceridae**
–	Pharynx bearing multiple jaw elements of different shapes and sizes (Fig. 43A, B)	**Goniadidae**
24(22)	Prostomial antennae absent; palps grooved, feeding type, anterodorsal, long (Fig. 13C); pygidial appendages four cirri (Fig. 13B)	**Apistobranchidae**
–	Prostomial antennae present; palps tapering, sensory type, anteroventral, both short (Fig. 86C); pygidial appendages one pair of cirri and single medial papilla (Fig. 86B)	**Paralacydoniidae**
25(22)	Palps anterodorsal; small forms epizoic on crustaceans, or large free-living forms	**26**
–	Palps anteroventral; free-living forms	**27**
26(25)	Body segment number fixed at ~ 9–13; in life, body translucent, gut visible; prostomium without deep incision anteriorly; pygidium deeply cleft forming two large feet or posterior locomotory appendages; peristomium a single ring bearing paired cirri (anterior locomotory appendages; Fig. 49A)	**Histriobdellidae**
–	Body segment number variable; in life, body opaque, gut usually not visible; prostomium anteriorly incised or indented (Fig. 35A); peristomium a double ring (Fig. 35A); pygidium simple lobe	**Eunicidae**
27(25)	Palps tapering (usually) sensory type; 1^st^ segment chaetous bearing neurochaetae only; parapodia uniramous	**28**
–	Palps grooved (usually) feeding type; 1^st^ segment achaetous, tri-annulate (Fig. 1A); 1^st^ chaetiger with both notochaetae and neurochaetae; parapodia biramous (Fig. 1C)	**Aberrantidae**
28(27)	Prostomium anteriorly without deep incision; prostomial antennae present (rarely absent), paired, lateral; frontal lips absent; peristomium a double ring (Fig. 29B)	**Dorvilleidae**
–	Prostomium anteriorly incised; prostomial antennae include median and paired laterals; frontal lips present (Fig. 80); peristomium a single ring	**Onuphidae**
29(4)	Neuropodial lobes represented by at least one chaetal lobe; coastal to deep sea, rarely freshwater	**30**
–	Neuropodial lobes as low ridges (tori); coastal to deep sea, rarely freshwater	**38**
–	Neuropodial lobes absent; oceanic, usually deep sea or shelf	**42**
30(29)	Pygidial appendages absent; radiolar crown usually present, if absent then body and its segments extremely long	**31**
–	Pygidial appendages (including cirri and/or papillae) present; radiolar crown absent, body and segments not overly long	**34**
31(30)	Fecal groove absent; small-bodied fan worms with radiolar crown bearing a pair of ventral filamentous appendages, or non-tubicolous worm	**32**
–	Fecal groove present (Fig. 108A); typical tube-dwelling fan or feather duster worms lacking paired ventral filamentous appendages on radiolar crown	**33**
32(31)	Body segments strongly elongate in midbody (Fig. 56A); eyes on head absent; palps present; 1^st^ chaetiger with both notochaetae and neurochaetae (Fig. 56C)	**Longosomatidae**
–	Body segments similar dimensions throughout (Fig. 37F); eyes on head present (one pair on peristomium) (Fig. 37F); palps absent; 1^st^ chaetiger with notochaetae only	**Fabriciidae**
33(31)	Radiolar crown bearing a single (rarely double or more) peduncular operculum (very rarely absent; Fig. 114); tube hard, calcareous	**Serpulidae**
–	Radiolar crown without peduncular operculum (Fig. 108A, B); tube soft, leathery, or membranous	**Sabellidae**
34(30)	Biramous parapodia absent or very low	**35**
–	Biramous parapodia prominent	**37**
35(34)	Ventral groove absent; nuchal organs indistinct as ciliated patches; branchiae arise from dorsal body; spines present	**36**
–	Ventral groove present (Fig. 81A); nuchal organs as posterior prostomial projections; branchiae arise from lateral body (Fig. 81A, F); spines absent	**Opheliidae**
36(35)	Body regionalization comprising two regions; capillary chaetae, internally chambered; forked chaetae tines more-or-less equal in length; hooks without distal hood, beard, or ligament (Fig. 82E–K)	**Orbiniidae**
–	Body regionalization comprising three regions; capillary chaetae not chambered or hollow; forked chaetae tines distinctly unequal in length; hooks with a distal hood (Fig. 87E–M)	**Paraonidae**
37(34)	Body regions demarcated by structural differences in parapodia along body; prostomium triangular to trapezoidal (narrow end posteriorly); palps present (Fig. 146A, E); ventral cirri absent	**Trochochaetidae**
–	Body regions demarcated by laterally-directed thoracic parapodia and dorsally-directed midbody and abdominal parapodia; prostomium rounded to oval; palps absent (Fig. 46A); ventral cirri present	**Hartmaniellidae**
38(29)	Body regionalized; regions demarcated by change in chaetal types on body; radiolar crown absent	**39**
–	Body regionalized; regions demarcated by structural differences in parapodia on body; radiolar crown absent	**40**
–	Body regionalized; regions demarcated by inversion of parapodia; radiolar crown present	**41**
39(38)	Body segments similar dimensions throughout; prostomium bluntly conical (Fig. 18); nuchal organs present; pharynx dorsolateral ciliated folds absent	**Capitellidae**
–	Body segments strongly elongate in midbody; prostomium rounded to oval (Fig. 84, A, E–G); nuchal organs absent; pharynx dorsolateral ciliated folds present	**Oweniidae**
40(38)	Body regionalization comprising three regions; palps present (Fig. 19A, F); macrobiotic size	**Chaetopteridae**
–	Body regionalization comprising two regions; ; palps absent (Fig. 104A); meiobiotic size	**Psammodrilidae**
41(38)	Radiolar crown bearing a single (rarely double or more) peduncular operculum (Fig. 114A, B)	**Serpulidae**
–	Radiolar crown without peduncular operculum or paired ventral filamentous appendages (Fig. 108A, B)	**Sabellidae**
–	Radiolar crown bearing a pair of ventral filamentous appendages (Fig. 37F)	**Fabriciidae**
42(29)	Caudal region with rows of uncini on each segment; not inhabiting environments below	**43**
–	Caudal region with four peg-like chaetae in most segments; lives in deep-sea reducing sediments	**Siboglinidae, Frenulata**
–	Caudal region with long-handled hooks; lives in deep sea on sunken bones of vertebrates	**Siboglinidae, *Osedax***
43(42)	Body pigmentation absent; buccal tentacles present (Fig. 116A, B), smooth; peristomium a single ring; tube plug absent; lives in deep sea on sunken plant material	**Siboglinidae, *Sclerolinum***
–	Body pigmentation present; buccal tentacles present as a branchial plume (Fig. 117C), pinnulate; peristomium expanded, elaborately collared ring (operculum or obturaculum) present (Fig. 117C); tube plug present; lives on hard substrates at hydrothermal vents and cold seeps	**Siboglinidae, Vestimentifera**
44(3)	Chaetae first appear on 1^st^ segment after peristomium; buccal tentacles absent; operculum present	**45**
–	Chaetae first appear on 2^nd^ segment after peristomium; buccal tentacles present or absent; operculum absent	**48**
–	Chaetae first appear on 3^rd^ segment after peristomium; buccal tentacles present; operculum absent	**50**
–	Chaetae first appear on 4^th^ segment after peristomium; buccal tentacles present; operculum absent	**53**
–	Chaetae first appear on 5^th^ or 6^th^ segment after peristomium; buccal tentacles present (Fig. 144A, B); operculum absent	**Trichobranchidae**
45(44)	Caudal region comprising unmodified typical segments (but shorter and with reduced parapodia compared to anterior ones); head lacking an operculum	**46**
–	Caudal region an unsegmented tube; paleate operculum present (Fig. 107A)	**Sabellariidae**
–	Caudal region short, few segments, mostly achaetous, with frilly lobes (Fig. 90A); operculum with golden cephalic paleae (Fig. 90A)	**Pectinariidae**
46(45)	Head lobe-like without appendages; prostomium conical, tapering to slender tip (Fig. 112A); palps absent; forked chaetae absent	**47**
–	Head bearing appendages; prostomium T-shaped, wide end anteriorly (Fig. 111A, B, D); palps present; forked chaetae present	**Scalibregmatidae, Scalibregmatinae**
47(46)	Epidermis thick and rugose or papillate (Fig. 112A, C); pharynx dorsolateral ciliated folds absent	**Scalibregmatidae, *Travisia***
–	Epidermis more-or-less smooth (Fig. 81A, C); pharynx dorsolateral ciliated folds present	**Opheliidae**
48(44)	Body regions demarcated by structural differences in parapodia along body, caudally, with a prominent achaetous sacrificial tail (Fig. 14A); prostomium bluntly conical; 1^st^ chaetiger with both notochaetae and neurochaetae; pygidial appendages absent	**Arenicolidae**
–	Body regions demarcated by absence of abdominal notopodia, without an achaetous caudal region; prostomium hood-like, covering the tentacles dorsally; 1^st^ chaetiger with notochaetae only; pygidial appendages present	**49**
49(48)	Thoracic collar-like dorsolateral expansion absent (Fig. 10A); spines in dorsal (notopodial) position	**Ampharetidae**
–	Thoracic collar-like dorsolateral expansion present (Fig. 65A); spines in ventral (neuropodial) position	**Melinnidae**
50(44)	Discrete head absent; buccal tentacles arise on one side of mouth; peristomium expanded into well-developed upper and lower lips; spines in ventral (neuropodial) position	**51**
–	Discrete head present; buccal tentacles arise inside mouth; peristomium a single ring; spines in dorsal (notopodial) position	**52**
51(50)	Spines, when present, more-or-less straight and smooth, present in mid and posterior neuropodia (Fig. 134G); hooks absent; tube absent	**Terebellidae, Terebellinae, Polycirrini**
–	Spines, when present, sharply bent (= geniculate) or recurved, present only in one or a few anterior neuropodia (Fig. 144F); hooks present (Fig. 144G); tube present	**Trichobranchidae**
52(50)	Body regionalization absent; nuchal organs absent; pygidial appendages absent; spines slightly curved and more-or-less smooth	**Alvinellidae**
–	Body regionalization present; nuchal organs present; pygidial appendages present; spines sharply bent (= geniculate) or recurved	**Ampharetidae**
53(44)	Thoracic hooks absent; lower lip obvious but not expanded	**54**
–	Thoracic hooks present; lower lip often greatly expanded (Fig. 144)	**Trichobranchidae**
54(53)	Thoracic ventral glandular area with distinct mid-ventral shield-shaped shields (Fig. 136A); branchiae present	**55**
–	Thoracic ventral glandular area with distinct paired ventrolateral pads (Fig. 134C); branchiae absent	**Terebellidae, Terebellinae, Polycirrini**
55(54)	Uncini arranged in one row	**Terebellidae,Thelepodinae**
–	At least some uncini arranged in two rows	**Terebellidae, Terebellinae (excl. Polycirrini)**
56(3)	Prostomium bluntly conical; papillated body surface, though papillae may be restricted to parapodia (single interramal papilla in the extreme)	**57**
–	Prostomium rounded to oval; body surface smooth in meiofauna-sized forms and pelagic forms, papillated in the macrofaunal benthic forms	**58**
–	Prostomium narrow, keel- or ridge-shaped (Fig. 5B); papillae present on parts of the body (Fig. 5B); may have adhering sediment grains	**Acrocirridae**
–	Prostomium T-shaped, wide end anteriorly (Fig. 111B); not papillated (although epidermis may be thick and rugose) (Fig. 111B, D, H)	**Scalibregmatidae, Scalibregmatinae**
57(56)	Body pigmentation absent; palps absent; chaetae first appear on 1^st^ segment after peristomium (Fig. 38A); capillary chaetae not appearing barred	**Fauveliopsidae**
–	Body pigmentation usually present (Fig. 39G); palps present; chaetae first appear on 3^rd^ segment after peristomium; capillary chaetae appearing barred (pseudosegmented) (Fig. 39D, E)	**Flabelligeridae**
58(56)	Head lobe-like without appendages; parapodia absent	**59**
–	Head bearing appendages; parapodia present	**61**
59(58)	Body segmentation present; pharynx dorsolateral ciliated folds absent	**60**
–	Body segmentation absent, except for indistinct creases in the body wall (Fig. 28A); pharynx dorsolateral ciliated folds present	**Diurodrilidae**
60(59)	Chaetae absent (Fig. 27A, B); gut more-or-less straight, lacking side branches	**Dinophilidae**
–	Chaetae present; 2^nd^ and subsequent segments chaetous (Fig. 88A, D); gut straight except for a large mid-body loop	**Parergodrilidae**
61(58)	Peristomium not visible (prostomium merges into 1^st^ chaetiger); pygidial appendages present (Fig. 77A–G); compound chaetae appendage without hoods or guards (Fig. 77H)	**Nerillidae**
–	Peristomium as a single ring; pygidial appendages absent (Fig. 5A, B); compound chaetae appendage with a single hood open in front	**Acrocirridae**
–	Peristomium as a double ring (Fig. 29B); pygidial appendages present or absent; compound chaetae appendage with paired guards on each side of the crest	**Dorvilleidae**
62(3)	Prostomium triangular to trapezoidal (narrow end posteriorly); caruncle present (Fig. 11B); notopodia represented by at least one chaetal lobe, tufted branchiae	**Amphinomidae**
–	Prostomium rounded to oval; caruncle absent; notopodia represented by radial or transverse dorsal ridges, without branchiae (Fig. 129A)	**Spintheridae**
–	Prostomium narrow, keel- or ridge-shaped; caruncle present; notopodia represented by long dorsal ridges bearing branchiae (Fig. 36B)	**Euphrosinidae**
63(2)	Dorsal body surface with protective covering absent	**64**
–	Dorsal body surface with protective covering of scales (elytrae) [scaleworms]	**80**
–	Dorsal body surface with protective covering of shield-like spines (paleae) [includes golden petal worms]	**86**
64(63)	Prostomium conical, tapering to slender tip (prostomium may be reduced; Fig. 119A, D–F); parapodia biramous but reduced, dorsal cirri small, articulated and flask-shaped (Fig. 119B); benthic forms only	**Sigalionidae, Pisioninae**
–	Prostomium bluntly conical (includes inverted T-shaped; Fig. 76A–D); parapodia and dorsal cirri well-developed (uniramous or biramous); benthic and pelagic forms	**65**
–	Prostomium pentagonal to quadrangular; parapodia and dorsal cirri well-developed (uniramous or biramous); benthic forms only	**67**
–	Prostomium rounded to oval; parapodia (uniramous or biramous) and dorsal cirri well-developed (rarely reduced); benthic and pelagic forms	**69**
65(64)	In life, body translucent, gut visible; eyes on head absent; palps absent (Fig. 148A, B); compound chaetae absent; all holopelagic	**Typhloscolecidae**
–	In life, body opaque, gut usually not visible; eyes on head present; palps present; compound chaetae present; benthic, apart from sexually-mature reproductive forms which may be pelagic	**66**
66(65)	Eyes one pair; palps unarticulated; pharynx jaws absent; distal ring of papillae present (Fig. 92A–C)	**Phyllodocidae sensu lato**
–	Eyes two pairs; palps bi-articulated; pharynx jaws present (Fig. 76A–D); distal ring of papillae absent	**Nereididae**
67(64)	Head lobe-like without appendages (prostomial antennae absent); palps absent (Fig. 151A); capillary chaetae absent; all holopelagic	**Yndolaciidae**
–	Head bearing appendages (prostomial antennae); palps present; capillary chaetae present; benthic, apart from sexually-mature reproductive forms which may be pelagic	**68**
68(67)	Prostomial antennae include median and paired laterals (Fig. 132A, B); proventricle present (Fig. 132A); chaetae first appear on 2^nd^ segment after peristomium; 1^st^ chaetiger with neurochaetae only	**Syllidae**
–	Prostomial antennae paired, laterals only; proventricle absent (Fig. 75A, B); chaetae first appear on 1^st^ segment after peristomium; 1^st^ chaetiger with both notochaetae and neurochaetae	**Nephtyidae**
69(64)	Notopodial lobes represented by at least one chaetal lobe; benthic forms	**70**
–	Notopodial lobes absent; benthic and holopelagic forms	**73**
–	Notopodial lobes elongate, ending in rounded lappet (Fig. 143A–C); holopelagic	**Tomopteridae**
70(69)	1^st^ chaetiger with neurochaetae only; capillary chaetae present	**71**
–	1^st^ chaetiger with both notochaetae and neurochaetae; capillary chaetae absent (Fig. 20A)	**Chrysopetalidae, Calamyzinae**
71(70)	Tentacular cirri arise on a single segment, internal aciculae absent; 2^nd^ segment chaetous	**72**
–	Tentacular cirri rise on two or more segments, internal aciculae present in at least some cirri; 2^nd^ segment achaetous (Fig. 47A, B)	**Hesionidae**
72(71)	In life, body translucent, gut visible; pygidial appendages one pair of cirri and single medial papilla (Fig. 55D); dorsal cirri flattened and foliaceous (Fig. 55E); compound chaetae present	**Lacydoniidae**
–	In life, body opaque, gut usually not visible; pygidial appendages one pair of cirri; dorsal cirri more-or-less cirriform (Fig. 94C–F); compound chaetae absent	**Pilargidae**
73(69)	Eyes, one pair; benthic and holopelagic	**74**
–	Eyes, two pairs; benthic	**78**
–	Eyes, three pairs; benthic (non-reproductive individuals)	**Syllidae**
74(73)	Dorsal cirri more-or-less cirriform; holopelagic or benthic	**75**
–	Dorsal cirri flattened and foliaceous; holopelagic	**76**
75(74)	In life, body translucent, gut visible; 1^st^ and subsequent chaetigers with neurochaetae only; pygidium simple lobe (Fig. 99A, B); pharynx with papillae in subterminal position; holopelagic	**Pontodoridae**
–	In life, body opaque, gut usually not visible; 1^st^ chaetiger with both notochaetae and neurochaetae; pygidium membranous anal plate (Fig. 67A, B); pharynx smooth; benthic	**Microphthalmidae**
76(74)	Body shape dorsoventrally flattened; dorsal cirri digitate or slender and leaf-like (Fig. 57A–C)	**Lopadorrhynchidae**
–	Body shape more-or-less cylindrical; dorsal cirri flattened and foliaceous	**77**
77(76)	Head lobe-like without appendages; prostomial antennae absent; pygidial appendages absent (Fig. 52A, B, D); capillary chaetae hirsute-serrate	**Iospilidae**
–	Head bearing appendages; prostomial antennae present (Fig. 93A, B); pygidial appendages present; capillary chaetae smooth	**Phyllodocidae, Eteoninae, Alciopini**
78(73)	Tentacular cirri rise on two or more segments with internal aciculae present in at least some cirri; 2^nd^ segment achaetous (Fig. 47A, B); proventricle absent	**Hesionidae**
–	Tentacular cirri arise on a single segment with internal aciculae absent; 2^nd^ segment chaetous; proventricle present	**79**
79(78)	Capillary chaetae absent	**Sphaerodoridae**
–	Capillary chaetae present	**Syllidae**
80(63)	Elytra with raised concentric rings (Fig. 118B); facial tubercle present; notopodial silky (feltage) chaetae absent	**Sigalionidae, Pholoinae**
–	Elytra smooth with lateral pouches (Fig. 4C); facial tubercle present; notopodial silky (feltage) chaetae present (may be incorporated into tube) (Fig. 4C, G)	**Acoetidae**
–	Elytra with a tuberculated pentagonal or hexagonal pattern (Fig. 53B); facial tubercle absent; notopdial silky (feltage) chaetae absent	**Iphionidae**
–	Elytra with papillae, tubercles or smooth; facial tubercle present or absent; notopodial silky (feltage) chaetae present or absent	**81**
81(80)	Body shape dorsoventrally flattened; spines present; compound chaetae absent	**82**
–	Body shape more-or-less cylindrical; spines absent; compound chaetae present	**84**
82(81)	Spines in dorsal (notopodial) position only (Fig. 34D)	**Eulepethidae**
–	Spines in ventral (neuropodial) position only (Fig. 4C)	**Acoetidae**
–	Spines in both dorsal and ventral positions	**83**
83(82)	Prostomium anteriorly not incised, with median antenna only (may be very small); ommatophores present (Fig. 12A, B); paired jaws plate-like; venter always papillated	**Aphroditidae**
–	Prostomium anteriorly incised, usually with median antenna and paired lateral antennae (antennae may be absent in deep-sea taxa) (Fig. 98A, B); ommatophores absent; paired jaws fang-like; venter rarely papillated	**Polynoidae**
84(81)	Dorsal cirri absent	**Sigalionidae, Pelogeniinae**
–	Dorsal cirri present	**85**
85(84)	Body often pigmented; silky chaetae absent; tube absent	**Sigalionidae, Sigalioninae**
–	Body unpigmented; silky chaetae (feltage) may be present (but only in notopodia of chaetiger 2); tube present	**Sigalionidae, Sthenelanellinae**
86(63)	Nuchal organs paired low projections from posterolateral prostomium; paleate chaetae absent; spines in dorsal (notopodial) position only (Fig. 22A, C)	**Chrysopetalidae, Dysponetinae**
–	Nuchal organs as an unpaired caruncle or nuchal fold; paleate chaetae present (Fig. 21A, B, D); spines in both dorsal and ventral positions	**Chrysopetalidae, Chrysopetalinae**
87(1)	Chaetae only present anteriorly; spines absent	**88**
–	Chaetae present anteriorly and posteriorly; spines present (Fig. 138A, B)	**Thalassematidae, Echiurinae**
88(87)	Proboscis short and scoop-like (Fig. 141A)	**Thalassematidae, Urechinae**
–	Proboscis very long (longer than trunk)	89
89(88)	Proboscis truncate distally (Figs 139A–C, 140A–C)	**Thalassematidae (Ikedinae and Thalassematinae)**
–	Proboscis usually bifid distally (Fig. 137A)	**Thalassematidae, Bonelliinae**
90(1)	Buccal tentacles (when expanded) arising from one side of mouth	**91**
–	Buccal tentacles (when expanded) encircling mouth	**92**
91(90)	Introvert shorter than trunk (Fig. 124A)	**Sipuncula, Antillesomatidae**
–	Introvert about equal in length to trunk (Fig. 123A)	**Sipuncula, Phascolosomatidae**
–	Introvert longer than trunk (Figs 120A, F, 123D)	**Sipuncula, Aspidosiphonidae, Sipuncula, Phascolosomatidae**
92(90)	Introvert papillae absent; tentacles arising from a stem-like extension of oral disc or directly from oral disc (Fig. 121B, C, E)	**Sipuncula, Golfingiidae**
–	Introvert papillae present; tentacles arising from a tentacular fold or directly from oral disc (Fig. 126C)	**93**
93(92)	Trunk smooth (Fig. 125)	**Sipuncula, Siphonosomatidae**
–	Trunk roughened by papillae or rounded skin bodies (Fig. 126B, D)	**Sipuncula, Sipunculidae**
94(1)	Body consists of 15 segments; adhesive mouth parts for attaching to their host (Fig. 16A)	**Branchiobdellidae**
–	Body consists of 31 segments including two pre-oral ‘segments’ (prostomium and peristomium) and 29 post-oral segments; four pairs of hooked chaetae for attaching to their host (Fig. 2B, C)	**Acanthobdellidae**
–	Body consists of 34 segments including two pre-oral ‘segments’ (prostomium and peristomium) and 32 post-oral segments; anterior sucker for attaching to their host [true leeches]	**95**
95(94)	Elongate proboscis absent; sucker with large mouth; jaws usually present (gnathous); circulatory system absent	**96**
–	Elongate proboscis present; sucker with small mouth pore; jaws absent (agnathous); circulatory system present	**108**
96(95)	Muscular axial pharynx ridges rotated 60° to the right (strepsilaematous)	**97**
–	Muscular axial pharynx ridges not rotated (euthylaematous) [jawed leeches, ‘Gnathobdellidae’]	**98**
97(96)	Mid-body segments 6-annulate; testes, only a few pairs; aquatic, widespread, but absent from Americas and Antarctic	**Salifidae**
–	Mid-body segments 5-annulate or 8-annulate or more; testes, many pairs; terrestrial or aquatic [jawless ‘Arhynchobdellidae’]	**106**
98(96)	Body shape dorsoventrally flattened; anterior sucker lacking lateral cirri	**99**
–	Body shape more-or-less cylindrical; anterior sucker with pair of short lateral cirri (sensory palps) (Fig. 150B); native to Neotropics and Palaearctic	**Xerobdellidae**
99(98)	Midgut caecae absent to a few; raptorial (predatory)	**100**
–	Midgut caecae many (10–12 pairs) with an extra pair in hindgut; haematophagous	**104**
100(99)	Eyes absent or if present 3–5 pairs; shared oviduct from egg sac	**101**
–	Eyes usually present, 5 pairs; oviduct separate one for each egg sac	**103**
101(100)	Eyes on head absent; egg sacs tubular; native to Palaearctic and Indo-Malay region	**Gastrostomobdellidae**
–	Eyes on head present; egg sacs globular	**102**
102(101)	Head eyes, 1–3 pairs (usually three pairs arranged on two separate segments) (Fig. 83D); caeca of midgut absent; testes in multiple grape-like clusters per segment; male and female pores separated by 5 or 6 annuli (Fig. 83B); free-living on soft substrata; native to East Asia	**Orobdellidae**
–	Head eyes, usually 5 pairs (arranged in an arc on segments II–VI, with 3^rd^ and 4^th^ pairs separated by one annulus) (Fig. 48B); caeca of midgut present; testes, one pair per segment; male and female pores separated by 3–5 annuli (Fig. 48B); epizoic	**Hirudinidae**
103(100)	Vaginal sac absent; egg sacs tubular; penis unknown; native to Neotropics; raptorial	**Cyclobdellidae**
–	Vaginal sac present; egg sacs globular; penis present, recurved; male and female pores separated by 3–5 annuli (Fig. 48B); epizoic, widespread	**Hirudinidae**
–	Vaginal sac present (Fig. 113E); egg sacs globular; penis present, with a hardened sheath (Fig. 113C); male and female pores separated by 5–8 annuli (Fig. 113C); raptorial	**Semiscolecidae**
104(99)	Epidermis more-or-less smooth; male and female pores separated by 3–5 annuli (Fig. 48B); widespread	**Hirudinidae**
–	Epidermis tessellated (Fig. 44C, D); male and female pores separated by 3–9 annuli (Fig. 44C); widespread in tropics and Southern Hemisphere	**Haemadipsidae**
–	Epidermis papillate	**105**
105(104)	Jaws with one row of teeth; male and female pores separated by 3–5 annuli (Fig. 48B); widespread, including Australia	**Hirudinidae**
–	Jaws with two rows of teeth, or a series of teeth or cutting plates (Fig. 100); male and female pores separated by 5 annuli (Fig. 100A); widespread, excluding Australia	**Praobdellidae**
106(97)	Testes, one pair per segment	**107**
–	Testes in multiple grape-like clusters per segment; most common in Nearctic, Palaearctic and Neotropics in moist terrestrial	**Erpobdellidae**
–	Testes, two pairs per segment (tetrad arrangement); aquatic, widespread, but absent from Americas and Antarctic	**Salifidae**
107(106)	Caeca of midgut absent; male atrium bilobed, anteriorly directed, deeply cleft with cornua; penis absent; native to Neotropics	**Cylicobdellidae**
–	Caeca of midgut present; male atrium fused comprising a dorsal prostate chamber and a ventral penile sac; penis present, short, conical; native to Neotropics	**Americobdellidae**
108(95)	Body regionalization absent (Fig. 40A); gonadal segments bearing a sperm transfer system in copulatory area absent; most records from Nearctic, Palaearctic and Neotropics	**Glossiphoniidae**
–	Body regionalization present (Fig. 85A); gonadal segments bearing a sperm transfer system in copulatory area present	**109**
109(108)	Anterior end sucker clearly separated from rest of body (Fig. 95A); lateral branchiae digitiform (Fig. 95B); marine members widespread; freshwater taxa mostly limited to the Palearctic and Nearctic	**Piscicolidae**
–	Anterior end sucker not clearly separated from rest of body; lateral branchiae branching (Fig. 85A); marine members widespread; freshwater taxa absent from Palearctic and Nearctic	**Ozobranchidae**
110(1)	Clitellum situated in region of male pore	**111**
–	Clitellum situated posterior to male pore	**125**
–	Clitellum situated anterior to male pore	**128**
111(110)	Male pores in segment following testicular segment (plesioporous)	**112**
–	Male pores two or more segments following testicular segment (opisthoporous)	**113**
112(111)	Dorsal pores on mid-dorsal line absent; chaetae first appear on 2^nd^ segment after peristomium (= S3 for oligochaete workers) (Fig. 106A); genital chaetae present (Fig. 106E); sperm sac absent; native to Nearctic and Neotropics	**Rhinodrilidae**
–	Dorsal pores on mid-dorsal line present; chaetae first appear on 1^st^ segment after peristomium (= S2 for oligochaete workers); genital chaetae absent; sperm sac present (Fig. 68A); widespread but most diverse in Asia	**Moniligastridae**
113(111)	Clitellum partially encircles body	**114**
–	Clitellum fully encircles body	**121**
114(113)	Calciferous glands absent; intestinal typhlosole absent; natively distributed in Northern Hemisphere but also found in Neotropics (Sparganophilidae) or only known from southern Africa (Tritogeniidae)	**Sparganophilidae, Tritogeniidae**
–	Calciferous glands present; intestinal typhlosole present	**115**
115(114)	Prostate gland absent	116
–	Prostate gland present	**120**
116(115)	Gonadal segments not bearing genital papillae	**117**
–	Gonadal segments bearing genital papillae present	**118**
117(116)	One pair nephridia in each segment (holonephridia); native to Neotropics	**Glossoscolecidae**
–	Multiple, minute, nephridia in each segment (meronephridia); native to southern Africa	**Tritogeniidae**
118(116)	Spermathecae post-testicular	**119**
–	Spermathecae pre-testicular (Fig. 41A); native to Neotropics	**Glossoscolecidae**
–	Spermathecae in testicular segments (Fig. 66A); native to southern Africa	**Microchaetidae**
119(118)	Sperm sac absent; spermathecal pores, two pairs (Fig. 147A); native to Neotropics	**Tumakidae**
–	Sperm sac present; spermathecal pores, four or five pairs (Fig. 66A); native to southern Africa	**Microchaetidae**
120(115)	Intestinal typhlosole formed from all layers of the intestine; clitellum situated in region of female pore (Fig. 50A); native to Palaearctic	**Hormogastridae**
–	Intestinal typhlosole formed from the inner (epithelial) layer only of the intestine; clitellum situated posterior to female pore (Fig. 41A); native to Neotropics	**Glossoscolecidae**
121(113)	Dorsal pores on mid-dorsal line absent; clitellum situated posterior to female pore; native to southern Japan (only known from Lake Biwa, S Japan)	**Biwadrilidae**
–	Dorsal pores on mid-dorsal line present; clitellum situated in region of female pore	**122**
122(121)	Clitellum short or long; one or two pairs of testes in total	**123**
–	Clitellum long (~ 50 segments); nine or ten pairs of testes (Fig. 61A); native to Nearctic (Louisiana, USA only)	**Lutodrilidae**
123(122)	Clitellum long (~ 30 segments) (Fig. 25A); tubercula pubertatis absent; gizzard and calciferous glands absent; native to Palaearctic	**Criodrilidae**
–	Clitellum short (15–17 segments); tubercula pubertatis present; gizzard and calciferous glands usually present	**124**
124(123)	Tubercula pubertatis as paired ridges ventral to the clitellum (Fig. 50B); spermathecal pores located near male and female (gonadal) pores; prostate glands, more than one pair (Fig. 50A); native to Palaearctic	**Hormogastridae**
–	Tubercula pubertatis as paired ridges on the ventrolateral margins of the clitellum (Fig. 78B); spermathecal pores located anterior to male and female (gonadal) pores; prostate gland, one pair (Fig. 78A); widespread	**Ocnerodrilidae**
125(110)	Spermathecae post-testicular	**126**
–	Spermathecae pre-testicular	**127**
–	Spermathecae in testicular segments (Fig. 58A); native to Holarctic but many cosmopolitan species	**Lumbricidae**
126(125)	Tubercula pubertatis absent; prostate gland present (Fig. 54A); last several segments not flattened; only known from Madagascar	**Kynotidae**
–	Tubercula pubertatis present; prostate gland absent (Fig. 8A); dorsal side of last several segments flattened for caudal respiration; circumtropical	**Almidae**
127(125)	Calciferous glands absent; intestinal typhlosole absent; genital chaetae absent; native to Northern Hemisphere but also found in Neotropics	**Sparganophilidae**
–	Calciferous glands present; intestinal typhlosole present; genital chaetae present; native to Northern Hemisphere but many cosmopolitan species	**Lumbricidae**
128(110)	Spermathecae unpaired, post-testicular (Fig. 33A); native to Afrotropics	**Eudrilidae**
–	Spermathecae paired, pre-testicular	**129**
129(128)	Male pore and prostate pore on segment XVIII united, discharge through single pore (Fig. 64A); all continents except Antarctica	**Megascolecidae**
–	Male pore and prostate pores (XVII, XVIII, respectively) not united (Fig. 3A); widespread, especially in Southern Hemisphere	**Acanthodrilidae**
130(1)	Brightly colored epidermal glands present; prostomium broadly rounded anteriorly; clitellum absent	**131**
–	Brightly colored epidermal glands absent; prostomium bluntly conical, clitellum present (in mature specimens) [= true microdriles]	**132**
131(130)	Prostomium wider than rest of body (Fig. 6B, C); chaetae more than two per bundle, hair-like and crotchets (chaetae rarely absent) (Fig. 6D, J); aquatic or terrestrial	**Aeolosomatidae**
–	Prostomium not wider than rest of body (Fig. 51A, B); chaetae two per bundle (usually), bristled and shovel-shaped (Fig. 51C); terrestrial	**Hrabeiellidae**
132(130)	Hair chaetae absent; usually only short simple-pointed crotchets present (rarely crotchets bifid or all chaetae absent) (Fig. 31C–H); spermathecal pores on segment 5 (Fig. 31A); terrestrial, freshwater, or marine	**Enchytraeidae**
–	Hair chaetae present or absent; crotchets present or absent; spermathecal pores otherwise	**133**
133(132)	Spermathecal pores located within one or two segments of male pores	**134**
–	Spermathecal pores located well anterior to male pores	**143**
134(133)	Eyes absent; spermathecae post-testicular	**135**
–	Eyes present or absent; spermathecae pre-testicular	**140**
–	Eyes present or absent; spermathecae in testicular segments	**142**
135(134)	Hair chaetae present or absent; crotchets present; ovaries, one pair	**136**
–	Hair chaetae absent; crotchets present; ovaries, two pairs; Nearctic and Palaearctic, mainly freshwater	**Lumbriculidae**
–	All chaetae absent; ovaries, unpaired; Neotropics, freshwater	**Narapidae**
136(135)	Dorsal bundle chaetae first appear on 1^st^ segment after peristomium (= S2 for oligochaete workers); internal support chaetae absent	**137**
–	Dorsal bundle chaetae first appear on 2^nd^ segment after peristomium (= S3 for oligochaete workers); internal support chaetae absent	**139**
–	Dorsal bundle chaetae first appear on 3^rd^ segment after peristomium (= S4 for oligochaete workers); support chaetae present (alongside hair chaetae); mostly freshwater Gondwanan	**Phreodrilidae**
137(136)	Pygidial (caudal) appendages absent; branchiae absent; hair chaetae absent	**138**
–	Pygidial (caudal) appendages present (three, ventrolateral ones longer than dorsomedial one); branchiae present; hair chaetae present	**Naididae, Opistocystinae**
138(137)	Both nephridial pores and gonoducts located around clitellum; male pores in segment following testicular segment (plesioporous); Palaearctic, freshwater	**Dorydrilidae**
–	Nephridial pores located posterior to gonoducts; male pore in same segment as corresponding testes (prosoporous); Nearctic and Palaearctic, mainly freshwater	**Lumbriculidae**
139(136)	1^st^ segment after peristomium (= S2) chaetous; ventral bundle chaetae first appear on 1^st^ segment; hair chaetae in dorsal (notopodial) position; nephridial pores located anteriorly, gonoducts located around clitellum; mostly freshwater Gondwanan	**Phreodrilidae**
–	1^st^ segment after peristomium (= S2) achaetous; ventral bundle chaetae first appear on 2^nd^ segment; hair chaetae in both dorsal and ventral positions (maybe very long); both nephridial pores and gonoducts located around clitellum; Nearctic and Palaearctic, freshwater	**Parvidrilidae**
140(134)	Prostomium anteriorly with a tentacle-like extension (‘proboscis’); both nephridial pores and gonoducts located around clitellum; macrobiotic size; Neotropics, freshwater	**Tiguassidae**
–	Prostomium rarely with an anterior tentacle-like extension (‘proboscis’); nephridial pores located posterior to gonoducts; meiobiotic size	**141**
141(140)	Eyes on head absent; male pore in same segment as corresponding testes (prosoporous); prostate gland absent; Palaearctic, mainly freshwater	**Lumbriculidae**
–	Eyes on head present or absent; male pore in segment following testicular segment (plesioporous); prostate gland present; cosmopolitan, mainly freshwater	**Naididae, Naidinae**
142(134)	Prostomium posteriorly demarcated from peristomium without a tongue (prolobic); eyes on head absent; nephridial pores located anteriorly, gonoducts located around clitellum	**Naididae, Tubificinae**
–	Prostomium not posteriorly demarcated from peristomium (zygolobic); eyes on head absent; both nephridial pores and gonoducts located around clitellum	**Naididae,Pristininae**
–	Prostomium not posteriorly demarcated from peristomium (zygolobic); eyes on head present (usually); nephridial pores located posterior to gonoducts	**Naididae, Naidinae**
143(133)	Male pore in same segment as corresponding testes (prosoporous); Nearctic and Oceanic, marine	**Randiellidae**
–	Male pore in segment following testicular segment (plesioporous)	**144**
–	Male pore two or more segments following testicular segment (opisthoporous)	**147**
144(143)	Chaetal bundles arranged in closely spaced lateral and ventrolateral pairs	**145**
–	Chaetal bundles arranged in widely spaced lateral and ventrolateral pairs	**146**
145(144)	Hair chaetae absent; crotchets from S2; clitellum situated posterior to male pore(s); Nearctic and Oceanic, marine	**Randiellidae**
–	Hair chaetae present from S3; clitellum situated in region of male pore(s); Neotropics, Australasia and Antarctica, terrestrial, freshwater, or marine	**Capilloventridae**
146(144)	Small worms; chaetae more than two per bundle; testes, one pair in total; female pores, one pair; Palaearctic, freshwater	**Propappidae**
–	Very elongate worms; chaetae one or two per bundle; testes, two pairs in total (rarely one pair); female pores, two pairs; cosmopolitan, typically aquatic or limnic	**Haplotaxidae s.s. ^1^**
147(143)	Tubercula pubertatis present; testes, two pairs in total; prostate glands, three pairs; Afrotropical, terrestrial	**Syngenodrilidae**
–	Tubercula pubertatis absent; testes, one pair in total; prostate glands, one pair; circumtropical (probably), freshwater and swampy ground	**Alluroididae**

### ﻿﻿Taxonomic accounts

#### ﻿Aberrantidae Wolf, 1987 [polychaete]

Fig. 1

**Common name.** None.

**LSID.** urn:lsid:marinespecies.org:taxname:233984.

**Figures 1, 2.**
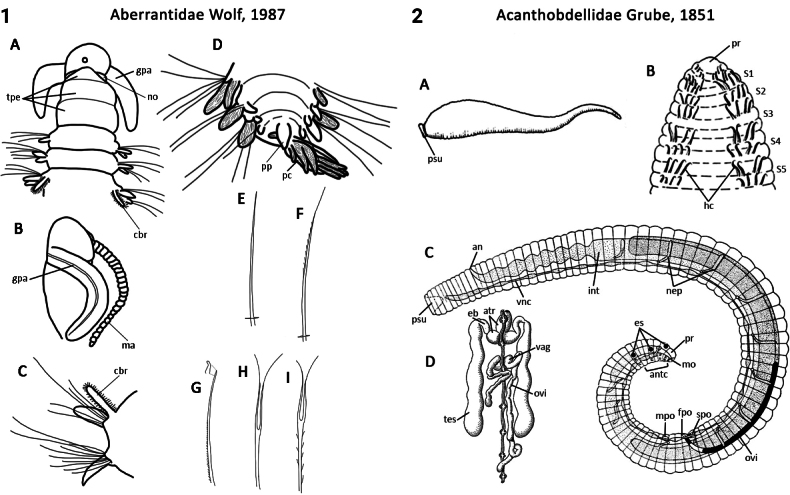
Distinguishing features: **1.**Aberrantidae: **A–E, G, H.***Aberrantaenignatica*; **F, I.***A.banyulensis*. **A.** Anterior region, dorsal, prostomial antenna missing; **B.** Anterior region, lateral; **C.** Parapodium from chaetiger 11; **D.** Posterior region, ventral (right pygidial cirrus missing); **E, F.** Capillary notochaetae; **G–I.** Lyriform and hooked neurochaetae. Abbreviations: cbr ciliated branchia; gpa grooved palp; ma median antenna; pc pygidial cirrus; pp pygidial papilla; tpe triannulate peristomium. Sources: **A–I** derivatives of Mackie (2019: fig. 7.2.1.2). **2.**Acanthobdellidae: **A–C.***Acanthobdella* sp.: **A.** General appearance; **B.** Anterior region; **C.** Diagram of anatomy, lateral view; **D.***Acanthobdellapeledina* reproductive system, female and male. Abbreviations: an anus antc anterior chaetigers atr atrium eb ejaculatory bulb es eye spot fpo female pore hc hook chaeta int intestine mo mouth mpo male pore nep nephridium ovi ovisac pr prostomium psu posterior sucker S segment spo spermathecal pore tes testis vag vagina vnc ventral nerve cord. Sources: **A–C** derivatives of fig. 12, Mann (1962), **D** derivative of fig. 4, Davies and Govedich (2001)

**Diagnosis (Level 3).** Peristomium a triple ring (or double ring with first segment achaetous) (Fig. 1A, tpe); notopodial and neuropodial lobes large, fusiform adorned with sensory hairs (Fig. 1C, D); branchiae present (Fig. 1C, cbr).

**Description.** See Suppl. material 1.

**Remarks.**Aberrantidae is represented by a single genus, *Aberranta* Hartman, 1956, and five species (WoRMS 2025) from Nearctic and Palearctic oceans and seas. Aberrantidae was revised by Mackie et al. (2005), and a key is provided by Parapar (2018).

**Environment and habitat.** Aquatic, marine, coastal, continental shelf, or deep sea; soft substrata.

#### ﻿Acanthobdellidae Grube, 1851 [leech]

Fig. 2

**Common name**. Hook-faced fish worm.

**LSID.** Urn:lsid:marinespecies.org:taxname:1779404.

**Diagnosis (Level 3).** Body segments fixed at 31 segments including two pre-oral ‘segments’ (prostomium and peristomium) and 29 post-oral segments; secondary annulation present; chaetae present (Fig. 2B); anterior sucker absent (Fig. 2A, C).

**Description.** See Suppl. material 1.

**Remarks.** We follow De Carle et al. (2023) in recognizing a single family of hook-faced fish worms (Acanthobdellidae, Acanthobdelliformes) comprising two genera, each with a single species. Prior to De Carle et al. (2023), a second monotypic family was recognised – Paracanthobdellidae for *Paracanthobdellalivanowi* (Epstein, 1966) but these authors found that morphological differences between the two acanthobdellidan species were not sufficient to warrant two families, although they continued to maintain the two monotypic genera, *Acanthobdella* Grube, 1851 and *Paracanthobdella* Epshtein, 1987. Acanthobdelliformes is the sister group of leeches (Hiriudinida) (Tessler et al. 2018); they differ from leeches most obviously in an underdeveloped anterior sucker, which is equipped with five rows of hooked chaetae. Acanthobdellidae are mostly ectoparasites of salmonid fishes, restricted to high boreal latitudes (Arctic and sub-Arctic).

**Environment and habitat.** Aquatic, freshwater; soft substrata or epizoic (ectoparasites, primarily of salmonid fishes).

#### ﻿Acanthodrilidae Claus, 1880 [megadrile; includes alternative representations ‘Benhamiidae’, ‘Exxidae’ and ‘Octochaetidae’]

Fig. 3

**Common name.** None.

**LSID.** Urn:lsid:marinespecies.org:taxname:994661.

**Figures 3, 4.**
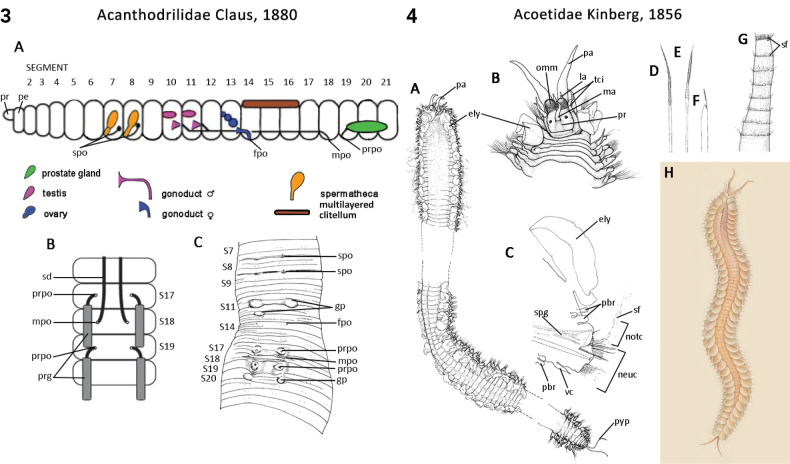
Distinguishing features: **3.**Acanthodrilidae: **A.** Schematic image of reproductive organs, dorsal side up; **B.** Arrangement of pores; **C.***Diplotremaaustralis*, genital field. Abbreviations: fpo female pore gp genital papilla mpo male pore pe peristomium prpo prostate pore pr prostomium prg prostate gland S segment sd sperm duct spo spermathecal pore. Sources: **A, B** derivatives of fig. 8.4 B, 8.8 A Jamieson (2006), **C** derivative of fig. 17 Dyne and Jamieson (2004). **4.**Acoetidae: **A.***Eupanthalis* sp., animal with details of anterior, middle and posterior segments, dorsal view. **B–F.***Polyodontesaustraliensis*: **B.** Anterior end, dorsal view; **C.** Parapodium of chaetiger 26; **D.** Superior neurochaetae from chaetiger 26; **E.** Spinose neurochaeta from chaetiger 27; **F.** Stout aristate neurochaeta from chaetiger 27; **G.** Anterior section of tube; **H.***Panthalisoerstedi* entire animal, dorsal view. Abbreviations: ely elytron la lateral antenna ma median antenna neuc neurochaetae notc notochaetae omm ommatophore pa palp pbr pinnate branchia pr prostomium pyp pygidial papilla sf silk fibre spg spinning gland vc ventral cirrus. Sources: **A–G** after fig. 1.68 Beesley et al. (2000), H after Pl. XXVIA, fig. 20, MacIntosh (1900–1922).

**Diagnosis (Level 2).** Secondary annulation present; clitellum fully encircles body, situated anterior to male pores (Fig. 3A, mpo), in region of female pores; 0–3 pairs spermathecal pores; calciferous glands absent.

**Description.** See Suppl. material 1.

**Remarks.**Acanthodrilidae as perceived here largely follows the concept of James and Davidson (2012), which is based on a molecular (28S, 18S, and 16S) phylogenetic study that found support for the broad concept of the family (= Acanthodrilinae sensu Jamieson 2000; Jamieson 2006), which includes Acanthodrilinae, Benhamiinae Michaelsen, 1897, Octochaetinae Michaelsen, 1900 and the monotypic Exxidae Blakemore, 2000. Although James and Davidson (2012) considered that the consistent support for the monophyly of Benhamiinae warranted its elevation to family rank, we have refrained from doing this because of the conflicting findings of a more recent phylogenomic study that showed the largest benhamiine genus *Dichogaster* Beddard, 1888 nested within Acanthodrilidae. As a result, the family concept adopted here is much broader than the one used in WoRMS (2025), which recognizes these subfamilies as families.

Acanthodrilidae s.l. is similar to Megascolecidae and Ocnerodrilidae and only distinguishable from these taxa at DELTA Diagnostic Level 2. Acanthodrilidae s.l. is well represented by endemics throughout former Gondwana and is absent from the Palaearctic; the presence of acanthodrilid species in the USA, Mexico, and the Caribbean Islands (Misirlioğlu et al. 2023) may represent introductions. Introduced species also occur in Gondwanan regions – Blakemore (1999) identified 12 non-endemic Acanthodrilidae species from Australia, demonstrating the group’s propensity for human-assisted spread. Acanthodrilidae is one of only two earthworm families having maritime members. Maritime acanthodriles include species of *Notiodrilus* Michaelsen, 1899 (referred to *Microscolex* Rosa, 1887 by some authors) and *Rhododrilus* Beddard, 1899. Jamieson et al. (2002) and Dyne and Jamieson (2004) provide useful treatments of the family and subfamilies. Plisko and Nxele (2015) provide a key to distinguish foreign Acanthodrilidae taxa from native ones of South Africa.

**Environment and habitat.** Terrestrial (rarely freshwater aquatic – Octochaetinae), moist terrestrial.

#### ﻿Acoetidae Kinberg, 1856 [polychaete]

Fig. 4

**Common name.** Bullet worm (*Eupolyodontes* Buchanan, 1894 species).

**LSID.** Urn:lsid:marinespecies.org:taxname:19199.

**Diagnosis (Level 3).** Prostomium pentagonal to quadrangular in shape; ommatophores present (Fig. 4B; omm); facial tubercle present.

**Description.** See Suppl. material 1.

**Remarks.**Acoetidae is represented by eight genera and 56 species (WoRMS 2025). Pettibone (1989) revised the family and provided a key to genera. Acoetidae appears to have a worldwide distribution in coastal areas and continental seas; deep sea and high latitude records are lacking (GBIF.org 2023). Gil (2011) and Parapar et al. 2015) provide revised keys to European taxa. Barnich (2011) provides a key to scale worms, including Acoetidae, of British and Irish waters. Salazar-Vallejo et al. (2014) provide a key to *Eupanthalis* McIntosh, 1876 of the world.

**Environment and habitat.** Aquatic, marine, coastal, continental shelf, or deep sea; soft substrata.

#### ﻿Acrocirridae Banse, 1969 [polychaete]

Fig. 5

**Common name.** None.

**LSID.** Urn:lsid:marinespecies.org:taxname:920.

**Figures 5, 6.**
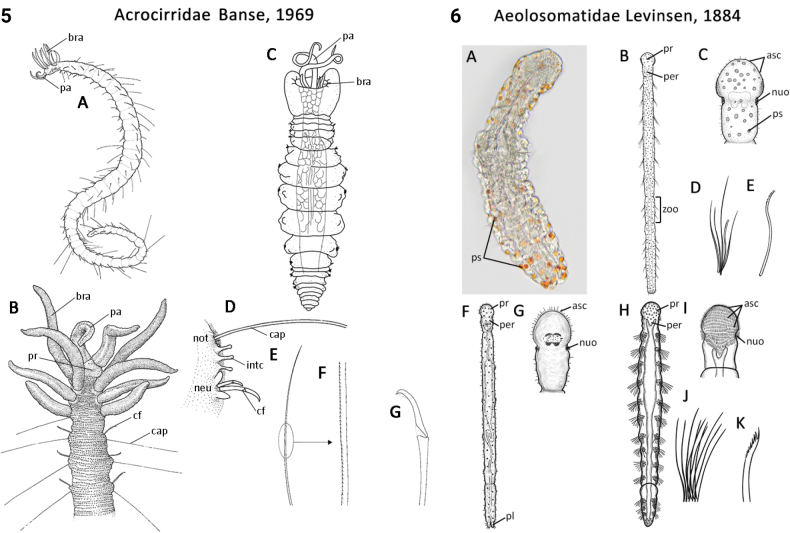
Distinguishing features: **5.**Acrocirridae: **A, B, D–G.***Macrochaetaaustraliensis*; **A.** Entire animal lateral view; **B.** Anterior end dorsal view; **C.***Chauvinelia* sp. dorsal view of entire animal, chaetae are not drawn; **D.** Parapodium of chaetiger 30; **E, F.** Capillary from parapodium of chaetiger 10; **G.** Compound falciger from parapodium of chaetiger 35. Abbreviations: bra branchia cap capillary chaetae cf compound falciger intc interramal cirrus neu neuropodium not notopodium pa palp pr prostomium. Sources: **A, B, D–G** derivatives of fig. 1.111 Beesley et al. (2000), **C** derivative of fig. 7.3.1.7.8 Martínez et al. (2019). **6.**Aeolosomatidae: **A.***Aeolosoma* sp., live animal, dorsal view; **B, C.**Aeolosomacf.hemprichi, entire animal with three zooids, dorsal view (**B**) anterior end, dorsal view (**C**); **D, E.***Aeolosomapsammophilum*, chaetal fascicle (**D**), sigmoid chaeta (**E**); **F, G.***Rheomorphaneizvestnovae* dorsal view (**F**), dorsal view of anterior end (**G**); **H–K.***Hystricosomachappuisi*, dorsal view (**H**), ventral view of anterior end (**I**), chaeta fascicle (**J**), sigmoid chaeta (**K**). Abbreviations: asc anterior sensory cilia nuo nuchal organ per peristomium pl pygidial lobe pr prostomium ps pigment spot zoo zoid. Sources: **A** after Malcolm Storey, Bioimages, The Virtual Field-Guide, UK https://www.bioimages.org.uk/, under cc by nc-sa-3.0; **B, C** after fig. 1.129 Beesley et al. (2000), **D–G** redrawn from Bunke (1967), **H–K** redrawn from Pop (1975).

**Diagnosis (Level 3).** Papillated epidermis (Fig. 5B); discrete head present, retractable into anterior segments (Fig. 5C); prostomium rounded to oval; or narrow, keel- or ridge-shaped (Fig. 5B); palps present (Fig. 5B, C); peristomium visible, first and second segment achaetous (Fig. 5A–C).

**Description.** See Suppl. material 1.

**Remarks.**Acrocirridae is a morphologically diverse family represented by ten genera and 45 species (WoRMS 2025), distributed worldwide in both benthic and holopelagic habitats. Key taxonomic publications on the family include Banse (1969), who described the family, originally based on only two genera removed from Cirratulidae, and Salazar-Vallejo et al. (2007), who include keys to known genera and species at the time. Osborn at al. (2009) erected the holopelagic genus, *Swima* Osborn, Haddock, Pleijel, Madin & Rouse, 2009 and analysed the phylogeny of the family in relation to other cirratuliforms. Gil (2011) provides a revised key to European genera and Salazar-Vallejo (2009) provides a key to Caribbean species. Magalhães and Bailey-Brock (2011) provide a key to world *Acrocirrus* Grube, 1873 species.

**Environment and habitat.** Aquatic, marine, coastal, or continental shelf or deep sea; soft or hard substrata, or holopelagic.

#### ﻿Aeolosomatidae Levinsen, 1884 [microdrile]

Fig. 6

**Common name.** Suction-feeding worms.

**LSID.** Urn:lsid:marinespecies.org:taxname:558773.

**Diagnosis (Level 3).** Body with fixed number segments (less than 14, when not budding asexually); dorsoventrally flattened (Fig. 6A); head ciliated ventrally and laterally (Fig. 6G, I, asc); nuchal organs present (Fig. 6C, G, I, nuo); male gonoducts absent.

**Description.** See Suppl. material 1.

**Remarks.**Aeolosomatidae is here considered to contain the monotypic Potamodrilidae Bunke, 1967 and belong to Oligochaeta following Rouse et al. (2022), which is how they have been considered at various times in the past, even though the resemblance is not strong, in particular, their lack of a clitellum. For the last 40 years or so, the two families were considered to be part of the order Aphanoneura Timm, 1981. Brinkhurst and Jamieson (1971) provide keys to genera and species at the time. The family contains four genera and 33 species (WoRMS 2025) which occur worldwide, although it is best known from the Palaearctic Realm, and there are few records from other realms, including polar regions (GBIF.org 2023).

**Environment and habitat.** Aquatic, brackish, or freshwater (meiopsammon of rivers; rarely coastal sediments); soft substrata or epizoic (rarely on crayfish or aquatic macrophytes).

#### ﻿Alluroididae Michaelsen, 1900 [microdrile]

Fig. 7

**Common name.** None.

**LSID.** Urn:lsid:marinespecies.org:taxname:1039992.

**Figures 7, 8.**
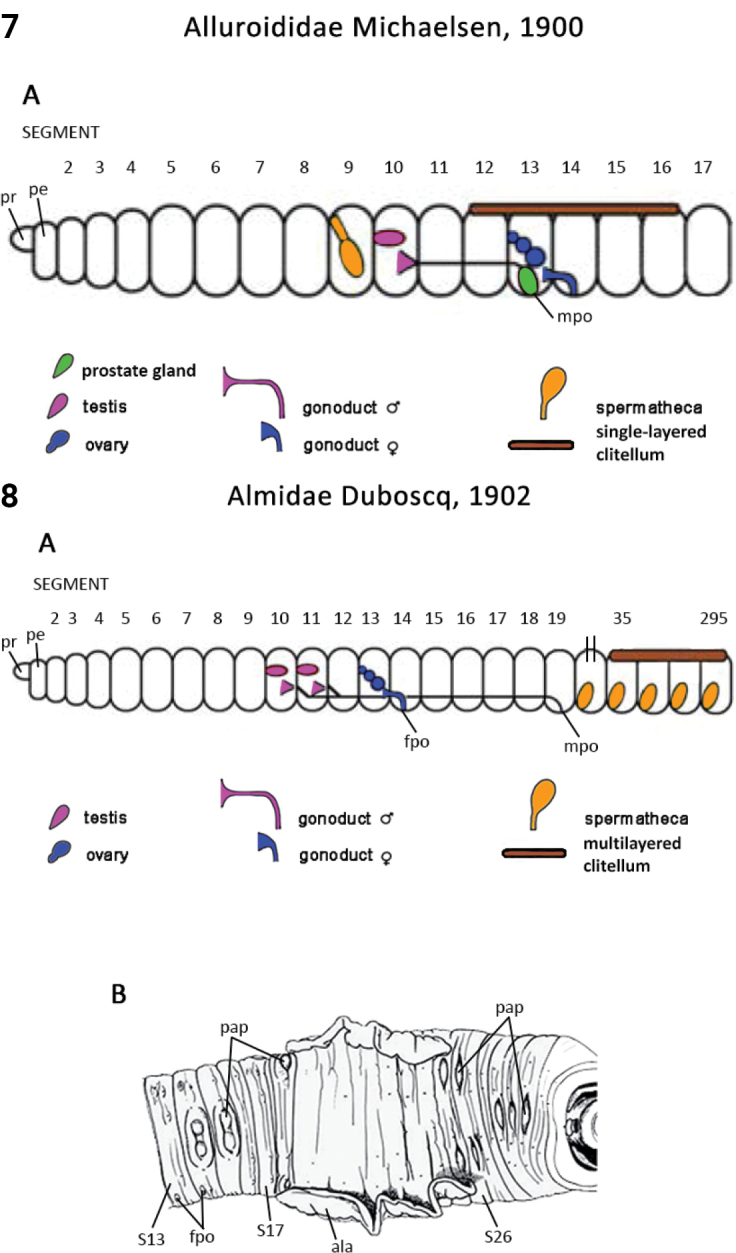
Distinguishing features: **7.**Alluroididae: **A.** Diagram of reproductive organs, dorsal side up. Abbreviations: mpo male pore pe peristomium pr prostomium. Sources: **A** derivative of fig. 8.4 A Jamieson (2006). **8.**Almidae: **A.** Schematic image of reproductive organs, dorsal side up; **B.***Glyphidriluskukenthali*, anterior end showing genital region, ventral view. Abbreviations: ala alae fpo female pore mpo male pore pap papilla pe peristomium pr prostomium S segment. Sources: **A, B** derivatives of fig. 8.4B, 8.38A Jamieson (2006).

**Diagnosis (Level 3).** Secondary annulation present; (Fig. 7A); tubercula pubertatis absent; dorsal pores on mid-dorsal line absent; nephridial pores and gonoducts located around clitellum; tubercula pubertatis absent (Fig. 7A); testes, one pair; male pores two or more segments following testicular segment (opisthoporous) (Fig. 7); spermathecal pores three pairs, located well anterior to male and female pores; prostate gland present.

**Description.** See Suppl. material 1.

**Remarks.**Alluroididae (along with Syngenodrilidae) are the sister group to Crassiclitellata (Jamieson 2006; Timm 2012), sharing several reproductive features in common, but having a single-celled clitellum like other microdriles. The family comprises eight genera and 13 species (Jamieson and Fragoso 2024) and are only found in inland waters of the Afrotropical and Neotropical realms, though this may reflect low sampling intensity of megadriles in swampy/semi-aquatic fresh waters worldwide. Jamieson and Fragoso (2024) provide a key to genera and descriptions of known species.

**Environment and habitat.** Aquatic (amphibious), freshwater (including swampy ground).

#### ﻿Almidae Duboscq, 1902 [megadrile]

Fig. 8

**Common name.** None.

**LSID.** Urn:lsid:marinespecies.org:taxname:1039993.

**Diagnosis (Level 1).** Clitellum situated posterior to male and female pores; spermathecae present; post-testicular (Fig. 8A); tubercula pubertatis present (Fig. 8B).

**Description.** See Suppl. material 1.

**Remarks.**Almidae is a widely distributed family of megadrile earthworms occurring in the tropics and subtropics, excluding Australia. It includes 64 species in seven genera (Misirlioğlu et al. 2023, and references therein). At Diagnostic Level 2 the family was indistinguishable from Lumbricidae. They are one of the few megadrile families that include species that are both aquatic and semi-aquatic (Martin et al. 2008). Chanabun et al. (2013, 2023) provide a key and accounts of worldwide *Glyphidrilus* Horst, 1899 species.

**Environment and habitat.** Terrestrial or aquatic, freshwater.

#### ﻿Alvinellidae Desbruyères & Laubier, 1986 [polychaete]

Fig. 9

**Common name.** Pompeii worms; palm worms.

**LSID.** Urn:lsid:marinespecies.org:taxname:233985.

**Figures 9, 10.**
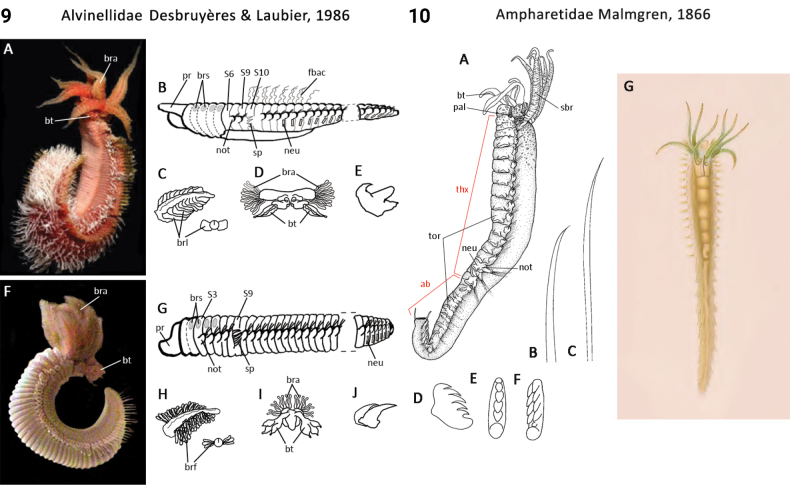
Distinguishing features: **9.**Alvinellidae: **A.***Alvinellapompejana* entire animal; **B–E.***A.pompejana*; **B.** Body shape and position of the uncinigerous tori; **C.** Gill shape; **D.** Buccal apparatus with tentacles; **E.** Shape of uncini; **F.***Paralvinellasulfincola*, entire animal; **G–J.***P.sulfincola*; **G.** Body shape and position of the uncinigerous tori; **H.** Gill shape; **I.** Buccal apparatus with tentacles; **J.** Shape of uncini. Abbreviations: bra branchia brl branchial lamella brs branchial scar bt buccal tentacles fbac filamentous bacteria neu neuropodium not notopodium pr prostomium S segment sp spine. Sources: **A–J** derivatives of fig. 4, fig. 10 Jollivet and Hourdez (2021). **10.**Ampharetidae: **A–E.***Amphicteisdalmatica*; **A.** Entire animal lateral view; **B, C.** Chaetae: **B.** Palea; **C.** Capillary notochaeta; **D, E.** Neurochaetal uncinus from chaetiger 15 lateral (**D**) and frontal (**E**) views; **F.***Auchenoplax* sp., neurochaetal uncinus almost frontal view; **G.***Amphareteacutifrons*, entire animal. Abbreviations: ab abdomen bt buccal tentacles neu neuropodium not notopodium pal paleae sbr smooth branchia thx thorax tor torus. Sources: **A–F** derivatives of fig. 1.112 Beesley et al. (2000), **K** derivative of MacIntosh (1900–1922).

**Diagnosis (Level 3).** Body regionalization absent (Fig. 9A, B, F, G); prostomium hood-like and covering buccal tentacles dorsally (Fig. 9B, F, G); first and second segments achaetous (Fig. 9B, G) thoracic ventral glandular areas present, indistinct mid-ventral swelling.

**Remarks.**Alvinellidae comprises two genera and 12 species (WoRMS 2025). Once thought to be confined to the Pacific, they now appear to be distributed in deep waters of all majoroceans, except perhaps the Atlantic; they are associated with deep-sea hydrothermal vents. The group is apparently absent from high latitudes.

**Environment and habitat.** Aquatic, marine, continental shelf, or deep sea; hydrothermal vents (not presently known from cold seeps).

#### ﻿Americobdellidae Caballero, 1956 [leech]

**Common name.** None.

**LSID.** Urn:lsid:marinespecies.org/aphia.php?p = taxdetails&id = 1602782.

**Diagnosis (Level 3).** Body regionalization absent, pigmentation present; eyes on head absent; ridges on pharynx rotated 60° to the right (strepsilaematous); gonadal segments bearing a copulatory area for sperm transfer; penis present.

**Description.** See Suppl. material 1.

**Remarks.**Americobdellidae is a monotypic family represented by *Americobdellavaldiviana* (Philippi, 1872). It occurs only in southern Chile. Ringuelet (1985) summarizes previous studies finding that the leech occurs in both terrestrial and aquatic habitats (below); this information, in combination with key character differences, e.g., presence or absence of eyes, between descriptions suggests that more than one species exists under this name. Once thought to be a member of Hirudiniformes based on overall morphology and feeding biology, it is now considered more closely allied with Erpobdellidae and Salifidae (Arhynchobdella), with which it shares the lack of eyes and poorly developed jaws (Siddall and Borda 2004). Its reproductive system is distinctive, resembling that of Piscicolidae and Ozobranchidae (Siddall et al. 2006).

**Environment and habitat.** Terrestrial or aquatic, in soil and freshwater; soft substrata.

#### ﻿Ampharetidae Malmgren, 1866 [polychaete]

Fig. 10

**Common name.** None.

**LSID.** Urn:lsid:marinespecies.org:taxname:981.

**Diagnosis (Level 2).** Body shape widest anteriorly and tapering posteriorly (Fig. 10A, G); prostomium hood-like, covering the tentacles dorsally (Fig. 10a); chaetae first appear on second or third segment after peristomium; thoracic lobe-like dorsolateral expansion absent (Fig. 10A).

**Description.** See Suppl. material 1.

**Remarks.**Ampharetidae is a large family with a worldwide distribution and represented by an estimated 64 genera and 300 species (Ebbe and Purschke 2021), although only 55 genera and 279 species are listed in WoRMS (2025). Key taxonomic publications on the family include Holthe (1986) and Reuscher et al. (2009) which include keys to known genera at the time. Gil (2011) provides a revised key to subfamilies and genera of Europe (at the time Melinninae (now Melinnidae) was a subfamily of Ampharetidae); Jirkov (2013) provides a key to boreal ampharetids; Jirkov and Leontovich (2013) provide a key to Terebellomorpha, including Ampharetidae, from the eastern Atlantic and the North Polar seas; and Alvestad and Budaeva (2020) provide interactive keys to species of Norwegian seas.

**Environment and habitat.** Aquatic, marine (very rarely fresh water), coastal, continental shelf, or deep sea; soft substrata or hydrothermal vents (not presently known from cold seeps).

#### ﻿Amphinomidae Lamarck, 1818 [polychaete]

Fig. 11

**Common name.** Fireworm, golden bristle worm.

**LSID.** Urn:lsid:marinespecies.org:taxname:960.

**Figures 11, 12.**
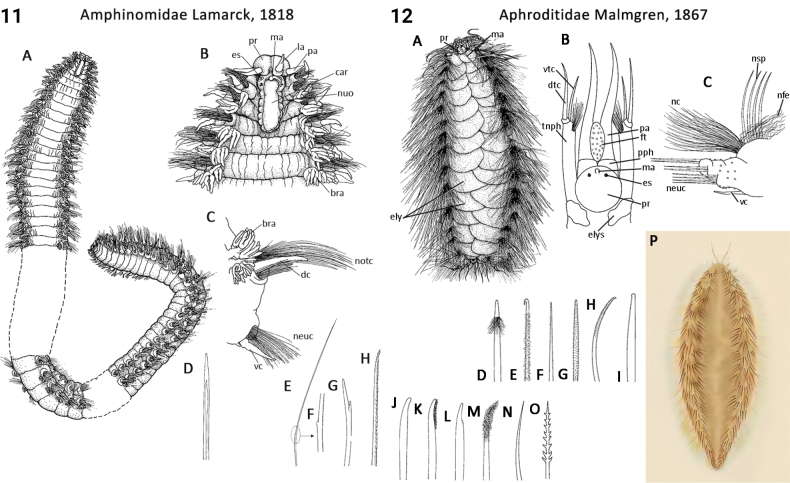
Distinguishing features: **11.**Amphinomidae: **A–H.***Eurythoe* sp.; **A.** Entire animal; **B.** Anterior end dorsal view; **C.** Parapodium of chaetiger 13; **D–F.** Chaetae from parapodium of chaetiger 13: **D.** Notoacicular spine; **E.** Fine ‘spurred’ notochaeta; **F.** Detail of spur as indicated in **E**. **G, H.** Chaetae from parapodium of chaetiger 38: **G.** Furcate neurochaeta; **H.** ‘Harpoon’ notochaeta. Abbreviations: bra branchia car caruncle dc dorsal cirrus es eye spot la lateral antenna ma median antenna neuc neurochaetae notc notochaetae nuo nuchal organ pa palp pr prostomium vc ventral cirrus. Sources: **A–H** derivatives of fig. 1.66 Beesley et al. (2000). **12.**Aphroditidae: **A.***Laetmonicemoluccana* dorsal view; **B–O.** Generalised aphroditid: **B.** Prostomium, first segment dorsal view; **C.** Parapodium posterior view; **D–I.** Notochaetal types; **J–O.** Neurochaetal types; **P.***Aphroditaaculeata*, dorsal view. Abbreviations: dtc dorsal tentacular cirri ely elytron elys elytral scar es eye spot ft facial tubercle ma median antenna nc notopodial capillary neuc neurochaetae nfe notopodial felt nsp notopodial spine pa palp pph palophore pr prostomium tnph tentaculophore vc ventral cirrus vtc ventral tentacular cirri. Sources: **A–O** derivatives of fig. 1.70 Beesley et al. (2000), **P** derivative of MacIntosh (1900–1922).

**Diagnosis (Level 3).** Prostomium triangular to trapezoidal (narrow end posteriorly), caruncle present (Fig. 11A, B); palps anteroventral (Fig. 11B); dorsal cirri present (Fig. 11C); notopodial lobes represented by at least one chaetal lobe (Fig. 11C); chaetae calcareous, brittle; lateral branchiae a single tuft per parapodium (Fig. 11C).

**Description.** See Suppl. material 1.

**Remarks.**Amphinomidae is represented by 21 genera and 158 species (WoRMS 2025; excluding fossil taxa), and have a worldwide distribution, although they are most diverse in warm coastal marine waters. Gil (2011) provides a key to European genera, Arias et al. (2013) provide a key to Mediterranean genera, and Sun and Li (2017) provide a key to world genera.

**Environment and habitat.** Aquatic, marine, coastal, continental shelf, or deep sea; soft or hard substrata.

#### ﻿Aphroditidae Malmgren, 1867 [polychaete]

Fig. 12

**Common name.** Sea mouse.

**LSID.** Urn:lsid:marinespecies.org:taxname:938.

**Diagnosis (Level 3).** Papillated epidermis; prostomium rounded to oval with median antenna only (Fig. 12A, B); ommatophores present; facial tubercle present (Fig. 12B); silky (feltage) chaetae) arising from notopodia of midbody (Fig. 12C, nfe).

**Description.** See Suppl. material 1.

**Remarks.**Aphroditidae is represented by seven genera and 110 species (WoRMS 2025, excluding fossil taxa), and are distributed worldwide. Pettibone (1966) provides a key to genera at the time. Gil (2011) and Parapar et al. (2015) provide a revised key to European taxa, while Hutchings and McRae (1993) provide keys to all genera (known at the time) and Australian species. Barnich and Fiege (2000) provide keys to *Aphrodita* Linnaeus, 1758 and *Aphroditella* Roule, 1898 of the NE Atlantic and Mediterranean. Barnich (2011) provides a key to scale worms, including Aphroditidae, of British and Irish waters.

**Environment and habitat.** Aquatic, marine, coastal, continental shelf, or deep sea; soft or hard substrata.

#### ﻿Apistobranchidae Mesnil & Caullery, 1898 [polychaete]

Fig. 13

**Common name.** None.

**LSID.** Urn:lsid:marinespecies.org:taxname:912.

**Figures 13, 14.**
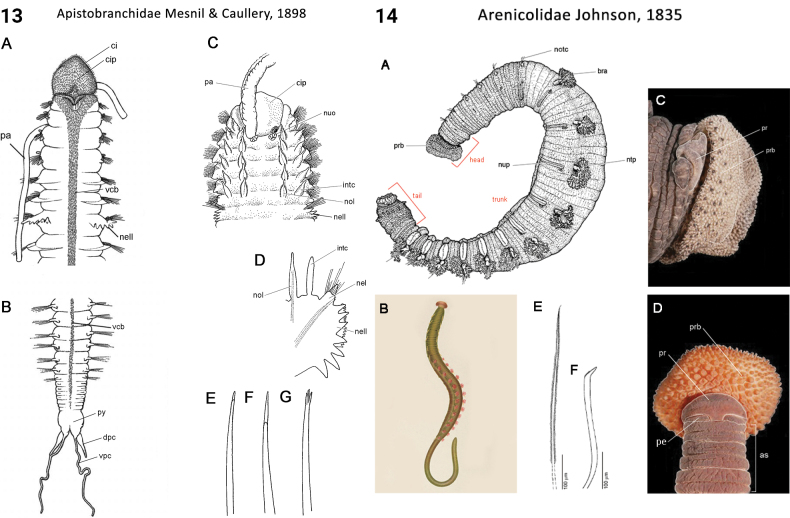
Distinguishing features: **13.**Apisthobranchidae: **A–G.**Apistobranchus, various species; **A, B.** Head and tail ends, ventral view; **C.** Head, dorsal view; **D.** Chaetiger 7; **E–G.** Neurochaetae showing differently worn tips. Abbreviations: ci cilia cip ciliated prostomium dpc dorsal pygidial cirrus intc interramal cirrus nel neuropodial lobe nell neuropodial lamella nol notopodial lobe nuo nuchal organ pa palp pr prostomium py pygidium vpc ventral pygidial cirrus vcb ventral ciliary band. Sources: **A, B** after fig. 5.1.1 Blake (1996a), **C–G** after fig. 1.103 Beesley et al. (2000). **14.**Arenicolidae: **A, E, F.***Arenicolabombayensis*; **B.***Arenicolamarina*; **C.***Abarenicolawellsi*; **D.***Arenicoloidesbranchialis*; **A.** Whole body, lateral view, showing three distinct parts, achaetous head, chaetigerous trunk and achaetous tail (tail may be chaetigerous in some species); **B.** Whole body; **C, D.** Two types of prostomia: **C.** Trilobate; **D.** Transverse; **E.** Pilose notopodial capillary; **F.** Neuropodial hook. Abbreviations: as achaetous segment, bra branchia, notc notochaetae, ntp notopodium, nup neuropodium, pe peristomium, pr prostomium, prb proboscis (everted). Sources: **A, E, F** derivatives of fig. 1.48, Beesley et al. (2000), **B** pl. LXXXVIII, fig. 5, McIntosh (1915), **C, D** derivatives of Darbyshire (2020).

**Diagnosis (Level 3).** Notopodial lobes slender, flask- or spindle-shaped (Fig. 13C, D); interramal fleshy process present (Fig. 13D; intc); ventral cirri present; tube-dwelling.

**Description.** See Suppl. material 1.

**Remarks.**Apistobranchidae is represented by seven species in a single genus, *Apistobranchus* Levinsen, 1884 (WoRMS 2025). For much of the last century Apistobranchidae has been allied with spioniform families, but molecular evidence has now revealed a closer association with chaetopteriforms and also revealed a potential synapomorphy (presence of aciculae) (Blake and Petti 2019; Rouse et al. 2022). Blake (1996a) provides a key to species at the time. The family appears to have a worldwide distribution but may be restricted to coastal areas and the continental shelf, as few records occur in the deep sea (GBIF.org 2023).

**Environment and habitat.** Aquatic, marine, coastal, or continental shelf; soft substrata.

#### ﻿Arenicolidae Johnston, 1835 [polychaete]

Fig. 14

**Common name.** Lugworms (larger forms).

**LSID.** Urn:lsid:marinespecies.org:taxname:922.

**Diagnosis (Level 3).** Body shape widest anteriorly and tapering posteriorly to an achaetous caudal region (tail; Fig. 14B); secondary annulation present, epidermis thick and rugose (Fig. 14A, D); prostomium bluntly conical (Fig. 14C); chaetae first appear on the second segment after peristomium (i.e., first segment achaetous, Fig. 14D, as).

**Description.** See Suppl. material 1.

**Remarks.**Arenicolidae currently contains four genera and 23 species (WoRMS 2025; excluding fossil taxa); they are distributed worldwide, although Antarctic and oceanic records appear to be rare (GBIF.org 2023). Wells (1959) redefined the genera and provided a generic key, and Darbyshire (2020) reviewed the family. Gil (2011) provides a revised key to European genera.

**Environment and habitat.** Aquatic, marine, coastal; soft substrata (and gravel), or rarely, algal mats (*Branchiomaldane* Langerhans, 1881).

#### ﻿Biwadrilidae Jamieson, 1971 [megadrile]

Fig. 15

**Common name.** None.

**LSID.** Urn:lsid:marinespecies.org:taxname:1039994.

**Figures 15, 16.**
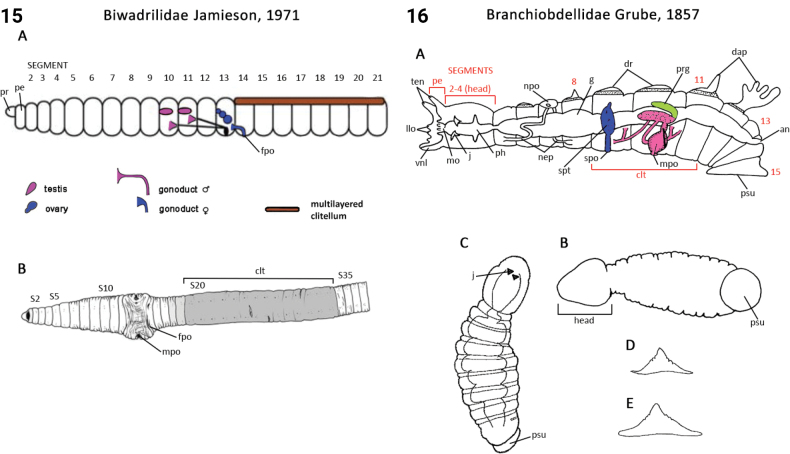
Distinguishing features: **15.**Biwadrilidae: **A.** Diagram of reproductive organs, dorsal side up. **B.***Biwadrilusbathybates*, ventral view. Abbreviations: clt clitellum, fpo female pore, mpo male pore, pe peristomium, pr prostomium, S segment. Sources: **A** derivative of fig. 8.4, Jamieson (2006), Non-leech Clitellata, **B** derivative of fig. 1, Blakemore (2008). **16.**Branchiobdellidae: **A.** Diagram of lateral aspect of a hypothetical branchiobdellid showing anatomic characters; **B.** Ventral view of *Triannulatamagna*; **C–E.***Branchiobdellaparasita*: **C.** Dorsal view; **D.** Upper jaw; **E.** Lower jaw. Abbreviations: an anus clt clitellum dap dorsal appendage dr dorsal ridge g gut j jaw llo lateral lobe mo mouth mpo male pore nep nephridium npo nephridial pore ph pharynx prg prostate gland psu posterior sucker spo spermathecal pore spt spermatheca ten tentacle vnl ventral lip. Sources: **A–C** derivatives of fig. 49, 55, 56 Brinkhurst and Gelder (2001), **D, E** derivatives of Timm (2009).

**Diagnosis (Level 3).** Dorsal pores on mid-dorsal line absent; clitellum present, fully encircles body at maturity, situated posterior to female pore (Fig. 15A); tubercula pubertatis present; gizzard absent; spermathecae absent; prostate gland lobular.

**Description.** See Suppl. material 1.

**Remarks.**Biwadrilidae is a monotypic family represented by *Biwadrilusbathybates* (Stephenson, 1917), known only from the Lake Biwa region in Japan (Misirlioğlu et al. 2023). Blakemore (2008) examined new material of *B.bathybates* from Lake Biwa and concluded that Biwadrilidae should be subsumed within Criodrilidae Vejdovsky, 1884, although the synonymy appears not to have been widely adopted. On the other hand, molecular analysis shows this family as sister to the Madagascan endemic family Kynotidae and, therefore, quite distant phylogenetically from Criodrilidae (James and Davidson 2012). Our dataset shows Biwadrilidae to be morphologically distinct from both families.

**Environment and habitat.** Terrestrial or aquatic, freshwater.

#### ﻿Branchiobdellidae Grube, 1850 [leech]

Fig. 16

**Common name.** Crayfish worms.

**LSID.** Urn:lsid:marinespecies.org:taxname:1060956.

**Diagnosis (Level 3).** Body sausage-shaped, grub-shaped, or pyriform; 15 body segments; testes, two pairs in total (Fig. 16A).

**Description.** See Suppl. material 1.

**Remarks.** Crayfish commensal worms, Branchiobdellidae, have been classified at various taxonomic levels from family to class, although for a long time, they been considered a family of oligochaetes (Brinkhurst and Gelder 2001; Govedich et al. 2009). They lack the anterior sucker of typical leeches, and instead have adhesive mouth parts for attaching to their host, and one pair of dorsal-ventral jaws instead of the typical triangular-arranged leech-type jaws. We follow the more recent phylogenomic evidence presented in Erséus et al. (2020) who regarded them as a leech. Crayfish worms comprise 22 genera and 156 species (WoRMS 2025) and occur only in the Nearctic and Palaearctic (Europe and East Asia); two species have been introduced to the UK (James et al. 2015). Holt and Opell (1993) provide an illustrated key to North American forms (formerly Cambarincolidae) and Gelder (1996) provides a checklist of species.

**Environment and habitat.** Aquatic, freshwater; soft substrata (rarely) or epizoic.

#### ﻿Capilloventridae Harman & Loden, 1984 [microdrile]

Fig. 17

**Common name.** None.

**LSID.** Urn:lsid:marinespecies.org:taxname:370516.

**Figures 17, 18.**
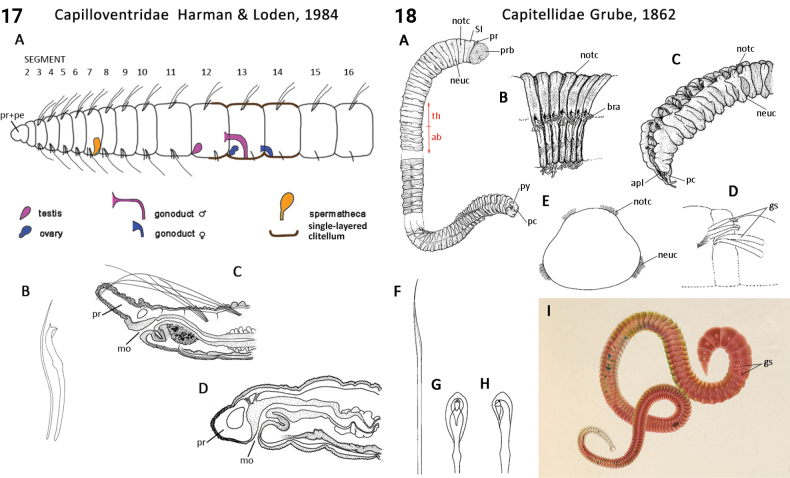
Distinguishing features: **17.**Capilloventridae: **A.** Diagram showing location of reproductive organs, dorsal side up; **B.***Capilloventeraustralis*, hair and crotchet chaeta, **C.** Anterior of *C.longicapitus* with long prostomium; **D.** Anterior of *C.acheronensis* with rounded prostomium. Abbreviations: mo mouth pe peristomium pr prostomium. Sources: **A** derivative of fig. 12.3 Thorp et al. (2019), **C, D** derivatives of figs 6, 45, 46 Pinder (2013). **18.**Capitellidae: **A.***Notomastustorquatus* parts of the entire animal; **B.***Dasybranchus* species posterior segments bearing dorsal branchiae; **C.***Scyphoproctustowraensis* posterior end with anal plate; **D.***Capitella* ‘*capitata*’ thoracic segments (7 to 9) showing genital spines lateral view; **E, F.***Notomastustorquatus*: **E.** Transverse section of body at chaetiger 10 showing arrangement of notopodia and neuropodia; **F.** Capillary notochaeta from thorax; **G, H.** Abdominal notochaetae of *Mediomastusaustraliensis*; **G.** Frontal view; **H.** Lateral view; **I.** Entire animal *Capitella* ‘*capitata*’. Abbreviations: apl anal plate gs genital spine bra branchia notc notochaetae pc pygidial cirrus pr prostomium prb proboscis py pygidium SI segment 1 Sources: **A–G** after fig. 1.49 Beesley et al. (2000), **I** after MacIntosh (1900–1922), pl. XCII, fig. 3.

**Diagnosis (Level 3).** Chaetae present, first appear on second or third segment after peristomium (Fig. 17A); hair chaetae present in both dorsal and ventral bundles (Fig. 17B); spermathecae pre-testicular, spermathecal pores well anterior to male and female (gonadal) pores (Fig. 17A).

**Remarks.**Capilloventridae was created for a marine species from Brazil (Harman and Loden 1984); a single genus, *Capilloventer* Harman & Loden, 1984 and six species are now known globally from both marine and freshwater environments of the Southern Hemisphere including southeastern Australia and Antarctica (Pinder and Brinkhurst 1997; Martin et al. 2008; Pinder 2013). Pinder (2013; in press) provides a key to the freshwater Australian species.

**Environment and habitat.** Aquatic; marine, brackish, or freshwater; coastal, continental shelf, littoral, or supralittoral.

#### ﻿Capitellidae Grube, 1862 [polychaete]

Fig. 18

**Common name.** Sludge worms.

**LSID.** Urn:lsid:marinespecies.org:taxname:921.

**Diagnosis (Level 3).** Body regionalization present as a thorax and abdomen; segments similar dimensions throughout (Fig. 18A, I); discrete head lobe-like without appendages, bluntly conical (Fig. 18A, I).

**Description.** See Suppl. material 1.

**Remarks.**Capitellidae were once grouped with Clitellata (Rouse et al. 2022) as they are similar in external and internal (pharynx) morphology. Then followed a long association with sedentary polychaetes until recently when molecular phylogenetic studies revealed a sister grouping with Thalassematidae (formerly Echiura) within Sedentaria. The family is represented by 42 genera and 224 species (WoRMS 2025), and is widely distributed around the world. Warren et al. (1994) revised the genus *Mediomastus* Hartman, 1944. Gravina and Somaschini (1990) and Green (2002) provide keys to genera known at the time. Gil (2011) provides a revised key to European genera. Silva et al. (2017) provide a key to *Capitella* Blainville, 1828 species of the world, Silva and Amaral (2019) provide a well-illustrated key to *Scyphoproctus* Gravier, 1904 species of the world, and García-Garza et al. (2019) provide a world checklist of *Notomastus* M. Sars, 1851 and a key to its species from the Gulf of California. Hernández-Alcántara et al. (2022) provide a key to species of *Notomastus* with hooded hooks in thoracic chaetigers.

**Environment and habitat.** Aquatic, marine or brackish (very rarely freshwater); coastal, continental shelf or deep sea; soft substrata.

#### ﻿Chaetopteridae Audouin & Milne Edwards, 1833 [polychaete]

Fig. 19

**Common name.** Parchment-tube worms (benthic *Chaetopterus* Cuvier, 1830 only); paddle worms, spindle worms, pigbutt worm (one holopelagic species).

**LSID.** Urn:lsid:marinespecies.org:taxname:918.

**Figures 19, 20.**
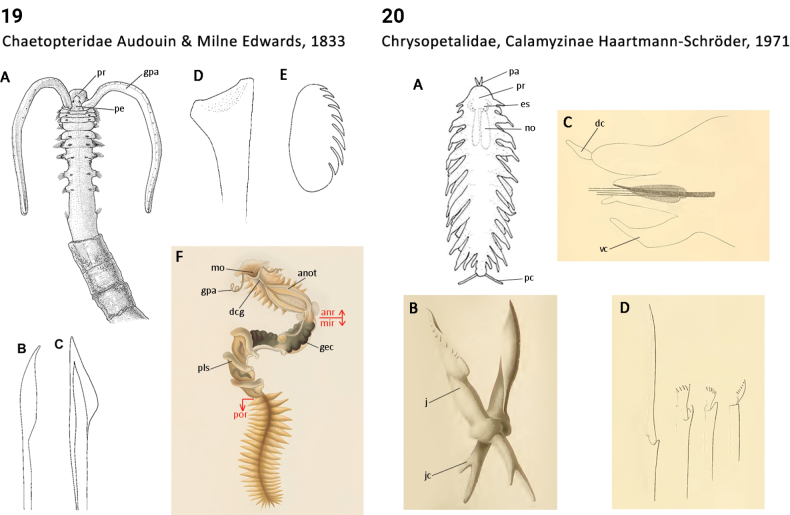
Distinguishing features: **19.**Chaetopteridae: **A–D.***Spiochaetopterus* sp.: **A.** Anterior end of worm extended from its tube; **B, C.** Long notochaetae from chaetiger 6; **D.** ‘Cup’ chaeta from chaetiger 4; **E.** Uncinus from chaetiger 7; **F.***Chaetopterusvariopedatus*, entire animal. Abbreviations: anot ailiform notopodia anr anterior region dcg dorsal ciliary groove gec green epithelial cells gpa grooved palp mir mid region mo mouth pe peristomium pls piston-like segment por posterior region pr prostomium. Sources: **A–E** after fig. 1.104 Beesley et al. (2000), **F** after MacIntosh (1900–1922), pl. LXXXIX, fig. 3. **20.**Chrysopetalidae, Calamyzinae: *Ichthyotomussanguinarius*: **A.** Complete juvenile individual 1 mm long; **B.** Stylet jaws in open position; **C.** Mid-body parapodium; **D.** Neuropodial spiniger and falcigers. Abbreviations: dc dorsal cirrus es eye spot j jaw jc jaw carrier no nuchal organ pa palp pr prostomium vc ventral cirrus. Sources: **A** derivative of fig. 1 Watson (2022), **B–D** derivatives of text fig. 13, plate 1, fig. 12, plate 6, fig. 3b Eisig (1906).

**Diagnosis (Level 3).** Body elongate, comprising three regions (Fig. 19F); prostomium rounded to oval (Fig. 19A); peristomium visible, as a single ring, collar-like (Fig. 19A); palps present, grooved (Fig. 19A, gpa); spines present (Fig. 19D); neuropodial lobes low ridges (tori) bearing uncini (Fig. 19E).

**Description.** See Suppl. material 1.

**Remarks.**Chaetopteridae is represented by four genera and 83 species (WoRMS 2025) and has a worldwide distribution in benthic and holopelagic environments. Like Apistobranchidae, the family has been allied with spioniform families until recently, when molecular evidence revealed its association with Apistobranchidae; the two families share the presence of aciculae, which is unusual for Sedentaria (see Rouse et al. 2022). Holopelagic species like *Chaetopteruspugaporcinus* Osborn, Rouse, Goffredi & Robison, 2007 differ from their benthic cousins in their highly modified bodies and may not be identifiable with the present dataset. Blake (1996b) and Gil (2011) provide keys to European genera. Sun and Qiu (2014) provide a key to *Chaetopterus* from the Pacific region, and Wang and Li (2017) provide a key to Pacific species of *Phyllochaetopterus* Grube, 1863.

**Environment and habitat.** Aquatic, marine; coastal, continental shelf, or deep sea; soft or hard substrata, including cold seeps (not presently known from hydrothermal vents), or holopelagic.

#### ﻿Chrysopetalidae Ehlers, 1864, sensu lato [polychaete]

**Common name.** None.

**LSID.** Urn:lsid:marinespecies.org:taxname:944.

**Diagnosis (Level 3).** Dorsal body surface usually with a protective covering of shield-like spines (paleae); capillary chaetae absent; prostomial antennae present, one median and pair of lateral ones; pharynx jaws present.

**Description.** See Suppl. material 1.

**Remarks.**Chrysopetalidae shows great morphological diversity, so we have provided subfamily coding to reduce polymorphism in the dataset (only the family was coded in POLiKEY; Glasby and Fauchald 2003). The broad concept of the family now comprises 31 genera and 113 species (WoRMS 2025), but a much narrower version was for many years associated with Phyllodocida, particularly nereidiforms. During the last ten years or so, Chrysopetalidae has expanded considerably. Aguado et al. (2013) found that the mostly parasitic Calamyzidae and the deep-sea bivalve endosymbionts Nautiliniellidae were morphologically and genetically allied to Chrysopetalidae. Finally, Rouse et al. (2022) moved the single parasitic species comprising Ichthyotomidae to Chrysopetalidae on the basis of morphological similarity and grouped all members of Chrysopetalidae sensu lato within Hesionoidea. The family comprises three clades (Rouse et al. 2022), which are here recognized as subfamilies: Calamyzinae (containing Ichthyotomidae and Nautiliniellidae), Chrysopetalinae and Dysponetinae. San Martín (2004) provides a key to chrysopetaline genera at the time. Gil (2011) provides an updated key to European taxa and Parapar et al. (2018) provides a key to European Calamyzinae. Cruz-Gómez (2021) provides a checklist of all chrysopetalids species (including calamyzines) recorded from the Tropical East Pacific.

**Environment and habitat.** Aquatic, marine; coastal, continental shelf, or deep sea; soft or hard substrata, hydrothermal vents, and cold seeps or epizoic.

#### ﻿Chrysopetalidae, Calamyzinae Hartmann-Schröder, 1971 [polychaete]

Fig. 20

**Common name.** None.

**LSID.** Urn:lsid:marinespecies.org:taxname:949.

**Diagnosis (Level 3).** Body surface lacking protective covering (paleae) (Fig. 20A, C); prostomium with antennae absent and sensory palps present, unarticulated (Fig. 20A, pa).

**Description.** See Suppl. material 1.

**Remarks.**Calamyzinae (containing the now defunct Ichthyotomidae and Nautiliniellidae; coded as families in POLiKEY, Glasby and Fauchald 2003) is a poorly known, mostly parasitic group comprising 18 genera and 26 species, including an unusually high number of monotypic genera (WoRMS 2025). Databased distributional records are few and include records from South America (*Ichthyotomus* Eisig, 1906), and North America (Nautiliniellidae) only (GBIF.org 2023). Identification keys for the subfamily are lacking.

**Environment and habitat.** Aquatic, marine; coastal, continental shelf or deep sea; soft or hard substrata, hydrothermal vents, and cold seeps or epizoic.

#### ﻿Chrysopetalidae, Chrysopetalinae Ehlers, 1864 [polychaete]

Fig. 21

**Common name.** Golden petal worm.

**LSID.** Urn:lsid:marinespecies.org:taxname:744405.

**Figures 21, 22.**
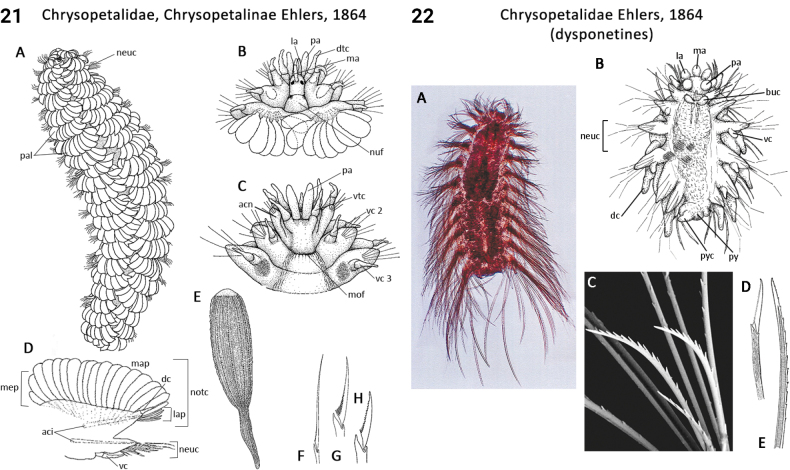
Distinguishing features: **21.**Chrysopetalidae, Chrysopetalinae: **A.***Paleanotus* sp. entire animal dorsal view; **B–H.***Paleaequorsetula*: **B.** Anterior end dorsal view; paleae on chaetigers 1 and 2 removed; **C.** Anterior end ventral view; **D.** Parapodium from chaetiger 30; notochaetal paleal groups shown; **E.** Main palea from the midbody showing internal and external structure; **F–H.** Compound neurochaetae from chaetiger 34: **F.** Superior spiniger; **G.** Mid-superior falciger; **H.** Mid-inferior falciger. Abbreviations: aci aciculae acn acirrose neuropodium segment dc dorsal cirrus dtc dorsal tentacular cirri la lateral antenna lap lateral paleae ma median antenna map main paleae mep median paleae mof mouth fold neuc neurochaetae notc notochaetae nuf nuchal fold pa palp pal parapodial palea vc ventral cirrus vc2 ventral cirrus, chaetiger 2 vc ventral cirrus, chaetiger 3 vtc ventral tentacular cirri. Sources: **A–H** after fig. 1.71 Beesley et al. (2000). **22.**Chrysopetalidae, Dysponetinae: **A.***Dysponetuscaecus*, anterior end, dorsal view; **B, D, E.***Dysponetuspygmaeus*: **B.** 7-segmented juvenile ventral view; **D.** Neurochaetae; **E.** Notochaetae; **C.** Notochaetae. Abbreviations: buc buccal cirrus, dc dorsal cirrus, la lateral antenna, ma median antenna, neuc neurochaetae, pa palp, py pygidium, pyc pygidial cirrus. Sources: **A** derivative of fig. 3 Watson et al. (2014), **B** derivative of fig. 27.1 Bhaud and Cazaux (1987), **C** derivative of Handbook of Zoology, Watson (2022), **D, E** derivatives of Pl. 10, Imajima and Hartman (1964).

**Diagnosis (Level 3).** Body surface with protective covering of shield-like spines (paleae); paleate notochaetae present (Fig. 21D, E; map, mep) (Fig. 21A–C); nuchal organs unpaired caruncle or nuchal fold (Fig. 21B; nuf); spines (spine-like paleae) in both dorsal and ventral positions (Fig. 21D, lap).

**Description.** See Suppl. material 1.

**Remarks.**Chrysopetalinae is the largest, and most widespread subfamily of Chrysopetalidae, comprising 12 genera and ~ 65 species (GBIF.org 2023; WoRMS 2025).

**Environment and habitat.** Aquatic, marine; coastal, continental shelf, or deep sea; soft or hard substrata, or hydrothermal vents and cold seeps.

#### ﻿Chrysopetalidae, Dysponetinae Aguado, Nygren & Rouse, 2013 [polychaete]

Fig. 22

**Common name.** None.

**LSID.** Urn:lsid:marinespecies.org:taxname:744406.

**Diagnosis (Level 3).** Body surface with protective covering shield-like spines, spines in dorsal position only (Fig. 22A, C); paleate chaetae absent; prostomium bearing antennae and bi-articulated sensory palps (Fig. 22B, la, ma, pa); nuchal organs as paired low projections.

**Description.** See Suppl. material 1.

**Remarks.**Dysponetinae comprises a single genus, *Dysponetus*, and 16 species (WoRMS 2025), distributed around the world, although occurrences are patchy (GBIF.org 2023) probably due to sampling bias. Darbyshire and Brewin (2015) compare three newly described species with other members of the genus.

**Environment and habitat.** Aquatic, marine; coastal, continental shelf or deep sea; soft or hard substrata.

#### ﻿Cirratulidae Ryckholt, 1851 [polychaete]

Fig. 23

**Common name.** None.

**LSID.** Urn:lsid:marinespecies.org:taxname:919.

**Figures 23, 24.**
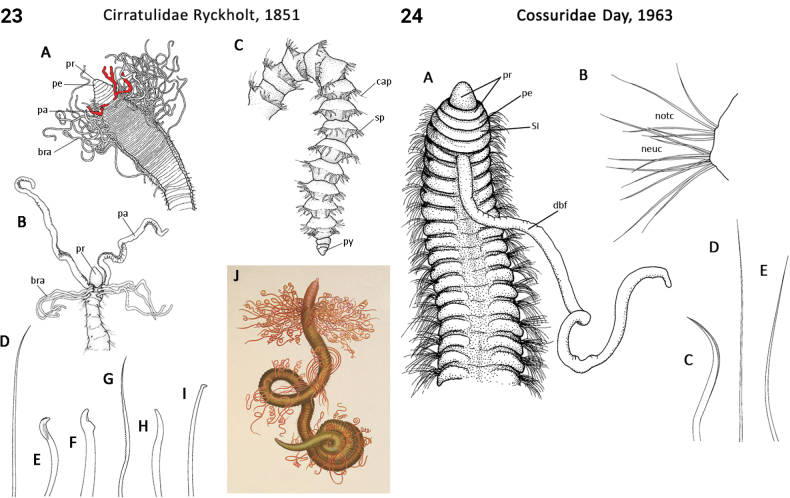
Distinguishing features: **23.**Cirratulidae: **A.** Entire animal of Cirriformiacf.filigera dorsal view; **B.** Anterior end of *Dodecaceria* species dorsal view; **C.** Posterior end of *Chaetozonesetosa*; **D–I.** Chaetae: **D.** Capillary notochaeta from mid-body chaetiger *Cirratulus* species; **E.** Spine from mid-body neuropodia of *Caullerielladimorphosetosa*; **F.** Hook from mid-body neuropodia *Dodecaceria* species; **G.** Capillary notochaeta from posterior chaetiger *Monticellinaaphelocephalus*; **H.** Spine from posterior chaetiger of *Chaetozonesetosa*; **I.** Spine from posterior chaetiger of *Tharyxlongisetosa*; **J.***Cirriformiatentaculata*, entire animal. Abbreviations: bra branchia cap capillary chaetae pa palp pe peristomium pr prostomium py pygidium sp spine. Sources: **A–I** after fig. 1.113 Beesley et al. (2000), **J** after McIntosh (1900–1922), pl. XCI, fig. 1. **24.**Cossuridae: *Cossura* sp. **A.** Anterior end dorsal view; **B.** Parapodium of chaetiger 18; **C.** Neurochaeta from parapodium of chaetiger 8; **D.** Neurochaeta from parapodium of chaetiger 23; **E.** Posterior abdominal chaeta. Abbreviations: dbf dorsal branchial filament neuc neurochaetae notc notochaetae pe peristomium pr prostomium SI segment 1. Sources: **A–E** after fig. 1.50 Beesley et al. (2000).

**Diagnosis (Level 3).** Body lacking regionalization, epidermis more-or-less smooth (Fig. 23A, J); discrete head, lobe-like without appendages, prostomium conical, palps present, anterodorsal (Fig. 23A, B, pa); chaetae first appear on second segment after peristomium; capillary chaetae present (Fig. 23D, G), spines of various types present including hook-like types (Fig. 23E, F, H, I); branchiae present (Fig. 23A, B, J).

**Description.** See Suppl. material 1.

**Remarks.**Cirratulidae is paraphyletic without the inclusion of Ctenodrilidae (Rouse et al. 2022); however, we have treated the two families separately to facilitate identification, particularly because the former is easily distinguished by having palps, whereas the latter does not. The distinction has also been maintained by Blake and Magalhães (2019), who reported 15 genera and over 160 described species of Cirratulidae distributed worldwide (12 genera and 390 species according to WoRMS (2025)). Keys to genera are provided in Blake (2018) and Çinar and Petersen (2011) provide a key to multi-tentaculate genera. Gil (2011) provides an updated key to European taxa, including Ctenodrilinae and Raphidrilinae.

**Environment and habitat.** Aquatic, marine; coastal, continental shelf or deep sea; soft or hard substrata, or epizoic (on mollusk shells or coralline algae).

#### ﻿Cossuridae Day, 1963 [polychaete]

Fig. 24

**Common name.** None.

**LSID.** Urn:lsid:marinespecies.org:taxname:908.

**Diagnosis (Level 3).** Body shape elongate, body regionalization absent; discrete head lobe-like without appendages (Fig. 24A); buccal tentacles present (rarely exposed), ciliated; first chaetiger with notochaetae only; branchia present (single long dorsal filament on anterior chaetiger) (Fig. 24A; dbf).

**Description.** See Suppl. material 1.

**Remarks.**Cossuridae is currently represented by a single genus, *Cossura* Webster & Benedict, 1887, and 30 species (WoRMS 2025). The family is poorly known biologically and taxonomically, which is reflected in its current position of Sedentaria incertae sedis Rouse et al. (2022). They are distributed worldwide but appear to be species-poor in the deep sea (GBIF 2023). Gil (2011) and Parapar et al. (2012) provide keys to European taxa, Read (2000) and Sousa et al. (2019) provide useful tabulated comparisons of all species.

**Environment and habitat.** Aquatic, marine; coastal, continental shelf or deep sea; soft substrata.

#### ﻿Criodrilidae Vejdovskì, 1884 [megadrile]

Fig. 25

**Common name.** None.

**LSID.** Urn:lsid:marinespecies.org:taxname:1039995.

**Figures 25, 26.**
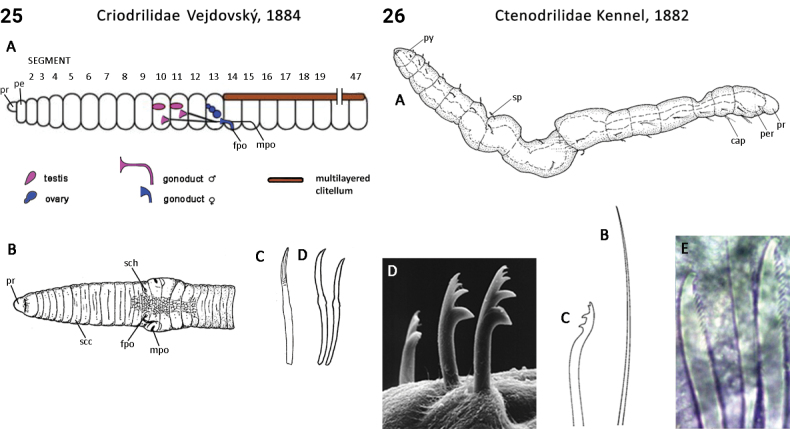
Distinguishing features: **25.**Criodrilidae: **A.** Diagram of reproductive organs dorsal side up; **B–D.***Criodriluslacuum*: **B.** Anterior body of sexually mature specimen in ventral view with glandular pads, beginning of clitellum and a spermatophore; **C.** Spermathecal chaeta; **D.** Chaetal bundle, paired simple-pointed crotchets. Abbreviations: fpo female pore sch spermathecal chaeta mpo male pore pe peristomium pr prostomium scc simple crochet chaeta. Sources: **A** derivative of fig. 8.4, Jamieson (2006), **B–D** derivatives of fig. 12.2, 12.28, 12.3 Thorp et al. (2019). **26.**Ctenodrilidae: **A–C.***Aphropharynx* sp.: **A.** Lateral view of entire animal; **B.** Capillary chaeta from parapodium of chaetiger 12; **C.** Multidentate spine from parapodium of chaetiger 12; **D.**Ctenodriluscf.serratus, chaetal hooks; **E.***Raricirrusvariabilis*, pectinate chaetiger. Abbreviations: cap capillary chaetae pe peristomium pr prostomium py pygidium sp spine. Sources: **A–D** after fig. 1.114 Beesley et al. (2000), **E** derivative of fig. 7.15.5.2 Dean and Blake (2019).

**Diagnosis (Level 1).** Tubercula pubertatis absent; clitellum situated in region of male pores (Fig. 25A).

**Remarks.**Criodrilidae includes two species in a single genus, *Criodrilus* Hoffmeister, 1845, which is native to the western Palaearctic (Misirlioğlu et al. 2023); many more generic and species names are available but are currently considered not valid (WoRMS 2025). Criodrilidae is similar to Ocnerodrilidae and only distinguishable at DELTA Diagnostic Level 1.

**Environment and habitat.** Terrestrial or aquatic, freshwater.

#### ﻿Ctenodrilidae Kennel, 1882 [polychaete]

Fig. 26

**Common name.** None.

**LSID.** Urn:lsid:marinespecies.org:taxname:905.

**Diagnosis (Level 3).** Body regionalization absent; discrete head lobe-like without appendages (Fig. 26A); prostomium bluntly conical; palps absent (Fig. 26A); chaetae first appear on second segment after peristomium; capillary chaetae (Fig. 26A, B, cap) in both dorsal and ventral positions; spines present, with small teeth or spinelets or sharply bent (= geniculate) or recurved (Fig. 26C–E).

**Description.** See Suppl. material 1.

**Remarks.**Ctenodrilidae is included as a subfamily (Ctenodrilinae) of Cirratulidae in WoRMS (2025) and Rouse et al. (2022) but treated as a separate family in the Handbook of Zoology (Dean and Blake 2019). We follow the latter authors simply because ctenodrilids are morphologically distinguishable from other cirratulids in lacking palps, among other features (it was also treated as a family in POLiKEY; Glasby and Fauchald 2003). The family, in this sense, contains four genera (*Aphropharynx* Wilfert, 1974, *Ctenodrilus* Claparède, 1863, *Raphidrilus* Monticelli, 1910, *Raricirrus* Hartman, 1961) and 17 species (WoRMS 2025), and is distributed worldwide although deep-sea records are rare (GBIF.org 2023). Petersen and George (1991) provide a key to genera. Gil (2011) provides an updated key to European taxa and Magalhães et al. (2011) provide a key to all genera of Ctenodrilidae and species of *Raphidrilus*.

**Environment and habitat.** Aquatic; marine; coastal or continental shelf; soft substrata.

#### ﻿Cyclobdellidae Ringuelet, 1972b [leech]

**Common name.** None.

**Diagnosis (Level 1).** Dorsoventrally flattened; anterior end sucker with large mouth on ventral surface; eyes on head present; jaws present, one row of denticles (monostichodont); egg sacs tubular.

**Description.** See Suppl. material 1.

**Remarks.**Cyclobdellidae is a doubtfully valid family of jawed Hirudiniformes, represented by a single genus and three species, which are endemic to South America (Christoffersen 2008). The family is very similar to Praobdellidae and only distinguishable at DELTA Diagnostic Level 1. Taxonomic literature on the family is scarce. Christoffersen (2008) listed the taxa from South America.

**Environment and habitat.** Unknown.

#### ﻿Cylicobdellidae Ringuelet, 1972a [leech]

**Common name.** None.

**LSID.** Urn:lsid:marinespecies.org/aphia.php?p = taxdetails&id = 1603546.

**Diagnosis (Level 2).** Body pigmentation present; eyes on head absent; pharyngeal ridges rotated 60° to the right (strepsilaematous); mid-body secondary annulation, 5-annulate; gonadal segments lacking a copulatory area for sperm transfer; male atrium bilobed.

**Description.** See Suppl. material 1.

**Remarks.**Cylicobdellidae is a poorly-known jawless erpodelliform family (Siddall et al. 2006), known only for two genera (*Cylicobdella* Grube, 1871 and *Blanchardiella* Weber, 1914) and four and 11 species respectively from South America and the Caribbean; the type genus/species of the family is *Cylicobdellalumbricoides* Grube, 1871 (Christoffersen 2008; WoRMS 2025). See Siddall (1995) for an opinion on the validity of the family.

**Environment and habitat.** Terrestrial, soil; soft substrata.

#### ﻿Dinophilidae Macalister, 1876 [polychaete]

Fig. 27

**Common name.** None.

**LSID.** Urn:lsid:marinespecies.org:taxname:155089.

**Figures 27, 28.**
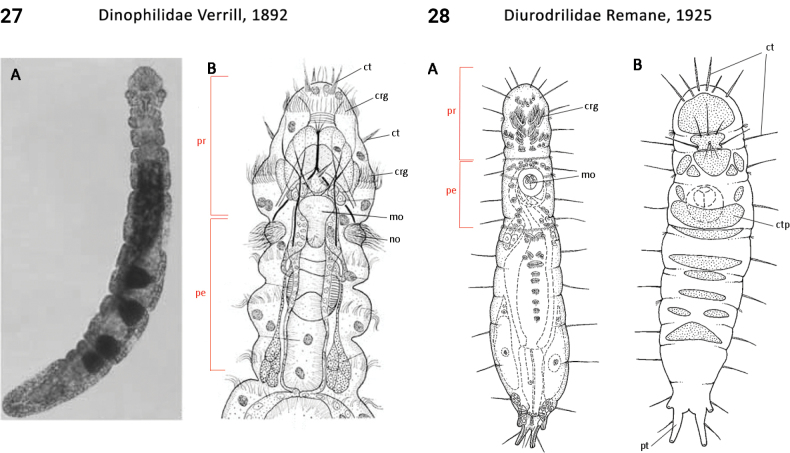
Distinguishing features: **27.**Dinophilidae: **A.***Trilobodrilusaxi* live specimen, mature female; **B.***Trilobodrilus* species, details of head, dorsal view. Abbreviations: crg ciliary ring, ct ciliary tuft, mo mouth, no nuchal organ, pe peristomium, pr prostomium. Sources: **A, B** derivatives of fig. 1B, fig. 2A Westheide (2019), respectively. **28.**Diurodrilidae: **A, B.***Diurodrilus* sp.: **A.** Entire adult female, ventral view; **B.** Entire young female, dorsal view showing ‘cuticular plates’. Abbreviations: crg ciliary ring, ct ciliary tuft, ctp cuticular plate, mo mouth, pe peristomium, pr prostomium, pt pygidial toe. Sources: **A, B** after fig. 1.64 Beesley et al. (2000).

**Diagnosis (Level 3).** Body short with fixed number of segments, secondary annulation present (Fig. 27A); discrete head lobe-like without appendages (Fig. 27A, B); peristomium not visible; chaetae absent.

**Description.** See Suppl. material 1.

**Remarks.**Dinophilidae, comprising three genera and 19 species (WoRMS 2025), was considered until recently to be part of Dorvilleidae (Rouse and Pleijel 2001; Glasby and Fauchald 2003), a family renowned for containing paedomorphic species; however, based on recent phylogenomic evidence it is now considered to belong within Sedentaria (Rouse et al. 2022), meaning that the idea that paedomorphosis occurs in the family needs to be reviewed. One aberrant dinophilid, *Apharyngtuspunicus* Westheide, 1971, thought to represent a separate lineage from other dinophilids, has been placed in its own family, Apharyngtidae Worsaae, Kerbl, Di Domenico, Gonzalez, Bekkouche & Martínez, 2021 (Worsaae et al. 2021), but has recently been allocated to Diurodrilidae (see next). Dwarf males are known for *Dinophilus* Schmidt, 1848, and these may not be identifiable using this dataset. Dinophilidae are currently only known from the Northern Hemisphere (GBIF.org 2023). See the Dorvilleidae treatment for literature containing keys to dinophilid taxa.

**Environment and habitat.** Aquatic, marine; coastal, littoral or supralittoral; soft or hard substrata.

#### ﻿Diurodrilidae Kristensen & Niilonen, 1982 [polychaete]

Fig. 28

**Common name.** None.

**LSID.** Urn:lsid:marinespecies.org:taxname:18912.

**Diagnosis (Level 3).** Body segmentation, parapodia and chaetae absent (ciliary tufts resemble fine chaetae) (Fig. 28A, B); nuchal organs absent; discrete ovaries present; gut more-or-less straight, lacking side branches (Fig. 28A).

**Description.** See Suppl. material 1.

**Remarks.**Diurodrilidae is a poorly known family, now thought to contain two genera, *Diurodrilus* Remane, 1925 and *Apharyngtus* (Rouse et al. 2022) and eight species (WoRMS 2025). Its composition and position have varied over the years, from being part of Dinophilidae (*Apharyngtus* only), Dorvilleidae, belonging to Orbiniida (Struck 2019), and even being outside of Annelida (Rouse et al. 2022, and references therein). Herein, we consider the family as part of Polychaeta by default, as they have never been affiliated with any other annelid group. Diurodrilidae is only known from the Northern Hemisphere (GBIF.org 2023). The key to Dorvilleidae in Gil (2011) includes the two current diurodrilid genera.

**Environment and habitat.** Aquatic, marine or brackish; coastal; soft substrata (usually coarse sands of beaches).

#### ﻿Dorvilleidae Chamberlin, 1919 [polychaete]

Fig. 29

**Common name.** None.

**LSID.** Urn:lsid:marinespecies.org:taxname:971.

**Figures 29, 30.**
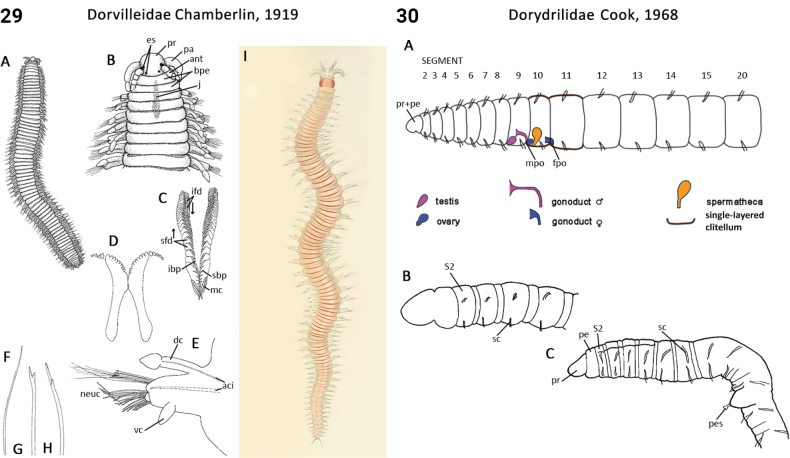
Distinguishing features: **29.**Dorvilleidae: **A–H.***Schistomeringosloveni*: **A.** Entire animal dorsal view; **B.** Anterior end dorsal view; **C, D.** Jaw parts: **C.** Maxillae dorsal view; **D.** Mandibles ventral view; **E.** Parapodium from chaetiger 10; **F–H.** Chaetae from parapodium of chaetiger 10: **F.** Simple chaeta; **G.** Furcate chaeta; **H.** Compound falciger; **I.***Dorvillearubrovittata* entire animal. Abbreviations: aci aciculae bpe biannulate peristomium dc dorsal cirrus es eye spot ibp inferior base plate ifd inferior free denticle j jaw mc marginal cirrus neuc neurochaetae pa palp pr prostomium sbp superior base plate sfd superior free denticle vc ventral cirrus. Sources: **A–H** after figs 1.58 A–H, Beesley et al. (2000), **I** after MacIntosh (1900–1922), pl. LV, fig. 1. **30.**Dorydrilidae: **A.** Schematic diagram showing location of reproductive organs, dorsal side up; **B.***Dorydrilustetrathecus*, anterior end; **C.***Dorydrilusmichaelseni*, side view of anterior body with a protruded penis. Abbreviations: fpo female pore mpo male pore pe peristomium pes penis sheath pr prostomium S segment sc sigmoid chaeta. Sources: **A** derivative of fig. 12.3 Thorp et al. (2019). **B, C** derivatives of Timm (2009), fig. 135.

**Diagnosis (Level 3).** Discrete head bearing appendages, prostomium rounded to oval, anteriorly not indented or with projections (Fig. 29A, B, I); peristomium a double ring (Fig. 29B; bpe); first segment chaetous, parapodia uniramous (Fig. 29B, E); hooks absent; pharynx bearing multiple jaws and free denticles (Fig. 29B–D).

**Description.** See Suppl. material 1.

**Remarks.**Dorvilleidae is a morphologically heterogeneous group that was treated as three subgroups (Dorvilleidae subgroups 1, 2, 3) in POLiKEY (Glasby and Fauchald 2003). In the present dataset we treat the family in its broad sense (including Iphitimidae), essentially the same concept as used in Rouse et al. (2022). As such, the family is more polymorphic than most annelid families, meaning that specimens of some highly modified genera may not be identifiable as a dorvilleid. The family contains 31 genera and 227 species (WoRMS 2025), which are distributed worldwide. Campoy and San Martín (1980) and Eibye-Jacobsen and Kristensen (1994) provide a key to genera, although the latter contains members of Dinophilidae, which are now considered a separate family. The Dorvilleidae of Gil (2011) includes both Dinophilidae and Diurodrilidae. Wiklund et al. (2021) provide the most recent key to genera.

**Environment and habitat.** Aquatic, marine, coastal, continental shelf, or deep sea; hard or soft substrata, hydrothermal vents, and cold seeps or epizoic.

#### ﻿Dorydrilidae Cook, 1968 [microdrile]

Fig. 30

**Common name.** None.

**LSID.** Urn:lsid:marinespecies.org:taxname:1039996.

**Diagnosis (Level 0).** Chaetae present, two per bundle (Fig. 30A, sc); hair chaetae absent; simple crotchet chaetae present; male pores in segment following testicular segment (plesioporous); nephridial pores and gonoducts located around clitellum (Fig. 30A).

**Description.** See Suppl. material 1.

**Remarks.**Dorydrilidae is indistinguishable from the large and morphologically variable Naididae using the present dataset, and differs from the mostly Gondwanan Phreodrilidae at DELTA Diagnostic Level 1 (see Remarks under Naididae for further comments). The reader is referred to the full description to verify identification. The plesioporous male duct in Dorydrilidae has probably arisen with the reduction of the posterior pair of testes and vasa deferentia (T. Timm, pers. comm., Jan. 2025), and details of the male reproductive system may prove useful for distinguishing closely similar families. Dorydrilidae is known for a single genus and three species (all from Europe, including Germany, Switzerland, France, Austria, United Kingdom) and an unpublished occurrence in Spain (PM pers. obs.; WoRMS 2025). Brinkhurst and Jamieson (1971) provide keys to genera and species at the time.

**Environment and habitat.** Aquatic, freshwater (including subterranean waters).

#### ﻿Enchytraeidae d’Udekum, 1855 [microdrile]

Fig. 31

**Common name.** Potworms.

**LSID.** Urn:lsid:marinespecies.org:taxname:2038.

**Diagnosis (Level 0).** Crotchet chaetae almost always simple-pointed, short; if bifid, then upper tooth shorter; crotchets mostly lacking a nodulus; genital chaetae absent; testes, one pair; male pores in segment following testicular segment (plesioporous); spermathecal pores located well anterior to male pores.

**Figures 31, 32.**
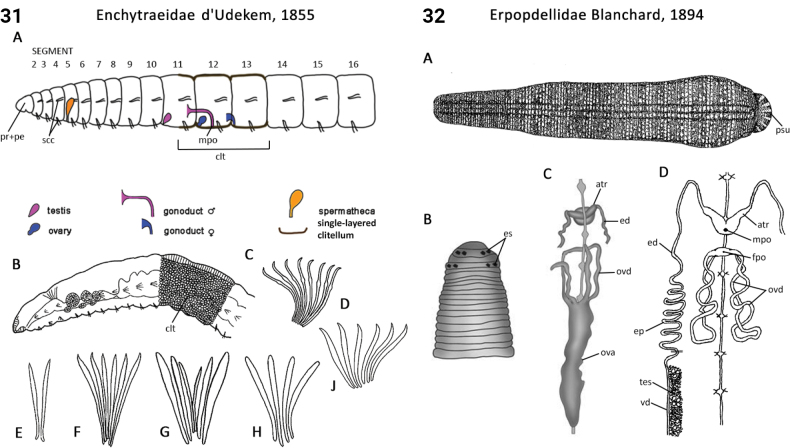
Distinguishing features: **31.**Enchytraeidae: **A.** Schematic diagram showing location of reproductive organs, dorsal side up; **B.***Enchytraeusalbidus* anterior body of sexually mature specimen with clitellum; **C–J.** Chaetae types. Abbreviations: clt clitellum mpo male pore pe peristomium pr prostomium scc simple crochet chaeta. Sources: **A** derivative of fig. 12.3 Thorp (2019), **B–J** derivatives of Timm (2009) figs 139, 161. **32.**Erpobdellidae: **A.***Dina orientalis*, dorsal view; **B.** Head end, dorsal view showing arrangement of eyespots in *Erpobdellaoctoculata*; **C.** Reproductive system of *Erpobdellacostata*; **D.** Reproductive system of *Motobdellasedonensis*. Abbreviations: atr atrium ed ejaculatory duct ep epididymus fpo female pore mpo male pore ova ovary ovd oviduct psu posterior sucker tes testis vd vas deferens. Sources: **A** after fig. 1 Grosser et al. (2011), **B, C** after fig. 2 Oceguera-Figueroa et al. 2010, **D** after fig. 3 Govedich et al. 1998.

**Description.** See Suppl. material 1.

**Remarks.**Enchytraeidae cannot be separated from the monogeneric Propappidae at DELTA Diagnostic Level 1; however, fine details of the crotchet chaetae may prove sufficient to differentiate the two families (see full descriptions of each). Enchytraeidae is cosmopolitan and perhaps the most widely occurring clitellate family, although relatively less common in the tropics (Martin et al. 2008; Erséus et al. 2010b). The family occupies primarily moist terrestrial habitats including groundwater and moist forest soils, but also inhabits marine sediments. Marine enchytraeids mainly occur in the upper intertidal, but some genera (e.g., *Grania* Southern, 1913) are widespread in offshore benthos (Erséus and Healy 2001). Globally there are 821 species in 36 genera (WoRMS 2025). Brinkhurst and Jamieson (1971) provide keys to global genera and species at that time, Schmelz and Collado (2010) provide keys to European terrestrial and freshwater taxa and Erséus and Healy (2001) provide a key to marine taxa. Locke and Coates (2000) provide a key using anatomical characters to distinguish nine species of *Grania* and two species of *Randidrilus* Coates & Erséus, 1985 in eastern North America and the Caribbean. Worsfold (2003) provides an introduction and key to oligochaetes, including Enchytraeidae, of the NE Atlantic. Enchytraeidae is present in Australia, though its true diversity is grossly underestimated (Pinder in press).

**Environment and habitat.** Terrestrial or aquatic; marine, brackish, or freshwater; coastal, continental shelf, deep sea, littoral, or supralittoral; soft substrata (soil).

#### ﻿Erpobdellidae Blanchard, 1894 [leech]

Fig. 32

**Common name.** None.

**LSID.** Urn:lsid:marinespecies.org:taxname:160005.

**Diagnosis (Level 2).** Body dorso-ventrally flattened and having the typical leech segment number (34 segments including 2 pre-oral ‘segments’ (prostomium and peristomium) and 32 post-oral segments); anterior end sucker present, large mouth (Fig. 32B); eyes on head present; pharyngeal ridges rotated 60° to the right (strepsilaematous); testes numerous, arranged in multiple grape-like clusters per segment.

**Description.** See Suppl. material 1.

**Remarks.**Erpobdellidae is part of the jawless Arhynchobdella. WoRMS (2025) lists six genera (*Archaeobdella* Grimm, 1876, *Dina* Blanchard, 1892, *Erpobdella* Lamarck, 1818, *Fadejewobdella* Lukin, 1962, *Nephelopsis* Verrill, 1872, and *Trocheta* Dutrochet, 1817) and 25 species. Another three genera, *Mooreobdella*, *Motobdella* and *Croatobranchus*, have been included in the family; however, based on a phylogenetic review, Siddall (2002) synonymised all three genera with *Erpobdella*, leaving the family only with the six traditional genera. This taxonomy appears to be widely followed (e.g., Khomenko et al. 2020), and Uttam and Langer (2021) provide an identification key to the approximately 45 species now subsumed within *Erpobdella*. *Erpobdella* is a well-supported monophyletic group mainly found in the Nearctic and Palearctic (Oceguera-Figueroa et al. 2011); three species occur in the Neotropical region (Christoffersen 2008), and one is found in New Zealand. The family has more troglobiont species than any other leech family, with cave species occurring across the Palearctic from southern Europe to China and in the Nearctic (US) (Sket and Trontelj 2008).

**Environment and habitat.** Terrestrial (rarely) or aquatic; moist terrestrial, subterranean or hyporheic (e.g., *Dina*), freshwater; soft substrata.

#### ﻿Eudrilidae Claus, 1880 [megadrile]

Fig. 33

**Common name.** None.

**LSID.** Urn:lsid:marinespecies.org:taxname:998124.

**Figures 33, 34.**
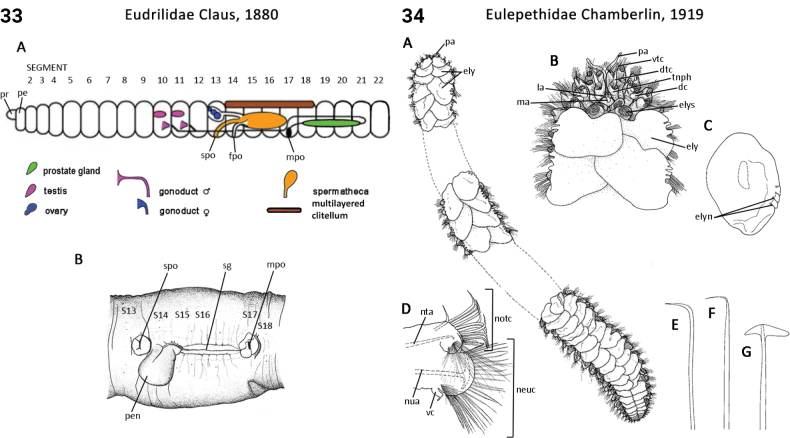
Distinguishing features: **33.**Eudrilidae: **A.** Diagram of reproductive organs, dorsal side up; **B.***Stuhlmanniavariabilis* showing ventral view and seminal groove. Abbreviations: fpo female pore mpo male pore pe peristomium pen penis pr prostomium S segment sg seminal grove spo spermathecal pore. Sources: **A, B** derivatives of fig. 8.4 B, 8.10 Jamieson (2006). **34.**Eulepethidae: *Mexieulepis* sp. **A.** Entire animal dorsal view, anterior, middle and posterior sections; **B.** Dorsal view of anterior end with first two pairs of elytra removed; **C.** Elytron from chaetiger 13; **D.** Parapodium of chaetiger 21 with elytron removed; **E, F.** Chaetae from parapodium of chaetiger 27: **E.** Superior notochaetal spine; **F.** Neurochaetal spine. **G.** Neuroacicula from parapodium of chaetiger 27. Abbreviations: dc dorsal cirrus dtc dorsal tentacular cirrus ely elytron elyn elytron notch elys elytral scar la lateral antenna ma median antenna neuc neurochaetae notc notochaetae nta notoacicula nua neuroacicula pa palp tnph tentaculophore vc ventral cirrus vtc ventral tentacular cirrus. Sources: **A–G** derivative of fig. 1.72 Beesley et al. (2000).

**Diagnosis (Level 3).** Gizzard present; clitellum present, usually situated anterior to male and female pores (Fig. 33B, see also Fig. 33A and Remarks for alternative expressions); spermathecae present, post-testicular; spermathecal pore unpaired; calciferous glands present (Fig. 33A, B).

**Description.** See Suppl. material 1.

**Remarks.**Eudrilidae consists of 45 genera and 305 species distributed natively only in the Afrotropical Realm (Misirlioğlu et al. 2023). Two species in the family are regarded as widely introduced: the African Nightcrawler *Eudriluseugeniae* (Kinberg, 1866), sold as fish bait, is widespread in the tropics and subtropics including Australia (Blakemore 1999) and the more restricted *Hyperiodrilusafricanus* Beddard, 1891, which has spread to Western and Central Africa and Brazil (Misirlioğlu et al. 2023). Hippoperidae is considered a junior synonym of Eudrilidae. Taylor (1949) erected Hippoperidae for some unusual reproductive anatomical features including (1) two pairs of male genital pores in the clitellar segments; (2) a pair of vitelline or nutritive glands whose yolk-cells pass by means of yolk duets into a pair of large sacs; these sacs are connected with the female reproductive system and function as spermathecae; and (3) an extensive closed cavity called a “clitellar pouch”, which covers the floor and, at its widest, extends up the lateral walls of the clitellum. Sims (1987) provides a key to genera of Eudrilidae, excluding *Hyperiodrilus* Beddard, 1891. Clausen (2003) provides a key to all described species of *Libyodrilus* Beddard, 1891. Plisko and Nxele (2015) provide a key to distinguish foreign Eudrilidae taxa from native ones of South Africa.

**Environment and habitat.** Terrestrial (very rarely aquatic).

#### ﻿Eulepethidae Chamberlin, 1919 [polychaete]

Fig. 34

**Common name.** Comb-back scale worms.

**LSID.** Urn:lsid:marinespecies.org:taxname:942.

**Diagnosis (Level 3).** Body dorsoventrally flattened, epidermis more-or-less smooth, dorsal body surface with protective covering scales (elytrae) (Fig. 34A–C); tentacular cirri present (Fig. 34B, dtc, vtc); paired plate-like jaws; paired nuchal organs, which may be barely visible or obvious club-shaped posterior projections; parapodia having axe-head shaped neuroaciculae (Fig. 34D, nua).

**Description.** See Suppl. material 1.

**Remarks.**Eulepethidae is represented by six genera and 24 species (WoRMS 2025), and has a wide distribution, except for the deep sea and Palearctic seas, where records are conspicuously absent (GBIF.org 2023). Pettibone (1969, 1986a) revised and family and provided a key to genera. Barnich and Fiege (2003) provide keys and species descriptions for Mediterranean taxa.

**Environment and habitat.** Aquatic, marine; coastal, continental shelf, or deep sea; soft substrata.

#### ﻿Eunicidae Berthold, 1827 [polychaete]

Fig. 35

**Common name.** Bloodworms (*Marphysa*), palolo worms (*Palola*), Bobbit worms (large-sized *Eunice*), decorator worms (*Diopatra*).

**LSID.** Urn:lsid:marinespecies.org:taxname:966.

**Figures 35, 36.**
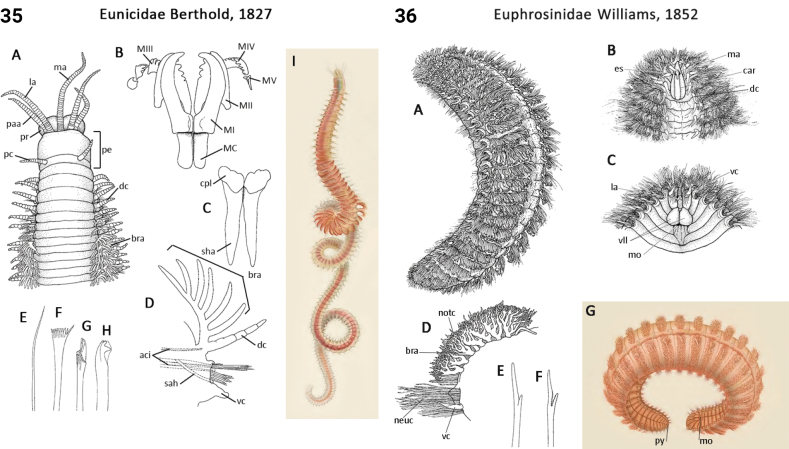
Distinguishing features: **35.**Eunicidae: **A–H.***Euniceantennata*: **A.** Anterior end, dorsal view; **B, C.** Jaw parts: **B.** Maxillae dorsal view; **C.** Mandibles ventral view; **D.** Parapodium from chaetiger 26; **E–H.** Chaetae: **E.** Simple limbate, view from parapodium of chaetiger 41; **H.** Subacicular hook from parapodium of chaetiger 29; **I.***Marphysabelli* entire animal. Abbreviations: aci acicula bra branchia cpl cutting plate dc dorsal cirrus la lateral antenna M maxilla ma median antenna MC maxillary carrier paa palpal antenna pc peristomial cirrus pe peristomium pr prostomium sah subacicular hook sha shaft vc ventral cirrus. Sources: **A–H** derivatives of fig. 1.59 Beesley et al. (2000), **I** derivative of MacIntosh (1900–1922), pl. LV, fig. 5. **36.**Euphrosinidae: **A–C.** An unidentified euphrosinid species: **A.** Entire animal dorsolateral view; **B.** Anterior end dorsal view; **C.** Anterior end ventral view. **D–F.**Euphrosinecf.superba: **D.** Parapodium of chaetiger 19; **E, F.** Furcate and ringent notochaetae from parapodium of chaetiger 32; **G.***Euphrosinefoliosa* dorsal, ventral surfaces. Abbreviations: bra branchia car caruncle dc dorsal cirrus es eye spot la lateral antenna ma median antenna mo mouth neuc neurochaetae notc notochaetae py pygidium vc ventral cirrus vll ventrolateral lip. Sources: **A–F** derivatives of fig. 1.67 Beesley et al. (2000), **G** derivative of MacIntosh (1900–1922; pl. XXIV, fig. 3).

**Diagnosis (Level 3).** Prostomium anteriorly incised, frontal lips absent (Fig. 35A); prostomial antennae present, articulated (less commonly unarticulated) (Fig. 35A, la, ma); peristomium double ring (Fig. 35A; pe); comb-like chaetae present (Fig. 35F); pharynx with jaws (Fig. 35B, C).

**Description.** See Suppl. material 1.

**Remarks.**Eunicidae is represented by 11 genera and 478 species (WoRMS 2025; excluding fossil taxa) and has a worldwide distribution. A key to genera is provided in Carrera-Parra and Salazar-Vallejo (1998) and Zanol et al. (2013); the former key is conveniently partitioned into five *Eunice* Cuvier, 1817 morpho-groups which is most useful. Wu et al. (2013) provide a key to *Eunice* species from China seas, and Hsueh and Li (2014) provide a key to *Eunice* and *Nicidion* Kinberg, 1865 from Taiwan. Gravina et al. (2021) provide a key for Mediterranean species of *Eunice*.

**Environment and habitat.** Aquatic, marine; coastal, continental shelf, or deep sea; soft or hard (usually) substrata.

#### ﻿Euphrosinidae Williams, 1852 [polychaete]

Fig. 36

**Common name.** None.

**LSID.** Urn:lsid:marinespecies.org:taxname:961.

**Diagnosis (Level 3).** Body shape ovate to elliptical (Fig. 36A); prostomium narrow, keel- or ridge-shaped; caruncle present (Fig. 36B); notopodial lobes long dorsal ridges (Fig. 36D, G); lateral branchiae as several tufts per parapodium (Fig. 36D, bra).

**Description.** See Suppl. material 1.

**Remarks.**Euphrosinidae is represented by four genera and 64 species (WoRMS 2025), which have a worldwide distribution. Kudenov (1993) and Parapar et al. (2012) provide keys.

**Environment and habitat.** Aquatic, marine; coastal, continental shelf or deep sea; soft or hard substrata.

#### ﻿Fabriciidae Rioja, 1923 [polychaete]

Fig. 37

**Common name.** Fan worms.

**LSID.** Urn:lsid:marinespecies.org:taxname:154918.

**Figures 37, 38.**
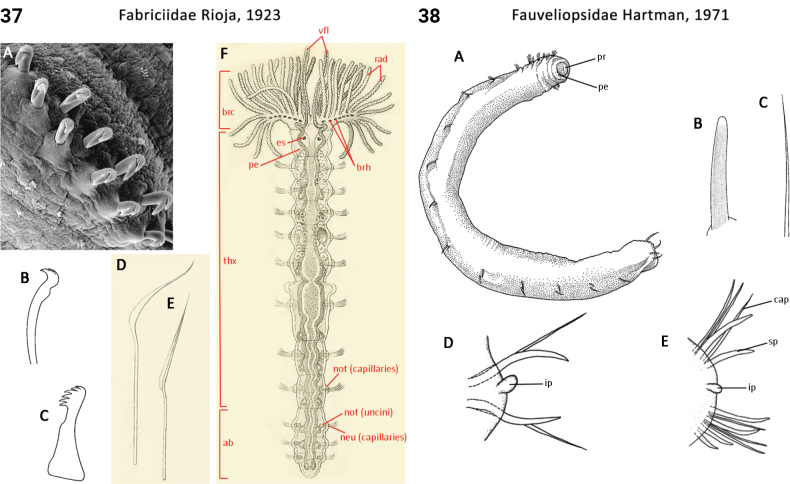
Distinguishing features: **37.**Fabriciidae: **A.***Fabricinuda* sp. thoracic uncini; **B, C.***Fabriciola* sp.: **B.** Neurochaetal uncinus; **C.** Abdominal notochaetal uncinus; **D, E.** Capillary chaetae *Fabricialeidyii*; **F.** Whole body *Manayunkiaspeciosa*. Abbreviations: ab abdomen brh branchial heart es eye spot neu neuropodium not notopodium pe peristomium rad radiole rc radiolar crown thx thorax vfl ventral filamentous appendage. Sources: **A** derivative of fig. 1.100, **B, C** derivatives of fig. 1.99 Beesley et al. (2000), **D–F** derivatives of Leidy (1884 [1883]; pl. IX, figs 1, 4, 14). **38.**Fauveliopsidae: **A.***Fauveliopsis* sp. entire animal ventrolateral view; **B.** Notopodial spine from chaetiger 2; **C.** Simple chaeta from posterior end; **D, E.***Fauveliopsisglabra*: **D.** Parapodium of chaetiger 10; **E.** Parapodium of chaetiger 21. Abbreviations: cap capillary chaetae ip interramal papilla pe peristomium pr prostomium sp spine. Sources: **A–C** derivatives of fig. 1.115 of Beesley et al. (2000), **D, E** derivatives of fig. 7.15.3.7 of Zhadan and Salazar-Vallejo (2019).

**Diagnosis (Level 3).** Thoracic and abdominal body regions demarcated by inversion of parapodia (Fig. 37F); radiolar crown present, including a pair of ventral filamentous appendages (Fig. 37F, vfl) fecal groove absent; pygidium a simple lobe (Fig. 37F).

**Description.** See Suppl. material 1.

**Remarks.**Fabriciidae (as Fabriciinae) were included among Sabellidae in POLiKEY (Glasby and Fauchald 2003). Kupriyanova and Rouse (2008), using a limited molecular dataset, showed that Fabriciinae were closer to Serpulidae than Sabellidae, and elevated the subfamily to family level. Capa et al. (2010) combined morphology with molecular data in their phylogenetic analysis, which supported the sister relationship of Fabriciidae and Serpulidae. Later, Tilic et al. (2020) using a large-scale phylogenomic dataset found that Fabriciidae were actually the sister group of Serpulidae + Sabellidae. Fabriciidae is represented by 19 genera and 89 species (WoRMS 2025) and has a global distribution. Some genera (*Brandtika* Jones, 1974, *Fabricia* Blainville, 1928, *Fabriciola* Friedrich, 1939, *Manayunkia* Leidy, 1859) have colonized fresh and brackish water. Giangrande et al. (2014, 2015) and Cepeda et al. (2022) provide keys and species diagnoses for Mediterranean and Atlantic species.

**Environment and habitat.** Aquatic, marine; brackish or freshwater, coastal or continental shelf; soft substrata.

#### ﻿Fauveliopsidae Hartman, 1971 [polychaete]

Fig. 38

**Common name.** None.

**LSID.** Urn:lsid:marinespecies.org:taxname:978.

**Diagnosis (Level 3).** Discrete head present, retractable into anterior segments (Fig. 38A); interramal fleshy process present (Fig. 38E; ip); capillary chaetae present, in both dorsal and ventral positions (Fig. 38D, E, cap).

**Description.** See Suppl. material 1.

**Remarks.**Fauveliopsidae is represented by three genera and 26 species (WoRMS 2025), and appear to have a global distribution. Petersen (2000) and Salazar-Vallejo et al. (2019c) provide keys to distinguish species and genera. Jimi et al. (2023) provide a key to identify Japanese species of Fauveliopsidae.

**Environment and habitat.** Aquatic, marine; coastal (rarely), continental shelf or deep sea; soft or hard substrata (often found in empty shells of gastropods and scaphopods and tests of foraminiferans).

#### ﻿Flabelligeridae de Saint-Joseph, 1894 [polychaete]

Fig. 39

**Common name.** Bristle-cage worms.

**LSID.** Urn:lsid:marinespecies.org:taxname:976.

**Figures 39, 40.**
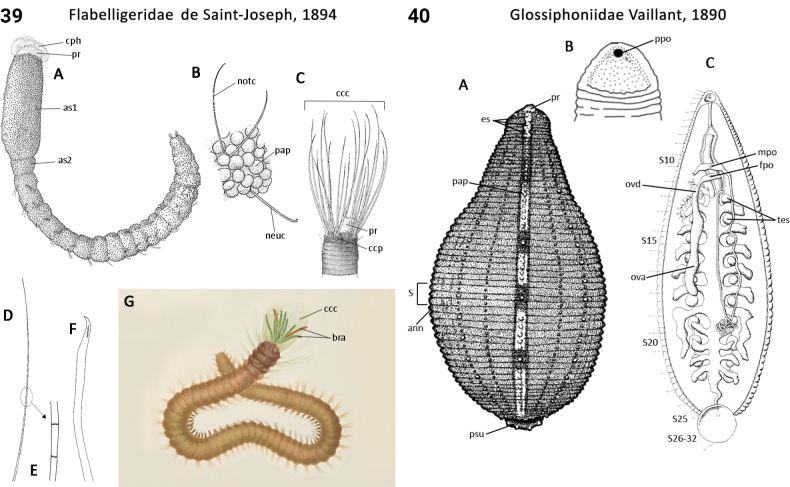
Distinguishing features: **39.**Flabelligeridae: **A, B.***Diplocirrus* sp.: **A.** Entire animal dorsolateral view; **B.** Chaetae and papillae of chaetiger 15; **C.** Anterior end of *Therochaeta* sp. showing the cephalic cage dorsal view; **D–F.** Chaetae from chaetiger 20 of *Pherusa* sp.: **D.** Barred capillary with details of surface shown in **E**; **F.** Spine; **G.***Pherusaplumosa* entire animal. Abbreviations: as1 achaetous segment 1 as2 achaetous segment 2 bra branchia ccc cephalic cage chaetae ccp cephalic cage papillae cph cephalic hood neuc neurochaetae notc notochaetae pap papillae. Sources: **A–F** after fig. 1.116 of Beesley et al. (2000), **G** after MacIntosh (1900–1922; pl. LXXXIX, fig. 1). **40.**Glossiphonidae: **A.***Placobdellacostata*, dorsal view; **B.** Ventral view of head of *Placobdella* sp. showing mouth pore of sucker; **C.** Anatomy of *Glossiphoniacomplanata*. Abbreviations: ann annulus es eye spot fpo female pore mpo male pore ova ovary ovd oviduct pap papillae ppo proboscis pore pr prostomium psu posterior sucker S segment tes testis. Sources: **A** derivative of fig. 1 Sket and Trontelj (2008), **B** derivative of fig. 4 Mann (1962), **C** derivative of fig. 14 Mann (1962).

**Diagnosis (Level 3).** Body with papillated epidermis (Fig. 39A, B, pap); head retractable into anterior segments, obscured by cephalic hood and cephalic cage chaetae (Fig. 39A, C, G; cph, ccc); prostomium bluntly conical (Fig. 39A); peristomium not visible; palps present; second segment achaetous; capillary chaetae appearing barred (pseudosegmented).

**Description.** See Suppl. material 1.

**Remarks.**Flabelligeridae is represented by 25 genera and 248 species (WoRMS 2025) and has a global distribution. The number of taxa in the family has doubled in the last 15 years or so, primarily because of the revisionary studies by Salazar-Vallejo and colleagues (summarized in Salazar-Vallejo 2019), and the incorporation of two holopelagic taxa, Poeobiidae Heath, 1930 and Flotidae Buzhinskaya, 1996, both which were treated as separate families in POLiKEY (Glasby and Fauchald 2003). Flabelligeridae is morphologically diverse reflecting its holopelagic (two genera) and benthic (remaining genera) forms. One genus, *Daylithos* Salazar-Vallejo, 2012, bores into rocks and corals (Salazar-Vallejo 2019). Fauchald (1977), Oug et al. (2011) and Salazar-Vallejo (2017a) provide keys to the benthic genera.

**Environment and habitat.** Aquatic, marine; coastal, continental shelf or deep sea; soft or hard substrata, holopelagic (rarely), or epizoic (rarely).

#### ﻿Gastrostomobdellidae Richardson, 1971 [leech]

**Common name.** None.

**LSID.** Urn:lsid:marinespecies.org/aphia.php?p = taxdetails&id = 1594288.

**Diagnosis (Level 2).** Typical leech segment number (34 segments including two pre-oral ‘segments’ (prostomium and peristomium) and 32 post-oral segments); mid-body secondary annulation, 6-annulate; post-anal secondary annulation, uni-annulate; eyes on head absent; pharyngeal ridges not rotated (euthylaematous); caeca of midgut (= posterior crop caeca) absent; oviduct single, shared oviduct from egg sac; penis absent.

**Description.** See Suppl. material 1.

**Remarks.**Gastrostomobdellidae contained three genera (*Gastrostomobdella* Moore, 1929, *Mimobdella* Blanchard, 1897 and *Orobdella* Oka, 1895) until recently. The family was characterized by, among others, the presence of gastropores on gonadal segments, which open to the crop via two pores (not unique to the family, though). Sawyer (1986) later moved these genera to a subfamily within Cylicobdellidae (Hirudiniformes), which appears not to have been followed. Following investigation using partial nucleic 18S, 28S rDNA and mitochondrial 12S rDNA sequences, Nakano et al. (2012) restricted Gastrostomobdellidae to its type genus, *Gastrostomobdella*, which contained four species from Southeast Asia and Hawaii. Nakano et al. (2012) also erected the monotypic family Orobdellidae for *Orobdella* and moved *Mimobdella* to Salifidae. Later, Nakano et al. (2018) added two more species to *Gastrostomobdella* (*G.extenta* Nakano & Jeratthitikul in Nakano et al., 2018 and *G.ampunganensis* Nakano in Nakano et al., 2018), and a new combination for the Kinabalu Giant Red Leech [*Gastrostomobdellabuettikoferi* (Blanchard, 1897)], bringing the number of species in the genus to seven; this study also added a second genus, *Scaptobdella* Blanchard, 1897, containing four species, thus giving the family a distribution ranging from Sundaland to Indochina.

**Environment and habitat.** Terrestrial.

#### ﻿Glossiphoniidae (Vaillant, 1890. Revised) [leech]

Fig. 40

**Common name.** None.

**LSID.** Urn:lsid:marinespecies.org:taxname:160008.

**Diagnosis (Level 3).** Body regionalization absent; body dorsoventrally flattened (Fig. 40A); protrusible proboscis present; foregut without a distinct ventral or axial pharynx (Fig. 40C); gonadal segments lacking a copulatory area for sperm transfer; branchiae absent.

**Description.** See Suppl. material 1.

**Remarks.** ﻿Glossiphoniidae is a widespread family of jawless leeches (Rhynchobdellida) occurring in fresh waters. It comprises 15 genera and 76 species (WoRMS 2025). ﻿Glossiphoniidae is the only leech family to show parental care: adults keep their offspring attached to the ventral body surface or, in rare cases, inside a brood pouch. Some genera are restricted to the Holarctic, for example, *Glossiphonia* Johnson, 1816 and *Placobdella* Blanchard, 1893 with the majority of species in the Nearctic, and *Torix* Blanchard, 1893, which is limited to the eastern Palearctic; *Placobdella* species are known for parasitizing birds, mammals, amphibians, and reptiles. The glossiphoniid genus *Haementeria* de Filippi, 1849 occurs in the Neotropical region and *Helobdella* Blanchard, 1896 has speciated (over 35 species known) in colder (elevated) regions of South America (Sket and Trontelj 2008); the few species of *Helobdella* in Australia may all be introductions, including the South American native *Helobdellaeuropaea* Kutschera, 1987, which has also been introduced to Europe, USA, Taiwan, and North Africa (Rashni et al. 2023). Other genera such as *Theromyzon* Philippi, 1867 have species distributed across all biogeographical regions except for the Australasian region (Sket and Trontelj 2008). Some species have been spread by humans.

**Environment and habitat.** Aquatic; freshwater; epizoic or endozoic (*Hemiclepsis* occurs in the mantle cavity of freshwater mollusks).

#### ﻿Glossoscolecidae Michaelsen, 1900 [megadrile]

Fig. 41

**Common name.** None.

**LSID.** Urn:lsid:marinespecies.org:taxname:994665.

**Figures 41, 42.**
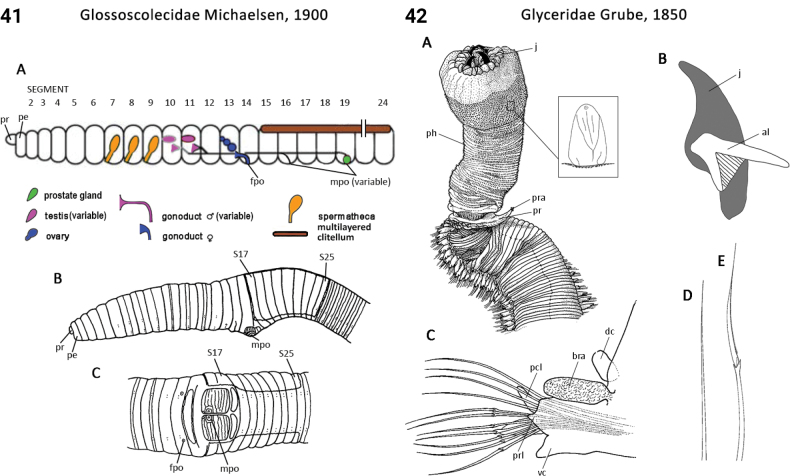
Distinguishing features: **41.**Glossoscolecidae: **A.** Schematic image of reproductive organs, dorsal side up; **B, C.**Glossoscolex (Praedrilus) lutocolus: **B.** Lateral view; **C.** Ventral view. Abbreviations: fpo female pore mpo male pore pe peristomium pr prostomium S segment. Sources: **A** derivative of fig. 8.4 B Jamieson (2006), **B, C** derivatives of fig. 1a, b Bartz et al. (2012). **42.**Glyceridae: **A.** Anterior end of *Glycera* species with pharynx everted; inset showing papilla; **B.** Jaw apparatus; **C.** Mid-body parapodium of *Glycera* species; **D, E.** Chaetae of *Glycera* species: **D.** Simple notochaeta; **E.** Compound neurochaeta. Abbreviations: al aileron bra branchia dc dorsal cerrus j jaw pa palp ph pharynx pcl postchaetal lobe pr prostomium pra prostomial appendages prl prechaetal lobe vc ventral cirrus. Sources: **A, C–E** derivative of fig. 1.73 Beesley et al. (2000), **B** derivatives of fig. 2 Böggemann (2022).

**Diagnosis (Level 1).** Secondary annulation present; gut straight with side branches; clitellum partially encircles body, situated in region of male pores and posterior to female pores (Fig. 41A–C); nephridia, one pair in each segment (holonephridia); calciferous glands present.

**Description.** See Suppl. material 1.

**Remarks.**Glossoscolecidae is similar to Tritogeniidae and only distinguishable from it at DELTA Diagnostic Level 1. Glossoscolecidae is native to the Neotropical Realm but has been introduced to other continents and even to oceanic islands. It comprises six genera (*Enantiodrilus* Cognetti, 1902, *Fimoscolex* Michaelsen, 1900, *Glossodrilus* Cognetti de Martiis, 1905, *Glossoscolex* Leuckarton Froriep, 1835, *Holoscolex* Cognetti de Martiis, 1904, *Righiodrilus* Benham, 1890) and 156 species (Misirlioğlu et al. 2023). Brinkhurst and Jamieson (1971) provide keys to genera and species at the time. James (2000) provides a key to species of the Samoan Archipelago. Plisko and Nxele (2015) provide a key to distinguish foreign Glossoscolecidae taxa from native ones of South Africa. Dos Santos et al. (2017) provide a key for all species of *Righiodrilus*.

**Environment and habitat.** Terrestrial.

#### ﻿Glyceridae Grube, 1850 [polychaete]

Fig. 42

**Common name.** Bloodworms.

**LSID.** Urn:lsid:marinespecies.org:taxname:952.

**Diagnosis (Level 1).** Body with secondary annulation; pharynx jaws present, two pairs (Fig. 42A, B).

**Description.** See Suppl. material 1.

**Remarks.**Glyceridae is very similar to Goniadidae and only distinguishable at DELTA Diagnostic Level 1. Glyceridae includes four genera and 82 species (WoRMS 2025), and have a global distribution. Böggemann (2002, 2023) revised the family and provides DELTA interactive keys to world genera and species. Rizzo et al. (2007) provide a key to species of *Glycera* Lamarck, 1818 from southeastern-southern Brazil. Gil (2011) provides key to European taxa. Böggemann and Fiege (2001) list all the valid species of *Glycera* and provide a key to all species of the genus and Böggemann (2015) provides a key to glycerids from Lizard Island, Great Barrier Reef, Australia.

**Environment and habitat.** Aquatic, marine; coastal, continental shelf or deep sea; soft or hard substrata.

#### ﻿Goniadidae Kinberg, 1866 [polychaete]

Fig. 43

**Common name.** None.

**LSID.** Urn:lsid:marinespecies.org:taxname:953.

**Figures 43, 44.**
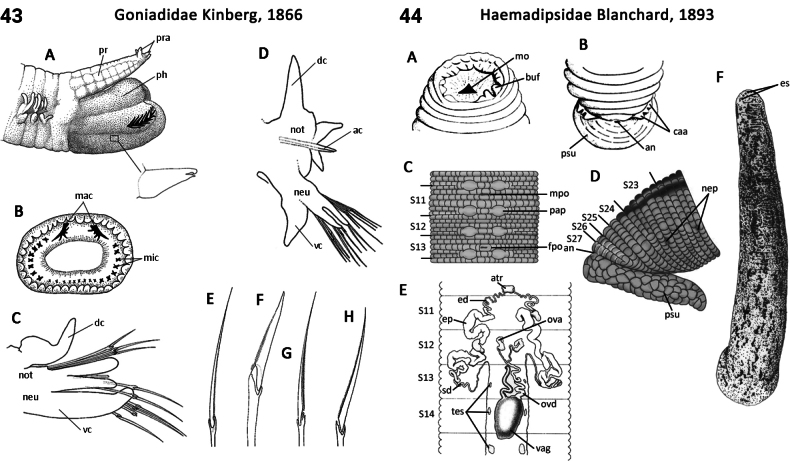
Distinguishing features: **43.**Goniadidae: **A.** Anterior end of *Goniada* species with pharynx everted partially; inset showing papilla; **B.** Details of pharynx of *Glycindearmigera* showing the micrognaths and macrognaths, terminal anterior view; **C.** Anterior parapodium of *Goniada* species; **D.** Posterior parapodium of *Goniada* species; **E–H.** Compound neurochaetae of *Goniada* species from chaetiger 23 (**E**) chaetiger 21 (**F**) chaetiger 27 (**G**) chaetiger 40 (**H**). Abbreviations: ac acicular spine dc dorsal cirrus mac macrognath mic micrognath neu neuropodium not notopodium ph pharynx pr prostomium pra prostomial appendage vc ventral cirrus. Sources: **A–I** derivatives of fig. 1.74 Beesley et al. (2000). **44.**Haemadipsidae: **A, B.***Haemadipsa* sp.: **A.** Anterior end ventral view; **B.** Posterior end dorsal view; **C, D.***Idiobdellaseychellensis*: **C.** Ventral view showing position of gonopores and medioventral papillae; **D.** Lateral view of tail end, lacking caudal auricles; **E.** Reproductive organs of *Idiobdellaseychellensis*; **F.***Haemadipsarjukjuana*, dorsal view. Abbreviations: an anus atr atrium buf buccal frill caa caudal auricle ed ejaculatory duct ep epididymus es eye spot fpo female pore mo mouth mpo male pore nep nephridiopore ovi ovary ovd oviduct pap papilla psu posterior sucker S segment sd sperm duct tes testis vag vagina. Sources: **A, B** derivatives of fig. 21 Mann (1962), **C, D** derivatives of fig. 2 Borda and Siddall (2010), **E** derivative of fig. 6 Harding (1913), **F** derivative of fig. 1 Lai et al. (2011).

**Diagnosis (Level 1).** Body with secondary annulation; pharynx bearing multiple jaw elements of different shapes and sizes (Fig. 43A, B; mac, mic).

**Description.** See Suppl. material 1.

**Remarks.**Goniadidae is very similar to Glyceridae and only distinguishable at DELTA Diagnostic Level 1. Goniadidae includes eight genera and 90 species (WoRMS 2025), and has a global distribution. Böggemann (2005, 2023) revised the family and provides keys to world genera and species. Gil (2011) provides key to European taxa and Böggemann (2015) provides a key to goniadids from Lizard Island, Great Barrier Reef, Australia.

**Environment and habitat.** Aquatic, marine; coastal, continental shelf or deep sea; soft substrata.

#### ﻿Haemadipsidae Blanchard, 1893 [leech]

Fig. 44

**Common name.** None.

**LSID.** Urn:lsid:marinespecies.org:taxname:765258.

**Diagnosis (Level 3).** Body dorsoventrally flattened, epidermis tessellated (Fig. 44C, D, F) ; jaw apparatus includes pseudognaths, cutting plates and stylets, one row of teeth; pharyngeal ridges not rotated (euthylaematous); caeca present in midgut (= posterior crop caeca); posterior sucker present, elliptical in shape, rays present (Fig. 44B, psu); egg sacs globular; single shared oviduct from egg sac (Fig. 44E); vaginal sac present (Fig. 44E); nephridial pores single (ventromedial).

**Description.** See Suppl. material 1.

**Remarks.**Haemadipsidae belongs to the jawed Hirudiniformes (Arhynchobdella) which includes both blood-feeding and invertebrate predatory leeches. The family includes 19 species in five genera (WoRMS 2025), which are distributed throughout the Indo-Pacific, including Asia (*Haemadipsa* Tennent, 1859, *Tritetrabdella* Moore, 1938), Madagascar and Seychelles (*Chtonobdella* Grube, 1866), Australia and Papua New Guinea (*Philaemon* Lambert, 1898, *Chtonobdella*), and the Pacific Islands (*Chtonobdella*, *Nesophilaemon* Nybelin, 1942) (Soos 1967; GBIF Secretariat 2025). Borda and Siddall (2010) recognised three subfamilies: Haemadipsinae, for trignathous human blood-feeding *Haemadipsa* species, Domanibdellinae, for the duognathous species, and Tritetrabdellinae for *Haemadipsacavatuses* + *Tritetrabdella*. As such we do not recognize the family Domanibdellidae Richardson, 1975 in this dataset. Soos (1967) provides a key to Haemadipsidae genera of the world, with a catalogue of the species. Nesemann and Sharma (2001) provide a well-illustrated account of three species from Nepal and Bihar, India.

**Environment and habitat.** Terrestrial (mainly); moist terrestrial, subterranean or hyporheic (rarely); epizoic.

#### ﻿Haplotaxidae Michaelsen, 1900, sensu stricto [microdrile]

Fig. 45

**Common name.** None.

**LSID.** Urn:lsid:marinespecies.org:taxname:1039997.

**Figures 45, 46.**
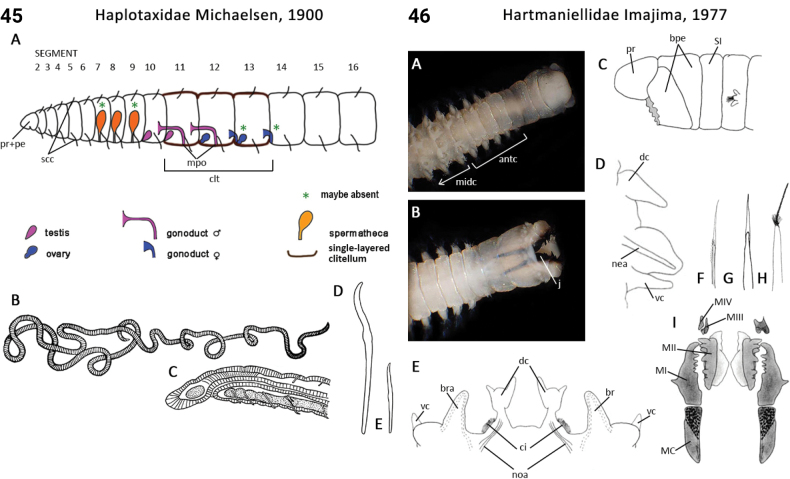
Distinguishing features: **45.**Haplotaxidae: **A.** Schematic diagram showing location of reproductive organs, dorsal side up; **B–E.***Haplotaxisgordioides*: **B.** General view; **C.** Anterior end; **D.** Ventral chaeta; **E.** Dorsal chaeta. Abbreviations: clt clitellum, mpo male pore pe peristomium pr prostomium scc simple crochet chaeta. Sources: **A–C** derivatives of fig. 12.24, 12.3 Thorp et al. (2019), **D, E** derivatives of Timm (2009), fig. 163. **46.**Hartmaniellidae: **A–I.***Hartmaniella* sp.: **A.** Anterior end in ventral view; **B.** Anterior end in dorsal view; **C.** Anterior end in lateral view; **D.** Parapodium from anterior chaetiger; **E.** Parapodium from mid-body chaetiger; **F.** Compound spiniger; **G.** Acicular spine; **H.** Acicula; **I.** Jaw apparatus (schematic). Abbreviations: antc anterior chaetigers bpe biannulate peristomium bra branchia ci cilia dc dorsal cirrus j jaw MI maxilla 1 MII maxilla 2 MIII maxilla 3 MIV maxilla 4 MC maxillary carrier midc midbody chaetigers nea neuroacicula noa notoacicula pr prostomium SI segment 1 vc ventral cirrus. Sources: **A, B** photo Chris Glasby, **C–I** after fig. 2 Carrera Parra (2003).

**Diagnosis (Level 3).** Body elongate; peristomium long, usually bi-annulate; chaetae arranged in widely spaced lateral and ventrolateral pairs (lumbricine arrangement), with two pairs of chaetae (crotchets) or only a single chaeta per bundle (Fig. 45A, C); testes, two pairs in total (Fig. 45A); male pores in segment following testicular segment (plesioporous); nephridial pores located anteriorly and gonoducts located around clitellum, or nephridial pores located posterior to gonoducts; spermathecae pre-testicular, spermathecal pores located well anterior to male and female (gonadal) pores (Fig. 45A); prostate gland absent.

**Description.** See Suppl. material 1.

**Remarks.** Until recently, Haplotaxidae was thought to have a cosmopolitan distribution, typically aquatic or limnic (including groundwater), with 20 species and eight genera (Martin et al. 2008). However, both morphological and molecular studies (Jamieson 2006; Anderson et al. 2017; Erséus et al. 2020) indicated that the family was not monophyletic and needed to be split into several families. Martin et al. (2024) then restricted the concept of the family to include only aquatic forms belonging to *Haplotaxis* (which forms the basis of our family coding) and established four new families – Pelodrilidae (Europe and the Australian region; seven genera, 16 species), Haplotaxoididae (North America; one genus, two species) and the monospecific Limpluvidae and Ohtakianidae, both only known from Japan – and a redefined Haplotaxidae sensu stricto. Despite this more restricted single-genus concept comprising eight species (WoRMS 2025), the family is still distributed across most of the world’s majorrealms. The aquatic *Haplotaxisgordioides* (Hartman, 1821) was once thought to be widely distributed but has been shown recently to consist of a diverse complex of species (Martin et al. 2023a). Brinkhurst and Jamieson (1971) provide keys to genera and species at the time. Pinder (in press) provides a key to Australian species.

**Environment and habitat.** Aquatic (including freshwater, subterranean, and hyporheic); soft substrata (usually coarse sand).

#### ﻿Hartmaniellidae Imajima, 1977 [polychaete]

Fig. 46

**Common name.** None.

**LSID.** Urn:lsid:marinespecies.org:taxname:249686.

**Diagnosis (Level 3).** Body regionalization present; pharynx jaws present (Fig. 46B, I); jaws with maxillary carriers similar in length to combined length of maxillae (Fig. 46I); prominent parapodial lobes with interramal fleshy process present (Fig. 46E); compound chaetae present (Fig. 46F, G).

**Description.** See Suppl. material 1.

**Remarks.**Hartmaniellidae is represented by a single genus, *Hartmaniella* Imajima, 1977, and three species (WoRMS 2025). *Hartmaniella* has few distribution records in GBIF.org (2023), but this may simply reflect an unfamiliarity with the taxon.

**Environment and habitat.** Aquatic, marine; continental shelf; soft substrata.

#### ﻿Hesionidae Grube, 1850 [polychaete]

Fig. 47

**Common name.** None.

**LSID.** Urn:lsid:marinespecies.org:taxname:946.

**Figures 47, 48.**
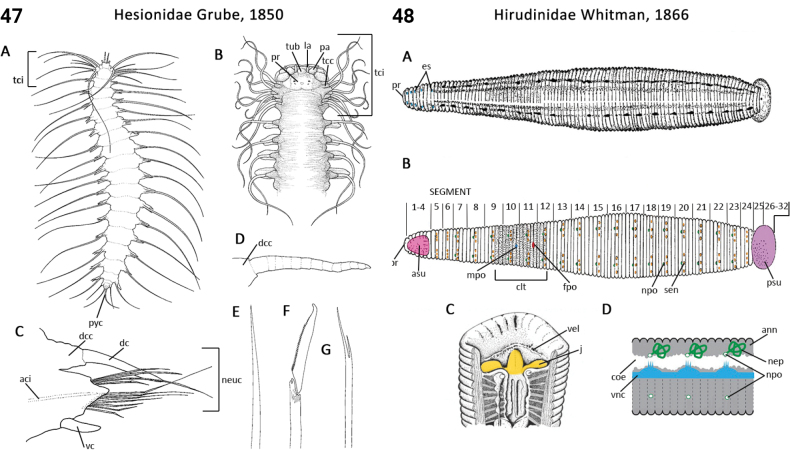
Distinguishing features: **47.**Hesionidae: **A.** Hesionid sp. entire animal, dorsal view; **B.** Anterior end *Leocrates* sp. dorsal view; **C.** Median parapodium *Nereimyra* sp.; **D–F.** Cirrus and chaetae *Nereimyra* sp.: **D.** Details of dorsal cirrus; **E.** Simple chaeta; **F.** Compound chaeta; **G.** Forked chaeta *Gyptis* sp. from chaetiger 14. Abbreviations: aci acicula dc dorsal cirrus dcc dorsal cirri cirrophore la lateral antenna neuc neurochaetae pa palp pr prostomium pyc pygidial cirri tcc tentacular cirri cirrophore tci tentacular cirri tub tubercle vc ventral cirrus. Sources: **A–G** derivatives of fig. 1.75 Beesley et al. (2000). **48.**Hirudinidae: **A–C.***Hirudomedicinalis*: **A.** Dorsal view of the medicinal leech; **B.** Ventral view; **C.** Dissected anterior end to show jaws; **D.** General organisation of body segments in leeches. Abbreviations: ann annulus asu anterior sucker coe coelom clt clitellum es eye spot fpo female pore j jaw (coloured) mpo male pore nep nephridium npo nephridial pore pr prostomium psu posterior sucker sen sensillium vel vellum vnc ventral nerve cord. Sources: **A–C** derivatives of Mann (1962), **D** derivative of fig. 2, Kuo and Lai (2019).

**Diagnosis (Level 3).** Prostomium rounded to oval bearing antennae (Fig. 47B, la); first segment tentaculate, 6–8 pairs tentacular cirri, some with internal aciculae (Fig. 47A, B; tci); second segment achaetous; chaetae first appear 3–5 segments after peristomium (Fig. 47B); first chaetiger with neurochaetae only; pygidium as a simple lobe (often bearing cirri) (Fig. 47A; pyc).

**Description.** See Suppl. material 1.

**Remarks.**Hesionidae has a complicated taxonomy and its concept has changed significantly during the last decades. The interstitial genera *Microphthalmus* Mecznikow, 1865 and *Hesionides* Friedrich, 1937 were removed by Pleijel and Dahlgren (1998), and that change was reflected in POLiKEY (Glasby and Fauchald 2003); in 2019 those two genera were finally allocated a family, Microphthalmidae (Salazar-Vallejo et al. 2019a). Hesionidae now includes 35 genera and 224 species (WoRMS 2025), and has a global distribution. Approximately 30 species are considered obligate or facultative invertebrate symbionts (Martin and Britayev 1998, 2018). Rizzo and Salazar-Vallejo (2014) provide a key to all genera, Gil (2011) provides key to European taxa, which includes 15 genera including *Microphthalmus* and *Hesionides*, Salazar-Vallejo (2022) provides keys to world species of *Psamathe* Johnston, 1836 and Pamungkas (2024) provides a key to hesionids of Indonesia.

**Environment and habitat.** Aquatic, marine; coastal, continental shelf, or deep sea; soft or hard substrata, hydrothermal vents, and cold seeps, epizoic, or sunken bones of vertebrates.

#### ﻿Hirudinidae Whitman, 1886 [leech]

Fig. 48

**Common name.** Medicinal leeches.

**LSID.** Urn:lsid:marinespecies.org:taxname:160020.

**Diagnosis (Level 0).** Anterior end sucker present (Fig. 48B); jaws include pseudognaths, cutting plates and stylets (Fig. 48C), one row of teeth present; posterior sucker lacking rays (Fig. 48A, B); egg sacs globular.

**Description.** See Suppl. material 1.

**Remarks.**Hirudinidae belongs to the jawed Hirudiniformes (Arhynchobdella) which includes both blood-feeding and invertebrate predatory leeches. It comprises 13 genera and 31 species (WoRMS 2025) and is widely distributed, occurring on all continents except Antarctic (Sket and Trontelj 2008). We follow the current broad concept of the family (e.g., Sawyer 1986; Borda et al. 2008; WoRMS 2025) in most aspects, even though the family is likely to be non-monophyletic (Phillips and Siddall 2009). Using our dataset, Hirudinidae is not diagnosable (DELTA Diagnostic Level 0), as it is not separable from Praobdellidae and especially Semiscolecidae. Thus, the diagnosis above is not sufficient for confirming family identification (refer to full description); we suggest that the family is in need of future phylogenetic revision. Hirudinidae comprises four to six subfamilies depending on author: Ornithobdellinae, Macrobdellinae, Hirudinariinae, Richardsonianinae, Hirudininae, and Praobdellinae; we treat Praobdellinae as a family in this dataset following Phillips et al. (2010). Ornithobdellinae contains terrestrial species that feed on seabirds in the subantarctic islands of Australia and New Zealand; Richardsonianinae (*Bassianobdella* Richardson, 1970, *Goddardobdella* Richardson, 1969, *Richardsonianus* Soos, 1968) is endemic to Australia (Sket and Trontelj 2008); Macrobdellinae Richardson, 1969 encompasses the blood-feeding genera *Macrobdella* Verrill, 1872, and *Oxyptychus* Grube, 1850, as recognised in WoRMS (2025); and Hirudininae comprises several genera in Eurasia and Africa, including *Haemopsis* Savigny, 1822 (i.e., the type genus of the monotypic family Haemopidae), which is also not recognised in this dataset. Nesemann and Sharma (2001) provide a well-illustrated account of the group from Nepal and Bihar, India.

**Environment and habitat.** Terrestrial or aquatic; moist terrestrial, freshwater; epizoic.

#### ﻿Histriobdellidae Claus & Moquin-Tandon, 1884 [polychaete]

Fig. 49

**Common name.** None.

**LSID.** Urn:lsid:marinespecies.org:taxname:972.

**Figures 49, 50.**
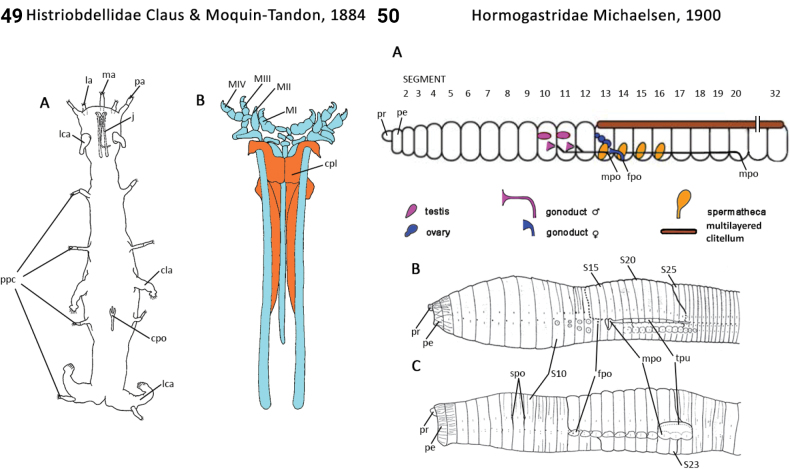
Distinguishing features: **49.**Histriobdellidae: *Stratiodrilustasmanicus*. **A.** Entire animal ventral view; **B.** Prionagnath-type jaws with maxillae everted ventral view. Maxillae (blue) showing long carrier and multiple jaw elements; mandibles (orange) with long shaft and unfused cutting plate. Abbreviations: cla clasper cpl cutting plate cpo copulatory organ j jaw la lateral antenna lca locomotory appendage ma median antenna MI maxilla 1 MII maxilla 2 MIII maxilla 3 MIV maxilla 4 pa palp ppc parapodial cirrus. Sources: **A, B** after fig. 1.65 Beesley et al. (2000). **50.**Hormogastridae: **A.** Schematic image of reproductive organs, dorsal side up; **B.***Hormogasterrediiinsularis*, anterior end showing genital fields; **C.***Ailoscolexlacteospumosus*, anterior end showing genital fields. Abbreviations: fpo female pore mpo male pore pe peristomium pr prostomium S segment spo spermathecal pore tpu tubercular pubertatis. Sources: **A, B, C** derivatives of figs 8.4B, 8.39B, 8.39A Jamieson (2006).

**Diagnosis (Level 3).** Prostomial antennae present (Fig. 49A, me, la); second segment chaetous; pygidium deeply cleft forming two large ‘feet’ (Fig. 49A).

**Description.** See Suppl. material 1.

**Remarks.**Histriobdellidae includes three genera and 13 species (Helm et al. 2021; WoRMS 2025), which occur in coastal areas (marine and, mainly, freshwater) in the Americas and Australia. As is typical of symbiotic annelids, its precise relationship within Annelida has been difficult to determine (using morphology or DNA), although the form of the jaws allies them with Eunicida, in particular Dorvilleidae.

**Environment and habitat.** Aquatic, marine; brackish or freshwater; coastal; epizoic.

#### ﻿Hormogastridae Michaelsen, 1900 [megadrile]

Fig. 50

**Common name.** None.

**LSID.** Urn:lsid:marinespecies.org:taxname:1060952.

**Diagnosis (Level 1).** Dorsal pores on midline present; gizzard present; clitellum situated in region of male and female pores (Fig. 49A–C); tubercula pubertatis form paired ridges ventral to clitellum; sperm sac absent; prostate gland present.

**Remarks.**Hormogastridae includes nine genera (*Ailoscolex* Bouché, 1969, *Boucheona* Marchán et al. 2018, *Carpetania* Marchán et al. 2018, *Diazcosinia* Marchán et al. 2018, *Hemigastrodrilus* Bouché, 1970, *Hormogaster* Rosa, 1887, *Norana* Marchán et al. 2018, *Vignysa* Bouché, 1970, *Xana* Díaz Cosín, Briones & Trigo, 1989) and 37 species (Marchán et al. 2018a, b). The family is similar to Tritogeniidae and only distinguishable from it at DELTA Diagnostic Level 1. Unlike the southern African Tritogeniidae, Hormogastridae is restricted to the Palaearctic Realm. Molecular evidence places *Ailoscolexlacteospumosus* Bouché, 1969 within Hormogastridae (James and Davidson 2012); therefore, Ailoscolecidae is a probable synonym of Hormogastridae, and we treat it as such here. Hormogastridae may also include the monogeneric Komarekionidae (only species, *Komarekionaeatoni* Gates, from the eastern USA) as Blakemore (2006) considered this family to be a junior synonym of Ailoscolecidae, citing the morphological reasons given in Sims (1980); however, we side with the more recent molecular data that strongly indicate a Komarekionidae – Sparganophilidae relationship (see comments under Sparganophilidae). Gates and Reynolds (2017) include *Komarekionaeatoni* in their key.

**Environment and habitat.** Terrestrial, soil; soft substrata.

#### ﻿Hrabeiellidae Christoffersen, 2012 [microdrile]

Fig. 51

**Common name.** None.

**Figures 51, 52.**
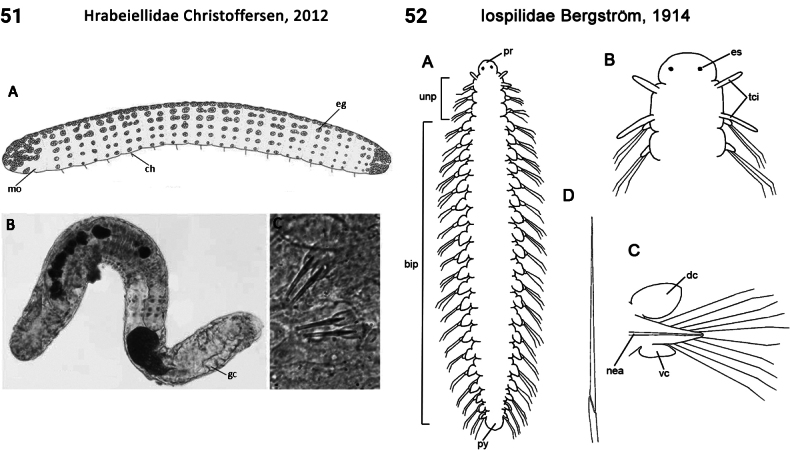
Distinguishing features: **51.**Hrabeiellidae: **A–C.***Hrabeiellaperiglandulata*: **A.** Whole specimen showing characteristic arrangement of epidermal glands; **B.** Living adult animal; **C.** Typical arrangement of chaetae segment. Abbreviations: ch chaeta eg epidermal gland gc gland cell mo mouth. Sources: **A–C** derivative of fig. 7.5.1.1 Purschke (2019a). **52.**Iospilidae: **A–C.***Iospilusphalacroides*: **A.** Entire animal dorsal view; **B.** Head dorsal view; **C.** Uniramous parapodium of anterior body; **D.** Compound chaeta *Phalacrophorusuniformis*. Abbreviations: bip biramous parapoda dc dorsal cirrus es eye spot nea neuroacicula pr prostomium py pygidium tci tentacular cirri unp uniramous parapodia. Sources: **A–D** derivatives of fig. 1.76, Beesley et al. (2000).

**Diagnosis (Level 3).** Body more-or-less cylindrical; segment number fixed at 16 segments including 1 preoral ‘segment’ (peristomium) and 15 postoral segments (Fig. 51A, B, eg); clitellum absent; nuchal organs present; chaetae present, distally bristled and shovel-shaped, two or three per bundle (exceptionally four).

**Description.** See Suppl. material 1.

**Remarks.**Hrabeiellidae are simple-bodied terrestrial annelids assigned to Oligochaeta by Rouse et al. (2022). They are included among microdriles here based on overall appearance following recent phylogenetic investigations including morphological and molecular evidence presented by Zrzavý et al. (2009), who identified them as the sister group of 'true' clitellates. However, their phylogenetic position is far from clear, particularly because they have nuchal organs (a typical polychaete trait), lack a clitellum (a feature of Clitellata), and have sperm morphology that resembles neither polychaetes nor clitellates (Rota and Lupetti 2015). Purschke (2019a) provides a recent comprehensive morphological treatment of the family from a polychaete perspective. The family is represented by a single species in the genus *Hrabeiella*, *H.periglandulata* Pižl & Chalupský, 1984, which has a Palearctic distribution.

**Environment and habitat.** Terrestrial, soil; soft substrata.

#### ﻿Iospilidae Bergström, 1914 [polychaete]

Fig. 52

**Common name.** None.

**LSID.** Urn:lsid:marinespecies.org:taxname:155189.

**Diagnosis (Level 3).** Body shape more-or-less cylindrical; in life, body translucent, gut visible; discrete lobe-like head without appendages (antennae); eyes present (Fig. 52 A, B); palps present; peristomium not visible; tentacular cirri present (Fig. 52B); notopodial lobes absent (Fig. 52C); pygidial appendages absent (Fig. 52A).

**Description.** See Suppl. material 1.

**Remarks.**Iospilidae is maintained as a family contra Rouse et al. (2022) who considered the holopelagic taxon to be a junior synonym of Phyllodocidae based on molecular evidence; morphological data supporting this conclusion are not yet available. The family has been maintained as such in WoRMS, where it is currently listed to have three genera and five species (WoRMS 2025); it was also treated as a valid family in POLiKEY (Glasby and Fauchald 2003). Iospilids can be distinguished from phyllodocids, including Alciopini, by the lack of lateral antennae. They are globally distributed. Dales (1957) provides keys to genera and species of Iospilidae of the Pacific Ocean; O’Sullivan (1982) provides a key to Southern Ocean taxa.

**Environment and habitat.** Aquatic, marine; coastal, continental shelf or deep sea; holopelagic.

#### ﻿Iphionidae Kinberg, 1856 [polychaete]

Fig. 53

**Common name.** None.

**LSID.** Urn:lsid:marinespecies.org:taxname:155222.

**Figures 53, 54.**
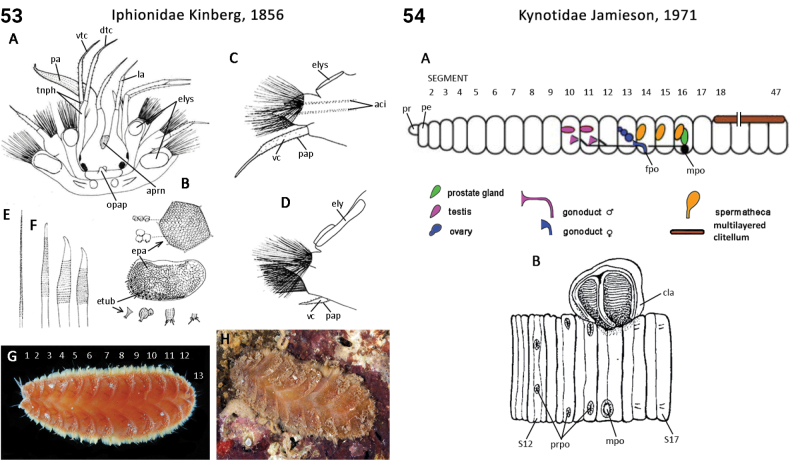
Distinguishing features: **53.**Iphionidae: **A.***Iphionetreadwelli* anterior end dorsal view (right palp regenerating); **B–F.***Iphionemuricata*: **B.** Left middle elytron, with detail of polygonal area and spinous tubercles; **C.** Right parapodium from segment 2, anterior view; **E.** Neurochaeta from segment 2; **D.** Right middle elytrigerous parapodium, anterior view; **F.** Upper, middle, and lower neurochaeta; **G, H.** Entire animal (elytra pairs numbered). Abbreviations: aci acicula aprn anterior prostomial notch dtc dorsal tentacular cirrus ely elytron elys elytral scar epa elytra polygonal area etub elytra tubercle la lateral antenna opap occipital papilla pa palp pap papilla tnph tentaculophore vc ventral cirrus vtc ventral tentacular cirri. Sources: **A–F** derivative of figs 1, 2, 7 Pettibone (1986), **G, H** photo: Arthur Anker. **54.**Kynotidae: **A.** Schematic image of reproductive organs, dorsal side up; **B.***Kynotuscingulatus* ventral surface of segment 13–16, showing the pores of three pairs of prostates; a fourth pair discharges at the male pores; clasper is shown evaginated through the left male pore (clasper pore). Abbreviations: cla clasper fpo female pore mpo male pore pe peristomium pr prostomium prpo prostate pore S segment. Sources: **A, B** derivatives of fig. 8.4 B, 8.37 B, Jamieson (2006).

**Diagnosis (Level 1).** Body shape ovate to elliptical (Fig. 53G, H); prostomium anteriorly incised (Fig. 53A); elytra with a tuberculated pentagonal or hexagonal pattern (Fig. 53B).

**Description.** See Suppl. material 1.

**Remarks.**Iphionidae were, until relatively recently, treated as a subfamily of Polynoidae (e.g., Pettibone, 1986); in 2012 the subfamily was found to fall out as sister group to a clade consisting of Polynoidae and Acoetidae (Norlinder et al. 2012), and later as sister to Acoetidae exclusively (Gonzalez et al. 2018; Zhang et al. 2018). In POLiKEY (Glasby and Fauchald 2003) they were treated as a subfamily of Polynoidae. In the present dataset, they are only distinguishable from Polynoidae at DELTA Diagnostic Level 1. Unlike Polynoidae, the family is restricted to low and mid-latitude oceans and seas (GBIF.org 2023). Iphionidae comprises four genera and 23 species (WoRMS 2025). Piotrowski (2014) provides a key to all *Iphione* Kinberg, 1856 species at the time.

**Environment and habitat.** Aquatic, marine; coastal, continental shelf or deep sea; soft or hard substrata, hydrothermal vents, and cold seeps.

#### ﻿Kynotidae Jamieson, 1971 [megadrile]

Fig. 54

**Common name.** None.

**LSID.** Urn:lsid:marinespecies.org:taxname:1727867.

**Diagnosis (Level 2).** Spermathecae present, post-testicular (Fig. 54A); clitellum present, situated posterior to male and female pores (Fig. 54A); tubercula pubertatis absent; prostate gland present.

**Description.** See Suppl. material 1.

**Remarks.**Kynotidae includes a single genus (*Kynotus*) and 22 species and is endemic to Madagascar (Misirlioğlu et al. 2023; WoRMS 2025). The family cannot be distinguished from Almidae at Diagnostic Level 3. Razafindrakoto et al. (2017) provide an identification key to *Kynotus* Michaelsen, 1891 species.

**Environment and habitat.** Terrestrial or aquatic, freshwater.

#### ﻿Lacydoniidae Bergström, 1914 [polychaete]

Fig. 55

**Common name.** None.

**LSID.** Urn:lsid:marinespecies.org:taxname:954.

**Figures 55, 56.**
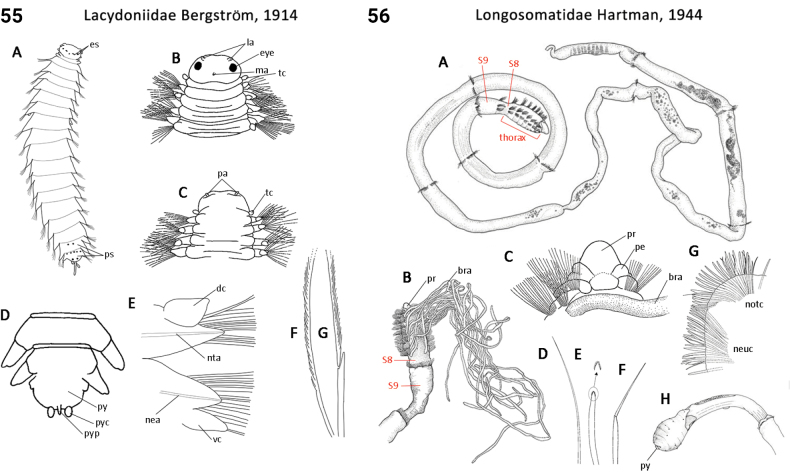
Distinguishing features: **55.**Lacydoniidae: **A.** Entire animal *Lacydoniabrasiliensis*, dorsal view; **B, C.** Anterior end of *Lacydoniaoculata*, dorsal view (**B**), vental view (**C**); **D.** Posterior end of *Lacydoniaoculata*; **E.** Parapodium of *Lacydoniamiranda*; **F.** Notopodial capillary chaeta of *Lacydonialaureci*; **G.** Neuropodial spinigerous chaeta of *Lacydonialaureci*. Abbreviations: dc dorsal cirrus es eye spot eye eye la lateral antenna ma median antenna nea neuroacicula nta notoacicula pa palp pyc pygidial cirrus pyp pygidial papilla ps pigment spot py pygidium vc vental cirrus. Sources: **A** derivative of fig. 1 Rizzo and Magalhães (2022a), **B, C, D** derivatives of fig. 1.77 Beesley et al. (2000), **E, F, G** derivatives of fig. 2, Rizzo and Magalhães (2022a). **56.**Longosomatidae: **A.***Heterospiolongissima* posteriorly incomplete; **B.***Heterospio* sp. anterior end dorsal view; **C.** Detail of head dorsal view, palps missing; **D–F.** Chaetae: **D.** Capillary notochaeta; **E.** Spine of posterior chaetiger with detail of tip; **F.** Aristate notochaeta; **G.***Heterospiomediterranea* parapodium from elongated segment with circle of spines and capillaries; **H.***H.longissima* posterior end lateral view. Abbreviations: bra branchia neuc neurochaetae notc notochaetae pe peristomium pr prostomium py pygidium S8 segment 8 S9 segment 9. Sources: **A, G** derivatives of fig. 1 H derivative of fig. 2 Blake and Maciolek (2019a), **B–F** derivative of fig. 1.105 Beesley et al. (2000).

**Diagnosis (Level 3).** In life, body translucent, gut visible; prostomium antennae present (Fig. 55B, C, la, ma); tentacular cirri present, 1 pair (Fig. 55B, C); second segment chaetous; dorsal cirri present, flattened and foliaceous; notopodial lobes represented by at least one chaetal lobe (Fig. 55E); pygidial appendages present, one pair of pygidial cirri and a single medial papilla (Fig. 55D; pyc, pyp); pharyngeal jaws absent.

**Description.** See Suppl. material 1.

**Remarks.**Lacydoniidae is represented by a single genus, *Lacydonia* Marion, 1874, and 15 species (WoRMS 2025). Rizzo et al. (2015) provide a key to world species and Gil (2011) and Parapar et al. (2012) provide keys to European species. The family probably has a worldwide distribution, although records are scarce for some oceans and seas (GBIF.org 2023).

**Environment and habitat.** Aquatic, marine; coastal, continental shelf or deep sea; soft substrata.

#### ﻿Lobatocerebridae Rieger, 1980 [annelid, possibly a polychaete]

**Common name.** None.

**LSID.** Urn:lsid:marinespecies.org:taxname:869064.

**Diagnosis (Level 3).** Body transparent (gut visible), segmentation absent; body shape elongate, more-or-less equal width along entire length; pygidium absent, anus subterminal; testes present.

**Remarks.**Lobatocerebridae is represented by a single genus, *Lobatocerebrum* Rieger, 1980, and only two species (WoRMS 2025). The family is very un-annelid like, lacking segmentation, chaetae, a clearly defined head, nuchal organs, a pygidium and even a coelom. The phylogenomic analysis of Martín-Durán et al. (2021) suggested affinities with Polychaeta.

**Environment and habitat.** Aquatic, marine; coastal or continental shelf; soft substrata.

#### ﻿Longosomatidae Hartman, 1944 [polychaete]

Fig. 56

**Common name.** None.

**LSID.** Urn:lsid:marinespecies.org:taxname:22608.

**Diagnosis (Level 3).** Body shape elongate, more-or-less equal width along entire length; body regionalization present, comprising three regions, mid-body segments strongly elongated (Fig. 56A, B); prostomium bluntly conical; palps present (though easily dislodged and not shown in Fig. 56); parapodia indistinct (chaetae arising directly from body wall) (Fig. 56G); branchiae present (Fig. 56B, C).

**Description.** See Suppl. material 1.

**Remarks.**Longosomatidae is represented by a single genus, *Heterospio* Ehlers, 1874, and 24 species (WoRMS 2025). Rouse et al. (2022) present molecular data showing the family to be the sister group of Cirratulidae (Acrocirridae + Flabelligeridae), which supported the opinion of Wilson (2000) based on morphology. The family appears to have a wide distribution, although records are sparse in some oceans; they appear to be more common in continental shelf and coastal areas (GBIF.org 2023). Gil (2011) provides a key to European species and Blake and Maciolek (2023) provide species descriptions and a comparative table of characters.

**Environment and habitat.** Aquatic, marine; coastal, continental shelf or deep sea; soft substrata.

#### ﻿Lopadorrhynchidae Claparède, 1870 [polychaete]

Fig. 57

**Common name.** None.

**LSID.** Urn:lsid:marinespecies.org:taxname:933.

**Figures 57, 58.**
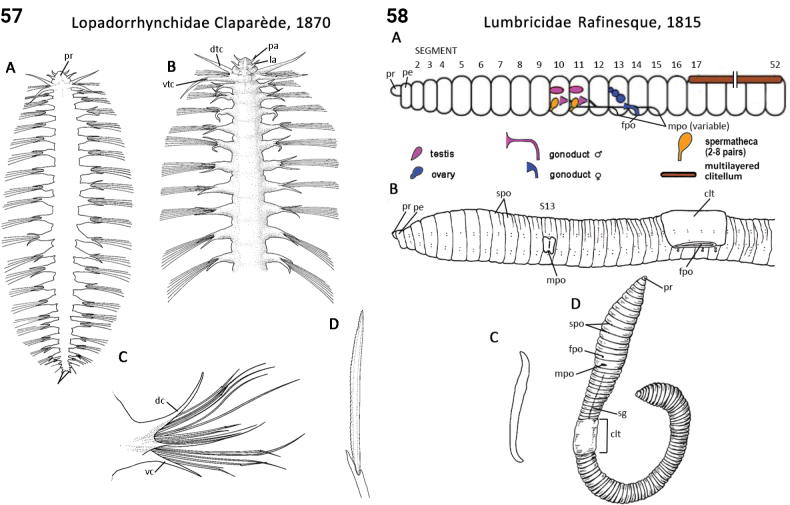
Distinguishing features: **57.**Lopadorrhynchidae: **A–D.***Pelagobiaviguieri*; **A.** Entire animal ventral view; **B.** Anterior end ventral view; **C.** Parapodium from chaetiger 2; **D.** Compound neurochaeta from parapodium of chaetiger 2. Abbreviations: dc dorsal cirrus dtc dorsal tentacular cirrus la lateral antenna pa palp pc pygidial cirrus pr prostomium vc ventral cirrus. Sources: **A–D** derivative of fig. 1.78 Beesley et al. (2000). **58.**Lumbricidae: **A.** Diagram of reproductive organs, dorsal side up; **B.***Eiseniellatetraedra*, anterior body lateral view; **C.** Chaeta; **D.***Lumbricusterrestris* external features of worm showing genital pores, seminal groove and clitellum. Abbreviations: clt multilayered clitellum fpo female pore mpo male pore pe peristomium pr prostomium S segment sg seminal grove spo spermathecal pore. Sources: **A, D** derivatives of fig. 8.4 B, 8.53 A Jamieson (2006), **B** derivative of fig. 30, Martin and Aït Boughrous (2012), **C** derivatives of Timm (2009: 189).

**Diagnosis (Level 3).** Body elongate, more-or-less equal width along entire length, dorsoventrally flattened; in life, translucent, gut visible (Fig. 57A); prostomial antennae present, paired, lateral (Fig. 57B); paired nuchal organs present, may be barely visible or obvious posterolateral ciliated bulbs; notopodial lobes absent, dorsal cirri flattened and foliaceous (slender) (Fig. 57C).

**Description.** See Suppl. material 1.

**Remarks.**Lopadorrhynchidae is a holopelagic family comprising seven genera and 24 species with a global distribution (WoRMS 2025). Pleijel and Dales (1991) provide a key to species from northern Europe while Kolbasova et al. (2020) provide a key to genera. Dales (1957) provides keys to genera and species of Lopadorrhynchidae of the Pacific Ocean; O’Sullivan (1982) provides a key to Southern Ocean taxa.

**Environment and habitat.** Aquatic, marine; coastal, continental shelf or deep sea; holopelagic.

#### ﻿Lumbricidae Rafinesque-Schmaltz, 1815 [megadrile]

Fig. 58

**Common name.** Variety of species-specific common names.

**LSID.** Urn:lsid:marinespecies.org:taxname:154884.

**Diagnosis (Level 1).** Calciferous glands present; clitellum situated posterior to male pore (Fig. 58A–C); spermathecae present; two pores, pre-testicular or in testicular segments (Fig. 58A, B, D).

**Description.** See Suppl. material 1.

**Remarks.**Lumbricidae comprises ~ 615 species and 47 genera (Misirlioğlu et al. 2023). We include the genus *Diporodrilus* Bouché, 1970 within Lumbricidae – it comprises five species/subspecies endemic to the Mediterranean islands of Corsica and Sardinia (Misirlioğlu et al. 2023). Although the genus has been considered recently as a separate monogeneric family, Diporodrilidae (e.g., Marchán et al. 2022, 2024), the morphological differences between it and Lumbricidae (two rows of lateral coelomic pores vs. one row of dorsal pores, nephridial vesicles which open to the exterior both through nephropores and shared collector channels vs through nephropores alone) were not fully explored in our DELTA dataset. Lumbricidae is also similar to Almidae and only distinguishable from it at DELTA Diagnostic Level 1. *Diporodrilus* was found to be sister taxon of the remaining Lumbricidae (Marchán et al. 2022, 2024). Plisko and Nxele (2015) provide a key to distinguish foreign Lumbricidae taxa from native ones of South Africa. Gates and Reynolds (2017) provide a key to North American members of the family. Finally, Blakemore (1999, 2009) estimated that ~ 33 species are cosmopolitan or widespread, and often invasive, including 24 non-endemic species in Australia.

**Environment and habitat.** Terrestrial (mainly) or aquatic, freshwater; soft substrata.

#### ﻿Lumbriculidae Claus, 1872 [microdrile]

Fig. 59

**Common name.** None.

**LSID.** Urn:lsid:marinespecies.org:taxname:177535.

**Figures 59, 60.**
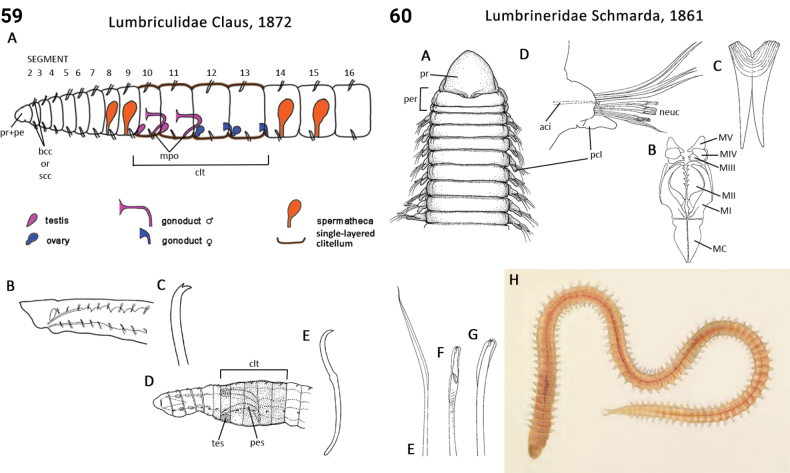
Distinguishing features: **59.**Lumbriculidae: **A.** Diagram showing typical location of reproductive organs (highly variable), dorsal side up; **B, C.***Lumbriculusvariegatus*: **B.** Anterior end; **C.** Bifid crotchet chaeta; **D–F.***Styloscolextubulatus*: **D.** Anterior body of mature specimen with clitellum; **E.** Simple crotchet chaeta. Abbreviations: bcc bifid crotchet chaeta clt clitellum mpo male pore pe peristomium pes penis sheath pr prostomium scc simple crochet chaeta tes testis. Sources: **A, D, E** derivatives of fig. 12.26, 12.3 Thorp et al. (2019), **B, C** derivatives of Timm (2009: 171). **60.**Lumbrineridae: **A–G.**Lumbrineriscf.latreilli; **A.** Anterior end dorsal view; **B, C.** Jaw parts: **B.** Maxillae dorsal view; **C.** Mandibles ventral view; **D.** Parapodium from chaetiger 11; **E–G.** Chaetae: **E.** Simple limbate chaeta from parapodium of chaetiger 11; **F.** Compound hook from parapodium of chaetiger 11; **G.** Simple hook from parapodium of chaetiger 43; **H.***L.latreillii* entire animal. Abbreviations: aci acicula MI maxilla 1 MII maxilla 2 MIII maxilla 3 MIV maxilla 4 MV maxilla 5 MC maxillary carrier neuc neurochaetae pcl postchaetal lobe pr prostomium. Sources: **A–G** derivatives of fig. 1.60 Beesley et al. (2000), H derivative of MacIntosh (1900–1922), pl. LIV, fig. 6.

**Diagnosis (Level 2).** Clitellum situated in region of male pores (Fig. 59A); male pores in same segment as corresponding testes (prosoporous); spermathecal pores paired, 5 pairs (Fig. 59A).

**Description.** See Suppl. material 1.

**Remarks.**Lumbriculidae includes ~ 240 species and subspecies (Fend et al. 2020) and are most diverse in the freshwater of the Holarctic and to a lesser extent, the Nearctic. Lumbriculidae is similar to several families including Dorydrilidae, Haplotaxidae, Naididae, Narapidae, Propappidae and Phreodrilidae, which are unusual among microdriles in often lacking hair chaetae. It cannot be distinguished from Naididae at Diagnostic Level 3. Brinkhurst and Jamieson (1971) provide keys to genera and species at the time. Pinder (2013; in press) provides a key to the only two known Australian species, which are almost certainly introduced. Rodriguez and Fend (2022) provide a key to known species of *Eremidrilus* Fend & Rodriguez, 2003.

**Environment and habitat.** Aquatic, freshwater (rarely brackish water; also including subterranean waters); soft substrata.

#### ﻿Lumbrineridae Schmarda, 1861 [polychaete]

Fig. 60

**Common name.** None.

**LSID.** Urn:lsid:marinespecies.org:taxname:967.

**Diagnosis (Level 3).** Body regionalization absent (Fig. 60H); head, discrete, lobe-like without appendages; prostomium bluntly conical; eyes absent (Fig. 60A); pharynx with jaws present, multiple tooth plates (Fig. 60B); peristomium a double ring (Fig. 60A); first chaetiger with neurochaetae only.

**Description.** See Suppl. material 1.

**Remarks.**Lumbrineridae includes 21 genera and 293 species and has a global distribution (WoRMS 2025). Carrera-Parra (2006) investigated the family phylogeny, created six new genera and provided a key. Gil (2011), D’Alessandro et al. (2016) and Martins et al. (2012) provide keys to European and Mediterranean taxa. Sedick et al. (2023) provide a key to the *Ninoe* from southern Africa, and Martins et al. (2024) provide a taxonomic key to lumbrinerid species from Iberian waters.

**Environment and habitat.** Aquatic, marine; coastal, continental shelf or deep sea; soft or hard substrata, or epizoic (algal holdfasts).

#### ﻿Lutodrilidae McMahan, 1976 [megadrile]

Fig. 61

**Common name.** None.

**LSID.** Urn:lsid:marinespecies.org:taxname:992891.

**Figures 61, 62.**
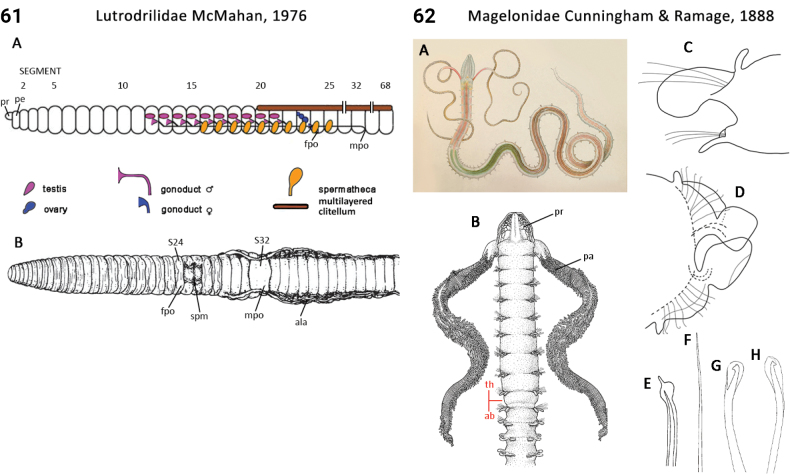
Distinguishing features: **61.**Lutodrilidae: **A.** Diagram of reproductive organs, dorsal side up; **B.***Lutodrilusmultivesiculatus*, anterior end with genital region, in ventral view, showing alae. Abbreviations: ala alae fpo female pore mpo male pore pe peristomium pr prostomium S segment spm spermatophore. Sources: **A, B** derivatives of figs 8.4B, 8.38A Jamieson (2006). **62.**Magelonidae: **A.***Magelonapapillicornis* entire animal; **B.** Anterior end of *Magelonadakini* dorsal view; **C.** Thoracic parapodium of *Magelonasinbadi*; **D.** Abdominal parapodium of *Magelonacrenulifrons*; **E–G.** Notochaetae of *Magelona* sp. **H.** Neurochaeta of *Magelona* sp. Abbreviations: ab abdomen pa palp pr prostomium th thorax. Sources: **A** derivative of McIntosh (1915), pl. XC, fig. 6, B, **E–H** derivatives of fig. 1.106 Beesley et al. (2000), **C, D** derivatives of fig. 4.2.8 Mortimer (2019).

**Diagnosis (Level 3).** Secondary annulation present; gizzard present; clitellum fully encircles body, in region of male pores; spermathecae present (Fig. 61A), spermathecal pores absent, spermatophore present (Fig. 61B, smp); testes, many pairs (Fig. 61A); prostate gland absent.

**Remarks.**Lutodrilidae is a monotypic family represented by *Lutodrilusmultivesiculatus* McMahan, 1976, a distinctive species which is restricted to eastern Louisiana, USA (McMahan 1979; Misirlioğlu et al. 2023).

**Environment and habitat.** Terrestrial or semi-aquatic (freshwater).

#### ﻿Magelonidae Cunningham & Ramage, 1888 [polychaete]

Fig. 62

**Common name.** Shovel-head worms.

**LSID.** Urn:lsid:marinespecies.org:taxname:914.

**Diagnosis (Level 3).** Body (subtly) divided into a thorax and abdomen based on differences in chaetal types (Fig. 62B, th, ab); prostomium flattened, shovel-shaped; palps present (grooved and papillated) (Fig. 62A, B, pa); peristomium not visible.

**Description.** See Suppl. material 1.

**Remarks.**Magelonidae is represented by a single genus, *Magelona* F. Müller, 1858, and 83 species (WoRMS 2025), which follows the recommendation of Mortimer et al. (2021) to place a second genus, *Octomagelona* Aguirrezabalaga, Ceberio & Fiege, 2001 into synonymy with *Magelona*. Meißner et al. (2023), however, subsequently described a new species of *Octomagelona* and proposed that this genus is distinct from *Magelona* and should be reinstated as valid. Fitzhugh et al. (2024) then argued that this action forced *Magelona* into paraphyly and recommended that *Octomagelona* again be synonymized with *Magelona*. *Magelona* has a global distribution although oceanic records are few (GBIF.org 2023), suggesting a preference for coastal and continental shelf sediments. Gil (2011) and Mortimer et al. (2011, 2020) provide keys to European taxa.

**Environment and habitat.** Aquatic, marine; coastal, continental shelf or deep sea; soft substrata.

#### ﻿Maldanidae Malmgren, 1867 [polychaete]

Fig. 63

**Common name.** Bamboo worms.

**LSID.** Urn:lsid:marinespecies.org:taxname:923.

**Figures 63, 64.**
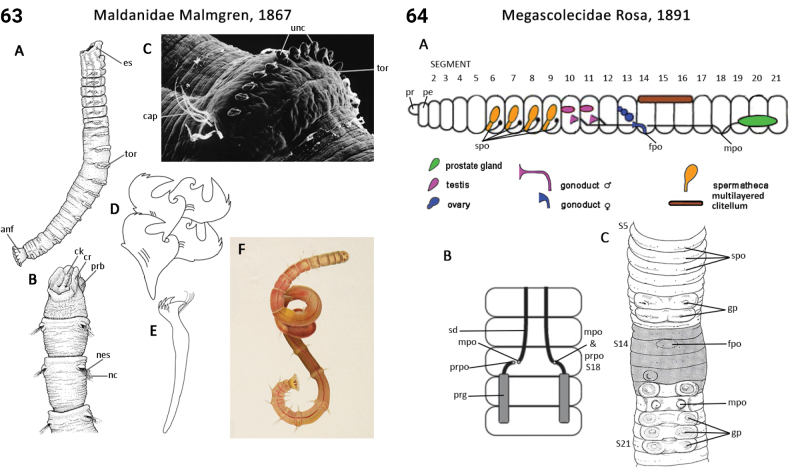
Distinguishing features: **63.**Maldanidae: **A.***Micromaldanepamelae*, entire animal, lateral view; **B.** Anterior end of *Euclymenetrinalis* dorsolateral view; **C.** Mid-body parapodium of *Micromaldanepamelae*; **D.** Double row of uncini of *Rhodineloveni*; **E.** Anterior hook of *Lumbriclymeneinterstricta*; **F.***Nicomachemaculata*, entire animal. Abbreviations: anf anal funnel cap capillary chaetae ck cephalic keel cr cephalic rim es eye spots nc notopodial capillary nes neuropodial spine prb proboscis tor torus unc uncini. Sources: **A–C** derivatives of fig. 1.51 Beesley et al. (2000), **D, E** derivative of figs 4, 5 Assis et al. (2021), **F** derivative of McIntosh (1900–1922), pl. XCII, fig. 5. **64.**Megascolecidae: **A.** Schematic image of reproductive organs, dorsal side up; **B.** Arrangement of pores; **C.***Spenceriellapenolaensis* showing genital markings. Abbreviations: fpo female pore gp genital papilla mpo male pore pe peristomium prpo prostate pore pr prostomium prg prostate gland S segment sd sperm duct spo spermathecal pore. Sources: **A–C** derivatives of figs 8.4B, 8.8A, 8.12A Jamieson (2006).

**Diagnosis (Level 3).** Body elongate, more-or-less equal width along entire length, segments strongly elongate in midbody, with elongate segments having distinct (but truncate) notopodia and low ridge-like neuropodia (tori) (Fig. 63A–F; tor); prostomium narrow, keel- or ridge-shaped (Fig. 63B, ck); first segment chaetous.

**Description.** See Suppl. material 1.

**Remarks.**Maldanidae comprises 41 genera and 269 species (WoRMS 2025), and have a global distribution. Gil (2011) and Parapar et al. (2018) provide a key to European taxa, and Assis et al. (2022) provide a tabulated comparison of all genera of the subfamily Euclymeninae.

**Environment and habitat.** Aquatic, marine; coastal, continental shelf or deep sea; soft or hard substrata (and associated algal mats).

#### ﻿Megascolecidae Rosa, 1891 [megadrile]

Fig. 64

**Common name.** Asian earthworms, giant earthworms.

**LSID.** Urn:lsid:marinespecies.org:taxname:22960.

**Diagnosis (Level 2).** Gizzard present; clitellum situated anterior to male pore (Fig. 64A); gonadal segments bearing genital papillae; spermathecae present, pre-testicular (Fig. 64A); spermathecal pores, 4–5 pairs.

**Description.** See Suppl. material 1.

**Remarks.**Megascolecidae, as perceived here, follows the concept of subfamily Megascolecinae of Jamieson (2000), Jamieson et al. (2002), Dyne and Jamieson (2004) and Jamieson (2006). Although the concept is narrower than other authors accept, it is nevertheless not distinguishable from Acanthodrilidae and Ocnerodrilidae at DELTA Diagnostic Level 3. Megascolecidae is the largest family of ‘earthworms’ with > 2200 spp. and 85 genera (Misirlioğlu et al. 2023), although WoRMS (2025) reports far fewer species presumably because many species names have yet to be formally assessed. Members of one genus, *Pontodrilus*, also occur in the littoral zone. Molecular studies support the monophyly of the family and also show little genetic differentiation within the family, which appears not to support previously suggested taxonomic subdivisions (Misirlioğlu et al. 2023 and references therein). Unfortunately, there appear to be few morphological characters that clearly define the family and groups within.

Megascolecidae is one of only two earthworm families having maritime members – *Pontodriluslitoralis* is found in and above the littoral zone under beach-washed algae and in detritus. It is apparently more common in the tropics and subtropics but has a near cosmopolitan distribution and a complicated synonymy; although some revisions conﬁrm that widespread records can be assigned to a single species (Easton 1984; Anderson et al. 2017), a recent integrative study suggests that morphospecies *P.litoralis* is a complex of four or more cryptic species (Seesamut et al. 2024). Twenty species of the family are thought to have been introduced to Australia (Blakemore 1999). Plisko and Nxele (2015) provide a key to distinguish foreign Megascolecidae taxa from native ones of South Africa. Perhaps the most economically important Megascolecidae is the *Pheretima* group of genera, a clade native to Australia, southeast and eastern Asia, but nowadays globally distributed as a result of introductions; the group currently contains over 1000 species in 14 genera (Chang et al. 2016). Gates and Reynolds (2017) provide a key to North American members of the family, which has been updated by Chang et al. (2016); and James (2004) provides a key to species belonging to several genera in the Solomon Archipelago.

**Environment and habitat.** Terrestrial or aquatic (rarely moist terrestrial, marine, brackish or freshwater; soft substrata.

#### ﻿Melinnidae Chamberlin, 1919 [polychaete]

Fig. 65

**Common name.** Grapple worms.

**LSID.** Urn:lsid:marinespecies.org/aphia.php?p = taxdetails&id = 155483.

**Figures 65, 66.**
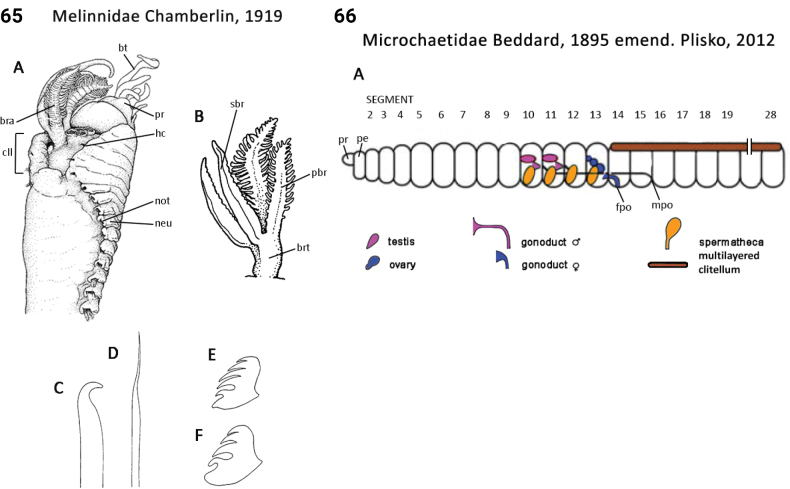
Distinguishing features: **65.**Melinnidae: **A, B.***Isoldapulchella*: **A.** Anterior end dorsolateral view right branchiae removed; **B.** Branchia. **C, D.** Chaetae: **C.** Hook from segment 4; **D.** Acicular neurochaeta from chaetiger 1. **E, F.***Melinnauruguayi*: **E.** Anterior uninus; **F.** Posterior uninus. Abbreviations: bra branchia brt branchial trunk bt buccal tentacles cll colar-like lateral lobe hc hook chaeta neu neuropodium not notopodium pbr pinnate branchia pr prostomium sbr smooth branchia. Sources: **A–D** derivative of fig. 1.112 Beesley et al. (2000), **E, F** derivative of fig. 7, Hessle (1917). **66.**Microchaetidae: **A.** Diagram of reproductive organs, dorsal side up. Abbreviations: fpo female pore mpo male pore pe peristomium pr prostomium. Sources: **A** derivative of fig. 8.4B Jamieson (2006).

**Diagnosis (Level 2).** Buccal tentacles grooved (Fig. 65A); thoracic collar-like dorsolateral expansion present (Fig. 65A; cll); thoracic ventral glandular areas present; second segment chaetous (Fig. 65A).

**Description.** See Suppl. material 1.

**Remarks.**Melinnidae comprise four genera and 56 species (WoRMS 2025), and are globally distributed in marine environments, but more common in deep water. The family was until recently included within Ampharetidae as a subfamily until Stiller et al. (2020) found that it was more closely related to Terebellidae + Trichobranchidae than to Ampharetidae. Nevertheless, morphologically it appears to be more similar to Ampharetidae and is only distinguished from this family at Diagnostic Level 2. Alvestad and Budaeva (2020) provide an interactive key to species of Norwegian seas. Gunton et al. (2023) provide a table with the main diagnostic characteristics of *Melinna* species.

**Environment and habitat.** Aquatic, marine; coastal, continental shelf or deep sea; soft substrata.

#### ﻿Microchaetidae Beddard, 1895 emend. Plisko, 2012 [megadrile]

Fig. 66

**Common name.** African giant earthworms.

**LSID.** Urn:lsid:marinespecies.org:taxname:1045110.

**Diagnosis (Level 2).** Gizzard present; clitellum situated in region of both male and female pores (Fig. 66A); one pair nephridia in each segment (holonephridia); gonadal segments bearing genital papillae; sperm sac present; prostate gland absent.

**Description.** See Suppl. material 1.

**Remarks.**Microchaetidae consists of at least three genera (*Geogenia*, *Microchaetus* and *Proandricus*) and 81 species found only in southern Africa and Madagascar (Misirlioğlu et al. 2023 and references therein). Despite the connotation, the family contains some of the largest earthworms including *Microchaetusrappi*, the African giant earthworm. Another three genera, *Tritogenia*, *Michalakus* and *Kazimierzus* were reported in the family by Plisko (2012). The first two were moved to a new family, Tritogeniidae, by Plisko (2013), but *Kazimierzus* was left in Microchaetidae. We have followed this suggestion – the 25 species of *Kazimierzus* (currently Kazimierzidae), also restricted to South Africa (Misirlioğlu et al. 2023), are unable to be distinguished from Microchaetidae based on the present dataset. It should be noted that Nxele et al. (2016) were able to distinguish Kazimierzidae from Microchaetidae on characters associated with the circulatory and excretory systems and the form of the male gonoducts in relation to their corresponding septa, characters that were not included/scored here. Microchaetidae is not distinguishable from several families at DELTA Diagnostic Level 3. Plisko and Nxele (2015) provide a key to distinguish foreign Microchaetidae taxa from native ones of South Africa.

**Environment and habitat.** Terrestrial; soft substrata.

#### ﻿Microphthalmidae Hartmann-Schröder, 1971, emended Salazar-Vallejo et al. 2019 [polychaete]

Fig. 67

**Common name.** None.

**LSID.** Urn:lsid:marinespecies.org/taxname:322549.

**Figures 67, 68.**
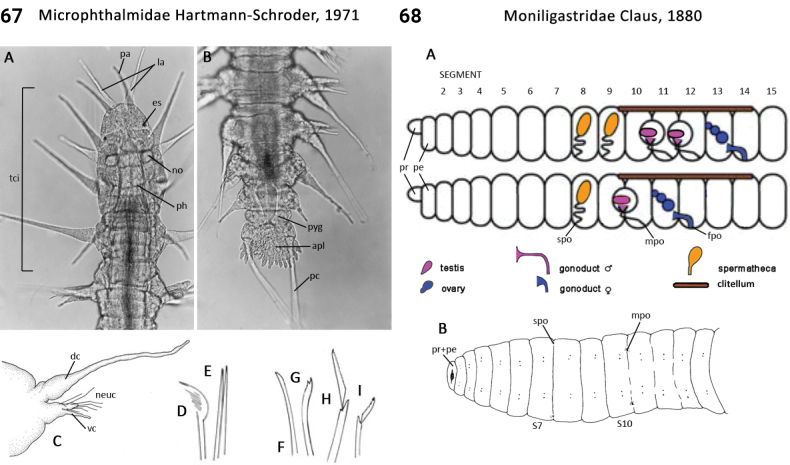
Distinguishing features: **67.**Microphthalmidae: **A–I.***Microphthalmusmahensis*: **A.** Anterior end; **B.** Posterior end showing fimbriate anal plate; **C.** Parapodium from a mid-body chaetiger; **D, E.** Notopodial chaetae: **D.** Pectinate chaeta; **E.** Two straight chaetae; **F–I.** Neuropodial chaetae: **F.** Superiormost simple chaeta; **G.** Inferiormost simple chaeta; **H.** Supraacicular compound chaeta with long blade; **I.** Subacicular compound chaeta with short blade. Abbreviations: apl anal plate dc dorsal cirrus es eye spot la lateral antenna neuc neurochaetae no nuchal organ pa palp pc pygidial cirrus ph pharynx pyg pygidium tci tentacular cirri vc ventral cirrus. Sources: **A–I** derivatives of figs 1, 2 Westheide (2013). **68.**Moniligastridae: **A.** Schematic image of reproductive organs, dorsal side up, of *Desmogaster* (top) and *Drawida* and *Moniligaster* (bottom); **B.***Drawidapolydiverticulata* ventral view. Abbreviations: fpo female pore mpo male pore pe peristomium pr prostomium S segment spo spermathecal pore. Sources: **A** derivative of fig. 8.4 A Jamieson (2006), and fig. 2 Martínez-Ansemil (1993), **B** derivative of fig. 1 A Narayanan et al. (2017), ZooKeys 691: 1–18.

**Diagnosis (Level 3).** Prostomium rounded to oval, antennae present; tentacular cirri present, internal aciculae absent (Fig. 67A); second segment achaetous; pygidium membranous anal plate (Fig. 67B); first chaetiger with both notochaetae and neurochaetae; notopodial lobes absent (Fig. 67C).

**Description.** See Suppl. material 1.

**Remarks.**Microphthalmidae was revised by Salazar-Vallejo et al. (2019a) to accommodate the unplaced interstitial genera *Microphthalmus* and *Hesionides* (removed from Hesionidae by Pleijel and Dahlgren 1998) and four other genera. Note that *Microphthalmus* and *Hesionides* were treated as an unnamed group and coded as such in POLiKEY (Glasby and Fauchald 2003). The family now has eight genera and 61 species (WoRMS 2025) and has a widespread distribution, although records are uneven (far more records in the Northern Hemisphere than the Southern Hemisphere) probably as a result of sampling bias (GBIF.org 2023). Salazar-Vallejo et al. (2019a) provide a key to genera and Westheide (2013) provides a key to *Microphthalmus*.

**Environment and habitat.** Aquatic; marine or freshwater (rarely); coastal or continental shelf or deep sea; soft substrata or epizoic.

#### ﻿Moniligastridae Claus, 1880 [megadrile]

Fig. 68

**Common name.** None.

**LSID.** Urn:lsid:marinespecies.org:taxname:1027685.

**Diagnosis (Level 3).** Chaetae first appear on first segment after peristomium (= S2 for oligochaete workers), arranged in closely spaced lateral and ventrolateral pairs (lumbricine arrangement); clitellum structure single layered but thick (Fig. 68A, B); genital chaetae absent; dorsal pores on mid-dorsal line present; male pores in segment following testicular segment (plesioporous) (Fig. 68A, B); spermathecae with basal diverticula; prostate gland lobular.

**Description.** See Suppl. material 1.

**Remarks.**Moniligastridae is a widespread family especially well represented in the Oriental region. It includes 185 species in five genera (*Desmogaster*, *Drawida*, *Eupolygaster*, *Hastirogaster*, *Moniligaster*), but this species number includes many names that have yet to be formally assessed (WoRMS 2025). Three species, *Drawidabarwelli* (Beddard), *Drawidajaponica* (Michaelsen) and *Drawidanepalensis* Michaelsen have a cosmopolitan distribution (Misirlioğlu et al. 2023). *Drawidabarwelli* has been reported as introduced to Australia (Blakemore (1999). James (2000) provides a key to species of the Samoan Archipelago. Gates and Reynolds (2017) provide a key to North American members of the family.

**Environment and habitat.** Terrestrial, soil.

#### ﻿Myzostomida von Graff, 1877 [polychaete]

Fig. 69

**Common name.** None.

**LSID.** Urn:lsid:marinespecies.org:taxname:233983.

**Figures 69, 70.**
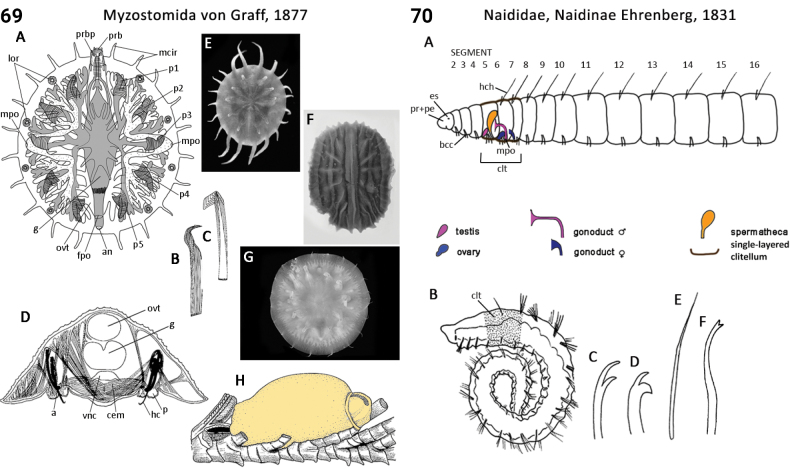
Distinguishing features: **69.**Myzostomida: **A.** General anatomy of Myzostomida; **B.** Hook of *Myzostoma* sp.; **C.** Support rod aciculum of *Myzostomagigas*; **D.** cross-section of *Myzostomaglabrum*; **E–G.** Body shape variation in *Myzostoma* species; **H.***Endomyzostoma* sp. in situ in soft cyst on arm host crinoid. Abbreviations: a acicula an anus cem central muscle fpo female pore g gut hc hook chaeta lor lateral organ mcir marginal cirrus mpo male pore ovt ovarian tube p parapodium prb proboscis prbp proboscis papilla vnc ventral nerve cord. Sources: **A** redrawn from fig. 1 Graff (1884), **B–H** derivative of figs 2.1, 2.2, 2.3, 2.4, 2.10, 2.13 Beesley et al. (2000). **70.**Naididae, Naidinae: **A.** Schematic diagram showing location of reproductive organs, dorsal side up; **B–E.***Naisbarbata*: **B.** General view of a sexually mature specimen with clitellum; **C.** Anterior ventral chaeta; **D.** Posterior ventral chaeta; **E.** Needle chaeta; **F.***Naiscommunis* bifid needle chaeta. Abbreviations: bcc bifid crotchet chaeta clt single-layered clitellum es eye spot hch hair chaetae mpo male pore pe peristomium pr prostomium. Sources: **A** derivative of fig. 12.3 Thorp et al. (2019), **B–F** derivatives of Timm (2009: 55, 63).

**Diagnosis (Level 3).** Body shape usually circular; body margins cirrate, scalloped or irregular; body segmentation absent (Fig. 69A, E–G; mcir)

**Description.** See Suppl. material 1.

**Remarks.**Myzostomida is treated here as an order within Polychaeta following Rouse et al. (2022). Their position as part of Annelida still appears to be uncertain despite Weigert et al. (2014) finding them to be firmly placed within Annelida as sister group to Errantia based on phylogenomic data. The order comprises seven families, 12 genera and 194 species (WoRMS 2025). Current distribution records suggest the family is globally distributed (GBIF.org 2023). Parapar et al. (2018) provide a key to the myzostomes of Spain.

**Environment and habitat.** Aquatic, marine; coastal, continental shelf or deep sea; soft or hard substrata or epizoic.

#### ﻿Naididae Ehrenberg, 1831 [microdrile]

**Common name.** None.

**LSID.** Urn:lsid:marinespecies.org:taxname:2039.

**Diagnosis (Level 0).** Prostomium not demarcated (zygolobic); chaetae present, from first segment after peristomium (= S2); male pores in segment following testicular segment (plesioporous); spermathecae post-testicular (less often) or in testicular segments (usually).

**Description.** See Suppl. material 1.

**Remarks.**Naididae sensu lato represents a massive, morphologically disparate group of taxa that includes nine subfamilies some of which are separately coded in this dataset (viz., Naidinae, Opistocystinae, Pristininae and Tubificinae); the remainder were not coded because, although they are clearly separable morphologically, the level of detail in the present dataset was insufficient to discern them, including Limnodriloidinae (12 genera), Phallodrilinae (35 genera), Rhyacodrilinae (20 genera), Rhyacodriloidinae (1 genus) and Telmatodrilinae (4 genera). Further, given that we found that the family is not diagnosable (DELTA Diagnostic Level 0), as it is not separable from Dorydrilidae and Phreodrilidae, future efforts to define subfamilies using a more detailed genital anatomy character set may be fruitful. The reader is referred to the full description to verify identification. Naididae sensu lato and Phreodrilidae together comprise the order Tubificida Jamieson, 1978 (Jamieson 1978; Schmelz et al. 2021). Phallodrilinae includes some species-rich genera, such as *Phallodrilus* Pierantoni, 1902 (99 species and subspecies), and aberrant forms, such as the chemoheterotrophs *Olavius* and *Inanidrilus*, which lack a gut and instead rely on bacterial symbionts for food. Rhyacodrilinae includes the species-rich genus *Rhyacodrilus* Bretscher, 1901 (77 species), known from subterranean and epigean waters (Martin et al. 2010; T. Timm, pers. comm., Jan. 2025), and the cosmopolitan species *Branchiurasowerbyi* Beddard, 1892.

**Environment and habitat.** Terrestrial (moist terrestrial) or aquatic; marine, brackish, freshwater; coastal, continental shelf, deep sea, littoral or supralittoral; soft substrata, or epizoic.

#### ﻿Naididae, Naidinae Ehrenberg, 1831 [microdrile; alternative representation “Naididae”]

Fig. 70

**Common name.** None.

**LSID.** Urn:lsid:marinespecies.org:taxname:176043.

**Diagnosis (Level 3).** Prostomium with eyes (usually); spermathecae pre-testicular or in testicular segment (Fig. 70A); nephridial pores located posterior to gonoducts; penis absent.

**Description.** See Suppl. material 1.

**Remarks.**Naidinae is a large subfamily comprising 24 genera and over 600 species and subspecies (WoRMS 2025). The largely epibenthic fresh- and brackish-water Naidinae can be differentiated ecologically from sediment-dwelling Naididae such as Tubificinae (Timm 2009). Morphologically, they differ from other Naididae subfamilies by the more anterior position of the genital system (clitellum on IV–VIII), with male pores usually in V or VI, and spermathecae and spermathecal pores paired in testicular segment IV or VI. Naidinae have been recorded from all continents (Martin et al. 2008). Some species are commensal, for example, the Australian endemic Dero (Allodero) litoria, which inhabits the ureters of the Green Tree Frog (*Litoriacaerulea*) and possibly other frogs (Pinder and Brinkhurst 1998; Pinder et al. 1998). Brinkhurst and Jamieson (1971) provide keys to genera and species at the time. Pinder (in press) provides a key to Australian species. Worsfold (2003) provides an introduction and key to oligochaetes, including Naidinae, of the NE Atlantic.

**Environment and habitat.** Aquatic (including moist terrestrial), freshwater (mostly, rarely brackish); soft substrata, or endozoic (some species of Dero (Allodero) are internal parasites of frogs; others live in the mantle cavity of mollusks).

#### ﻿Naididae, Opistocystinae Černosvitov, 1936 [microdrile; alternative representation “Opistocystidae”]

Fig. 71

**Common name.** None.

**LSID.** Urn:lsid:marinespecies.org:taxname:1347251.

**Figures 71, 72.**
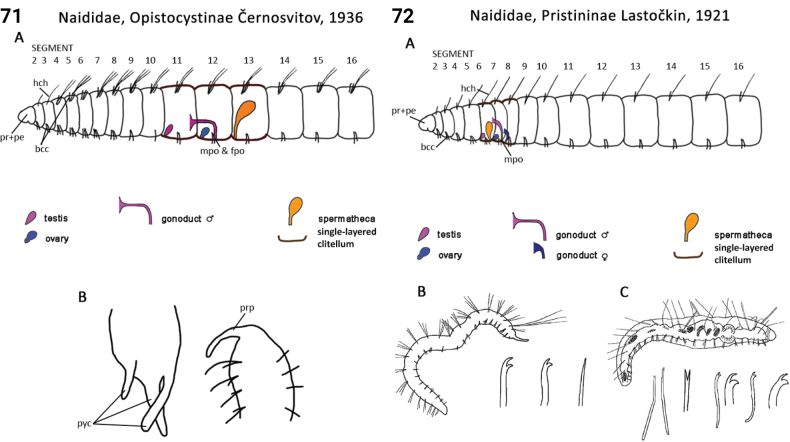
Distinguishing features: **71.**Naididae, Opistocystinae: **A.** Diagram showing location of reproductive organs, dorsal side up; **B.***Crustipellis* sp., left to right: tail end, head end. Abbreviations: bcc bifid crotchet chaeta clt single-layered clitellum fpo female pore hch hair chaetae mpo male pore pe peristomium pr prostomium prp prostomial palpode pyc pygidial cirrus. Sources: **A** derivatives of fig. 12.3 Thorp et al. (2019), **B** derivatives of fig. 11 Brinkhurst and Gelder (2001). **72.**Naididae, Pristininae: **A.** Schematic diagram showing location of reproductive organs, dorsal side up; **B.***Pristinalongiseta*, left to right: whole body, ventral crotchet chaeta (2), needle chaeta; **C.***Pristinabilobata*, left to right: whole body, needle chaetae (3), anterior ventral chaeta (2), posterior ventral crotchet chaeta (2). Abbreviations: bcc bifid crotchet chaeta hch hair chaetae mpo male pore pe peristomium pr prostomium. Sources: **A** derivative of fig. 12.3 Thorp et al. (2019), **B, C** derivatives of Timm (2009: 67, 69).

**Diagnosis (Level 3).** Eyes on head absent; branchiae present; three pygidial (caudal) appendages present (Fig. 71B); spermathecae post-testicular (Fig. 71A); penis present.

**Description.** See Suppl. material 1.

**Remarks.**Opistocystinae is recognised at the subfamily level following Erséus et al. (2010a, 2017). Opistocystinae contains three genera, *Opistocysta* Černosvitov, 1936, *Trieminentia* Harman & Loden, 1978 and *Crustipellis* Harman & Loden, 1978 (WoRMS 2025), and is largely Neotropical in distribution. The subfamily is known from the Americas and there is a single record from Africa, which may have been introduced (Harman 1969; Erséus 2010a). Brinkhurst and Jamieson (1971) provide keys to genera and species at the time.

**Environment and habitat.** Terrestrial (moist terrestrial) or aquatic.

#### ﻿Naididae, Pristininae Lastočkin, 1921 [microdrile]

Fig. 72

**Common name.** None.

**LSID.** Urn:lsid:marinespecies.org:taxname:1040009 (as subfamily).

**Diagnosis (Level 3).** Prostomium not demarcated (zygolobic); eyes absent; needle chaetae present (Fig. 72B, C); spermathecae in testicular segment (Fig. 72A); male pores in segment VIII following testicular segment (plesioporous) (Fig. 72A); nephridial pores and gonoducts located around clitellum (Fig. 72A); pygidial (caudal) appendages absent (Fig. 72B, C); prostate gland absent.

**Description.** See Suppl. material 1.

**Remarks.**Pristininae is recognised at the subfamily level following Erséus et al. (2010a, 2017). In contrast, Timm (2009) treats the taxon at the family level. Morphologically, they differ from other Naididae subfamilies by lacking eyes and pygidial gills and having testes and spermathecae in VII and the male pore in VIII. Pristininae has two genera (*Pristina* Ehrenberg, 1828 and *Pristinella* Brinkhurst, 1985) and over 100 species (Martin et al. 2023b) and is globally distributed in freshwater. Pinder (in press) provides a key to Australian species but notes that the family’s true diversity is poorly known.

**Environment and habitat.** Terrestrial (moist) or aquatic (freshwater).

#### ﻿Naididae, Tubificinae d’Udekem, 1855 [microdrile; alternative representation “Tubificidae”]

Fig. 73

**Common name.** Sludge worms.

**LSID.** Urn:lsid:marinespecies.org:taxname:137344.

**Figures 73, 74.**
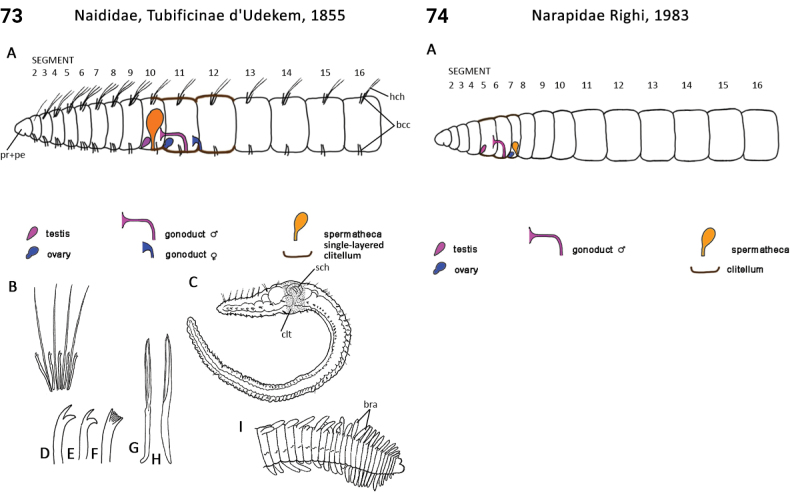
Distinguishing features: **73.**Naididae, Tubificinae: **A.** Diagram showing location of reproductive organs, dorsal side up; **B.** Dorsal chaetal bundle with hair and bifid (or pectinate) chaetae; **C–H.***Potamothrixhammoniensis*: **C.** General view of sexual mature specimen with clitellum; **D.** Anterior ventral chaeta; **E.** Posterior ventral chaeta; **F.** Pectinate chaeta; **G, H.** Spermathecal chaetae; **I.***Branchiurasowerbyi*, posterior end with gills. Abbreviations: bcc bifid crotchet chaeta bra branchia clt single-layered clitellum hch hair chaetae pe peristomium pr prostomium sch spermathecal chaeta. Sources: **A** derivative of fig. 12.3 Thorp et al. (2019), **B–I** derivatives of Timm (2009: 85, 97). **74.**Narapidae: **A.** Schematic diagram showing location of reproductive organs, dorsal side up. Sources: **A.** Derivative of fig. 12.3 Thorp et al. (2019) (modification of schematic diagram of Naididae based on information in Erséus (2005), fig. 1; chaetae removed from diagram).

**Diagnosis (Level 3).** Prostomium bluntly conical and demarcated from peristomium without a tongue (prolobic), without an anterior projection, eyes absent (Fig. 73A, C); prostate gland present; nephridial pores located anteriorly, gonoducts located around clitellum (Fig. 73A); pygidial appendages (branchiae) absent (Fig. 73C, I).

**Description.** See Suppl. material 1.

**Remarks.**Tubificinae is a large subfamily comprising 37 genera and over 850 species and subspecies (WoRMS 2025), and is the most species-rich marine oligochaete group. Erséus et al. (2000) established its status as a subfamily of Naididae. The subfamily includes the common bioturbator annelids *Limnodrilus* Claparède, 1862. Tubificinae also includes *Tubifextubifex* (Müller, 1774), one of the most commonly studied freshwater oligochaetes. Tubificinae have been recorded from all continents in freshwater, brackish and marine habitats; they dominate in Holarctic freshwaters (Martin et al. 2008). Brinkhurst and Jamieson (1971) provide keys to genera and species at the time. Brinkhurst (1982) provides a key to marine and estuarine European species. Pinder (in press) provides a key to Australian species while acknowledging its true diversity is unknown. Worsfold (2003) provides an introduction and key to oligochaetes, including Tubificinae, of the Northeast Atlantic.

**Environment and habitat.** Aquatic (including subterranean or hyporheic); marine, brackish, or freshwater; coastal, continental shelf, deep sea, littoral or supralittoral; soft substrata.

#### ﻿Narapidae Righi & Varela, 1983 [microdrile]

Fig. 74

**Common name.** None.

**LSID.** Urn:lsid:marinespecies.org/aphia.php?p = taxdetails&id = 1039998.

**Diagnosis (Level 3).** Chaetae absent; ovaries, unpaired; female gonoduct absent (Fig. 74A); spermathecae post-testicular

**Description.** See Suppl. material 1.

**Remarks.**Narapidae is a monotypic family occurring only in South America (WoRMS 2025). Its only species is poorly known, yet it is still diagnosable at Level 2; beyond that, it is not separable from Lumbriculidae.

**Environment and habitat.** Aquatic, freshwater.

#### ﻿Nephtyidae Grube, 1850 [polychaete]

Fig. 75

**Common name.** Catworms.

**LSID.** Urn:lsid:marinespecies.org:taxname:956.

**Figures 75, 76.**
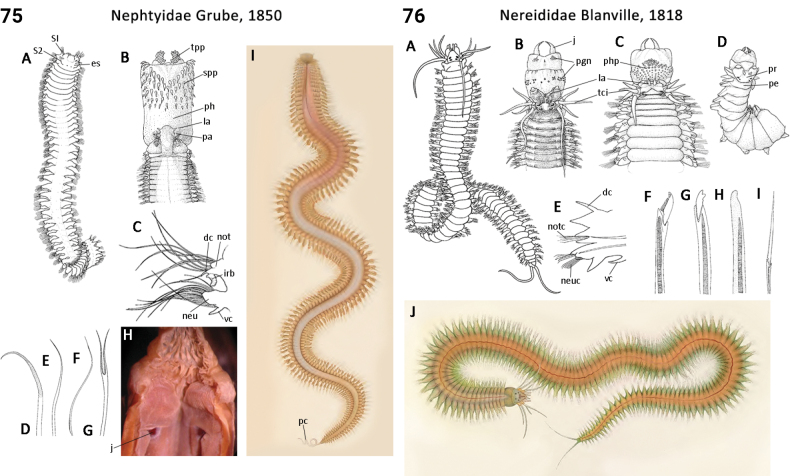
Distinguishing features: **75.**Nephytidae: **A.** Entire animal of *Nephtysinornata* dorsal view; **B.** Anterior end of *Nephtyslongipes* with pharynx everted dorsal view; **C.** Parapodium of *Nephtyslongipes* from chaetiger 15; **D, E.** Chaetae of *Nephtysinornata*: **D.** Barred notochaeta; **E.** Capillary notochaeta; **F.** Capillary neurochaeta of *Nephtyslongipes*; **G.** Lyrate chaeta from neuropodium of *Inermonephtyspalpata*; **H.**Nephtyscf.tulearensis preserved specimen dissected pharynx; **I.***Nephthyscaeca* entire animal. Abbreviations: dc dorsal cirrus es eye spot irb interramal branchia j jaw la lateral antenna neu neuropodium not notopodium pa palp pc pygidial cirrus ph pharynx SI segment 1 S2 segment 2 spp subterminal pharyngeal papilla tpp terminal pharyngeal papilla vc vental cirrus. Sources: **A–G** after fig. 1.79, Beesley et al. (2000), **H** after fig. 1H Ravara et al. (2022), **I** MacIntosh (1900–1922), pl. XLIII, fig. 1. **76.**Nereididae: **A.** Entire animal of *Platynereisantipoda*; **B–D.** Anterior ends with pharynx everted: **B.***Neanthesvaalii*; **C.***Australonereisehlersi*; **D.***Namanereislittoralis* Hutchings & Turvey, 1982; **E.** Parapodium from chaetiger 20 of *Neanthescricognatha*; **F.** Notopodial heterogomph falciger *Nereismaxillodentata*; **G.** Notopodial homogomph falciger from median parapodium *Nereismaxillodentata*; **H.** Simple falciger *Simpliseta* sp.; **I.** Notopodial heterogomph spiniger from *Neantheskerguelensis*; **J.***Hedistediversicolor*. Abbreviations: dc dorsal cirrus j jaw la lateral antenna neuc neurochaetae notc notochaetae pe peristomium pgn pharyngeal paraganths php pharyngeal papillae pr prostomium tci tentacular cirri vc ventral cirrus. Sources: **A–I** derivatives of fig. 1.80 Beesley et al. (2000), **J** derivative of MacIntosh (1900–1922), pl. LII, fig. 3.

**Diagnosis (Level 3).** Prostomium pentagonal to quadrangular in shape, antennae present (Fig. 75A, B); postcephalic subdermal eyes may be present (Fig. 75A, es); interramal fleshy process present (Fig. 75C; irb); pygidial appendages present (Fig. 75I; pc), including a small single medial cirrus; pharyngeal jaws present (Fig. 75H).

**Description.** See Suppl. material 1.

**Remarks.**Nephtyidae includes four genera and 150 species and is globally distributed (WoRMS 2025). Ravara et al. (2010) performed the first phylogenetic study of the family and included a key to genera. The most recent phylogenetic study including Nephtyidae found them to be the sister group of Pilargidae (Tilic et al. 2022), which may explain the presence of bifid palps in *Bipalponephtys* Ravara, Wiklund, Cunha & Pleijel, 2010, a feature only known outside Nephtyidae in some members of Pilargidae. Gil (2011), Kuş et al. (2021) and Dnestrovskaya (2020) provide keys to European taxa.

**Environment and habitat.** Aquatic, marine; coastal, continental shelf or deep sea; soft substrata.

#### ﻿Nereididae Blainville, 1818 [polychaete]

Fig. 76

**Common name.** Ragworms, clam worms, pile worms.

**LSID.** Urn:lsid:marinespecies.org:taxname:22496.

**Dianosis (Level 3).** Prostomium bluntly conical (or inverted T-shaped) bearing two pairs of eyes and a pair of antennae; palps present, bi-articulated; tentacular cirri present; peristomium (also called a tentacular belt) visible (Fig. 76A–D); pharynx jaws present (Fig. 76B); capillary chaetae absent.

**Description.** See Suppl. material 1.

**Remarks.**Nereididae contains 46 genera and 771 species (WoRMS 2025) and is globally distributed. Three or four subfamilies are recognised, but not all are monophyletic, so they are not coded in this dataset. Gil (2011) and Viéitez et al. (2004) provide keys to European taxa, Hsueh (2022) provides a key to *Platynereis* Kinberg, 1865 species of East Asia, while Wilson et al. (2023) provide both dichotomous and interactive keys to all genera, as well as many additional references to species-level keys.

**Environment and habitat.** Terrestrial (rarely) or aquatic, including moist terrestrial, subterranean, hyporheic (rarely), marine, brackish or freshwater; coastal, continental shelf, deep sea, littoral or supralittoral; soft or hard substrata, or hydrothermal vents and cold seeps (including methane seeps), or epizoic.

#### ﻿Nerillidae Levinsen, 1883 [polychaete]

Fig. 77

**Common name.** None.

**LSID.** Urn:lsid:marinespecies.org:taxname:992.

**Figures 77, 78.**
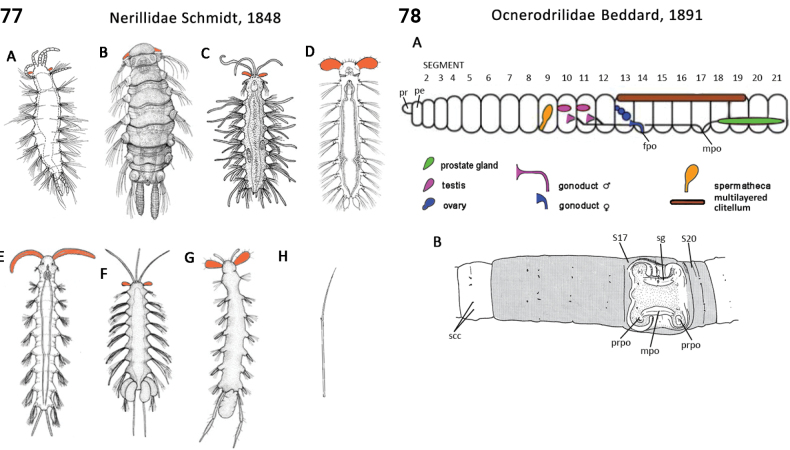
Distinguishing features: **77.**Nerillidae: **A–G.** Variation in body morphology across a selection of nerillid genera (all dorsal views); **A.** Entire animal of *Nerillaaustralis*; **B.***Paranerillacilioscutata*; **C.***Leptonerillaprospera*; **D.***Thalassochaetuspalpifoliaceus*; **E.***Meganerillaswedmarki*; **F.***Mesonerillaintermedia*; **G.***Nerillidiummediterraneum*; **H.** Compound chaeta of *Mesonerillaroscovita*. Sources: **A, H** derivatives of fig. 1.93 Beesley et al. (2000), **B–G** derivatives of fig. 5C, **E, I, L, N, Q** of Worsaae (2021), original sources as indicated therein. Palps coloured to emphasize shape variation. **78.**Ocnerodrilidae: **A.** Schematic image of reproductive organs, dorsal side up; **B.***Eukerriaborellii*, ventral view, showing seminal grooves connecting prostate and male pores. Abbreviations: fpo female pore mpo male pore pe peristomium pr prostomium prpo prostate pore S segment scc simple crochet chaeta sg seminal grove. Sources: **A, B** derivatives of fig. 8.4 B, 8.9 Jamieson (2006).

**Diagnosis (Level 3).** Body short with a fixed segment number (Fig. 77A–G); secondary annulation present (Fig. 77B, E); prostomial antennae present (usually); peristomium not visible; interramal fleshy processes present; pygidial appendages present (Fig. 77A–G).

**Description.** See Suppl. material 1.

**Remarks.**Nerillidae contains 16 genera and 60 species. The family is widely distributed, although apparently under-sampled in the Southern Hemisphere (GBIF.org 2023). Worsaae (2005, 2021) provided a morphological phylogenetic analysis of the family and a key and diagnoses of its genera; Worsaae (2021) presented phylogenomic evidence supporting the position of the meiofaunal-sized Nerillidae within the subclass Errantia. Gil (2011) provides a key to European taxa.

**Environment and habitat.** Terrestrial or aquatic including subterranean or hyporheic (phreatic and hyporheic caves, wells, and springs) and marine; coastal, continental shelf, deep sea, littoral or supralittoral; soft substrata.

#### ﻿Ocnerodrilidae Beddard, 1891 [megadrile]

Fig. 78

**Common name.** None.

**LSID.** Urn:lsid:marinespecies.org:taxname:1039999.

**Diagnosis (Level 1).** Clitellum fully encircles body, situated in region of male pore (Fig. 78A); tubercula pubertatis present, forming paired ridges on the ventrolateral margins of clitellum (Fig. 78B); prostate gland present, tubular.

**Remarks.**Ocnerodrilidae is similar to Megascolecidae, Criodrilidae and Eudrilidae and only distinguishable from these taxa at DELTA Diagnostic Level 1. Ocnerodrilidae is a widespread family comprising 37 genera and 172 species (Misirlioğlu et al. 2023), but this species number includes many names that have yet to be formally assessed (WoRMS 2025). Several species have a worldwide distribution (Misirlioğlu et al. 2023), including four species introduced to Australia (Blakemore 1999). Plisko and Nxele (2015) provide a key to distinguish foreign Ocnerodrilidae taxa from native ones of South Africa. Fragoso and Rojas (2009) provide a key to the genera of Ocnerodrilidae. Gates and Reynolds (2017) provide a key to North American members of the family.

**Environment and habitat.** Terrestrial or aquatic; moist terrestrial (including leaf axils), freshwater; soft substrata.

#### ﻿Oenonidae Kinberg, 1865 [polychaete]

Fig. 79

**Common name.** None.

**LSID.** Urn:lsid:marinespecies.org:taxname:22610.

**Figures 79, 80.**
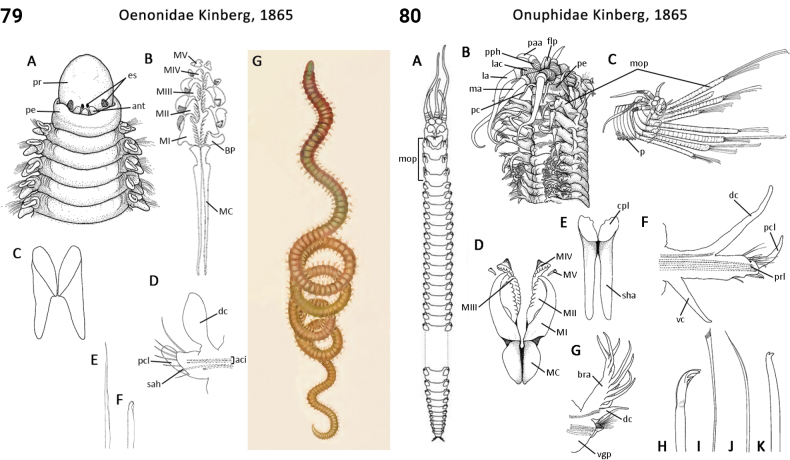
Distinguishing features: **79.**Oenonidae: **A–F.***Oenonefulgida*; **A.** Anterior end dorsal view; **B, C.** Jaw parts: **B.** Maxillae dorsal view; **C.** Mandibles ventral view; **D.** Parapodium from chaetiger 45; **E, F.** Chaetae from parapodium of chaetiger 45: **E.** Simple limbate chaeta; **F.** Subacicular hook; **G.***Arabellairicolor* entire animal. Abbreviations: aci aciculae ant antennae BP base plate dc dorsal cirrus es eye spot MI maxilla 1 MII maxilla 2 MIII maxilla 3 MIV maxilla 4 MV maxilla 5 MC maxillary carrier pcl postchaetal lobe pe peristomium pr prostomium sah subacicular hook. Sources: **A–F** derivatives of fig. 1.61 Beesley et al. (2000), **G** derivative of MacIntosh (1900–1922), pl. LIV, fig. 4. **80.**Onuphidae: **A.** Entire animal ventral view; **B.** Anterior end of *Hirsutonuphismariahirsuta* dorsolateral view; **C.** Anterior end of *Longibrachiumlongipes* dorsolateral view; **D, E.** Jaw parts of *H.mariahirsuta*: **D.** Maxillae dorsal view; **E.** Mandible ventral view; **F.** Parapodium of *H.mariahirsuta* from chaetiger 3; **G.** Posterior parapodium of *H.mariahirsuta* from chaetiger 30; **H.** Pseudocompound hook from chaetiger 3 of *H.mariahirsuta*; **I.** Pectinate chaetae from chaetiger 31 of *H.mariahirsuta*; **J.** Limbate chaeta; **K.** Subacicular hook. Abbreviations: bra branchia cpl cutting plate dc dorsal cirrus flp frontal lip la lateral antenna lac lateral antenna ceratophore M maxilla MC maxillary carrier ma median antenna mop modified parapodium p parapodium paa palpal antenna pc pygidial cirrus pcl postchaetal lobe pe peristomium pph palophore prl prechaetal lobe sha shaft vc ventral cirrus vgp ventral glandular pad. Sources: **A** derivative of fig. 3 Budaeva (2021), **B–K** derivatives of fig. 1.62 Beesley et al. (2000).

**Diagnosis (Level 3).** Discrete head lobe-like without appendages; prostomium bluntly conical; eyes present on head (Fig. 79A, G); first chaetiger with both notochaetae and neurochaetae (Fig. 79A); dorsal cirri present (Fig. 79D); multiple jaws present (4–5 toothed plates), maxillary carriers longer than combined length of maxillae (Fig. 79B).

**Description.** See Suppl. material 1.

**Remarks.**Oenonidae comprises 16 valid genera and 97 species (WoRMS 2025), and has a global distribution. The family is present worldwide but appears to be less species-rich in the Antarctic and oceanic regions (GBIF.org 2023); they are never abundant. Gil (2011) and Parapar et al. (2018) provide keys to European taxa. Zanol and Ruta (2015) provide a key to taxa from the northern Great Barrier Reef, Australia. Rizzo et al. (2020) provide a key to all species of *Labrorostratus* Saint-Joseph, 1888.

**Environment and habitat.** Aquatic, marine; coastal, continental shelf, deep sea; soft substrata or endozoic (*Labrorostratus* species are endoparasites of other polychaetes).

#### ﻿Onuphidae Kinberg, 1865 [polychaete]

Fig. 80

**Common name.** Beach worms (Australia).

**LSID.** Urn:lsid:marinespecies.org:taxname:965.

**Diagnosis (Level 3).** Prostomium antennae present, antennae consisting of basal ceratophore and distal ceratostyle; frontal lips present (Fig. 80B, flp); peristomium as a single ring; comb-like chaetae present (Fig. 80I).

**Description.** See Suppl. material 1.

**Remarks.**Onuphidae comprises two subfamilies, Hyalinoeciinae and Onuphinae (Paxton 1986; Budaeva et al. 2016), with 5/17 genera, respectively, and 354 species in all (WoRMS 2025); the family is globally distributed. Paxton (1986) provides a key to world genera; Paxton (1996) provides a key to Australian *Hirsutonuphis* Paxton, 1986; Paxton and Budaeva (2013) provide a key to world *Paradiopatra* Ehlers, 1887 species. Paxton (2017) provides a table and key to distinguish species of *Aponuphis*; and Budaeva and Fauchald (2008) provide a key to Caribbean onuphids.

**Environment and habitat.** Aquatic, marine; coastal, continental shelf, deep sea; soft or hard substrata.

#### ﻿Opheliidae Malmgren, 1867 [polychaete]

Fig. 81

**Common name.** None.

**LSID.** Urn:lsid:marinespecies.org:taxname:924.

**Figures 81, 82.**
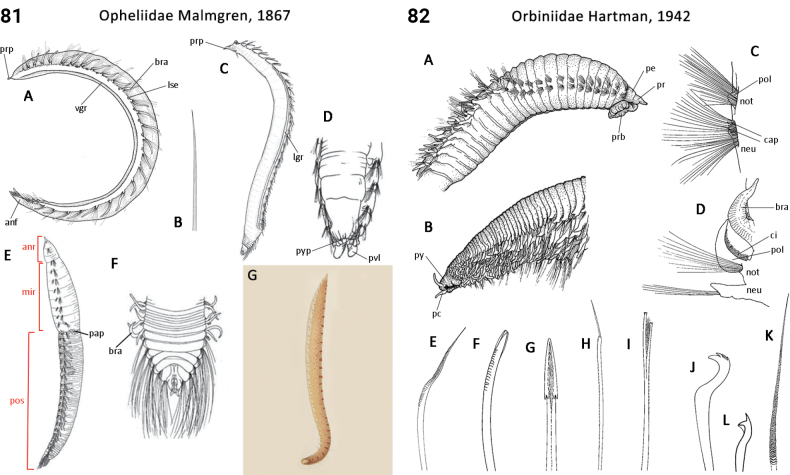
Distinguishing features: **81.**Opheliidae: **A, B.***Armandiaintermedia*: **A.** Entire animal lateral view; **B.** Capillary chaeta from mid-body parapodium; **C, D.***Opheliaalgida*: **C.** Entire animal; lateral view; **D.** Posterior end dorsal view; **E, F.***Thoracopheliamammillatus*: **E.** Entire animal, lateral view; **F.** Posterior end dorsal view; **G.***Polyophthalmuspictus*, entire animal. Abbreviations: anf anal funnel anr anterior region bra branchia lgr lateral groove lse lateral segmental eyespot mir mid region pap papilla por posterior region prp prostomial palpode pvl pygidial ventral lobe pyp pygidial papilla vgr ventral groove. Sources: **A, B** derivatives from fig. 1.52 Beesley et al. (2000), **C–F** derivatives of figs 7.6.1.4, 7.6.1.5 Blake and Maciolek (2019f), **G** derivative from MacIntosh (1900–1922), pl. LXXXVIII, fig. 2. **82.**Orbiniidae: **A.** Anterior end of *Scoloplosnormalis* lateral view; **B.** Posterior end of *Scoloploscylindrifer* dorsolateral view; **C, D.** Parapodia of *Scoloplosnormalis*: **C.** Thoracic parapodium from chaetiger 4 anterior view; **D.** Mid-abdominal parapodium from chaetiger 28 anterior view; **E–K.** Chaetae: **E.**Subuluncini from thoracic neuropodium of chaetiger 9 *Nainerisaustralis*; **F.** Hooded spine from thoracic neuropodium of chaetiger 9 *N.australis*; **G.** Arrow-shaped spine from thoracic neuropodium of chaetiger 14 *Phylofelix*; **H.** Flail-tipped chaeta from abdominal neuropodium *Orbiniahartmanae*; **I.** Furcate abdominal chaeta *P.felix*; **J.** ‘Swanshaped’ hook from abdominal neuropodium *Proscoloplosconfusus*; **K.** Crenulate capillary from abdominal neuropodium of chaetiger 28 *S.normalis*; **E.** Hook from chaetiger 13. Abbreviations: bra branchia cap capillary chaetae ci cilia neu neuropodium not notopodium pc pygidial cirrus pe peristomium pol postchaetal lobe pr prostomium prb proboscis py pygidium. Sources: **A–K** derivatives from figs 1.53, 1.55 Beesley et al. (2000).

**Diagnosis (Level 2).** Epidermis more-or-less smooth, discrete head lobe-like without appendages but with an anterior extension (palpode) (Fig. 81A, C), ventral groove present (Fig. 81A, vgr), sometimes also lateral grooves (Fig. 81C, lgr); pharynx with dorsolateral ciliated folds present.

**Description.** See Suppl. material 1.

**Remarks.**Opheliidae comprises eight genera and 177 species (WoRMS 2025), and is present worldwide (GBIF.org 2023). *Travisia* Johnston, 1840 was part of Opheliidae for many years until Blake (2000) showed that the genus should be part of Scalibregmatidae on the basis of morphological similarities, which was later confirmed by molecular systematic studies. Nevertheless, the present dataset highlights the similarities between these groups as Opheliidae is only distinguished from Scalibregmatidae at Diagnostic Level 2. Gil (2011) and Parapar et al. (2012) provide keys to European taxa. Parapar et al. (2023) provide a key for species of *Ophelina* Örsted, 1843 in the Indo-Pacific, including southern Asia, Indo-Malay Archipelago and Australia. Magalhães et al. (2019) and Parapar et al. (2021a) tabulate key morphological characters of species and provide a key to *Polyophthalmus* Quatrefages, 1850 from the Indian and Pacific Oceans, respectively.

**Environment and habitat.** Aquatic, marine; coastal, continental shelf or deep sea; soft substrata (usually clean, fine to medium sand).

#### ﻿Orbiniidae Hartman, 1942 [polychaete]

Fig. 82

**Common name.** None.

**LSID.** Urn:lsid:marinespecies.org:taxname:902.

**Diagnosis (Level 3).** Body elongate, more-or-less equal width along entire length; body regionalization present, two regions demarcated by laterally-directed parapodia (thorax) and dorsally-directed parapodia (midbody and abdomen) (Fig. 82A, B); head lobe-like, without appendages (Fig. 82A); pharynx jaws absent; branchiae present (Fig. 82D); capillary chaetae, internally distinctly chambered (Fig. 82K).

**Description.** See Suppl. material 1.

**Remarks.**Orbiniidae comprises 21 genera and 238 species and is present worldwide (GBIF.org 2023). Gil (2011) and Parapar et al. (2012) provide keys to European taxa Orbiniidae, and also to Questidae, which are now considered a part of Orbiniidae. Note that Questidae was treated as a family in POLiKEY2 (Glasby and Fauchald 2003). Sun et al. (2018) provide a key to all known species of *Leodamas* Kinberg, 1866. Núñez and Martínez (2023) provide a key to *Questa* of the world. *Proscoloploscygnochaetus* Day, 1954 appears to be one of few genuine cosmopolitan species based on a morphological and molecular systematic study (Meyer et al. 2008), and currently the only accepted species in the genus (WoRMS 2025).

**Environment and habitat.** Aquatic, marine or brackish; coastal, continental shelf, deep sea; soft substrata or hydrothermal vents and cold seeps (rarely).

#### ﻿Orobdellidae Nakano, Zainudin & Hikida, 2012 [leech]

Fig. 83

**Common name.** None.

**LSID.** Urn:lsid:marinespecies.org:taxname:1593940.

**Figures 83, 84.**
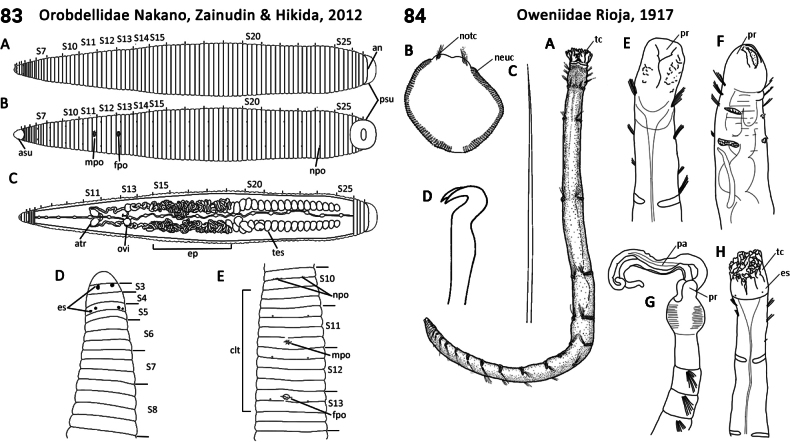
Distinguishing features: **83.**Orobdellidae: **A–C.***Orobdellanaraharaetmagarum*, external (**A, B**) and internal (**C**) view showing alimentary canal and male and female reproductive systems; **D, E.***O.naraharaetmagarum* showing anterior end, dorsal (**D**) and midbody, ventral (**E**), showing pores. Abbreviations: an anus, asu anterior sucker, atr atrium, clt clitellum, ep epididymus, es eye spot, fpo female pore, mpo male pore, npo nephridiopore, ovi ovisac, psu posterior sucker, S segment, tes testis. Sources: **A–E** derived from fig. 1, fig. 3 Nakano (2016a). **84.**Oweniidae: **A–D.**Oweniacf.fusiformis: **A.** Entire animal, dorsal view; **B.** Transverse section of body showing chaetal distribution; **C.** Capillary notochaeta from parapodium of chaetiger 10; **D.** Neurochaetal hook from parapodium of chaetiger 10; **E–H.** Head ends of different genera: **E.***Galathoweniaoculata*; **F.***Myriocheleheeri*; **G.***Myrioweniacaliforniensis*; **H.***Oweniaaustralis*. Abbreviations: es eye spots neuc neurochaetae notc notochaetae tc tentacular crown pa palp pr prostomium. Sources: **A–D** derivatives of fig. 1.97 Beesley et al. (2000), **E–H** derivatives of fig. 4.1.1 Capa et al. (2019c).

**Diagnosis (Level 3).** Body dorsoventrally flattened; head eyes; number one to three pairs (Fig. 83D, es); jaws paired with fine teeth or a series of soft teeth; testes arranged in multiple grape-like clusters per segment (Fig. 83C, tes); egg sacs globular (Fig. 83C); caeca of midgut absent.

**Description.** See Suppl. material 1.

**Remarks.**Orobdellidae belongs to the jawed Hirudiniformes (Arhynchobdella) and is one of the most recently described families of leeches. Established for the genus *Orobdella* by Nakano et al. (2012), it contains ~ 20 species from East Asia (Nakano 2016b; Nakano and Prozorova 2019) and remains restricted to the eastern Palaearctic.

**Environment and habitat.** Terrestrial (moist terrestrial); soft substrata.

#### ﻿Oweniidae Rioja, 1917 [polychaete]

Fig. 84

**Common name.** None.

**LSID.** Urn:lsid:marinespecies.org:taxname:975.

**Diagnosis (Level 3).** Body regionalization present; two body regions demarcated by a change in chaetal types along body (Fig. 84A); mid-body segments strongly elongate bearing distinct (but truncate) notopodia, and neuropodial tori with hook chaetae (Fig. 84B, notc, neuc).

**Description.** See Suppl. material 1.

**Remarks.**Oweniidae comprises four genera and 57 species (WoRMS 2025), and is present worldwide (GBIF.org 2023). Gil (2011) provides a key to European taxa, in which four of the five genera are included, while Parapar (2006) provides a key to boreal Atlantic species. Nilsen and Holthe (1985) provide a key to the Arctic and Scandinavian oweniid species. Capa et al. (2012b) amended the family diagnosis and provided keys to world genera and Australian species. Parapar and Moreira (2015) provide a key to *Owenia* Delle Chiaje, 1844 species in Southeast Asia and Australasian regions; Ibrahim et al. (2024) provide identification keys to species of oweniids from the South China Sea region; and Cantone and Di Pietro (1998) provide a key to Antarctic Oweniidae based on non-hook chaetae characters.

**Environment and habitat.** Aquatic, marine; coastal, continental shelf or deep sea; soft substrata.

#### ﻿Ozobranchidae (Pinto, 1921) [leech]

Fig. 85

**Common name.** Turtle leech.

**LSID.** Urn:lsid:marinespecies.org:taxname:22593.

**Figures 85, 86.**
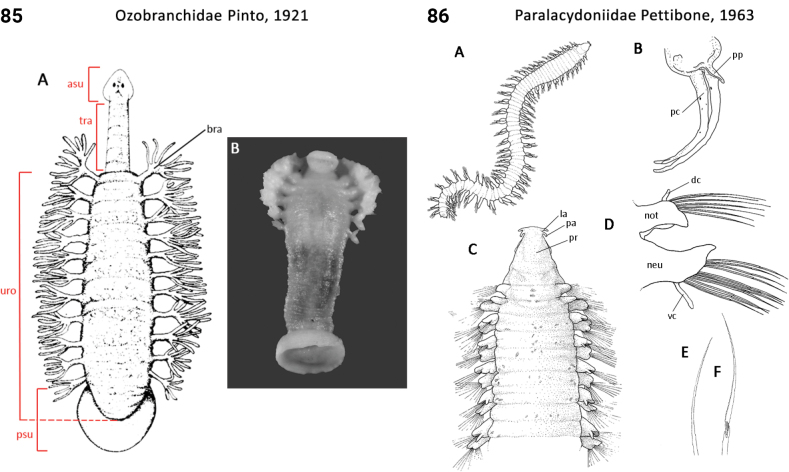
Distinguishing features: **85.**Ozobranchidae: **A.***Ozobranchusjantzeanus*, dorsal view; **B.***Ozobranchusmargoi*, dorsal view. Abbreviations: asu anterior sucker bra branchia psu posterior sucker tra trachelostome uro urostome. Sources: **A** derivative of fig. 19e Mann (1962), **B** derivative of fig. 2 Göpper et al. (2018). **86.**Paralacydoniidae: **A, C–F.**Paralacydoniacf.weberi; **A.** Entire animal, dorsal view; **B.***Paralacydoniaparadoxa*, pygidium; **C.** Anterior end dorsal view; **D.** Parapodium from chaetiger 15; **E.** Capillary from notopodium of chaetiger 15; **F.** Compound spiniger from neuropodium of chaetiger 15. Abbreviations: dc dorsal cirrus la lateral antenna neu neuropodium not notopodium pa palp pc pygidial cirrus pp pygidial papilla pr prostomium vc ventral cirrus. Sources: **A, C–F** derivatives from fig. 1.81 Beesley et al. (2000), **B** derivative of fig. 1 Rizzo and Magalhães (2022b).

**Diagnosis (Level 2).** Body regionalization present (Fig. 85A, tra, uro), midbody secondary segmentation bi-annulate or tri-annulate; anterior end sucker not clearly separated from rest of body; branchiae present (Fig. 85A, B, asu, bra).

**Description.** See Suppl. material 1.

**Remarks.**Ozobranchidae is a family of jawless leeches (Rhynchobdellida) parasitic on marine and freshwater turtles and crocodilians. The family contains two genera, *Ozobranchus* Quatrefages, 1852 (5 species) and *Bogobdella* Richardson, 1969 (1 species) (WoRMS 2025). Three marine species are known: *Ozobranchusbranchiatus* (Menzies, 1791), *O.margoi* (Apáthy, 1890) and *O.polybranchus* Sanjeeva Raj, 1951. Sawyer et al. (1975) provide a key to the marine leeches of the eastern US and Gulf of Mexico, which includes two species of Ozobranchidae. Christoffersen (2008) provides a checklist of species from South America. Burreson (2020) provides a key to species of Australia and New Zealand.

**Environment and habitat.** Marine, brackish, or freshwater; coastal or continental shelf; epizoic.

#### ﻿Paralacydoniidae Pettibone, 1963 [polychaete]

Fig. 86

**Common name.** None.

**LSID.** Urn:lsid:marinespecies.org:taxname:22611.

**Diagnosis (Level 3).** Prostomium bluntly conical; paired, lateral; antennae present; peristomium not visible; tentacular cirri absent; first segment achaetous (Fig. 86A, C); compound chaetae present (Fig. 86F); ventral cirri present (Fig. 86D).

**Description.** See Suppl. material 1.

**Remarks.**Paralacydoniidae is represented by a single genus, *Paralacydonia* Fauvel, 1913, and two species (WoRMS 2025). The family is poorly known taxonomically and its relationship within Phyllodocida is uncertain. Viéitez et al. (2004) provide a key to European species of *Paralacydonia*.

**Environment and habitat.** Aquatic, marine; coastal, continental shelf or deep sea; soft substrata.

#### ﻿Paraonidae Cerruti, 1909 [polychaete]

Fig. 87

**Common name.** None.

**LSID.** Urn:lsid:marinespecies.org:taxname:903.

**Figures 87, 88.**
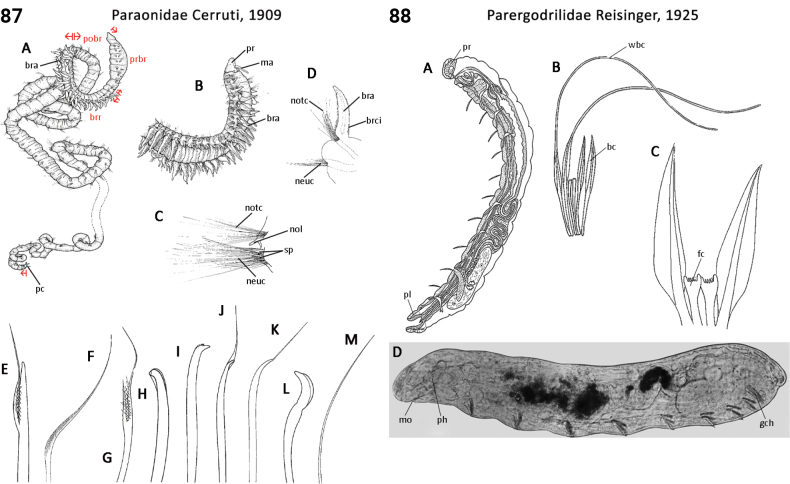
Distinguishing features: **87.**Paraonidae: **A.***Levinseniagracilis* entire animal; **B.** Anterior end of Aricideacf.fauveli ventrolateral view; **C, D.** Parapodia of Aricidea (*Acmira*) sp.: **C.** From chaetiger 12; **D.** From posterior chaetiger; **E–M.** Chaetae: **E.** Notopodial bayonet chaeta from anterior postbranchial chaetiger *Cirrophorus* sp. **F.** Neuropodial capillary from chaetiger 6 Aricidea (Allia) sp. **G.** Notopodial lyrate chaeta from posterior chaetiger *Cirrophorus* sp. **H.** Posterior neuropodial hook Aricidea sp. **I.** Neuropodial spine from posterior chaetiger Aricidea (Acmira) sp. **J.** Neuropodial spine from posterior chaetiger of Aricideacf.fauveli; **K.** Modified neurochaeta from posterior chaetiger Aricidea (Allia) sp.; **L.** Neuropodial hook from posterior chaetiger *Levinseniagracilis*; **M.** Capillary from posterior chaetiger of Aricidea (Allia) sp. Abbreviations: bra branchia brci branchial cilia brr branchial region ma median antenna neuc neurochaetae nol notopodial lobe notc notochaetae pc pygidial cirrus pobr post branchial region pr prostomium prbr prebranchial region sp spine. Sources: **A–M** after fig. 1.54, Beesley et al. (2000). **88.**Parergodrilidae: **A–C.***Stygocapitellasubterannea*: **A.** Entire animal sagittal view of a male; **B.** Chaetae from parapodium of chaetiger 1; **C.** Chaetae from parapodium of chaetiger 2; **D.***Parergodrilusheideri*, entire animal sagittal view of a male. Abbreviations: bc bilimbate chaeta, fc forked chaeta, gch genital chaetae, pr prostomium, pl pygidial lobe, wbc whip-like bilimbate chaeta. Sources: **A–C** derivatives of fig. 1.130 Beesley et al. (2000), **D** derivative of fig. 1B Purschke (2019b).

**Diagnosis (Level 3).** Body regionalization present (Fig. 87A, prbr, brr, pobr); prostomium bluntly conical (Fig. 87B); peristomium not visible; buccal tentacles absent; branchiae present (Fig. 87B, D, brr).

**Description.** See Suppl. material 1.

**Remarks.**Paraonidae comprises at least seven genera and 191 species (WoRMS 2025) but taxonomic diversity is likely to be much higher given the existence of four subgenera and widely distributed species that are likely to encompass cryptic species; the genus *Aricidea* Webster, 1879 sensu lato alone comprises ~ 100 species (WoRMS 2025). The group is particularly well represented in the deep sea. Strelzov (1979), originally published in Russian in 1973, remains the best guide to the taxonomy and morphology of the family. Gil (2011) and Parapar et al. (2012) provide keys to European taxa, Hartley (1981) provides a key to British species, and Çinar et al. (2011) provide a key to all *Levinsenia* Mesnil, 1897 species at the time.

**Environment and habitat.** Aquatic, marine; continental shelf or deep sea (rarely found in coastal waters); soft substrata.

#### ﻿Parergodrilidae Reisinger, 1925 [polychaete]

Fig. 88

**Common name.** None.

**LSID.** Urn:lsid:marinespecies.org:taxname:906.

**Diagnosis (Level 3).** Body short with a fixed number of segments, secondary annulation present (each segment comprises two rings); parapodia absent (Fig. 88A, D).

**Description.** See Suppl. material 1.

**Remarks.**Parergodrilidae comprises two genera and 12 species (WoRMS 2025), including the marine *Stygocapitellasubterranean* Knöllner, 1934, which until recently was thought to be the only species in the genus and to have a cosmopolitan distribution (Struck et al. 2017; Cerca et al. 2020). *Stygocapitella* Knöllner, 1934 now comprises 11 species and appears to have a global distribution (GBIF.org 2023). The monotypic terrestrial *Parergodrilus* Reisinger, 1925 has only been recorded from the Northern Hemisphere (mostly Europe). Its only species exhibits extreme size sexual dimorphism (males smaller) and the males also carry copulatory chaetae in the posterior end.

**Environment and habitat.** Terrestrial or aquatic, moist terrestrial (upper shore of beaches), marine (coastal); soft substrata.

#### ﻿Parvidrilidae Erséus, 1999 [microdrile]

Fig. 89

**Common name.** None.

**LSID.** Urn:lsid:marinespecies.org:taxname:1040001.

**Figures 89, 90.**
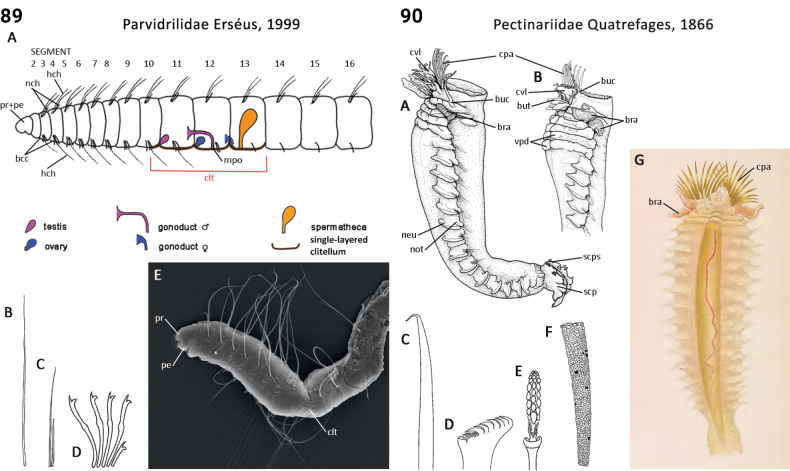
Distinguishing features: **89.**Parvidrilidae: **A.** Diagram showing location of reproductive organs, dorsal side up; **B.** Smooth hair chaetae; **C.** Dorsal bundle with a single hair and needle chaeta; **D.** Ventral bundle of bifid crotchets; **E.***Parvidrilusmeyssonnieri* anterior end. Abbreviations: bcc bifid crotchet chaeta clt single-layered clitellum hch hair chaetae mpo male pore nch needle chaetae pe peristomium pr prostomium. Sources: **A–E** derivatives of fig. 12.1, 12.2, 12.3 Thorp et al. (2019). **90.**Pectinariidae: **A.***Pectinariaantipoda* entire animal lateral view; **B–E.***Amphictenecrassa*: **B.** Anterior end, ventrolateral view; **C.** Palea; **D, E.** Uncini from chaetiger 6, lateral (**D**), frontal (**E**) view; **F.** tube of a pectinariid; **G.***Lagiskoreni* entire animal. Abbreviations: bra branchia buc buccal cirri but buccal tentacles cpa cephalic palea cvl cephalic veil neu neuropodium not notopodium scp scaphe scps scaphal spine vpd ventral pads. Sources: **A–F** derivatives of fig. 1.117 Beesley et al. (2000), **G** derivative of MacIntosh (1900–1922), pl. CXII, fig. 2.

**Diagnosis (Level 2).** Chaetae present, first appear on second segment after peristomium (= S3, Fig. 89A); hair chaetae present, smooth, dorsal (and ventral) ones may be very long (Fig. 89B, C, E); needle chaetae present (Fig. 89A, C, nch); male pores single, median (Fig. 89A); spermathecal pores post-testicular.

**Description.** See Suppl. material 1.

**Remarks.**Parvidrilidae comprises a single genus and eight species described from groundwater of North America and Europe (WoRMS 2025). Parvidrilidae (parvus = small) include some of the smallest known oligochaetes and as such, features that are normally useful for recognizing oligochaete families and discerning species, such as the location of spermathecal and male pores, are extremely difficult to determine; even identifying them as ‘spermathecal’ and ‘male’ pores remains speculative. The combination of tiny size and the presence of hair chaetae in the ventral (as well as the more usual dorsal) position on segments, with both dorsal and ventral chaetae beginning on segment III (Fig. 89A, hch) are better identifying criteria for the family.

**Environment and habitat.** Aquatic (including subterranean or hyporheic), freshwater; soft substrata.

#### ﻿Pectinariidae Quatrefages, 1866 [polychaete]

Fig. 90

**Common name.** Ice-cream cone worms.

**LSID.** Urn:lsid:marinespecies.org:taxname:980.

**Diagnosis (Level 3).** Body with short caudal region with few segments, mostly chaetous, and has frilly lobes (Fig. 90A, G); secondary annulation present; paleate chaetae present (Fig. 90A–C, G; cpa); pygidium as an anal flap or ligule.

**Description.** See Suppl. material 1.

**Remarks.**Pectinariidae comprises five genera and 73 species (WoRMS 2025), although in the past only one (or two) genera were accepted, with *Pectinaria* Lamarck, 1818 comprising subgenera. The family has a global distribution. Hutchings and Peart (2002) provide a key to Australian genera and species, which was updated by Zhang and Hutchings (2019). Gil (2011) provides a key to European taxa, which includes all five genera, and Parapar et al. (2020b) provides a key to NE Atlantic taxa. Jirkov and Leontovich (2013) provide a key to Terebellomorpha, including Pectinariidae, from the eastern Atlantic and the North Polar seas.

**Environment and habitat.** Aquatic, marine; coastal, continental shelf or deep sea; soft substrata.

#### ﻿Phreodrilidae Beddard, 1891 [microdrile]

Fig. 91

**Common name.** None.

**LSID.** Urn:lsid:marinespecies.org:taxname:390845.

**Figures 91, 92.**
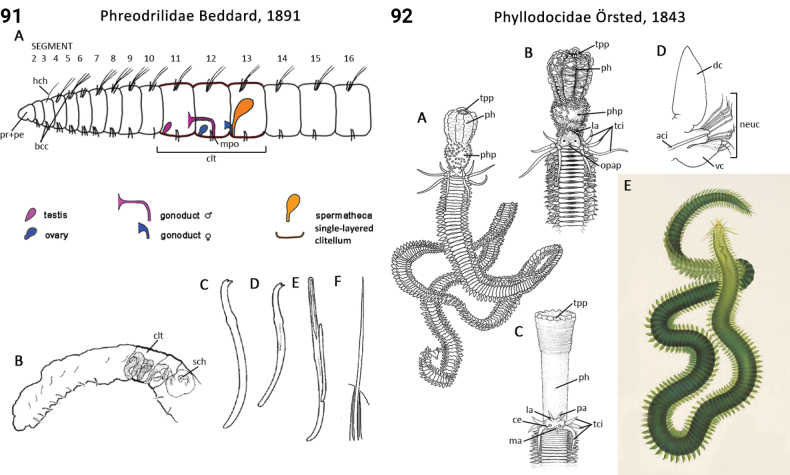
Distinguishing features: **91.**Phreodrilidae: **A.** Diagram showing location of reproductive organs, dorsal side up; **B–E.***Astacopsidrilusryuteki*: **B.** Forebody of sexually mature specimen with clitellum; **C.** Ventral bifid crotchet chaeta; **D.** Dorsal bifid crotchet chaeta; **E.** Spermathecal chaeta; **F.** Unknown phreodrilid, dorsal chaeta. Abbreviations: bcc bifid crotchet chaeta clt single-layered clitellum hch hair chaetae mpo male pore pe peristomium pr prostomium sch spermathecal chaeta. Sources: **A–E** derivatives of fig. 12.22, 12.3 Thorp et al. (2019), **F** derivative of Timm (2009, 137). **92.**Phyllodocidae: **A, B.***Phyllodoce* sp.: **A.** Entire animal with pharynx everted dorsal view; **B.** Anterior end with pharynx everted dorsal view. **C.** Anterior end of *Eumidafuscolutata* with pharynx everted dorsal view; **D.** Median parapodium of *Phyllodoce* sp.; **E.***Eulaliaviridis* entire animal. Abbreviations: aci acicula ce compound eye dc dorsal cirrus la lateral antenna ma median antenna neuc neurochaetae opap occipital papilla pa palp ph pharynx php pharyngeal papilla tci tentacular cirri tpp terminal pharyngeal papillae vc ventral cirrus. Sources: **A–D** derivatives of fig. 1.83 Beesley et al. (2000), **E** derivative of MacIntosh (1900–1922), pl. XLIV, fig. 1.

**Diagnosis (Level 0).** Chaetae present, including crotchets (Fig. 91F) ; nephridial pores located anteriorly; gonoducts located around clitellum (Fig. 91A); pygidial (caudal) appendages absent; spermathecae post-testicular.

**Description.** See Suppl. material 1.

**Remarks.** Using our dataset, Phreodrilidae is not diagnosable (DELTA Diagnostic Level 0) from Naididae. The reader is referred to the full description to verify identification. This is somewhat at odds with the opinion of Brinkhurst and Jamieson (1971) who consider Phreodrilidae separable from Naididae, Opistocystinae by the presence of three caudal processes (Opistocystinae) vs none (Phreodrilidae) and > 2 crotchet chaetae per bundle ventrally (Opistocystinae) vs two crotchet chaetae per bundle (Phreodrilidae), and the dorsal bundle with hair chaetae only (Opistocystinae) vs hair chaetae and needles (Phreodrilidae). However, Erséus et al. (2010a) later found that Opistocystinae were variable in the dorsal bundle and usually have both hairs and needles. Considering this, and the present findings, the family is in need of phylogenetic revision. ﻿Phreodrilidae comprises seven genera and 53 species (WoRMS 2025) and mostly occur in the southern hemisphere (Australia, New Zealand, Africa, South America, Sri Lanka, and Southern Ocean islands); they are rarely found and suspected to be introduced to Europe and Japan (Timm 2009; Pinder 2013). Australia has the highest diversity with all seven genera present and 32 named species, although the actual species diversity is likely to be at least double that (Pinder 2013). Pinder (in press) provides a key to Australian species. Three species have been reported from marine/estuarine environments (Pinder and Erséus 2000).

**Environment and habitat.** Terrestrial (rarely) or aquatic (including epigean and subterranean waters); freshwater, brackish, or marine (rarely); soft substrata or epizoic (rarely ectocommensal on crayfish).

#### ﻿Phyllodocidae Örsted, 1843 sensu lato, excluding Alciopini [polychaete]

Fig. 92

**Common name.** Paddle worms.

**LSID.** Urn:lsid:marinespecies.org:taxname:931.

**Diagnosis (Level 3).** In life, body opaque, gut usually not visible (Fig. 92E); prostomium bluntly conical, one pair of eyes (Fig. 92B, C); palps present; peristomium not visible; tentacular cirri present; pharynx without jaws (Fig. 92A–C; tpp); parapodia with dorsal cirri flattened and foliaceous and notopodial lobes absent (Fig. 92D); compound chaetae with shaft distally inflated near joint (Fig. 92D).

**Description.** See Suppl. material 1.

**Remarks.**Phyllodocidae has a wide range of forms among Phyllodocida; hence we have provided separate coding for the benthic Phyllodocidae sensu lato and the holopelagic tribe Alciopini, which follows the classification used in WoRMS (Glasby and Fauchald 2003). Note that the holopelagic Iospilidae, which is considered a junior synonym of Phyllodocidae by Rouse et al. (2022), is maintained as a valid family in this dataset, following WoRMS (2025). Phyllodocidae (excluding Alciopini) includes 21 genera and 461 species (WoRMS 2025), and is globally distributed. Pleijel and Dales (1991), Viéitez et al. (2004), and Gil (2011) provide keys to European taxa, which includes most of the genera including the European alciopines. Oliveira et al. (2021) provide a key to species of *Phyllodoce* occurring in Brazil. Salazar-Vallejo (2022) provides keys to world species of *Anaitides* Czerniavsky, 1882 (now accepted as *Phyllodoce* Lamarck, 1818), *Nereiphylla* Blainville, 1828, and *Pterocirrus* Claparède, 1868.

**Environment and habitat.** Aquatic, marine; coastal, continental shelf or deep sea; soft or hard substrata.

#### ﻿Phyllodocidae, Eteoninae, Alciopini Ehlers, 1864 [polychaete]

Fig. 93

**Common name.** Pelagic paddle worms.

**LSID.** Urn:lsid:marinespecies.org:taxname:932.

**Figures 93, 94.**
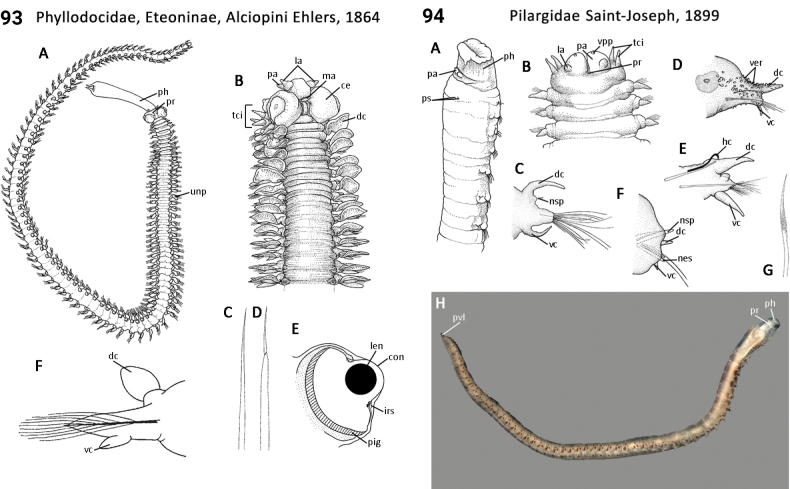
Distinguishing features: **93.**Alciopini: **A.***Torreacandida* entire animal with pharynx everted dorsal view; **B–D.** An undescribed Australian species of *Torrea*: **B.** Anterior end dorsal view; **C.** Simple chaeta; **D.** Compound chaeta. **E.** Cross-section of eye of *Torreacandida*; **F.***Vanadislongissima* uniramous parapodium of anterior body. Abbreviations: ce compound eye con cornea dc dorsal cirrus irs iris la lateral antenna len lens ma medianantenna pa palp ph pharynx pig pigment layer pr prostomium tci tentacular cirri unp uniramous parapodium vc ventral cirrus. Sources: **A–E** after fig. 1.69 Beesley et al. (2000), **F** after fig. 22 Pettibone (1963). **94.**Pilargidae: **A, G.***Hermundura* sp.; **A.** Anterior end with pharynx everted dorsolateral view; **B.***Pilargis* sp. dorsal view of anterior end; **C–F.** Parapodial morphology: **C.***Glyphohesioneklatti*; **D.***Pilargisverrucosa*; **E.***Sigambraparva*; **F.***Litocorsastremma*; **G.** Capillary neurochaeta from parapodium of chaetiger 45; **H.***Hermunduragladstonensis* entire animal, lateral view. Abbreviations: dc dorsal cirrus hc hook chaeta la lateral antenna nes neuropodial spine nsp notopodial spine pa palp ph pharynx pr prostomium ps pigment spot pvl pygidial ventral lobe tci tentacular cirri vc ventral cirrus ver verruca vpp ventrolateral palpal papilla. Sources: **A, B, G** derivatives of fig. 1.84 Beesley et al. (2000), **C–F, H** derivatives of fig. 13 Glasby and Salazar-Vallejo (2022).

**Diagnosis (Level 3).** In life, body translucent, gut visible (Fig. 93A); prostomium rounded to oval, bearing a pair of enlarged eyes (Fig. 93B, E); compound chaetae with shaft tapering slightly, or evenly thick from emergence to joint (Fig. 93D); holopelagic.

**Description.** See Suppl. material 1.

**Remarks.**Alciopini was treated as a family (Alciopidae) in POLiKEY (Glasby and Fauchald 2003). It has been downgraded to tribe based on Halanych et al. (2007) and Rouse et al. (2022) as presented in WoRMS (2025). Alciopini is a holopelagic group nested within the benthic Phyllodocidae-Eteoninae; this placement is supported by both morphological and molecular data (Rouse et al. 2022, and references therein). The tribe includes 11 genera and 46 species (WoRMS 2025), and has a global distribution, although records at high latitudes are rare (GBIF.org 2023). Dales (1957) provides keys to genera and species of Alciopini of the Pacific Ocean; Gil (2011) includes the European alciopines in his key to Phyllodocidae. Jiménez-Cueto and Suárez-Morales (2008) provide a key to species of *Alciopina* Claparède & Panceri, 1867, *Torrea* Quatrefages, 1850 and *Rhynchonereella* Costa, 1864 from the Caribbean. O’Sullivan (1982) provides a key to Southern Ocean taxa.

**Environment and habitat.** Aquatic, marine; coastal, continental shelf, or deep sea; holopelagic (upper and mid-water levels).

#### ﻿Pilargidae Saint-Joseph, 1899 [polychaete]

Fig. 94

**Common name.** None.

**LSID.** Urn:lsid:marinespecies.org:taxname:15009.

**Diagnosis (Level 3).** In life, body opaque, gut usually not visible (Fig. 94H); pharynx lacking jaws; tentacular cirri present (Fig. 94A, B); second segment chaetous; first chaetiger with parapodia more-or-less laterally directed and free from head, with neurochaetae only; thereafter parapodia biramous (Fig. 94C–F); compound chaetae absent; notopodial lobes represented by at least one chaetal lobe (Fig. 94C–F).

**Description.** See Suppl. material 1.

**Remarks.**Pilargidae was suspected of including Antonbruunidae for many years on the basis of shared key morphological features (Salazar-Vallejo 1986; Glasby 1993). Molecular data have confirmed this placement (Mackie et al. 2015; Huč et al. 2024). The family includes ten genera in two subfamilies, Pilarginae (*Ancistrosyllis* McIntosh, 1878, *Cabira* Webster, 1879, *Pilargis* Saint-Joseph, 1899, *Sigambra* Müller, 1858) and Synelminae (*Antonbruunia* Hartman & Boss, 1966; *Glyphohesione* Friedrich, 1950, *Litocorsa* Pearson, 1970, *Otopsis*, *Pseudexogone* Augener, 1922, *Synelmis* Chamberlin, 1919), plus the unplaced *Sigatargus* Misra, 1999, and 125 species (WoRMS 2025), and has a global distribution. Viéitez et al. (2004) and Gil (2011) provide keys to European taxa, Salazar‐Vallejo and Harris (2006) provide a key to world *Pilargis* species, Salazar-Vallejo et al. (2019b) provide keys to species of *Sigambra*, and Plathong et al. (2021a) provide world keys to *Cabira* and *Ancistrosyllis*.

**Environment and habitat.** Aquatic, marine; coastal, continental shelf, or deep sea; soft substrata or endozoic (*Antonbruunia* is an endosymbiont of bivalves).

#### ﻿Piscicolidae (Johnston, 1865) [leech]

Fig. 95

**Common name.** Fish leech.

**LSID.** Urn:lsid:marinespecies.org:taxname:2043.

**Figures 95, 96.**
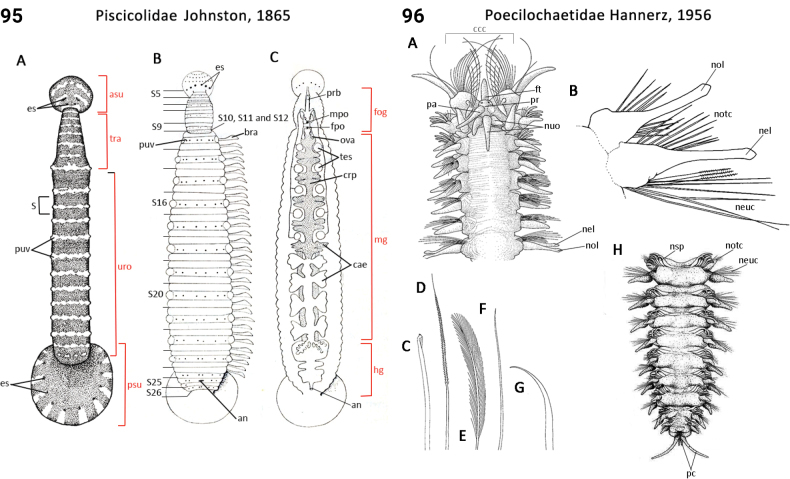
Distinguishing features: **95.**Piscicolidae: **A.***Cystobranchusfasciatus*, a fish leech dorsal view; **B, C.***Branchelliontorpedinis*: **B.** Dorsal view (branchia removed on left side); **C.** Diagram of gut and reproductive system. Abbreviations: an anus asu anterior sucker bra branchia cae caecum crp crop es eye spot fog foregut fpo female pore hg hind gut mg mid gut mpo male ova ovary prb proboscis pore psu posterior sucker puv pulsatile vescicle S segment tes testis tra trachelstome uro urostome. Sources: **A** derivative of fig. 1 Sket and Trontelj (2008), **B, C** derivative of fig. 18 Mann (1962). **96.**Poecilochaetidae: **A–G.***Poecilochaetus* sp.: **A.** Anterior end dorsal view; **B.** Median parapodium posterior view; **C–G.** Chaetae from parapodium of chaetiger 12: **C.** Falcate spine; **D.** Spinose capillary; **E.** Plumose capillary; **F.** Slender smooth capillary; **G.** Spine from parapodium of chaetiger 40; **H.***P.serpens* posterior end dorsal view. Abbreviations: ccc cephalic cage chaetae ft facial tubercle nel neuropodial lobe neuc neurochaetae nuo nuchal organ nol notopodial lobe notc notochaetae nsp notopodial spine pa palp pc pygidial cirrus pr prostomium. Sources: **A–G** derivatives of fig. 1.107 Beesley et al. (2000), **H** derivative of fig. 7.4.2.1 Blake and Maciolek (2019b).

**Diagnosis (Level 2).** Body regionalization present (Fig. 95A, tra, uro); mid-body secondary annulation, 4–7-annulate; anterior end sucker clearly separated from rest of body (Fig. 95A); proboscis present (Fig. 95C, prb).

**Description.** See Suppl. material 1.

**Remarks.**Piscicolidae is a large family of jawless leeches (Rhynchobdellida). While the family has a worldwide distribution, comprising 63 genera and 156 species (WoRMS 2025), freshwater taxa are mostly limited to the Palearctic and Nearctic (Sket and Trontelj 2008), and marine members have a global distribution. Sawyer et al. (1975) provide a key to the marine leeches of the eastern US and Gulf of Mexico, which include mostly Piscicolidae species. Christoffersen (2008) provides a checklist of species from South America. Burreson (2020) provides a key to marine and estuarine species of Australia and New Zealand.

**Environment and habitat.** Aquatic, marine, brackish, or freshwater; coastal, continental shelf, or deep sea; epizoic.

#### ﻿Poecilochaetidae Hannerz, 1956 [polychaete]

Fig. 96

**Common name.** None.

**LSID.** Urn:lsid:marinespecies.org:taxname:916.

**Diagnosis (Level 3).** Prostomial antennae present; facial tubercle present (Fig. 96A, ft); notopodial and neuropodial lobes slender, flask- or spindle-shaped (Fig. 96B); pygidial appendages include three cirri or four cirri (Fig. 96H); tube present.

**Description.** See Suppl. material 1.

**Remarks.**Poecilochaetidae is maintained here as a family-level taxon following POLiKEY and WoRMS, contra Rouse et al. (2022) who consider them as part of Spionidae. Their position within Spionidae is supported by both morphological (including reproductive and larval characters) and molecular evidence (Rouse et al. 2022 and references therein). Morphological studies not supporting an expanded concept of Spionidae seem to have been unduly influenced by using outgroups that are not sufficiently distant from the ingroup (Rouse et al. 2022). The family includes a single genus and 33 species (WoRMS 2025), and has a global distribution. Mackie (1990) provides a key to groups of species of Hong Kong, while Gil (2011) provides a key to European taxa.

**Environment and habitat.** Aquatic, marine; coastal, continental shelf, or deep sea; soft substrata.

#### ﻿Polygordiidae Czerniavsky, 1881 [polychaete]

Fig. 97

**Common name.** Knot worms.

**LSID.** Urn:lsid:marinespecies.org:taxname:993.

**Figures 97, 98.**
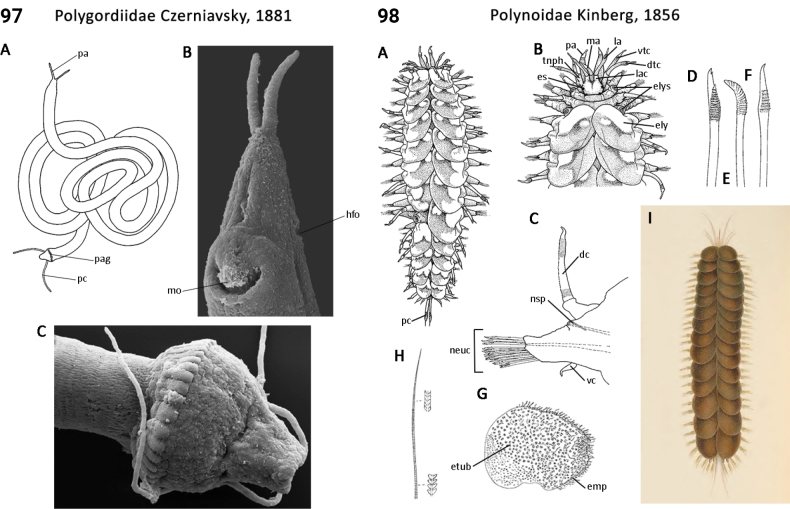
Distinguishing features: **97.**Polygordiidae: **A.***Polygordiusappendiculatus*, entire animal, dorsal view; **B.** Anterior end of *Polygordiusjouinae*; **C.** Posterior end of *Polygordius* sp. Abbreviations: hfo head fold pa palp pc pygidial cirrus pag pygidial adhesive glands mo mouth. Sources: **A** derivative of fig. 1.125 Beesley et al. (2000), **B, C** derivatives of fig. 7.5.1 and fig. 7.5.4 Ramey-Balci et al. (2021). **98.**Polynoidae: **A–E.***Lepidonotusmelanogrammus*: **A.** Entire animal dorsal view; **B.** Anterior end dorsal view first and second elytral pairs removed; **C.** Anterior view parapodium from chaetiger 11; **D.** Bidentate neurochaeta from chaetiger 11; **E.** Notochaeta from chaetiger 11; **F.** Unidentate neurochaeta from chaetiger 13 *Gastrolepidiaclavigera*; **G.** Elytron from midbody of *Harmothoecharlottae*, *H.notochaeta*, with detail of part; **I.***Gattyanacirrhosa*, entire animal. Abbreviations: dc dorsal cirrus dtc dorsal tentacular cirri es eye spot ely elytron elys elytral scar emp elytra marginal papilla etub elytra tubercle la lateral antenna lac lateral antenna ceratophore ma median antenna neuc neurochaetae nsp notopodial spine pa palp pc pygidial cirrus tnph tentaculophore vc ventral cirrus vtc ventral tentacular cirri. Sources: **A–G** derivatives of fig. 1.86, Beesley et al. (2000), **H** derivative of fig. 7 Pettibone (1986), **I** derivative of MacIntosh (1900–1922), pl. XXV, fig. 3.

**Diagnosis (Level 2).** Paired frontal palps present (Fig. 97A, B); parapodia and chaetae absent; ventral groove present; pygidium as a simple lobe although subterminally inflated and appearing bulb-shaped (bulb adorned with a ring of papilla-sized adhesive glands); paired pygidial cirri present (Fig. 97A, C).

**Description.** See Suppl. material 1.

**Remarks.**Polygordiidae is represented by a single currently accepted genus, *Polygordius* Schneider, 1868, and 21 species (WoRMS June 2025). Polygordiidae are slender, nematode-like interstitial polychaetes. The family is only distinguishable from Protodriloididae at DELTA Level 2; phylogenomic evidence indicates it forms a clade together with Protodrilidae, Protodriloididae, and Saccocirridae (Martínez et al. 2021a, and references therein). Polygordiidae has a global distribution on coasts having coarse, sandy sediments. Gil (2011) and Parapar et al. (2018) provide keys to European species of *Polygordius*.

**Environment and habitat.** Aquatic, marine; coastal, continental shelf, littoral, or supralittoral; soft substrata (coarse sand, particularly surf and swash zones).

#### ﻿Polynoidae Kinberg, 1856 [polychaete]

Fig. 98

**Common name.** Scaleworms.

**LSID.** Urn:lsid:marinespecies.org:taxname:939.

**Diagnosis (Level 1).** Prostomium rounded to oval, anteriorly incised (Fig. 98B); dorsal body surface with protective covering of scales (elytrae); elytrae covered with papillae, tubercles or smooth (Fig. 98A, B, G, I).

**Description.** See Suppl. material 1.

**Remarks.**Polynoidae is one of the most taxon-rich families of Polychaeta, with 173 genera and 870 species (WoRMS 2025), and has a global distribution. In the present dataset, it is only distinguishable from Iphionidae at DELTA Diagnostic Level 1. Polynoidae is divided into 21 subfamilies (Wehe 2006), but no attempt has been made to code them here as revisionary work is currently in review (RSW and A. Murray, pers. comm., Jan. 2025), and concepts are likely to change. Barnich and Fiege (2003) provide keys to Mediterranean taxa, and Gil (2011) and Parapar et al. (2015) provide keys to European subfamilies, genera, and species. Barnich et al. (2004) provide a key to Polynoidae of the South China Sea. Barnich (2011) provides a key to scale worms, including Polynoidae, of British and Irish waters, while Barnich et al. (2019) emended the definition of *Malmgrenia* McIntosh, 1874 and provide an updated key it its species from Mediterranean and the North Atlantic. Hanley and Burke (1991) provide keys to the polynoids of Coral Sea, NE Australia. Bonifácio and Menot (2019) provide a taxonomic key for genera of the subfamily Macellicephalinae.

**Environment and habitat.** Aquatic, marine; coastal, continental shelf, or deep sea; soft or hard substrata, hydrothermal vents and cold seeps, holopelagic, epizoic, or sunken bones of vertebrates.

#### ﻿Pontodoridae Bergström, 1914 [polychaete]

Fig. 99

**Common name.** None.

**LSID.** Urn:lsid:marinespecies.org:taxname:934.

**Figures 99, 100.**
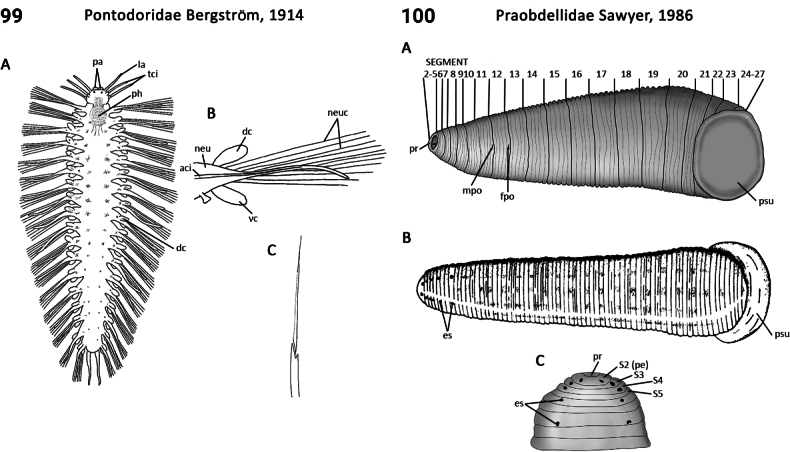
Distinguishing features: **99.**Pontodoridae: **A–C.***Pontodorapelagica*: **A.** Entire animal dorsal view; **B.** Parapodium; **C.** Compound neurochaeta. Abbreviations: aci acicula dc dorsal cirrus la lateral antenna neu neuropodium neuc neurochaetae pa palp ph pharynx tci tentacular cirri vc ventral cirrus. Sources: **A–C** derivatives of fig. 1.87 Beesley et al. (2000). **100.**Praobdellidae: **A.** Schematic diagram showing whole body ventral view; **B.***Praobdellabuettneri* entire animal, dorsal view; **C.** Eyespot arrangement illustrated dorsally. Abbreviations: es eye spot fpo female pore mpo male pore pe peristomium pr prostomium psu posterior sucker S segment. Sources: **A, C** derivative of fig. 3 Phillips et al. (2010), **B** derivative of fig. 21 Mann (1962).

**Diagnosis (Level 3).** Body more-or-less cylindrical; in life, translucent, gut visible; prostomium rounded to oval, antennae present, paired, lateral (Fig. 99A, la); tentacular cirri present, 2 pairs; proboscis with subterminal papillae; pharynx proventricle present (Fig. 99A, ph); dorsal cirri more-or-less cirriform (Fig. 99B, dc); capillary chaetae absent; pygidial appendages present, one pair of cirri (Fig. 99A).

**Description.** See Suppl. material 1.

**Remarks.**Pontodoridae is represented by a single genus, *Pontodora* Greeff, 1879, and a single species, *P.pelagica* Greeff, 1879, a holopelagic species distributed around the world. The family is currently considered incertae sedis within Phyllodocida (Rouse et al. 2022).

**Environment and habitat.** Aquatic, marine; coastal, continental shelf, or deep sea; holopelagic.

#### ﻿Praobdellidae Sawyer, 1986 [leech]

Fig. 100

**Common name.** None.

**LSID.** Urn:lsid:marinespecies.org:taxname:1324745.

**Diagnosis (Level 1).** Dorsoventrally flattened, mid-body secondary annulation, 5-annulate (Fig. 100A, B); eyes on head present (Fig. 100B, C); jaw apparatus includes cutting plates or paired fine teeth or a series of soft teeth.

**Description.** See Suppl. material 1.

**Remarks.**Praobdellidae is a widespread family belonging to the jawed Hirudiniformes which includes both blood-feeding and invertebrate predatory leeches. Although diagnosable at DELTA Diagnostic Level 1, it is not separable from Hirudinidae and Cyclobdellidae at Level 2. The family was originally thought to contain specialized mucous-membrane feeders of mammals including those of humans, until Nakano et al. (2017) expanded the host list to include invertebrates after finding a species feeding on a Japanese freshwater crab. Phillips et al. (2010) expanded the taxonomic content of the family to include *Tyrannobdella*Phillips et al., 2010, *Dinobdella* Moore, 1927, *Myxobdella* Oka, 1917, *Praobdella* Blanchard, 1896 and *Pintobdella* Caballero, 1937 (along with *Limnobdella* Blanchard, 1893 and *Limnatis* Moquin-Tandon, 1827).

**Environment and habitat.** Aquatic, freshwater; epizoic or endozoic.

#### ﻿Propappidae Coates, 1986 [microdrile]

Fig. 101

**Common name.** None.

**LSID.** Urn:lsid:marinespecies.org:taxname:468020.

**Figures 101, 102.**
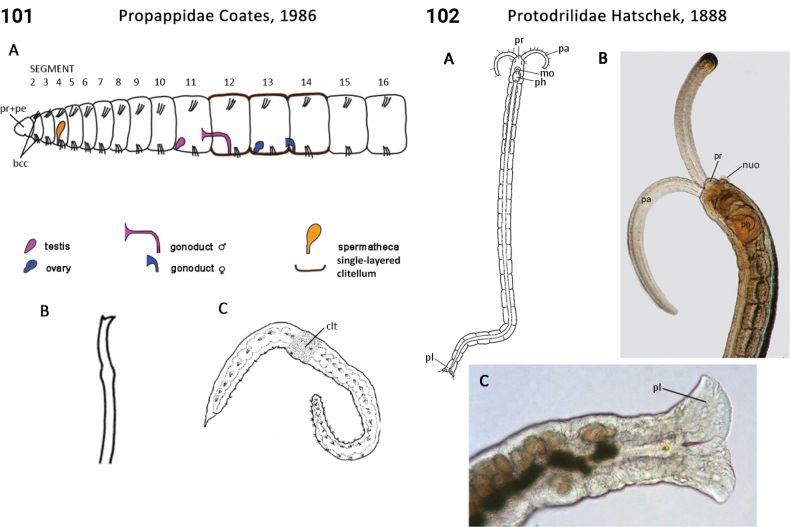
Distinguishing features: **101.**Propappidae: **A.** Diagram showing location of reproductive organs, dorsal side up; **B.** Bifid crotchet; **C.***Propappusvolki*, general view of sexually mature specimen with clitellum. Abbreviations: bcc bifid crotchet chaeta clt single-layered clitellum pe peristomium pr prostomium. Sources: **A, B** derivatives of fig. 12.3, 12.22 of Thorp et al. (2019), **C** derivative of Timm (2009, 137). **102.**Protodrilidae: **A.** Entire animal of *Protodrilusadhaerens* dorsal view; **B.** Anterior end of *Protodrilussmithsoni*; **C.** Pygidium of *Lindrilusrubropharyngeus*. Abbreviations: mo mouth no nuchal organ pa palp ph pharynx pl pygidial lobe pr prostomium. Sources: **A** after fig. 1.126 Beesley et al. (2000), **B, C** after fig. 10 and fig. 14 Martínez et al. (2021a).

**Diagnosis (Level 1).** Chaetae > 2 per bundle (Fig. 101A, B), arranged in widely spaced lateral and ventrolateral pairs (lumbricine arrangement; Fig. 101C); testes, one pair; spermathecal pores located well anterior to male and female (gonadal) pores, in segment III–V (Fig. 101A).

**Description.** See Suppl. material 1.

**Remarks.**Propappidae, a monogeneric family with three species (WoRMS 2025) thought to be restricted to the Palaearctic region, is now known from North America where it is considered a probable introduction (Martin et al. 2008). The family is similar to Enchytraeidae and only distinguishable from it at DELTA Diagnostic Level 1.

**Environment and habitat.** Aquatic, freshwater; soft substrata.

#### ﻿Protodrilidae Hatschek, 1888 [polychaete]

Fig. 102

**Common name.** None.

**LSID.** Urn:lsid:marinespecies.org:taxname:994.

**Diagnosis (Level 2).** Elongate body, equal in width along its length (Fig. 102A); palps present, anteroventral (Fig. 102A, B); first and second segments achaetous (as are all following segments); ventral groove present; pygidium bilobed (Fig. 102C).

**Description.** See Suppl. material 1.

**Remarks.**Protodrilidae, together with Protodriloididae and Saccocirrdae, are small interstitial polychaetes constituting Protodrilida (Rouse et al. 2022; Table 1). The family is only distinguishable from Protodriloididae at DELTA Level 2. Protodrilidae includes six genera and 39 species (WoRMS 2025). It has a global distribution in marine coastal sands at low to mid-latitudes; it appears to be uncommon at high southern latitudes (GBIF.org 2023). Gil (2011) and Parapar et al. (2018) provide keys to European *Protodrilus* Hatschek, 1881.

**Environment and habitat.** Aquatic, marine (very rarely freshwater); coastal, littoral or supralittoral; soft substrata (often coarse sand on high energy coasts), or holopelagic (very rarely).

#### ﻿Protodriloididae Purschke & Jouin, 1988 [polychaete]

Fig. 103

**Common name.** None.

**LSID.** Urn:lsid:marinespecies.org:taxname:155531.

**Figures 103, 104.**
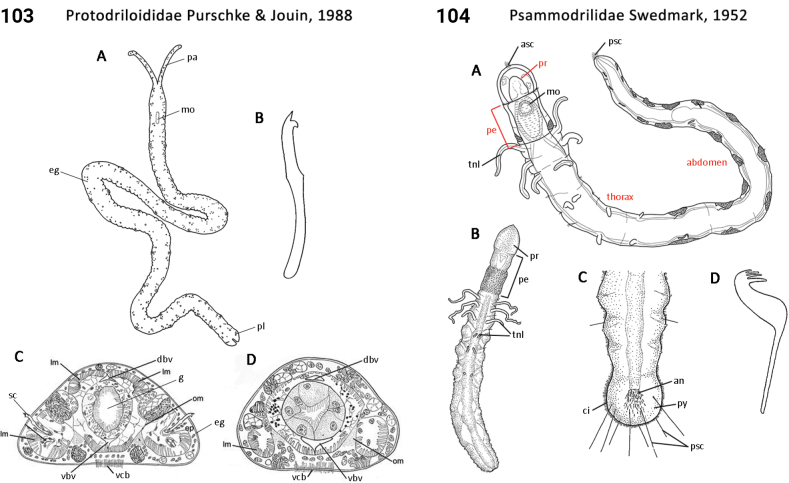
Distinguishing features: **103.**Protodriloididae: **A.***Protodriloidessymbioticus*, entire animal, dorsal view; **B.** Bidentate sigmoid chaeta; **C, D.** Transverse section of trunk of *Protodriloides* sp. Abbreviations: dbv dorsal blood vessel eg epidermal gland g gut lm longitudinal muscle mo mouth om oblique muscle pa palp sc sigmoid chaeta vbv ventral blood vessel. Sources: **A, B** derivatives of fig. 1.127 Beesley et al. (2000), **C, D** derivatives of fig. 2 Martínez (2021b). **104.**Psammodrilidae: **A, B.** Two different species of *Psammodrilus*, entire animal, dorsal view; **C.** Pygidium dorsal view; **D.** Uncinus. Abbreviations: an anus asc anterior sensory cilia ci cilia mo mouth opening pe peristomium pr prostomium psc posterior sensory cilia py pygidium tnl thoracic notopodial lobes. Sources: **A** derivative of fig. 5.2.1 Worsaae (2019). **B–D** derivatives of fig. 1.131 Beesley et al. (2000).

**Diagnosis (Level 2).** Palps present, frontal (Fig. 103A); first segment achaetous; ventral groove present; pygidium bilobed (Fig. 103A).

**Description.** See Suppl. material 1.

**Remarks.**Protodriloididae are small interstitial polychaetes which, together with the more diverse Protodrilidae, and Saccocirridae, form a well-supported group, Protodrilida (Rouse et al. 2022; Table 1). The family is only distinguishable from Polygordiidae and Protodrilidae at DELTA Level 2. Protodriloididae superficially resembles Protodrilidae and Saccocirridae due to the presence of paired frontal palps and a bilobed adhesive pygidium (Martínez et al. 2021b, and references therein). Protodriloididae is represented by a single genus, *Protodriloides* Jouin, 1966, and two species (WoRMS 2025). GBIF records are widespread but very patchy with some continents having one or no records (GBIF.org 2023). Gil (2011) and Parapar et al. (2018) provide keys to European *Protodriloides*.

**Environment and habitat.** Aquatic, marine; coastal, littoral or supralittoral (e.g., associated with groundwater discharge); soft substrata.

#### ﻿Psammodrilidae Swedmark, 1952 [polychaete]

Fig. 104

**Common name.** None.

**LSID.** Urn:lsid:marinespecies.org:taxname:11787.

**Diagnosis (Level 3).** In life, body translucent, gut visible; body regionalized comprising a thorax and abdomen (Fig. 104A, B); discrete head present, lobe-like without appendages; peristomium as a double ring (Fig. 104A, B); distinct ventral or axial pharynx absent (in place is a unique muscularised diaphragm in second peristomial ring; Fig. 104B, pe); neuropodial lobes as low ridges bearing uncini, capillary chaetae absent, aciculae present (Fig. 104A, D).

**Description.** See Suppl. material 1.

**Remarks.**Psammodrilidae is represented by a single genus, *Psammodrilus* Swedmark, 1952, and eight species (WoRMS 2025). GBIF records are widespread but very patchy with some continents having one or no records (GBIF.org 2023). Three of its eight species are included in a key to European *Psammodrilus* by Gil (2011).

**Environment and habitat.** Aquatic, marine; coastal; soft substrata (sandy-gravel).

#### ﻿Randiellidae Erséus & Strehlow, 1986 [microdrile]

Fig. 105

**Common name.** None.

**LSID.** Urn:lsid:marinespecies.org:taxname:475487.

**Figures 105, 106.**
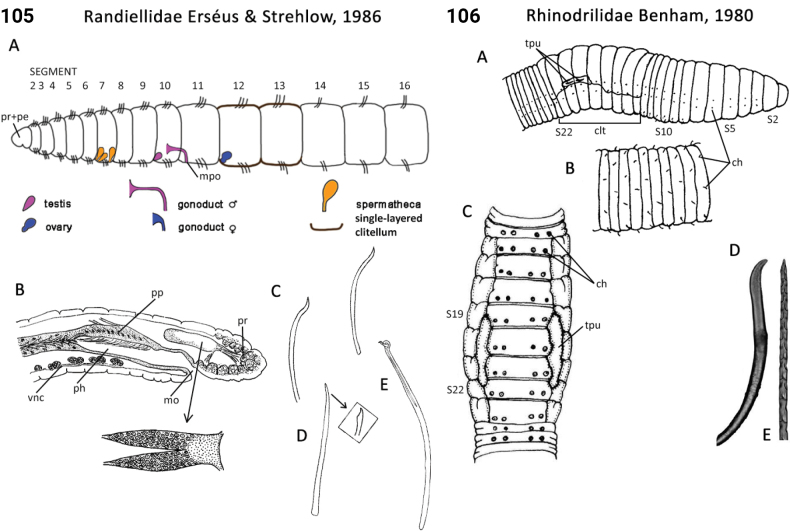
Distinguishing features of annelid families. **105.**Randiellidae: **A.** Diagram of reproductive organs, dorsal side up; **B.***Randiellamultitheca* anterior end lateral view, *Randiella* sp. brain, dorsal view; **C.** Somatic chaeta; **D.** Modified chaeta of segment 12 of *R.multitheca*; **E.** Genital chaeta from segment 10 of *R.litoralis*. Abbreviations: mo mouth mpo male pore pe peristomium ph pharynx pp pharyngeal pad pr prostomium S segment vnc ventral nerve cord. Sources: **A** derivative of fig. 1 Erséus (2005), **B–E** derivatives of figs 1, 2, 4 Erséus and Strehlow (1986). **106.**Rhinodrilidae: *Pontoscolexcorethrurus*; **A.** Head end, lateral view; **B.** Tail portion. **C.***Pontoscolexbora* clitellar region, ventral view. Andriorrhinus (Turedrillus) yukuna; **D.** Common chaetae; **E.** Genital chaetae, distal part. Abbreviations: ch chaeta, clt clitellum, S segment, tpu tubercula pubertatis. Sources: **A, B** derivative of fig. 3 James (2004), **C** derivative of fig. 8B, **D, E**, fig. 5A Feijoo and Celis (2012).

**Diagnosis (Level 3).** Prostomium not demarcated posteriorly (zygolobic); chaetae present from first segment after peristomium (= S2), crotchets, two or more per bundle (Fig. 105A), arranged in closely spaced lateral and ventrolateral pairs (lumbricine arrangement); hair chaetae absent; clitellum situated posterior to male pore (Fig. 105A); male pores in same segment as corresponding testes (prosoporous) or in segment following them (plesioporous); spermathecal pores located well anterior to male and female pores (Fig. 105A); female gonoducts absent.

**Description.** See Suppl. material 1.

**Remarks.**Randiellidae contains a single genus, *Randiella* Erséus & Strehlow, 1986, and four species, all of which are marine (Erséus and Strehlow 1986) and found on the continental shelf of the Nearctic and in shallow waters of the Pacific. The discovery of a *Randiella* species in New Caledonia (Erséus 1997) gives the family a disjunct distribution and suggests the group is more widespread than current records indicate.

**Environment and habitat.** Aquatic, marine; coastal or continental shelf; soft substrata.

#### ﻿Rhinodrilidae Benham, 1890 [megadrile]

Fig. 106

**Common name.** None.

**LSID.** Urn:lsid:marinespecies.org:taxname:1040004.

**Diagnosis (Level 3).** Gizzard present; chaetae first appearing on second segment after peristomium (= S3 for oligochaete workers) (Fig. 106A); dorsal pores on mid-dorsal line absent; calciferous glands present; male pores in segment following testicular segment (plesioporous); spermathecal pores, 3 pairs.

**Description.** See Suppl. material 1.

**Remarks.**Rhinodrilidae includes 38–45 genera and ~ 376 species (Misirlioğlu et al. 2023), but these numbers include many names that have yet to be formally assessed (WoRMS 2025). The family has a Nearctic and Neotropical distribution; one species, *Pontoscolexcorethrurus* (Müller), is probably the most widely distributed earthworm in the world (Misirlioğlu et al. 2023 and references therein), having been introduced widely including to Australia (Blakemore 1999), and has been extensively used as a biological model in soil science.

**Environment and habitat.** Terrestrial.

#### ﻿Sabellariidae Johnston, 1865 [polychaete]

Fig. 107

**Common name.** Honeycomb worms, sandcastle worms.

**LSID.** Urn:lsid:marinespecies.org:taxname:979.

**Figures 107, 108.**
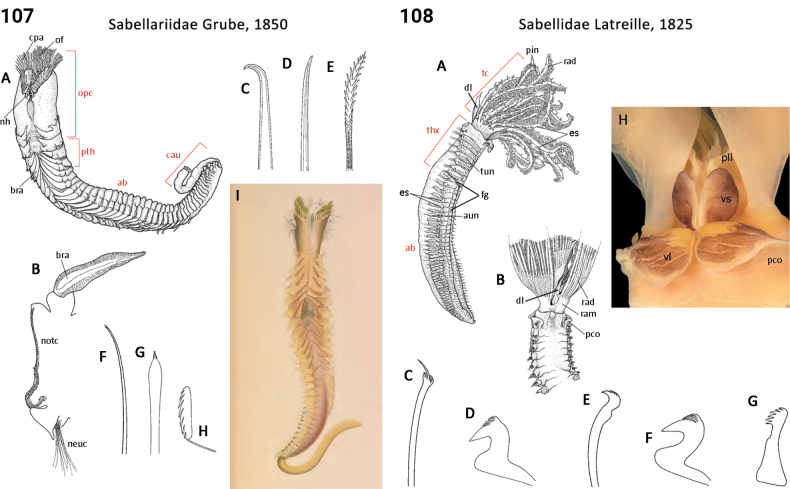
Distinguishing features: **107.**Sabellariidae: **A–H.***Idanthyrsuspennatus*: **A.** Entire animal dorsolateral view; **B.** Parapodium of chaetiger 7 posterior view; **C.** Dorsal hook from operculum; **D.** Inner palea from opercular peduncle; **E.** Outer palea from opercular peduncle; **F, G.** Chaetae from chaetiger 2: **F.** Accessory chaeta; **G.** Oar-shaped chaeta; **H.** Notopodial uncinus from chaetiger 1; **I.** Entire animal of *Sabellariaspinulosa*. Abbreviations: ab abdomen bra branchia cau caudal region nh nuchal hook notc notochaetae neuc neurochaetae of oral filaments opa opercula palea opc operculum pth parathoracic region. Sources: **A–H** derivatives of fig. 1.98 Beesley et al. (2000), **I** derivatives of MacIntosh (1900–1922), pl. CXII, fig. 1. **108.**Sabellidae: **A, B.***Branchiomma* species: **A.** Whole animal lateral view; **B.** Anterior end of *Sabellaspallanzanii* showing proximal half of tentacular crown dorsal view; **C–E.** Thoracic chaetae; **C.** ‘Companion’ notochaeta of *Demonax* sp.; **D.** Neurochaetal uncinus of *Demonax* sp.; **E.** Neurochaetal uncinus of *Fabriciola* sp.; **F, G.** Abdominal notochaetae: **F.** Uncinus of *Demonax* sp.; **G.** Uncinus of *Fabriciola* sp.; **H.** Detail of base of radiolar crown ventral view *S.spallanzanii*. Abbreviations: ab abdomen aun abdominal uncini dl dorsal lip es eye spot fg faecal groove pco peristomial collar pin pinnule pll parallel lamellae rad radiole ram radiolar membrane rc radiolar crown thx thorax tun thoracic uncini vl ventral lappets vs ventral sacs. Sources: **A–G** derivatives of fig. 1.99 Beesley et al. (2000), **H** derivative of fig. 7.4.6.3 Capa et al. (2019).

**Diagnosis (Level 3).** Body widest anteriorly and tapering posteriorly (Fig. 107A); thoracic ventral glandular areas absent; paleate chaetae present (Fig. 107A, D, E; opa); caudal region an unsegmented tube (Fig. 107A; cau).

**Description.** See Suppl. material 1.

**Remarks.**Sabellariidae comprises 12 genera and 152 species and has a global distribution (WoRMS 2025). Kirtley (1994) provides well-illustrated keys for the family, Capa et al. (2012a) provide keys to genera, and Gil (2011) provides a key to European taxa. Zhang et al. (2020) provide a key to all Australian species of sabellariids, and Chávez-López (2022) provides a key to all Caribbean Sea and the Gulf of Mexico species. The family has been recorded from all oceans; although most sabellariids live in intertidal or shallow habitats, some are restricted to the continental shelf or deep sea (Kirtley 1994; Capa et al. 2012a).

**Environment and habitat.** Aquatic, marine; coastal or continental shelf or deep sea; hard substrata.

#### ﻿Sabellidae Latreille, 1825 [polychaete]

Fig. 108

**Common name.** Fan worms; Feather duster worms, eyelash worms (*Myxicola* species), spirograph worm (*Sabellaspallanzanii*).

**LSID.** Urn:lsid:marinespecies.org:taxname:985.

**Diagnosis (Level 3).** Radiolar crown present, modified radioles absent (Fig. 108A, tc, rad); body regions (thorax and abdomen) demarcated by inversion of parapodia; faecal groove present (Fig. 108A, fg).

**Description.** See Suppl. material 1.

**Remarks.**Sabellidae included three subfamilies – Fabriciinae, Myxicolinae and Sabellinae – until relatively recently (as coded in POLiKEY; Glasby and Fauchald 2003). The family was later restricted to the latter two families by Kupriyanova and Rouse (2008) who showed that Fabriciinae were closer to Serpulidae than Sabellidae. Later, Tilic et al. (2020), using a large-scale phylogenomic dataset, found that Fabriciidae were actually the sister group of Serpulidae + Sabellidae. Today, Sabellidae is represented by 42 genera and 552 species (Capa et al. 2019a; WoRMS 2025), and is distributed worldwide mostly in marine areas from the deep sea to the intertidal; the family has a relatively high proportion of freshwater species, with one genus (*Caobangia* Giard, 1893) having colonised and radiated in fresh water in Southeast Asia. Tovar-Hernández and Salazar-Vallejo (2006) provide a taxonomic key for the sabellid species occurring in the Grand Caribbean, Giangrande et al. (2015) and Cepeda et al. (2022) provide keys and species diagnoses for Mediterranean and Atlantic species. Capa and Murray (2009) provide a key to Australian species of *Megalomma* Johansson, 1926 (now accepted as *Acromegalomma* Gil & Nishi, 2017) and Capa and Murray (2015) provide a key to species of *Parasabella* Bush, 1905 and *Sabellomma* Nogueira, Fitzhugh & Rossi, 2010 from Australia. Sabellidae in Australia is represented by a relatively high proportion of non-native species (14 species), including the infamous spirograph worm (*Sabellaspallanzanii* (Gmelin, 1791)), which can be identified using the online key of Kupriyanova et al. (2013); along with species of Serpulidae they are one of the most reported invasive polychaete families (Capa et al. 2021).

**Environment and habitat.** Aquatic, marine, brackish, or freshwater (rarely); coastal, continental shelf or deep sea; soft or hard substrata, epizoic or endozoic (some species bore into mollusks and coral).

#### ﻿Saccocirridae Czerniavsky, 1881 [polychaete]

Fig. 109

**Common name.** None.

**LSID.** Urn:lsid:marinespecies.org:taxname:995.

**Figures 109, 110.**
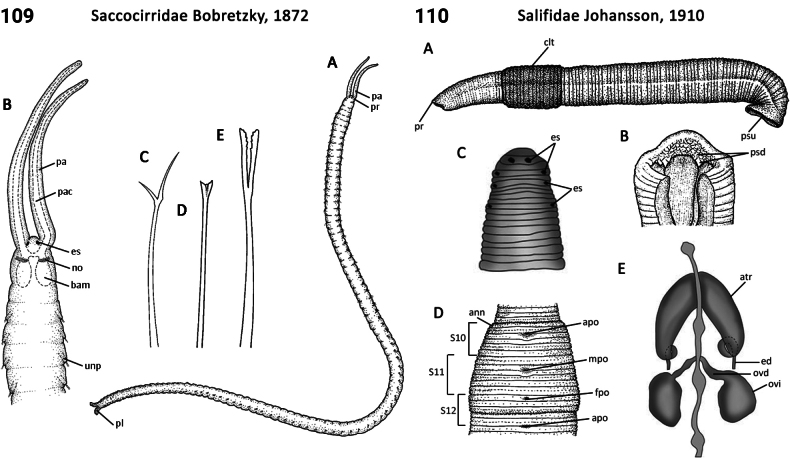
Distinguishing features: **109.**Saccocirridae: **A–E.***Pharyngocirrusjouinae*: **A.** Entire animal, dorsal view; **B.** Anterior end, dorsal view; **C–E.** Chaetae. Abbreviations: bam basal ampulla es eye spot no nuchal organ pa palp pac palp canal pl pygidial lobe pr prostomium unp uniramous parapodium. Sources: **A–E** derivatives of fig. 1.128 Beesley et al. (2000). **110.**Salifidae: **A.***Barbroniaassiuti*, lateral view; **B.** Pharynx of *Odontobdellakrishna* showing pseudognaths and stylets; **C.** Arrangement of eyespots in *Salifaperspicax*; **D.** Ventral view of clitellum of *B.assiuti* with genital and accessory openings; the latter are found only in some species; **E.** Male and female genitalia (dark shading) of *S.perspicaxatrium*. Abbreviations: ann annulus apo accessory pore clt clitellum ed ejaculatory duct fpo female pore mpo male pore ovi ovisac ovd oviduct pr prostomium psd pseudognath psu posterior sucker S segment. Sources: **A, D** after fig. 1 Sket and Trontelj (2008), **B** after fig. 3 Nesemann and Sharma (2012), **C, E** after fig. 2 Oceguera-Figueroa et al. (2010).

**Diagnosis (Level 3).** In life, body translucent, gut visible; prostomium bluntly conical, eyes present (Fig. 109B); palps present, anteroventral (Fig. 109A, B); ventral groove present; first and second segment chaetous (as are all following segments); parapodia present, uniramous, notopodial lobes absent (Fig. 109B, unp); pygidium bilobed (Fig. 109A, pl).

**Description.** See Suppl. material 1.

**Remarks.**Saccocirridae are small interstitial polychaetes belonging to Protodrilida, together with Protodrilidae and Protodriloididae (Rouse et al. 2022). The family is represented by two genera and 24 species (Di Domenico et al. 2021; WoRMS 2025) and has a global distribution in marine coastal sands at low to mid-latitudes, but appears to be uncommon at high latitudes (GBIF.org 2023). Gil (2011) and Parapar et al. (2018) provide keys to European *Saccocirrus* Bobretzky, 1872.

**Environment and habitat.** Aquatic, marine; coastal, littoral or supralittoral; soft substrata (coarse sand and gravel, especially in surf zone).

#### ﻿Salifidae Johansson, 1910 [leech]

Fig. 110

**Common name.** None.

**LSID.** Urn:lsid:marinespecies.org:taxname:1488164.

**Diagnosis (Level 3).** Body dorsoventrally flattened, mid-body with secondary annulation, 6-annulate (Fig. 110D); eyes on head present (Fig. 110C); pharyngeal ridges rotated 60° to right (strepsilaematous); testes, present but only a few pairs, two pairs per segment.

**Description.** See Suppl. material 1.

**Remarks.**Salifidae belongs to the jawed Hirudiniformes (Arhynchobdella) and is found mainly in Africa, India, Australia, and islands in the South Pacific (Oceguera-Figueroa et al. 2011). Nakano (2011) added the genus *Mimobdella* Blanchard, 1897 under Salifidae on the basis of morphological and molecular analyses, yielding seven current genera (Nakano and Nguyen 2015). The family is part of the jawless Arhynchobdella together with Erpobdellidae and Americobdellidae. The Asian freshwater salfid leech, *Barbroniaweberi* Blanchard, 1897, has been introduced to Europe and the Americas (GBIF Secretariat 2025, https://www.gbif.org/species/2307447).

**Environment and habitat.** Aquatic (rarely terrestrial), freshwater; soft substrata.

#### ﻿Scalibregmatidae, Scalibregmatinae Malmgren, 1867 [polychaete]

Fig. 111

**Common name.** Maggot worms.

**LSID.** Urn:lsid:marinespecies.org:taxname:925.

**Figures 111, 112.**
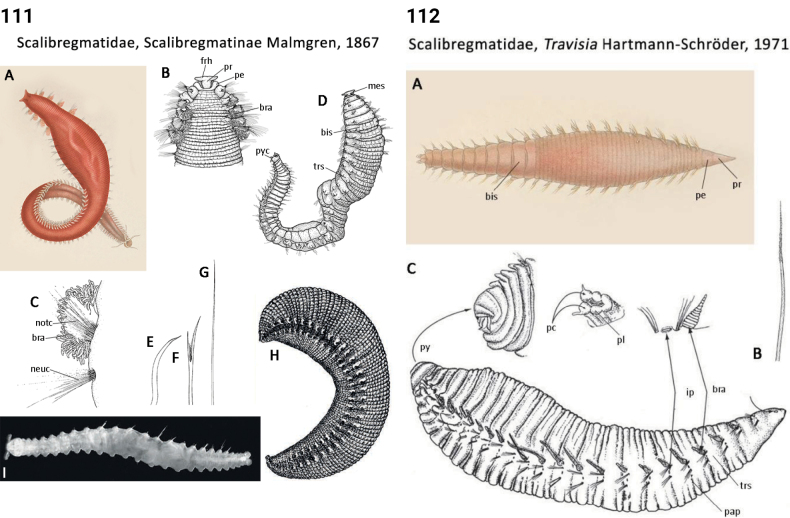
Distinguishing features: **111.**Scalibregmatidae, Scalibregmatinae: **A–C.***Scalibregma* sp.: **A.** Entire animal; **B.** Anterior end dorsal view; **C.** Parapodium of chaetiger 5; **D.***Hyboscolexdichranochaetus* entire animal dorsolateral view; **E–G.***Asclerocheilusheterochaetus*; **E.** Spine from notopodium of chaetiger 1; **F.** Furcate chaeta from mid body parapodium; **G.** Capillary from mid body parapodium; **H.***Polyphysiacrassa* with a maggot-shaped body; **I.***Axiokebuita* sp. with a narrow elongate body. Abbreviations: bis biannulate segment bra branchia frh frontal horn mes merged eyespots neuc neurochaetae notc notochaetae pe peristomium pr prostomium pyc pygidial cirri trs triannulate segment. Sources: **A** derivative of MacIntosh (1900–1922), pl. LXXXVIII, fig. 4, **B–G** derivatives of fig. 1.56 Beesley et al. (2000), **H–I** derivatives of fig. 7.6.3.1 Blake (2019). **112.**Scalibregmatidae, genus *Travisia*: **A.***Travisiaforbesii* entire animal; **B.**Travisiacf.concinna capillary chaeta from parapodium of chaetiger 18; **C.***Travisiapalmeri* entire animal in lateral view with insets of a parapodium, posterior end showing anal cirri, and detail of pygidium. Abbreviations: bra branchia bis biannulate segment ip interramal papilla pap papillae pc pygidial cirrus pe peristomium pl pygidial lobe pr prostomium py pygidium trs triannulate segment. Sources: **A** derivative of MacIntosh (1900–1922), pl. LXXXVIII, fig. 3, **B** derivative of fig. 1.52 Beesley et al. (2000), **C** derivative of fig. 7.6.2.1 Blake (2019).

**Diagnosis (Level 3).** Body usually widest anteriorly and tapering posteriorly or grub-shaped, with epidermis thick and rugose or tessellated (Fig. 111A, D, H, I); prostomium T-shaped (resembling frontal horns), wide end anteriorly (Fig. 111B; frh); ventral groove present; forked chaetae present (Fig. 111D).

**Description.** See Suppl. material 1.

**Remarks.**Scalibregmatinae comprises 15 genera and 88 species (WoRMS 2025), and has a global distribution. The family Scalibregmatidae is particularly common in shallow marine waters of northern Europe and the Antarctic Ocean (Parapar et al. 2021b). Gil (2011) provides a key to European taxa, including the majority of genera; it also includes *Travisia* Johnston, 1840, which until recently was considered a separate family (Travisiidae) but is now considered to belong to Scalibregmatidae, but distinct from Scalibregmatinae (see next entry). Mendes et al. (2024) provide a key to *Pseudoscalibregma* Ashworth, 1901 species.

**Environment and habitat.** Aquatic, marine; coastal, continental shelf or deep sea; soft substrata.

#### ﻿Scalibregmatidae, *Travisia* Johnston, 1840 [polychaete]

Fig. 112

**Common name.** Stink worms.

**LSID.** Urn:lsid:marinespecies.org:taxname:322534.

**Diagnosis (Level 2).** Body shape widest anteriorly and tapering posteriorly (Fig. 112A, C); epidermis thick and rugose or papillate; prostomium conical, tapering to a slender tip (Fig. 112A); ventral groove present.

**Description.** See Suppl. material 1.

**Remarks.***Travisia* comprises 43 species (WoRMS 2025) and has a worldwide distribution, although it appears to be better represented in moderate and high latitudes, than low latitudes (GBIF.org 2023). Once considered to belong to Opheliidae, recent reviews (Blake and Maciolek 2019e; Parapar et al. 2021b) have treated it as a monotypic family, Travisiidae. However, we follow Rouse et al. (2022) in placing the genus within Scalibregmatidae which is based on both morphological and molecular evidence (see references within Rouse et al. 2022). *Travisia* is only distinguishable from Opheliidae at DELTA Level 2. Gil (2011) provides a brief taxonomic history of *Travisia* and includes it in his key to European Scalibregmatidae. Wilson and Avery (2023) provide a key to all species of *Travisia*.

**Environment and habitat.** Aquatic, marine; coastal, continental shelf or deep sea; soft substrata.

#### ﻿Semiscolecidae Scriban & Autrum, 1934 [leech]

Fig. 113

**Common name.** None.

**Figures 113, 114.**
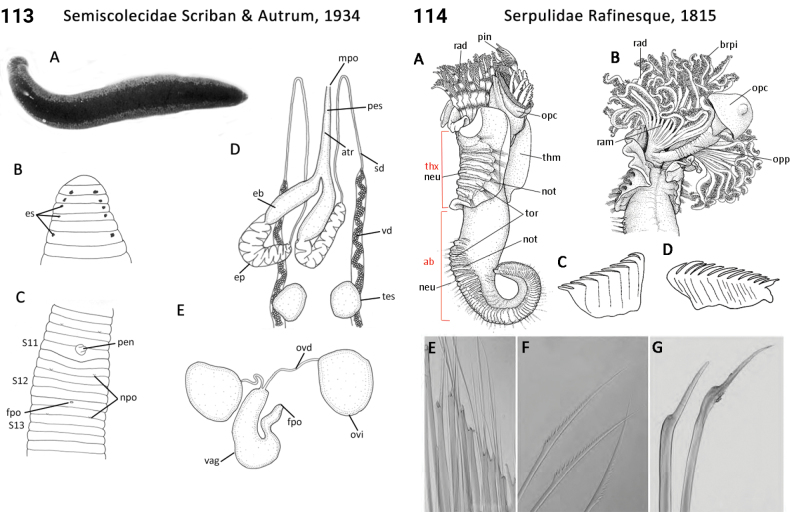
Distinguishing features: **113.**Semiscolecidae: **A.** Whole specimen of *Patagoniobdellavariabilis* showing two-tone color pattern, **B.** Head end, dorsal view showing eyes arranged in an arc; **C.** Segments 11–13 ventral view showing gonopores and nephridial pores; **D, E.** Male (**D**) and female (**E**) reproductive system. Abbreviations: atr atrium eb ejaculatory bulb, ep epididymus, es eye spot, fpo female pore, mpo male pore, npo nephridial pore, ovi ovisac, ovd oviduct, pen penis, pes penis sheath, S segment, sd sperm duct, tes testis, vag vagina, vd vas deferens. Sources: **A** after fig. 15 Siddall and Borda (2004), **B–E** after figs 2–5 Oceguera-Figueroa (2006). **114.**Serpulidae: **A.***Galeolariacaespitosa*, entire animal, lateral view; **B.***Neovermiliaglobula*, anterior end, dorsal view; **C, D.** Uncini of *G.caespitosa*; **C.** Thoracic neuropodial uncinus; **D.** Notopodial uncinus from abdominal chaetiger; **E–G.** Collar chaetae: **E.** Bayonet-type chaetae of *Hydroides*; **F.***Ficopomatus* – type chaetae; **G.***Spirobranchus*-type chaetae. Abbreviations: ab abdomen brpi branchial pinnule neu neuropodium not notopodium opc operculum opp opercular peduncle ped peduncle rad radiole ram radiolar membrane thm thoracic membrane thx thorax tor torus. Sources: **A–D** derivatives of fig. 1.101 Beesley et al. (2000), **E–G** derivatives of fig. 7.4.7.2 Kupriyanova et al. (2019).

**Diagnosis (Level 0).** Jaws with one row of teeth; posterior sucker with rays; egg sacs globular (Fig. 113E, ovi).

**Description.** See Suppl. material 1.

**Remarks.**Semiscolecidae with type genus, *Semiscolex* Kinberg, 1866, is a poorly-known family (Siddall et al. 2006) belonging to the jawed Hirudiniformes (Arhyncobdellida), which now includes both blood-feeding and invertebrate predatory leeches. Using our dataset, the family is not diagnosable (DELTA Diagnostic Level 0) being inseparable from Hirudinidae; we suggest that the family is in need of phylogenetic revision. For taxon confirmation, the user is referred to the full description.

Recently, the concept of Semiscolecidae was expanded to include *Semiscolex*, *Macrobdella*, *Limnatis*, *Limnobdella*, *Oxyptychus*, *Patagoniobdella*, and *Philobdella*, a group which spans the Neotropics and Nearctic (Borda and Siddall 2004; Phillips and Siddall 2009). Phillips et al. (2010), using combined 18S rDNA, 28s rDNA, 12s rDNA, and COI datasets, recognised two smaller monophyletic groups (the North American medicinal leeches, Macrobdellidae and Praobdellidae) within the expanded Semiscolecidae; they formally expanded Praobdellidae to include *Tyrannobdella*, *Dinobdella*, *Myxobdella*, *Praobdella*, and *Pintobdella*. This left Semiscolecidae comprising only the non-bloodfeeding South American taxa (*Semiscolex* and *Patagoniobdella*) [the same genera that Ringuelet (1972b) had included in his emended Semiscolecidae] and Macrobdellinae Richardson, 1969 (Hirudinidae) to encompass the blood-feeding genera *Macrobdella*, *Philobdella* Verrill, 1874, and *Oxyptychus* Grube, 1850.

**Environment and habitat.** Aquatic, freshwater.

#### ﻿Serpulidae Rafinesque, 1815 [polychaete]

Fig. 114

**Common name.** Fan worms; calcareous tubeworms.

**LSID.** Urn:lsid:marinespecies.org:taxname:988.

**Diagnosis (Level 3).** Body regionalized into a thorax and abdomen (Fig. 114A, thx, abd); a radiolar crown bearing in addition to radioles, a single (rarely double or more) peduncular operculum (Fig. 114A, B, opc); thoracic membranes present (Fig. 114A, thm); pygidium bilobed; hard, calcareous tube.

**Description.** See Suppl. material 1.

**Remarks.** ﻿Serpulidae is a diverse group of worms that live in calcareous tubes, comprising three subfamilies (Filograninae, Serpulinae and Spirorbinae) and 74 genera and 578 species (Kupriyanova et al. 2019; WoRMS 2025), and has a global distribution. Hove and Kupriyanova (2009) provide a key to genera, Gil (2011) and Cepeda et al. (2022) provide keys to European taxa, Sun et al. (2012) provide a key to Serpulidae from Hong Kong, Rzhavsky et al. (2014) provide a detailed guide and key to 37 Arctic taxa, Kupriyanova et al. (2015) provide a key to serpulid species from Lizard Island, Great Barrier Reef, Australia, and Pazoki et al. (2023) provide a key to serpulids of the Persian Gulf and Gulf of Oman. Kupriyanova et al. (2023) identified chaetal characters (presence/absence of thoracic ‘Apomatus chaetae’ = ‘sickle-shaped chaetae’ and the structure of abdominal chaetae that define the subfamilies, but at this stage these features have not been coded at the subfamily level. Serpulidae in Australia is represented by a relatively high proportion of non-native species (38 species), which can be identified using the online key of Kupriyanova et al. (2013); along with species of Sabellidae they are one of the most reported invasive polychaete families (Capa et al. 2021).

**Environment and habitat.** Terrestrial or aquatic, including subterranean or hyporheic (rarely), marine, brackish or freshwater (rarely); coastal or continental shelf or deep sea (rarely); soft substrata (rarely), or hard substrata, hydrothermal vents and cold seeps, epizoic, or epiphytic.

#### ﻿Siboglinidae Caullery, 1914 [polychaete]

**Common name.** None – see subfamily groups.

**LSID.** Urn:lsid:marinespecies.org:taxname:129096.

**Diagnosis (Level 3).** Body segments strongly elongate in midbody bearing indistinct parapodia; uncini arising from raised annuli; caudal region (= opisthosoma) very short, multi-segmented; gut absent; tube-dwelling.

**Description.** See Suppl. material 1.

**Remarks.**Siboglinidae is a globally widespread family whose members display a wide range of morphological variation; we have coded the following clades within the family to reduce polymorphism in the dataset: Frenulata, Vestimentifera, *Sclerolinum* Southward, 1961 and *Osedax* Rouse, Goffredi & Vrijenhoek, 2004. Originally, two separate phyla (Pogonophora and Vestimentifera) were placed in the polychaetes by Rouse and Fauchald (1997), and the name Siboglinidae was used to refer to both. Gil (2011) provides a key to European taxa, which includes members of Frenulata, *Sclerolinum*, and *Osedax*.

**Environment and habitat.** Aquatic, marine; coastal (rarely), continental shelf, deep sea; soft or hard substrata or hydrothermal vents and cold seeps, sunken plant material or bones of vertebrates.

#### ﻿Siboglinidae, Frenulata Webb, 1969 [polychaete]

Fig. 115

**Common name.** Beard worms.

**LSID.** Urn:lsid:marinespecies.org:taxname:1298

**Diagnosis (Level 3).** In life, body long and translucent, internal organs visible, gut absent (Fig. 116D); the peristomium expanded glandular ring (Fig. 116B, J, agr); caudal region (= opisthosoma) very short, multi-segmented (Fig. 116B, psr) with four peg-like chaetae in most segments (Fig. 116G–I); tube-dwelling, long tube with transverse ridges (Fig. 116A, D, F).

**Figures 115, 116.**
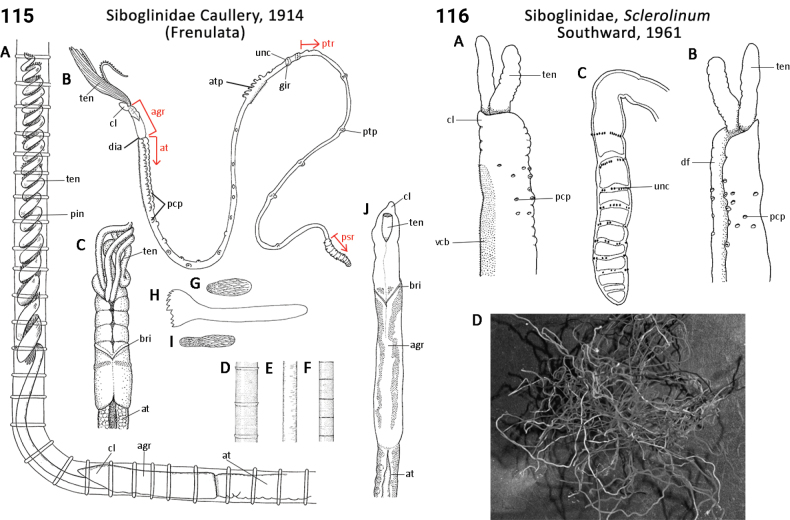
Distinguishing features: **115.**Siboglinidae, Frenulata: **A.***Siboglinum* sp. in tube; **B.** External features of generalised frenulate; **C.***Oligobrachiakernohanae* anterior end of animal dorsal view; **D, E, F.** Sections of tube: **G, H, I.** Girdle chaetae (uncini) of: **G.***Siboglinumatlanticum*; **H.***Siboglinumfiordicum*; **I.***Lamellisabellacoronata*; **J.***Siboglinum* sp., anterior end, dorsal view, most of the tentacle has been removed. Abbreviations: agr anterior gland region at anterior trunk atp anterior trunk papilla bri bridle cl cephalic lobe dia diaphragm gir girdle pcp plaque-capped papilla pin pinnule psr posterior segmented region ptp posterior trunk papillae ptr posterior trunk ten tentacle unc uncini. Sources: **A** derivative of fig. 2 Caullery (1914), **B–J** derivatives of figs 3.2, 3.4, 3.7, 3.14, 3.16 Beesley et al. (2000). **116.**Siboglinidae, *Sclerolinum*: **A–C.***Sclerolinummajor*; **A, B.** Unsegmented anterior end of animal: **A.** Ventrolateral view; **B.** Dorsolateral view; **C.** Segmented posterior end; **D.** Hair-like tubes of *Sclerolinum* sp. Abbreviations: cl cephalic lobe df dorsal furrow pcp plaque-capped papillae ten tentacle unc uncini vcb ventral ciliary band. Sources: **A–D** derivatives of fig. 3.18 Beesley et al. (2000).

**Description.** See Suppl. material 1.

**Remarks.**Frenulata is a clade of Siboglinidae containing members of the former phylum Pogonophora (Rouse et al. 2022). They are widely distributed in all oceans and seas. The group currently has no accepted Linnean rank name and its former families – Oligobrachiidae, Lamellisabellidae, Polybrachiidae and Spirobrachiidae – are now all collectively accepted as Siboglinidae (WoRMS 2025). Southward (1999) provides a key to former pogonophoran families (and *Sclerolinum*) from Australia.

**Environment and habitat.** Aquatic, marine; continental shelf or deep sea; soft substrata (reducing sediments).

#### ﻿Siboglinidae, *Osedax* Rouse, Goffredi & Vrijenhoek, 2004 [polychaete]

**Common name.** Boneworms, bone-eating worms, zombie worms.

**LSID.** Urn:lsid:marinespecies.org:taxname:265008.

**Diagnosis (Level 3).** Hooks present; uncini absent; tube membranous.

**Description.** See Suppl. material 1.

**Remarks.***Osedax* is the sister group of Monilifera within Siboglinidae (Rouse et al. 2004; Li et al. 2015, 2017); although all Siboglinidae harbour symbiotic bacteria in their bodies, *Osedax* is readily distinguishable not only morphologically, but by habitat/behavior (consumes the organic component of bones of sunken marine carcasses), and therefore the genus is scored separately within this dataset. We have based the description on females, as the males are generally progenetic larval dwarfs (Rouse et al. 2022). The genus is represented by over 30 species (WoRMS 2025) and is globally distributed in all oceans and seas. A key to species is lacking.

**Environment and habitat.** Aquatic, marine; deep sea; sunken bones of vertebrates.

#### ﻿Siboglinidae, *Sclerolinum* Southward, 1961 [polychaete]

Fig. 116

**Common name.** None.

**LSID.** Urn:lsid:marinespecies.org:taxname:129106.

**Diagnosis (Level 3).** In life, body opaque, gut usually not visible, pigmentation absent (Fig. 116D); buccal tentacles smooth (Fig. 116A, B, ten); peristomium a single ring; uncini present, hooks absent; tube-dwelling, long and thin tubes (Fig. 116D). (Fig. 116C, unc).

**Description.** See Suppl. material 1.

**Remarks.** The genus *Sclerolinum* and sister group Vestimentifera belong to the clade Monilifera (Rouse 2001). Rather than code Monilifera, we have coded both *Sclerolinum* and Vestimentifera separately in this study as they are morphologically and ecologically distinguishable. *Sclerolinum* inhabits long thin, ringed, tubes, which are often two or three times longer than worm. *Sclerolinum* contains eight species and is possibly globally distributed in all oceans and seas, but current records are patchy (GBIF.org 2023). Xu et al. (2022) provide a key to species.

**Environment and habitat.** Aquatic, marine; deep sea; sunken plant material.

#### ﻿Siboglinidae, Vestimentifera Webb, 1969 [polychaete]

Fig. 117

**Common name.** Vent worms.

**LSID.** Urn:lsid:marinespecies.org:taxname:129094.

**Figures 117, 118.**
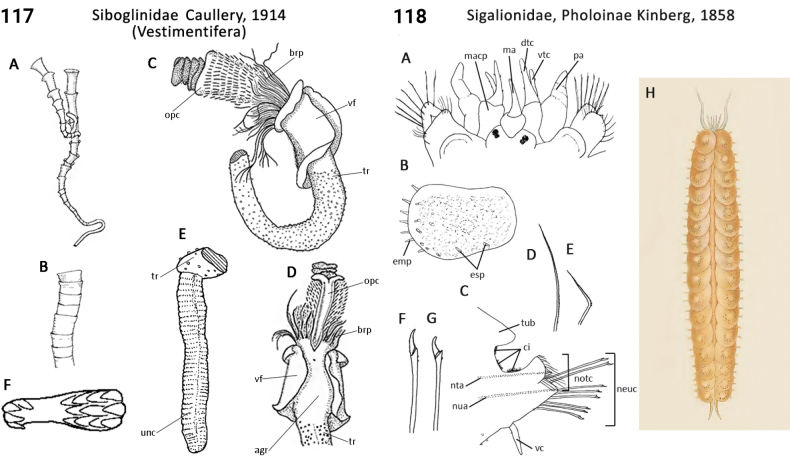
Distinguishing features: **117.**Siboglinidae, Vestimentifera: **A.** Two entire tubes of *Ridgeiapiscesae*; **B.** Anterior section of tube of *Lamellibrachiabarhami*; **C, D, E.** External features of *R.piscesae*: **C.** Lateral view of anterior end and part of trunk; **D.** Dorsal view of anterior end; **E.** Lateral view of posterior end; **F.** Uncini of *Lamellibrachiacolumna*. Abbreviations: agr anterior gland region brp branchial plume opc operculum tr trunk unc uncini vf vestimentiferal flap. Sources: **A–F** derivatives of figs 3.3, 3.6, 3.7 Beesley et al. (2000). **118.**Sigalionidae, Pholoinae: **A–G.***Pholoepolymorpha*: **A.** Anterior end, dorsal view; **B.** Left middle elytron; **C.** Right parapodium with dorsal tubercle, anterior view, acicula dotted; **D, E.** Notochaetae; **F, G.** Upper and lower neurochaetae; **H.** Entire animal, dorsal view of *Pholoeminuta*. Abbreviations: ci cilia dtc dorsal tentacular cirrus emp elytra marginal papilla esp elytra surface papilla ma median antenna macp median antenna ceratophore neuc neurochaetae notc notochaetae nta notoacicula nua neuroacicula pa palp tub tubercle vc ventral cirrus vtc ventral tentacular cirri. Sources: **A–G** derivative of fig. 2 Pettibone (1992), **H** derivatives of MacIntosh (1900–1922), pl. XXVIA, fig. 24.

**Diagnosis (Level 3).** Body pigmentation present; peristomium expanded as an elaborately collared ring (Fig. 117C, D, agr, vf); tube-dwelling, animal able to plug entrance of tube with an operculum (Fig. 117, A, C, opc).

**Description.** See Suppl. material 1.

**Remarks.**Vestimentifera is a clade of Siboglinidae containing members of the former phylum Vestimentifera (Rouse et al. 2022). It currently has no Linnean rank within Siboglinidae and its former families – Alaysiidae, Arcovestiidae, Escarpiidae, Lamellibrachiidae, Ridgeiidae and Tevniidae – are now all collectively accepted as Siboglinidae (WoRMS 2025). Together with the genus *Sclerolinum* it belongs to the clade Monilifera (Rouse 2001). Rather than code Monilifera, we have coded both *Sclerolinum* and Vestimentifera separately in this study as they are both morphologically and ecologically distinguished. Vestimentifera is represented by 18 species (WoRMS 2025). A key to species is lacking.

**Environment and habitat.** Aquatic, marine; coastal (rarely), continental shelf or deep sea; hard substrata, or hydrothermal vents and cold seeps.

#### ﻿Sigalionidae Kinberg, 1856 [polychaete]

**Common name.** Sand scaleworm (general name for members of the family).

**LSID.** Urn:lsid:marinespecies.org:taxname:943.

**Diagnosis (Level 3).** In life, body opaque, gut usually not visible; prostomial antennae present; tentacular cirri present; pharynx jaws present, two pairs; first chaetiger with parapodia anteriorly directed and wrapping around head; notopodial lobes represented by at least one chaetal lobe; compound chaetae present, shaft solid, without a distinct core; spines absent; one pair of pygidial cirri.

**Description.** See Suppl. material 1.

**Remarks.**Sigalionidae is a widely distributed scaleworm family with a wide diversity of forms; as such, we provide subfamily coding to reduce polymorphism in the dataset. We follow the revised classification of Sigalionidae as proposed by Gonzalez et al. (2018) and followed by Rouse et al. (2022), viz. Pelogeniinae, Pisioninae, Pholoinae, Sthenelanellinae, and the currently polyphyletic Sigalioninae. Note that Pisioninae and Pholoinae have been previously regarded as families, including in POLiKEY (see Glasby and Fauchald 2003). Barnich and Fiege (2003) provide keys to Mediterranean species, Gil (2011), and Parapar et al. (2015) provide keys to European sigalionid taxa, which excludes Pisioninae and Pholoinae (each provided separately). Barnich (2011) provides a key to scaleworms, including Sigalionidae, of British and Irish waters.

**Environment and habitat.** Aquatic, marine; coastal, continental shelf or deep sea; soft substrata.

#### ﻿Sigalionidae, Pelogeniinae Chamberlin, 1919 [polychaete]

**LSID.** Urn:lsid:marinespecies.org:taxname:237626

**Diagnosis (Level 1).** Dorsal cirri absent.

**Description.** See Suppl. material 1.

**Remarks.**Pelogeniinae includes eight genera and 41 species (WoRMS 2025) and have a widespread distribution at low and mid latitudes (GBIF.org 2023). The subfamily is similar to Pelogeniinae, Sigalioninae, and Sthenelanellinae and only distinguishable at DELTA Diagnostic Level 1. See Remarks under Sigalionidae sensu lato for references having keys.

**Environment and habitat.** Aquatic, marine; coastal, continental shelf or deep sea; soft substrata.

#### ﻿Sigalionidae, Pholoinae Kinberg, 1858 [polychaete]

Fig. 118

**LSID.** Urn:lsid:marinespecies.org:taxname:941.

**Diagnosis (Level 3).** Dorsal body surface with protective covering scales (elytrae) (Fig. 118H); after segment 7 elytra occurring on every other segment to end of body; prostomium rounded to oval, antennae consist of basal ceratophore and distal ceratostyle (Fig. 118A, macp); facial tubercle present; elytra with raised concentric rings (Fig. 118B); spines absent.

**Description.** See Suppl. material 1.

**Remarks.**Pholoinae includes six genera and 31 species and subspecies (WoRMS 2025) and has a widespread global distribution. Pettibone (1992) revised the group and provided a world key. See Remarks under Sigalionidae sensu lato for further references having keys. The subfamily was established after Pholoidae as a family was demoted by Wiklund et al. (2005), who found molecular evidence that *Pholoe* Johnston, 1839 (and *Pisione* Grube, 1857) both fell within Sigalionidae, and suggested that both family names Pholoidae and Pisionidae should be treated as junior synonyms of Sigalionidae. Evidence for this conclusion has strengthened in recent years, so it is followed here. Pholoidae was included as a family in POLiKEY (Glasby and Fauchald 2003).

**Environment and habitat.** Aquatic, marine, coastal, continental shelf, or deep sea; soft or hard substrata, or epizoic.

#### ﻿Sigalionidae, Pisioninae Ehlers, 1901 [polychaete]

Fig. 119

**LSID.** Urn:lsid:marinespecies.org:taxname:1498425.

**Figures 119, 120.**
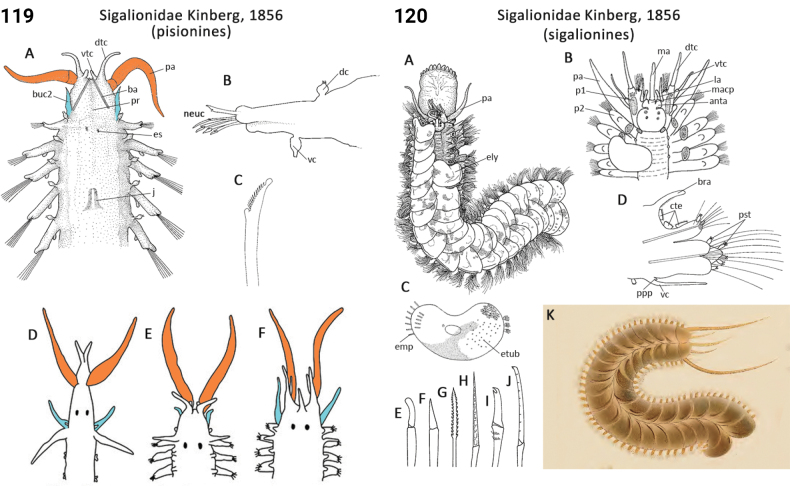
Distinguishing features: **119.**Sigalionidae, Pisioninae: **A–C.***Pisione* sp.: **A.** Anterior end, dorsal view; **B.** Parapodium of chaetiger 22; **C.** Neurochaeta of chaetiger 22; **D–F.** Pisionidens sp., *Anoplopisione* sp., *Pisionella* sp. dorsal views, anterior ends. Abbreviations: ba buccal acicula buc2 buccal cirri (segment 2) dc dorsal cirrus dtc dorsal tentacular cirri es eye spot j jaw neuc neurochaetae pa palp pr prostomium vc ventral cirrus vtc ventral tentacular cirri. Sources: **A–C** derivatives of fig. 1.85 Beesley et al. (2000), **D–F** derivatives of fig. 1 Gonzalez et al. (2022). **120.**Sigalionidae, Sigalioninae: **A.***Fimbriosthenelais* sp. anterior end dorsal view with pharynx everted; **B–J.** Diagrammatic figures of a generalised sigalionid: **B.** Anterior end dorsal view; **C.** Elytron; **D.** Parapodium. **E–J.** Types of neurochaetae: **E.** Compound chaeta with blunt-tipped blade; **F.** Compound chaeta with conical-tipped blade; **G.** Bipinnate spine; **H.** Canaliculate spiniger; **I.** Compound falciger with no articles on blade; **J.** Compound falciger with articulated blade; **K.** Anterior end of *Sthenelaisboa*. Abbreviations: anta antennal auricle bra branchia cte ctenidium dtc dorsal tentacular cirri ely elytron emp elytra marginal papilla etub elytra tubercle la lateral antenna ma median antenna macp median antenna ceratophore p1 parapodium 1 p2 parapodium 2 pa palp ppp parapodia papilla pst parapodial stylode vc ventral cirrus vtc ventral tentacular cirri. Sources: **A–J** derivatives of fig. 1.88 Beesley et al. (2000), **K** derivative of MacIntosh (1900–1922), pl. XXVI, fig. 7.

**Diagnosis (Level 3).** Body surface without protective covering of elytrae; prostomium conical and tapering to slender tip (Fig. 119D) or engulfed by forward projecting tentacular cirri (Fig. 119A, E, F); antennae present, paired lateral (Fig. 119A, D–F).

**Description.** See Suppl. material 1.

**Remarks.**Pisioninae includes four genera and 51 species (WoRMS 2025), and have a widespread global distribution. For the largest genus, *Pisione* (46 species), Viéitez et al. (2004) and Gil (2011) provide keys to European species, separate from other Sigalionidae, and Salcedo et al. (2015) provide a key species of the East Pacific. The subfamily was established after Pisionidae as a family was demoted by Wiklund et al. (2005), who found molecular evidence that *Pisione* (and *Pholoe*) both fell within Sigalionidae, and suggested that both family names Pisionidae and Pholoidae should be treated as junior synonyms of Sigalionidae. Evidence for this conclusion has strengthened in recent years, so it is followed here.

**Environment and habitat.** Aquatic, marine or brackish (very rarely freshwater); coastal (mostly) or continental shelf or deep sea; soft substrata.

#### ﻿Sigalionidae, Sigalioninae Kinberg, 1856 [polychaete]

Fig. 120

**LSID.** Urn:lsid:marinespecies.org:taxname:1499830.

**Diagnosis (Level 1).** Dorsal cirri present; branchiae present (Fig. 120D); tube absent.

**Description.** See Suppl. material 1.

**Remarks.**Sigalioninae, as listed in WoRMS includes 13 genera and 127 species (WoRMS 2025) but another two unplaced genera may be included in this catch-all default subfamily (see Gonzalez et al. 2018). The subfamily is very similar to both Pelogeniinae and Sthenelanellinae, and only distinguishable at DELTA Diagnostic Level 1. Barnich and Van Haaren (2021) provide a key to Atlantic and Mediterranean members of the subfamily. See Remarks under Sigalionidae sensu lato for further references having keys.

**Environment and habitat.** Aquatic, marine; coastal, continental shelf or deep sea; soft substrata.

#### ﻿Sigalionidae, Sthenelanellinae Gonzalez, Martínez, Borda, Iliffe, Eibye-Jacobsen & Worsaae, 2018 [polychaete]

**LSID.** Urn:lsid:marinespecies.org:taxname:1499725.

**Diagnosis (Level 1).** Tube-dwelling, tube leathery or parchment-like reinforced with feltage chaetae.

**Description.** See Suppl. material 1.

**Remarks.**Sthenelanellinae includes the type genus, *Sthenelanella* and six species (WoRMS Dec 2025). The subfamily is very similar to Pelogeniinae and Sigalioninae, and only distinguishable at DELTA Diagnostic Level 1. The subfamily has a widespread distribution at low and mid-latitudes (GBIF.org 2023). See Remarks under Sigalionidae sensu lato for references having keys.

**Environment and habitat.** Aquatic, marine; coastal, continental shelf or deep sea; soft substrata.

#### ﻿Sipuncula Stephen, 1965 [sipunculan]

**Common name.** Peanut worm (general name for members of the family).

**LSID.** Urn:lsid:marinespecies.org:taxname:1268.

**Diagnosis (Level 3).** Body peanut-shaped, segmentation absent; anus positioned near anterior end (dorsally, near the introvert–trunk junction); nuchal organs present.

**Description.** See Suppl. material 1.

**Remarks.** The former phylum Sipuncula, treated here at the ordinal level (as per WoRMS 2025; Rouse et al. 2022), comprises six families: Antillesomatidae, Aspidosiphonidae, Golfingiidae, Phascolosomatidae, Sipunculidae, and Siphonosomatidae (Schulze et al. 2019; Rouse et al. 2022), 18 genera and ~ 170 species (WoRMS 2025), although this is almost certainly an underestimate as many of the putatively cosmopolitan species are likely to be found to comprise species complexes. The phylogenetic placement of sipunculans has long been controversial (see Eibye-Jacobsen and Vinther (2012), Saiz Salinas (2018)), and it was not until molecular evidence became available that their position within Annelida has stabilized. Their position as part of Annelida was firmly established based on a phylogenetic analysis of transcriptomic data, which supported preceding molecular studies (Weigert et al. 2014 and references therein). Cutler (1994) provides a key to global taxa known at the time, and Pancucci-Papadopoulou et al. (1999) provide a key to Mediterranean sipunculans.

**Environment and habitat.** Aquatic, marine or brackish; coastal, continental shelf, deep sea, littoral or supralittoral; soft or hard substrata, or sunken plant material.

#### ﻿Sipuncula, Antillesomatidae Kawauchi, Sharma & Giribet, 2012 [sipunculan]

Fig. 121

**LSID.** Urn:lsid:marinespecies.org:taxname:1450687.

**Figures 121, 122.**
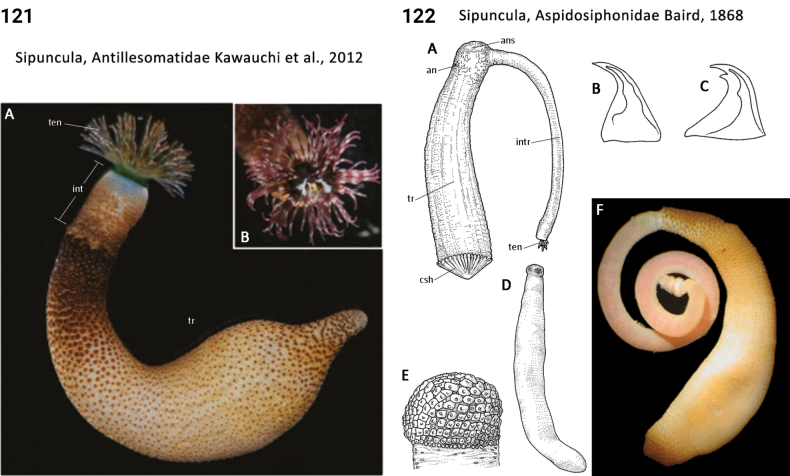
Distinguishing features: **121.**Sipuncula, Antillesomatidae: **A.***Antillesomaantillarum* whole animal; **B.** Tentacular crown. Abbreviations: intr introvert ten tentacle tr trunk. Sources: **A, B** after fig. 6.1.9 Schulze et al. (2019). **122.**Sipuncula, Aspidosiphonidae: **A, B, C.***Aspidosiphon* species: **A.** Entire animal; **B, C.** Introvert hooks; **D, E.***Cloeosiphonaspergillus*: **D.** Entire animal; **E.** Anterior end; **F.***Aspidosiphonparvulus* entire animal. Abbreviations: an anus ans anal shield csh caudal shield intr introvert ten tentacle tr trunk. Sources: **A–C** after fig. 5.16, **D, E** after fig. 5.17 Beesley et al. (2000), **F** after fig. 6.1.9 Schulze et al. (2019).

**Diagnosis (Level 1).** Introvert shorter than trunk (Fig. 121A); buccal tentacles arise from one side of mouth (Fig. 121B).

**Description.** See Suppl. material 1.

**Remarks.**Antillesomatidae comprises a single genus, *Antillesoma* (Stephen & Edmonds, 1972) and two species (WoRMS 2025), and appears to have a widespread distribution in warm, shallow waters at low and mid-latitudes (GBIF.org 2023). Antillesomatidae is similar to other Sipuncula families, and only distinguishable at DELTA Diagnostic Level 1. See Remarks under Sipuncula sensu lato for references having keys.

**Environment and habitat.** See Sipuncula

#### ﻿Sipuncula, Aspidosiphonidae Quatrefages, 1865 [sipunculan]

Fig. 122

**LSID.** Urn:lsid:marinespecies.org:taxname:1644.

**Diagnosis (Level 0).** Introvert longer than trunk (Fig. 122A, F).

**Description.** See Suppl. material 1.

**Remarks.**Aspidosiphonidae comprises three genera and 24 species (WoRMS 2025), and has a widespread global distribution. It is one of four sipunculan families well represented in deep waters (greater than 2000 m) (Saiz Salinas et al. 2016). Aspidosiphonidae is not diagnosable (DELTA Diagnostic Level 0) from Antillesomatidae and Phascolosomatidae using the present dataset; see Suppl. material 1 for a full description of the family. See Remarks under Sipuncula sensu lato for references having keys.

**Environment and habitat.** See Sipuncula.

#### ﻿Sipuncula, Golfingiidae Stephen & Edmonds, 1972 [sipunculan]

Fig. 123

**LSID.** Urn:lsid:marinespecies.org:taxname:2032.

**Figures 123, 124.**
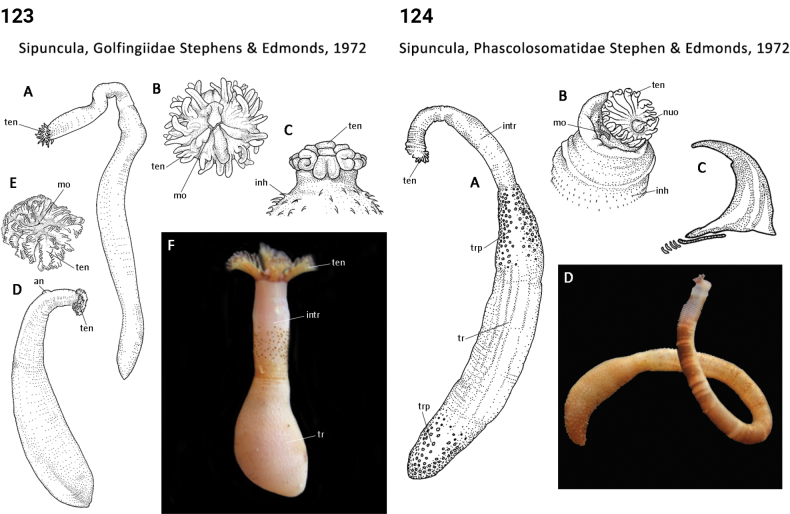
Distinguishing features: **123.**Sipuncula, Golfingiidae: **A.***Golfingiamargaritacea* entire specimen; **B.** Oral disk of *Golfingia* sp. **C.** Anterior region of *Nephasomaabyssorum*; **D, E.***Themistecymodoceae*: **D.** Entire specimen; **E.** Branching structure of tentacles; **F.***Themistealutacea* entire animal. Abbreviations: an anus inh introvert hook intr introvert mo mouth ten tentacle tr trunk. Sources: **A–C** after fig. 5.12, **D, E** after fig. 5.13 Beesley et al. (2000); **F** after fig. 6.1.8 Schulze et al. (2019). **124.**Sipuncula, Phascolosomatidae: **A.***Phascolosomascolops* entire specimen; **B.***Phascolosoma* species, tip of introvert showing tentacles; **C.** Introvert hook of *P.stephensoni*; **D.***P.nigrescens* entire specimen. Abbreviations: inh introvert hook intr introvert mo mouth nuo nuchal organ ten tentacle trp trunk papilla Sources: **A–C** after fig. 5.15 Beesley et al. (2000), **D** after fig. 6.1.9 Schulze et al. (2019).

**Diagnosis (Level 1).** Anterior extremity of trunk with hardened or calcareous structures absent; introvert papillae absent; buccal tentacles surrounding perimeter of mouth (Fig. 123B, ten, mo).

**Description.** See Suppl. material 1.

**Remarks.**Golfingiidae comprises seven genera and 80 species (WoRMS 2025) and has a widespread global distribution. It is one of four sipunculan families well represented in deep waters (greater than 2000 m) (Saiz Salinas et al. 2016). Golfingiidae is similar to Antillesomatidae and Siphonosomatidae, and is only distinguishable at DELTA Diagnostic Level 1. See Remarks under Sipuncula sensu lato for references having keys.

**Environment and habitat.** See Sipuncula.

#### ﻿Sipuncula, Phascolosomatidae Stephen & Edmonds, 1972 [sipunculan]

Fig. 124

**LSID.** Urn:lsid:marinespecies.org:taxname:1645.

**Diagnosis(Level 0).** Anterior extremity of trunk with hardened or calcareous structures (hardened papillae) present (Fig. 124A, trp); introvert about equal to trunk, or longer than trunk (Fig. 124A, D).

**Description.** See Suppl. material 1.

**Remarks.**Phascolosomatidae comprise three genera and 30 species (WoRMS 2025), and has a widespread global distribution. It is one of four sipunculan families well represented in deep waters (greater than 2000 m) (Saiz Salinas et al. 2016). Phascolosomatidae is not distinguishable (DELTA Level 0) from Antillesomatidae and Aspidosiphonidae using the present dataset; see Suppl. material 1 for a full description of the family. See Remarks under Sipuncula sensu lato for references having keys.

**Environment and habitat.** See Sipuncula.

#### ﻿Sipuncula, Siphonosomatidae Kuwauchi et al., 2012 [sipunculan]

Fig. 125

**LSID.** Urn:lsid:marinespecies.org:taxname:1450685.

**Figures 125, 126.**
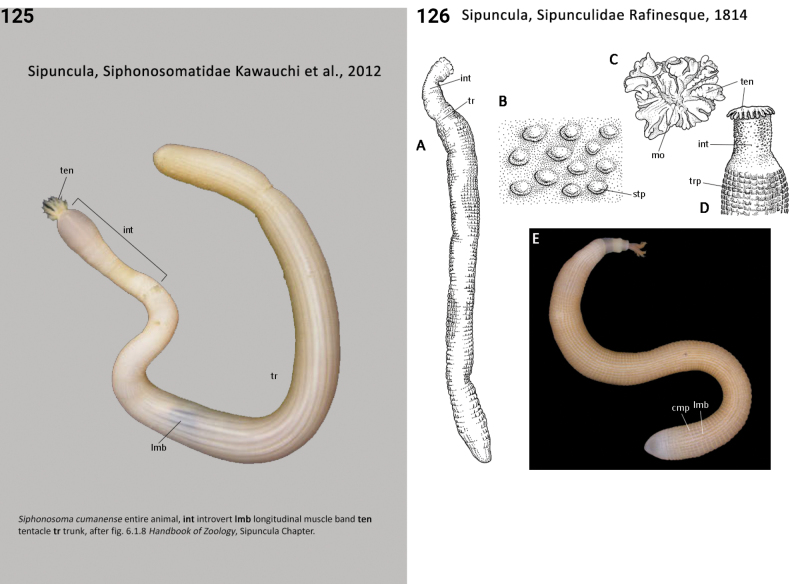
Distinguishing features: **125.**Sipuncula, Siphonosomatidae: *Siphonosomacumanense* entire animal. Abbreviations: int introvert lmb longitudinal muscle band ten tentacle tr trunk. Sources: after fig. 6.1.8 Schulze et al. (2019). **126.**Sipuncula, Sipunculidae: **A.***Sipunculusrobustus* entire animal; **B.** Details of subtriangular papillae from surface of introvert; **C.** Oral disk of *Sipunculusnudus*; **D.** Anterior region of a *Sipunculus* sp. **E.***S.nudus* entire animal. Abbreviations: cmb circular muscle band intr introvert lmb longitudinal muscle band mo mouth stp subtriangular papillae ten tentacle tr trunk trp trunk papillae. Sources: **A–C** after fig. 5.11, **D** after fig. 5.2 Beesley et al. (2000), **E** after fig. 6.1.7 Schulze et al. (2019).

**Diagnosis (Level 1).** Trunk smooth (Fig. 125); introvert papillae present.

**Remarks.**Siphonosomatidae comprise two genera and 16 species (WoRMS 2025), and appears to have a widespread distribution in warm shallow waters at low and mid latitudes (GBIF.org 2023). Siphonosomatidae is similar to Antillesomatidae, Golfingiidae and Sipunculidae are only distinguishable at DELTA Diagnostic Level 1. See Remarks under Sipuncula sensu lato for literature having keys.

**Environment and habitat.** See Sipuncula.

#### ﻿Sipuncula, Sipunculidae Rafinesque, 1814 [sipunculan]

Fig. 126

**LSID.** Urn:lsid:marinespecies.org:taxname:1648.

**Diagnosis (Level 1).** Anterior extremity of trunk roughened by papillae or rounded skin bodies (Fig. 126A, B, D, E, trp); introvert papillae present; buccal tentacles present, surrounding perimeter of mouth (Fig. 126C, D, ten).

**Description.** See Suppl. material 1.

**Remarks.**Sipunculidae comprise two genera and 19 species (WoRMS 2025), and has a widespread global distribution. It is one of four sipunculan families well represented in deep waters (greater than 2000 m) (Saiz Salinas et al. 2016). Sipunculidae is similar to Antillesomatidae and Siphonosomatidae and only distinguishable at DELTA Diagnostic Level 1. See Remarks under Sipuncula sensu lato for references having keys.

**Environment and habitat.** See Sipuncula

#### ﻿Sparganophilidae Michaelsen, 1921 [megadrile]

Fig. 127

**Common name.** None.

**LSID.** Urn:lsid:marinespecies.org:taxname:992681.

**Figures 127, 128.**
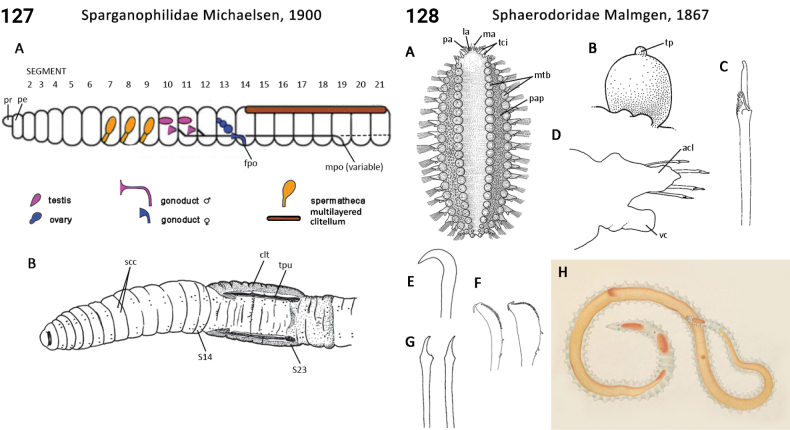
Distinguishing features: **127.**Sparganophilidae: **A.** Schematic image of reproductive organs, dorsal view; **B.***Sparganophilustamesis* anterior end with genital region, ventral view. Abbreviations: clt multilayered clitellum, fpo female pore mpo male pore pe peristomium pr prostomium S segment scc simple crochet chaeta tpu tubercula pubertatis. Sources: **A, B** derivatives of fig. 8.4 A, 8.40 B Jamieson (2006). **128.**Sphaerodoridae: **A, B.***Sphaerephesia* sp.: **A.** Entire animal dorsal view; **B.** Dorsal macrotubule; **C, D.***Sphaerodoropsis* sp.; **C.** Compound falciger from chaetiger 9; **D.** Uniramous parapodium from chaetiger 9; **E.***Ephesiopsisguayanae* recurved hook from first chaetiger; **F.***Euritmiahamulisetosa* compound chaeta; **G.***Sphaerodorumflavum* simple chaeta; **H.***S.gracilis*, entire animal. Abbreviations: acl acicular lobe la lateral antenna ma median antenna mtb macrotubercule pa palp pap papilla tci tentacular cirri tp terminal papilla vc ventral cirrus. Sources: **A–D** derivatives of fig. 1.89 Beesley et al. (2000), **E–G** derivatives of fig. 13, 14 Capa et al. (2022), **H** derivative of McIntosh (1900–1922), pl. LXXXVIII, fig. 10.

**Diagnosis (Level 1).** Secondary annulation present; gut straight with side branches; clitellum situated posterior to female pore (Fig. 127A); genital chaetae absent; spermathecal pores, 2 or 3 pairs.

**Description.** See Suppl. material 1.

**Remarks.**Sparganophilidae has only one genus (*Sparganophilus* Benham, 1892) and 11 species primarily in the Northern Hemisphere (WoRMS 2025). Most species occur in the Nearctic, but the family also occurs in the Palearctic and Neotropical realms (Martin et al. 2008); at least one species is widespread in the Northern Hemisphere (Misirlioğlu et al. 2023 and references therein). It is one of the few megadrile families that include species that are aquatic or semi-aquatic (Martin et al. 2008). Molecular data (28S, 18S, 16S) strongly support a sister-group relationship between Sparganophilidae and the monogeneric Komarekionidae (only species, *Komarekionaeatoni* Gates, 1974 from the eastern USA) (Jamieson et al. 2002; James and Davidson 2012) and on this basis we have included data of *Komarekionaeatoni* together with Sparganophilidae. By contrast, morphology supports a closer relationship between *Ailoscolex* Bouché, 1969 and *Komarekiona* Gates, 1974 prompting Sims (1980) to synonymise Komarekionidae with Ailoscolecidae (now Hormogastridae). Gates and Reynolds (2017) provide a key to North American members of this family. Our data reveal that the family is similar to Tritogeniidae and only distinguishable at DELTA Diagnostic Level 1.

**Environment and habitat.** Terrestrial or aquatic (rarely), freshwater (wetlands); soft substrata.

#### ﻿Sphaerodoridae Malmgren, 1867 [polychaete]

Fig. 128

**Common name.** None.

**LSID.** Urn:lsid:marinespecies.org:taxname:957.

**Diagnosis (Level 1).** Body with papillate or tuberculate epidermis (Fig. 128A, mtb, pap); pharynx proventricle present (Fig. 128H); capillary chaetae absent; pygidial appendages present, one pair of cirri and single medial papilla.

**Description.** See Suppl. material 1.

**Remarks.**Sphaerodoridae includes 11 genera and 133 species (WoRMS 2025), and has a global distribution. The family is similar to Syllidae and only distinguishable at DELTA Diagnostic Level 1. Generic delimitation is based on the number of longitudinal and transverse rows of dorsal macrotubercles (Capa et al. 2019b). Fauchald (1974) provided a key to all species then known, while the family has been recently revised by Capa and colleagues (Capa et al. 2018, 2019b). Gil (2011) and Parapar et al. (2012) provide keys to European taxa, and Capa et al. (2016) provide a key to species reported from the Northwestern Atlantic. Capa and Bakken (2015) provide a key to Australian species. Reuscher and Fiege (2011) provide a key to all *Sphaerodoropsis* Hartman & Fauchald, 1971 species.

**Environment and habitat.** Aquatic, marine; coastal, continental shelf or deep sea; soft or hard substrata, hydrothermal vents, and cold seeps, epizoic, or sunken bones of vertebrates.

#### ﻿Spintheridae Augener, 1913 [polychaete]

Fig. 129

**Common name.** None.

**LSID.** Urn:lsid:marinespecies.org:taxname:962.

**Figures 129, 130.**
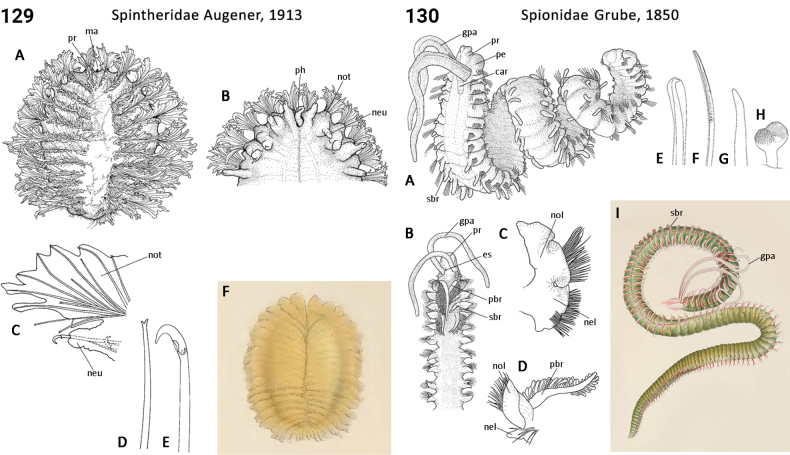
Distinguishing features: **129.**Spintheridae: **A–F.***Spinther* sp.: **A.** Entire animal dorsal view; **B.** Anterior end ventral view; **C.** Neuropodium and part of notopodium from chaetiger 8 anterior view; **D.** Notochaetal spine from chaetiger 8; **E.** Compound neurochaeta from chaetiger 8; **F.** Entire animal. ma median antenna neu neuropodium not notopodium ph pharynx pr prostomium. Sources: **A–E** derivatives from fig. 1.94 Beesley et al. (2000), **F** derivative of MacIntosh (1900–1922), pl. XXIV, fig. 1. **130.**Spionidae: **A.** Entire animal of *Polydora* sp.; **B.** Anterior end of *Prionospiocoorilla* dorsal view; **C.** Parapodium from chaetiger 25 *Scolelepisvictoriensis*; **D.** Parapodium from chaetiger 5 *Prionospiokirrae*; **E–H.** Chaetae: **E.** Neuropodial hooded hook *Pr.kirrae*; **F.** Neuropodial sabre chaeta from parapodium of chaetiger 48 *Pr.kirrae*; **G.** Spine from parapodium of chaetiger 5 *Carazziella* sp.; **H.** Brush-tipped spine from parapodium of chaetiger 5 *Carazziella* sp.; **I.** Entire animal of *Scolelepissquamata*. Abbreviations: car caruncle es eye spot gpa grooved palp nel neuropodial lobe nol notopodial lobe pbr pinnate branchiae pe peristomium pr prostomium sbr smooth branchiae. Sources: **A–H** after fig. 1.108 Beesley et al. (2000), **I** after MacIntosh (1900–1922), pl. XC, fig. 2.

**Diagnosis (Level 3).** Body shape ovate to elliptical, dorsoventrally flattened (Fig. 129A, F); epidermis with radial or transverse dorsal ridges (Fig. 129A, C, F); first segment achaetous; notopodial lobes as long dorsal ridges (Fig. 129A, C); spines present (Fig. 129D).

**Description.** See Suppl. material 1.

**Remarks.**Spintheridae is represented by a single genus, *Spinther* Johnston, 1845, and 11 species (WoRMS 2025). Family members are ectoparasites or commensals of sponges. *Spinther* has a widespread but patchy distribution, with some continents lacking records altogether (GBIF.org 2023). Parapar et al. (2012) provide a key to European taxa, and Yamamoto and Imajima (1985) provide a table with distinguishing morphological features of all species.

**Environment and habitat.** Aquatic, marine; coastal or continental shelf; epizoic.

#### ﻿Spionidae Grube, 1850, sensu stricto [polychaete]

Fig. 130

**Common name.** Mud-blister worms, mud worms (*Polydora*).

**LSID.** Urn:lsid:marinespecies.org:taxname:913.

**Diagnosis (Level 3).** Body shape elongate, more-or-less equal width along entire length (Fig. 130A, I); prostomium conical, tapering to slender tip, triangular to trapezoidal (narrow end posteriorly), or T-shaped, wide end anteriorly (Fig. 130A, B, I); nuchal organs present, single antenna-like projection on posterior prostomium sometimes accompanied by a posteriorly projecting caruncle (Fig. 130A, car); palps present (Fig. 130A, B, I, gpa); peristomium a single collar-like ring (Fig. 130A); hooks present (Fig. 130E); branchiae present (Fig. 130A, B, I).

**Description.** See Suppl. material 1.

**Remarks.**Spionidae sensu stricto is a species-rich family with 40 genera and 685 species (WoRMS 2025), with a global distribution. In line with POLiKEY (Glasby and Fauchald 2003) and WoRMS we present a narrow (sensu stricto) concept of the family, which does not include Poecilochaetidae, Trochochaetidae, and Uncispionidae as has recently been recently suggested (Rouse et al. 2022). Although this sensu stricto concept may not be defined by synapomorphy, it is nevertheless distinguishable from the other three families at DELTA Diagnostic Level 2. Radashevsky (2012) provides a key to genera of UK waters, Gil (2011) provides a key to European taxa, Radashevsky (2015) provides a key to the spionid genera reported from or likely to be found on the Great Barrier Reef, Australia, and Sikorski and Pavlova (2015) provide a key for *Scolelepis* Blainville, 1828 from the Atlantic sector of the Arctic. Meißner and Hutchings (2003) provide a key to *Spiophanes* Grube, 1860 species of eastern Australia, Bick (2005) provides a key for all *Marenzelleria* Mesnil, 1896 species and Radashevsky (2021) provides a key to *Pseudopolydora* Czerniavsky, 1881 of Europe and adjacent waters. Spionidae in Australia are represented by a relatively high proportion of non-native species (14 species), which can be identified using the online key of Kupriyanova et al. (2013).

**Environment and habitat.** Aquatic, marine or brackish (very rarely freshwater); coastal, continental shelf or deep sea; soft or hard substrata, hydrothermal vents and cold seeps, or epizoic (on mollusk shells).

#### ﻿Sternaspidae Carus, 1863 [polychaete]

Fig. 131

**Common name.** Mud owls.

**LSID.** Urn:lsid:marinespecies.org:taxname:974.

**Figures 131, 132.**
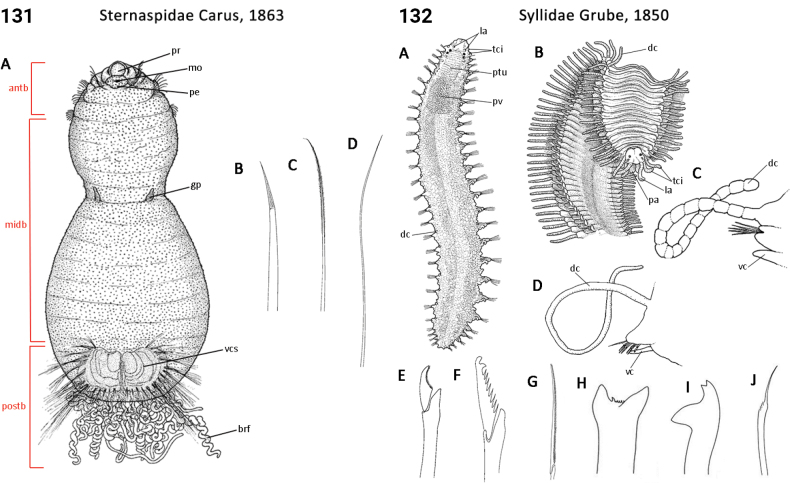
Distinguishing features: **131.**Sternaspidae: **A–D.***Sternaspisscutata*: **A.** Entire animal ventral view; **B.** Spine from anterior body; **C, D.** Pilose capillary chaeta (**C**) and smooth capillary chaeta (**D**), posterior body. Abbreviations: antb anterior body brf branchial filaments gp genital papilla midb mid body mo mouth pe peristomium postb posterior body pr prostomium vcs ventrocaudal shield. Sources: **A–D** derivatives of fig. 1.120 Beesley et al. (2000). **132.**Syllidae: **A.** Entire animal of *Sphaerosyllis* sp. (Exogoninae) dorsal view; **B.** Anterior end of *Trypanosyllis* sp. median antenna absent; **C.** Parapodium from midbody of *Sylline* sp.; **D.** Parapodium from midbody of *Eusylline* sp.; **E.** Heterogomph falciger from *Trypanosyllis* sp.; **F.** Heterogomph falciger from *Eusylline* sp.; **G.** Spinigerous chaeta *Syllisgarciai*; **H.** Simple chaeta *Sy.gracilis*; **I.** Simple chaeta *Haplosyllisspongicola*; **J.** Dorsal simple bayonet-shaped chaeta *Myrianidaquindecimdentata*. Abbreviations: dc dorsal cirrus la lateral antenna pa palp ptu pharyngeal tube pv proventricle tci tentacular cirri vc ventral cirrus. Sources: **A–F** derivatives of fig. 1.90 Beesley et al. (2000), **G–J** derivatives of fig. 8 San Martín and Aguado (2022).

**Diagnosis (Level 3).** Body peanut-shaped, segmentation present; discrete head present, retractable into anterior segments (Fig. 131A); ventrocaudal shield on posterior segments present (Fig. 131A, vcs).

**Description.** See Suppl. material 1.

**Remarks.**Sternaspidae contains four genera and 48 species (WoRMS 2025). The family is globally distributed but appears to be absent, or rare, in the deep sea (GBIF.org 2023). Sendall and Salazar-Vallejo (2013) revised the genus *Sternaspis* Otto, 1820 and provided keys and species descriptions. Gil (2011) and Salazar-Vallejo (2017b) provide keys to differentiate genera and species from Europe and the Tropics, respectively. Plathong et al. (2021b) provide a key for the identification of all species of *Petersenaspis* Sendall & Salazar-Vallejo, 2013.

**Environment and habitat.** Aquatic, marine; coastal, continental shelf or deep sea; soft substrata.

#### ﻿Syllidae Grube, 1850 [polychaete]

Fig. 132

**Common name.** None.

**LSID.** Urn:lsid:marinespecies.org:taxname:948.

**Diagnosis (Level 1).** Tentacular cirri present; pharynx proventricle present (Fig. 132A, B, pv); parapodia lacking notopodial lobes (Fig. 132C, D), capillary chaetae present (Fig. 132J).

**Description.** See Suppl. material 1.

**Remarks.**Syllidae is one of the most taxon-rich families of Polychaeta, with 92 valid genera and 1119 species (WoRMS 2025). Five subfamilies are currently recognised: Anoplosyllinae, Autolytinae, Eusyllinae, Exogoninae, and Syllinae (WoRMS 2025). The family is similar to Sphaerodoridae and only distinguishable at DELTA Diagnostic Level 1. San Martín (2003), Gil (2011) and San Martín and Worsfold (2015) provide keys to European taxa. San Martín (2005), San Martín and Hutchings (2006), San Martín et al. (2008) and Lattig et al. (2010) provide keys to genera and species of Australian Syllidae, and San Martín et al. (2013) provide a key to all species of *Branchiosyllis* Ehlers, 1887.

**Environment and habitat.** Aquatic, marine; coastal or continental shelf or deep sea; soft or hard substrata, or epizoic/endozoic (especially Anthozoa, decapods, echinoderms, and sponges).

#### ﻿Syngenodrilidae Smith & Green, 1919 [microdrile]

Fig. 133

**Common name.** None.

**LSID.** Urn:lsid:marinespecies.org:taxname:1040002.

**Figures 133, 134.**
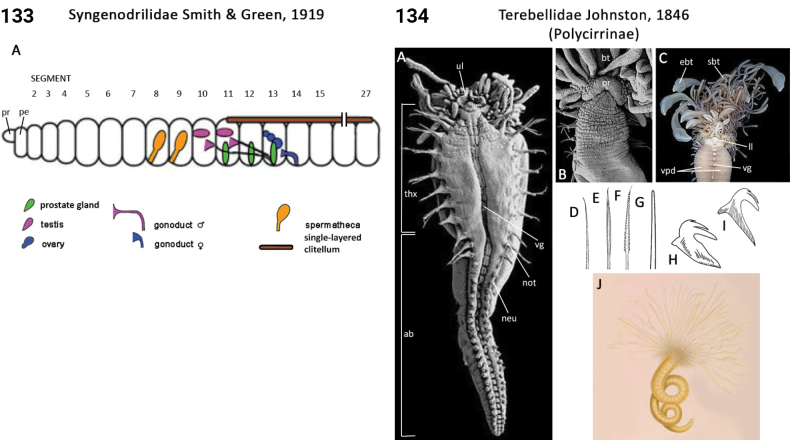
Distinguishing features: **133.**Syngenodrilidae: **A.** Diagram of reproductive organs, dorsal side up. Abbreviations: pe peristomium pr prostomium. Sources: **A** derivative of fig. 8.4A Jamieson (2006). **134.**Terebellinae, Polycirrini: **A, B.***Polycirruspapillatus*: **A.** Entire animal ventral view; **B.** Anterior end, dorsal view; **C.***Amaeanabrasiliensis* ventral view; **D–F.** Notochaetae: **D.***Amaeanaapheles*; **E.***Lysillabicrinalis*; **F.***Lysillabilobata*; **G.***Amaeana* sp. neurochaeta; **H.***P.californicus* uncinus from posterior chaetiger; **I.***P.coccineus* uncinus from posterior chaetiger; **J.***P.medusa* entire animal. Abbreviations: ab abdomen ebt expanded buccal tentacle ll lower lip neu neuropodium not notopodium pr prostomium sbt short buccal tentacle thx thorax ul upper lip vg ventral groove vpd ventral pads. Sources: **A–C** derivatives of figs 1, 2, 3 Hutchings et al. (2021b), **D–G** derivatives of fig. 1.121 Beesley et al. (2000), **H, I** derivatives of Glasby and Hutchings (2014), figs 13, 17, **J** derivative of MacIntosh (1900–1922), pl. CXIIIA, fig. 3.

**Diagnosis (Level 3).** Gizzard present; hair chaetae absent; clitellum situated in region of male pores; tubercula pubertatis form paired ridges on the ventrolateral margins of the clitellum; testes, two pairs; male pores two or more segments following testicular segment (opisthoporous) (Fig. 133A); spermathecal pores two pairs, located near males and female pores; prostate gland present, > 1 pair (Fig. 133A).

**Description.** See Suppl. material 1.

**Remarks.**Syngenodrilidae comprises a single genus and species from Africa (WoRMS 2025). Like their sister group, Alluroididae, they share several reproductive features in common with earthworms (Crassiclitellata), but have a single-celled clitellum like other microdriles (Jamieson 2006; Timm 2012). They are tentatively treated as microdriles because of the form of the clitellum, but can be distinguished from most microdriles by their lack of hair chaetae.

**Environment and habitat.** Terrestrial.

#### ﻿Terebellidae Johnston, 1846 sensu lato [polychaete]

**Common name.** Spaghetti worms (general name for members of the family).

**LSID.** Urn:lsid:marinespecies.org:taxname:982.

**Diagnosis (Level 1).** Thoracic ventral glandular areas present, with mid-ventral shield-shaped swellings; hooks absent.

**Description.** See Suppl. material 1.

**Remarks.**Terebellidae sensu lato is a large family comprising 65 genera and 653 species (WoRMS 2025); it has a widespread distribution and shows a wide range of morphologies, which have been documented in Nogueira et al. (2010). The family is similar to Trichobranchidae and only distinguishable at DELTA Diagnostic Level 1. In order to reduce polymorphism in the dataset we have coded subfamilies (and, in one case, tribe). The subfamilies (and tribe, Polycirrini) were treated as families in Nogueira et al. (2013), and this position was maintained by Hutchings et al. (2021a, b). Note that the terebelline tribes Lanicini, Procleini, and Terebellini (sensu Stiller et al. (2020)) as invoked in WoRMS, are not coded because they cannot be distinguished based on the current dataset. Similarly, Telothelepodinae (or Telothelepodidae sensu Nogueira et al. (2013)) could not be uniquely coded based on the present dataset. Holthe (1986) provides a key to world genera at the time, and Gil (2011) and Lavesque et al. (2021a, b) provide keys to European taxa. Nogueira et al. (2015) provide keys to terebellid taxa from Lizard Island, Great Barrier Reef, Australia and Jirkov and Leontovich (2013) provide a key to Terebellomorpha, including Pectinariidae, from the eastern Atlantic and the North Polar seas.

**Environment and habitat.** Aquatic, marine; coastal, continental shelf or deep sea; soft or hard substrata, or epizoic (algal holdfasts and seagrass).

#### ﻿Terebellidae, Terebellinae, Polycirrini Malmgren, 1866 [polychaete]

Fig. 134

**LSID.** Urn:lsid:marinespecies.org:taxname:181512.

**Diagnosis (Level 1).** Thoracic ventral glandular areas present as distinct paired ventrolateral swellings (Fig. 134A, C, vg, vpd); branchiae absent (Fig. 134B, J); not tube-dwelling.

**Description.** See Suppl. material 1.

**Remarks.** The present tribal classification of Polycirrini is based on Stiller et al. (2020), and follows the classification presented in WoRMS (2025). This taxon is considered a family by Nogueira et al. (2013). Polycirrini comprises six genera and 122 species (WoRMS 2025). Lavesque et al. (2021b) provide keys to European members of the tribe, which they refer to as a family (Polycirridae) and Glasby and Hutchings (2014) provide a key to world *Polycirrus* Grube, 1850 species. See Remarks under Terebellidae sensu lato for more references having identification keys.

**Environment and habitat.** Aquatic, marine; coastal, continental shelf or deep sea; soft or hard substrata, or occasionally holopelagic (*Biremis*).

#### ﻿Terebellidae, Terebellinae Johnston, 1846 (excluding Polycirrini) [polychaete]

Fig. 135

**LSID.** Urn:lsid:marinespecies.org:taxname:322588.

**Figures 135, 136.**
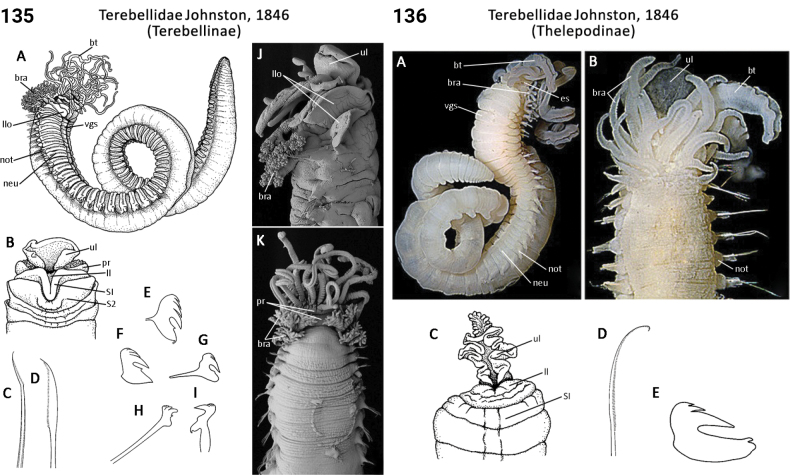
Distinguishing features: **135.**Terebellidae, Terebellinae: **A.***Longicarpusmodestus* entire animal; **B.***Pistaaustralis* head end ventral view; **C.***Terebellamaculata* notochaetae; **D.***Lanassaocellata* notochaetae; **E–I.** Neurochaetae: **E.***Loimia* sp.; **F.***Terebella* sp.; **G.***Pista* sp.; **H.***Lanicides* sp.; **I.***Longicarpus* sp.; **J.***Loimiapseudotriloba* lateral view; **K.***Nicoleavaili* dorsal view. Abbreviations: bra branchia bt buccal tentacles ll lower lip llo lateral lobe neu neuropodium not notopodium pr prostomium SI segment 1 S2 segment 2 ul upper lip vgs ventral glandular shield Sources: **A–I** derivatives of fig. 1.121 Beesley et al. (2000), **J, K** derivatives of fig. 8, fig. 21 Hutchings et al. (2021b). **136.**Terebellidae, Thelepodinae: **A.***Thelepuspaiderotos*, entire animal; **B.***Euthelepusaserrula* anterior end, dorsal view; **C.***Rhinothelepuslobatus* anterior end, ventral view; **D.***Euthelepusserratus* notochaeta; **E.***Euthelepus* sp. neurochaeta. Abbreviations: bra branchia bt buccal tentacles es eye spot ll lower lip neu neuropodium not notopodium SI segment 1 ul upper lip vgs ventral glandular shield. Sources: **A, B** derivatives of figs 1, 6 Hutchings et al. (2021b), **C–E** derivatives of figs 1.121, 1.123 Beesley et al. (2000).

**Diagnosis (Level 2).** Thoracic ventral glandular areas present, as mid-ventral shield-shaped swellings (Fig. 135A, vgs); branchiae present, branching, or multiple filaments arising from a central stalk (Fig. 135A, J, K); uncini arranged in two rows in anterior neuropodia; mostly tube-dwelling.

**Description.** See Suppl. material 1.

**Remarks.** The present subfamily classification of Terebellinae is based on Stiller et al. (2020), and follows the classification presented in WoRMS (2025). It is diagnosable at Level 2, but beyond that, it cannot be differentiated from Thelepodinae. This taxon is considered a family by Noguiera et al. (2013). Terebellinae comprises 49 genera and 396 species (WoRMS 2025); we exclude Polycirrini, which are coded separately, but include the other tribes (Lanicini, Procleini, Terebellini, Artacamini), which are more difficult to distinguish with the current character dataset. Lavesque et al. (2021b) provide keys to European members of the subfamily, which they refer to as a family (Terebellidae). See Remarks under Terebellidae sensu lato for more references having identification keys.

**Environment and habitat.** Aquatic, marine; coastal, continental shelf or deep sea; soft or hard substrata.

#### ﻿Terebellidae, Thelepodinae Hessle, 1917 [polychaete]

Fig. 136

**LSID.** Urn:lsid:marinespecies.org:taxname:181511.

**Diagnosis (Level 1).** Thoracic ventral glandular areas present; as mid-ventral shield-shaped swellings (Fig. 136A, C, vgs); dorsal branchiae simple filaments each arising directly from body wall (Fig. 136A; bra); tube-dwelling.

**Description.** See Suppl. material 1.

**Remarks.** The present subfamily classification of Thelepodinae is based on Stiller et al. (2020) and follows the classification presented in WoRMS (Dec 2025). This taxon is presented as two families (Thelepodidae and Telothelepodidae) in Nogueira et al. (2013), but they could not be separated using the present character set. Thelepodinae is similar to Terebellinae and only distinguishable at DELTA Diagnostic Level 1. Thelepodinae comprises ten genera and 131 species (WoRMS 2025). Lavesque et al. (2020) provide keys to the European species. See Remarks under Terebellidae sensu lato for more references having identification keys.

**Environment and habitat.** Aquatic, marine; coastal, continental shelf or deep sea; soft or hard substrata, or epizoic.

#### ﻿Thalassematidae Forbes & Goodsir, 1841 [echiuran]

**Common name.** Spoon worms, anchor worms, fat innkeeper worms (general name for members of the family).

**LSID.** Urn:lsid:marinespecies.org:taxname:110348

**Diagnosis (Level 3).** Body regionalized (sausage or grub-shaped body and ribbon-like proboscis), segmentation absent; proboscis non-retractable; gut straight except for a large mid-body loop; anus positioned posteriorly, but pygidium absent.

**Description.** See Suppl. material 1.

**Remarks.**Thalassematidae sensu lato comprise five subfamilies, 40 genera and 175 species (WoRMS 2025). Thalassematidae has a wide range of body forms so we have provided subfamily coding to reduce polymorphism in the dataset. Further, the group has long been recognised at the rank of phylum, viz., Echiura, with the current five subfamilies treated as families, so there is substantial family-level taxonomic literature, some including keys to genera and species. The family has a widespread global distribution; subfamily members appear to have a preference for particular latitudes or ocean basins, but this may well reflect patchy sampling. Edmonds (2000) provides a key to the five subfamilies (then families); Saiz Salinas et al. (2000) provide a key to Antarctic taxa. Biseswar (2009) provides keys for the identification of genera and species of echiurans from the Atlantic, and Biseswar (2010) provides a checklist of Indo-west Pacific taxa.

**Environment and habitat.** Aquatic, marine or brackish; coastal, continental shelf or deep sea; soft or hard substrata.

#### ﻿Thalassematidae, Bonelliinae Lacaze-Duthiers, 1858 [echiuran; alternative representation ‘Bonelliidae’]

Fig. 137

**LSID.** Urn:lsid:marinespecies.org:taxname:110347.

**Figures 137, 138.**
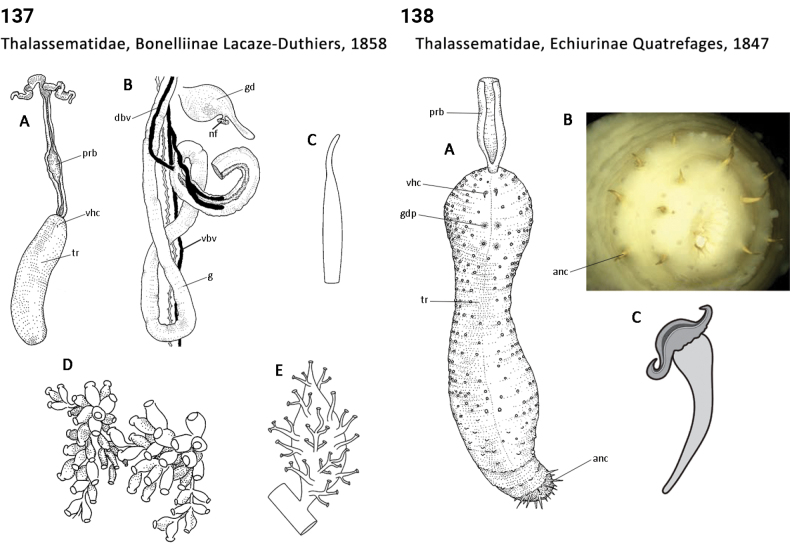
Distinguishing features: **137.**Thalassematidae, Bonelliinae: *Metabonelliahaswelli*; **A.** Ventral view of entire animal with proboscis extended; **B.** Internal organs of anterior region of trunk; **C.** Ventral hook chaeta; **D.** Section of anal vesicle; **E.***Bonelliaviridis* general schema of gonoduct. Abbreviations: dbv dorsal blood vessel g gut gd gonoduct nf nephrostomal funnel prb proboscis tr trunk vbv ventral blood vessel vhc ventral hook chaeta. Sources: **A–D** after fig. 4.15 Beesley et al. (2000), **E** after fig. 7.6.5.9 Dohren (2019). **138.**Thalassematidae, Echiurinae: **A.***Echiurusechiurus* ventral view of entire animal; **B.** Posterior end; **C.** Schema of gonoduct. Abbreviations: anc anal chaeta gdp gonoduct pore prb proboscis tr trunk vhc ventral hook chaeta. Sources: **A** after fig. 4.11 Beesley et al. (2000), **B** after fig. 7.6.5.6 **C** after fig. 7.6.5.11 Dohren (2019).

**Diagnosis (Level 0).** Elongate, bifid proboscis (Fig. 137A).

**Description.** See Suppl. material 1.

**Remarks.**Bonelliinae comprise 30 genera and 78 species (WoRMS 2025; recently elevated to family Bonelliidae), having a widespread global distribution at all depths. Bonelliinae is not diagnosable ((DELTA Diagnostic Level 0), being inseparable from other Thalassematidae using the present character set. The subfamily displays sexual dimorphism and, as such, this dataset has only been scored for females. The dwarf males live on or inside the body of the female; they lack pigmentation and a proboscis and have a rudimentary gut, and are thus very different from the present description. See Remarks under Thalassematidae sensu lato for references having regional keys that include Bonelliinae.

**Environment and habitat.** See Thalassematidae

#### ﻿Thalassematidae, Echiurinae Quatrefages, 1847 [echiuran; alternative representation ‘Echiuridae’]

Fig. 138

**LSID.** Urn:lsid:marinespecies.org:taxname:110349.

**Diagnosis (Level 2).** Truncate proboscis (Fig. 138A, prb); spine chaetae present posteriorly, arranged in two rings (Fig. 138A, B; anc).

**Description.** See Suppl. material 1.

**Remarks.**Echiurinae comprises a single genus, *Echiurus* Guérin-Méneville, 1831 with four species having a wide distribution, mainly at high latitudes (GBIF.org 2023; WoRMS 2025). It is diagnosable at DELTA level 2, but thereafter indistinguishable from Ikedinae and Thalassematidae. Saiz Salinas et al. (2000) provide a key to Antarctic taxa. Biseswar (2019) provides a key for the identification of all genera and species of southern Africa (all belong to Echiurinae).

#### ﻿Thalassematidae, Ikedinae Bock, 1942 [echiuran; alternative representation ‘Ikedidae’]

Fig. 139

**LSID.** Urn:lsid:marinespecies.org:taxname:366284.

**Figures 139, 140.**
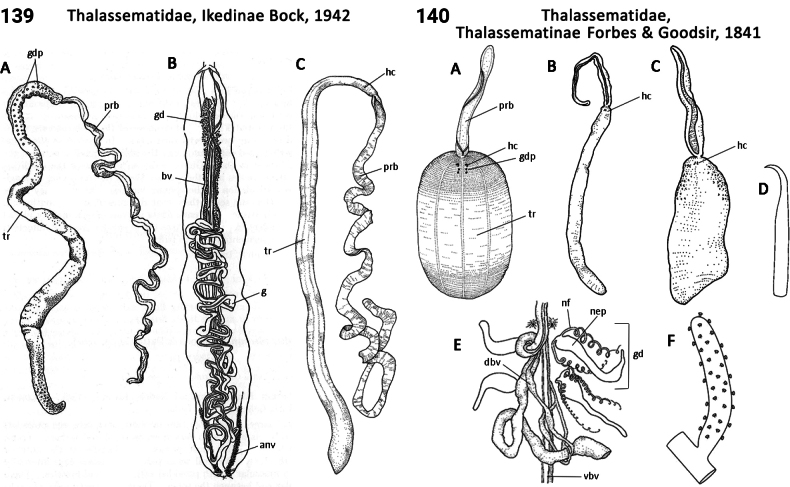
Distinguishing features: **139.**Thalassematidae, Ikedinae: **A.***Ikedapirotansis* entire animal; **B, C.***Ikedataenioides*: **C.** Dissected female specimen; **B.** Ventral view of entire animal. Abbreviations: anv anal vescicle bv blood vessel g gut gd gonoduct gdp gonoduct pore hc hook chaeta prb proboscis tr trunk. Sources: **A, B** after fig. 58, Stephen and Edmonds (1972), **C** after fig. 4.19 Beesley et al. (2000). **140.**Thalassematidae, Thalassematinae: **A.** External morphology of *Listriolobuspelodes* ventral view; **B.***Arhynchitehiscocki*; **C.***Thalassemasydniense*; **D.***Ochetostomaaustraliense* vental hook chaeta; **E.** Generalised internal organs of the anterior region of trunk; **F.***T.thalassema* general schema of anal vescicle. Abbreviations: nf nephrostomal funnel dbv dorsal blood vessel gd gonoduct gdp gonoduct pore hc hook chaeta nep nephridium prb proboscis tr trunk vbv vental blood vessel. Sources: **A** after fig. 4.2, **B, C** after fig. 4.12, **D** after fig. 4.13, **E** after fig. 4.14 Beesley et al. (2000), **F** after fig. 7.6.5.9 Dohren (2019).

**Diagnosis (Level 0).** Proboscis very long (Fig. 139A, C); and chaetae (hook chaetae) only present anteriorly (Fig. 139C, hc).

**Description.** See Suppl. material 1.

**Remarks.**Ikedinae comprises a single genus, *Ikeda* Wharton, 1913 and two species, which have an Indo-west Pacific distribution (GBIF.org 2023; WoRMS 2025, recently elevated to family Ikedidae). Using our dataset, Ikedinae is not diagnosable (DELTA Diagnostic Level 0), being inseparable from other Thalassematidae. Goto et al. (2020), using molecular data, advocate for the inclusion of *Ikeda* (the only genus of Ikedinae) within Bonelliidae.

**Environment and habitat.** See Thalassematidae

#### ﻿Thalassematidae,Thalassematinae Forbes & Goodsir, 1841 [echiuran; alternative representation ‘Thalassematidae’]

Fig. 140

**LSID.** Urn:lsid:marinespecies.org:taxname:234514.

**Diagnosis (Level 0).** Proboscis truncate, very long (Fig. 140A–C), and anal spines absent.

**Description.** See Suppl. material 1.

**Remarks.**Thalassematidae comprises seven genera and 85 species (WoRMS 2025), and has a widespread global distribution. Using the present dataset, Thalassematinae is not diagnosable (DELTA Diagnostic Level 0), being inseparable from the other four subfamilies. Biseswar (1984) provides a key to species of the large genus *Anelassorhynchus* Annandale, 1922. See Remarks under Thalassematidae sensu lato for references having regional keys that include Thalassematidae.

**Environment and habitat.** See Thalassematidae.

#### ﻿Thalassematidae, Urechinae Monro, 1927 [echiuran; alternative representation ‘Urechidae’]

Fig. 141

**LSID.** Urn:lsid:marinespecies.org:taxname:255181.

**Figures 141, 142.**
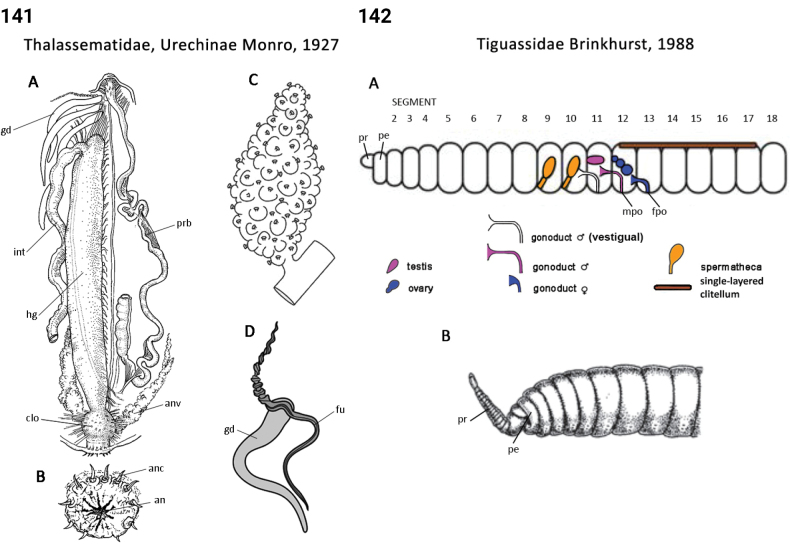
Distinguishing features: **141.**Thalassematidae, Urechinae: **A.***Urechiscaupo* disected trunk; **B.** Circlet of anal chaetae *Urechis* sp. **C.** Anal vesicle general schema *U.caupo*; **D.** Schema of gonoduct *Urechis* sp. Abbreviations: anc anal chaeta anv anal vescicle clo cloaca fu funnel gd gonoduct hg hindgut int intestine prb proboscis. Sources: **A, B** after fig. 4.18 Beesley et al. (2000), **C** after fig. 7.6.5.9 and **D** after fig. 7.6.5.11 of Dohren (2019). **142.**Tiguassidae: **A.** Schematic image of reproductive organs, dorsal side up; **B.***Tiguassureginae* showing proboscis-like prostomium. Abbreviations: fpo female pore mpo male pore pe peristomium pr prostomium. Sources: **A, B** derivatives of fig. 8.4 A, 8.28 Jamieson (2006).

**Diagnosis (Level 1).** Proboscis short and scoop-like; chaetae only present anteriorly (Fig. 141B; an).

**Description.** See Suppl. material 1.

**Remarks.**Urechinae comprises a single genus, *Urechis* Seitz, 1907 and four species (WoRMS 2025, recently elevated to family Urechidae); it appears to have largely a ‘Pacific rim’ distribution, with only a few records in the Atlantic of Argentina (GBIF.org 2023). The family is diagnosable at DELTA Diagnostic Level 1, but indistinguishable from other Thalassematidae at higher levels. Goto et al. (2020) suggested its synonymy with Echiurinae, on the basis of molecular similarity and their shared arrangement of anal chaetae in rings. See Remarks under Thalassematidae sensu lato for references having regional keys that include Urechinae.

**Environment and habitat.** See Thalassematidae.

#### ﻿Tiguassidae Brinkhurst, 1988 [microdrile]

Fig. 142

**Common name.** None.

**LSID.** Urn:lsid:marinespecies.org:taxname:1040003.

**Diagnosis (Level 2).** Prostomium anteriorly with an anterior tentacle-like extension (‘proboscis’) (Fig. 142B); chaetae arranged in closely spaced lateral and ventrolateral pairs (lumbricine arrangement); clitellum situated in region of male pores; testes, one pair; spermathecae pre-testicular; spermathecal pores, two pairs, located within one or two segments of male pores (Fig. 142A); pygidium absent.

**Description.** See Suppl. material 1.

**Remarks.**Tiguassidae is a monotypic family, known for *Tiguassureginae* Righi, Ayres & Bittencourt, 1978 of the Amazon region, Brazil (WoRMS 2025). Little is known about its biology, including the form of its clitellum, which has made placement within microdriles or megadriles difficult. Although its position within Oligochaeta is currently incertae sedis (Jamieson 2006; Martin et al. 2024), we have assumed it belongs within the microdriles, based on the similarity of its reproductive system with haplotaxids (Jamieson 2006), and therefore that the clitellum is thin and single-celled as is usual for microdriles.

**Environment and habitat.** Aquatic, freshwater.

#### ﻿Tomopteridae Grube, 1850 [polychaete]

Fig. 143

**Common name.** Gossamer worms.

**LSID.** Urn:lsid:marinespecies.org:taxname:958.

**Figures 143, 144.**
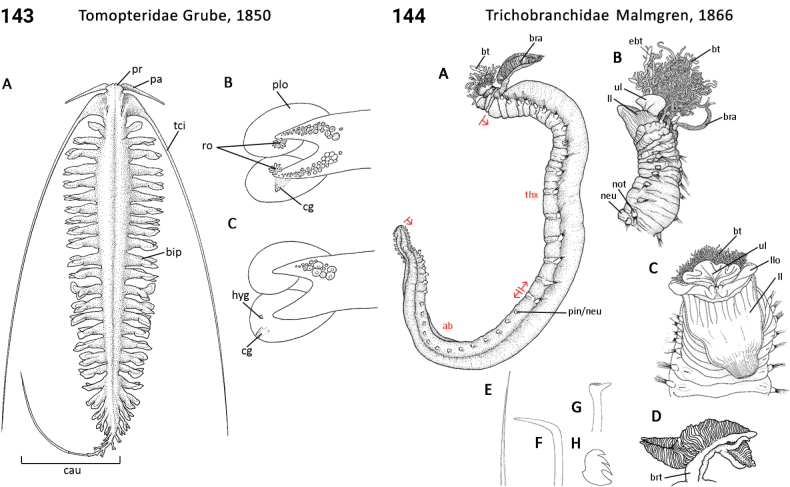
Distinguishing features: **143.**Tomopteridae: **A.***Tomopterisaustraliensis* entire animal, dorsal view; **B.***Tomopterishelgolandica* parapodium, chaetae not shown; **C.***Tomopterisseptentrionalis* parapodium, chaetae not shown. Abbreviations: bip biramous parapodium cau caudal region cg chromophile glands hyg hyaline gland pa palp plo parapodial lobe pr prostomium ro rosette organ tci tentacular cirri. Sources: **A** derivative of fig. 1.91 Beesley et al. (2000), **B, C** derivative of fig. 25 Pettibone (1963). **144.**Trichobranchidae: **A.***Terebellides* sp. entire animal dorsolateral view; **B, C.***Trichobranchus* sp. anterior end showing inflatable lower lip; **B.** Lateral view; **C.** Antero-ventral view; **D.***Terebellidesstroemii* four lobed branchia; **E.***Terebellides* sp. notochaetae, thoracic capillary chaeta; **F–H.***Terebellides* sp. neurochaetae: **F.** Spine from parapodia of chaetiger 1; **G.** Anterior acicular thoracic hook; **H.** Anterior abdominal uncinus. Abbreviations: bra branchia brt branchial trunk bt buccal tentacles ebt expanded buccal tentacle ll lower lip llo lateral lobe neu neuropodium not notopodium pin pinnule ul upper lip. Sources: **A–H** derivatives of fig. 1.123, fig. 1.124 Beesley et al. (2000).

**Diagnosis (Level 3).** In life, body translucent, gut visible; ventral groove absent; tentacular cirri present (Fig. 143A, tci); postcephalic eyes present; parapodia present, notopodial and neuropodial lobes elongate, ending in rounded lappets (Fig. 143B, C).

**Description.** See Suppl. material 1.

**Remarks.**Tomopteridae is a holopelagic family comprising three monotypic genera and a fourth, *Tomopteris* Eschscholtz, 1825, with 51 species (WoRMS 2025). The family is globally distributed in oceanic and coastal waters, mostly in the top few hundred metres. They are easily recognizable as live specimens by their translucent body and they are the only holopelagic polychaete to lack external chaetae. Dales (1957) provides keys to genera and species of ﻿Tomopteridae of the Pacific Ocean, Pleijel and Dales (1991) provide a key to European taxa, O’Sullivan (1982) provides a key to Southern Ocean taxa, and Jiménez-Cueto and Suárez-Morales (1999) provide a key to the Caribbean species of *Tomopteris*.

**Environment and habitat.** Aquatic, marine; coastal, continental shelf or deep sea; holopelagic (from surface to a few hundred meters mainly).

#### ﻿Trichobranchidae Malmgren, 1866 [polychaete]

Fig. 144

**Common name.** None.

**LSID.** Urn:lsid:marinespecies.org:taxname:983.

**Diagnosis (Level 1).** Thoracic ventral glandular areas present (Fig. 144A); hooks present (Fig. 144G).

**Description.** See Suppl. material 1.

**Remarks.**Trichobranchidae contains three genera and 118 species (WoRMS 2025), and has a widespread distribution. Trichobranchidae was originally erected as a subfamily within Terebellidae and this same position within Terebellidae was also found by Nogueira et al. (2013) (along with families Ampharetidae, Melinnidae and Pectinariidae) using a morphological cladistic approach; however, the recent finding of Stiller et al. (2020) that Trichobranchidae is an independent family (based on integrative phylogenetic study) has corroborated most phylogenetic studies during the last 30 years or so. Nevertheless, using the present morphological data Trichobranchidae is not diagnosable from Terebellidae at DELTA Diagnostic Level 2 and only diagnosable from other polychaete families at Level 1. Holthe (1986) and Gil (2011) provide keys to European taxa, Hutchings and Peart (2000) provide a key to Australian taxa, and Lavesque et al. (2019) provide a key to Trichobranchidae of France. The genus *Terebellides* Sars, 1835 with over 90 species worldwide, has received the most attention from taxonomists. Schüller and Hutchings (2013) provide a key to all known *Terebellides* at the time; Parapar et al. (2013) provide a key to Adriatic *Terebellides* species; Parapar et al. (2016) provide a key to *Terebellides* species in SE Indo-Pacific waters; Parapar et al. (2020a) provide a key to *Terebellides* species in the NE Atlantic and Barroso et al. (2022) provide an updated identification key to all described species of *Terebellides* from the NE Atlantic. Zhang and Hutchings (2018) provide a key to *Terebellides* from the NW Pacific, and Lavesque et al. (2024) provide a key to *Terebellides* species of the Central Indo-Pacific.

**Environment and habitat.** Aquatic, marine; coastal, continental shelf or deep sea; soft substrata.

#### ﻿Tritogeniidae Plisko, 2013 [megadrile]

Fig. 145

**Common name.** Common stumpy earthworms.

**LSID.** Urn:lsid:marinespecies.org:taxname:1060959.

**Figures 145, 146.**
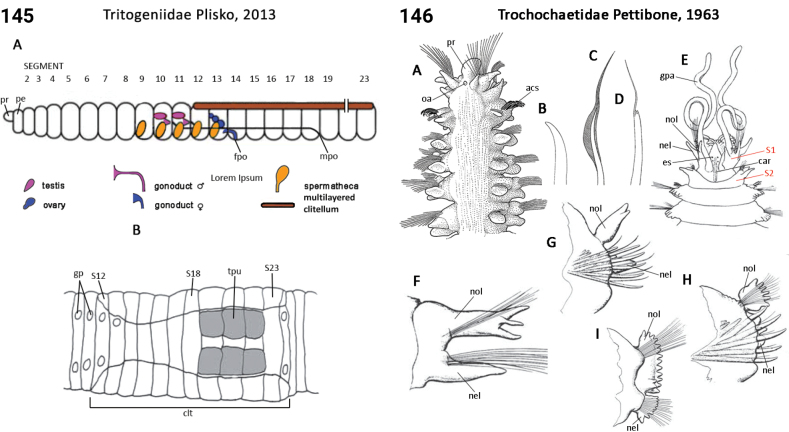
Distinguishing features: **145.**Tritogeniidae: **A.** Schematic image of reproductive organs, dorsal side up; **B.***Michalakusinitus* genital markings. Abbreviations: clt clitellum gp genital papilla fpo female pore mpo male pore pe peristomium pr prostomium S segment tpu tubercular pubertatis. Sources: **A** adapted from information in Plisko (2013), **B** derivative 8.35B Jamieson (2006). **146.** Trochochaetide: **A–D.***Trochochaeta* sp.: **A.** Anterior end dorsolateral view palps missing; **B–D.** Chaetae: **B.** Spine from neuropodium of chaetiger 3; **C.** Winged capillary from parapodium of chaetiger 5; **D.** Aristate neurochaeta from parapodium of chaetiger 19; **E.***Trochochaetafranciscana* anterior end dorsal view; **F–H.***T.franciscana* parapodia: **F.** Chaetiger 1; **G.** Chaetiger 2; **H.** Chaegiter 3; **I.** Chaetiger 4. Abbreviations: acs acicular spine car caruncle es eye spot gpa grooved palp nel neuropodial lobe nol notopodial lobe oa occipital antenna pr prostomium SI segment 1 S2 segment 2. Sources: **A–D** derivatives of fig. 1.109 Beesley et al. (2000), **E** derivative of fig. 7.4.3.1 Beesley et al. (2000), **F, G** derivatives of fig. 7.4.3.2 Blake and Maciolek (2019c).

**Diagnosis (Level 1).** Spermathecal pores, 5 pairs (Fig. 145A); gonadal segments bearing genital papillae absent (genital papillae usually anterior to clitellum; Fig. 145B); prostate gland absent.

**Description.** See Suppl. material 1.

**Remarks.**Tritogeniidae, which was erected by Plisko (2013), includes two genera, *Michalakus* Plisko, 1996 with a single species and *Tritogenia* Kinberg, 1866 with 38 species; all species are endemic to southern Africa (Misirlioğlu et al. 2023 and references therein). Plisko (1997) includes a key to species and species groups of *Tritogenia* from southern Africa, and Plisko and Nxele (2015) provide a key to distinguish foreign Tritogeniidae taxa from native ones of South Africa. Tritogeniidae is similar to both Hormogastridae and Sparganophilidae and is only distinguishable at DELTA Diagnostic Level 1.

**Environment and habitat.** Terrestrial.

#### ﻿Trochochaetidae Pettibone, 1963 [polychaete]

Fig. 146

**Common name.** None.

**LSID.** Urn:lsid:marinespecies.org:taxname:915.

**Diagnosis (Level 3).** Body regionalization present; prostomium triangular to trapezoidal (narrow end posteriorly); palps present (Fig. 146E, gpa); peristomium as a single collar-like ring; first chaetiger with slender elongate chaetae forming a cage around the head (Fig. 146A, E); hooks absent.

**Description.** See Suppl. material 1.

**Remarks.**Trochochaetidae is maintained here as a family-level taxon following POLiKEY (Glasby and Fauchald 2003) and WoRMS, despite morphological (including reproductive and larval characters) and molecular evidence that they fall within Spionidae (Blake and Arnofsky 1999; see also references in Rouse et al. 2022). We prefer to wait until conclusive evidence, including greater taxon sampling, before coding an expanded concept of Spionidae (including *Poecilochaetus* Claparède in Ehlers, 1875, *Trochochaeta* Levinsen, 1884 and *Uncispio* Green, 1982), especially because Poecilochaetidae, Trochochaetidae and Uncispionidae are morphologically diagnosable and may prove to be useful concepts for ecological studies. The family contains two genera and 13 species (WoRMS 2025), which have a global distribution but are apparently absent from the deep sea (GBIF.org 2023). Mackie (1990) provides a key to species of Hong Kong, Gil (2011) provides a key to European taxa, while Bochert and Zettler (2013) and Radashevsky et al. (2018) provide keys to all known species of *Trochochaeta* at the time.

**Environment and habitat.** Aquatic, marine; coastal, continental shelf or deep sea; soft substrata.

#### ﻿Tumakidae Righi, 1995 [megadrile]

Fig. 147

**Common name.** None.

**LSID.** Urn:lsid:marinespecies.org:taxname:1060962.

**Figures 147, 148.**
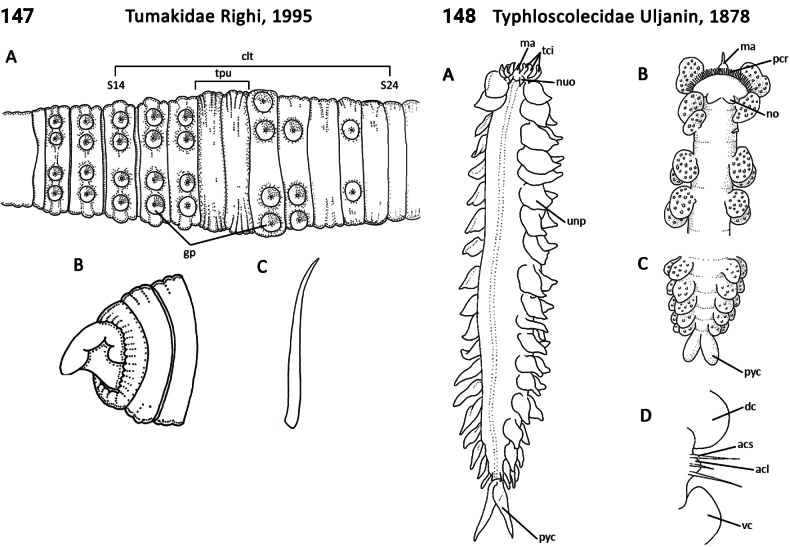
Distinguishing features: **147.**Tumakidae: *Tumakamari*, **A.** Mid-body ventral view showing clitellum and genital field; **B.** Pygidium; **C.** Crotchet chaeta, S segment. Abbreviations: clt clitellum, gp genital papilla, tpu tubercular pubertatis. Sources: **A–C** derivatives of Fig. 2 Celis and Rengel (2015). **148.**Typhloscolecidae: **A.***Travisiopsislanceolata* dorsolateral view of entire animal; **B.** Anterior end; **C.** Posterior end of *Typhloscolexmuelleri* dorsal view; **D.** Uniramous parapodium of *Travisiopsislobifera*. Abbreviations: acl acicular lobe acs acicular spine dc dorsal cirrus ma median antenna nuo nuchal organ pcr prostomial ciliated ridge pyc pygidial cirrus tci tentacular cirri unp uniramous parapodium vc ventral cirrus. Sources: **A–D** derivatives of fig. 1.92, Beesley et al. (2000).

**Diagnosis (Level 3).** Secondary annulation present (Fig. 147A, B); calciferous glands present; gut more-or-less straight, lacking side branches; clitellum situated in the region of the male pores (Fig. 147A); tubercula pubertatis form paired ridges on the ventrolateral margins of the clitellum (Fig. 147A, tpu); gonadal segments bearing genital papillae (Fig. 147A); sperm sac absent; male pores single, median; spermathecal pores, 2 pairs; prostate gland absent.

**Description.** See Suppl. material 1.

**Remarks.**Tumakidae is only known for three species in the genus *Tumak* Righi, 1995 which inhabit the soils of South America (Misirlioğlu et al. 2023 and references therein).

**Environment and habitat.** Terrestrial, soil; soft substrata.

#### ﻿Typhloscolecidae Uljanin, 1878 [polychaete]

Fig. 148

**Common name.** None.

**LSID.** Urn:lsid:marinespecies.org:taxname:959.

**Diagnosis (Level 3).** In life, body translucent, gut visible; prostomium bluntly conical, ciliated (Fig. 148B, pcr); nuchal organs may be visible as paired projections from posterior head (Fig. 148A, B); tentacular cirri present (second segment tentaculate) (Fig. 148A; tci); chaetae appear on 5 or 6^th^ segment after peristomium; spines present (Fig. 148D, acs).

**Description.** See Suppl. material 1.

**Remarks.**Typhloscolecidae is a holopelagic family comprising three genera and 17 species (WoRMS 2025) and has a global distribution occurring from shallow waters down to abyssal depths. Dales (1957) provides keys to genera and species of Typhloscolecidae of the Pacific Ocean; Pleijel and Dales (1991) provide a key to European taxa. O’Sullivan (1982) provides a key to Southern Ocean taxa.

**Environment and habitat.** Aquatic, marine; coastal, continental shelf or deep sea; holopelagic.

#### ﻿Uncispionidae Green, 1982 [polychaete]

Fig. 149

**Common name.** None.

**LSID.** Urn:lsid:marinespecies.org:taxname:249746.

**Figures 149, 150.**
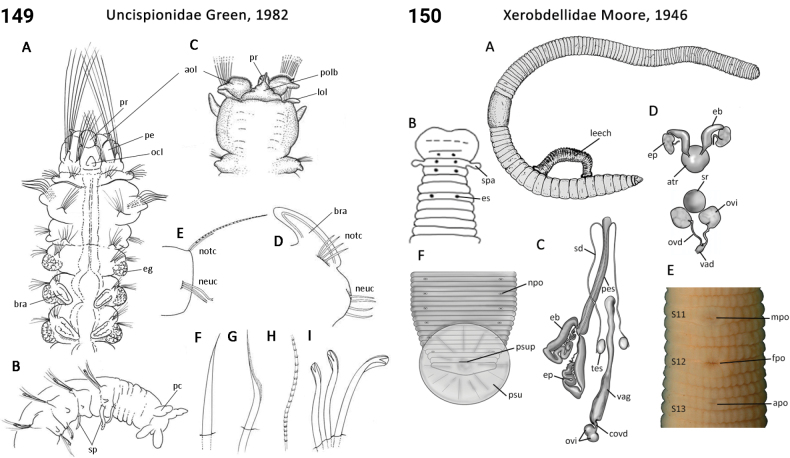
Distinguishing features: **149.**Uncispionidae: **A, B.***Rhamphispiotridentata*: **A.** Anterior end, dorsal view; **B.** Posterior end, lateral view; **C.***Uncopherusapapillata* anterior end ventral view of oral structures; **D, E.***Uncispiohartmanae*: **D.** Chaetiger 6 with branchiae; **E.** Chaetiger 31; **F.** Notopodial spine of chaetiger 1 (*U.papillata*); **G.** Neuropodial fringed capillary of anterior chaetiger (*R.tridentata*); **H.** Spinose notopodial capillary from middle segment (*U.hartmanae*); **I.***U.papillata* hooded hooks. Abbreviations: aol anterior oral lobe bra branchia eg epidermal gland lol lateral oral lobe neuc neurochaetae notc notochaetae ocl occipital lobe pc pygidial cirrus pe peristomium polb posterior oral lobe pr prostomium sp spine. Sources: **A–E** derivatives of fig. 7.4.4.1, **F–I** derivatives of fig. 7.4.4.3 Blake and Maciolek (2019d). **150.**Xerobdellidae: **A.***Xerobdellalecomtei* feeding on earthworm; **B.***Xerobdella* sp., dorsal view showing arrangement of eyes and sensory palps; **C, D.** Male and female reproductive system of *Diestecostomamexicana* (**C**) and *X.lecomtei* (**D**); **E.** Ventral side of segment 11–13 of *X.lecomtei*; **F.***Mesobdellagemmata*, ventral view of posterior end. Abbreviations: apo accessory pore atr atrium covd common oviduct eb ejaculatory bulb ep epididymus es eye spot fpo female pore mpo male pore npo nephridial pore ovi ovisac ovd oviduct pes penis sheath psu posterior sucker psup posterior sucker pore S segment sd sperm duct spa sensory palp sr female seminal receptacle tes testis vag vagina. Sources: **A** after fig. 3 Kutschera et al. (2007), **B** after fig. 3 Mann (1962), **C, D** after fig. 2, **F** after fig. 1 Borda et al. (2008), **E** after fig. 2 Grosser (2020).

**Diagnosis (Level 3).** Prostomium triangular to trapezoidal (narrow end posteriorly), antennae present (Fig. 149C); peristomium a single collar-like ring; parapodia of first chaetiger very elongated, chaetae slender forming a cage around head (Fig. 149A, C); pygidium with multiple digitate lobes (Fig. 149B, pc).

**Description.** See Suppl. material 1.

**Remarks.**Uncispionidae is maintained here as a family-level taxon following POLiKEY (Glasby and Fauchald 2003) and WoRMS, despite morphological evidence that they fall within an expanded Spionidae, including Poecilochaetidae and Trochochaetidae (Blake and Arnofsky 1999; see also references in Rouse et al. 2022); DNA evidence, however, is still lacking. *Uncispio* contains three genera and eight species (WoRMS 2025) having a global distribution but are apparently absent from the deep sea (GBIF.org 2023). Blake and Maciolek (2018) provide a key to all species of Uncispionidae.

**Environment and habitat.** Aquatic, marine; continental shelf or deep sea; soft substrata.

#### ﻿Xerobdellidae Moore, 1946 [leech]

Fig. 150

**Common name.** None.

**LSID.** Urn:lsid:marinespecies.org:taxname:1603422.

**Diagnosis (Level 2).** Body shape more-or-less cylindrical (Fig. 150A); anterior sucker with pair of short lateral cirri (sensory palps) (Fig. 150B); posterior sucker with rays (Fig. 150F); egg sacs globular (Fig. 150C, D, ovi); nephridial pores paired (ventrolateral); male atrium fused (Fig. 150D, atr).

**Description.** See Suppl. material 1.

**Remarks.**Xerobdellidae is a small family belonging to the jawed Hirudiniformes which includes both blood-feeding and invertebrate predatory leeches. The family was resurrected by Borda et al. (2008) to accommodate the monotypic genera *Xerobdella* Frauenfeld, 1868, *Mesobdella* Blanchard, 1893 and *Diestecostoma* Vaillant, 1890, which occur in the Neotropics (*Mesobdella* and *Diestecostoma*) and Palaearctic (*Xerobdella*).

**Environment and habitat.** Terrestrial (mainly), moist terrestrial; epizoic.

#### ﻿Yndolaciidae Støp-Bowitz, 1987 [polychaete]

Fig. 151

**Common name.** None.

**LSID.** Urn:lsid:marinespecies.org:taxname:249688.

**Figure 151.**
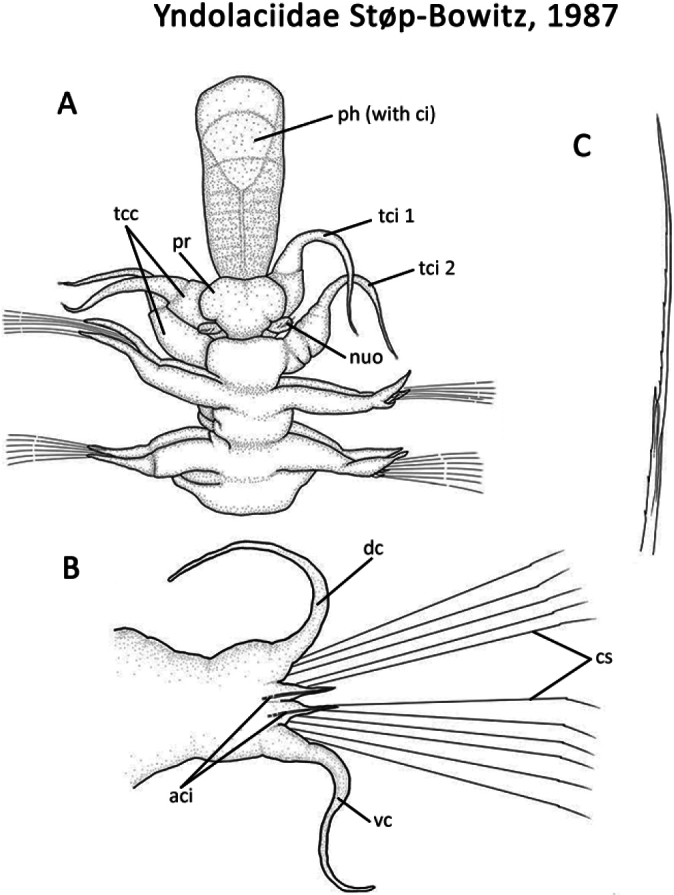
Distinguishing features: Yndolaciidae: **A–C.***Yndolacia* sp.; **A.** Anterior end, pharynx is everted; **B.** Parapodium; **C.** Compound spiniger. Abbreviations: aci aciculae ci cilia cs compound spiniger dc dorsal cirrus nuo nuchal organ ph pharynx pr prostomium tcc tentacular cirri cirrophore tci tentacular cirri vc ventral cirrus. Sources: **A–C** after fig. 1 Zhadan and Tzetlin (2009).

**Diagnosis (Level 3).** Discrete head lobe-like without appendages; prostomium pentagonal to quadrangular, eyes may be present; palps absent; nuchal organs may be obvious posterolateral projections (ciliated bulbs) (Fig. 151A, nuo); tentacular cirri present (Fig. 151A, tci); dorsal cirri present (Fig. 151B, dc); compound chaetae present (Fig. 151C).

**Description.** See Suppl. material 1.

**Remarks.**Yndolaciidae is a holopelagic group comprising three monotypic genera (WoRMS 2025) and is currently only known from the Arctic Ocean and Gulf of Guinea. It is currently considered incertae sedis within Phyllodocida (Rouse et al. 2022).

**Environment and habitat.** Aquatic, marine; deep sea; holopelagic.

## Footnotes

Haplotaxidae s.s. has been characterized based on the type genus Haplotaxis as it was recently split creating Haplotaxidae sensu stricto and four new families (Martin et al. 2024); as this present work was in review, the new families were not able to be included; however, they will be incorporated into the first revision of the online ANNiKEY Interactive.

## ﻿﻿Additional information

### Author ORCIDs

Christopher J. Glasby https://orcid.org/0000-0002-9464-1938

Olga Biriukova https://orcid.org/0009-0002-6760-2295

Patrick Martin https://orcid.org/0000-0002-6033-8412

Geoffrey R. Dyne https://orcid.org/0000-0002-5475-2385

Serge Utevsky https://orcid.org/0000-0003-1290-6742

Robin S. Wilson https://orcid.org/0000-0002-9441-2131

### Data availability

All of the data that support the findings of this study are available in the main text, the Supplementary Information and on the Zenodo ANNiKEY Interactive page (https://zenodo.org/records/13738486).

## Supplementary materials

﻿Supplementary material 1 Morphological descriptions of annelid family-level taxa Data type docx https://binary.pensoft.net/file/1385541This dataset is made available under the Open Database License (http://opendatacommons.org/licenses/odbl/1.0/). The Open Database License (ODbL) is a license agreement intended to allow users to freely share, modify, and use this Dataset while maintaining this same freedom for others, provided that the original source and author(s) are credited.Christopher J. Glasby, Olga Biriukova, Patrick Martin, Geoffrey R. Dyne, Serge Utevsky, Robin S. Wilson

﻿Supplementary material 2 Annotated morphological character list for Annelida Data type docx https://binary.pensoft.net/file/1385542This dataset is made available under the Open Database License (http://opendatacommons.org/licenses/odbl/1.0/). The Open Database License (ODbL) is a license agreement intended to allow users to freely share, modify, and use this Dataset while maintaining this same freedom for others, provided that the original source and author(s) are credited.Christopher J. Glasby, Olga Biriukova, Patrick Martin, Geoffrey R. Dyne, Serge Utevsky, Robin S. Wilson

﻿Supplementary material 3 NEXUS file of morphological characters for Annelida Data type txt https://binary.pensoft.net/file/1385543This dataset is made available under the Open Database License (http://opendatacommons.org/licenses/odbl/1.0/). The Open Database License (ODbL) is a license agreement intended to allow users to freely share, modify, and use this Dataset while maintaining this same freedom for others, provided that the original source and author(s) are credited.Christopher J. Glasby, Olga Biriukova, Patrick Martin, Geoffrey R. Dyne, Serge Utevsky, Robin S. Wilson

## References

[B1] Aguado MT Nygren A Rouse GW (2013) Two apparently unrelated groups of symbiotic annelids, Nautiliniellidae and Calamyzidae (Phyllodocida, Annelida), are a clade of derived chrysopetalid polychaetes. Cladistics 29: 610–628. 10.1111/cla.12011 34798772

[B2] Alvestad T Budaeva N (2020) Ampharetidae and Melinnidae. Universitetsmuseet i Bergen. https://www.artsdatabanken.no/Pages/299547

[B3] Anderson FE Williams BW Horn KM Erséus C Halanych KM Santos SR James SW (2017) Phylogenomic analyses of Crassiclitellata support majorNorthern and Southern Hemisphere clades and a Pangaean origin for earthworms. BMC Evolutionary Biology 17: 123. 10.1186/s12862-017-0973-4 PMC5450073 28558722

[B4] Andrade SCS Novo M Kawauchi GY Worsaae K Pleijel F Giribet G Rouse GW (2015) Articulating “Archiannelids”: Phylogenomics and Annelid Relationships, with Emphasis on Meiofaunal Taxa. Molecular Biology and Evolution 32: 2860–2875. 10.1093/molbev/msv157 26205969

[B5] Arias A Barroso R Anadón N Paiva PC (2013) On the occurrence of the fireworm *Eurythoecomplanata* complex (Annelida, Amphinomidae) in the Mediterranean Sea with an updated revision of the alien Mediterranean amphinomids. ZooKeys 33: 19–33. 10.3897/zookeys.337.5811 PMC3800783 24146576

[B6] Assis JE de Bleidorn C Christoffersen ML (2021) 7.7.6 Maldanidae Malmgren, 1867. In: Purschke G Böggemann M Westheide W (Eds) Handbook of Zoology, Annelida, Volume 3: Pleistoannelida, Sedentaria III and Errantia I.De Gruyter, Berlin, 186–201. 10.1515/9783110291704-009

[B7] Assis JE de Souza JRBD Fitzhugh K Christoffersen ML (2022) A new species of *Euclymene* (Maldanidae, Annelida) from Brazil, with new combinations, and phylogenetic implications for Euclymeninae. Anais da Academia Brasileira de Ciências 94: e20210283. 10.1590/0001-3765202220210283 36541974

[B8] Banse K (1969) Acrocirridae n.fam. (PolychaetaSedentaria). Journal of the Fisheries Research Board of Canada 26(10): 2595–2620. 10.1139/f69-253

[B9] Barnich R (2011) Identification of scale worms in British and Irish waters. NMBAQC 2010 taxonomic workshop, Dove Marine Laboratory, 52 pp. https://www.nmbaqcs.org/scheme-components/invertebrates/literature-and-taxonomic-keys/

[B10] Barnich R Fiege D (2000) Review of the North East Atlantic and Mediterranean species of *Aphrodita* Linnaeus, 1758 and *Aphroditella* Roule, 1898 (Polychaeta: Aphroditidae). Ophelia 53(2): 131–140. 10.1080/00785236.2000.10409443

[B11] Barnich R Fiege D (2003) The Aphroditoidea (Annelida: Polychaeta) of the Mediterranean Sea. Abhandlungen der Senckenbergischen Naturforschenden Gesellschaft 559: 1–167.

[B12] Barnich R Van Haaren T (2021) Revision of *Sthenelais* Kinberg, 1856, *Fimbriosthenelais* Pettibone, 1971 and *Eusthenelais* McIntosh, 1876 (Polychaeta, Sigalionidae) in the Northeast Atlantic. European Journal of Taxonomy 740: 138–171. 10.5852/ejt.2021.740.1287

[B13] Barnich R Fiege D Ruiping S (2004) Polychaeta (Annelida) of Hainan Island, South China Sea Part III. Aphroditoidea. Species Diversity 9: 285–329. 10.12782/specdiv.9.285

[B14] Barnich R Dietrich A Hager T Fiege D (2019) On the genera *Malmgrenia* McIntosh, 1874 and *Pettibonesia* Nemésio, 2006 in the Northeast Atlantic and Mediterranean Sea, with descriptions of two new species (Polychaeta: Polynoidae). Marine Biodiversity 49: 315–324. 10.1007/s12526-017-0802-4

[B15] Barroso M Moreira J Capa M Nygren A Parapar J (2022) A further step towards the characterisation of *Terebellides* (Annelida, Trichobranchidae) diversity in the Northeast Atlantic, with the description of a new species. Zookeys 1132: 85–126. 10.3897/zookeys.1132.91244 36760494 PMC9836732

[B16] Bartolomaeus T (1999) Structure, function and development of segmental organs in Annelida. Hydrobiologia 402: 21–37. 10.1023/A:1003780223216

[B17] Bartz MLC James SW Pasini A Brown GG (2012) New earthworm species of *Glossoscolex* Leuckart, 1835 and *Fimoscolex* Michaelsen, 1900 (Clitellata: Glossoscolecidae) from Northern Paraná, Brazil. Zootaxa 3458: 59–85. 10.11646/zootaxa.3458.1.3 40173772

[B18] Beesley PL Ross GJB Glasby CJ (2000) Fauna of Australia. Volume 4A, Polychaetes & Allies: The Southern Synthesis. CSIRO Publishing Australia, 465 pp. http://www.environment.gov.au/science/abrs/publications/fauna-of-australia/fauna-4a

[B19] Bhaud MR Cazaux CP (1987) Description and identification of polychaete larvae; their implications in current biological problems. Oceanis 13(6): 595–753.

[B20] Bick A (2005) A new Spionidae (Polychaeta) from North Carolina, and a redescription of *Marenzelleriawireni* Augener, 1913, from Spitsbergen, with a key for all species of *Marenzelleria*. Helgoland Marine Research 59: 265–272. 10.1007/s10152-005-0002-7

[B21] Biseswar R (1984) A Key to the species of *Anelassorhynchus* (Echiura) with a description of a new species from the east coast of southern Africa. South African Journal of Zoology 19: 16–21. 10.1080/02541858.1984.11447851

[B22] Biseswar R (2009) The geographic distribution of echiurans in the Atlantic Ocean (Phylum Echiura). Zootaxa 2222: 17–30. 10.11646/zootaxa.2222.1.2

[B23] Biseswar R (2010) Zoogeography of the echiuran fauna of the Indo-West Pacific Ocean (Phylum: Echiura). Zootaxa 2727: 21. 10.11646/zootaxa.2727.1.2

[B24] Biseswar R (2019) The echiuran fauna of southern Africa (Class: Echiura, Phylum: Annelida). African Zoology 54: 73–90. 10.1080/15627020.2019.1600429

[B25] Blake JA (1996a) Family Apistobranchidae Mesnil & Caullery. In: Blake JA Hilbig B Scott PH (Eds) Taxonomic Atlas of the Benthic Fauna of the Santa Maria Basin and Western Santa Barbara Channel.Vol. 6. The Annelida. Part 3: Polychaeta: Orbiniidae to Cossuridae. Santa Barbara Museum of Natural History, Santa Barbara, 71–79.

[B26] Blake JA (1996b) Family Chaetopteridae Malmgren, 1867. In: Blake JA Hilbig B Scott PH (Eds) Taxonomic Atlas of the Benthic Fauna of the Santa Maria Basin and Western Santa Barbara Channel.6 – The Annelida Part 3. Polychaeta: Orbiniidae to Cossuridae. Santa Barbara Museum of Natural History. Santa Barbara, 223–251.

[B27] Blake JA (2000) Family Opheliidae Malmgren, 1867. In: Blake JA Hilbig B Scott PV (Eds) Taxonomic atlas of the benthic fauna of the Santa Maria Basin and western Santa Barbara Channel, vol.7 – The Annelida, Part 4. Polychaeta: Flabelligeridae to Sternaspidae. Santa Barbara Museum of Natural History, Santa Barbara, 145–168.

[B28] Blake JA (2018) Bitentaculate Cirratulidae (Annelida, Polychaeta) collected chiefly during cruises of the R/V Anton Bruun, USNS Eltanin, USCG Glacier, R/V Hero, RVIB Nathaniel B. Palmer, and R/V Polarstern from the Southern Ocean, Antarctica, and off Western South America. Zootaxa 4537: 1. 10.11646/zootaxa.4537.1.1 30647335

[B29] Blake JA (2019) 7.6.3 Scalibregmatidae Malgren, 1867. In: Purschke G Böggemann M Westheide W (Eds) Handbook of Zoology, Annelida, Volume 2: Pleistoannelida, Sedentaria II.De Gruyter, Berlin, 312–349. 10.1515/9783110291681-010

[B30] Blake JA Arnofsky PL (1999) Reproduction and larval development of the spioniform Polychaeta with application to systematics and phylogeny. Hydrobiologia 402: 27–106. 10.1023/A:1003784324125

[B31] Blake JA Maciolek NJ (2018) New species and records of Uncispionidae and *Pygospiopsis* (Polychaeta, Spionida) from deep water off the east and west coasts of North America, the Gulf of Mexico, the Antarctic Peninsula, and Southeast Asia. Zootaxa 4450: 151–195. 10.11646/zootaxa.4450.2.1 30313844

[B32] Blake JA Maciolek NJ (2019a) 7.3.1.9 Longosomatidae Hartman, 1944. In: Purschke G Böggemann M Westheide W (Eds) Handbook of Zoology, Annelida, Volume 1: Annelida Basal Groups and Pleistoannelida, Sedentaria I.De Gruyter, Berlin, 457–466.

[B33] Blake JA Maciolek NJ (2019b) 7.4.2 Poecilochaetidae Hennerz, 1956. In: Purschke G Böggemann M Westheide W (Eds) Handbook of Zoology, Annelida, Volume 2: Pleistoannelida, Sedentaria II.De Gruyter, Berlin, 103–119. 10.1515/9783110291681-003

[B34] Blake JA Maciolek NJ (2019c) 7.4.3 Trochochaetidae Pettibone, 1963. In: Purschke G Böggemann M Westheide W (Eds) Handbook of Zoology, Annelida, Volume 2: Pleistoannelida, Sedentaria II.De Gruyter, Berlin, 120–135.

[B35] Blake JA Maciolek NJ (2019d) 7.4.4 Uncispionidae Green, 1982. In: Purschke G Böggemann M Westheide W (Eds) Handbook of Zoology, Annelida, Volume 2: Pleistoannelida, Sedentaria II.De Gruyter, Berlin, 136–144. 10.1515/9783110291681-004

[B36] Blake JA Maciolek NJ (2019e) 7.6.2 Travisiidae Hartmann-Schröder, 1971, new family status. In: Purschke G Böggemann M Westheide W (Eds) Handbook of Zoology, Annelida, Volume 2: Pleistoannelida, Sedentaria II.De Gruyter, Berlin, 302–311. 10.1515/9783110291681-009

[B37] Blake JA Maciolek NJ (2019f) 7.6.1 Opheliidae Malmgren, 1867. In: Purschke G Böggemann M Westheide W (Eds) Handbook of Zoology, Annelida, Volume 2: Pleistoannelida, Sedentaria II.De Gruyter, Berlin, 285–301. 10.1515/9783110291681-008

[B38] Blake JA Maciolek NJ (2023) New species and records of Heterospio (Annelida, Longosomatidae) from continental shelf, slope and abyssal depths of the Atlantic Ocean, Pacific Ocean, Indian Ocean and adjacent seas. Zootaxa 5260: 1–74. 10.11646/zootaxa.5260.1.1 37044570

[B39] Blake JA Magalhães W (2019) 7.3.1.5 Cirratulidae Ryckholt, 1851. In: Purschke G Böggemann M Westheide W (Eds) Handbook of Zoology, Annelida, Volume 1: Annelida Basal Groups and Pleistoannelida, Sedentaria I.De Gruyter, Berlin, 339–397.

[B40] Blake JA Petti MAV (2019) 5.1 Apistobranchidae Mesnil & Caullery, 1898. In: Purschke G Böggemann M Westheide W (Eds) Handbook of Zoology, Annelida, Volume 1: Annelida Basal Groups and Pleistoannelida, Sedentaria I.De Gruyter, Berlin, 133–142. 10.1515/9783110291582-005

[B41] Blakemore R (1999) Diversity of exotic earthworms in Australia – a status report. In: Ponder W Lunney D (Eds) The Other 99%.The conservation and biodiversity of Invertebrates. Transactions of the Royal Zoological Society of New South Wales, Mosman, NSW, 182–187. 10.7882/RZSNSW.1999.030

[B42] Blakemore RJ (2006) Introductory key to the Revised Families of Earthworms of the world. In: Kaneko N, Ito MT (Eds) A Series of Searchable Texts on Earthworm Biodiversity, Ecology and Systematics from Various Regions of the World. CD-ROM publication by Soil Ecology Research Group, Graduate School of Environment & Information Sciences, Yokohama National University, Yokohama, Japan, 14 pp. [accessed via ResearchGate, Dec. 2024]

[B43] Blakemore RJ (2008) Review of Criodrilidae (Annelida: Oligochaeta) including *Biwadrilus* from Japan. Opuscula Zoologica Budapest 37: 11–22.

[B44] Blakemore RJ (2009) Cosmopolitan earthworms – a global and historical perspective. In: Shain DH (Ed.) Annelids as Model Systems in the Biological Sciences.John Wiley & Sons, New York, 257–283. 10.1002/9780470455203.ch14

[B45] Bochert R Zettler ML (2013) A record of the genus *Trochochaeta* (Polychaeta) in the southern hemisphere with description of a new species. Journal of the Marine Biological Association of the UK 93: 967–972. 10.1017/S0025315412001142

[B46] Böggemann M (2002) Revision of the Glyceridae Grube 1850 (Annelida: Polychaeta). Abhandlungen Der Senckenbergischen Naturforschenden Gesellschaft 555: 1–249.

[B47] Böggemann M (2005) Revision of the Goniadidae (Annelida, Polychaeta). Abhandlungen des Naturwissenschaftlichen Vereins Hamburg (NF) 39: 1–354.

[B48] Böggemann M (2015) Glyceriformia Fauchald, 1977 (Annelida: “Polychaeta”) from Lizard Island, Great Barrier Reef, Australia. Zootaxa 4019: 70–97. 10.11646/zootaxa.4019.1.7 26624067

[B49] Böggemann M (2022) 7.13.4.1 Glyceridae Grube, 1850. In: Purschke G Böggemann M Westheide W (Eds) Handbook of Zoology, Annelida, Volume 3: Pleistoannelida, Errantia II.De Gruyter, Berlin, 323–344. 10.1515/9783110647167-012

[B50] Böggemann M (2023) GLYCEREA – Identification guide to the species of Glyceridae and Goniadidae. https://www.uni-vechta.de/biologie/das-team/boeggemann-markus/online-publication [Accessed Jan. 2024]

[B51] Böggemann M Fiege D (2001) Description of seven new species of the genus *Glycera* Savigny, 1818 (Annelida: Polychaeta: Glyceridae). Ophelia 54: 29–49. 10.1080/00785326.2001.10409454

[B52] Bonifácio P Menot L (2019) New genera and species from the Equatorial Pacific provide phylogenetic insights into deep-sea Polynoidae (Annelida). Zoological Journal of the Linnean Society 185: 555–635. 10.1093/zoolinnean/zly063

[B53] Borda E Siddall ME (2004) Arhynchobdellida (Annelida: Oligochaeta: Hirudinida): Phylogenetic relationships and evolution. Molecular Phylogenetics and Evolution 30: 213–225. 10.1016/j.ympev.2003.09.002 15022771

[B54] Borda E Siddall ME (2010) Insights into the evolutionary history of Indo-Pacific bloodfeeding terrestrial leeches (Hirudinida: Arhynchobdellida: Haemadipsidae). Invertebrate Systematics 24: 456–472. 10.1071/IS10013

[B55] Borda E Oceguera-Figueroa A Siddall ME (2008) On the classification, evolution and biogeography of terrestrial haemadipsoid leeches (Hirudinida: Arhynchobdellida: Hirudiniformes). Molecular Phylogenetics and Evolution 46: 142–154. 10.1016/j.ympev.2007.09.006 17977750

[B56] Brinkhurst RO (1982) British and other marine and estuarine oligochaetes. Keys and notes for the identification of the species. Synopses of the British Fauna (New Series). 21. Cambridge: Cambridge University Press, 127 pp.

[B57] Brinkhurst RO Gelder SR (2001) 12. Annelida: Oligochaeta, including Branchiobellida. In: Thorp JH Covich AP (Eds) Ecology and Classification of North American Freshwater Invertebrates.Academic Press, 431–463. 10.1016/B978-012690647-9/50013-2

[B58] Brinkhurst RO Jamieson BGM (1971) Aquatic Oligochaeta of the World with contributions by D.G. Cook, D.V. Anderson, J. van der Land. University of Toronto Press, Toronto, Canada, 860 pp.

[B59] Budaeva N (2021) 7.12.3 Onuphidae Kinberg, 1865. In: Purschke G Böggemann M Westheide W (Eds) Handbook of Zoology, Annelida, Volume 3: Pleistoannelida, Sedentaria III and Errantia I.De Gruyter, Berlin, 383–413. 10.1515/9783110291704-019

[B60] Budaeva N Fauchald K (2008) *Diopatratuberculantennata*, a new species of Onuphidae (Polychaeta) from Belize with a key to onuphids from the Caribbean Sea. Zootaxa 1795: 29–45. 10.11646/zootaxa.1795.1.2

[B61] Budaeva N Schepetov D Zanol J Neretina T Willassen E (2016) When molecules support morphology: Phylogenetic reconstruction of the family Onuphidae (Eunicida, Annelida) based on 16S rDNA and 18S rDNA. Molecular Phylogenetics and Evolution 94 (Part B): 791–801. 10.1016/j.ympev.2015.10.011 26497420

[B62] Bunke D (1967) Zur Morphologie und Systematik der Aeolosomatidae Beddard 1895 und Potamodrilidae nov. fam. (Oligochaeta). Zoologische Jahrbücher, Abteilung für Systematik, Ökologie und Geographie der Tiere 94(2/3): 187–368.

[B63] Burreson EM (2020) Marine and estuarine leeches (Hirudinida : Ozobranchidae and Piscicolidae) of Australia and New Zealand with a key to the species. Invertebrate Systematics 34: 235–259. 10.1071/IS19048

[B64] Campoy A San Martín G (1980) *Pettiboneiaurciensis* sp.n.: un nouveau Dorvilleidae (Polychètes: Errantes) de la Méditerranée. Cahiers de Biologie Marine 21: 201–207.

[B65] Cantone G Di Pietro N (1998) A new species of *Myriochele* (Polychaeta, Oweniidae) from Antarctica, with considerations on the Antarctic oweniids. Polar Biology 19: 421–423. 10.1007/s003000050268

[B66] Capa M Bakken T (2015) Revision of the Australian Sphaerodoridae (Annelida) including the description of four new species. Zootaxa 4000: 227–267. 10.11646/zootaxa.4000.2.3 26623612

[B67] Capa M Kupriyanova E Nogueira JMM Bick A Tovar-Hernández MA (2021) Fanworms: Yesterday, Today and Tomorrow. Diversity 13: 130. 10.3390/d13030130

[B68] Capa M Murray A (2009) Review of the genus *Megalomma* (Sabellidae: Polychaeta) in Australia with description of three new species, new records and notes on certain features with phylogenetic implications. Records of the Australian Museum 61(2): 201–224. 10.3853/j.0067-1975.61.2009.1529

[B69] Capa M Murray A (2015) Integrative taxonomy of *Parasabella* and *Sabellomma* (Sabellidae: Annelida) from Australia: description of new species, indication of cryptic diversity, and translocation of some species out of their natural distribution range. Zoological Journal of the Linnean Society 175: 764–811. 10.1111/zoj.12308

[B70] Capa M Hutchings P Aguado T Bott NJ (2010) Phylogeny of Sabellidae (Annelida) and relationships with other taxa inferred from morphology and multiple genes. Cladistics 27: 449–469. 10.1111/j.1096-0031.2010.00341.x 34875800

[B71] Capa M Hutchings PA Peart R (2012a) Systematic revision of Sabellariidae (Polychaeta) and their relationships with other polychaetes using morphological and DNA sequence data. Zoological Journal of the Linnean Society 164: 245–284. 10.1111/j.1096-3642.2011.00767.x

[B72] Capa M Parapar J Hutchings P (2012b) Phylogeny of Oweniidae (Polychaeta) based on morphological data and taxonomic revision of Australian fauna. Zoological Journal of the Linnean Society 166: 236–278. 10.1111/j.1096-3642.2012.00850.x

[B73] Capa M Osborn KJ Bakken T (2016) Sphaerodoridae (Annelida) of the deep Northwestern Atlantic, including remarkable new species of *Euritmia* and *Sphaerephesia*. ZooKeys 615: 1–32. 10.3897/zookeys.615.9530 PMC5027775 27667938

[B74] Capa M Bakken T Meißner K Nygren A (2018) Three, two, one! Revision of the long-bodied sphaerodorids (Sphaerodoridae, Annelida) and synonymization of *Ephesiella*, *Ephesiopsis* and *Sphaerodorum*. PeerJ 6(1): e5783. 10.7717/peerj.5783 PMC6204827 30386698

[B75] Capa M Giangrande A Nogueira JMM Tover-Hernandez MA (2019a) 7.4.6 Sabellidae Latreille, 1825. In: Purschke G Böggemann M Westheide W (Eds) Handbook of Zoology, Annelida, Volume 2: Pleistoannelida, Sedentaria II.De Gruyter, Berlin, 164–212.

[B76] Capa M Nygren A Parapar J Bakken T Meißner K Moreira J (2019b) Systematic re-structure and new species of Sphaerodoridae (Annelida) after morphological revision and molecular phylogenetic analyses of the North East Atlantic fauna. ZooKeys 845: 1–97. 10.3897/zookeys.845.32428 31148919 PMC6531415

[B77] Capa M Parapar J Hutchings P (2019c) 4.1 Oweniidae Rioja, 1917. In: Purschke G Böggemann M Westheide W (Eds) Handbook of Zoology, Annelida, Volume 1: Annelida Basal Groups and Pleistoannelida, Sedentaria I.De Gruyter, Berlin, 91–112. 10.1515/9783110291582-004

[B78] Capa M Bakken T Purschke G (2022) 7.13.14 Sphaerodoridae Malmgren, 1867. In: Purschke G Böggemann M Westheide W (Eds) Handbook of Zoology.Annelida. Vol. 4: Pleistoannelida, Errantia II. De Gruyter, Berlin, 413–438. 10.1515/9783110647167-020

[B79] Carrera-Parra LF (2003) Redescription of *Hartmaniellatulearensis* n. comb. (Amoureux, 1978) with comments on *Hartmaniella* sp. and affinities of the family (Polychaeta: Hartmaniellidae). Journal of Natural History 37: 49–55. 10.1080/713834394

[B80] Carrera-Parra LF (2006) Phylogenetic analysis of Lumbrineridae Schmarda, 1861 (Annelida: Polychaeta). Zootaxa 1332: 1–36. 10.11646/zootaxa.1332.1.1

[B81] Carrera-Parra LF Salazar-Vallejo SI (1998) A new genus and 12 new species of EunicidaePolychaeta) from the Caribbean Sea. Marine Biological Association of the UK 78: 145–182. 10.1017/S0025315400040005

[B82] Caullery M (1914) Sur les Siboglinidae, type nouveau d’invertébrés receuillis par l’expédition du Siboga. Comptes rendus hebdomadaires des séances de l’Académie des sciences 158: 2014–2017. http://biodiversitylibrary.org/page/7159983

[B83] Celis LV Rangel-Ch O (2015) Dos especies nuevas (Oligochaeta: Tumakidae) de la región Caribe de Colombia. Papeis Avulso de Zoologica 55: 405–414. 10.1590/0031-1049.2015.55.30

[B84] Cepeda D López E San Martín G Parapar J (2022) AnnelidaPolychaeta VI. In: Fauna Ibérica vol. 47. MA Ramos et al. (Eds). Museo Nacional de Ciencias Naturales, CSIC, Madrid, 452 pp.

[B85] Cerca J Meyer C Purschke G Struck TH (2020) Delimitation of cryptic species drastically reduces the geographical ranges of marine interstitial ghost-worms (*Stygocapitella*; Annelida, Sedentaria). Molecular Phylogenetics and Evolution 143: 106663. [1–16] https://www.sciencedirect.com/science/article/pii/S1055790319303975 10.1016/j.ympev.2019.106663 31669400

[B86] Chanabun R Sutcharit C Tongkerd P Panha S (2013) The semi-aquatic freshwater earthworms of the genus *Glyphidrilus* Horst, 1889 from Thailand (Oligochaeta, Almidae) with re-descriptions of several species. Zookeys 265: 1–76. 10.3897/zookeys.265.3911 PMC3591759 23653518

[B87] Chanabun R Jirapatrasilp P Seesamut T Bantaowong U Aoonkum A Inkhavilay K (2023) Two New Species of Semi-Aquatic Freshwater Earthworm Genus *Glyphidrilus* Horst, 1889 (Oligochaeta: Almidae) from Thailand and Laos. Tropical Natural History 7: 31–40.

[B88] Chang CH Snyder BA Szlavecz K (2016) Asian pheretimoid earthworms in North America north of Mexico: An illustrated key to the genera *Amynthas*, *Metaphire*, *Pithemera*, and *Polypheretima* (Clitellata: Megascolecidae). Zootaxa 4179(3): 495–529. [PMID: 27811684] 10.11646/zootaxa.4179.3.7 27811684

[B89] Chávez-López Y (2022) New species of sabellariids (Annelida: Sabellariidae) from the Caribbean Sea and the Gulf of Mexico. European Journal of Taxonomy 831: 109–148. 10.5852/ejt.2022.831.1873

[B90] Christoffersen ML (2008) A Catalogue of the Piscicolidae, Ozobranchidae, and Arhynchobdellida (Annelida, Clitellata, Hirudinea) from South America. Neotropical Biology and Conservation 3(1): 39–48.

[B91] Çinar ME Petersen ME (2011) Re-description of *Cirratulusdollfusi* (Polychaeta: Cirratulidae), and *Fauvelicirratulus* as a new genus. Journal of the Marine Biological Association of the UK 91: 415–418. 10.1017/S0025315410000895

[B92] Çinar ME Daglia E Açik S (2011) Annelids (Polychaeta and Oligochaeta) from the Sea of Marmara, with descriptions of five new species. Journal of Natural History 45: 2105–2143. 10.1080/00222933.2011.582966

[B93] Clausen MW (2003) Description of seven new species of *Libyodrilus* Beddard from Cameroon, with a key to the species of the genus (Oligochaeta: Eudrilidae). Journal of Natural History 38: 1851–1880. 10.1080/00222930310001613575

[B94] Cruz-Gómez C (2021) A new genus and seven new species of chrysopetalids (Annelida, Chrysopetalidae) from the Tropical Eastern Pacific. Zootaxa 5068(1): 1–59. 10.11646/zootaxa.5068.1.1 34810723

[B95] Cutler E (1994) The Sipuncula. Their systematics, biology, and evolution. Cornell University Press, Ithaca, New York, 453 pp. 10.7591/9781501723643

[B96] D’Alessandro M Romeo T Castriota L Cosentino A Perzia P Martins R (2016) New records of Lumbrineridae (Annelida: Polychaeta) in the Mediterranean biogeographic province, with an updated taxonomic key. Italian Journal of Zoology 83: 233–243. 10.1080/11250003.2016.1154615

[B97] Dales KP (1957) Pelagic Polychaetes of the Pacific Ocean. UC San Diego: Scripps Institution of Oceanography. https://escholarship.org/uc/item/51c8j5nj

[B98] Dallwitz MJ (1974) A flexible computer program for generating identification keys. Systematic Zoology 23(1): 1–50. 10.2307/2412239

[B99] Dallwitz MJ (1980) A general system for coding taxonomic descriptions. Taxon 29(1): 41–46. 10.2307/1219595

[B100] Dallwitz MJ (2020) Installing and running the programs of the DELTA System. Reports, Division of Entomology CSIRO Australia. https://www.delta-intkey.com/www/programs.htm [Accessed 31 January 2023]

[B101] Dallwitz MJ Paine TA (2015) Definition of the DELTA format. Reports, Division of Entomology CSIRO Australia. https://www.delta-intkey.com/www/standard.pdf [Accessed 31 January 2023]

[B102] Dallwitz MJ Paine TA Zurcher EJ (1993) User’s guide to the DELTA System: a general system for processing taxonomic descriptions. Reports, Division of Entomology CSIRO Australia. https://www.delta-intkey.com/www/uguide.htm [Accessed 1 October 2023]

[B103] Darbyshire T (2020) 7.7.5 Arenicolidae Johnston, 1835. In: Purschke G Böggemann M Westheide W (Eds) Handbook of Zoology, Annelida, Volume 3: Pleistoannelida, Sedentaria III and Errantia I.De Gruyter, Berlin, 163–185. 10.1515/9783110291704-008

[B104] Darbyshire T Brewin PE (2015) Three new species of *Dysponetus* Levinsen, 1879 (Polychaeta: Chrysopetalidae) from the South Atlantic and Southern Ocean, with a re-description of *Dysponetusbulbosus* Hartmann-Schröder, 1982. Zootaxa 4040: 359. 10.11646/zootaxa.4040.3.7 26624671

[B105] Davies RW Govedich FR (2001) 13. Annelida: Euhirudinea and Acanthobdellidae. In: Thorp JH Covich AP (Eds) Ecology and classification of North American freshwater Invertebrates.Academic Press, 465–504. 10.1016/B978-012690647-9/50014-4

[B106] De Carle DB Gajda L Bielecki A Cios S Cichocka JM Golden HE Gryska AD Sokolov S Shedko MB Knudsen R Utevsky S Śwaitek P Tessler M (2023) Recent evolution of ancient Arctic leech relatives: systematics of Acanthobdellida. Zoological Journal of the Linnean Society 196: 149–168. 10.1093/zoolinnean/zlac006

[B107] Dean HK Blake JA (2019) 7.3.1.4 Ctenodrilidae Kennel, 1882. In: Purschke G Böggemann M Westheide W (Eds) Handbook of Zoology, Annelida, Volume 1: Annelida Basal Groups and Pleistoannelida, Sedentaria I.De Gruyter, Berlin, 328–338.

[B108] Di Domenico M Worsaae K Purschke G (2021) 7.11.2 Saccocirridae Czerniavsky, 1881. In: Purschke G Böggemann M Westheide W (Eds) Handbook of Zoology, Annelida, Volume 3: Pleistoannelida, Sedentaria III and Errantia I.De Gruyter, Berlin, 280–298. 10.1515/9783110291704-014

[B109] Dnestrovskaya NY (2020) Identification key to Nephtyidae (Annelida) of the Black Sea. ZooKeys 908: 1–17. 10.3897/zookeys.908.38203 32123496 PMC7024970

[B110] Dohren J von (2019) 7.6.5 Echiura Stephen, 1965 (= Thalassematidae, Forbes & Goodsir, 1841). In: Purschke G Böggemann M Westheide W (Eds) Handbook of Zoology, Annelida, Volume 2: Pleistoannelida, Sedentaria II.De Gruyter, Berlin, 404–450. 10.1515/9783110291681-012

[B111] Dos Santos BTS Bartz MLC Hernández-García LM Rousseau GX Martins MB James SW (2017) New earthworm species of *Righiodrilus* (Clitellata, Glossoscolecidae) from eastern Amazonia. Zootaxa 4242: 392–400. 10.11646/zootaxa.4242.2.11 28610191

[B112] Dyne GR Jamieson BGM (2004) Native Earthworms of Australia II (Megascolecidae, Acanthodrilinae). Department of the Environment and Heritage, Australian Government, 200 pp.

[B113] Easton EG (1984) Earthworms (Oligochaeta) from islands of the south-western Pacific, and a note on two species from Papua New Guinea. New Zealand Journal of Zoology 11: 111–128. 10.1080/03014223.1984.10423750

[B114] Ebbe B Purschke G (2021) 7.7.2 Ampharetidae Malmgren, 1866. In: Purschke G Böggemann M Westheide W (Eds) Handbook of Zoology, Annelida, Volume 3: Pleistoannelida, Sedentaria III and Errantia I.De Gruyter, Berlin, 50–67. 10.1515/9783110291704-005

[B115] Edmonds SJ (2000) Phylum Echiura. In: Beesley PL Ross GJB Glasby CJ (Eds) Polychaetes & Allies: The Southern Synthesis.Fauna of Australia Vol. 4A Polychaeta, Myzostomida, Pogonophora, Echiura, Sipuncula. CSIRO Publishing, Melbourne, 353–374.

[B116] Eibye-Jacobsen D Kristensen RM (1994) A new genus and species of Dorvilleidae (Annelida, Polychaeta) from Bermuda, with a phylogenetic analysis of Dorvilleidae, Iphitimidae and Dinophilidae. Zoologica Scripta 23(3): 107–131. 10.1111/j.1463-6409.1994.tb00379.x

[B117] Eibye-Jacobsen D Vinther J (2012) Reconstructing the ancestral annelid. Journal of Zoological Systematics and Evolution Research 50: 85–87. 10.1111/j.1439-0469.2011.00651.x

[B118] Eisig H (1906) *Ichthyotomussanguinarius*, eine auf Aalen schmarotzende Annelide. 266 Fauna und Flora des Golfes von Neapel und der angrenzenden Meeres-Abschnitte 28: 1–300.

[B119] Erséus C (1997) A record of *Randiella* from New Caledonia, the first known occurrence of the marine interstitial family Randiellidae (AnnelidaOligochaeta) in the South Pacific Ocean. Journal of Natural History 31: 1745–1750. 10.1080/00222939700770931

[B120] Erséus C (2005) Phylogeny of oligochaetous Clitellata. Hydrobiologia 535/536: 357–372. 10.1007/s10750-004-4426-x

[B121] Erséus C Healy BM (2001) Oligochaeta. In: Costello MJ Emblow C White RJ (Eds) European register of marine species: a check-list of the marine species in Europe and a bibliography of guides to their identification.Collection Patrimoines Naturels 50. Muséum national d’Histoire naturelle, Paris, 231–234.

[B122] Erséus C Strehlow DR (1986) Four new interstitial species of marine Oligochaeta representing a new family. Zoologica Scripta 15: 53–60. 10.1111/j.1463-6409.1986.tb00208.x

[B123] Erséus C Prestegaard T Källersjö M (2000) Phylogenetic analysis of Tubificidae (Annelida, Clitellata) Based on 18S rDNA Sequences. Molecular Phylogenetics and Evolution 15: 381–389. 10.1006/mpev.1999.0761 10860647

[B124] Erséus C Envall I Marchese M Gustavsson L (2010a) The systematic position of Opistocystidae (Annelida, Clitellata) revealed by DNA data. Molecular Phylogenetics and Evolution 54: 309–313. 10.1016/j.ympev.2009.09.037 19825421

[B125] Erséus C Rota E Matamoros L De Wit P (2010b) Molecular phylogeny of Enchytraeidae (Annelida, Clitellata). Molecular Phylogenetics and Evolution 57: 849–858. 10.1016/j.ympev.2010.07.005 20637291

[B126] Erséus C Envall I De Wit P Gustavsson LM (2017) Molecular data reveal a tropical freshwater origin of Naidinae (Annelida, Clitellata, Naididae). Molecular Phylogenetics and Evolution 115: 115–127. 10.1016/j.ympev.2017.07.016 28743643

[B127] Erséus C Williams BW Horn KM Halanych KM Santos SR James SW Creuzé des Châtelliers M Anderson FE (2020) Phylogenomic analyses reveal a Palaeozoic radiation and support a freshwater origin for clitellate annelids. Zoologica Scripta 49: 614–640. 10.1111/zsc.12426

[B128] Fauchald K (1974) Sphaerodoridae (Polychaeta: Errantia) from world-wide areas. Journal of Natural History 8: 257–289. 10.1080/00222937400770241

[B129] Fauchald K (1977) The polychaete worms, definitions and keys to the orders, families and genera. Natural History Museum of Los Angeles County: Los Angeles, CA (USA), Science Series 28: 1–188. http://hdl.handle.net/10088/3435

[B130] Fauchald K Jumars PA (1979) The diet of worms: a study of polychaete feeding guilds. Annual Review of Oceanography and Marine Biology 17: 193–284.

[B131] Feijoo MA Celis LV (2012) New species of earthworms (Oligochaeta: Glossoscolecidae) in the Amazon region of Colombia. Zootaxa 3458: 104–119. 10.11646/zootaxa.3458.1.5

[B132] Fend SV Ohtaka A Torii T (2020) New species and new records of Japanese Lumbriculidae (Annelida, Clitellata). Zoosymposia 17: 14–33. 10.11646/zoosymposia.17.1.5

[B133] Fitzhugh K (2006) The philosophical basis of character coding for the influence of phylogenetic hypotheses. Zoological Scripta 35: 261–286. 10.1111/j.1463-6409.2006.00229.x

[B134] Fitzhugh K Mortimer K dos Santos Brasil AC (2024) The monophyly of *Magelona* F. Müller, 1858 (Polychaeta, Magelonidae): Comments on Meißner et al.’s (2023) reinstatement of *Octomagelona* Aguirrezabalaga, Ceberio & Fiege, 2001. Zootaxa 5497: 496–504. 10.11646/zootaxa.5497.4.2 39647137

[B135] Fragoso C Rojas P (2009) A new ocnerodrilid earthworm genus from southeastern Mexico (Annelida: Oligochaeta), with a key for the genera of Ocnerodrilidae. Megadrilogica 13(9): 141–152.

[B136] García-Garza ME León-González JA Tovar-Hernández MA (2019) Catalogue of *Notomastus* M. Sars, 1851 (Annelida, Capitellidae) and the description of a new species from the Gulf of California. Zootaxa 4577(2): 249–273. 10.11646/zootaxa.4577.2.2 31715721

[B137] Gates GE Reynolds JW (2017) Preliminary key to North American megadriles (Annelida, Oligochaeta), based on external characters, insofar as possible. Megadrilogica 22(10): 201–219.

[B138] GBIF.org (2023) GBIF Home Page. https://www.gbif.org [Accessed Dec. 2023]

[B139] GBIF Secretariat (2025) GBIF Backbone Taxonomy. https://www.gbif.org/species/search [Accessed 22 Jan. 2025]

[B140] Gelder SR (1996) A review of the taxonomic nomenclature and a checklist of the species of the Branchiobdellae (Annelida: Clitellata). Proceedings of the Biological Society of Washington 109: 653–663.

[B141] Giangrande A Gambi MC Micheli F Kroeker KJ (2014) Fabriciidae (Annelida, Sabellida) from a naturally acidified coastal system (Italy) with description of two new species. Journal of the Marine Biological Association of the United Kingdom 94: 1417–1427. 10.1017/S0025315414000678

[B142] Giangrande A Licciano M Wasson B (2015) Guide to identification of Sabellidae and Fabriciidae (Polychaeta) in north east Atlantic and Mediterranean waters. NMBAQC 2014 taxonomic workshop, Dove Marine Laboratory, Cullercoats, UK, 91 pp. http://www.nmbaqcs.org/scheme-components/invertebrates/literature-and-taxonomic-keys/sabellid-guide/

[B143] Gil J (2011) The European fauna of AnnelidaPolychaeta. PhD Thesis, Universidade de Lisboa, 1554 pp. https://repositorio.ul.pt/handle/10451/4600

[B144] Glasby CJ (1993) Family revision and cladistic analysis of the Nereidoidea (Polychaeta: Phyllodocida). Invertebrate Taxonomy 7: 1551–1573. 10.1071/IT9931551

[B145] Glasby CJ Fauchald K (2003) POLiKEY. An information system for polychaete families and higher taxa. Version 2, 5 June 2003. Australian Biological Resources Study, Department of Climate Change, Energy, the Environment and Water, Australian Government, Canberra. https://www.dcceew.gov.au/science-research/abrs/online-resources/polikey

[B146] Glasby CJ Hutchings PA (2014) Revision of the taxonomy of *Polycirrus* Grube, 1850 (Annelida: Terebellida: Polycirridae). Zootaxa 3877(1): 1–117. 10.11646/zootaxa.3877.1.1 25544345

[B147] Glasby CJ Salazar-Vallejo SI (2022) 7.13.3.4 Pilargidae Saint-Joseph, 1899. In: Purschke G Böggemann M Westheide W (Eds) Handbook of Zoology, Annelida, Volume 3: Pleistoannelida, Errantia II.De Gruyter, Berlin, 308–323. 10.1515/9783110647167-011

[B148] Glasby CJ Biriukova O Martin P Utevsky S Wilson RS (2025a) ANNiKEY Interactive–a taxonomic information system and a multi-access identification key to Annelida family-level taxa. https://zenodo.org/records/13738486 10.3897/zookeys.1247.137606 PMC12344570 40808970

[B149] Glasby CJ Biriukova O Martin P Utevsky S Wilson RS (2025b) ANNiKEY Online – A taxonomic information system and glossary for the Annelida. 10.5281/zenodo.16392356 [all versions]

[B150] Gonzalez BC Martínez A Borda E Iliffe TM Eibye-Jacobsen D Worsaae K (2018) Phylogeny and systematics of Aphroditiformia. Cladistics 34: 225–259. 10.1111/cla.12202 34645076

[B151] Gonzalez BC Worsaae K Eibye-Jacobsen D (2022) 7.13.1.5.1. Sigalionidae, Pisioninae Ehlers, 1901. In: Purschke G Böggemann M Westheide W (Eds) Handbook of Zoology, Annelida, Volume 4: Pleistoannelida, Errantia II.De Gruyter, Berlin, 138–151. 10.1515/9783110647167-007

[B152] Göpper BM Voogt NM Ganswindt A (2018) First record of the marine turtle leech (*Ozobranchusmargoi*) on hawksbill turtles (*Eretmochelysimbricata*) in the inner granitic Seychelles. Onderstepoort Journal of Veterinary Research 85(1): e1–e3. [PMID: 30198282; PMCID: PMC6238800] 10.4102/ojvr.v85i1.1604 PMC6238800 30198282

[B153] Goto R Monnington J Sciberras M Hirabayashi I Rouse GW (2020) Phylogeny of Echiura updated, with a revised taxonomy to reflect their placement in Annelida as sister group to Capitellidae. Invertebrate Systematics 34: 101–111. 10.1071/IS19020

[B154] Govedich FR Bain BA Moser WE Gelder SR Davies RW Brinkhurst RO (2009) Annelida (Clitellata): Oligochaeta, BranchiobdellidaHirudinida, and Acanthobdellida. In: Thorp JH Covich AP (Eds) Ecology and Classification of North American Freshwater Invertebrates, 3rd Ed.Academic Press, San Diego, 385–436. 10.1016/B978-0-12-374855-3.00012-1

[B155] Graff L von (1884) Report on the Myzostomida collected during the voyage of H.M.S. Challenger during the years 1873–1876. Report on the Scientific Results of the Voyage of H.M.S. Challenger during the years 1873–76. Zoology 10(27): 1–82. [pls 1–16] https://www.biodiversitylibrary.org/page/49395731

[B156] Gravina MF Pierri C Mercurio M Nonnis Marzano C Giangrande A (2021) Polychaete Diversity Related to Different Mesophotic Bioconstructions along the Southeastern Italian Coast. Diversity 13: 239. 10.3390/d13060239

[B157] Gravina MF Somaschini A (1990) Censimento dei Policheti dei Mari Italiani: Capitellidae Grube, 1862. Atti della Società Toscana di Scienze Naturali di Pisa. Memorie Series B, 97: 259–285.

[B158] Green KD (2002) Capitellidae (Polychaeta) from the Andaman Sea. Phuket Marine Biological Center Special Publication 24: 249–343.

[B159] Grosser C (2020) First Record of *Xerobdellaanulata* Autrum, 1958 (Hirudinida: Xerobdellidae) from Serbia. Biologia Serbica 42: 10–13.

[B160] Grosser C Nesemann HF Pešić V (2011) *Dina orientalis* sp. nov. – an overlooked new leech (Annelida: Hirudinea: Erpobdellidae) species from the Near and Middle East. Zootaxa 2746: 20–24. 10.11646/zootaxa.2746.1.2

[B161] Gunton LM Zhang W Kupriyanova EK Hutchings PA (2023) New Species of *Melinna* (Melinnidae, Annelida) from the Australian Abyss with Comments on *M.albicincta*, *M.cristata* and *M.elisabethae*. Records of the Australian Museum 75: 125–154. 10.3853/j.2201-4349.75.2023.1803

[B162] Halanych KM Cox LN Struck TH (2007) A brief review of holopelagic annelids. Integrative Comparative Biology 47: 872–879. 10.1093/icb/icm086 21669766

[B163] Hanley JR Burke M (1991) PolychaetaPolynoidae: Scaleworms of the Chesterfield Islands and Fairways Reefs, Coral Sea. In: Crosnier A (Ed.) Résultats des Campagnes MUSORSTOM, Volume 8.Mémoires Museum national Histoire, naturelle (A) 151: 9–82.

[B164] Harding WA (1913) No. III–On a new land-leech from the Seychelles. Transactions of the Linnean Society of London, 2^nd^ Series. Zoology 16: 39–44. 10.1111/j.1096-3642.1914.tb00123.x

[B165] Harman WJ (1969) Revision of the Family Opistocystidae (Oligochaeta). Transactions of the American Microscopical Society 88: 472–478. 10.2307/3224240

[B166] Harman WJ Loden MS (1984) *Capilloventeratlanticus* gen. et sp. n., a member of a new family of marine Oligochaeta from Brazil. Hydrobiologia 115: 51–54. 10.1007/BF00027892

[B167] Hartley JP (1981) The family Paraonidae (Polychaeta) in British waters: a new species and new records with a key to species. Journal of the Marine Biological Association of the United Kingdom 61: 133–149. 10.1017/S0025315400045975

[B168] Helm C Vila I Budaeva N (2021) 7.12.5 Histriobdellidae Claus & Moquin-Tandon, 1884. In: Purschke G Böggemann M Westheide W (Eds) Handbook of Zoology, Annelida, Volume 3: Pleistoannelida, Sedentaria III and Errantia I.De Gruyter, Berlin, 452–459. 10.1515/9783110291704-021

[B169] Hermans CO (1969) The systematic position of Archiannelida. Systematic Biology 18: 85–102. 10.1093/sysbio/18.1.85

[B170] Hernández-Alcántara P García-Garza ME Solís-Weiss V (2022) *Notomastusbermejoi*, a new species of Capitellidae (Annelida, Polychaeta) from the Gulf of California, with morphological remarks on species with hooks in thoracic chaetigers. ZooKeys 1102: 43–58. 10.3897/zookeys.1102.83198 36761148 PMC9848876

[B171] Hessle C (1917) Zur Kenntnis der terebellomorphen Polychaeten. Zoologiska Bidrag från Uppsala 5: 39–258. [plates I–V] https://biodiversitylibrary.org/page/38891407

[B172] Holt PC Opell BD (1993) A checklist of and illustrated key to the genera and species of the central and North American Cambarincolidae (Clitellata: Branchiobdellida). Proceedings of the Biological Society of Washington 106(2): 251–295.

[B173] Holthe T (1986) Evolution, systematics, and distribution of the PolychaetaTerebellomorpha, with a catalogue of the taxa and a bibliography. Gunneria 55: 1–236. http://www.diva-portal.org/smash/get/diva2:331918/FULLTEXT01.pdf

[B174] Hove HA ten Kupriyanova EK (2009) Taxonomy of Serpulidae (Annelida, Polychaeta): The state of affairs. Zootaxa 2036: 1–126. 10.11646/zootaxa.2036.1.1

[B175] Hsueh P-W (2022) Three new species of *Platynereis* (Annelida, Polychaeta, Nereididae) from Taiwan. Zoological Studies 61: 30. 10.6620/ZS.2022.61-30 PMC9522625 36245912

[B176] Hsueh P-W Li Y-H (2014) New species and new records of eunicids (Polychaeta, Eunicidae) from Taiwan. Zootaxa 3802(2): 151–172. 10.11646/zootaxa.3802.2.1 24871000

[B177] Huč S Hiley AS McCowin MF Rouse GW (2024) A Mitogenome-Based Phylogeny of Pilargidae (Phyllodocida, Polychaeta, Annelida) and Evaluation of the Position of *Antonbruunia*. Diversity 16(3): 134. 10.3390/d16030134

[B178] Hutchings PA McRae J (1993) The Aphroditidae (Polychaeta) from Australia, together with a redescription of the Aphroditidae collected during the Siboga Expedition. Records of the Australian Museum 45(3): 279–363. 10.3853/j.0067-1975.45.1993.24

[B179] Hutchings PA Nogueira JMM Carrerette O (2021b) 7.7.3 Terebellidae s.l.: Polycirridae Malmgren, 1866, Terebellidae Johnston, 1846, Thelepodidae Hessle, 1917, Trichobranchidae Malmgren, 1866, and Telothelepodidae Nogueira, Fitzhugh & Hutchings, 2013. In: Purschke G Böggemann M Westheide W (Eds) Handbook of Zoology, Annelida, Volume 3: Pleistoannelida, Sedentaria III and Errantia I.De Gruyter, Berlin, 68–144. 10.1515/9783110291704-006

[B180] Hutchings PA Peart R (2000) A revision of the Australian Trichobranchidae (Polychaeta). Invertebrate Systematics 14: 225–272. 10.1071/IT98005

[B181] Hutchings PA Peart R (2002) A review of the genera of Pectinariidae (Polychaeta) together with a description of the Australian fauna. Records of the Australian Museum 54: 99–127. 10.3853/j.0067-1975.54.2002.1356

[B182] Hutchings PA Carrerette O Nogueira JMM Hourdez S Lavesque N (2021a) The Terebelliformia – recent developments, future directions. Diversity 13: 60. 10.3390/d13020060

[B183] Ibrahim NF Villalobos-Guerrero TF Idris I (2024) Review of Oweniidae Rioja, 1917 (Annelida, Palaeoannelida) from Malaysia, with a description of two new species and a key to South China Sea species. Zoosystema 46(20): 513–542. 10.5252/zoosystema2024v46a20

[B184] Imajima M Hartman O (1964) The polychaetous annelids of Japan. Occasional Papers of the Allan Hancock Foundation 26: 1–452. http://digitallibrary.usc.edu/cdm/ref/collection/p15799coll82/id/18946

[B185] James SW (2000) An Illustrated Key to the Earthworms of The Samoan Archipelago (Oligochaeta: Glossoscolecidae, Moniligastridae). Technical Report No. 49, 11 pp.

[B186] James SW (2004) An illustrated key to the earthworms of the Samoan Archipelago (Oligochaeta: Glossoscolecidae, Megascolecidae, Moniligastridae). Micronesica 37(1): 1–13.

[B187] James SW Davidson SK (2012) Molecular phylogeny of earthworms (Annelida: Crassiclitellata) based on 28S, 18S and 16S gene sequences. Invertebrate Systematics 26: 213–229. 10.1071/IS11012

[B188] James J Cable J Richardson G Davidson KE Mackie ASY (2015) Two alien species of Branchiobdellida (Annelida: Clitellata) new to the British Isles: a morphological and molecular study. Aquatic Invasions 10: 371–383. 10.3391/ai.2015.10.4.02

[B189] Jamieson BGM (1978) Phylogenetic and phenetic systematics of the opisthoporous Oligochaeta (Annelida: Clitellata). Evolutionary Theory 3: 195–233.

[B190] Jamieson BGM (2000) Native Earthworms of Australia (Megascolecidae, Megascolecinae). Science Publishers, Inc., Enfield, New Hampshire, USA.

[B191] Jamieson BGM (2006) Non-leech Clitellata (with contributions by Marco Ferraguti). In: Rouse G Pleijel F (Eds) Reproductive Biology and Phylogeny of Annelida.Series Editor BGM Jamieson. Volume 4. Science Publishers, Enfield, New Hampshire, 235–392.

[B192] Jamieson BGM Fragoso C (2024) A monograph of the Oligochaete family Alluroididae. Zootaxa 5529(3): 401–435. 10.11646/zootaxa.5529.3.1 39646403

[B193] Jamieson BGM Tillier S Tillier A Justine J-L Ling E James S McDonald K Hugall AF (2002) Phylogeny of the Megascolecidae and Crassiclitellata (Annelida, Oligochaeta): combined versus partitioned analysis using nuclear (28S) and mitochondrial (12S, 16S) rDNA. Zoosystema 24: 707–734.

[B194] Jiménez-Cueto S Suárez-Morales E (1999) Tomopterids (Polychaeta: Tomopteridae) of the Western Caribbean Sea. Bulletin de L’institut Royal des Sciences Naturelles de Belgique, Biologie 69: 5–14.

[B195] Jiménez-Cueto S Suárez-Morales E (2008) An account of *Alciopina*, *Torrea*, and *Rhynconereella* (Polychaeta: Alciopidae) of the western Caribbean Sea. Belgium Journal of Zoology 138(1): 70–80.

[B196] Jimi N Hookabe N Fujimoto S Kise H Ogawa A Tsuchiya M (2023) Three species of Fauveliopsidae (Annelida) from the north-western Pacific including a new species. Plankton Benthos Research 18(4): 178–184. 10.3800/pbr.18.178

[B197] Jirkov IA (2013) Identification keys for Terebellomorpha (Polychaeta) of the Eastern Atlantic and the North Polar Basin. II. Ampharetidae. NMBAQC 2008 taxonomic workshop, Dove Marine Laboratory, Cullercoats, UK, 6 pp. http://www.nmbaqcs.org/scheme-components/invertebrates/literature-and-taxonomic-keys/terebellomorpha-polychaeta-part-ii/

[B198] Jirkov IA Leontovich MK (2013) Identification keys for Terebellomorpha (Polychaeta) of the eastern Atlantic and the North Polar Basin. Invertebrate Zoology 10: 217–243. 10.15298/invertzool.10.2.02

[B199] Jollivet D Hourdez S (2021) 7.7.4 Alvinellidae Desbruyères & Laubier, 1986. In: Purschke G Böggemann M Westheide W (Eds) Handbook of Zoology, Annelida, Volume 3: Pleistoannelida, Sedentaria III and Errantia I.De Gruyter, Berlin, 145–162. 10.1515/9783110291704-007

[B200] Jumars PA Dorgan KM Lindsay SM (2015) Diet of Worms Emended: An Update of Polychaete Feeding Guilds. Annual Reviews of Marine Science 7: 497–520. 10.1146/annurev-marine-010814-020007 25251269

[B201] Khomenko A Utevsky S Utevsky A Trontelj P (2020) Unrecognized diversity of *Trocheta* species (Hirudinea: Erpobdellidae): resolving a century-old taxonomic problem in Crimean leeches. Systematics and Biodiversity 18: 129–141. 10.1080/14772000.2020.1739776

[B202] Kirtley DW (1994) A review and taxonomic revision of the family Sabellariidae Johnston, 1865 (AnnelidaPolychaeta). Science Series 1: 1–223. Sabecon Press, Vero Beach, Florida.

[B203] Kolbasova G Kosobokova K Neretina T (2020) Bathy- and mesopelagic Annelida from the Arctic Ocean: description of new, redescription of known and notes on some cosmopolitan species. Deep Sea Research, part I: Oceanographic Research Papers 165: 103–327. 10.1016/j.dsr.2020.103327

[B204] Kudenov J (1993) Amphinomidae and Euphrosinidae (Annelida: Polychaeta) Principally from Antarctica, The Southern Ocean, and Subantarctic Regions. Antarctic Research Series 58: 93–150. 10.1029/AR058p0093

[B205] Kuo D-H Lai Y-T (2019) On the origin of leeches by evolution of development. Development, Growth and Differentiation 61: 43–57. 10.1111/dgd.12573 30393850

[B206] Kupriyanova EK Rouse GW (2008) Yet another example of paraphyly in Annelida: molecular evidence that Sabellidae contains Serpulidae. Molecular Phylogenetics and Evolution 46: 1174–1181. 10.1016/j.ympev.2007.10.025 18262800

[B207] Kupriyanova EK Wong E Hutchings PA [Eds] (2013) Invasive Polychaete Identifier – an Australian perspective. Version 1.1, 04 Dec 2013. http://polychaetes.australianmuseum.net.au/

[B208] Kupriyanova EK Sun Y Hove HA ten Wong E Rouse GW (2015) Serpulidae (Annelida) of Lizard Island, Great Barrier Reef, Australia. Zootaxa 4019: 275–353. 10.11646/zootaxa.4019.1.13 26624073

[B209] Kupriyanova EK Rzhavsky AV ten Hove HA (2019) 7.4.7 Serpulidae Rafinesque, 1815. In: Purschke G Böggemann M Westheide W (Eds) Handbook of Zoology, Annelida, Volume 2: Pleistoannelida, Sedentaria II.De Gruyter, Berlin, 213–275. 10.1515/9783110291681-006

[B210] Kupriyanova E Hove HA ten Rouse GW (2023) Phylogeny of Serpulidae (Annelida, Polychaeta) Inferred from Morphology and DNA Sequences, with a New Classification. Diversity 15: 398. 10.3390/d15030398

[B211] Kuş S Kurt G Çinar ME (2021) Nephtyidae (Annelida: Polychaeta) from the Sea of Marmara and Black Sea, with descriptions of two new species. Zootaxa 5060: 33–64. 10.11646/zootaxa.5060.2.2 34811176

[B212] Kutschera U Pfeiffer I Ebermann E (2007) The European land leech: biology and DNA-based taxonomy of a rare Species that is threatened by climate warming. Naturwissenschaften 94: 967–974. 10.1007/s00114-007-0278-3 17646954

[B213] Lai Y Nakano T (2011) Three species of land leeches from Taiwan, *Haemadipsarjukjuana* comb. n., a new record for *Haemadipsapicta* Moore, and an updated description of *Tritetrabdellataiwana* (Oka). ZooKeys 139: 1–22. 10.3897/zookeys.139.1711 PMC3260908 22259307

[B214] Lattig P Martin D San Martin G (2010) Syllinae (Syllidae: Polychaeta) from Australia. Part 4. The genus *Haplosyllis* Langerhans, 1879 (Polychaeta: Syllidae: Syllinae). Zootaxa 2552: 1–36. 10.11646/zootaxa.2552.1.1

[B215] Lavesque N Hutchings P Daffe G Nygren A Londoño-Mesa MH (2019) A revision of the French Trichobranchidae (Polychaeta), with descriptions of nine new species. Zootaxa 4664: 151–190. 10.11646/zootaxa.4664.2.1 31716675

[B216] Lavesque N Londoño-Mesa MH Daffe G Hutchings P (2020) A revision of the French Telothelepodidae and Thelepodidae (Annelida, Terebelliformia), with descriptions of three species and first European record of a non-indigenous species. Zootaxa 4810: 305–327. 10.11646/zootaxa.4810.2.4 33055897

[B217] Lavesque N Daffe G Londoño-Mesa MH Hutchings P (2021a) Revision of the French Terebellidae sensu stricto (Annelida, Terebelliformia), with descriptions of nine new species. Zootaxa 5038: 1–63. 10.11646/zootaxa.5038.1.1 34811100

[B218] Lavesque N Hutchings P Londoño-Mesa MH Nogueira JMM Daffe G Nygren A Blanchet H Bonifácio P Broudin C Dauvin J-C Droual G Gouillieux B Grall J Guyonnet B Houbin C Humbert S Janson A-L Jourde J Labrune C Lamarque B Latry L Le Garrec V Pelaprat C Pezy J-P Sauriau P-G De Montaudouin X (2021b) The “Spaghetti Project”: the final identification guide to European Terebellidae (sensu lato) (Annelida, Terebelliformia). European Journal of Taxonomy 782(1): 108–156. 10.5852/ejt.2021.782.1593

[B219] Lavesque N Nogueira JMM Daffe Gu Hutchings P (2024) Five new species of *Terebellides* (Annelida, Polychaeta, Trichobranchidae) from Papua New Guinea (Bismarck and Solomon seas). Frontiers in Ecology and Evolution 12: 1349362. 10.3389/fevo.2024.1349362

[B220] Leidy J (1884) [1883 year] *Manayunkiaspeciosa*. Journal of the Academy of Natural Sciences of Philadelphia 35: 204–212. [1 plate] https://biodiversitylibrary.org/page/26279862

[B221] Li YN Kocot KM Schander C Santos SR Thornhill DJ Halanych KM (2015) Mitogenomics reveals phylogeny and repeated motifs in control regions of the deep-sea family Siboglinidae (Annelida). Molecular Phylogenetics and Evolution 85(2015): 221–229. 10.1016/j.ympev.2015.02.008 25721539

[B222] Li YN Kocot KM Whelan NV Santos SR Waits DS Thornhill DJ Halanych KM (2017) Phylogenomics of tubeworms (Siboglinidae, Annelida) and comparative performance of reconstruction methods. Zoologica Scripta 46: 200–213. 10.1111/zsc.12201

[B223] Locke JM Coates KA (2000) An illustrated key to the species of *Grania* and *Randidrilus* (Annelida: Clitellata: Enchytraeidae) of eastern North America, Bermuda, and the Caribbean area. Proceedings of the Biological Society of Washington 113: 617–632.

[B224] Mackie ASY (1990) The Poecilochaetidae and Trochochaetidae (Annelida: Polychaeta) of Hong Kong. Proceedings of the Second International Marine Biological Workshop: The Marine Flora and Fauna of Hong Kong and Southern China, Hong Kong, 1986: 337–362. 10.1515/9789882202177-009

[B225] Mackie ASY (2019) 7.2.1 Aberrantidae Wolf, 1987. In: Purschke G Böggemann M Westheide W (Eds) Handbook of Zoology, Annelida, Volume 1: Annelida Basal Groups and Pleistoannelida, Sedentaria I.De Gruyter, Berlin, 269–273.

[B226] Mackie ASY Pleijel F Rouse GW (2005) Revision of *Aberranta* (Aberrantidae: Annelida), with descriptions of new species from the Mediterranean and Hong Kong. Marine Ecology 26: 197–208. 10.1111/j.1439-0485.2005.00064.x

[B227] Mackie ASY Oliver PG Nygren A (2015) *Antonbruuniasociabilis* sp. nov. (Annelida: Antonbruunidae) associated with the chemosynthetic deep-sea bivalve *Thyasirascotiae* Oliver & Drewery, 2014, and a re-examination of the systematic affinities of Antonbruunidae. Zootaxa 3995(1): 20–36. 10.11646/zootaxa.3995.1.4 26250300

[B228] Magalhães WF Bailey-Brock JH (2011) A new species of *Acrocirrus* (Polychaeta: Acrocirridae) from Coconut Island, Oahu, Hawaii. Journal of the Marine Biological Association of the United Kingdom 92: 1019–1022. 10.1017/S0025315411000634

[B229] Magalhães WF Brock JH Davenport J (2011) On the genus *Raphidrilus* Monticelli, 1910 (Polychaeta: Ctenodrilidae) with description of two new species. Zootaxa 2804(1): 1–14. 10.11646/zootaxa.2804.1.1

[B230] Magalhães WF Rizzo AE Bailey-Brock JH (2019) Opheliidae (Annelida: Polychaeta) from the western Pacific islands, including five new species. Zootaxa 4555(2): 209–235. 10.11646/zootaxa.4555.2.3 30790958

[B231] Mann KH (1962) Leeches (Hirudinea): Their Structure, Physiology, Ecology and Embryology. With an Appendix on the Systematics of Marine Leeches. Pergamon Press, New York, 201 pp. 10.5962/bhl.title.7350

[B232] Marchán DF Fernández R de Sosa I Sánchez N Díaz Cosín DJ Novo M (2018a) Integrative systematic revision of a Mediterranean earthworm family: Hormogastridae (Annelida, Oligochaeta). Invertebrate Systematics 32: 652–671. 10.1071/IS17048

[B233] Marchán DF Fernández R Sánchez N de Sosa I Díaz Cosín DJ Novo M (2018b) Insights into the diversity of Hormogastridae (Annelida, Oligochaeta) with descriptions of six new species. Zootaxa 4496(1): 65. 10.11646/zootaxa.4496.1.6 30313686

[B234] Marchán DF James SW Lemmon AR Lemmon EM Novo M Domínguez J Díaz Cosín DJ Trigo D (2022) A strong backbone for an invertebrate group: anchored phylogenomics improves the resolution of genus level relationships within the Lumbricidae (Annelida, Crassiclitellata). Organisms Diversity & Evolution 22: 915–924. 10.1007/s13127-022-00570-y

[B235] Marchán DF Martínez Navarro A Gérard S Decaëns T Novo M (2024) Ancient diversity within *Diporodrilus* (Crassiclitellata, Annelida) clarify the historical biogeography of Corso-Sardinian earthworms. Organisms Diversity & Evolution 24: 163–179. 10.1007/s13127-024-00639-w

[B236] Martin P Aït Boughrous A (2012) Guide taxonomique des oligochètes dulçaquicoles du Maghreb. ABC Taxa 12, 186 pp.

[B237] Martin D Britayev T (1998) Symbiotic polychaetes: review of known species. Oceanography and Marine Biology: An Annual Review 36: 217–340.

[B238] Martin D Britayev T (2018) Symbiotic polychaetes revisited: an update of the known species and relationships (1998–2017) Oceanography and Marine Biology: An Annual Review 56: 371–447. 10.1201/9780429454455-6

[B239] Martin P Martinez-Ansemil E Pinder A Timm T Wetzel MJ (2008) Global diversity of oligochaetous clitellates (‘‘Oligochaeta’’, Clitellata) in freshwater. Hydrobiologia 595: 117–127. 10.1007/s10750-007-9009-1

[B240] Martin P Martínez-Ansemil E Sambugar B (2010) The Baikalian genus *Rhyacodriloides* in Europe: phylogenetic assessment of Rhyacodriloidinae subfam. n. within the Naididae (Annelida). Zoologica Scripta 39: 462–482. 10.1111/j.1463-6409.2010.00434.x

[B241] Martin P Knüsel M Alther R Altermatt F Ferrari B Vivien R (2023a) *Haplotaxisgordioides* (Hartmann in Oken, 1819) (Annelida, Clitellata) as a sub-cosmopolitan species: a commonly held view challenged by DNA barcoding. Zoosymposia 23: 78–93. 10.11646/zoosymposia.23.1.10

[B242] Martin P Reynolds J van Haaren T (2023b) World List of Marine Oligochaeta. *Pristina* Ehrenberg, 1831. https://www.marinespecies.org/aphia.php?p%20=%20taxdetails&id%20=%20475503 [Accessed 18 Oct 2023]

[B243] Martin P Fend S Martinsson S Klinth M Torii T Erséus C (2024) Towards an integrative revision of Haplotaxidae (Annelida: Clitellata). Zoological Journal of the Linnean Society 202: zlae141. 10.1093/zoolinnean/zlae141

[B244] Martín-Durán JM Vellutini BC Marlétaz F Cetrangolo V Cvetesic N Thiel D Henriet S Grau-Bové X Carrillo-Baltodano AM Gu W Kerbl A Marquez Y Bekkouche N Chourrout D Gómez-Skarmeta JL Irimia M Lenhard B Worsaae K Hejnol A (2021) Conservative route to genome compaction in a miniature annelid. Nature Ecology and Evolution 5: 231–242. 10.1038/s41559-020-01327-6 33199869 PMC7854359

[B245] Martínez A Worsaae K Núñez J (2019) 7.3.1.7 Acrocirridae Banse, 1968. In: Purschke G Böggemann M Westheide W (Eds) Handbook of Zoology, Annelida, Volume 1: Annelida Basal Groups and Pleistoannelida, Sedentaria I.De Gruyter, Berlin, 422–439.

[B246] Martínez A Purschke G Worsaae K (2021a) 7.11.3 Protodrilidae Hatschek, 1888. In: Purschke G Böggemann M Westheide W (Eds) Handbook of Zoology, Annelida, Volume 3: Pleistoannelida, Sedentaria III and Errantia I.De Gruyter, Berlin, 299–337. 10.1515/9783110291704-015

[B247] Martínez A Worsaae K Purschke G (2021b) 7.11.4 Protodriloididae Purschke & Jouin,1988. In: Purschke G Böggemann M Westheide W (Eds) Handbook of Zoology, Annelida, Volume 3: Pleistoannelida, Sedentaria III and Errantia I.De Gruyter, Berlin, 338–353. 10.1515/9783110291704-016

[B248] Martínez-Ansemil E (1993) Etudes sur les oligochètes aquatiques des pays du pourtour de la Méditerranée: taxonomie, phylogénie, biogéographie et écologie. Thèse de Docteur d’Etat (Sciences), Université Paul Sabatier, Toulouse, 616 pp.

[B249] Martins R Carrera-Parra LF Quintino V Rodrigues AM (2012) Lumbrineridae (Polychaeta) from the Portuguese continental shelf (NE Atlantic) with the description of four new species. Zootaxa 3416: 1–21. 10.11646/zootaxa.3416.1.1

[B250] McIntosh WC (1900–1922) A monograph of the British marine annelids. Vols I–IV. The Ray Society, London. [Contents: I. Nemertinea and Polychaeta: Amphinomidae to Sigalionidae. 1873–1900.—II. Polychaeta; Nephthydidae to Ariciidae. 1908–10.—III. Polychaeta: Opheliidae to Ammocharidae. 1915.—IV. Polychaeta: Hermellidae to Serpulidae, with additions to the British marine Polychaeta during the publication of the monograph, 1922–23].

[B251] McMahan ML (1979) Anatomical notes on *Lutodrilusmultivesiculatus* (Annelida: Oligochaeta). Proceedings of the Biological Society of Washington 92(1): 84–97.

[B252] Meißner K Hutchings PA (2003) *Spiophanes* species (Polychaeta: Spionidae) from Eastern Australia—with descriptions of new species, new records and an emended generic diagnosis. Records of the Australian Museum 55: 117–140. 10.3853/j.0067-1975.55.2003.1379

[B253] Meißner K Schwentner M Götting M Knebelsberger T Fiege D (2023) Polychaetes distributed across oceans—examples of widely recorded species from abyssal depths of the Atlantic and Pacific Oceans. Zoological Journal of the Linnean Society 199: 906–944. 10.1093/zoolinnean/zlad069

[B254] Mendes SL da SD de Paiva PC Rizzo AE (2024) New species of *Pseudoscalibregma* Ashworth, 1901 (Annelida: Scalibregmatidae Malmgren, 1867) from Brazil. Zootaxa 5399(1): 19–36. 10.11646/zootaxa.5399.1.2 38221178

[B255] Meyer A Bleidorn C Rouse GW Hausen H (2008) Morphological and molecular data suggest a cosmopolitan distribution of the polychaete *Proscoloploscygnochaetus* Day, 1954 (Annelida, Orbiniidae). Marine Biology 153: 879–889. 10.1007/s00227-007-0860-4

[B256] Misirlioğlu M Reynolds JW Stojanoviæ M Trakić T Sekulić J James SW Csuzdi C Decaëns T Lapied E Phillips HRP Cameron EK Brown GG (2023) Earthworms (Clitellata, Megadrili) of the world: an updated checklist of valid species and families, with notes on their distribution. Zootaxa 5255(1): 417–438. 10.11646/zootaxa.5255.1.33 37045245

[B257] Mortimer K (2019) 4.2 Magelonidae Cunningham & Ramage, 1888. In: Purschke G Böggemann M Westheide W (Eds) Handbook of Zoology, Annelida, Volume 1: Annelida Basal Groups and Pleistoannelida, Sedentaria I.De Gruyter, Berlin, 112–131.

[B258] Mortimer K Gil J Fiege D (2011) Portuguese *Magelona* (Annelida: Magelonidae) with a description of a new species, a re-description of *Magelonawilsoni* Glémarec, 1966 and a key to adult Magelonidae from European waters. Italian Journal of Zoology 78(S1): 1–16. 10.1080/11250003.2011.583449

[B259] Mortimer K Mills K Jordana E Pinedo S Gil J (2020) A further review of European Magelonidae (Annelida), including descriptions of *Magelonaequilamellae* and *Magelonafiliformis*. Zootaxa 4767: 89–114. 10.11646/zootaxa.4767.1.4 33056574

[B260] Mortimer K Fitzhugh K dos Brasil AC Lana P (2021) Who’s who in *Magelona*: phylogenetic hypotheses under Magelonidae Cunningham & Ramage, 1888 (Annelida: Polychaeta). PeerJ 9: e11993. 10.7717/peerj.11993 PMC8759375 35070516

[B261] Nakano T (2011) Holotype redescription of *Mimobdellajaponica* (Hirudinida, Arhynchobdellida, Erpobdelliformes) and taxonomic status of the genus *Mimobdella*. ZooKeys 119: 1–10. 10.3897/zookeys.119.1501 PMC3192424 21998513

[B262] Nakano T (2016a) A new quadrannulate species of *Orobdella* (Hirudinida, Arhynchobdellida, Orobdellidae) from western Honshu, Japan. ZooKeys 553: 33–51. 10.3897/zookeys.553.6723 PMC4740979 26877670

[B263] Nakano T (2016b) Four new species of the genus *Orobdella* from Shikoku and Awajishima island, Japan (Hirudinida, Arhynchobdellida, Orobdellidae). Zoosystematics and Evolution 92: 79–102. 10.3897/zse.91.7616

[B264] Nakano T Nguyen ST (2015) A new predatory leech from Vietnam (Hirudinida: Arhynchobdellida: Salifidae): its phylogenetic position with comments on the classification of the family. Invertebrate Systematics 29: 473–486. 10.1071/IS15008

[B265] Nakano T Prozorova L (2019) A new species of *Orobdella* (Hirudinida: Arhynchobdellida: Orobdellidae) from Primorye Territory, Russian Far East. Journal of Natural History 53(5–6): 351–364. 10.1080/00222933.2019.1593539

[B266] Nakano T Ramlah Z Hikida T (2012) Phylogenetic position of gastrostomobdellid leeches (Hirudinida, Arhynchobdellida, Erpobdelliformes) and a new family for the genus *Orobdella*. Zoologica Scripta 41: 177–185. 10.1111/j.1463-6409.2011.00506.x

[B267] Nakano T Tomikawa K Sakono T Yoshikawa N (2017) Praobdellidae (Hirudinida: Arhynchobdellida) is not specific only to the mucous-membrane after all: Discovery of a praobdellid leech feeding on the Japanese freshwater crab *Geothelphusadehaani*. Parasitology International 66: 210–213. 10.1016/j.parint.2017.01.018 28137668

[B268] Nakano T Eto K Nishikawa K Hossmann MY Jeratthitikule E (2018) Systematic revision of the Southeast Asian macrophagous leeches, with the description of two new gastrostomobdellid species (Hirudinida: Arhynchobdellida: Erpobdelliformes). Zoological Journal of the Linnean Society 184: 1–30. 10.1093/zoolinnean/zlx097

[B269] Narayanan SP Sathrumithra S Christopher G Julka JM (2017) New species and new records of earthworms of the genus *Drawida* from Kerala part of the Western Ghats biodiversity hotspot, India (Oligochaeta, Moniligastridae). ZooKeys 691: 1–18. 10.3897/zookeys.691.13174 PMC5672664 29200921

[B270] Nesemann H Sharma S (2001) Leeches of the suborder Hirudiniformes (Hirudinea: Haemopidae, Hirudinidae, Haemadipsidae) from the Ganga watershed (Nepal, India: Bihar). Annalen des Naturhistorisches Museum Wien 103B: 77–88.

[B271] Nesemann H Sharma G (2012) Description of a new species of the leech family Salifidae (*Odontobdellakrishna* sp. nov.) from the River Ganga at Patna, Bihar (India). Records of the Zoological Survey of India 111: 1–7. 10.26515/rzsi/v111/i3/2011/158849

[B272] Nilsen R Holthe T (1985) Arctic and Scandinavian Oweniidae (Polychaeta) with a description of *Myriochelefragilis* sp.n., and comments on the phylogeny of the family. Sarsia 70: 17–32. 10.1080/00364827.1985.10420615

[B273] Nogueira JMM Hutchings PA Fukuda MF (2010) Morphology of terebelliform polychaetes (Annelida: Polychaeta: Terebelliformia). Zootaxa 2460: 1–185. 10.11646/zootaxa.2460.1.1

[B274] Nogueira JMM Fitzhugh K Hutchings PA (2013) The continuing challenge of phylogenetic relationships in Terebelliformia (Annelida: Polychaeta). Invertebrate Systematics 27(2): 186–238. 10.1071/IS12062

[B275] Nogueira JMM Hutchings PA Fukuda MF (2015) Terebellidae (Annelida, Terebelliformia) from Lizard Island, Great Barrier Reef, Australia. Zootaxa 4019: 484–576. 10.11646/zootaxa.4019.1.18 26624078

[B276] Norlinder E Nygren A Wiklund H Pleijel F (2012) Phylogeny of scale-worms (Aphroditiformia, Annelida), assessed from 18SrRNA, 28SrRNA, 16SrRNA, mitochondrial cytochrome c oxidase subunit I (COI), and morphology. Molecular Phylogenetics and Evolution 65: 490–500. 10.1016/j.ympev.2012.07.002 22789762

[B277] Núñez J Martínez A (2023) Two new species and records of the genus *Questa* (Annelida: Orbiniidae) from Azores and Canary Islands, Central Atlantic Ocean. Zootaxa 5319: 403–412. 10.11646/zootaxa.5319.3.6 37518223

[B278] Nxele TC Plisko JD Mwabvu T Zishiri TO (2016) A new family Kazimierzidae for the genus *Kazimierzus*, earlier recorded to the composite Microchaetidae (Annelida, Oligochaeta). African Invertebrates 57(2): 111–117. 10.3897/AfrInvertebr.57.10042

[B279] O’Sullivan D (1982) A guide to the pelagic polychaetes of the Southern Ocean and adjacent waters. Australian National Antarctic Research Expedition, Notes 3. Antarctic Division, Department of Science and Technology, Kingston, Tasmania, Australia, 63 pp.

[B280] Oceguera-Figueroa A (2006) A new leech species of *Semiscolex* (Arhynchobdellida: Semiscolecidae) from Lake Catemaco, Veracruz, Mexico, Revista Mexicana Biodiversidad 77(2): 199–203. 10.22201/ib.20078706e.2006.002.331

[B281] Oceguera-Figueroa A Phillips AJ Pacheco-Chaves B Reeves WK Siddall ME (2011) Phylogeny of macrophagous leeches (Hirudinea, Clitellata) based on molecular data and evaluation of the barcoding locus. Zoologica Scripta 40: 194–203. 10.1111/j.1463-6409.2010.00465.x

[B282] Oliveira VM de Magalhães WF Lana P da C (2021) Ten new species of *Phyllodoce* Lamarck, 1818 (Phyllodocidae, Annelida) from Brazil. Zootaxa 4924(1): 1–61. 10.11646/zootaxa.4924.1.1 33756769

[B283] Osborn KJ Haddock SHD Pleijel F Madin LP Rouse GW (2009) Deep-sea, swimming worms with luminescent ‘bombs. Science 325: 964. 10.1126/science.1172488 19696343

[B284] Oug E Bakken T Kongsrud JA (2011) Guide to identification of Flabelligeridae (Polychaeta) in Norwegian and adjacent waters. Norwegian Polychaete Forum Guides Guide.

[B285] Pamungkas J (2024) *Leocratesbitungensis* (Hesionidae, Annelida): a new polychaete species from North Sulawesi, Indonesia. Raffles Bulletin of Zoology 72: 194–202.

[B286] Pancucci-Papadopoulou MA Murina GVV Zenetos A (1999) The Phylum Sipuncula in the Mediterranean Sea. Monographs on Marine Sciences 2. National Centre for Marine Research, Athens, Greece, 109 pp.

[B287] Parapar J (2006) The genera *Myriochele* and *Myrioglobula* (Polychaeta, Oweniidae) in Icelandic waters with the revision of type material of *Myriocheleheeri* Malmgren, 1867, and the description of a new species. Journal of Natural History 40: 523–547. 10.1080/00222930600711758

[B288] Parapar J Moreira J (2015) The Oweniidae (Annelida, Polychaeta) from Lizard Island (Great Barrier Reef, Australia) with the description of two new species of *Owenia* Delle Chiaje, 1844. Zootaxa 4019: 604–620. 10.11646/zootaxa.4019.1.20 26624080

[B289] Parapar J Alós C Núñez J Moreira J López E Aguirrezabalaga F Besteiro C Martínez A (2012) AnnelidaPolychaeta III. In: Ramos MA, et al. (Eds) Fauna Ibérica vol. 36. Museo Nacional de Ciencias Naturales, CSIC, Madrid, Spain, 416 pp.

[B290] Parapar J Mikac B Fiege D (2013) Diversity of the genus *Terebellides* (Polychaeta: Trichobranchidae) in the Adriatic Sea with the description of a new species. Zootaxa 3691: 333–350. 10.11646/zootaxa.3691.3.3 26167589

[B291] Parapar J Moreira J Núñez J Barnich R del Carmen Brito M Fiege D Capaccioni-Azzati R El-Haddad M (2015) AnnelidaPolychaeta IV. In: Fauna Ibérica vol. 41. MA Ramos et al. (Eds). Museo Nacional de Ciencias Naturales, CSIC, Madrid, 416 pp.

[B292] Parapar J Moreira J Martin D (2016) On the diversity of the SE Indo-Pacific species of *Terebellides* (Annelida; Trichobranchidae), with the description of a new species. PeerJ 4: e2313. 10.7717/peerj.2313 PMC4991861 27602280

[B293] Parapar J Adarraga I Aguado MT Aguirrezabalaga F Arias A Besteiro C Bleidorn C Capa M Capaccioni-Azzati R El-Haddad M Fernández-Alamo MA Lopéz E Martinez J Martinez-Ansemil E Moreira J Núñez J Ravara A (2018) Annelida: Polychaeta V. In: Fauna Ibérica, Volume 45, MA Ramos et al. (Eds). Museo Nacional de Ciencias Naturales, CSIC, 631 pp.

[B294] Parapar J Capa M Nygren A Moreira J (2020a) To name but a few: descriptions of five new species of *Terebellides* (Annelida, Trichobranchidae) from the North East Atlantic. ZooKeys 992: 1–58. 10.3897/zookeys.992.55977 33223905 PMC7677295

[B295] Parapar J Palomanes V Helgason GV Moreira J (2020b) Taxonomy and distribution of Pectinariidae (Annelida) from Iceland with a comparative analysis of uncinal morphology. European Journal of Taxonomy 666: 1–32. 10.5852/ejt.2020.666

[B296] Parapar J Al-Kandari M Candás M Moreira J (2021a) A new species of *Polyophthalmus* (Annelida, Opheliidae) from the Arabian Gulf, with an insight on internal anatomy and diversity of the genus. Zootaxa 5052: 501–528. 10.11646/zootaxa.5052.4.3 34810858

[B297] Parapar J Martínez A Moreira J (2021b) On the Systematics and Biodiversity of the Opheliidae and Scalibregmatidae. Diversity 13(2): 87. 10.3390/d13020087

[B298] Parapar J Al-Kandari M Barroso M Moreira J (2023) The genus *Ophelina* Örsted, 1843 (Annelida: Opheliidae) in the coast of Kuwait (northern Indian Ocean), with the description of a new species. European Journal of Taxonomy 870: 1–29. 10.5852/ejt.2023.870.2113

[B299] Paxton H (1986) Generic revision and relationships of the family Onuphidae (Annelida: Polychaeta). Records of the Australian Museum 38: 1–74. 10.3853/j.0067-1975.38.1986.175

[B300] Paxton H (1996) *Hirsutonuphis* (Polychaeta: Onuphidae) from Australia, with a discussion of setal progression in juveniles. Invertebrate Taxonomy 10: 77–96. 10.1071/IT9960077

[B301] Paxton H (2017) Three new species of *Aponuphis* (Annelida: Onuphidae) from eastern Australia. Zootaxa 4344(2): 246–260. 10.11646/zootaxa.4344.2.2 29245630

[B302] Paxton H Budaeva N (2013) *Paradiopatra* (Annelida: Onuphidae) from eastern Australian waters, with the description of six new species. Zootaxa 3686(2): 140–164. 10.11646/zootaxa.3686.2.2 26473212

[B303] Pazoki S Rahimian H Naderloo R Kupriyanova E (2023) Intertidal calcareous tubeworms (Annelida: Serpulidae) of the Persian Gulf and Gulf of Oman. Regional Studies in Marine Science 67: 1–27. 10.1016/j.rsma.2023.103184

[B304] Petersen ME (2000) A new genus of Fauveliopsidae (Annelida: Polychaeta) with a review of its species and redescription of some described taxa. Bulletin of Marine Science. 67(1): 491–515. https://www.ingentaconnect.com/content/umrsmas/bullmar/2000/00000067/00000001/art00041?crawler%20=%20true

[B305] Petersen ME George JD (1991) A new species of *Raricirrus* from northern Europe, with notes on its biology and a discussion of the affinities of the genus (Polychaeta: Ctenodrilidae). Ophelia, Supplement 5: 185–208. 10.1163/9789004629745_019

[B306] Pettibone MH (1963) Marine polychaete worms of the New England region. I. Aphroditidae through Trochochaetidae. Bulletin of the United States National Museum 227: 1–356. https://www.biodiversitylibrary.org/page/7870746

[B307] Pettibone MH (1966) *Heteraphroditaaltoni*, a new genus and species of polychaete worm (Polychaeta, Aphroditidae) from deep water off Oregon, and a revision of the aphroditid genera. Proceedings of the Biological Society of Washington 79: 95–108.

[B308] Pettibone MH (1969) Revision of the aphroditoid polychaetes of the family Eulepethidae Chamberlin (= Eulepidinae Darboux, = Pareulepidae Hartman). Smithsonian Contributions to Zoology 41: 1–44. 10.5479/si.00810282.41

[B309] Pettibone MH (1986a) Additions to the family Eulepethidae Chamberlin (Polychaeta: Aphroditacea). Smithsonian Contributions to Zoology 441: 1–51. 10.5479/si.00810282.441

[B310] Pettibone MH (1986b) Review of the Iphioninae (Polychaeta: Polynoidae) and revision of *Iphionecimex* Quatrefages, *Gattyanadeludens* Fauvel, and *Harmothoe*, *Iphionelloides* Johnson (Harmothoinae). Smithsonian Contributions to Zoology 428: 1–43. 10.5479/si.00810282.428

[B311] Pettibone MH (1989) Revision of the aphroditoid polychaetes of the family Acoetidae Kinberg (= Polyodontidae Augener) and reestablishment of *Acoetes* Audouin and Milne-Edwards, 1832, and *Euarche* Ehlers, 1887. Smithsonian Contributions to Zoology 464. 10.5479/si.00810282.464

[B312] Pettibone M (1992) Contribution to the Polychaete Family Pholoidae Kinberg. Smithsonian Contributions to Zoology 532: i–iii, 24 pp. 10.5479/si.00810282.532

[B313] Phillips AJ Siddall ME (2009) Poly-paraphyly of Hirudinidae: many lineages of medicinal leeches. BMC Evolutionary Biology 9: 246. 10.1186/1471-2148-9-246 PMC2768714 19811660

[B314] Phillips AJ Arauco-Brown R Oceguera-Figueroa A Gomez GP Beltrán M Lai Y-T Siddall ME (2010) *Tyrannobdellarex* N. Gen. N. Sp. and the Evolutionary Origins of Mucosal Leech Infestations. PLOS ONE 5(4): e10057. 10.1371/journal.pone.0010057 PMC2854684 20418947

[B315] Pinder A (2013) Tools for identifying Australian aquatic oligochaetes of the families Phreodrilidae, Lumbriculidae and Capilloventridae (Clitellata: Annelida). Museum Victoria Science Reports 18: 1–19. 10.24199/j.mvsr.2013.18

[B316] Pinder AM (in press) Subclass Oligochaeta. In: Rogers DC, Thorp JH (Eds) Keys to Australasian Fauna: Thorp and Covich’s Freshwater invertebrates – Volume VI. Academic Press/Elsevier, San Diego.

[B317] Pinder AM Brinkhurst RO (1997) The family Capilloventridae (Annelida, Clitellata) in Australia, with descriptions of two new species of *Capilloventer*. Zoologica Scripta 26: 255–265. 10.1111/j.1463-6409.1997.tb00415.x

[B318] Pinder AM Brinkhurst RO (1998) First records of *Slavina* (Oligochaeta: Naididae) in Australia and description of a new species. Proceedings of the Royal Society of Victoria 109: 149–155.

[B319] Pinder AM Erséus C (2000) New Phreodrilidae (Annelida: Clitellata) from Tasmanian estuaries. Papers and Proceedings of the Royal Society of Tasmania 134: 29–33. 10.26749/rstpp.134.29

[B320] Pinder AM Hill BD Green PE (1998) A new species of Dero (Allodero) (Naididae: Oligochaeta) inhabiting the Wolffian Ducts of the Green Tree Frog (*Litoriacaerulea*) in Queensland. Memoirs of the Queensland Museum 42: 559–564.

[B321] Piotrowski C (2014) A new scaleworm, *Iphionemalifera* (Polychaeta: Iphionidae), from a coral reef in the Philippine Islands. In: Williams GC Gosliner M (Eds) The Coral Triangle: The 2011 Hearst Philippine Biodiversity Expedition.California Academy of Sciences, San Francisco, 155–164.

[B322] Plathong J Dean HK Plathong S (2021a) Four new species of Pilargidae (Annelida: Pilarginae) from the Gulf of Thailand. Zootaxa 5071(4): 537–562. 10.11646/zootaxa.5071.4.4 35390895

[B323] Plathong J Plathong S Salazar-Vallejo SI (2021b) Three new species of Sternaspidae (Annelida: Sedentaria) from Thailand. Zootaxa 5081: 373–388. 10.11646/zootaxa.5081.3.4 35391003

[B324] Pleijel F Dahlgren TG (1998) Position and delineation of Chrysopetalidae and Hesionidae (Annelida, Polychaeta, Phyllodocida). Cladistics 14: 129–150. 10.1111/j.1096-0031.1998.tb00327.x 34902925

[B325] Pleijel F Dales RP (1991) British Phyllodocoideans, Typhloscolecoideans and Tomopteroideans. Synopses of the British Fauna (New Series) 45: 1–202.

[B326] Plisko JD (1997) New species of the genus *Tritogenia* Kinberg, 1867 from southern Africa (Oligochaeta: Microchaetidae). Annals of the Natal Museum 38: 241–281. https://hdl.handle.net/10520/AJA03040798_177

[B327] Plisko JD (2012) Notes on the status of the family Microchaetidae. Zoology in the Middle East 58(suppl. 4): 47–58. 10.1080/09397140.2012.10648984

[B328] Plisko JD (2013) A new family Tritogeniidae for the genera *Tritogenia* and *Michalakus*, earlier accredited to the composite Microchaetidae (Annelida: Oligochaeta). African Invertebrates 54: 69–92. 10.5733/afin.054.0107

[B329] Plisko JD Nxele TC (2015) An annotated key separating foreign earthworm species from the indigenous South Africian taxa (Oligochaeta: Acanthodrilidae, Eudrilidae, Glossoscolecidae, Lumbricidae, Megascolecidae, Microchaetidae, Ocnerodrilidae and Tritogeniidae). African Invertebrates 56: 663–708. 10.5733/afin.056.0312

[B330] Pop V (1975) Was ist *Hystricosomachappuisi* Michaelsen (Aeolosomatidae, Oligochaeta)? Mitteilungen aus den Zoologischen Staatsinstitut und Zoologischen Museum in Hamburg 72: 75–78.

[B331] Purschke G (2019a) Hrabeiellidae Christoffersen, 2012. In: Purschke G Böggemann M Westheide W (Eds) Handbook of Zoology, Annelida, Volume 2: Pleistoannelida, Sedentaria II.De Gruyter, Berlin, 275–284. 10.1515/9783110291681-007

[B332] Purschke G (2019b) 7.1.3 Parergodrilidae Reisinger, 1925. In: Purschke G Böggemann M Westheide W (Eds) Handbook of Zoology, Annelida, Volume 1: Annelida Basal Groups and Pleistoannelida, Sedentaria I.De Gruyter, Berlin, 237–250. 10.1515/9783110291582

[B333] Radashevsky VI (2012) Spionidae (Annelida) from shallow waters around the British Islands: an identification guide for the NMBAQC Scheme with an overview of spionid morphology and biology. Zootaxa 3152: 1–35. 10.11646/zootaxa.3152.1.1

[B334] Radashevsky VI (2015) Spionidae (Annelida) from Lizard Island, Great Barrier Reef, Australia: the genera *Aonides*, *Dipolydora*, *Polydorella*, *Prionospio*, *Pseudopolydora*, *Rhynchospio*, and *Tripolydora*. Zootaxa 4019: 635–694. 10.11646/zootaxa.4019.1.22 26624082

[B335] Radashevsky VI (2021) *Pseudopolydora* (Annelida: Spionidae) from European and adjacent waters with a key to identification and description of a new species. Marine Biodiversity 51: 31. 10.1007/s12526-020-01156-7

[B336] Radashevsky VI Rizzo AE Peixoto AJM (2018) First record of *Trochochaetajaponica* (Annelida: Spionidae) in Brazil with identification key to species of the genus. Zootaxa 4462: 29. 10.11646/zootaxa.4462.4.8 30313460

[B337] Ramey-Balci PA Fiege D Purschke G (2021) 7.11.1 Polygordiidae Czerniavsky, 1881. In: Purschke G Böggemann M Westheide W (Eds) Handbook of Zoology, Annelida, Volume 3: Pleistoannelida, Sedentaria III and Errantia I.De Gruyter, Berlin, 266–279. 10.1515/9783110291704-013

[B338] Rashni B Brown KT McLenachan PA Lockhart PJ Southgate PC Lal MM (2023) Leech breach: a first record of the invasive freshwater leech *Helobdellaeuropaea* (Hirudinea: Glossiphoniidae) in Fiji. Pacific Conservation Biology 30(1): 1–13. 10.1071/PC23017

[B339] Ravara A Wiklund H Cunha MR Pleijel F (2010) Phylogenetic relationships within Nephtyidae (Polychaeta, Annelida). Zoologica Scripta 39: 394–405. 10.1111/j.1463-6409.2010.00424.x

[B340] Razafindrakoto M Csuzdi C James S Blanchart E (2017) New earthworms from Madagascar with key to the *Kynotus* species (Oligochaeta: Kynotidae). Zoologischer Anzeiger 268: 126–135. 10.1016/j.jcz.2016.08.001

[B341] Read GB (2000) Taxonomy and distribution of a new *Cossura* species (Annelida: Polychaeta: Cossuridae) from New Zealand. Proceedings of the Biological Society of Washington 113: 1096–1110.

[B342] Reuscher M Fiege D (2011) Sphaerodoridae (Annelida: Polychaeta) from the deep south-west Pacific, with the description of a new species of *Sphaerodoropsis*. Journal of the Marine Biological Association of the United Kingdom 91: 439–445. 10.1017/S0025315410000469

[B343] Reuscher M Fiege D Wehe T (2009) Four new species of Ampharetidae (Annelida: Polychaeta) from Pacific hot vents and cold seeps, with a key and synoptic table of characters for all genera. Zootaxa 2191: 1–40. 10.11646/zootaxa.2191.1.1

[B344] Ringuelet RA (1972a) Cylicobdellidae, nueva familia de hirudineos erpobdelloideos. Physis 31(83): 337–344.

[B345] Ringuelet RA (1972b) Nuevos taxia de Hirudineos neotropicos con la redefinicion de Semiscolecidae y la descripcion de Cyclobdellidae fam. nov. y Mesobdellidae fam. nov. Physis 31(82): 193–201.

[B346] Ringuelet RA (1985) Sinopsis de los hirudineos de Chile (Annelida). Boletin de la Sociedad de Biología de Concepción 56: 163–179.

[B347] Rizzo AE Magalhães WF (2022a) 7.13.6 Lacydoniidae Bergström, 1914. In: Purschke G Böggemann M Westheide W (Eds) Handbook of Zoology.Annelida. Vol. 4: Pleistoannelida, Errantia II. De Gruyter, Berlin, 368–375. 10.1515/9783110647167-015

[B348] Rizzo AE Magalhães WF (2022b) 7.13.7Paralacydoniidae Pettibone, 1963. In: Purschke G Böggemann M Westheide W (Eds) Handbook of Zoology.Annelida. Vol. 4: Pleistoannelida, Errantia II. De Gruyter, Berlin, 376–380. 10.1515/9783110647167-016

[B349] Rizzo AE Salazar-Vallejo SI (2014) Hesionidae Grube, 1850 (Annelida: Polychaeta) from South-Southeastern Brazil, with descriptions of four new species. Zootaxa 3856: 267–291. 10.11646/zootaxa.3856.2.7 25284658

[B350] Rizzo AE Steiner TM Amaral ACZ (2007) Glyceridae Grube 1850 (Annelida: Polychaeta) from Southern and Southeastern Brazil, including a new species of *Glycera*. Biota Neotropical 7(3): 41–59. 10.1590/S1676-06032007000300005

[B351] Rizzo AE Magalhães WF Santos CSG (2015) Lacydoniidae Bergström, 1914 (Polychaeta) in the South Atlantic: morphology, three new species and five new records. Journal of the Marine Biological Association of the United Kingdom 96(6): 1265–1285. 10.1017/S0025315415001381

[B352] Rizzo AE Almeida dos Santos S da Conceição Guerreiro Couto E (2020) First report of *Labrorostratuscaribensis* (Annelida, Oenonidae) as endoparasite of *Haplosyllisrosenalessoae* (Annelida, Syllidae) from Brazil. International Journal of Parasitology: Parasites Wildlife 12: 64–66. 10.1016/j.ijppaw.2020.04.007 PMC7315102 32612924

[B353] Rodriguez P Fend SV (2022) New Nearctic *Eremidrilus* species (Clitellata: Lumbriculidae). Part 2, western species with one spermathecal segment. Zootaxa 5159: 245–264. 10.11646/zootaxa.5159.2.4 36095549

[B354] Rota E Lupetti P (2015) An ultrastructural investigation of *Hrabeiella* Pizl & Chalupský, 1984 (Annelida). II. The spermatozoon. Tissue and Cell 29(5): 603–609. 10.1016/S0040-8166(97)80061-4 18627831

[B355] Rouse GW (2001) A cladistic analysis of Siboglinidae Caullery, 1914 (Polychaeta, Annelida): formerly the phyla Pogonophora and Vestimentifera. Zoological Journal of the Linnean Society 132: 55–80. 10.1111/j.1096-3642.2001.tb02271.x

[B356] Rouse GW Fauchald K (1997) Cladistics and polychaetes. Zoological Scripta 26: 139–204. 10.1111/j.1463-6409.1997.tb00412.x

[B357] Rouse GW Pleijel F (2001) Polychaetes. Oxford: Oxford University Press, 354 pp.

[B358] Rouse GW Goffredi SK Vrijenhoek RC (2004) *Osedax*: bone-eating marine worms with dwarf males. Science 305(5684): 668–671. 10.1126/science.1098650 15286372

[B359] Rouse GW Pleijel F Tilic E (2022) Annelida. Oxford University Press, Oxford, UK, 432 pp. 10.1093/oso/9780199692309.001.0001

[B360] Rzhavsky AV Kupriyanova EK Sikorski AV Dahle S (2014) Calcareous tubeworms (Polychaeta, Serpulidae) of the Arctic Ocean. KMK Scientific Press, Moscow, 200 pp.

[B361] Saiz Salinas JI (2018) Almost five centuries of systematic study of the enigmatic sipunculan worms. In: Proceedings of the Second International Symposium on the Biology of the Sipuncula. Smithsonian Contribution to the Marine Sciences No. 42, Smithsonian Institution Scholarly Press, Washington, DC, USA, 219–235.

[B362] Saiz Salinas JI Dean HK Cutler EB (2000) Echiura from Antarctic and adjacent waters. Polar Biology 23: 661–670. 10.1007/s003000000135

[B363] Saiz Salinas JI Bustamante M Tajadura J (2016) A census of deep-water sipunculans (Sipuncula). Marine Biodiversity 1: 449–464. 10.1007/s12526-016-0568-0

[B364] Salazar-Vallejo SI (1986) Pilargidae (Annelida: Polychaeta) de Mexico: lista de especies, nueva especie y biografia. Cahiers Biologie Marine 27: 193–209. http://www.sb-roscoff.fr/cbm/article.htm?id=8673

[B365] Salazar-Vallejo SI (2009) Acrocirridae Banse, 1969. In: de Léon-Gonzales JA, Bastida-Zavala JR, Carrera-Parra LF, García-Garza ME, Peña-Rivera A, Salazar-Vallejo SI, Solís-Weiss V (Eds) Poliquetos (Annelida: Polychaeta) de México y América Tropical, Universidad Autónoma de Nuevo León, Monterrey, México, 35–39.

[B366] Salazar-Vallejo SI (2017a) Revision of *Brada* Stimpson, 1853, and *Bradabyssa* Hartman, 1967 (Annelida, Flabelligeridae). Zootaxa 4343: 1–98. 10.11646/zootaxa.4343.1.1 29245657

[B367] Salazar-Vallejo SI (2017b) Six new tropical sternaspid species (Annelida, Sternaspidae) with keys to identify genera and species Zoological Studies 56(32): 1–16. 10.6620/ZS.2017.56-32 PMC6517758 31966231

[B368] Salazar-Vallejo SI (2019) 7.3.1.6 Flabelligeridae Saint-Joseph, 1894 1. In: Purschke G Böggemann M Westheide W (Eds) Handbook of Zoology, Annelida, Volume 1: Annelida Basal Groups and Pleistoannelida, Sedentaria I.De Gruyter, Berlin, 398–421.

[B369] Salazar-Vallejo SI (2022) New species of hesionid and phyllodocid polychaetes (Annelida, Errantia) from Clipperton Island. Zoosystema 2022: 1–26. 10.5252/zoosystema2022v44a1

[B370] Salazar‐Vallejo SI Harris LH (2006) Revision of *Pilargis* de Saint‐Joseph, 1899 (Annelida, Polychaeta, Pilargidae). Journal of Natural History 40(3–4): 119–159. 10.1080/00222930600594212

[B371] Salazar-Vallejo SI Gillet P Carrera-Parra LF (2007) Revision of *Chauvinelia*, redescriptions of *Flabellisetaincrusta*, and *Helmetophorusrankini*, and their recognition as acrocirrids (Polychaeta: Acrocirridae). Journal of the Marine Biological Association of the United Kingdom 87: 465–474. 10.1017/S0025315407054501

[B372] Salazar-Vallejo SI Rizzo AE Fukuda MV (2014) Reinstatement of *Euarcherudipalpa* (Polychaeta: Acoetidae), with remarks on morphology and body pigmentation. Taxonomy and Nomenclature. Zoologia (Curitiba) 31(3): 264–270. 10.1590/S1984-46702014000300008

[B373] Salazar-Vallejo SI de León-González JA Carrera-Parra LF (2019a) Phylogeny of Microphthalminae Hartmann-Schröder, 1971, and revision of *Hesionella* Hartman, 1939, and *Struwela* Hartmann-Schröder, 1959 (Annelida, Errantia). PeerJ 7: 1–35. 10.7717/peerj.7723 PMC6754975 31579604

[B374] Salazar-Vallejo SI Rizzo AE de León-González JÁ Brauko KM (2019b) Four new Caribbean *Sigambra* species (Annelida, Pilargidae), and clarifications of three other *Sigambra* species. ZooKeys 893: 21–50. 10.3897/zookeys.893.39594 31844399 PMC6901610

[B375] Salazar-Vallejo SI Zhadan AE Rizzo AE (2019c) Revision of Fauveliopsidae Hartman, 1971 (Annelida, Sedentaria). Zootaxa 4637: 1–67. 10.11646/zootaxa.4637.1.1 31712490

[B376] Salcedo ODL Hernández-Alcántara P Solís-Weiss V (2015) Description of two new species of *Pisione* (Polychaeta: Sigalionidae) and first record of *Pisionegalapagoensis* Westheide in the Southern Mexican Pacific. Zootaxa 4039: 373–390. 10.11646/zootaxa.4039.2.10 26624485

[B377] San Martín G (2003) Familia Syllidae, Annelida, Polychaeta II. In: Fauna Ibérica, Vol. 21, MA Ramos et al. (Eds). Museo Nacional de Ciencias Naturales, CSIC, Madrid, 554 pp.

[B378] San Martín G (2004) Familia Chrysopetalidae Ehlers, 1864. In: Viéitez JM, Alós C, Parapar J, Besteiro C, Moreira J, Núñez J, Laborda J, San Martín G (Eds) Fauna Ibérica 25: 105–209. Museo Nacional de Ciencias Naturales, CSIC, Madrid.

[B379] San Martín G (2005) Exogoninae (Polychaeta: Syllidae) from Australia with the description of a new genus and twenty-two new species. Records of the Australian Museum 57: 39–152. 10.3853/j.0067-1975.57.2005.1438

[B380] San Martín G Aguado MT (2022) 7.13.2. Syllidae Grube, 1850. In: Purschke G Böggemann M Westheide W (Eds) Handbook of Zoology, Annelida, Volume 3: Pleistoannelida, Errantia II.De Gruyter, Berlin, 152–227. 10.1515/9783110647167-008

[B381] San Martín G Hutchings P (2006) Eusyllinae (Polychaeta: Syllidae) from Australia with the description of a new genus and fifteen new species. Records of the Australian Museum 58: 257–370. 10.3853/j.0067-1975.58.2006.1466

[B382] San Martín G Worsfold TM (2015) Guide and keys for the identification of Syllidae (Annelida, Phyllodocida) from the British Isles (reported and expected species). ZooKeys 488: 1–29. 10.3897/zookeys.488.9061 PMC4389122 25878521

[B383] San Martín G Hutchings P Aguado MT (2008) Syllinae (Polychaeta: Syllidae) from Australia. Part 1. Genera *Branchiosyllis*, *Eurysyllis*, *Karroonsyllis*, *Parasphaerosyllis*, *Plakosyllis*, *Rhopalosyllis*, *Tetrapalpia* n.gen., and *Xenosyllis*. Records of the Australian Museum 60: 119–160. 10.3853/j.0067-1975.60.2008.1494

[B384] San Martín G Álvarez-Campos P Aguado MT (2013) The genus *Branchiosyllis* Ehlers, 1887 (Annelida, Syllidae, Syllinae) from off the American coasts, with the description of a new species from Venezuela. Pan-American Journal of Aquatic Sciences 8: 166–179.

[B385] Sawyer RT (1986) Leech biology and behaviour. Vol. 1 Anatomy, Physiology, and Behaviour. Oxford Science Publications, Oxford UK, 418 pp.

[B386] Sawyer RT Lawler AR Overstreet RM (1975) Marine leeches of the eastern United States and the Gulf of Mexico with a key to the species. Journal of Natural History 9: 633–667. 10.1080/00222937500770531

[B387] Schmelz R Collado R (2010) A guide to European terrestrial and freshwater species of Enchytraeidae (Oligochaeta). Soil Organisms 82: 1–176.

[B388] Schmelz RM Erséus C Martin P van Haaren T Timm T (2021) A proposed order-level classification in Oligochaeta (Annelida, Clitellata). Zootaxa 5040(4): 589–597. 10.11646/zootaxa.5040.4.9 34811021

[B389] Schüller M Hutchings PA (2013) New species of *Terebellides* (Polychaeta: Trichobranchidae) from the deep Southern Ocean, with a key to all described species. Zootaxa 3619: 1–45. [PMID: 26131462] 10.11646/zootaxa.3619.1.1 26131462

[B390] Schulze A Boyle MJ Kawauchi GY (2019) 6.1. Sipuncula. In: Purschke G Böggemann M Westheide W (Eds) Handbook of Zoology, Annelida, Volume 1: Annelida Basal Groups and Pleistoannelida, Sedentaria I.De Gruyter, Berlin, 177–201. 10.1515/9783110291582-006

[B391] Sedick S Clarke D Biccard A Gihwala KN (2023) A new species of *Ninoe* (Annelida: Lumbrineridae) from the continental shelf off southern Namibia. Journal of the Marine Biological Association of the United Kingdom 103: e25. 10.1017/S0025315423000152

[B392] Seesamut T Oba Y Jirapatrasilp P Martinsson S Lindström M Erséus C Panha S (2024) Global species delimitation of the cosmopolitan marine littoral earthworm *Pontodriluslitoralis* (Grube, 1855). Scientific Reports 14: 1753. 10.1038/s41598-024-52252-8 PMC10799051 38243053

[B393] Sendall K Salazar-Vallejo SI (2013) Revision of *Sternaspis* Otto, 1821 (Polychaeta, Sternaspidae). ZooKeys 74: 1–74. 10.3897/zookeys.286.4438 PMC3677357 23794844

[B394] Siddall ME (1995) Phylogeny of the Euhirudinea: Independent evolution of blood feeding by leeches? Canadian Journal of Zoology 73(6): 1048–1064. 10.1139/z95-125

[B395] Siddall ME (2002) Phylogeny of the leech family Erpobdellidae (Hirudinea: Oligochaeta). Invertebrate Systematics 16: 1–16. 10.1071/IT01011

[B396] Siddall ME Borda E (2004) Leech collections from Chile including two new species of *Helobdella* (Annelida: Hirudinida). American Museum Novitates 3457: 1–18. 10.1206/0003-0082(2004)457<0001:LCFCIT>2.0.CO;2

[B397] Siddall ME Bely AE Borda E (2006) Hirudinida. In: Rouse G Pleijel F (Eds) Reproductive Biology and Phylogeny of Annelida.Series Editor BGM Jamieson. Volume 4. Science Publishers, Enfield, New Hampshire, 393–429.

[B398] Sikorski AV Pavlova LV (2015) New species of *Scolelepis* (Polychaeta, Spionidae) from the Norwegian coast and Barents Sea with a brief review of the genus. Fauna Norvegica 35: 9–19. 10.5324/fn.v35i0.1666

[B399] Silva CF Amaral ACZ (2019) *Scyphoproctus* Gravier, 1904 (Annelida, Capitellidae): description of three new species and relocation of *Heteromastides* Augener, 1914 in *Scyphoproctus*. Zootaxa 4560(1): 95–120. 10.11646/zootaxa.4560.1.5 30790993

[B400] Silva CF Seixas VC Barroso R Di Domenico M Amaral ACZ Paiva PC (2017) Demystifying the *Capitellacapitata* complex (Annelida, Capitellidae) diversity by morphological and molecular data along the Brazilian coast. PLOS ONE 12(5): e0177760. 10.1371/journal.pone.0177760 PMC5451021 28562616

[B401] Sims RW (1980) A classification and the distribution of earthworms, suborder Lumbricina (Haplotaxida: Oligochaeta). Bulletin of the British Museum (Natural History) Zoology 39: 103–124.

[B402] Sims RW (1987) A review of the Central African earthworm family Eudrilidae (Oligochaeta). In: Bonvicini Pagliai AM Omodeo P (Eds) On earthworms.Selected symposia and monographs, UZI, 2. Mucchi, Modena, 359–388.

[B403] Sket B Trontelj P (2008) Global diversity of leeches (Hirudinea) in freshwater. Hydrobiologia 595: 129–137. 10.1007/s10750-007-9010-8

[B404] Soos A (1967) Identification key to the leech (Hirudinoidea) genera of the world, with a catalogue of the species: IV. Haemadipsidae. Acta Zoologica Academiae Scientiarum Hungaricae 13: 417–432.

[B405] Sousa LKS Nogueira JMM Cutrim MVJ Oliveira VM (2019) *Cossurayacy* sp. nov. (Cossuridae, Annelida) from a tropical Brazilian estuary. Iheringia, Série Zoologia 109: e2019021. 10.1590/1678-4766e2019021

[B406] Southward E (1999) Class Pogonophora. In: Beesley PL Ross GJB Glasby CJ (Eds) [2000] Polychaetes & Allies: The Southern Synthesis.Fauna of Australia Vol 4A. CSIRO Publishing, Melbourne, 331–351. http://www.environment.gov.au/science/abrs/publications/fauna-of-australia/fauna-4a

[B407] Stephen AC Edmonds SJ (1972) The Phyla Sipuncula and Echiura. Trustees of the British Museum (Natural History), London, 528 pp.

[B408] Stiller J Tilic E Rousset V Pleijel F Rouse GW (2020) Spaghetti to a tree: A robust phylogeny for Terebelliformia (Annelida) based on transcriptomes, molecular and morphological data. Biology 9(4): 73. [29 pp. + supplementary material] 10.3390/biology9040073 32268525 PMC7236012

[B409] Strelzov VE (1979) Polychaete Worms of the Family Paraonidae Cerruti, 1909 (Polychaeta, Sedentaria). New Delhi, Amerind Publishing Co. Pvt Ltd, 212 pp.

[B410] Struck TH (2019) 2. Phylogeny. In: Purschke G Böggemann M Westheide W (Eds) Handbook of Zoology, Annelida Vol.1, Annelida basal groups and Pleistoannelida, Sedentaria, I. De Gruyter, Berlin, 37–68. 10.1515/9783110291582-002

[B411] Struck TH Koczula J Stateczny D (2017) Two new species in the annelid genus *Stygocapitella* (Orbiniida, Parergodrilidae) with comments on their biogeography. Zootaxa 4286(3): 301–332. 10.11646/zootaxa.4286.3.1

[B412] Sun Y Li X (2017) A new genus and species of bristle worm from Beibu Gulf, South China Sea (Annelida, Polychaeta, Amphinomidae). ZooKeys 708: 1–10. 10.3897/zookeys.708.12967 PMC5674149 29118631

[B413] Sun Y Qiu J-W (2014) A new species of *Chaetopterus* (Annelida, Chaetopteridae) from Hong Kong. Memoirs of Museum Victoria 71: 303–309. 10.24199/j.mmv.2014.71.23

[B414] Sun Y Hove HA ten Qiu J-W (2012) Serpulidae (Annelida: Polychaeta) from Hong Kong. Zootaxa 3424: 1–42. 10.11646/zootaxa.3424.1.1

[B415] Sun Y Sui J Li X (2018) A new species of *Leodamas* Kinberg, 1866 (Polychaeta: Orbiniidae) from the Yellow Sea and the East China Sea. Acta Oceanologica Sinica 37: 130–135. 10.1007/s13131-018-1313-2

[B416] Taylor AG (1949) A West African Earthworm, *Hippoperanigeriae*, belonging to a new Family Hippoperidae. Journal of Zoology 119: 703–710. 10.1111/j.1096-3642.1949.tb00898.x

[B417] Tessler M De Carle D Voiklis ML Gresham OA Neumann JS Cios S Siddall ME (2018) Worms that suck: phylogenetic analysis of Hirudinea solidifies the position of Acanthobdellida and necessitates the dissolution of Rhynchobdellida. Molecular Phylogenetics and Evolution 127: 129–134. 10.1016/j.ympev.2018.05.001 29778721

[B418] Thorp JH Lovell LL Timm T Martin P Gelder SR Govedich FR Moser WE Nakano T Bielecki A Bain BA Utevsky S Gil J Glasby CJ Martin D (2019) Phylum Annelida. In: Rogers DC Thorp JH (Eds) Keys to Palaearctic Fauna: Thorp and Covich’s Freshwater invertebrates – Volume IV.Academic Press/ Elsevier, San Diego, 357–518. [ISBN: 9780123850249] 10.1016/B978-0-12-385024-9.00012-5

[B419] Tilic E Sayyari E Stiller J Mirarab S Rouse GW (2020) More is needed—thousands of loci are required to elucidate the relationships of the ‘Flowers of the Sea’ (Sabellida, Annelida). Molecular Phylogenetics and Evolution 151: 106892. 10.1016/j.ympev.2020.106892 32562819

[B420] Tilic E Stiller J Campos E Pleijel F Rouse GW (2022) Phylogenomics resolves ambiguous relationships within Aciculata (Errantia, Annelida). Molecular Phylogenetics and Evolution 166: 107339. 10.1016/j.ympev.2021.107339 34751138

[B421] Timm T (2009) A guide to the freshwater Oligochaeta and Polychaeta of Northern and Central Europe. Lauterbornia 66: 1–235.

[B422] Timm T (2012) Life forms in Oligochaeta: a literature review. Zoology in the Middle East 58(sup. 4): 71–82. 10.1080/09397140.2012.10648986

[B423] Tovar-Hernández MA Salazar-Vallejo SI (2006) Sabellids (Polychaeta: Sabellidae) from the Grand Caribbean. Zoological Studies 45: 24–66.

[B424] Uttam S Langer S (2021) Distribution and Identification key for species of freshwater leech genus *Erpobdella* Blainville, 1818 (Hirudinida: Arhynchobdellida: Erpobdelliformes: Erpobdellidae). Ecology, Environment and Conservation 27: 1925–1936.

[B425] Viéitez JM Alós C Parapar J Besteiro C Moreira J Núñez J Laborda J San Martín G (2004) AnnelidaPolychaeta I. Fauna Ibérica, Vol. 25. In: Ramos MA et al. (Eds) Museo Nacional de Ciencias Naturales, CSIC, Madrid, 530 pp.

[B426] Wang Y Li X (2017) A new species of *Phyllochaetopterus* Grube, 1863 (Polychaeta: Chaetopteridae) from Hainan Island, South China Sea. Chinese Journal of Oceanology and Limnology 35: 360–366. 10.1007/s00343-016-5256-1

[B427] Warren LM Hutchings PA Doyle S (1994) A revision of the genus *Mediomastus* Hartman, 1944 (Polychaeta: Capitellidae). Records of the Australian Museum 46(3): 227–256. 10.3853/j.0067-1975.46.1994.6

[B428] Watson C (2022) 7.13.3.1 Chrysopetalidae Ehlers, 1864. In: Purschke G Böggemann M Westheide W (Eds) Handbook of Zoology.Annelida. Vol. 4: Pleistoannelida, Errantia II. De Gruyter, Berlin, 228–258. 10.1515/9783110647167-009

[B429] Wehe T (2006) Revision of the scale worms (Polychaeta: Aphroditoidea) occurring in the seas surrounding the Arabian Peninsula. Part I: Polynoidae. Fauna of Arabia 22: 23–197.

[B430] Weigert A Helm C Meyer M Nickel B Arendt D Hausdorf B Santos SR Halanych KM Purschke G Bleidorn C Struck TH (2014) Illuminating the Base of the Annelid Tree Using Transcriptomics. Molecular Biology and Evolution 31(6): 1391–1401. 10.1093/molbev/msu080 24567512

[B431] Wells GP (1959) The genera of Arenicolidae (Polychaeta). Proceedings of the Zoological Society of London 133: 301–314. 10.1111/j.1469-7998.1959.tb05564.x

[B432] Westheide W (2013) *Microphthalmusmahensis* sp.n. (Annelida, Phyllodocida) together with an annotated key of the genus. Helgoland Marine Research 67: 413–422. 10.1007/s10152-012-0332-1

[B433] Westheide W (2019) 7.1.1 Dinophilidae Verrill, 1892. In: Purschke G Böggemann M Westheide W (Eds) Handbook of Zoology, Annelida, Volume 1: Annelida Basal Groups and Pleistoannelida, Sedentaria I.De Gruyter, Berlin, 217–233. 10.1515/9783110291582-007

[B434] Wiklund H Nygren A Pleijel F Sundberg P (2005) Phylogeny of Aphroditiformia (Polychaeta) based on molecular and morphological data. Molecular Phylogenetics and Evolution 37: 494–502. 10.1016/j.ympev.2005.07.005 16112882

[B435] Wiklund H Purschke G Ravara A (2021) 7.12.2 Dorvilleidae Chamberlin, 1919. In: Purschke G Böggemann M Westheide W (Eds) Handbook of Zoology, Annelida, Volume 3: Pleistoannelida, Sedentaria III and Errantia I.De Gruyter, Berlin, 361–382. 10.1515/9783110291704-018

[B436] Wilson R (2000) Family Longosomatidae. In: Beesley PL, Ross GJB, Glasby CJ (Eds) Fauna of Australia. Volume 4A, Polychaetes & Allies: the Southern Synthesis. CSIRO Publishing, Melbourne, 193 pp.

[B437] Wilson RS Avery L (2023) *Travisia* (Annelida: Travisiidae) – A DELTA Intkey interactive key to species. 10.5281/ZENODO.8127410

[B438] Wilson RS Glasby CJ Bakken T (2023) The Nereididae (Annelida) – diagnoses, descriptions, and a key to the genera. ZooKeys 1182: 35–134. 10.3897/zookeys.1182.104258 37868122 PMC10585372

[B439] WoRMS (2025) Annelida. https://www.marinespecies.org/aphia.php?p=taxdetails&id=882 [Accessed 1 Apr. 2025]

[B440] Worsaae K (2005) Phylogeny of Nerillidae (Polychaeta, Annelida) as inferred from combined 18S rDNA and morphological data. Cladistics 21: 143–162. 10.1111/j.1096-0031.2005.00058.x 34892860

[B441] Worsaae K (2019) 5.2 Psammodrilidae Swedmark, 1952. In: Purschke G Böggemann M Westheide W (Eds) Handbook of Zoology, Annelida, Volume 1: Annelida Basal Groups and Pleistoannelida, Sedentaria I.De Gruyter, Berlin, 143–155.

[B442] Worsaae K (2021) [online version 2014] Errantia incertae sedis: Nerillidae Levinsen, 1883. In: Wesheide W, Purschke G, Böggemann M (Eds) Handbook of Zoology, Annelida, Volume 3 Pleistoannelida, Sedentaria III and Errantia I, Berlin, Boston: De Gruyter. 10.1515/9783110291704-011

[B443] Worsaae K Kerbl A Di Domenico M Gonzalez BC Bekkouche N Martínez A (2021) Interstitial Annelida. Diversity 13(2): 1–58. [Article 77] 10.3390/d13020077

[B444] Worsfold TM (2003) Introduction to Oligochaetes. Report to the NMBAQC 2003 Taxonomic Workshop participants – Dove Marine Laboratory. Unicomarine Report NMBAQC olig03, 22 pp. https://www.nmbaqcs.org/media/joocjvyb/introduction-to-oligochaeta-2006.pdf

[B445] Wu X Sun R Liu R Xu K (2013) Two new species of *Eunice* Cuvier, 1817 (Polychaeta, Eunicidae) from the coral reefs of Hainan Island with a key to 16 species of Eunice from China seas. Zootaxa 3652(2): 249–264. 10.11646/zootaxa.3652.2.3 26269828

[B446] Xu T Sun Y Wang Z Sen A Qian P-Y Qiu J-W (2022) The Morphology, Mitogenome, Phylogenetic Position, and Symbiotic Bacteria of a New Species of *Sclerolinum* (Annelida: Siboglinidae) in the South China Sea. Frontiers of Marine Science 8: 793645. 10.3389/fmars.2021.793645

[B447] Yamamoto R Imajima M (1985) A new species of the genus *Spinther* (Polychaeta, Spintheridae) from Japan. Bulletin of the National Science Museum, Tokyo. Series A (Zoology) 11: 129–135. https://www.kahaku.go.jp/english/research/publication/zoology/v11_3.html

[B448] Zanol J Ruta C (2015) New and previously known species of Oenonidae (Polychaeta: Annelida) from Lizard Island, Great Barrier Reef, Australia. Zootaxa. 4019: 745–772. 10.11646/zootaxa.4019.1.26 26624086

[B449] Zanol J Halanych KM Fauchald K (2013) Reconciling taxonomy and phylogeny in the bristleworm family Eunicidae (polychaete, Annelida). Zoologica Scripta 43(1): 79–100. 10.1111/zsc.12034

[B450] Zhadan AE Salazar-Vallejo SI (2019) 7.3.1.3 Fauveliopsidae Hartman, 1971. In: Purschke G Böggemann M Westheide W (Eds) Handbook of Zoology, Annelida, Volume 1: Annelida Basal Groups and Pleistoannelida, Sedentaria I.De Gruyter, Berlin, 317–327.

[B451] Zhadan AE Tzetlin AB (2009) [text date 2008] Polychaetes from deep pelagic zone of the Mid-Atlantic ridge. Invertebrate Zoology 5(2): 97–109. 10.15298/invertzool.05.2.02

[B452] Zhang J Hutchings P (2018) Taxonomy and distribution of *Terebellides* (Polychaeta: Trichobranchidae), with the description of a new species. Zootaxa 4377: 387–411. 10.11646/zootaxa.4377.3.4 29690048

[B453] Zhang J Hutchings P (2019) A revision of Australian Pectinariidae (Polychaeta), with new species and new records. Zootaxa 4611(1): 1–70. 10.11646/zootaxa.4611.1.1 31717089

[B454] Zhang Y Sun J Rouse GW Wiklund H Pleijel F Watanabe HK Chen C Qian PY Qiu JW (2018) Phylogeny, evolution and mitochondrial gene order rearrangement in scale worms (Aphroditiformia, Annelida) Molecular Phylogenetics and Evolution 125: 220–231. 10.1016/j.ympev.2018.04.002 29625228

[B455] Zhang J Hutchings P Burghardt I Kupriyanova E (2020) Two new species of Sabellariidae (Annelida, Polychaeta) from the abyss of eastern Australia. Zootaxa 4821(3): 487–510. [PMID: 33056312] 10.11646/zootaxa.4821.3.4 33056312

[B456] Zrzavý J Říha P Piálek L Janouškovec J (2009) Phylogeny of Annelida (Lophotrochozoa): total-evidence analysis of morphology and six genes – art. no. 189. BMC Evolutionary Biology 9: 189–189. 10.1186/1471-2148-9-189 19660115 PMC2732625

