# Supplementary material for: ﻿ANNiKEY Linear – diagnoses, descriptions, and a single-access identification key to Annelida family-level taxa
Source: Zookeys. 2025 Jul 31;1247:217–403. doi: 10.3897/zookeys.1247.137606 (PMC12344570; doi:10.3897/zookeys.1247.137606)
Supplement: ﻿Supplementary material 1 — Morphological descriptions of annelid family-level taxa [file zookeys-1247-217_article-137606__-s001.docx]

# Supplementary File S1. Morphological descriptions

**Aberrantidae Wolf, 1987**

**Description**. Body segment number: variable. Body shape elongate, more-or-less equal width over entire length. Epidermis more-or-less smooth (transverse rows of cilia on each segment and more generally on head). Body surface with protective covering absent. Pygidium simple lobe. Pygidial appendages present, one pair of cirri and single medial papilla. Discrete head present; complex in shape bearing appendages.

Prostomium rounded to oval, eyes on head present, one pair (red coloured in live animals); head eyes simple pigmented cups. Prostomial antennae present; median and paired laterals; unarticulated. Palps present; anteroventral; grooved (usually) feeding type; feeding palp longitudinally grooved. Nuchal organs present; paired low projections from posterolateral prostomium; indistinct dorsolateral ciliated patches. Peristomium a double ring (or triannulate; uncertain whether the rings represent a biannulate peristomium plus one achaetous segment or a triannulate peristomium).

Foregut a muscular ventral pharynx.

First segment achaetous (probably); second segment chaetous. First chaetiger with both notochaetae and neurochaetae; parapodia similar in length or slightly shorter than subsequent parapodia; parapodia more-or-less laterally directed and free from head; chaetae similar in orientation, length and thickness to other chaetae. Parapodia biramous; parapodial lobes prominent. Notopodial lobes large, fusiform adorned with sensory hairs. Neuropodial lobes large, fusiform adorned with sensory hairs. Dorsal cirri present; more-or-less cirriform. Ventral cirri present; cirriform or tapering. Branchiae present, arise from lateral body, occur on mid-body segments and occur near posterior end; lateral branchiae digitiform (and ciliated).

Chaetae first appear on second segment after peristomium (assuming one achaetous pre-chaetigerous segment), arranged in paired bundles (or rows) of many chaetae. Aciculae absent. Capillary chaetae present, in dorsal (notopodial) position, distally tapered to a point, edge smooth or hirsute-serrate. Capillary chaetae, externally not pseudo-segmented. Forked chaetae present (in neuropodia), lyrate, tines more-or-less equal in length. Spines present; slightly curved and more-or-less smooth (hook-like tip); present in most or all chaetigers; in ventral (neuropodial) position only. Hooks absent.

Tube absent.

**Literature used for description**. Glasby CJ, Fauchald K (2003). POLiKEY. An information system for polychaete families and higher taxa. Version 2, 5 June 2003. Australian Biological Resources Study, DCCEEW, Canberra, https:[//www.dcceew.gov.au/;](http://www.dcceew.gov.au/%3B) Wolf, PS (1987) Aberrantidae: A new family of Polychaeta (Annelida). Bulletin of the Biological Society of Washington 7: 50–52; Mackie ASY, Pleijel F,Rouse GW (2005) Revision of *Aberranta* (Aberrantidae: Annelida), with descriptions of new species from the Mediterranean and Hong Kong. Marine Ecology 26: 197–208; Mackie, ASY (2019) 7.2.1 Aberrantidae Wolf, 1987 In Purschke G, Böggemann M, Westheide W (Eds), Handbook of Zoology, Annelida, Volume 1: Annelida Basal Groups and Pleistoannelida, Sedentaria I. De Gruyter, Berlin, 269–273; Rouse GW, Pleijel, F, Tilic, E (2022) Annelida. Oxford University Press, Oxford, UK.

# Acanthobdellidae Grube, 1851

**Description**. Body segment number: fixed (segments generally obscured by 4 intervening superficial annulations, giving the impression of many more segments), 31 segments including 2 preoral 'segments' (prostomium and peristomium) and 29 postoral segments. Secondary annulation present (4 superficial annuli per segment). Body shape elongate, more-or-less equal width over entire length (when relaxed; but can be dramatically shortened by contraction of longitudinal muscles during movement). Anterior end sucker on ventral surface absent (vestigial). Posterior sucker on ventral surface present; circular; rays absent; anus positioned near posterior end (dorsally, a few segments before posterior sucker).

Prostomium bluntly conical. Postcephalic eyes present (3 pairs), epidermal; 3 annuli each with a pair of eyes.

Gut more-or-less straight, lacking side branches. Foregut a muscular axial pharynx. Pharynx jaws absent. Protrusible proboscis present. Circulatory system present.

Simple hook-formed chaetae on segments 1-5 only, 8 per segment arranged in one row.

Sexual reproduction hermaphroditic. Clitellum present. Testes present, many pairs. Sperm sac present (paired, extend over several segments). Ovaries present, one pair. Egg sacs present (paired. extend over several segments); tubular. Oviduct separate one for each egg sac. Segmental organs are protonephridia; nephridial pores single (ventromedial), located anteriorly; gonoducts located around clitellum (2 pairs). Spermathecae present, post-testicular. Spermathecal pores present, located located within 1 or 2 segments of male pores; 1 pair in segment XIII. Female gonoduct present. Female pores single, median, in segment XII. Male gonoducts present, with atrium present, bilobed. Male pores single, median, in segment XI.

**Literature used for description**. Mann KH (1962) Leeches (Hirudinea): Their Structure, Physiology, Ecology and Embryology. With an Appendix on the Systematics of Marine Leeches Pergamon Press, New York, 201 pp; Govedich FR, Bain BA, Moser WE, Gelder SR, Davies RW, Brinkhurst RO (2009) Annelida (Clitellata): Oligochaeta, Branchiobdellida Hirudinida, and Acanthobdellida. In: Thorp JH, Covich AP (eds) Ecology and Classification of North American Freshwater Invertebrates, 3rd Edition. Elsevier Inc., 385–436; Brusca, RC, Moore, W, Shuster, SM (eds) (2016). Chapter 14. Phylum Annelida. In: Invertebrates, 3rd Edition. Sinauer Associates, Sunderland, MA; Thorp JH, Lovell LL, Timm T, Martin P, Gelder SR, Govedich FR, Moser WE, Nakano T, Bielecki A, Bain BA, Utevsky S, Gil J, Glasby CJ Martin D (2019) Phylum Annelida. In: Rogers DC, Thorp JH (Eds.) Keys to Palaearctic Fauna: Thorp and Covich’s Freshwater invertebrates - Volume IV. Academic Press, Elsevier, pp. 357–518; De Carle DB, Gajda L, Bielecki A, Cios S, Cichocka JM, Golden HE, Gryska AD, Sokolov S, Shedko MB, Knudsen R, Utevsky S, Śwaitek P, Tessler M. (2023) Recent evolution of ancient Arctic leech relatives: systematics of Acanthobdellida. Zoological Journal of the Linnean Society 196: 149–168.

# Acanthodrilidae Claus, 1880

**Description**. Body segment number: variable. Secondary annulation present; mid-body biannulate, or triannulate. Body shape elongate, more-or-less equal width over entire length. Bioluminescence absent, or present. Dorsal intersegmental furrow present, pores on mid-dorsal line present. Pygidium simple lobe. Pygidial appendages absent.

Prostomium bluntly conical.

Gut straight with side branches. Foregut a muscular dorsal pharynx. Gizzard present (usually single; in segments I-III, rarely absent). Calciferous glands absent (rarely present). Intestinal typhlosole present, formed from the inner (epithelial) layer only of the intestine (?). Heart bodies present, appear from XI.

Chaetae first appear on first segment after peristomium (=S2 for oligochaete workers), in lumbricine arrangement - paired upper and lower bundles of one or a few chaetae each - closely spaced lateral and ventrolateral pairs or arranged in widely spaced lateral and ventrolateral pairs. Crotchet chaetae present, simple-pointed. Genital chaetae present (variety of penial and spermathecal chaetae).

Sexual reproduction hermaphroditic. Clitellum, thick, multilayered, fully encircles body (rarely saddle-shaped), anterior to male pores, in region of female pores. Clitellum occurs from XII to XVII. Seminal groove absent, or present. Tubercula pubertatis present. Gonadal segments bearing genital papillae present (paired or singular). Gonadal segments with extensions of the body wall absent. Testes present, one pair in total or two pairs in total; one pair per segment, present in segments X and XI. Ovaries present, one pair; present in segments XIII. Segmental organs are metanephridia; both nephridial pores and gonoducts located around clitellum; one pair nephridia in each segment (holonephridia) or multiple, minute, nephridia in each segment (meronephridia) or mixed holonephridia and meronephridia. Spermathecae present, with a basal diverticula, pre-testicular. Spermathecal pores present (rarely absent in Octochaetinae), located well anterior to male pores, 0–3 pairs in segment VII-IX, at intersegments; rarely absent. Female gonoduct present. Female pores one pair or two pairs, in segment XIV. Male gonoducts absent or present (inconspicuous or on small protruberances in Octochaetinae). Male pores single, median or one pair, in segment XVIII usually; rarely shifted one or two segments more posteriorly; opisthoporous (two or more segments following testicular segment). Prostate gland absent or present, tubular (usually, rarely tubuloracemose; one to three pairs). Prostate pores in segments XVII to XIX, ventral, may be prominent; prostate pore and male pore on segment XVII/XVIII not united, separate pores.

**Literature used for description**. Dyne GR, Jamieson BGM (2004) Native Earthworms of Australia II (Megascolecidae, Acanthodrilinae). Department of the Environment and Heritage, Australian Government, 200 pp; Blakemore, RJ (2005) Wither Octochaetidae? A review of its family status (Annelida: Oligochaeta) In AA & VV Pop (Eds) Advances in Earthworm Taxonomy II. Proceedings IOTM2, Cluj University Press, Romania, pp 63–84; Jamieson, BGM (2006) Non-leech Clitellata (with contributions by Marco Ferraguti). Pp. 235–392. In Reproductive Biology and Phylogeny of Annelida. Series Editor BGM Jamieson. Volume 4. Editors G. Rouse, F. Pleijel. Science Publishers, Enfield, New Hampshire; James SW, Davidson SK (2012) Molecular phylogeny of earthworms (Annelida : Crassiclitellata) based on 28S, 18S and 16S gene sequences. Invertebrate Systematics 26: 213–229; Plisko JD, Nxele, TC (2015) An Annotated Key Separating Foreign Earthworm Species from the Indigenous South African taxa (Oligochaeta: Acanthodrilidae, Eudrilidae, Glossoscolecidae, Lumbricidae, Megascolecidae, Microchaetidae, Ocnerodrilidae and Tritogeniidae). African Invertebrates 56:663–708; Rota E, de Jong Y (2015) Fauna Europaea: Annelida - Terrestrial Oligochaeta (Enchytraeidae and Megadrili), Aphanoneura and Polychaeta. Biodiversity Data Journal 3: e5737. doi: 10.3897/BDJ.3.e5737; Thorp JH et al. 2019. Phylum Annelida, In Keys to Palaearctic Fauna: Thorp and Covich's Freshwater Invertebrates, Volume IV. Academic Press, Elsevier; Misirlioðlu, M, Reynolds, JW, Stojanoviæ, M, Trakiæ, T, Sekuliæ, J, James, SW, Csuzdi, C, Decaëns, T, Lapied, E, Phillips, HRP, Cameron, EK, Brown, GG (2023). Earthworms (Clitellata, Megadrili) of the world: an updated checklist of valid species and families, with notes on their distribution. Zootaxa 5255 (1): 417–438.

# Acoetidae Kinberg, 1856

**Description**. Body segment number: variable. Body shape elongate, more-or-less equal width over entire length, dorsoventrally flattened. Epidermis more-or-less smooth. Body surface with protective covering as scales (elytrae), dorsally (silky fibres produced by the worm may also cover the dorsal surface). Pygidium simple lobe. Pygidial appendages present, one pair of cirri. Discrete head present; complex in shape bearing appendages.

Prostomium pentagonal to quadrangular, anteriorly incised, ommatophores present, ommatophores as stalk-like ocular peduncles, facial tubercle present, eyes on head present (anterior pair stalked, posterior pair sessile), two pairs; head eyes compound, with lenses. Prostomial antennae present; median and paired laterals; unarticulated. Palps present; anteroventral; tapering (usually) sensory type; unarticulated. Nuchal organs present; paired low projections from posterolateral prostomium; indistinct dorsolateral ciliated patches. Peristomium not visible.

Gut straight with side branches (as caeca in the midgut). Foregut a muscular axial pharynx. Pharynx jaws present, two pairs of jaws. Paired jaws fang-like. Protrusible proboscis present, distal ring of papillae present.

First segment chaetous and tentaculate (dorsal and ventral cirri enlarged and elongated); second segment chaetous. Tentacular cirri present (modified dorsal and ventral cirri); 2 pair(s); arise on a single segment. First chaetiger with neurochaetae only; parapodia similar in length or slightly shorter than subsequent parapodia; parapodia anteriorly directed and wrapping around head; chaetae similar in orientation, length and thickness to other chaetae. Parapodia biramous; parapodial lobes prominent. Notopodial lobes represented by at least one chaetal lobe. Neuropodial lobes represented by at least one chaetal lobe. Dorsal cirri present (present on non-elytrous segments); more-or-less cirriform. Elytra with papillae, tubercles or smooth, or with lateral pouches (paired elytra mainly restricted to dorsolateral body, only meeting mid-dorsum near head and tail). Elytra, post segment 7 occur on every other segment from segment 7 to end of body. Ventral cirri present; cirriform or tapering. Branchiae absent or present, arise from lateral body, occur on mid-body segments; lateral branchiae digitiform, or branching (pinnate).

Chaetae first appear on first segment after peristomium or second segment after peristomium, arranged in paired bundles (or rows) of many chaetae. Aciculae present; in both dorsal and ventral positions (=noto- and neuroaciculae). Neuroaciculae distally tapering. Capillary chaetae present, in dorsal (notopodial) position or in ventral (neuropodial) position, distally tapered to a point, edge spinose. Capillary chaetae, externally not pseudo-segmented. Capillary chaetae, internally not chambered or hollow. Silky (feltage) chaetae arising from notopodia of mid-posterior body present. Silky (feltage) chaetae incorporated into tube. Spines present; with small teeth or spinelets; present in most or all chaetigers; in ventral (neuropodial) position only. Hooks absent.

Spermathecae present.

Tube present (often very large and covered with commensal sessile organisms); membranous (mud embedded with silk-like chaetae).

**Literature used for description**. Glasby CJ, Fauchald K (2003). POLiKEY. An information system for polychaete families and higher taxa. Version 2, 5 June 2003. Australian Biological Resources Study, DCCEEW, Canberra, https:[//www.dcceew.gov.au/;](http://www.dcceew.gov.au/%3B) Hourdez S, Osborn KJ, Gonzalez BC (2022) 7.13.1.2. Acoetidae Kinberg, 1856, In: Purschke G, Böggemann M, Westheide W (Eds), Handbook of Zoology, Annelida, Volume 3: Pleistoannelida, Errantia II. De Gruyter, Berlin, 74–93; Rouse GW, Pleijel F, Tilic E (2022) Annelida. Oxford University Press, Oxford, UK.

# Acrocirridae Banse, 1969

**Description**. Body segment number: fixed or variable, less than 14 segments. Body shape elongate, more-or-less equal width over entire length or sausage or grub-shaped, regionalization absent (although some authors have recognized separate thoracic and abdominal regions on the basis of different chaetation). Body translucent, gut visible (holopelagic forms) or opaque, gut usually not visible, pigmentation absent or present, pigmentation *Acrocirrus* may be bright lime-green to yellow; epidermis papillate (over parts of the body; papillae may have adhering sediment grains). Bioluminescence absent, or present (in holopelagic species). Body surface with protective covering as gelatinous sheath (holopelagic forms), or absent. Pygidium simple lobe. Pygidial appendages absent (though may be papillate). Discrete head present; complex in shape bearing appendages; head retractable into anterior segments.

Prostomium rounded to oval or narrow, keel- or ridge-shaped, eyes on head absent or present, three pairs; head eyes simple pigmented cups. Palps present (often detached in preserved specimens); anterodorsal, or anteroventral; grooved (usually) feeding type; feeding palp longitudinally grooved. Nuchal organs present; paired low projections from posterolateral prostomium, or projecting considerably from prostomium; indistinct dorsolateral ciliated patches, or posterior projections. Peristomium a single ring.

Gut more-or-less straight, lacking side branches. Foregut a muscular ventral pharynx.

Gizzard absent. Calciferous glands absent. Circulatory system absent.

First segment achaetous (usually first several segments lack parapodia or cirri); second segment achaetous. First chaetiger with neurochaetae only, or with both notochaetae and neurochaetae; parapodia similar in length or slightly shorter than subsequent parapodia; parapodia more-or-less laterally directed and free from head, or anteriorly directed and wrapping around head (when prostomium retracted); chaetae similar in orientation, length and thickness to other chaetae. Parapodia biramous (first 1 or 2 chaetigers often with neuropodia only); parapodial lobes absent or very low. Notopodial lobes represented by at least one chaetal lobe. Neuropodial lobes represented by at least one chaetal lobe. Branchiae present (although may be missing in fixed specimens), arise from dorsal body, occur first few segments or occur on mid-body segments. Dorsal branchiae simple filaments each arising directly from body wall (called subulate branchiae).

Chaetae first appear on second segment after peristomium or third segment after peristomium or fourth segment after peristomium (chaetae may appear as late as segment 7), arranged in paired bundles (or rows) of many chaetae. Aciculae absent. Capillary chaetae present, in dorsal (notopodial) position, distally tapered to a point, edge smooth or hirsute-serrate. Capillary chaetae, externally not pseudo-segmented. Capillary chaetae, internally not chambered or hollow. Hooks absent, or present (rarely); occur in ventral (neuropodial) position; occur in posterior body only. Compound chaetae absent (rarely), or present; in ventral (neuropodial) position; appendage distally tapering to slender tips (=spinigerous), or distally curved (=falcate; ie., one distal tooth); appendage not canaliculated; appendage with a single hood open in front; joint, appearance flat or pseudocompound; joint effected by fold in external sheath of chaeta; shaft tapering slightly or evenly thick from emergence to joint; shaft, internally solid, without distinct core. Compound chaetae present in most or all chaetigers.

Segmental organs are metanephridia; metanephridia single anterior pair of excretory metanephridia and several more posterior ones for gamete release (excretory ones on segment 1).

Tube absent.

**Literature used for description**. Banse K (1969). Acrocirridae n.fam. (Polychaeta Sedentaria). Journal of the Fisheries Research Board of Canada. 26(10): 2595–2620; Glasby CJ, Fauchald K (2003). POLiKEY. An information system for polychaete families and higher taxa. Version 2, 5 June 2003. Australian Biological Resources Study, DCCEEW, Canberra, https:[//www.dcceew.gov.au/;](http://www.dcceew.gov.au/%3B) Martínez A, Worsaae K, Núñez J (2019) 7.3.1.7 Acrocirridae Banse, 1968 In: Purschke G, Böggemann M, Westheide W (Eds) Handbook of Zoology, Annelida, Volume 1: Annelida Basal Groups and Pleistoannelida, Sedentaria I. De Gruyter, Berlin, 422–439; Rouse GW, Pleijel, F, Tilic E (2022) Annelida. Oxford University Press, Oxford, UK.

# Aeolosomatidae Levinsen, 1884

**Description**. Body segment number: fixed, segmentation present (segments marked by presence of chaetae (or adhesive glands) rather than intersegmental lines; reproductive segments, or zooids, delineated by lines), less than 14 segments (although there may appear to be up to 20 segments in reproductive individuals bearing a posterior chain of zooids). Body shape elongate, more-or-less equal width over entire length or sausage or grub-shaped, dorsoventrally flattened. Body translucent, gut visible, pigmentation present (rarely absent), pigmentation most species have epidermal glands containing brightly coloured red, green or yellow oil (as seen in live specimens); epidermis more-or-less smooth. Dorsal intersegmental furrow absent or present. Underside of prostomium ciliated. Pygidium simple lobe, or bilobed. Pygidial appendages absent.

Prostomium rounded to oval (slightly flattened, wider than rest of body; bearing cilia; stiff or flexible; called 'anterior sensory cilia' in Annelida Glossary; [doi.org/10.5281/zenodo.14848165](https://doi.org/10.5281/zenodo.14848165)). Nuchal organs present; paired low projections from posterolateral prostomium; indistinct dorsolateral ciliated patches.

Gut more-or-less straight, lacking side branches or straight except for a large midbody loop (rarely). Foregut a muscular ventral pharynx.

First chaetiger with both notochaetae and neurochaetae. Chaetae present (rarely absent - in *Potamodrilus*), first appear on first segment after peristomium, arranged in paired bundles (or rows) of many chaetae. Capillary (=hair) chaetae present (rarely absent), in both dorsal and ventral positions, distally tapered to a point, edge smooth or hirsute-serrate. Capillary chaetae, externally not pseudo-segmented. Capillary chaetae, internally not chambered or hollow. Crotchet chaetae absent or present, bifid or distally pectinate.

Reproduction sexual or asexual reproduction by paratomy.

Testes present, two pairs in total (usually), one pair per segment (over two to several segments). Ovaries present (one or two in midbody), one pair (usually); present in segment V. Segmental organs are metanephridia; present in most segments of the body (usually), multiple, minute, nephridia in each segment (meronephridia). Spermathecae present (2–5 pairs in the anterior part of the body; rarely a single one (*Potamodrilus*)). Female gonoduct present (unpaired). Female pores one pair (located in ventral copulatory gland). Male gonoducts absent.

**Literature used for description**. Brinkhurst RO and Jamieson, BGM (1971) Aquatic Oligochaeta of the World with contributions by DG Cook, DV Anderson, J van der Land, University of Toronto Press, Toronto, Canada, 860 pp; Glasby CJ, Fauchald K (2003). POLiKEY. An information system for polychaete families and higher taxa. Version 2, 5 June 2003. Australian Biological Resources Study, DCCEEW, Canberra, [https://www.dcceew.gov.au/;](http://www.dcceew.gov.au/%3B) Pinder AM (2004) Annelida Aphanoneura In CM Yule and HS Yong (Eds), Freshwater Invertebrates of the Malaysian Region, Academy of Sciences Malaysia; Van der Land, J (1971) Family Aeleosomatidae, 665–707. In Aquatic Olgochaeta of the World, RO Brinkhurst & BGM Jamieson (Eds). Oliver & Boyd; HoZ Aphanoneura; Thorp JH, Lovell LL, Timm T, Martin P, Gelder SR, Govedich FR, Moser WE, Nakano T, Bielecki A, Bain BA, Utevsky S, Gil J, Glasby CJ Martin D (2019) Phylum Annelida. In: Rogers DC, Thorp JH (Eds) Keys to Palaearctic Fauna: Thorp and Covich’s Freshwater invertebrates - Volume IV. Academic Press, Elsevier, pp. 357–518; Purschke G (2022) Aphanoneura Veydovsky, 1884: Aeolosomatidae Levinsen, 1884 and Potamodrilidae Bunke, 1967 Grube, 1850 In: Purschke G, Böggemann, M, Westheide W (Eds) Handbook of Zoology. Annelida. Vol. 4: Pleistoannelida, Errantia II. De Gruyter, Berlin, 439–459; Rouse GW, Pleijel, F, Tilic E (2022) Annelida. Oxford University Press, Oxford, UK.

# Alluroididae Michaelsen, 1900

**Description**. Body segment number: variable. Secondary annulation present. Body shape elongate, more-or-less equal width over entire length. Dorsal pores on mid-dorsal line absent. Pygidium simple lobe. Pygidial appendages absent.

Prostomium bluntly conical, prolobic (prostomium demarcated from peristomium without a tongue).

Gut more-or-less straight, lacking side branches. Foregut a muscular dorsal pharynx. Gizzard absent or present.

Chaetae first appear on first segment after peristomium (=S2 for oligochaete workers), in lumbricine arrangement - paired upper and lower bundles of one or a few chaetae each - closely spaced lateral and ventrolateral pairs. Capillary (=hair) chaetae absent. Crotchet chaetae present, simple. Genital chaetae absent, or present (penial chaetae at male pores).

Sexual reproduction hermaphroditic. Clitellum thin, single layered, in region of male and female pores. Clitellum occurs from XII or XIII for a few segments. Tubercula pubertatis absent. Testes present, one pair in total (usually); present in segments X-XI. Ovaries present, one pair; present in segments XIII. Segmental organs are metanephridia; both nephridial pores and gonoducts located around clitellum. Spermathecae present, pre-testicular. Spermathecal pores present, unpaired or paired, located well anterior to male pores; 3 pairs (usually, although may be as few as 1); in segments VI to IX. Female gonoduct present. Female pores one pair, in segment XIV. Male gonoducts present, with atrium present. Male pores one pair (lateral), in segment XIII or XIV, opisthoporous (two or more segments following testicular segment). Penis present. Prostate gland present, tubular (or bulbous), one pair. Prostate pores in segments XI to XIII, prostate pore and male pore on segment XVII united, discharge through single pore.

**Literature used for description**. Brinkhurst RO and Jamieson, BGM (1971) Aquatic Oligochaeta of the World with contributions by DG Cook, DV Anderson, J van der Land, University of Toronto Press, Toronto, Canada, 860 pp; Omodeo P, Coates KA (2001) New alluroidids (Annelida, Clitellata) from Guyana. Hydrobiologia 463: 39–47; Jamieson, BGM (2006) Non-leech Clitellata (with contributions by Marco Ferraguti). Pp. 235–392. In Reproductive Biology and Phylogeny of Annelida. Series Editor BGM Jamieson. Volume 4. Editors G. Rouse, F. Pleijel. Science Publishers, Enfield, New Hampshire. Fragoso C, Rojas, P (2023). A new semiaquatic worm (Annelida, Oligochaeta) from southeastern Mexican tropical rain forests. Zootaxa 5255 (1): 136–156.

**Almidae Almidae Duboscq, 1902**

**Description**. Body segment number: variable. Body shape elongate, more-or-less equal width over entire length, more-or-less cylindrical (approaching quadrangular posteriorly; worms can flatten dorsal side of several last segment for caudal respiration). Dorsal intersegmental furrow present (?), pores on mid-dorsal line present (?). Pygidium simple lobe. Pygidial appendages absent.

Foregut a muscular dorsal pharynx. Gizzard present. Calciferous glands present. Intestinal typhlosole present. Heart bodies present, segments VIII-XI.

First segment chaetous. Branchiae present, occur adjacent to terminal segment or pygidium (caudal respiration).

Chaetae first appear on first segment after peristomium (=S2 for oligochaete workers), in lumbricine arrangement (always 2 chaetae per bundle). Crotchet chaetae present, simple-pointed. Genital chaetae present.

Sexual reproduction hermaphroditic. Clitellum thick, multilayered, posterior to male pore(s), posterior to female pore(s). Clitellum occurs from XXXV for many segments. Tubercula pubertatis present, paired ridges on ventrolateral margins of clitellum. Gonadal segments bearing genital papillae present (paired or midventral); with extensions of the body wall, as alae, or claspers. Testes present, one pair in total or two pairs in total; present in segments X and XI or XI only. Ovaries present, one pair; present in segment XIII. Segmental organs are metanephridia; both nephridial pores and gonoducts located around clitellum. Spermathecae present, post-testicular. Spermathecal pores present, located within 1 or 2 segments of male pores (just after male pore); in segment on clitellar segments from segment XXXIV. Female gonoduct present. Female pores one pair or two pairs, in segment XIV, or XIII and XIV. Male gonoducts present. Male pores one pair (may exit at the end of long claspers), in segment XIX (range XV-XXX), opisthoporous (two or more segments following testicular segment). Prostate gland absent (usually).

**Literature used for description**. Jamieson, BGM (2006) Non-leech Clitellata (with contributions by Marco Ferraguti). Pp. 235–392. In Reproductive Biology and Phylogeny of Annelida. Series Editor BGM Jamieson. Volume 4. Editors G Rouse, F Pleijel. Science Publishers, Enfield, New Hampshire; Misirlioðlu, M, Reynolds, JW, Stojanoviæ, M, Trakiæ, T, Sekuliæ, J, James, SW, Csuzdi, C, Decaëns, T, Lapied, E, Phillips, HRP, Cameron, EK, Brown, GG (2023). Earthworms (Clitellata, Megadrili) of the world: an updated checklist of valid species and families, with notes on their distribution. Zootaxa 5255 (1): 417–438.

# Alvinellidae Desbruyères & Laubier, 1986

**Description**. Body segment number: variable. Body shape widest anteriorly and tapering posteriorly. Epidermis more-or-less smooth (although commensal filamentous bacteria may be present on notopodia and dorsal surface of *Alvinella* species). Thoracic ventral glandular areas present; indistinct mid-ventral swelling. Pygidium simple lobe, or with multiple digitate lobes. Pygidial appendages absent. Discrete head present; complex in shape bearing appendages.

Prostomium hood-like, covering the tentacles dorsally (tentacles retractable into the mouth). Buccal tentacles present; arising inside mouth (can be retracted into mouth); grooved (two different sizes; larger ones only in males). Nuchal organs absent. Peristomium a single ring, extends forward as a lower lip.

Gut straight except for a large midbody loop. Foregut a muscular ventral pharynx (may bear small teeth). Heart bodies present.

First segment achaetous (may bear lateral lobes); second segment achaetous (may bear lateral lobes or dorsal branchiae). First chaetiger with notochaetae only; parapodia similar in length or slightly shorter than subsequent parapodia; parapodia more-or-less laterally directed and free from head; chaetae similar in orientation, length and thickness to other chaetae. Parapodia biramous (thorax, notopodia only), or uniramous (abdomen); parapodial lobes prominent. Notopodial lobes represented by at least one chaetal lobe. Neuropodial lobes low ridges (tori) (beginning between chaetiger 5 and posterior body). Lateral organs present. Dorsal cirri absent, or present; more-or-less cirriform. Branchiae present, arise from dorsal body, occur first few segments (branchiae either all on one segment or on four successive segments). Dorsal branchiae lamellate, or multiple filaments arising from a central stalk.

Chaetae first appear on third segment after peristomium, arranged in paired bundles (or rows) of many chaetae. Aciculae absent. Capillary chaetae present, in dorsal (notopodial) position, distally tapered to a point, edge smooth or hirsute-serrate. Capillary chaetae, externally not pseudo-segmented. Capillary chaetae, internally not chambered or hollow. Spines present; slightly curved and more-or-less smooth; present only in one or a few anterior chaetigers (between chaetigers 4–7); in dorsal (notopodial) position only. Hooks absent. Uncini present; with a single tooth surmounting main fang; in ventral (neuropodial) position throughout; arranged in one row.

Segmental organs are metanephridia; metanephridia several pairs metanephridia anteriorly for excretion, posterior ones (4–7 pairs) for gamete release; several pairs in anterior body.

Tube present; membranous, or leathery or parchment like.

**Literature used for description**. Glasby CJ, Fauchald K (2003). POLiKEY. An information system for polychaete families and higher taxa. Version 2, 5 June 2003. Australian Biological Resources Study, DCCEEW, Canberra, https:[//www.dcceew.gov.au/;](http://www.dcceew.gov.au/%3B) Stiller J, Tilic E, Rousset V, Pleijel F, Rouse GW (2020). Spaghetti to a Tree: A Robust Phylogeny for Terebelliformia (Annelida) Based on Transcriptomes, Molecular and Morphological Data. Biology 9, 73; doi:10.3390/biology9040073; Jollivet D, Hourdez S (2021) 7.7.4 Alvinellidae Desbruyères & Laubier, 1986 In: Purschke G, Böggemann M, Westheide W (Eds), Handbook of Zoology, Annelida, Volume 3: Pleistoannelida, Sedentaria III and Errantia I. De Gruyter, Berlin, 145–162; Rouse GW, Pleijel, F, Tilic E (2022) Annelida. Oxford University Press, Oxford, UK.

# Americobdellidae Caballero, 1956

**Description**. Body segment number: fixed (each segment has 14 to 16 tiny white circular sensory organs), 34 segments including 2 preoral 'segments' (prostomium and peristomium) and 32 postoral segments (true segments generally obscured by intervening superficial annulations, giving the impression of many more segments; segments VII to XXIV are complete). Secondary annulation present (except post-anally where they are absent; each annulus has 14–16 sensillae); posterior uniannulate, or 3-annulate, or 4-annulate, or 5-annulate. Body shape elongate, more-or-less equal width over entire length (the only species in the family is large and robust; body can be dramatically shortened by contraction of longitudinal muscles during movement), dorsoventrally flattened. Anterior end sucker on ventral surface present; not clearly separated from rest of body. Large mouth on ventral surface of sucker. Body pigmentation present, red or yellow mid-dorsal stripe. Posterior sucker on ventral surface present; anus positioned near posterior end.

Eyes on head absent (the presence or absence of eyes is controversial - we follow Siddall and Border (2004) who consider them to be absent; Ringulet mentions 6 pairs of eyes on segments II-IV). Caudal eyes absent.

Gut straight with side branches. Foregut a muscular axial pharynx; muscular axial pharynx ridges of pharynx rotated 60 degrees to the right (strepsilaematous); extends from XI-XII. Pharynx jaws present, leech-type (either soft-muscular or horny) (rudimentary); three (trignathous) muscular jaws arranged in a triangle (as prominent longitudinal folds, one dorsal and two ventrolateral). Protrusible proboscis absent. Crop present (segments XIII or XIV to XIX). Caeca of midgut (=posterior crop caeca) present (11 pairs). Caeca of hindgut present (one pair). Circulatory system absent.

Sexual reproduction hermaphroditic. Clitellum fully encircles body, in region of male and female pores. Clitellum occurs from X-XIII (usual position for leeches). Gonadal segments bearing a sperm transfer system in copulatory area present (as conducting strands between male reproductive apparatus and female gonoducts). Testes present, many pairs (9 pairs, almost all dendritic, epididymus in XII), one pair per segment; present in segments XIII-XXI. Sperm sac present. Ovaries present, one pair; present in segment XIII. Egg sacs present; tubular. Nephridial pores paired (ventrolateral) (? "ventral annulus b5"), present in most segments of the body (17 pairs). Female gonoduct present. Female pores single, median, in segment XII (in furrow). Male gonoducts present, with atrium present, fused (differentiated into a dorsal prostate chamber and a ventral bursa or penile sac). Male pores single, median, in segment XI (in furrow). Penis present (short, conical). Prostate gland absent.

**Literature used for description**. Ringuelet RA (1985). Sinopsis de los Hirudineos de Chile (Annelida). Bolitino de Societe de Concepcion, Chile 56: 163–179; Siddall ME, Borda E (2004) Leech Collections from Chile Including Two New species of *Helobdella* (Annelida: Hirudinida). American Museum Novitates 3457: 1–18; Siddall ME, Bely AE, Borda E (2006) Hirudinida Pp. 393–429. In Reproductive Biology and Phylogeny of Annelida. Series Editor BGM Jamieson. Volume 4. Editors G Rouse, F Pleijel. Science Publishers, Enfield, New Hampshire.

# Ampharetidae Malmgren, 1866

**Description**. Body segment number: variable. Body shape widest anteriorly and tapering posteriorly, regionalization present, regionalization comprising two regions, regions demarcated by absence of abdominal notopodia. Body pigmentation absent or present, including banding on branchiae or pigment spots on dorsal body; epidermis more-or-less smooth. Thoracic ventral glandular areas present; indistinct mid-ventral swelling. Pygidium simple lobe. Pygidial appendages present, one pair of cirri or more than four cirri. Discrete head present; complex in shape bearing appendages.

Prostomium hood-like, covering the tentacles dorsally (tentacles retractable into the mouth), eyes on head absent or present, numerous, unpaired; head eyes simple pigmented cups. Buccal tentacles present; arising inside mouth (on dorsal fold; can be retracted into mouth); smooth, or pinnulated. Nuchal organs present; paired low projections from posterolateral prostomium; indistinct dorsolateral ciliated patches. Peristomium a single ring, extends forward as a lower lip.

Gut straight except for a large midbody loop. Foregut a muscular ventral pharynx (may bear small teeth). Heart bodies present.

First segment achaetous (may bear lateral lobes); second segment chaetous. First chaetiger with notochaetae only; parapodia similar in length or slightly shorter than subsequent parapodia; parapodia more-or-less laterally directed and free from head; chaetae similar in orientation, length and thickness to other chaetae, or projecting obliquely, distinctly thicker and more shiny than subsequent ones (=paleae). Parapodia biramous (thorax), or uniramous (abdomen); parapodial lobes prominent. Notopodial lobes represented by at least one chaetal lobe (in anterior body; absent posteriorly). Neuropodial lobes low ridges (tori) (beginning chaetiger 5; may bear a dorsal cirrus). Lateral organs absent, or present. Branchiae present (usually 4 pairs), arise from dorsal body, occur first few segments (segments 2–5). Dorsal branchiae simple filaments each arising directly from body wall (may be flattened or not, smooth or pinnate).

Chaetae first appear on second segment after peristomium or third segment after peristomium, arranged in paired bundles (or rows) of many chaetae. Aciculae absent. Capillary chaetae present, in dorsal (notopodial) position, distally tapered to a point, edge smooth or hirsute-serrate. Capillary chaetae, externally not pseudo-segmented. Paleate chaetae absent, or present, associated with notopodia. Parapodial paleae arranged in two lateral bunches on segment 2. Spines absent, or present; sharply bent (=geniculate) or recurved (referred to as golden paleae); present only in one or a few anterior chaetigers (on first chaetiger, when present); in dorsal (notopodial) position only. Hooks absent. Uncini present; with teeth in vertical series, teeth usually similar-sized (=pectinate) (rarely crested); in ventral (neuropodial) position throughout; arranged in one row.

Segmental organs are metanephridia; metanephridia several pairs metanephridia anteriorly for excretion, posterior ones for gamete release (4–7 pairs); nephridial pores, several pairs in anterior body.

Tube present, membranous.

**Literature used for description**. Glasby CJ, Fauchald K (2003). POLiKEY. An information system for polychaete families and higher taxa. Version 2, 5 June 2003. Australian Biological Resources Study, DCCEEW, Canberra, https:[//www.dcceew.gov.au/;](http://www.dcceew.gov.au/%3B) Stiller J, Tilic E, Rousset V, Pleijel F, Rouse GW (2020). Spaghetti to a Tree: A Robust Phylogeny for Terebelliformia (Annelida) Based on Transcriptomes, Molecular and Morphological Data. Biology 9, 73; doi:10.3390/biology9040073; Alvestad T, Budaeva N (2021) Key to the species in Norwegian waters from the families Ampharetidae and Melinnidae. Online at https:[//www.artsdatabanken.no/Pages/302041/Key_to_the_species_in;](http://www.artsdatabanken.no/Pages/302041/Key_to_the_species_in%3B) Ebbe B, Purschke G (2021) 7.7.2 Ampharetidae Malmgren, 1866 In: Purschke G, Böggemann M, Westheide W (Eds), Handbook of Zoology, Annelida, Volume 3: Pleistoannelida, Sedentaria III and Errantia I. De Gruyter, Berlin, 50–67; Rouse GW, Pleijel, F, Tilic E (2022) Annelida. Oxford University Press, Oxford, UK.

# Amphinomidae Lamarck, 1818

**Description**. Body segment number: variable. Body shape elongate, more-or-less equal width over entire length or ovate to elliptical. Body pigmentation absent (many *Eurythoe*) or present, red-brown, green or white spots and stripes on dorsal surface; chaetae often appear yellowish-white; epidermis more-or-less smooth (often with dorsolateral longitudinal grooves). Pygidium simple lobe. Pygidial appendages present, one pair of cirri and single medial papilla. Discrete head present (but enclosed by surrounding parapodia); complex in shape bearing appendages.

Prostomium triangular to trapezoidal (narrow end posteriorly), eyes on head present, two pairs; head eyes simple pigmented cups. Prostomial antennae present; median and paired laterals; unarticulated. Palps present (resemble antennae); anteroventral; tapering (usually) sensory type; unarticulated, or multiple articulations (weakly). Caruncle present. Nuchal organs present; projecting considerably from prostomium (ciliated bands on either side of caruncle). Peristomium not visible.

Foregut a muscular ventral pharynx (mouth ventral, surrounded by paired ventrolateral lips). Protrusible proboscis present, with transverse ridges of thick cuticular lamellae, distal ring of papillae absent. Pharynx dorsolateral ciliated folds present.

First segment chaetous; second segment chaetous. First chaetiger with both notochaetae and neurochaetae; parapodia similar in length or slightly shorter than subsequent parapodia; parapodia anteriorly directed and wrapping around head, or more-or-less laterally directed and free from head; chaetae similar in orientation, length and thickness to other chaetae (rarely has stout recurved notopodial hooks). Parapodia biramous; parapodial lobes prominent. Notopodial lobes represented by at least one chaetal lobe. Neuropodial lobes represented by at least one chaetal lobe. Lateral organs present. Dorsal cirri present; more-or-less cirriform. Ventral cirri present; cirriform or tapering. Branchiae present, arise from lateral body (at base), occur on mid-body segments; lateral branchiae single tuft per parapodium.

Chaetae first appear on first segment after peristomium, calcareous, brittle, arranged in paired bundles (or rows) of many chaetae. Aciculae present; in both dorsal and ventral positions (=noto- and neuroaciculae) (numerous). Neuroaciculae distally tapering. Capillary chaetae present, in dorsal (notopodial) position or in ventral (neuropodial) position, distally tapered to a point or with subdistal spur, edge smooth or hirsute-serrate. Capillary chaetae, externally not pseudo-segmented. Capillary chaetae, internally hollow (tubular). Forked chaetae present, furcate, tines distinctly unequal in length. Spines present; slightly curved and more-or-less smooth (rarely distally recurved), or harpoon or arrow-shaped with reverse barbs; present in most or all chaetigers; in both dorsal and ventral positions. Hooks absent.

Tube absent.

**Literature used for description**. Kudenov, J. (1993) Amphinomidae and Euphrosinidae (Annelida: Polychaeta) Principally from Antarctica, The Southern Ocean, and Subantarctic Regions. Antarctic Research Series 58: 93–150; Glasby CJ, Fauchald K (2003). POLiKEY. An information system for polychaete families and higher taxa. Version 2, 5 June 2003. Australian Biological Resources Study, DCCEEW, Canberra, https:[//www.dcceew.gov.au/;](http://www.dcceew.gov.au/%3B) Rouse GW, Pleijel, F, Tilic E (2022) Annelida. Oxford University Press, Oxford, UK.

**Aphroditidae Malmgren, 1867**

**Description**. Body segment number: variable. Body shape elongate, more-or-less equal width over entire length or ovate to elliptical, dorsoventrally flattened. Body pigmentation present, iridescent chaetae; epidermis papillate (ventrally). Body surface with protective covering as scales (elytrae), dorsally (fine capillaries produced by the worm may cover the elytrae giving the worm a furry appearance). Pygidium simple lobe. Pygidial appendages present, one pair of cirri. Discrete head present; complex in shape bearing appendages.

Prostomium rounded to oval, ommatophores present, as low ocular mounds or as stalk-like ocular peduncles, facial tubercle present (absent in genus *Heteraphrodita*), eyes on head present, two pairs; head eyes compound, with lenses. Prostomial antennae present; median one only; unarticulated, or consist of basal ceratophore and distal ceratostyle. Palps present; anteroventral; tapering (usually) sensory type; unarticulated. Nuchal organs present; paired low projections from posterolateral prostomium; indistinct dorsolateral ciliated patches. Peristomium not visible.

Gut more-or-less straight, lacking side branches or straight with side branches (as caeca in the hindgut). Foregut a muscular axial pharynx. Pharynx jaws absent or present, two pairs of jaws. Paired jaws plate-like. Protrusible proboscis present, distal ring of papillae present.

First segment chaetous and tentaculate (=elongate dorsal and ventral cirri); second segment chaetous. Tentacular cirri present. First chaetiger without external chaetae, or with notochaetae only; parapodia similar in length or slightly shorter than subsequent parapodia; parapodia anteriorly directed and wrapping around head; chaetae similar in orientation, length and thickness to other chaetae. Parapodia biramous; parapodial lobes prominent. Notopodial lobes represented by at least one chaetal lobe. Neuropodial lobes represented by at least one chaetal lobe. Dorsal cirri present (on non-elytrous segments; except *Palmyra*); more-or-less cirriform. Elytra with papillae, tubercles or smooth. Elytra, post segment 7, occur on every other segment from segment 7 to end of body. Ventral cirri present; cirriform or tapering.

Chaetae first appear on first segment after peristomium or second segment after peristomium, arranged in paired bundles (or rows) of many chaetae (notochaetae, including felt-like chaetae, often extending over dorsal body). Aciculae present; in both dorsal and ventral positions (=noto- and neuroaciculae). Capillary chaetae present, in dorsal (notopodial) position, distally tapered to a point, edge smooth. Capillary chaetae, externally not pseudo-segmented. Capillary chaetae, internally not chambered or hollow. Silky (feltage) chaetae arising from notopodia of mid-posterior body present. Silky (feltage) chaetae forming a felt cover on dorsal surface. Paleate chaetae absent, or present, associated with notopodia. Parapodial paleae arranged in rosettes across dorsum (*Palmyra* only). Spines present; slightly curved and more-or-less smooth, or with small teeth or spinelets, or harpoon or arrow-shaped with reverse barbs; present in most or all chaetigers; in both dorsal and ventral positions. Hooks absent.

Segmental organs are metanephridia; metanephridia along most of the body; anterior ones for excretion, posterior ones for gamete release; present in most segments of the body.

Tube absent.

**Literature used for description**. Pettibone MH (1966) *Heteraphrodita altoni,* a new genus and species of polychaete wonn (Polychaeta, Aphroditidae) from deep water off Oregon, and a revision of the aphroditid genera. Proceedings of the Biological Society of Washington 79: 95–108; Glasby CJ, Fauchald K (2003). POLiKEY. An information system for polychaete families and higher taxa. Version 2, 5 June 2003. Australian Biological Resources Study, DCCEEW, Canberra, https:[//www.dcceew.gov.au/;](http://www.dcceew.gov.au/%3B) Rouse GW, Pleijel, F, Tilic E (2022) Annelida. Oxford University Press, Oxford, UK.

# Apistobranchidae Mesnil & Caullery, 1898

**Description**. Body segment number: variable. Body shape elongate, more-or-less equal width over entire length, regionalization absent (structure of the parapodia change a few times along the body, although there is no definite demarcation). Epidermis more-or-less smooth (dense cilia mid-ventrally (=ventral ciliary band)). Pygidium simple lobe. Pygidial appendages present, four cirri. Discrete head present; complex in shape bearing appendages. Prostomium bluntly conical (covered with cilia, particularly ventrally). Palps present; anterodorsal; grooved (usually) feeding type (often very long); feeding palp longitudinally grooved. Nuchal organs present; paired low projections from posterolateral prostomium; posterior projections. Peristomium a single ring, may be obscured by nuchal organs dorsally.

Foregut a non-muscular axial pharynx. Pharynx dorsolateral ciliated folds present.

First segment chaetous. First chaetiger with neurochaetae only; parapodia similar in length or slightly shorter than subsequent parapodia; parapodia more-or-less laterally directed and free from head; chaetae similar in orientation, length and thickness to other chaetae. Parapodia biramous (neuropodia are the larger of the two branches); parapodial lobes prominent. Notopodial lobes slender, flask- or spindle-shaped (absent from segment 1 and posterior segments, and sometimes also some anterior to mid body segments). Neuropodial lobes represented by at least one chaetal lobe (with a series of flattened lamellae ventral to the neuropodia on chaetiger 7). Interramal fleshy process present (on anterior segments). Interramal fleshy process cirrus-like (long, resembling a dorsal cirrus). Lateral organs present. Dorsal cirri absent. Ventral cirri present; cirriform or tapering.

Chaetae first appear on first segment after peristomium, arranged as a single bundle (or row) of many chaetae (lower bundle - neurochaetae - only). Aciculae present; in both dorsal and ventral positions (=noto- and neuroaciculae). Neuroaciculae distally tapering. Capillary chaetae present, in ventral (neuropodial) position, distally tapered to a point, edge smooth. Capillary chaetae, externally not pseudo-segmented. Capillary chaetae, internally not chambered or hollow. Hooks absent.

Segmental organs are metanephridia; metanephridia along most of the body; anterior ones for excretion, posterior ones for gamete release.

Tube present, membranous.

**Literature used for description**. Glasby CJ, Fauchald K (2003). POLiKEY. An information system for polychaete families and higher taxa. Version 2, 5 June 2003. Australian Biological Resources Study, DCCEEW, Canberra, https:[//www.dcceew.gov.au/;](http://www.dcceew.gov.au/%3B) Blake JA, Petti MAV (2019) 5.1 Apistobranchidae Mesnil & Caullery, 1898 In Purschke G, Böggemann M, Westheide W (Eds), Handbook of Zoology, Annelida, Volume 1: Annelida Basal Groups and Pleistoannelida, Sedentaria I. De Gruyter, Berlin, 133–142; Rouse GW, Pleijel, F, Tilic E (2022) Annelida. Oxford University Press, Oxford, UK.

# Arenicolidae Johnston, 1835

**Description**. Body segment number: variable. Secondary annulation present (typically 5 annuli per segment, except for anterior ones where the number of annuli is reduced). Body shape widest anteriorly and tapering posteriorly, regionalization absent or present, regionalization comprising two regions or three regions, regions demarcated by structural differences in parapodia over body (branchial or abranchial). Caudal end with a prominent achaetous sacrificial region (at least in *Arenicola* and *Abarenicola*). Body pigmentation present, dark red, green to black (large forms); epidermis thick and rugose. Pygidium simple lobe. Pygidial appendages absent. Discrete head present; lobe-like without appendages.

Prostomium bluntly conical, eyes on head absent or present, numerous, unpaired; head eyes simple pigmented cups. Nuchal organs present; paired low projections from posterolateral prostomium; indistinct dorsolateral ciliated patches. Peristomium a single ring, may be incomplete dorsally.

Gut straight with side branches (one to several pairs diverticula on posterior oesophagus). Foregut a muscular axial pharynx (adult state) or a muscular ventral pharynx (juvenile state). Protrusible proboscis present, with papillae in subterminal position, irregularly arranged, distal ring of papillae absent. Pharynx dorsolateral ciliated folds absent or present. Heart bodies present (in larger forms).

First segment achaetous; second segment chaetous (biramous or notopodia only). First chaetiger with both notochaetae and neurochaetae (rarely notopodia only); parapodia similar in length or slightly shorter than subsequent parapodia; parapodia more-or-less laterally directed and free from head; chaetae similar in orientation, length and thickness to other chaetae. Parapodia biramous; parapodial lobes absent or very low. Notopodial lobes represented by at least one chaetal lobe (low, rounded lobe). Neuropodial lobes low ridges (tori) (over most of body). Lateral organs present. Branchiae present, arise from lateral body (at base), occur on mid-body segments or occur near posterior end; lateral branchiae digitiform (rarely), or single tuft per parapodium.

Chaetae first appear on second segment after peristomium, arranged in paired bundles (or rows) of many chaetae. Aciculae absent. Capillary chaetae present, in dorsal (notopodial) position, distally tapered to a point, edge hirsute-serrate. Capillary chaetae, externally not pseudo-segmented. Capillary chaetae, internally not chambered or hollow. Hooks present (long handled, dentate); without distal hood, beard or ligament, or with a subdistal beard (juveniles only); occur in ventral (neuropodial) position; occur over entire body.

Segmental organs are metanephridia; metanephridia several pairs metanephridia anteriorly for excretion, posterior ones for gamete release (4–7 pairs, rarely up to 13 pairs); opening between segments 5–16, several pairs in anterior body.

Tube absent, or present, membranous (rarely).

**Literature used for description**. Glasby CJ, Fauchald K (2003). POLiKEY. An information system for polychaete families and higher taxa. Version 2, 5 June 2003. Australian Biological Resources Study, DCCEEW, Canberra, https:[//www.dcceew.gov.au/;](http://www.dcceew.gov.au/%3B) Darbyshire T (2021) 7.7.5 Arenicolidae Johnston, 1835 In: Purschke G, Böggemann M, Westheide W (Eds), Handbook of Zoology, Annelida, Volume 3: Pleistoannelida, Sedentaria III and Errantia I. De Gruyter, Berlin, 163–185; Rouse GW, Pleijel, F, Tilic E (2022) Annelida. Oxford University Press, Oxford, UK.

# Biwadrilidae Jamieson, 1971

**Description**. Body segment number: variable. Body shape elongate, more-or-less equal width over entire length, more-or-less cylindrical (canaliculate posteriorly). Dorsal intersegmental furrow present, pores on mid-dorsal line absent. Pygidium simple lobe. Pygidial appendages absent.

Prostomium bluntly conical, zygolobic (prostomium not demarcated).

Foregut a muscular dorsal pharynx. Gizzard absent. Intestinal typhlosole absent. Heart bodies present, segments VII-XI.

Chaetae first appear on first segment after peristomium (=S2 for oligochaete workers), two per bundle, in lumbricine arrangement with lateral and ventrolateral pairs closely spaced. Crotchet chaetae present. Genital chaetae present (bifid penial chaetae at male pores).

Sexual reproduction hermaphroditic. Clitellum long, with inconspicuous borders, thick, multilayered, fully encircles body, in region of male pores (covering male pores at least with its anterior edge), posterior to female pores. Clitellum occurs from XIV to XXXII. Seminal groove absent. Tubercula pubertatis present. Gonadal segments bearing genital papillae absent. Gonadal segments with extensions of the body wall absent. Testes present, two pairs in total, one pair per segment; present in segments X and XI. Sperm sac absent. Ovaries present, one pair; present in segment XIII. Segmental organs are metanephridia; nephridial pores paired (ventrolateral) (restricted to clitellum), both nephridial pores and gonoducts located around clitellum. Female gonoduct present. Female pores one pair, in segment XIV. Male gonoducts present (2 pairs), with atrium present. Male pores one pair, in segment XIII, opisthoporous (two or more segments following testicular segment). Prostate gland present (diffuse prostate), lobular, one pair; prostate pore and male pore on segment XVII united, discharge through single pore.

**Literature used for description**. Brinkhurst RO and Jamieson, BGM (1971) Aquatic Oligochaeta of the World with contributions by DG Cook, DV Anderson, J van der Land, University of Toronto Press, Toronto, Canada, 860 pp; Jamieson, BGM (2006) Non-leech Clitellata (with contributions by Marco Ferraguti). Pp. 235–392. In Reproductive Biology and Phylogeny of Annelida. Series Editor BGM Jamieson. Volume 4. Editors G Rouse, F Pleijel. Science Publishers, Enfield, New Hampshire; Blakemore RJ (2008) Review of Criodrilidae (Annelida: Oligochaeta) including *Biwadrilus* from Japan. Opuscula Zoologica Budapest 37: 11–22; Thorp JH, Lovell LL, Timm T, Martin P, Gelder SR, Govedich FR, Moser WE, Nakano T, Bielecki A, Bain BA, Utevsky S, Gil J, Glasby CJ, Martin D (2019) Phylum Annelida. In: Rogers DC, Thorp JH (Eds) Keys to Palaearctic Fauna: Thorp and Covich’s Freshwater invertebrates - Volume IV. Academic Press, Elsevier, pp. 357–518; Misirlioðlu, M, Reynolds, JW, Stojanoviæ, M, Trakiæ, T, Sekuliæ, J, James, SW, Csuzdi, C, Decaëns, T, Lapied, E, Phillips, HRP, Cameron, EK, Brown, GG (2023). Earthworms (Clitellata, Megadrili) of the world: an updated checklist of valid species and families, with notes on their distribution. Zootaxa 5255 (1): 417–438.

# Branchiobdellidae Grube, 1850

**Description**. Body segment number: fixed, 15 segments (11 segments externally obvious; 4 segments fused into head). Secondary annulation present. Body segments similar dimensions throughout. Body shape sausage or grub-shaped or pyriform, dorsoventrally flattened or more-or-less cylindrical. Anterior end sucker on ventral surface absent (true sucker replaced by an adhesive disk). Epidermis more-or-less smooth, or thick and rugose. Dorsal body surface, appearance under compound microscope smooth. Posterior sucker on ventral surface present; circular; rays absent; anus positioned near posterior end (dorsally, just before posterior sucker). Discrete head present (comprising the peristomium and first 4 segments; 16 oral papillae surround the mouth in most Branchiobdellidae).

Gut more-or-less straight, lacking side branches. Foregut a muscular axial pharynx; extends from first 4 segments. Pharynx jaws present, one pair of, fang-like dorsal-ventral jaws (broad at base; may be serrated near apex)Protrusible proboscis absent. Circulatory system present.

Chaetae absent.

Sexual reproduction hermaphroditic. Clitellum in region of male and female pores, from IX-XI. Testes present, two pairs in total; present in segments X and XI. Ovaries present, one pair; present in segment XII. Nephridial pores dorsal (segment 3) or lateral (segment 9), located anteriorly, gonoducts located around clitellum, several pairs in anterior body, one pair nephridia in each segment (holonephridia) (segments 2–4 and 8). Spermathecae present. Spermathecal pores present, located within 1 or 2 segments of male pores; 1 pair; in segment IX. Female gonoduct present. Female pores single, median, in segment X. Male gonoducts present, with atrium present. Male pores single, median, in segment X. Penis present. Prostate gland present, tubular, one pair. Prostate pores in segment X; prostate pore and male pore on segment XVII united, discharge through single pore.

**Literature used for description**. Gelder, Stuart R. (1996). A review of the taxonomic nomenclature and a checklist of the species of the Branchiobdellae (Annelida: Clitellata). Proceedings of the Biological Society of Washington. 109(4): 653–663; Govedich FR, Bain BA, Moser WE, Gelder SR, Davies RW, Brinkhurst RO (2009) Annelida (Clitellata): Oligochaeta, Branchiobdellida Hirudinida, and Acanthobdellida. In: Thorp JH, Covich AP (eds) Ecology and Classification of North American Freshwater Invertebrates, 3rd Edition. Elsevier Inc., 385–436; Brusca, RC, Moore, W & Shuster, SM. (eds) (2016). Chapter 14. Phylum Annelida. In: Invertebrates, 3rd Edition. Sinauer Associates, Sunderland, MA; Thorp JH et al. 2019. Phylum Annelida, In Keys to Palaearctic Fauna: Thorp and Covich's Freshwater Invertebrates, Volume IV. Academic Press, Elsevier.

# Capilloventridae Harman & Loden, 1984

**Description**. Body segment number: variable. Secondary annulation absent or present; mid-body triannulate, or 4-annulate, or 5-annulate. Body shape elongate, more-or-less equal width over entire length or sausage or grub-shaped. Epidermis more-or-less smooth. Pygidium simple lobe. Pygidial appendages absent.

Prostomium bluntly conical (rarely elongated), zygolobic (prostomium not demarcated).

Foregut a muscular dorsal pharynx.

Dorsal and ventral bundle chaetae first appear on second segment or third segment after peristomium, in lumbricine arrangement - paired upper and lower bundles of one or a few chaetae each - closely spaced lateral and ventrolateral pairs. Capillary (=hair) chaetae present, in both dorsal and ventral positions, distally tapered to a point, edge hirsute-serrate (may be elongated in anterior segments). Crotchet chaetae present, simple or bifid. Genital chaetae present (broad capillary penial chaetae ventrally on XII).

Sexual reproduction hermaphroditic. Clitellum thin, single layered, fully encircles body, in region of male and female pores. Clitellum occurs from XII to XIV. Testes present, one pair in total; present in segments XI or XII (XI according to Pinder and Brinkhurst (1997)). Ovaries present, one pair; present in segments XII or XIII (XII according to Brinkhurst and Jamieson (1977)). Segmental organs are metanephridia; both nephridial pores and gonoducts located around clitellum. Spermathecae present, pre-testicular. Spermathecal pores located well anterior to male pores, 1 pair; in segment VI/VII, intersegmental. Female gonoducts present. Female pores one pair, in segment XIII (or XIV). Male gonoducts present, with atrium absent. Male pores one pair, in segment XIII, plesioporous (in segment following testicular segment). Penis absent. Prostate gland absent.

**Literature used for description**. Harman, WJ, Loden MS (1984) *Capilloventer atlanticus* gen. et sp.n., a member of a new family of marine Oligochaeta from Brazil. Hydrobiologia 115, 51–54; Pinder AM, Brinkhurst RO (1997) The family Capilloventridae (Annelida, Clitellata) in Australia, with descriptions of two new species of *Capilloventer.* Zoologica Scripta 26: 255–265; Jamieson, BGM (2006) Non-leech Clitellata (with contributions by Marco Ferraguti). Pp. 235–392. In Reproductive Biology and Phylogeny of Annelida. Series Editor BGM Jamieson. Volume 4. Editors G Rouse, F Pleijel. Science Publishers, Enfield, New Hampshire; Martin, P, Martinez-Ansemil, E., Pinder, A, Timm, T, Wetzel, MJ (2008) Global diversity of oligochaetous clitellates (‘‘Oligochaeta’’; Clitellata) in freshwater. Hydrobiologia (2008) 595:117–127; Timm T (2012) Life forms in Oligochaeta: a literature review. Zoology in the Middle East, Supplement 4, 58: 71–82; Pinder, A (2013). Tools for identifying Australian aquatic oligochaetes of the families Phreodrilidae, Lumbriculidae and Capilloventridae (Clitellata: Annelida). Museum Victoria Science Reports 18: 1–20; Thorp JH, Lovell LL, Timm T, Martin P, Gelder SR, Govedich FR, Moser WE, Nakano T, Bielecki A, Bain BA, Utevsky S, Gil J, Glasby CJ, Martin D (2019) Phylum Annelida. In: Rogers DC, Thorp JH (Eds) Keys to Palaearctic Fauna: Thorp and Covich’s Freshwater invertebrates - Volume IV. Academic Press, Elsevier, pp. 357–518.

# Capitellidae Grube, 1862

**Description**. Body segment number: variable. Body shape elongate, more-or-less equal width over entire length, regionalization present, regionalization comprising two regions (short thorax and long abdomen), regions demarcated by change in chaetal types over body. Epidermis more-or-less smooth, or thick and rugose (anteriorly). Dorsal body surface, appearance under compound microscope smooth. Pygidium simple lobe, or plate-like (rarely, characteristic of *Scyphoproctus*). Pygidial appendages absent (usually) or present, one pair of cirri or single medial cirrus. Discrete head present; lobe-like without appendages.

Prostomium bluntly conical, eyes on head absent or present, one pair or numerous, unpaired; head eyes simple pigmented cups. Nuchal organs present; paired low projections from posterolateral prostomium; indistinct dorsolateral ciliated patches. Peristomium a single ring, may sometimes appear double in fixed specimens.

Foregut a muscular dorsal pharynx (though early studies referred to an axial pharynx).

Circulatory system absent. First segment chaetous, or achaetous (at least for *Leocapitellides, Mastobranchus, Pseudonotomastus, Schyphoproctus*); second segment chaetous. First chaetiger with notochaetae only, or with neurochaetae only, or with both notochaetae and neurochaetae; parapodia similar in length or slightly shorter than subsequent parapodia; parapodia more-or-less laterally directed and free from head; chaetae similar in orientation, length and thickness to other chaetae. Parapodia biramous; parapodial lobes absent or very low. Notopodial lobes low lateral ridges (tori) (posteriorly). Neuropodial lobes low ridges (tori) (posteriorly). Lateral organs present. Branchiae absent or present (=coelomic branchiae; may be retractile), arise from lateral body or arise from dorsal body, occur on mid-body segments; lateral branchiae digitiform, or branching. Dorsal branchiae lamellate.

Chaetae arranged in paired bundles (or rows) of many chaetae. Aciculae absent. Capillary chaetae present (only in anterior segments) or absent (rarely), in dorsal (notopodial) position or in ventral (neuropodial) position, distally tapered to a point, edge smooth (sometimes with 'wings'). Capillary chaetae, externally not pseudo-segmented. Capillary chaetae, internally not chambered or hollow. Spines absent, or present (rarely); slightly curved and more-or-less smooth; present only in one or a few anterior chaetigers, or present only in posterior chaetigers (genital spines occur in chaetigers 8, 9 in sexually mature individuals in a few genera; other genera have spines in the notopodia of pre-anal segments). Hooks present (long-handled hooded hooks); with a distal hood; occur in dorsal (notopodial) position, or occur in ventral (neuropodial) position; occur over entire body.

Segmental organs are metanephridia (although not apparently associated with gamete release); metanephridia restricted to posterior thorax and anterior abdomen (those in *Dasybranchus* extend along the abdomen); nephridial pores, several pairs in anterior body.

Tube absent, or present; membranous.

**Literature used for description**. Glasby CJ, Fauchald K (2003). POLiKEY. An information system for polychaete families and higher taxa. Version 2, 5 June 2003. Australian Biological Resources Study, DCCEEW, Canberra, https:[//www.dcceew.gov.au/;](http://www.dcceew.gov.au/%3B) Magalhães WF, Blake JA (2019) 7.6.4 Capitellidae Grube, 1862, In Purschke G, Böggemann M, Westheide W (Eds), Handbook of Zoology, Annelida, Volume 2: Pleistoannelida, Sedentaria II. De Gruyter, Berlin, 349–403; Rouse GW, Pleijel, F, Tilic, E (2022) Annelida. Oxford University Press, Oxford, UK.

# Chaetopteridae Audouin & Milne Edwards, 1833

**Description**. Body segment number: variable. Body shape elongate, more-or-less equal width over entire length (rarely highly modified with spherical body), regionalization present, regionalization comprising three regions, regions demarcated by structural differences in parapodia over body (midbody with lamellate, piston-like segments in some genera; inflated in the holopelagic *Chaetopterus porcinus*). Body pigmentation absent or present, palps may be banded and posterior body is translucent and typically appears dark because of gut contents; epidermis more-or-less smooth. Bioluminescence absent, or present (in benthic and holopelagic species). Pygidium simple lobe. Pygidial appendages absent. Discrete head present; complex in shape bearing appendages.

Prostomium rounded to oval, eyes on head absent or present, one pair; head eyes simple pigmented cups. Palps present; anterodorsal; grooved (usually) feeding type; feeding palp longitudinally grooved. Nuchal organs absent, or present; paired low projections from posterolateral prostomium; posterior projections. Peristomium visible. Peristomium a single ring, collar-like (collar projects anteriorly and laterally).

Foregut without a distinct ventral or axial pharynx.

First segment chaetous (one pair elongated dorsal cirri may be present); second segment chaetous. First chaetiger with notochaetae only; parapodia similar in length or slightly shorter than subsequent parapodia; parapodia more-or-less laterally directed and free from head; chaetae similar in orientation, length and thickness to other chaetae. Parapodia uniramous (anterior region), or biramous (mid- and posterior regions); parapodial lobes prominent. Notopodial lobes represented by at least one chaetal lobe (midbody notopodial lobes large, aliform used in pumping water). Neuropodial lobes low ridges (tori) (mid- and posterior chaetigers only).

Chaetae arranged in paired bundles (or rows) of many chaetae or as a single bundle (or row) of many chaetae (lower bundle - neurochaetae - only). Aciculae absent, or present (in posterior region); in dorsal position (=notoaciculae). Neuroaciculae distally tapering. Capillary chaetae present, in dorsal (notopodial) position, distally tapered to a point or expanded or knife-shaped, edge smooth. Capillary chaetae, externally not pseudo-segmented. Capillary chaetae, internally not chambered or hollow. Spines present; slightly curved and more-or-less smooth (robust); present in most or all chaetigers (on chaetiger 4 only); in both dorsal and ventral positions. Hooks absent. Uncini present; with teeth in vertical series, teeth usually similar-sized (=pectinate); in ventral (neuropodial) position throughout; arranged in more than two rows.

Segmental organs are metanephridia; metanephridia along most of the body; anterior ones for excretion, posterior ones for gamete release (nephridia lacking in anteriormost region).

Tube present; membranous, or leathery or parchment like, or translucent, chitin-like (U-shaped, straight or branched).

**Literature used for description**. Glasby CJ, Fauchald K (2003). POLiKEY. An information system for polychaete families and higher taxa. Version 2, 5 June 2003. Australian Biological Resources Study, DCCEEW, Canberra, https:[//www.dcceew.gov.au/;](http://www.dcceew.gov.au/%3B) Britayev TA, Martin D (2019) 5.3 Chaetopteridae Audouin & Milne Edwards, 1833 In Purschke G, Böggemann M, Westheide W (Eds), Handbook of Zoology, Annelida, Volume 1: Annelida Basal Groups and Pleistoannelida, Sedentaria I. De Gruyter, Berlin, 156–176; Rouse GW, Pleijel, F, Tilic, E (2022) Annelida. Oxford University Press, Oxford, UK.

# Chrysopetalidae Ehlers, 1864 sensu lato

**Description**. Body segment number: variable. Body shape elongate, more-or-less equal width over entire length or ovate to elliptical, dorsoventrally flattened or more-or-less cylindrical. Epidermis more-or-less smooth. Dorsal body surface, appearance under compound microscope smooth. Body surface with protective covering as shield-like spines (paleae), dorsally. Pygidium simple lobe. Pygidial appendages absent or present, one pair of cirri or one pair of cirri and single medial papilla. Discrete head present; complex in shape bearing appendages.

Prostomium rounded to oval, eyes on head absent or present, one pair or two pairs; head eyes compound, with lenses. Prostomial antennae present; median and paired laterals; unarticulated. Palps present; anteroventral; tapering (usually) sensory type; unarticulated, or biarticulated (faintly). Nuchal organs present; paired low projections from posterolateral prostomium; indistinct dorsolateral ciliated patches. Peristomium not visible.

Foregut a muscular axial pharynx. Pharynx jaws present, one pair of lateral jaws. Paired jaws fang-like (called stylet jaws). Protrusible proboscis present, smooth, distal ring of papillae absent or present.

First segment tentaculate; second segment chaetous (usually lack the ventral components). Tentacular cirri present; 2 pair(s); arise on a single segment; internal aciculae absent. First chaetiger with notochaetae only, or with both notochaetae and neurochaetae; parapodia similar in length or slightly shorter than subsequent parapodia; parapodia more-or-less laterally directed and free from head; chaetae similar in orientation, length and thickness to other chaetae. Parapodia biramous; parapodial lobes prominent. Notopodial lobes represented by at least one chaetal lobe. Neuropodial lobes represented by at least one chaetal lobe. Dorsal cirri present; more-or-less cirriform. Ventral cirri present (may be lacking on anterior chaetigers); cirriform or tapering.

Chaetae first appear on second segment after peristomium, arranged in paired bundles (or rows) of many chaetae (notochaetae may be arranged in rows). Aciculae present; in both dorsal and ventral positions (=noto- and neuroaciculae) (notoaciculae need confirmation). Neuroaciculae distally tapering. Capillary chaetae absent (their simple chaetae are here interpreted as spines). Paleate chaetae present, associated with notopodia. Parapodial paleae arranged in linear fans across dorsum. Spines absent, or present; with small teeth or spinelets; present in most or all chaetigers; in dorsal (notopodial) position only. Hooks absent. Compound chaetae present; in dorsal (notopodial) position, or in ventral (neuropodial) position; appendage distally tapering to slender tips (=spinigerous), or distally curved (=falcate; ie., one distal tooth); appendage not canaliculated; appendage without hoods or guards; joint, appearance distinctly asymmetrical (=heterogomph); joint effected by ligament(s); shaft tapering slightly or evenly thick from emergence to joint; shaft, internally chambered, with camerated core. Compound chaetae present in most or all chaetigers.

Tube absent.

**Literature used for description**. Glasby CJ, Fauchald K (2003). POLiKEY. An information system for polychaete families and higher taxa. Version 2, 5 June 2003. Australian Biological Resources Study, DCCEEW, Canberra, https:[//www.dcceew.gov.au/;](http://www.dcceew.gov.au/%3B) Watson C (2022) 7.13.3.1 Chrysopetalidae Ehlers, 1864 In: Purschke G, Böggemann, M, Westheide W (Eds) Handbook of Zoology. Annelida. Vol. 4: Pleistoannelida, Errantia II. De Gruyter, Berlin, 228–258; Rouse GW, Pleijel, F, Tilic, E (2022) Annelida. Oxford University Press, Oxford, UK.

# Chrysopetalidae, Calamyzinae Hartmann-Schröder, 1971

**Description**. Body segment number: variable. Body shape elongate, more-or-less equal width over entire length, dorsoventrally flattened or more-or-less cylindrical. Epidermis more-or-less smooth. Dorsal body surface, appearance under compound microscope smooth. Body surface with protective covering absent. Pygidium simple lobe. Pygidial appendages absent or present, one pair of cirri. Discrete head present; complex in shape bearing appendages.

Prostomium rounded to oval, eyes on head absent or present, two pairs; head eyes simple pigmented cups. Prostomial antennae absent (the antennae labelled in Rouse et al. (2022, fig.

14.3g) appear to be tentacular cirri). Palps present; anteroventral; tapering (usually) sensory type; unarticulated. Nuchal organs present; paired low projections from posterolateral prostomium; posterior projections. Peristomium not visible.

Foregut a muscular axial pharynx. Pharynx jaws absent or present, one pair of lateral jaws. Paired jaws fang-like or fang-like, with a basal support (crossed like a pair of scissors in *Ichthyotomus*). Protrusible proboscis present, smooth, distal ring of papillae absent or present.

First segment tentaculate (2 pairs); second segment chaetous (usually lack the ventral components). Tentacular cirri present; 2 pair(s); arise on a single segment; internal aciculae absent. First chaetiger with both notochaetae and neurochaetae; parapodia similar in length or slightly shorter than subsequent parapodia; parapodia more-or-less laterally directed and free from head; chaetae similar in orientation, length and thickness to other chaetae. Parapodia uniramous (some symbionts), or biramous; parapodial lobes prominent. Notopodial lobes represented by at least one chaetal lobe. Neuropodial lobes represented by at least one chaetal lobe. Dorsal cirri present; more-or-less cirriform. Ventral cirri present (may be lacking on anterior chaetigers); cirriform or tapering.

Chaetae first appear on second segment after peristomium, arranged in paired bundles (or rows) of many chaetae (notochaetae may be absent in symbiont forms). Aciculae present; in both dorsal and ventral positions (=noto- and neuroaciculae) (notoaciculae need confirmation). Neuroaciculae distally tapering. Capillary chaetae absent. Paleate chaetae absent. Spines present; with small teeth or spinelets; present in most or all chaetigers; in dorsal (notopodial) position only. Hooks absent, or present (In bivalve symbiont forms only); without distal hood, beard or ligament; occur in ventral (neuropodial) position; occur over entire body. Compound chaetae present; in dorsal (notopodial) position (may be absent), or in ventral (neuropodial) position; appendage distally tapering to slender tips (=spinigerous), or distally curved (=falcate; ie., one distal tooth), or multidentate; appendage not canaliculated; appendage without hoods or guards; joint effected by ligament(s); shaft tapering slightly or evenly thick from emergence to joint; shaft, internally solid, without distinct core. Compound chaetae present in most or all chaetigers.

Tube absent.

**Literature used for description**. Watson C (2022) 7.13.3.1 Chrysopetalidae Ehlers, 1864 In: Purschke G, Böggemann, M, Westheide W (Eds) Handbook of Zoology. Annelida. Vol. 4: Pleistoannelida, Errantia II. De Gruyter, Berlin, 228–258; Rizzo AE, Magalhães WF (2022) 7.13.5 Ichthyotomidae Eisig, 1906 In: Purschke G, Böggemann, M, Westheide W (Eds) Handbook of Zoology. Annelida. Vol. 4: Pleistoannelida, Errantia II. De Gruyter, Berlin, 366–368; Rouse GW, Pleijel, F, Tilic, E (2022) Annelida. Oxford University Press, Oxford, UK.

# Chrysopetalidae, Chrysopetalinae Ehlers, 1864

**Description**. Body segment number: variable. Body shape elongate, more-or-less equal width over entire length, dorsoventrally flattened or more-or-less cylindrical. Body pigmentation absent or present, gold colouration of dorsal surface due to reflective paleae; epidermis more-or-less smooth. Dorsal body surface, appearance under compound microscope smooth. Body surface with protective covering as shield-like spines (paleae), dorsally. Pygidium simple lobe. Pygidial appendages absent or present, one pair of cirri or one pair of cirri and single medial papilla. Discrete head present; complex in shape bearing appendages.

Prostomium rounded to oval, eyes on head absent or present, one pair or two pairs; head eyes compound, with lenses. Prostomial antennae present; median and paired laterals; unarticulated. Palps present; anteroventral; tapering (usually) sensory type; biarticulated. Nuchal organs present; unpaired caruncle or nuchal fold. Peristomium not visible.

Foregut a muscular axial pharynx. Pharynx jaws present, one pair of lateral jaws. Paired jaws fang-like (stylet-type jaws). Protrusible proboscis present, smooth, distal ring of papillae absent or present.

First segment tentaculate (2 pairs); second segment chaetous (usually lack the ventral components). Tentacular cirri present; 2 pair(s); arise on a single segment; internal aciculae absent. First chaetiger with notochaetae only; parapodia similar in length or slightly shorter than subsequent parapodia; parapodia more-or-less laterally directed and free from head; chaetae similar in orientation, length and thickness to other chaetae. Parapodia biramous; parapodial lobes prominent. Notopodial lobes represented by at least one chaetal lobe. Neuropodial lobes represented by at least one chaetal lobe. Dorsal cirri present; more-or-less cirriform. Ventral cirri present (may be lacking on anterior chaetigers); cirriform or tapering.

Chaetae first appear on second segment after peristomium, arranged in paired bundles (or rows) of many chaetae (notochaetae are arranged in rows). Aciculae present; in both dorsal and ventral positions (=noto- and neuroaciculae) (notoaciculae need confirmation). Neuroaciculae distally tapering. Paleate chaetae present, associated with notopodia. Parapodial paleae arranged in linear fans across dorsum (a few emerge laterally like typical chaetae). Spines present; with small teeth or spinelets; present in most or all chaetigers; in both dorsal and ventral positions (but greatly outnumbered by other types of chaetae). Hooks absent. Compound chaetae present; in ventral (neuropodial) position; appendage distally tapering to slender tips (=spinigerous), or distally curved (=falcate; ie., one distal tooth); appendage not canaliculated; appendage without hoods or guards; joint, appearance distinctly asymmetrical (=heterogomph); joint effected by ligament(s); shaft tapering slightly or evenly thick from emergence to joint; shaft, internally chambered, with camerated core. Compound chaetae present in most or all chaetigers.

Tube absent.

**Literature used for description**. Watson C (2022) 7.13.3.1 Chrysopetalidae Ehlers, 1864 In: Purschke G, Böggemann, M, Westheide W (Eds) Handbook of Zoology. Annelida. Vol. 4: Pleistoannelida, Errantia II. De Gruyter, Berlin, 228–258; Rouse GW, Pleijel, F, Tilic, E (2022) Annelida. Oxford University Press, Oxford, UK.

# Chrysopetalidae, Dysponetinae Aguado, Nygren & Rouse, 2013

**Description**. Body segment number: variable. Body shape elongate, more-or-less equal width over entire length or ovate to elliptical, dorsoventrally flattened or more-or-less cylindrical. Epidermis more-or-less smooth. Dorsal body surface, appearance under compound microscope smooth. Body surface with protective covering as shield-like spines (paleae), dorsally. Pygidium simple lobe. Pygidial appendages absent or present, one pair of cirri or one pair of cirri and single medial papilla. Discrete head present; complex in shape bearing appendages.

Prostomium rounded to oval, eyes on head absent or present, one pair or two pairs; head eyes compound, with lenses. Prostomial antennae present; median and paired laterals; unarticulated. Palps present; anteroventral; tapering (usually) sensory type; biarticulated. Nuchal organs present; paired low projections from posterolateral prostomium; indistinct dorsolateral ciliated patches. Peristomium not visible.

Foregut a muscular axial pharynx. Pharynx jaws present, one pair of lateral jaws. Paired jaws fang-like (stylet-type jaws). Protrusible proboscis present, smooth, distal ring of papillae present.

First segment tentaculate (2 pairs; ventral pair has been referred to as buccal cirri); second segment chaetous (usually lack the ventral components). Tentacular cirri present; 2 pair(s); arise on a single segment; internal aciculae absent. First chaetiger with notochaetae only; parapodia similar in length or slightly shorter than subsequent parapodia; parapodia more-or-less laterally directed and free from head; chaetae similar in orientation, length and thickness to other chaetae. Parapodia biramous; parapodial lobes prominent. Notopodial lobes represented by at least one chaetal lobe. Neuropodial lobes represented by at least one chaetal lobe. Dorsal cirri present; more-or-less cirriform. Ventral cirri present (may be lacking on anterior chaetigers); cirriform or tapering.

Chaetae first appear on second segment after peristomium, arranged in paired bundles (or rows) of many chaetae. Aciculae present; in both dorsal and ventral positions (=noto- and neuroaciculae) (notoaciculae need confirmation). Neuroaciculae distally tapering. Paleate chaetae absent (rarely present). Spines present; with small teeth or spinelets; present in most or all chaetigers; in dorsal (notopodial) position only. Hooks absent. Compound chaetae present; in dorsal (notopodial) position, or in ventral (neuropodial) position; appendage distally tapering to slender tips (=spinigerous), or distally curved (=falcate; ie., one distal tooth); appendage not canaliculated; appendage without hoods or guards; joint, appearance distinctly asymmetrical (=heterogomph); joint effected by ligament(s); shaft tapering slightly or evenly thick from emergence to joint; shaft, internally chambered, with camerated core. Compound chaetae present in most or all chaetigers.

Tube absent.

**Literature used for description**. Watson C (2022) 7.13.3.1 Chrysopetalidae Ehlers, 1864 In: Purschke G, Böggemann, M, Westheide W (Eds) Handbook of Zoology. Annelida. Vol. 4: Pleistoannelida, Errantia II. De Gruyter, Berlin, 228–258; Rouse GW, Pleijel, F, Tilic, E (2022) Annelida. Oxford University Press, Oxford, UK.

# Cirratulidae Ryckholt, 1851

**Description**. Body segment number: variable. Body shape elongate, more-or-less equal width over entire length. Body pigmentation absent or present, includes forms that are dark green, black, red-orange or yellow; epidermis more-or-less smooth. Bioluminescence absent, or present (in benthic species). Pygidium simple lobe, or bilobed (some *Caulleriella*). Pygidial appendages present or absent. Discrete head present; lobe-like without appendages.

Prostomium conical, tapering to slender tip or bluntly conical, eyes on head absent or present, numerous, unpaired; head eyes simple pigmented cups. Palps present (either paired palps or multiple palps, the latter which appear to derive from the first few segments behind head); anterodorsal; grooved (usually) feeding type; feeding palp longitudinally grooved. Nuchal organs present; paired low projections from posterolateral prostomium; indistinct dorsolateral ciliated patches. Peristomium a single ring or one or more achaetous segments may resemble a double peristomium.

Gut more-or-less straight, lacking side branches. Foregut a muscular ventral pharynx.

Pharynx dorsolateral ciliated folds present. Heart bodies present (at least in some taxa).

First segment chaetous, or achaetous (uncertain); second segment chaetous, or achaetous (uncertain). First chaetiger with both notochaetae and neurochaetae; parapodia similar in length or slightly shorter than subsequent parapodia; parapodia more-or-less laterally directed and free from head; chaetae similar in orientation, length and thickness to other chaetae. Parapodia biramous; parapodial lobes absent or very low. Notopodial lobes represented by at least one chaetal lobe. Neuropodial lobes represented by at least one chaetal lobe. Branchiae present (may resemble palps but usually reddish and thinner), arise from lateral body or arise from dorsal body, occur on mid-body segments or occur near posterior end; lateral branchiae digitiform. Dorsal branchiae simple filaments each arising directly from body wall.

Chaetae first appear on second segment after peristomium (usually), arranged in paired bundles (or rows) of many chaetae. Aciculae absent. Capillary chaetae present, in dorsal (notopodial) position or in ventral (neuropodial) position, distally tapered to a point, edge smooth or hirsute-serrate. Capillary chaetae, externally not pseudo-segmented. Capillary chaetae, internally not chambered or hollow. Spines absent, or present (also referred to as hooks); slightly curved and more-or-less smooth (may be distally bifid or knobbed); present in most or all chaetigers, or present only in posterior chaetigers; in both dorsal and ventral positions. Hooks absent.

Segmental organs are metanephridia; metanephridia single anterior pair of excretory metanephridia and several more posterior ones for gamete release (excretory ones on segment 1); nephridial pores, several pairs in anterior body.

Tube absent, or present; membranous (rarely).

**Literature used for description**. Glasby CJ, Fauchald K (2003). POLiKEY. An information system for polychaete families and higher taxa. Version 2, 5 June 2003. Australian Biological Resources Study, DCCEEW, Canberra, https:[//www.dcceew.gov.au/;](http://www.dcceew.gov.au/%3B) Blake JA, Magalhães W (2019) 7.3.1.5 Cirratulidae Ryckholt, 1851 In: Purschke G, Böggemann M, Westheide W (Eds) Handbook of Zoology, Annelida, Volume 1: Annelida Basal Groups and Pleistoannelida, Sedentaria I. De Gruyter, Berlin, 339–397; Rouse GW, Pleijel, F, Tilic E (2022) Annelida. Oxford University Press, Oxford, UK.

# Cossuridae Day, 1963

**Description**. Body segment number: variable. Body shape elongate, more-or-less equal width over entire length. Epidermis more-or-less smooth. Pygidium simple lobe. Pygidial appendages present, three cirri. Discrete head present; lobe-like without appendages.

Prostomium bluntly conical. Buccal tentacles present (though often retracted and not visible in fixed specimens); arising inside mouth (can be exposed by retraction of lower lip); ciliated. Nuchal organs present; paired low projections from posterolateral prostomium; indistinct dorsolateral ciliated patches. Peristomium a single ring, may appear double, but anterior ring is part of the prostomium.

Gut more-or-less straight, lacking side branches. Foregut without a distinct ventral or axial pharynx. Protrusible proboscis absent (tentacles exposed by lower lip retraction). Heart bodies present.

First segment chaetous. First chaetiger with notochaetae only (usually); parapodia similar in length or slightly shorter than subsequent parapodia; parapodia more-or-less laterally directed and free from head; chaetae similar in orientation, length and thickness to other chaetae. Parapodia biramous (rami very close together); parapodial lobes absent or very low. Notopodial lobes represented by at least one chaetal lobe. Neuropodial lobes represented by at least one chaetal lobe. Branchiae present (single long filament), arise from dorsal body (in middle of single anterior chaetiger), occur first few segments. Dorsal branchiae simple filaments each arising directly from body wall.

Chaetae first appear on first segment after peristomium, arranged in paired bundles (or rows) of many chaetae. Aciculae absent. Capillary chaetae present, in dorsal (notopodial) position or in ventral (neuropodial) position, distally tapered to a point, edge smooth or hirsute-serrate. Capillary chaetae, externally not pseudo-segmented. Capillary chaetae, internally not chambered or hollow. Spines absent, or present; slightly curved and more-or-less smooth; present in most or all chaetigers (though more usually in posterior ones); in both dorsal and ventral positions. Hooks absent.

Tube absent.

**Literature used for description**. Glasby CJ, Fauchald K (2003). POLiKEY. An information system for polychaete families and higher taxa. Version 2, 5 June 2003. Australian Biological Resources Study, DCCEEW, Canberra, https:[//www.dcceew.gov.au/;](http://www.dcceew.gov.au/%3B) Ebbe B, Blake JA (2019)

7.3.1.2 Cossuridae Day, 1963 In Purschke G, Böggemann M, Westheide W (Eds), Handbook of Zoology, Annelida, Volume 1: Annelida Basal Groups and Pleistoannelida, Sedentaria I. De Gruyter, Berlin, 309–316; Rouse GW, Pleijel, F, Tilic E (2022) Annelida. Oxford University Press, Oxford, UK.

# Criodrilidae Vejdovskì, 1884

**Description**. Body segment number: variable. Body shape elongate, more-or-less equal width over entire length, more-or-less cylindrical (approaching quadrangular). Dorsal intersegmental furrow present (?), pores on mid-dorsal line present (?). Pygidium simple lobe. Pygidial appendages absent.

Prostomium bluntly conical.

Foregut a muscular dorsal pharynx. Gizzard absent. Calciferous glands absent. Intestinal typhlosole present. Heart bodies present, segments VII-XI.

Chaetae first appear on first segment after peristomium (=S2 for oligochaete workers), in lumbricine arrangement - paired upper and lower bundles of 2 per bundle in - closely spaced lateral and ventrolateral pairs. Crotchet chaetae present, simple-pointed. Genital chaetae present (spermathecal chaetal on XIV).

Sexual reproduction hermaphroditic. Clitellum with inconspicuous borders, thick, multilayered, fully encircles body, in region of male pores (covering male pores at least with anterior edge) and female pores (covering females pores at least with anterior edge). Clitellum occurs from XIV-XVI to XLV-XLVII. Tubercula pubertatis absent. Gonadal segments bearing genital papillae present. Gonadal segments with extensions of the body wall absent. Testes present, two pairs in total, one pair per segment; present in segments X and XI. Sperm sac absent. Ovaries present; present in segments XIII or XIV. Segmental organs are metanephridia; both nephridial pores and gonoducts located around clitellum. Spermathecae absent. Female gonoduct present. Female pores one pair, in segment XIV-XV (intersegmental). Male gonoducts present. Male pores one pair (atop glandular poropores), in segment XIV-XVI, opisthoporous (two or more segments following testicular segment). Prostate gland absent or present; prostate pore and male pore on segment XVII united, discharge through single pore.

**Literature used for description**. Jamieson, BGM (2006) Non-leech Clitellata (with contributions by Marco Ferraguti). Pp. 235–392. In Reproductive Biology and Phylogeny of Annelida. Series Editor BGM Jamieson. Volume 4. Editors G Rouse, F Pleijel. Science Publishers, Enfield, New Hampshire; Blakemore RJ (2008) Review of Criodrilidae (Annelida: Oligochaeta) including *Biwadrilus* from Japan. Opuscula Zoologica Budapest 37: 11–22; Timm, T (2009) A guide to the freshwater Oligochaeta and Polychaeta of Northern and Central Europe. Lauterbornia 66: 1–235; Rota E, de Jong Y (2015) Fauna Europaea: Annelida - Terrestrial Oligochaeta (Enchytraeidae and Megadrili), Aphanoneura and Polychaeta. Biodiversity Data Journal 3: e5737. doi: 10.3897/BDJ.3.e5737; Thorp JH, Lovell LL, Timm T, Martin P, Gelder SR, Govedich FR, Moser WE, Nakano T, Bielecki A, Bain BA, Utevsky S, Gil J, Glasby CJ, Martin D (2019) Phylum Annelida. In: Rogers DC, Thorp JH (Eds) Keys to Palaearctic Fauna: Thorp and Covich’s Freshwater invertebrates - Volume IV. Academic Press, Elsevier, pp. 357–518; Misirlioðlu, M, Reynolds, JW, Stojanoviæ, M, Trakiæ, T, Sekuliæ, J, James, SW, Csuzdi, C, Decaëns, T, Lapied, E, Phillips, HRP, Cameron, EK, Brown, GG (2023). Earthworms (Clitellata, Megadrili) of the world: an updated checklist of valid species and families, with notes on their distribution. Zootaxa 5255 (1): 417–438.

# Ctenodrilidae Kennel, 1882

**Description**. Body segment number: variable. Body shape elongate, more-or-less equal width over entire length. Epidermis more-or-less smooth. Pygidium simple lobe. Discrete head present; lobe-like without appendages.

Prostomium bluntly conical. Nuchal organs present; paired low projections from posterolateral prostomium; indistinct dorsolateral ciliated patches. Peristomium a single ring.

Gut more-or-less straight, lacking side branches. Foregut a muscular ventral pharynx.

First segment chetous, or achaetous (uncertain); second segment chaetous. First chaetiger with both notochaetae and neurochaetae; parapodia similar in length or slightly shorter than subsequent parapodia; parapodia more-or-less laterally directed and free from head; chaetae similar in orientation, length and thickness to other chaetae. Parapodia present. Parapodia biramous; parapodial lobes absent or very low. Notopodial lobes represented by at least one chaetal lobe. Neuropodial lobes represented by at least one chaetal lobe. Branchiae absent or present.

Chaetae first appear on second segment after peristomium (uncertain), arranged in paired bundles (or rows) of many chaetae. Aciculae absent. Capillary chaetae present, in both dorsal and ventral positions, distally tapered to a point, edge smooth. Capillary chaetae, externally not pseudo-segmented. Capillary chaetae, internally not chambered or hollow. Spines present (includes multidentate types, which are also referred to as hooks); with small teeth or spinelets (referred to as pectinate), or sharply bent (=geniculate) or recurved; present in most or all chaetigers, or present only in posterior chaetigers; in both dorsal and ventral positions. Hooks absent.

Segmental organs are metanephridia; metanephridia single anterior pair of excretory metanephridia and several more posterior ones for gamete release (excretory ones on segment 1); nephridial pores located anteriorly, gonoducts located posteriorly.

Tube absent.

**Literature used for description**. Glasby CJ, Fauchald K (2003). POLiKEY. An information system for polychaete families and higher taxa. Version 2, 5 June 2003. Australian Biological Resources Study, DCCEEW, Canberra, https:[//www.dcceew.gov.au/;](http://www.dcceew.gov.au/%3B) Dean HK, Blake JA (2019)

7.3.1.4 Ctenodrilidae Kennel, 1882 In Purschke G, Böggemann M, Westheide W (Eds), Handbook of Zoology, Annelida, Volume 1: Annelida Basal Groups and Pleistoannelida, Sedentaria I. De Gruyter, Berlin, 328–338; Rouse GW, Pleijel, F, Tilic E (2022) Annelida. Oxford University Press, Oxford, UK.

# Cyclobdellidae Ringuelet, 1972a

**Description**. Body segment number: fixed, 34 segments including 2 preoral 'segments' (prostomium and peristomium) and 32 postoral segments. Secondary annulation present (disk-shaped receptors called sensillae mark the middle annulus of each segment); anterior region uniannulate, or biannulate; mid-body 5-annulate; posterior biannulate. Body shape elongate, more-or-less equal width over entire length (when relaxed; but can be dramatically shortened by contraction of longitudinal muscles during movement), dorsoventrally flattened. Anterior end sucker on ventral surface present; not clearly separated from rest of body. Large mouth on ventral surface of sucker. Posterior sucker on ventral surface present; anus positioned near posterior end (dorsally, just before posterior sucker).

Eyes on head present, five pairs (on segments II-V and VII); head eyes simple pigmented cups.

Foregut a muscular axial pharynx; muscular axial pharynx not rotated (euthylaematous). Pharynx jaws present, leech-type (either soft-muscular or horny); two (duognathous) muscular jaws, or three (trignathous) muscular jaws arranged in a triangle; one row of teeth. Protrusible proboscis absent. Crop present. Circulatory system absent.

Sexual reproduction hermaphroditic. Clitellum fully encircles body. Testes present, many pairs. Ovaries present, one pair. Egg sacs present; tubular. Oviduct separate one for each egg sac. Vaginal sac absent. Female gonoduct present, vaginal sac absent. Female pores single, median. Male gonoducts present. Male pores single, median.

**Literature used for description**. Ringuelet RA (1972a) Nuevos taxia de Hirudineos neotropicos con la redefinicion de Semiscolecidae y la descripcion de Cyclobdellidae fam. nov. y Mesobdellidae fam. nov. Physis 31 (82): 193–201; Christofferson ML (2008) A Catalogue of the Piscicolidae, Ozobranchidae, and Arhynchobdellida (Annelida, Clitellata, Hirudinea) from South America. Neotropical Biology and Conservation 3(1):39–48.

# Cylicobdellidae Ringuelet, 1972b

**Description**. Body segment number: fixed, 34 segments including 2 preoral 'segments' (prostomium and peristomium) and 32 postoral segments. Secondary annulation present (disk-shaped receptors called sensillae mark the middle annulus of each segment); mid-body 5-annulate. Body shape elongate, more-or-less equal width over entire length (when relaxed; but can be dramatically shortened by contraction of longitudinal muscles during movement). Anterior end sucker on ventral surface present; not clearly separated from rest of body. Large mouth on ventral surface of sucker (post-buccal rim present). Body pigmentation present, a vivid carmine-maroon reddish colour; epidermis papillate. Posterior sucker on ventral surface present; rays absent; anus positioned near posterior end (dorsally, just before posterior sucker).

Eyes on head absent. Postcephalic eyes absent. Eyes on trunk absent. Caudal eyes absent.

Gut more-or-less straight, lacking side branches or straight with side branches. Foregut a muscular axial pharynx; muscular axial pharynx ridges of pharynx rotated 60 degrees to the right (strepsilaematous); extends from long. Pharynx jaws present, leech-type (either soft-muscular or horny) (as soft muscular 'jaws'), denticles absent (pseudognaths absent). Protrusible proboscis absent. Crop present. Caeca of midgut (=posterior crop caeca) absent (occasionally with an unpaired caecum in XIX). Circulatory system absent.

Sexual reproduction hermaphroditic. Clitellum fully encircles body, in region of male and female pores. Clitellum occurs from X-XIII (usual position for leeches). Testes present, many pairs (9–12 pairs in total, sometimes missing on one side), one pair per segment; present in segments XX or XI (check Siddall et al 2006 say IX-XII). Sperm sac present. Ovaries present, one pair; present in segment XII. Egg sacs present (tubular, very long); tubular. Oviduct single, shared oviduct from egg sac. Vaginal sac absent. Segmental organs are metanephridia; metanephridia ventral. Female gonoduct present, vaginal sac absent. Female pores single, median, in segment XII. Male gonoducts present, with atrium present, bilobed (anterorly directed, deeply cleft with cornua). Male pores single, median, in segment XI. Penis absent. Prostate gland absent.

**Literature used for description**. Ringuelet RA (1972b) Cylicobdellidae, a new family of erpopdelloid Hirudinea. Physis 31 (83): 337–344; Ringuelet RA (1972a) Nuevos taxia de Hirudineos neotropicos con la redefinicion de Semiscolecidae y la descripcion de Cyclobdellidae fam. nov. y Mesobdellidae fam. nov. Physis 31 (82): 193–201; Siddall ME, Bely AE, Borda E (2006) Hirudinida Pp. 393–429. In Reproductive Biology and Phylogeny of Annelida. Series Editor BGM Jamieson. Volume 4. Editors G Rouse, F Pleijel. Science Publishers, Enfield, New Hampshire; Christofferson ML (2008) A Catalogue of the Piscicolidae, Ozobranchidae, and Arhynchobdellida (Annelida, Clitellata, Hirudinea) from South America. Neotropical Biology and Conservation 3(1):39–48.

# Dinophilidae Macalister, 1876

**Description**. Body segment number: fixed, segmentation present (6 poorly delineated segments have been reported), less than 14 segments. Secondary annulation present (posteriorly). Body shape sausage or grub-shaped (also referred to as cigar shaped). Body translucent, gut visible, pigmentation absent or present, pigmentation sometimes bright orange; epidermis more-or-less smooth. Dorsal body surface, appearance under compound microscope ciliated (transverse bands of cilia on body (usually two rows per segment dorsally); ciliary rings on head). Pygidium simple lobe. Pygidial appendages absent (although a tail-like extension is present in *Dimorphilus*). Discrete head present; lobe-like without appendages (bearing compound cilia - called 'anterior sensory cilia' in Annelida Glossary, [doi.org/10.5281/zenodo.14848165](https://doi.org/10.5281/zenodo.14848165), as well as normal ciliary bands).

Prostomium rounded to oval, eyes on head absent or present, one pair; head eyes simple pigmented cups (cup-type). Nuchal organs present; paired low projections from posterolateral prostomium; indistinct dorsolateral ciliated patches. Peristomium not visible.

Gut absent (in dwarf males of some *Dinophilus* species) or present, more-or-less straight, lacking side branches. Foregut a muscular axial pharynx. Circulatory system absent.

First segment achaetous; second segment achaetous. Parapodia absent. Chaetae absent.

Sexual reproduction gonochoric (copulation). Testes present (unpaired). Ovaries present (1–4). Egg sacs absent. Segmental organs are protonephridia. Female gonoduct present. Male gonoducts present (together with paired spermioducts and 1–2 pairs of seminal vesicles). Penis present (unpaired).

Tube absent.

**Literature used for description**. Glasby CJ, Fauchald K (2003). POLiKEY. An information system for polychaete families and higher taxa. Version 2, 5 June 2003. Australian Biological Resources Study, DCCEEW, Canberra, https:[//www.dcceew.gov.au/](http://www.dcceew.gov.au/) [as Dorvilleidae Group 3]; Rouse G, Pleijel F, Eds (2006) Reproductive Biology and Phylogeny of Annelida, BGM Jamieson (Series Ed), Science Publishers, Enfeld, New Hampshire, USA, 688 pp.; Westheide W (2019) 7.1.1 Dinophilidae Verrill, 1892 In Purschke G, Böggemann M, Westheide W (Eds), Handbook of Zoology, Annelida, Volume 1: Annelida Basal Groups and Pleistoannelida, Sedentaria I. De Gruyter, Berlin, 217–233; Worsaae K, Kerbl A., Di Domenico M., Gonzalez BC, Bekkouche N, Martínez A (2021) Interstitial Annelida. Diversity 13: 77. https://doi.org/10.3390/d13020077; Rouse GW, Pleijel, F, Tilic E (2022) Annelida. Oxford University Press, Oxford, U.

# Diurodrilidae Kristensen & Niilonen, 1982

**Description**. Body segmentation absent (5–20 segments have been inferred from presence of indistinct creases in the body wall). Body shape elongate, more-or-less equal width over entire length or sausage or grub-shaped. Body translucent, gut visible; epidermis more-or-less smooth. Dorsal body surface, appearance under compound microscope ciliated (at anterior and posteriorends; cuticularised 'plates' distributed along body in *Diurodrilus*). Pygidium simple lobe (sometimes), or with multiple digitate lobes (2–4 adhesive structure resembling toes). Pygidial appendages absent. Discrete head present; lobe-like without appendages (bearing cilia; stiff or flexible; called 'anterior sensory cilia' in Annelida Glossary, [doi.org/10.5281/zenodo.14848165](https://doi.org/10.5281/zenodo.14848165)).

Prostomium rounded to oval. Palps absent. Nuchal organs absent. Peristomium a single ring, may appear double.

Gut more-or-less straight, lacking side branches. Foregut a muscular ventral pharynx.

Protrusible proboscis absent. Pharynx dorsolateral ciliated folds present.

Parapodia absent.

Chaetae absent, although similar looking tactile bristles (also called ciliary tufts or 'sensoria') are present.

Sexual reproduction hermaphroditic, or gonochoric (rarely). Testes absent. Ovaries present. Egg sacs absent. Segmental organs are protonephridia (2–3 pairs); paired gonopores posteriorly (may be characteristic of the family). Female gonoduct absent. Male gonoducts present (with short ciliated funnels opening into a cloaca). Male pores one pair.

Tube absent.

**Literature used for description**. Glasby CJ, Fauchald K (2003). POLiKEY. An information system for polychaete families and higher taxa. Version 2, 5 June 2003. Australian Biological Resources Study, DCCEEW, Canberra, https:[//www.dcceew.gov.au/;](http://www.dcceew.gov.au/%3B) Worsaae, K, Kerbl, A., Di Domenico, M., Gonzalez, B.C., Bekkouche, N. & Martínez, A. (2021) Interstitial Annelida. Diversity 2021, 13, 77. https://doi.org/10.3390/d13020077; Rouse G, Pleijel F, Eds (2006) Reproductive Biology and Phylogeny of Annelida, BGM Jamieson (Series Ed), Science Publishers, Enfeld, New Hampshire, USA, 688 pp.; Worsaae K, Kristensen RM (2021) 7.8 Sedentaria incertae sedis: Diurodrilidae Kristensen & Niilonen, 1982 In: Purschke G, Böggemann M, Westheide W (Eds), Handbook of Zoology, Annelida, Volume 3: Pleistoannelida, Sedentaria III and Errantia I. De Gruyter, Berlin, 202–214; Rouse GW, Pleijel, F, Tilic E (2022) Annelida. Oxford University Press, Oxford, UK.

# Dorvilleidae Chamberlin, 1919

**Description**. Body segment number: fixed or variable, less than 14 segments. Body shape elongate, more-or-less equal width over entire length or sausage or grub-shaped. Body translucent, gut visible (small forms) or opaque, gut usually not visible, pigmentation absent or present, pigmentation usually red to brown transverse stripes or base colour; epidermis more-or-less smooth. Dorsal body surface, appearance under compound microscope ciliated. Pygidium simple lobe. Pygidial appendages absent or present, one pair of cirri or one pair of cirri and single medial papilla or four cirri. Discrete head present; complex in shape bearing appendages.

Prostomium rounded to oval, eyes on head absent or present, one pair or two pairs (second pair are minute); head eyes simple pigmented cups. Prostomial antennae absent (rarely), or present; paired, lateral; unarticulated, or articulated. Palps absent (rarely), or present; anteroventral; tapering (usually) sensory type; unarticulated, or biarticulated. Nuchal organs present; paired low projections from posterolateral prostomium; indistinct dorsolateral ciliated patches. Peristomium a double ring.

Gut more-or-less straight, lacking side branches. Foregut a muscular ventral pharynx. Pharynx jaws absent (rarely) or present, multiple jaw elements of different shapes and sizes. Multiple jaws comprising ventral mandibles and dorsal maxillae, 2 pairs of toothed plates plus many small free denticles arranged in longitudinal rows. Pharynx maxillae symmetrical. Superior row more than 8 pairs or fewer than 8 pairs (rarely). Superior base plates present, fused to each other posteriorly or present, free of each other or absent. Superior base plates fused with maxillary carrier, K-shaped (rarely) or fused with maxillary carrier, not K-shaped or not fused with maxillary carrier. Maxillary carriers shorter than combined length of maxillae. Pharynx dorsolateral ciliated folds present.

First segment chaetous (usually); second segment chaetous (usually). First chaetiger with neurochaetae only; parapodia similar in length or slightly shorter than subsequent parapodia; parapodia more-or-less laterally directed and free from head; chaetae similar in orientation, length and thickness to other chaetae. Parapodia present. Parapodia uniramous. Notopodial lobes represented by at least one chaetal lobe and absent (uncertainty over whether there is a simple notopodial lobe or a dorsal cirrus bearing an acicula). Neuropodial lobes represented by at least one chaetal lobe. Dorsal cirri absent (rarely), or present; more-or-less cirriform. Ventral cirri absent (rarely), or present; cirriform or tapering. Branchiae absent or present, arise from lateral body or arise from dorsal body, occur on mid-body segments; lateral branchiae digitiform. Dorsal branchiae branching, or simple filaments each arising directly from body wall.

Chaetae present (rarely absent), first appear on first segment after peristomium, arranged as a single bundle (or row) of many chaetae (lower bundle - neurochaetae - only). Aciculae present; in ventral position (=neuroaciculae), or in both dorsal and ventral positions (=noto- and neuroaciculae). Neuroaciculae distally tapering. Capillary chaetae present, in ventral (neuropodial) position, distally tapered to a point, edge smooth or hirsute-serrate or spinose. Capillary chaetae, externally not pseudo-segmented. Capillary chaetae, internally not chambered or hollow. Forked chaetae absent, or present, furcate, tines more-or-less equal in length, or tines distinctly unequal in length. Spines absent, or present (rarely); slightly curved and more-or-less smooth; present in most or all chaetigers; in ventral (neuropodial) position only. Hooks absent. Compound chaetae absent (rarely), or present; in ventral (neuropodial) position; appendage distally tapering to slender tips (=spinigerous), or distally curved (=falcate; ie., one distal tooth); appendage not canaliculated; appendage with paired guards on each side of the crest; joint, appearance distinctly asymmetrical (=heterogomph); joint effected by ligament(s); shaft tapering slightly or evenly thick from emergence to joint; shaft, internally solid, without distinct core. Compound chaetae present in most or all chaetigers.

Segmental organs are protonephridia, or metanephridia; present in most segments of the body.

Tube absent.

**Literature used for description**. Glasby CJ, Fauchald K (2003). POLiKEY. An information system for polychaete families and higher taxa. Version 2, 5 June 2003. Australian Biological Resources Study, DCCEEW, Canberra, https:[//www.dcceew.gov.au/](http://www.dcceew.gov.au/) [as Dorvilleidae Group 1]; Wiklund H, Purschke G, Ravara A (2021) 7.12.2 Dorvilleidae Chamberlin, 1919 In: Purschke G, Böggemann M, Westheide W (Eds), Handbook of Zoology, Annelida, Volume 3: Pleistoannelida, Sedentaria III and Errantia I. De Gruyter, Berlin, 361–382; Rouse GW, Pleijel, F, Tilic E (2022) Annelida. Oxford University Press, Oxford, UK.

# Dorydrilidae Cook, 1968

**Description**. Body segment number: variable. Secondary annulation absent or present; anterior region biannulate. Body shape elongate, more-or-less equal width over entire length. Pygidium simple lobe. Pygidial appendages absent.

Prostomium bluntly conical. Foregut a muscular dorsal pharynx.

First segment chaetous.

Chaetae two per bundle, first appear on first segment after peristomium, in lumbricine arrangement - paired upper and lower bundles in closely spaced lateral and ventrolateral pairs. Capillary (=hair) chaetae absent. Crotchet chaetae present, simple.

Sexual reproduction hermaphroditic. Clitellum thin, single layered, in region of male pores (covering male pores at least with anterior edge), and female pores. Clitellum occurs from X to XI. Testes present, one pair in total; present in segment IX. Ovaries present; present in segment X. Segmental organs are metanephridia; both nephridial pores and gonoducts located around clitellum. Spermathecae present, post-testicular. Spermathecal pores present, located within 1 or 2 segments of male pores (on same segment or subsequent one); 1–2 pairs; in segment X or XI, or both. Female gonoduct present. Female pores one pair, in segment XI. Male gonoducts present. Male pores one pair, in segment X, plesioporous (in segment following testicular segment). Penis present, with hardened sheath present. Prostate gland absent.

**Literature used for description**. Cook DG (1968) The genera of the family Lumbriculidae and the genus *Dorydlilus* (Annelida, Oligochaeta). Journal of the Zoological Society of London 156: 273–289; Brinkhurst RO and Jamieson, BGM (1971) Aquatic Oligochaeta of the World with contributions by DG Cook, DV Anderson, J van der Land, University of Toronto Press, Toronto, Canada, 860 pp; Jamieson, BGM (2006) Non-leech Clitellata (with contributions by Marco Ferraguti). Pp. 235–392. In Reproductive Biology and Phylogeny of Annelida. Series Editor BGM Jamieson. Volume 4. Editors G Rouse, F Pleijel. Science Publishers, Enfield, New Hampshire; Timm, T (2009) A guide to the freshwater Oligochaeta and Polychaeta of Northern and Central Europe. Lauterbornia 66: 1–235.

# Enchytraeidae d'Udekum, 1855

**Description**. Body segment number: variable. Secondary annulation absent or present. Body shape elongate, more-or-less equal width over entire length (small worms). Body translucent, gut visible (usually), pigmentation usually absent (white, yellowish or pinkish) but some are black. Bioluminescence absent, or present (only *Henlea* and *Enchytraeus*). Pygidium simple lobe. Pygidial appendages absent.

Prostomium bluntly conical (bearing a coelomic pore).

Gut more-or-less straight, lacking side branches or straight with side branches. Foregut a muscular dorsal pharynx. Protrusible proboscis absent. Circulatory system present (may be reduced in some taxa).

Chaetae absent (rarely, e.g., *Achaeta*) or present, first appear on first segment after peristomium (dorsal and ventral bundles), in lumbricine arrangement - paired upper and lower bundles of one or a few chaetae each or in paired bundles (or rows) of many chaetae - widely spaced lateral and ventrolateral pairs. Capillary (=hair) chaetae absent. Crotchet chaetae present, almost always simple-pointed and mostly lacking nodulus; rarely bifid in which case upper tooth shorter). Genital chaetae absent.

Sexual or asexual (fragmentation) reproduction. Sexual reproduction hermaphroditic. Clitellum thin, single layered, fully encircles body, in region of male pores (male pores on first clitellum segment), and female pores. Clitellum occurs from XI-XII, rarely to XIII. Testes present, one pair in total; present in segment XI (usually); rarely shifted forward. Ovaries present; present in segment XII, rarely shifted forward. Segmental organs are metanephridia; both nephridial pores and gonoducts located around clitellum, several pairs in anterior body, one pair nephridia in each segment (holonephridia). Spermathecae present, pre-testicular. Spermathecal pores present, located well anterior to male pores; 1 pair (usually); in segment V. Female gonoduct present. Female pores one pair, in segment XIII. Male gonoducts present, with atrium present. Male pores one pair, in segment XII (rarely before), plesioporous (in segment following testicular segment). Penis absent. Prostate gland absent (usually).

**Literature used for description**. Brinkhurst RO and Jamieson, BGM (1971) Aquatic Oligochaeta of the World with contributions by DG Cook, DV Anderson, J van der Land, University of Toronto Press, Toronto, Canada, 860 pp; Blakemore, RJ (2006). Introductory Key of the revised families of earthworms of the World. Unpub. March 2006; Jamieson, BGM (2006) Non-leech Clitellata (with contributions by Marco Ferraguti). Pp. 235–392. In Reproductive Biology and Phylogeny of Annelida. Series Editor BGM Jamieson. Volume 4. Editors G Rouse, F Pleijel. Science Publishers, Enfield, New Hampshire; Martin, P, Martinez-Ansemil, E., Pinder, A, Timm, T, Wetzel, MJ (2008) Global diversity of oligochaetous clitellates (‘‘Oligochaeta’’; Clitellata) in freshwater. Hydrobiologia (2008) 595:117–127; Timm, T (2009) A guide to the freshwater Oligochaeta and Polychaeta of Northern and Central Europe. Lauterbornia 66: 1–235; Erséus, C, Rota, E, Matamoros, L, De Wit, P (2010). Molecular phylogeny of Enchytraeidae (Annelida, Clitellata). Molecular Phylogenetics and Evolution 57: 849–858; Schmelz R, Collado, R (2010). A guide to European terrestrial and freshwater species of Enchytraeidae (Oligochaeta). Soil Organisms 82: 1–176; Rota E, de Jong Y (2015) Fauna Europaea: Annelida - Terrestrial Oligochaeta (Enchytraeidae and Megadrili), Aphanoneura and Polychaeta. Biodiversity Data Journal 3: e5737. doi: 10.3897/BDJ.3.e5737; Thorp JH, Lovell LL, Timm T, Martin P, Gelder SR, Govedich FR, Moser WE, Nakano T, Bielecki A, Bain BA, Utevsky S, Gil J, Glasby CJ, Martin D (2019) Phylum Annelida. In: Rogers DC, Thorp JH (Eds) Keys to Palaearctic Fauna: Thorp and Covich’s Freshwater invertebrates - Volume IV. Academic Press, Elsevier, pp. 357–518.

# Erpobdellidae Blanchard, 1894

**Description**. Body segment number: fixed, 34 segments including 2 preoral 'segments' (prostomium and peristomium) and 32 postoral segments (true segments generally obscured by intervening superficial annulations (3–16), giving the impression of many more segments). Secondary annulation present (disk-shaped receptors called sensillae mark the middle annulus of each segment); mid-body 5-annulate (typically), or 8-annulate, or more (rarely). Body shape elongate, more-or-less equal width over entire length (when relaxed; but can be dramatically shortened by contraction of longitudinal muscles during movement), dorsoventrally flattened. Anterior end sucker on ventral surface present (surrounding mouth); not clearly separated from rest of body. Large mouth on ventral surface of sucker. Body pigmentation absent or present, dark green to black base colour, sometimes with transverse yellow spots. Posterior sucker on ventral surface present; circular; rays absent, or present; anus positioned near posterior end (dorsally, just before posterior sucker).

Eyes on head present (rarely absent according to Sawyer 1986), four or five pairs (5 pairs on segments II-VI or four pairs over two segments; rarely 6 pairs) ); head eyes simple pigmented cups.

Gut more-or-less straight, lacking side branches or straight with side branches. Foregut a muscular axial pharynx; muscular axial pharynx ridges of pharynx rotated 60 degrees to the right (strepsilaematous). Pharynx jaws present, leech-type (either soft-muscular or horny) (as soft 'muscular' jaws); two (duognathous) muscular jaws, or three (trignathous) muscular jaws arranged in a triangle; denticles absent or present (stylets absent). Crop present. Caeca of midgut (=posterior crop caeca) absent, or present (in Nearctic genus *Motobdella*). Circulatory system absent.

Sexual reproduction hermaphroditic. Clitellum fully encircles body, in region of male and female pores. Clitellum occurs from X-XIII (usual position for leeches). Gastropores absent. Testes present, many pairs, in multiple grape-like clusters per segment. Sperm sac present. Ovaries present, one pair. Egg sacs present; tubular (with anterior ovarian branches on each). Oviduct separate one for each egg sac. Segmental organs are metanephridia; nephridial pores single (ventromedial). Female gonoduct present. Female pores single, median, in segment XI (*Trocheta blanchardi*) or XI/XII. Male gonoducts present, with atrium present, variable (cornua directed anteriorly or laterally). Male pores single, median, in segment XI. Penis absent. Prostate gland absent.

**Literature used for description**. Sawyer RT (1986) Leech biology and behaviour. Vol. 1 Anatomy, Physiology, and behaviour. Oxford Science Publications, Oxford UK, 418 pp; Siddall ME, Bely AE, Borda E (2006) Hirudinida Pp. 393–429. In Reproductive Biology and Phylogeny of Annelida. Series Editor BGM Jamieson. Volume 4. Editors G Rouse, F Pleijel. Science Publishers, Enfield, New Hampshire; Christofferson ML (2008) A Catalogue of the Piscicolidae, Ozobranchidae, and Arhynchobdellida (Annelida, Clitellata, Hirudinea) from South America. Neotropical Biology and Conservation 3(1):39–48; Oceguera-Figueroa A, Phillips AJ, Pacheco-Chaves B, Reeves WK, Siddall ME (2011) Phylogeny of macrophagous leeches (Hirudinea, Clitellata) based on molecular data and evaluation of the barcoding locus. Zoologica Scripta 40: 194–203; Brusca, RC, Moore, W & Shuster, SM. (eds) (2016) Chapter 14. Phylum Annelida. In: Invertebrates, 3rd Edition. Sinauer Associates, Sunderland, MA; Thorp JH, Lovell LL, Timm T, Martin P, Gelder SR, Govedich FR, Moser WE, Nakano T, Bielecki A, Bain BA, Utevsky S, Gil J, Glasby CJ, Martin D (2019) Phylum Annelida. In: Rogers DC, Thorp JH (Eds) Keys to Palaearctic Fauna: Thorp and Covich’s Freshwater invertebrates - Volume IV. Academic Press, Elsevier, pp. 357–518; Uttam S, Langer S (2021) Distribution and Identification key for species of freshwater leech genus *Erpobdella* Blainville, 1818 (Hirudinida: Arhynchobdellida: Erpobdelliformes: Erpobdellidae). Ecology, Environment and Conservation 27:1925–1936.

# Eudrilidae Claus, 1880

**Description**. Body segment number: variable. Body shape elongate, more-or-less equal width over entire length. Dorsal intersegmental furrow present (?), pores on mid-dorsal line present (?). Pygidium simple lobe. Pygidial appendages absent.

Prostomium bluntly conical.

Gut straight with side branches. Foregut a muscular dorsal pharynx. Gizzard present.

Calciferous glands present. Heart bodies present.

Chaetae first appear on first segment after peristomium, (=S2 for oligochaete workers), 2 per bundle, in lumbricine arrangement. Crotchet chaetae present, simple-pointed. Genital chaetae present (penial chaetae at entrance of male pores; variety of shapes).

Sexual reproduction hermaphroditic. Clitellum thick, multilayered (often long, extending over many segments or, rarely, forming a closed cavity called a “clitellar pouch”), fully encircles body, anterior to male pores (last segment of clitellum may encompass male pore), in region of female pores. Clitellum occurs from XIII to XVIII. Seminal groove absent, or present (extends from male pore to tip of penis). Tubercula pubertatis present. Testes present, two pairs in total, one pair per segment; present in segments XX or XI. Ovaries present, one pair; present in segment XIII. Egg sacs present. Segmental organs are metanephridia; both nephridial pores and gonoducts located around clitellum, one pair nephridia in each segment (holonephridia). Spermathecae present, post-testicular. Spermathecal pores present (often several pairs; rarely combined with female pores as in *Hyperiodrilus*), unpaired (usually), located within 1 or 2 segments of male pores ; 1 pair; in segment XIV, combined with female pore. Female gonoduct present, vaginal sac present. Female pores one pair (rarely combined with spermathecal pores), in segment XIV, combined with spermathecal pores. Male gonoducts present. Male pores one pair or two pairs (rarely, *Hyperiodrilus* only), in segment XVII or XVIII; in intersegmental furrow; combined with prostatic pore, opisthoporous (two or more segments following testicular segment). Penis present (segment XIV). Prostate gland present (dilated loop of the male duct), tubular (lumen enlarged, acorn-shaped), one pair. Prostate pores in segments XVII and XVIII, anterior to intersegmental furrow, ventral; prostate pore and male pore on segment XVII united, discharge through single pore.

**Literature used for description**. Taylor AG (1949). A West African Earthworm, *Hippopera nigeriae*, belonging to a new Family Hippoperidae. Journal of Zoology 119: 703–710; Sims, RW (1987) A review of the Central African earthworm family Eudrilidae (Oligochaeta). In: Bonvicini Pagliai, AM, Omodeo, P (Eds.), On earthworms. Selected symposia and monographs, UZI, 2. Mucchi, Modena, pp. 359–388; Jamieson, BGM (2006) Non-leech Clitellata (with contributions by Marco Ferraguti). Pp. 235–392. In Reproductive Biology and Phylogeny of Annelida. Series Editor BGM Jamieson. Volume 4. Editors G Rouse, F Pleijel. Science Publishers, Enfield, New Hampshire; Plisko, JD, Nxele, TC (2015). An annotated key separating foreign earthworm species from the indigenous South Africian taxa (Oligochaeta: Acanthodrilidae, Eudrilidae, Glossoscolecidae, Lumbricidae, Megascolecidae, Microchaetidae, Ocnerodrilidae and Tritogeniidae). African Invertebrates 56: 663–708; Thorp JH, Lovell LL, Timm T, Martin P, Gelder SR, Govedich FR, Moser WE, Nakano T, Bielecki A, Bain BA, Utevsky S, Gil J, Glasby CJ, Martin D (2019) Phylum Annelida. In: Rogers DC, Thorp JH (Eds) Keys to Palaearctic Fauna: Thorp and Covich’s Freshwater invertebrates - Volume IV. Academic Press, Elsevier, pp. 357–518; Misirlioðlu, M, Reynolds, JW, Stojanoviæ, M, Trakiæ, T, Sekuliæ, J, James, SW, Csuzdi, C, Decaëns, T, Lapied, E, Phillips, HRP, Cameron, EK, Brown, GG (2023). Earthworms (Clitellata, Megadrili) of the world: an updated checklist of valid species and families, with notes on their distribution. Zootaxa 5255 (1): 417–438.

# Eulepethidae Chamberlin, 1919

**Description**. Body segment number: variable. Body shape elongate, more-or-less equal width over entire length, dorsoventrally flattened. Epidermis more-or-less smooth. Body surface with protective covering as scales (elytrae), dorsally. Pygidium simple lobe. Pygidial appendages present, one pair of cirri. Discrete head present; complex in shape bearing appendages.

Prostomium rounded to oval, eyes on head absent or present, two pairs; head eyes compound, with lenses. Prostomial antennae present; median and paired laterals; consist of basal ceratophore and distal ceratostyle. Palps present; anteroventral; tapering (usually) sensory type; unarticulated. Nuchal organs present; paired low projections from posterolateral prostomium; club-shaped. Peristomium not visible.

Foregut a muscular axial pharynx. Pharynx jaws present, two pairs of jaws. Paired jaws plate-like. Protrusible proboscis present, distal ring of papillae present.

First segment chaetous and tentaculate (elongated dorsal and ventral cirri); second segment chaetous. Tentacular cirri present (dorsal pair bearing large basal tentaculophores); 2 pair(s); arise on a single segment; internal aciculae present in at least some cirri. First chaetiger without external chaetae, or with notochaetae only; parapodia similar in length or slightly shorter than subsequent parapodia; parapodia anteriorly directed and wrapping around head; chaetae similar in orientation, length and thickness to other chaetae. Parapodia biramous; parapodial lobes prominent. Notopodial lobes represented by at least one chaetal lobe. Neuropodial lobes represented by at least one chaetal lobe (very wide, distally truncate with distal edges supported by narrow sclerotinised ridges). Dorsal cirri present (present on non-elytrous segments); more-or-less cirriform. Elytra with papillae, tubercles or smooth (with characteristic marginal papillae and intervening 'elytron notches'). Elytra, post segment 7 occur on every other segment from segment 7 to midbody, then present on every segment, or absent, to end of body. Ventral cirri present; cirriform or tapering. Branchiae absent or present, arise from dorsal body, occur on mid-body segments (non-elytrous segments). Dorsal branchiae simple filaments each arising directly from body wall (dorsal to notopodia).

Chaetae first appear on first segment after peristomium or second segment after peristomium, arranged in paired bundles (or rows) of many chaetae. Aciculae present; in both dorsal and ventral positions (=noto- and neuroaciculae). Neuroaciculae distally axehead-shaped. Capillary chaetae present, in dorsal (notopodial) position or in ventral (neuropodial) position, distally tapered to a point, edge smooth or hirsute-serrate or spinose. Capillary chaetae, externally not pseudo-segmented. Capillary chaetae, internally not chambered or hollow. Spines present; sharply bent (=geniculate) or recurved; present in most or all chaetigers; in dorsal (notopodial) position only. Hooks absent.

Tube absent.

**Literature used for description**. Glasby CJ, Fauchald K (2003). POLiKEY. An information system for polychaete families and higher taxa. Version 2, 5 June 2003. Australian Biological Resources Study, DCCEEW, Canberra, https:[//www.dcceew.gov.au/;](http://www.dcceew.gov.au/%3B) Rouse GW, Pleijel, F, Tilic E (2022) Annelida. Oxford University Press, Oxford, UK.

# Eunicidae Berthold, 1827

**Description**. Body segment number: variable. Body shape elongate, more-or-less equal width over entire length. Body pigmentation absent or present, often an iridescent greenish or reddish base colour; epidermis more-or-less smooth. Pygidium simple lobe. Pygidial appendages present, one pair of cirri or four cirri. Discrete head present; complex in shape bearing appendages.

Prostomium rounded to oval, anteriorly incised (forming two ventrally inflated pad-like lips which have erroneously been called palps in the old literature), eyes on head absent or present, two pairs (anterior pair minute); head eyes simple pigmented cups. Prostomial antennae present; median and paired laterals (rarely lateral ones absent; a second pair of antennae-like structures - referred to as palpal antennae - are also present); articulated (rarely smooth). Palps absent, or present ('palpal antennae' closely resemble the more medial true antennae); anterodorsal; tapering (usually) sensory type; multiple articulations (articulations may be strong or weak; the sensory palps closely resemble the antennae). Nuchal organs present; paired low projections from posterolateral prostomium; indistinct dorsolateral ciliated patches. Peristomium a double ring. Peristomial cirri absent, or present, 1 pair (on posterior ring).

Gut more-or-less straight, lacking side branches. Foregut a muscular ventral pharynx. Pharynx jaws present, multiple jaw elements of different shapes and sizes. Multiple jaws comprising ventral mandibles and dorsal maxillae, 4–5 (right) and 4–6 (left) toothed plates in a semicircle. Pharynx maxillae asymmetrical (right maxilla III missing; right maxilla IV larger than left one). Maxillary carriers shorter than combined length of maxillae. Pharynx dorsolateral ciliated folds present.

First segment chaetous; second segment chaetous. First chaetiger with neurochaetae only; parapodia similar in length or slightly shorter than subsequent parapodia; parapodia more-or-less laterally directed and free from head; chaetae similar in orientation, length and thickness to other chaetae. Parapodia uniramous. Notopodial lobes absent (notopodial cirrus only present). Neuropodial lobes represented by at least one chaetal lobe. Lateral organs present (as a ciliated patch at ventral base of dorsal cirrus). Dorsal cirri present; more-or-less cirriform. Ventral cirri present; cirriform or tapering. Branchiae present, arise from lateral body (dorsal cirri), occur on mid-body segments; lateral branchiae branching.

Chaetae first appear on first segment after peristomium, arranged as a single bundle (or row) of many chaetae (lower bundle - neurochaetae - only). Aciculae present; in both dorsal and ventral positions (=noto- and neuroaciculae) (dorsal one sometimes absent; when present dorsal one at base of branchiae). Neuroaciculae distally tapering. Capillary chaetae present, in ventral (neuropodial) position, distally tapered to a point, edge smooth. Capillary chaetae, externally not pseudo-segmented. Capillary chaetae, internally not chambered or hollow. Comb-like chaetae present. Comb-like chaetae comb comprised of many long tines. Hooks present; with a distal hood; occur in ventral (neuropodial) position; occur over entire body (subacicular position). Compound chaetae present; in ventral (neuropodial) position; appendage distally tapering to slender tips (=spinigerous), or distally curved (=falcate; ie., one distal tooth); appendage not canaliculated; appendage with paired guards on each side of the crest; joint, appearance distinctly asymmetrical (=heterogomph); joint effected by ligament(s) (double ligaments); shaft tapering slightly or evenly thick from emergence to joint; shaft, internally solid, without distinct core. Compound chaetae present in most or all chaetigers.

Segmental organs are metanephridia; present in most segments of the body.

Tube absent, or present; leathery or parchment like.

**Literature used for description**. Glasby CJ, Fauchald K (2003). POLiKEY. An information system for polychaete families and higher taxa. Version 2, 5 June 2003. Australian Biological Resources Study, DCCEEW, Canberra, https:[//www.dcceew.gov.au/;](http://www.dcceew.gov.au/%3B) Zanol J, Budaeva N (2021)

7.12.1 Eunicidae In: Purschke G, Böggemann M, Westheide W (Eds), Handbook of Zoology, Annelida, Volume 3: Pleistoannelida, Sedentaria III and Errantia I. De Gruyter, Berlin, 414–452; Rouse GW, Pleijel, F, Tilic E (2022) Annelida. Oxford University Press, Oxford, UK.

# Euphrosinidae Williams, 1852

**Description**. Body segment number: variable. Body shape ovate to elliptical. Body pigmentation absent or present, often red-brown body and white chaetae; epidermis more-or-less smooth. Pygidium simple lobe. Pygidial appendages present, one pair of cirri and single medial papilla. Discrete head present (but enclosed by surrounding parapodia); complex in shape bearing appendages.

Prostomium narrow, keel- or ridge-shaped, eyes on head present, two pairs; head eyes simple pigmented cups. Prostomial antennae present; median and paired laterals; unarticulated. Palps present (resemble antennae); anteroventral; tapering (usually) sensory type; unarticulated, or multiple articulations (weakly). Caruncle present. Nuchal organs present; projecting considerably from prostomium (ciliated bands on either side of caruncle). Peristomium not visible.

Foregut a muscular ventral pharynx (mouth ventral, surrounded by paired ventrolateral lips). Protrusible proboscis present, with transverse ridges of thick cuticular lamellae, distal ring of papillae absent. Pharynx dorsolateral ciliated folds present.

First segment chaetous; second segment chaetous. First chaetiger with both notochaetae and neurochaetae; parapodia similar in length or slightly shorter than subsequent parapodia; parapodia anteriorly directed and wrapping around head, or more-or-less laterally directed and free from head; chaetae similar in orientation, length and thickness to other chaetae. Parapodia biramous; parapodial lobes prominent. Notopodial lobes long dorsal ridges. Neuropodial lobes represented by at least one chaetal lobe. Lateral organs present. Dorsal cirri present; more-or-less cirriform. Ventral cirri present; cirriform or tapering. Branchiae present, arise from lateral body (along notopodial ridges), occur on mid-body segments; lateral branchiae several tufts per parapodium.

Chaetae first appear on first segment after peristomium, calcareous, brittle, arranged in paired bundles (or rows) of many chaetae. Aciculae present; in both dorsal and ventral positions (=noto- and neuroaciculae) (numerous). Neuroaciculae distally tapering. Capillary chaetae present, in ventral (neuropodial) position. Forked chaetae present, furcate, or ringent, tines distinctly unequal in length. Spines present. Hooks absent.

Tube absent.

**Literature used for description**. Kudenov, J. (1993) Amphinomidae and Euphrosinidae (Annelida: Polychaeta) Principally from Antarctica, The Southern Ocean, and Subantarctic Regions. Antarctic Research Series 58: 93–150; Glasby CJ, Fauchald K (2003). POLiKEY. An information system for polychaete families and higher taxa. Version 2, 5 June 2003. Australian Biological Resources Study, DCCEEW, Canberra, https:[//www.dcceew.gov.au/;](http://www.dcceew.gov.au/%3B) Rouse GW, Pleijel, F, Tilic E (2022) Annelida. Oxford University Press, Oxford, UK.

# Fabriciidae Rioja, 1923

**Description**. Body segment number: fixed or variable, less than 14 segments. Body shape elongate, more-or-less equal width over entire length, regionalization present (subtle; thoracic uncini in neuropodial position, abdominal uncini notopodial), regionalization comprising two regions (comprising 8 thoracic segments and 2–4 abdominal segments), regions demarcated by inversion of parapodia (and shift in position of faecal groove from dorsal (thoracic) to ventral (abdominal)). Body translucent, gut visible or opaque, gut usually not visible, pigmentation absent or present, pigmentation radiolar crown may be pigmented; darkly pigmented spermathecae may resemble a pair of eyespots; epidermis more-or-less smooth. Pygidium simple lobe. Pygidial appendages absent. Discrete head absent (as a branchial crown comprising radioles and a pair of similar looking ventral filamentous appendages); radiolar crown present (2–3 pairs of radioles); internal 'skeleton' absent; modified radioles present, as a pair of ventral filamentous appendages.

Eyes on head present, one pair (on peristomium only); head eyes simple pigmented cups (red or black). Eyes on trunk absent, or present. Trunk eyes on lateral body. Caudal eyes absent, or present. Nuchal organs present (pair of internalized pouches in a dorsal pit above mouth). Peristomium a single ring, collar-like.

Foregut without a distinct ventral or axial pharynx. Protrusible proboscis absent. Heart bodies present (several pairs of branchial (or radiolar) hearts).

First segment chaetous; second segment chaetous. First chaetiger with notochaetae only (called collar chaetae); parapodia similar in length or slightly shorter than subsequent parapodia; parapodia more-or-less laterally directed and free from head; chaetae similar in orientation, length and thickness to other chaetae. Parapodia biramous; parapodial lobes prominent. Notopodial lobes represented by at least one chaetal lobe (anteriorly), or low lateral ridges (tori) (posteriorly). Neuropodial lobes low ridges (tori) (anteriorly), or represented by at least one chaetal lobe (posteriorly).

Chaetae first appear on first segment after peristomium, arranged in paired bundles (or rows) of many chaetae. Aciculae absent. Capillary chaetae present, in dorsal (notopodial) position or in ventral (neuropodial) position, distally tapered to a point or expanded (winged), edge smooth. Capillary chaetae, externally not pseudo-segmented. Capillary chaetae, internally not chambered or hollow. Uncini present (acicular type; i.e., with long shafts); with teeth arranged in transverse series above an enlarged main fang (=crested); in ventral (neuropodial) position on thorax and dorsal (notopodial) position on abdomen; arranged in one row.

Reproduction sexual.

Sexual reproduction hermaphroditic, or gonochoric. Segmental organs are metanephridia; metanephridia single anterior pair of excretory metanephridia and several more posterior ones for gamete release (excretory ones on segment 1); nephridial pores located anteriorly.

Tube present; membranous.

**Literature used for description**. Thorp JH, Lovell LL, Timm T, Martin P, Gelder SR, Govedich FR, Moser WE, Nakano T, Bielecki A, Bain BA, Utevsky S, Gil J, Glasby CJ, Martin D (2019) Phylum Annelida. In: Rogers DC, Thorp JH (Eds) Keys to Palaearctic Fauna: Thorp and Covich’s Freshwater invertebrates - Volume IV. Academic Press, Elsevier, pp. 357–518; Bick A (2021) 7.4.8 Fabriciidae Rioja, 1923 In: Purschke G, Böggemann M, Westheide W (Eds), Handbook of Zoology, Annelida, Volume 3: Pleistoannelida, Sedentaria III and Errantia I. De Gruyter, Berlin, 1–33; Capa M, Kupriyanova E, Nogueira JMdM, Bick A, Tovar-Hernández MA (2021) Fanworms:Yesterday, Today and Tomorrow. Diversity 13: 130, https://doi.org/10.3390/d13030130; Rouse GW, Pleijel F, Tilic E (2022) Annelida. Oxford University Press, Oxford, UK.

# Fauveliopsidae Hartman, 1971

**Description**. Body segment number: variable. Body shape elongate, more-or-less equal width over entire length or sausage or grub-shaped. Epidermis more-or-less smooth, or papillate. Pygidium simple lobe. Pygidial appendages absent (although small papillae around the anus are present in *Fauveliopsis adriatica*). Discrete head present; lobe-like without appendages; head retractable into anterior segments.

Prostomium bluntly conical. Nuchal organs present; paired low projections from posterolateral prostomium; indistinct dorsolateral ciliated patches. Peristomium a single ring, although appears double in some taxa.

Foregut a muscular ventral pharynx (uncertain). Pharynx dorsolateral ciliated folds present.

Heart bodies absent.

First segment chaetous (uncertain; possibly an achaetous segment is present); second segment chaetous. First chaetiger with both notochaetae and neurochaetae (needs confirmation); parapodia similar in length or slightly shorter than subsequent parapodia; parapodia more-or-less laterally directed and free from head, or anteriorly directed and wrapping around head (when prostomium retracted); chaetae similar in orientation, length and thickness to other chaetae. Parapodia biramous; parapodial lobes absent or very low. Notopodial lobes represented by at least one chaetal lobe. Neuropodial lobes represented by at least one chaetal lobe. Interramal fleshy process present. Interramal fleshy process papilla-like.

Chaetae first appear on first segment after peristomium (uncertain, may be the second), arranged in paired bundles (or rows) of many chaetae. Aciculae absent. Capillary chaetae present, in both dorsal and ventral positions, distally tapered to a point, edge smooth. Capillary chaetae, externally not pseudo-segmented. Capillary chaetae, internally not chambered or hollow. Spines present; slightly curved and more-or-less smooth; present in most or all chaetigers (spines only slightly thicker than capillaries); in both dorsal and ventral positions. Hooks absent.

Sperm sac present (chaetigers 5–8, sometimes associated with genital papillae). Ovaries present, unpaired; present in segments chaetigers 5–12. Segmental organs are metanephridia.

Tube absent, or present; membranous.

**Literature used for description**. Glasby CJ, Fauchald K (2003). POLiKEY. An information system for polychaete families and higher taxa. Version 2, 5 June 2003. Australian Biological Resources Study, DCCEEW, Canberra, https:[//www.dcceew.gov.au/;](http://www.dcceew.gov.au/%3B) Zhadan AE, Salazar-Vallejo SI (2019) 7.3.1.3 Fauveliopsidae Hartman, 1971 In Purschke G, Böggemann M, Westheide W (Eds), Handbook of Zoology, Annelida, Volume 1: Annelida Basal Groups and Pleistoannelida, Sedentaria I. De Gruyter, Berlin, 317–327; Rouse GW, Pleijel, F, Tilic E (2022) Annelida. Oxford University Press, Oxford, UK.

# Flabelligeridae de Saint-Joseph, 1894

**Description**. Body segment number: fixed (rarely) or variable, less than 14 segments (segments may be poorly defined). Body shape elongate, more-or-less equal width over entire length or sausage or grub-shaped. Body opaque, gut usually not visible or translucent, gut visible (rarely, gelatinous), pigmentation present, pigmentation yellowish to greenish-brown; epidermis papillate (benthic forms often with sediment often glued to their papillae). Bioluminescence absent, or present (in holopelagic species). Body surface with protective covering as gelatinous sheath, or absent. Pygidium simple lobe. Pygidial appendages absent. Discrete head present (though rarely seen as it is retractable); complex in shape bearing appendages; head retractable into anterior segments.

Eyes on head present, two pairs; head eyes simple pigmented cups. Palps present; anterodorsal; grooved (usually) feeding type; feeding palp longitudinally grooved. Nuchal organs present; paired low projections from posterolateral prostomium; indistinct dorsolateral ciliated patches. Peristomium not visible.

Gut straight except for a large midbody loop. Foregut a muscular ventral pharynx. Pharynx dorsolateral ciliated folds present. Heart bodies present.

First segment achaetous (unknown number of anterior achaetous segments that form an 'oral siphon'); second segment achaetous. First chaetiger with both notochaetae and neurochaetae; parapodia similar in length or slightly shorter than subsequent parapodia; parapodia more-or-less laterally directed and free from head, or anteriorly directed and wrapping around head (when prostomium retracted); chaetae similar in orientation, length and thickness to other chaetae, or slender and elongate, forming cage (or basket) around head (up to 6 chaetigers contributing to cage of chaetae). Parapodia biramous; parapodial lobes absent or very low. Notopodial lobes represented by at least one chaetal lobe. Neuropodial lobes represented by at least one chaetal lobe. Branchiae present, arise from dorsal body, occur first few segments (on the oral siphon). Dorsal branchiae simple filaments each arising directly from body wall (four or more pairs, may be retractable).

Chaetae absent (only in the holopelagic *Poebius*) or present, first appear on third segment after peristomium (poorly known; assuming the oral siphon comprises two fused segments), arranged in paired bundles (or rows) of many chaetae. Aciculae absent. Capillary chaetae present (some appearing barred), in dorsal (notopodial) position or in ventral (neuropodial) position, distally tapered to a point, edge smooth. Capillary chaetae, externally pseudo-segmented. Capillary chaetae, internally not chambered or hollow. Spines absent, or present; slightly curved and more-or-less smooth, or sharply bent (=geniculate) or recurved (sometimes called hooks); present in most or all chaetigers; in ventral (neuropodial) position only. Hooks absent. Compound chaetae absent, or present; in ventral (neuropodial) position; appendage distally curved (=falcate; ie., one distal tooth); appendage not canaliculated; appendage with a single hood open in front; joint, appearance flat or pseudocompound; joint effected by fold in external sheath of chaeta; shaft tapering slightly or evenly thick from emergence to joint; shaft, internally solid, without distinct core. Compound chaetae present in most or all chaetigers.

Segmental organs are metanephridia; metanephridia single anterior pair of excretory metanephridia and several more posterior ones for gamete release (excretory ones on segment 1); nephridial pores located anteriorly, gonoducts located around clitellum.

Tube absent.

**Literature used for description**. Glasby CJ, Fauchald K (2003). POLiKEY. An information system for polychaete families and higher taxa. Version 2, 5 June 2003. Australian Biological Resources Study, DCCEEW, Canberra, https:[//www.dcceew.gov.au/;](http://www.dcceew.gov.au/%3B) Salazar-Vallejo SI (2019)

7.3.1.6 Flabelligeridae Saint-Joseph, 1894 1 In: Purschke G, Böggemann M, Westheide W (Eds) Handbook of Zoology, Annelida, Volume 1: Annelida Basal Groups and Pleistoannelida, Sedentaria I. De Gruyter, Berlin, 398–421; Rouse GW, Pleijel, F, Tilic E (2022) Annelida. Oxford University Press, Oxford, UK.

# Gastrostomobdellidae Richardson 1971

**Description**. Body segment number: fixed, 34 segments including 2 preoral 'segments' (prostomium and peristomium) and 32 postoral segments. Secondary annulation present (disk-shaped receptors called sensillae mark the middle annulus of each segment); mid-body 6-annulate (typically); post-anal region uniannulate. Body shape elongate, more-or-less equal width over entire length (when relaxed; but can be dramatically shortened by contraction of longitudinal muscles during movement), dorsoventrally flattened. Anterior end sucker on ventral surface present; not clearly separated from rest of body. Body pigmentation absent or present, the Kinabalu Giant Red Leech is bright orange-red; epidermis papillate (papillae numerous, minute, hardly visible, 1–2 rows on every annulus). Posterior sucker on ventral surface present; anus positioned near posterior end (dorsally, just before posterior sucker).

Eyes on head absent. Postcephalic eyes absent. Eyes on trunk absent. Caudal eyes absent.

Gut straight with side branches. Foregut a muscular axial pharynx; muscular axial pharynx not rotated (euthylaematous) (check, some reports say strepsilaematous). Pharynx jaws absent or present, leech-type (either soft-muscular or horny) (as soft muscular 'jaws'). Protrusible proboscis absent. Crop present (tubular). Caeca of midgut (=posterior crop caeca) absent. Caeca of hindgut absent. Circulatory system absent.

Sexual reproduction hermaphroditic. Clitellum fully encircles body, in region of male and female pores. Clitellum occurs from X-XIII (usual position for leeches). Gastropores present (columnar duct with Y-shaped canal; two pores open to the crop); on segments XIV/XV. Testes present, many pairs, one pair per segment (= 2 or 3 in each annulus). Sperm sac present. Ovaries present, one pair. Egg sacs present; tubular. Oviduct single, shared oviduct from egg sac. Female gonoduct present. Female pores single, median, in segment XII or XIII. Male gonoducts present, with atrium present (weakly muscularised). Male pores single, median, in segment XI or XII. Penis absent (ejaculatory bulb also absent). Prostate gland absent.

**Literature used for description**. Sawyer RT (1986) Leech biology and behaviour. Vol. 1 Anatomy, Physiology, and behaviour. Oxford Science Publications, Oxford UK, 418 pp; Oceguera-Figueroa A, Phillips AJ, Pacheco-Chaves B, Reeves WK, Siddall ME (2011) Phylogeny of macrophagous leeches (Hirudinea, Clitellata) based on molecular data and evaluation of the barcoding locus. Zoologica Scripta 40: 194–203; Nakano T, Ramlah Z, Hikida T (2012) Phylogenetic position of gastrostomobdellid leeches (Hirudinida, Arhynchobdellida, Erpobdelliformes) and a new family for the genus Orobdella. Zoologica Scripta 41: 177–185. Nakano T, Eto K, Nishikawa K, Hossmann MY, Jeratthitikule E (2018) Systematic revision of the Southeast Asian macrophagous leeches, with the description of two new gastrostomobdellid species (Hirudinida: Arhynchobdellida: Erpobdelliformes). Zoological Journal of the Linnean Society 184: 1–30.

# Glossiphoniidae (Vaillant, 1890. Revised)

**Description**. Body segment number: fixed, 34 segments including 2 preoral 'segments' (prostomium and peristomium) and 32 postoral segments (true segments generally obscured by intervening superficial annulations, giving the impression of many more segments). Secondary annulation present (disk-shaped receptors called sensillae mark the middle annulus of each segment); anterior region uniannulate, or biannulate; mid-body triannulate (typically); posterior uniannulate, or biannulate. Body segments similar dimensions throughout. Body shape elongate, more-or-less equal width over entire length (when relaxed; but can be dramatically shortened by contraction of longitudinal muscles during movement becoming pyriform or leaf-like in shape), dorsoventrally flattened. Anterior end sucker on ventral surface present (poorly defined - narrower than body; surrounding mouth); not clearly separated from rest of body. Small mouth pore on ventral surface of sucker. Epidermis papillate, or tuberculate, with bumps of various sizes and arrangements; tuberculae arrangement in longitudinal rows (along 3 or 4 ridges). Posterior sucker on ventral surface present; circular; rays absent; anus positioned near posterior end (dorsally, just before posterior sucker). Elongate proboscis present; retractable and tube-like (protruded through a small mouth pore).

Eyes on head present, one pair or two pairs or three pairs (usually on the midline; rarely 4 annuli with paired eyespots; two pairs of eyespots on segment III in *Placobdella*); head eyes simple pigmented cups. Postcephalic eyes absent. Caudal eyes absent.

Gut straight with side branches. Foregut without a distinct ventral or axial pharynx. Pharynx jaws absent. Protrusible proboscis present. Crop present. Caeca of midgut (=posterior crop caeca) present (5–11 pairs). Caeca of hindgut present (4 pairs). Circulatory system present.

Sexual reproduction hermaphroditic. Clitellum fully encircles body, in region of male and female pores. Male and female pores separated by 1–2 annuli. Clitellum occurs from X-XIII (usual position for leeches). Testes present, many pairs (5–10 pairs in total), one pair per segment. Sperm sac present. Ovaries present, one pair. Egg sacs present; tubular (rarely bilobed). Oviduct separate one for each egg sac. Segmental organs are metanephridia; nephridial pores single (ventromedial), both nephridial pores and gonoducts located around clitellum. Female gonoduct present. Female pores single, median, in segment XII. Male gonoducts present, with atrium present, bilobed or fused (rarely, eg., *Theromyzon*). Male pores single, median, in segment XI. Penis absent. Prostate gland absent.

**Literature used for description**. Siddall ME, Borda E (2004) Leech Collections from Chile Including Two New species of *Helobdella* (Annelida: Hirudinida). American Museum Novitates 3457: 1–18; Siddall ME, Bely AE, Borda E (2006) Hirudinida Pp. 393–429. In Reproductive Biology and Phylogeny of Annelida. Series Editor BGM Jamieson. Volume 4. Editors G Rouse, F Pleijel. Science Publishers, Enfield, New Hampshire; Brusca, RC, Moore, W & Shuster, SM. (eds) (2016) Chapter 14. Phylum Annelida. In: Invertebrates, 3rd Edition. Sinauer Associates, Sunderland, MA; Thorp JH et al. 2019. Phylum Annelida, In Keys to Palaearctic Fauna: Thorp and Covich's Freshwater Invertebrates, Volume IV. Academic Press, Elsevier; Ahmed, RB, Gajda L, Utevsky S, Kvist S, Świątek P (2023) *Placobdella nabeulensis* sp. nov. (Hirudinea: Glossiphoniidae), a new glossiphoniiform leech from Palearctic North Africa. Molecular Biology Reports 50(8): 6753–6767; Rashni B, Brown KT, McLenachan PA, Lockhart PJ , Southgate PC, Lal MM (2023) Leech breach: a first record of the invasive freshwater leech *Helobdella europaea* (Hirudinea: Glossiphoniidae) in Fiji. Pacific Conservation Biology doi:10.1071/PC23017; Torres-Carrera G, Muñiz-Pareja FC, Albert N Maza-Acuña, Alejandro Oceguera-Figueroa (2023) Broad phylogenetic analyses of the leech family Glossiphoniidae (Annelida: Clitellata) reveals two independent origins of kangaroo leeches. Biological Journal of the Linnean Society, Volume 139: 192–201.

# Glossoscolecidae Michaelsen, 1900

**Description**. Body segment number: variable. Secondary annulation present. Body shape elongate, more-or-less equal width over entire length. Dorsal intersegmental furrow present (?), pores on mid-dorsal line present (?). Pygidium simple lobe. Pygidial appendages absent.

Prostomium bluntly conical.

Gut straight with side branches. Foregut a muscular dorsal pharynx. Gizzard present, segment VI. Calciferous glands present, in segment VII-XIV. Intestinal typhlosole present, formed from the inner (epithelial) layer only of the intestine. Heart bodies present, segment V.

Chaetae first appear on first segment after peristomium (=S2 for oligochaete workers), 2 per bundle (typically), in lumbricine arrangement, closely spaced lateral and ventrolateral pairs (anteriorly) or arranged in widely spaced lateral and ventrolateral pairs (in posterior body segments alternate between closely and widely spaced). Crotchet chaetae present, simple-pointed. Genital chaetae present (penial chaetae absent).

Sexual reproduction hermaphroditic. Clitellum thick, multilayered (often long, extending over many segments), partially encircles body, in region of male pores, posterior to female pores. Clitellum occurs from XV to XXV. Tubercula pubertatis present, paired ridges ventral to clitellum. Testes present, one pair in total or two pairs in total, one pair per segment; present in segments XX or XX and XI. Sperm sac absent, or present. Ovaries present, one pair; present in segment XIII. Egg sacs present. Segmental organs are metanephridia; both nephridial pores and gonoducts located around clitellum, one pair nephridia in each segment (holonephridia) (in intestinal region). Spermathecae absent or present, simple (usually), pre-testicular. Spermathecal pores present (often several pairs), located within 1 or 2 segments of male pores or located well anterior to male pores; 3 pairs; in segment VII-IX. Female gonoduct present. Female pores one pair (rarely 2 pairs), in segment XIV. Male gonoducts present (macroscopic male pores with ejaculatory bulbs), with atrium absent or present (with prostate like bursae). Male pores one pair, in segment XVI to XIX, opisthoporous (two or more segments following testicular segment). Prostate gland absent or present; prostate pore and male pore on segment XVII united, discharge through single pore.

**Literature used for description**. Brinkhurst RO and Jamieson, BGM (1971) Aquatic Oligochaeta of the World with contributions by DG Cook, DV Anderson, J van der Land, University of Toronto Press, Toronto, Canada, 860 pp; James SW (2000). An Illustrated Key to the Earthworms of The Samoan Archipelago (Oligochaeta: Glossoscolecidae, Moniligastridae). Technical Report No. 49, 11 pp.; Jamieson, BGM (2006) Non-leech Clitellata (with contributions by Marco Ferraguti). Pp. 235–392. In Reproductive Biology and Phylogeny of Annelida. Series Editor BGM Jamieson. Volume 4. Editors G Rouse, F Pleijel. Science Publishers, Enfield, New Hampshire; James SW, Davidson SK (2012) Molecular phylogeny of earthworms (Annelida : Crassiclitellata) based on 28S, 18S and 16S gene sequences. Invertebrate Systematics 26: 213–229; Plisko JD, Nxele, TC (2015) An Annotated Key Separating Foreign Earthworm Species from the Indigenous South African taxa (Oligochaeta: Acanthodrilidae, Eudrilidae, Glossoscolecidae, Lumbricidae, Megascolecidae, Microchaetidae, Ocnerodrilidae and Tritogeniidae). African Invertebrates 56:663–708; Thorp JH, Lovell LL, Timm T, Martin P, Gelder SR, Govedich FR, Moser WE, Nakano T, Bielecki A, Bain BA, Utevsky S, Gil J, Glasby CJ, Martin D (2019) Phylum Annelida. In: Rogers DC, Thorp JH (Eds) Keys to Palaearctic Fauna: Thorp and Covich’s Freshwater invertebrates - Volume IV. Academic Press, Elsevier, pp. 357–518; Misirlioðlu, M, Reynolds, JW, Stojanoviæ, M, Trakiæ, T, Sekuliæ, J, James, SW, Csuzdi, C, Decaëns, T, Lapied, E, Phillips, HRP, Cameron, EK, Brown, GG (2023) Earthworms (Clitellata, Megadrili) of the world: an updated checklist of valid species and families, with notes on their distribution. Zootaxa 5255 (1): 417–438.

# Glyceridae Grube, 1850

**Description**. Body segment number: variable. Secondary annulation present (2 or 3 rings). Body shape elongate, more-or-less equal width over entire length (highly tapered at ends). Body translucent, gut visible (when alive) or opaque, gut usually not visible, pigmentation absent (blood vessels easily visible through body wall); epidermis more-or-less smooth. Pygidium simple lobe. Pygidial appendages present, one pair of cirri. Discrete head present; complex in shape bearing appendages.

Prostomium conical, tapering to slender tip (elongate and often annulated), anteriorly with four identical small cirriform projections (prostomial appendages). Prostomial antennae absent (though cirriform appendages at tip of prostomium resemble antennae or antennae and palps). Nuchal organs present; paired low projections from posterolateral prostomium; posterolateral ciliated bulbs. Peristomium not visible.

Gut more-or-less straight, lacking side branches. Foregut a muscular axial pharynx. Pharynx jaws present, two pairs of jaws. Paired jaws fang-like, with a basal support. Protrusible proboscis present, with papillae in subterminal position (sometimes with terminal fingernail-like structures), irregularly arranged, distal ring of papillae present. Circulatory system absent.

First segment chaetous; second segment chaetous. First chaetiger with neurochaetae only; parapodia similar in length or slightly shorter than subsequent parapodia; parapodia more-or-less laterally directed and free from head; chaetae similar in orientation, length and thickness to other chaetae. Parapodia biramous (rarely uniramous, eg., *Hemipodia*); parapodial lobes prominent. Notopodial lobes represented by at least one chaetal lobe (rarely absent). Neuropodial lobes represented by at least one chaetal lobe. Dorsal cirri present; more-or-less cirriform. Ventral cirri present; cirriform or tapering. Branchiae absent or present, arise from lateral body (often retracted and not visible in fixed specimen), occur on mid-body segments; lateral branchiae digitiform, or single tuft per parapodium.

Chaetae arranged in paired bundles (or rows) of many chaetae or as a single bundle (or row) of many chaetae (lower bundle - neurochaetae - only). Aciculae present; in both dorsal and ventral positions (=noto- and neuroaciculae) (dorsal one absent in uniramous parapodia). Capillary chaetae present, in dorsal (notopodial) position, distally tapered to a point, edge spinose. Capillary chaetae, externally not pseudo-segmented. Capillary chaetae, internally not chambered or hollow. Hooks absent. Compound chaetae present; in ventral (neuropodial) position; appendage distally tapering to slender tips (=spinigerous), or distally curved (=falcate; ie., one distal tooth) (*Glycerella* only); appendage not canaliculated; appendage without hoods or guards; joint, appearance more-or-less symmetrical (=homogomph; includes also hemigomph and sesquigomph); joint effected by ligament(s); shaft tapering slightly or evenly thick from emergence to joint; shaft, internally solid, without distinct core. Compound chaetae present in most or all chaetigers.

Sexual reproduction gonochoric (some species undergo epitokous metamorphosis prior to breeding). Segmental organs are protonephridia; present in most segments of the body.

# Tube absent.

**Literature used for description**. Glasby CJ, Fauchald K (2003). POLiKEY. An information system for polychaete families and higher taxa. Version 2, 5 June 2003. Australian Biological Resources Study, DCCEEW, Canberra, https:[//www.dcceew.gov.au/;](http://www.dcceew.gov.au/%3B) Böggemann M (2022)

7.13.4.1 Glyceridae Grube, 1850, In: Purschke G, Böggemann M, Westheide W (Eds), Handbook of Zoology, Annelida, Volume 3: Pleistoannelida, Errantia II. De Gruyter, Berlin, 323–344; Rouse GW, Pleijel, F, Tilic E (2022) Annelida. Oxford University Press, Oxford, UK.

# Goniadidae Kinberg, 1866

**Description**. Body segment number: variable. Secondary annulation present (2 or 3 rings). Body shape elongate, more-or-less equal width over entire length (highly tapered at ends). Body translucent, gut visible (when alive, although less so than Glyceridae) or opaque, gut usually not visible, pigmentation absent (slightly iridescent); epidermis more-or-less smooth. Pygidium simple lobe. Pygidial appendages present, one pair of cirri. Discrete head present; complex in shape bearing appendages.

Prostomium conical, tapering to slender tip (elongate and often annulated), anteriorly with four identical small cirriform projections (prostomial appendages), eyes on head absent or present, one pair (basal) or two pairs (basal and distal); head eyes simple pigmented cups. Prostomial antennae absent (though cirriform appendages at tip of prostomium resemble antennae or antennae and palps). Nuchal organs present; paired low projections from posterolateral prostomium; posterolateral ciliated bulbs. Peristomium not visible.

Foregut a muscular axial pharynx. Pharynx jaws present, multiple jaw elements of different shapes and sizes. Multiple jaws comprising many dorsal jaw pieces (micrognaths) arranged in ring and usually pair ventral toothed plates (macrognaths) (chevrons may also be present proximally). Protrusible proboscis present, with papillae in subterminal position and with hardened (sclerotised) papillae of different structures (including micrognaths, macrognaths and chevrons), distal ring of papillae present. Circulatory system absent.

First segment chaetous; second segment chaetous. First chaetiger with neurochaetae only; parapodia similar in length or slightly shorter than subsequent parapodia; parapodia more-or-less laterally directed and free from head; chaetae similar in orientation, length and thickness to other chaetae. Parapodia biramous (notopodia reduced anteriorly); parapodial lobes prominent. Notopodial lobes represented by at least one chaetal lobe. Neuropodial lobes represented by at least one chaetal lobe. Dorsal cirri present; more-or-less cirriform. Ventral cirri present; cirriform or tapering.

Chaetae arranged in paired bundles (or rows) of many chaetae or as a single bundle (or row) of many chaetae (lower bundle - neurochaetae - only). Aciculae present; in both dorsal and ventral positions (=noto- and neuroaciculae) (dorsal one absent in uniramous parapodia). Capillary chaetae present, in dorsal (notopodial) position, distally tapered to a point, edge spinose. Capillary chaetae, externally not pseudo-segmented. Capillary chaetae, internally not chambered or hollow. Spines absent, or present; with a single distal or subdistal hair (=aristate or hooded) (*Glycinde*); present in most or all chaetigers; in dorsal (notopodial) position only. Hooks absent. Compound chaetae present; in ventral (neuropodial) position; appendage distally tapering to slender tips (=spinigerous), or distally curved (=falcate; ie., one distal tooth); appendage not canaliculated; appendage without hoods or guards; joint, appearance more-or-less symmetrical (=homogomph; includes also hemigomph and sesquigomph), or distinctly asymmetrical (=heterogomph); joint effected by ligament(s); shaft tapering slightly or evenly thick from emergence to joint; shaft, internally solid, without distinct core. Compound chaetae present in most or all chaetigers.

Segmental organs are protonephridia; present in most segments of the body. Tube absent.

**Literature used for description**. Glasby CJ, Fauchald K (2003). POLiKEY. An information system for polychaete families and higher taxa. Version 2, 5 June 2003. Australian Biological Resources Study, DCCEEW, Canberra, https:[//www.dcceew.gov.au/;](http://www.dcceew.gov.au/%3B) Böggemann M (2022)

7.13.4.1 Goniadidae Kinberg, 1865, In: Purschke G, Böggemann M, Westheide W (Eds), Handbook of Zoology, Annelida, Volume 3: Pleistoannelida, Errantia II. De Gruyter, Berlin, 345–365; Rouse GW, Pleijel, F, Tilic E (2022) Annelida. Oxford University Press, Oxford, UK.

# Haemadipsidae Blanchard, 1893

**Description**. Body segment number: fixed, 34 segments including 2 preoral 'segments' (prostomium and peristomium) and 32 postoral segments (true segments generally obscured by intervening superficial annulations, giving the impression of many more segments). Secondary annulation present (disk-shaped receptors called sensillae mark the middle annulus of each segment); mid-body triannulate, or 4-annulate, or 5-annulate, or 6-annulate, or 7-annulate; posterior uniannulate, or biannulate, or 3-annulate. Body shape elongate, more-or-less equal width over entire length (when relaxed; but can be dramatically shortened by contraction of longitudinal muscles during movement), dorsoventrally flattened. Anterior end sucker on ventral surface present (well-developed lateral buccal lobes and frill surrounding mouth); not clearly separated from rest of body. Large mouth on ventral surface of sucker. Epidermis tesselated (dorsum more so than ventrum). Posterior sucker on ventral surface present; elliptical (bearing a pair of trilobed ear-like flaps - caudal auricles - at base); rays present; anus positioned near posterior end (dorsally, just before posterior sucker).

Eyes on head absent or present, five pairs (usually; arranged in an arc on segments II-VI, with 3rd and 4th pairs separated by one annulus = ocular arch); head eyes simple pigmented cups.

Gut straight with side branches. Foregut a muscular axial pharynx; muscular axial pharynx not rotated (euthylaematous); extends from segments VII-IX, bulbous. Pharynx jaws present, leech-type (either soft-muscular or horny) (as soft muscular 'jaws'); two (duognathous) muscular jaws, or three (trignathous) muscular jaws arranged in a triangle (with or without teeth); denticles present, one row of teeth. Protrusible proboscis absent. Crop present (VIII-XIX). Caeca of midgut (=posterior crop caeca) present (10–12 pairs). Caeca of hindgut absent. Circulatory system absent.

Chaetae absent.

Sexual reproduction hermaphroditic. Clitellum fully encircles body, in region of male and female pores. Male and female pores separated by 3–9 annuli. Clitellum occurs from IX to XIII. Gonadal segments bearing genital papillae present. Testes present, many pairs (9 or 10 pairs), one pair per segment; present in segments XIII/XIV to XXII/XXIII. Sperm sac present. Ovaries present, one pair; present in segment XII. Egg sacs present; globular. Oviduct single, shared oviduct from egg sac. Vaginal sac present (VII/XIV-XV/XVII; connected to oviduct anteriorly). Segmental organs are metanephridia; metanephridia 17 pairs; metanephridia lateral (and intersegmental); nephridial pores single (ventromedial). Female gonoduct present, vaginal sac present (VII/XIV-XV/XVII; connected to female pore anteriorly). Female pores single, median, in segment XII/XIII. Male gonoducts present, with atrium present (may be lined with prostate tissue), fused. Male pores single, median, in segment XI. Penis present (straight). Prostate gland absent.

**Literature used for description**. Soos, A. (1967) Identification key to the leech (Hirudinoidea) genera of the world, with a catalogue of the species: IV. Haemadipsidae. Acta Zool. Acad. Scient. Hung. 13: 417–432; Siddall ME, Bely AE, Borda E (2006) Hirudinida Pp. 393–429. In Reproductive Biology and Phylogeny of Annelida. Series Editor BGM Jamieson. Volume 4. Editors G Rouse, F Pleijel. Science Publishers, Enfield, New Hampshire; Borda E, Oceguera-Figueroa A, Siddall ME (2008) On the classification, evolution and biogeography of terrestrial haemadipsoid leeches (Hirudinida: Arhynchobdellida: Hirudiniformes). Molecular Phylogenetics and Evolution 46: 142–154; Borda E, Siddall ME (2010) Insights into the evolutionary history of Indo-Pacific bloodfeeding terrestrial leeches (Hirudinida : Arhynchobdellida : Haemadipisdae). Invertebrate Systematics 24: 456–472; Lai YT, Nakano T, Chen JH (2011) Three species of land leeches from Taiwan, *Haemadipsa rjukjuana* comb. n., a new record for *Haemadipsa picta* Moore, and an updated description of *Tritetrabdella taiwana* (Oka). ZooKeys 139: 1–22. doi: 10.3897/zookeys.139.1711; Brusca, RC, Moore, W & Shuster, SM. (eds) (2016) Chapter 14. Phylum Annelida. In: Invertebrates, 3rd Edition. Sinauer Associates, Sunderland, MA; Thorp JH, Lovell LL, Timm T, Martin P, Gelder SR, Govedich FR, Moser WE, Nakano T, Bielecki A, Bain BA, Utevsky S, Gil J, Glasby CJ, Martin D (2019) Phylum Annelida. In: Rogers DC, Thorp JH (Eds) Keys to Palaearctic Fauna: Thorp and Covich’s Freshwater invertebrates - Volume IV. Academic Press, Elsevier, pp. 357–518.

# Haplotaxidae Michaelsen, 1900, sensu stricto

**Description**. Body segment number: variable. Secondary annulation absent or present (not in all segments). Body shape elongate, more-or-less equal width over entire length (very long and thread-like). Pygidium simple lobe. Pygidial appendages absent.

Prostomium bluntly conical.

Foregut a muscular dorsal pharynx (highly muscular in *Haplotaxis*). Gizzard present.

Chaetae present, one per bundle (rarely two per bundle; sometimes missing in dorsal bundles), first appear on first segment after peristomium, present along most of body (dorsal chaetae sometimes absent from anterior segments), in lumbricine arrangement, - widely spaced lateral and ventrolateral pairs. Capillary (=hair) chaetae absent. Crotchet chaetae present, simple or bifid (shorter upper tooth; dorsal crotchets may be much smaller than ventral ones, and sometimes lacking). Genital chaetae absent, or present.

Sexual reproduction hermaphroditic. Clitellum thin, single layered, in region of male and female pores. Clitellum occurs from X or XI for several segments. Testes present, two pairs in total (rarely one pair), one pair per segment; present in segments X and XI (rarely IX and X). Ovaries present, one pair or two pairs; present in segments XII and XIII (rarely XI and XII or only in XII). Segmental organs are metanephridia; nephridial pores located anteriorly, gonoducts located around clitellum or nephridial pores located posterior to gonoducts (in both cases pores near ventral chaetae). Spermathecae present, simple, pre-testicular. Spermathecal pores present, located well anterior to male pores ; 1–4 pairs; in segment VI-IX. Female gonoduct absent or present. Female pores two pairs, in segment XIII and XIV (or XI and XII; XII and XIII). Male gonoducts present, with atrium absent. Male pores one pair or two pairs, in segment XI and XII (or X and XI), plesioporous (in segment following testicular segment). Penis absent or present. Prostate gland absent.

**Literature used for description**. Brinkhurst RO and Jamieson, BGM (1971) Aquatic Oligochaeta of the World with contributions by DG Cook, DV Anderson, J van der Land, University of Toronto Press, Toronto, Canada, 860 pp; Blakemore, RJ (2006). Introductory Key of the revised families of earthworms of the World. Unpub. March 2006; Jamieson, BGM (2006) Non-leech Clitellata (with contributions by Marco Ferraguti). Pp. 235–392. In Reproductive Biology and Phylogeny of Annelida. Series Editor BGM Jamieson. Volume 4. Editors G Rouse, F Pleijel. Science Publishers, Enfield, New Hampshire; Timm, T (2009) A guide to the freshwater Oligochaeta and Polychaeta of Northern and Central Europe. Lauterbornia 66: 1–235; Martin P, Fend S, Martinsson S, Klinth M, Torii T, Erséus C (2024) Towards an integrative revision of Haplotaxidae (Annelida: Clitellata). Zoological Journal of the Linnean Society 202, zlae141, doi.org/10.1093/zoolinnean/zlae141

.

# Hartmaniellidae Imajima, 1977

**Description**. Body segment number: variable. Body shape elongate, more-or-less equal width over entire length, regionalization present, regionalization comprising two regions, regions demarcated by laterally-directed thoracic parapodia and dorsally-directed midbody and abdominal parapodia. Pygidium simple lobe. Pygidial appendages present, one pair of cirri. Discrete head present; lobe-like without appendages.

Prostomium rounded to oval. Nuchal organs present; paired low projections from posterolateral prostomium; indistinct dorsolateral ciliated patches. Peristomium a double ring. Foregut a muscular ventral pharynx. Pharynx jaws present, multiple jaw elements of different shapes and sizes. Multiple jaws comprising ventral mandibles and dorsal maxillae, 4–5 (right) and 4–6 (left) toothed plates in a semicircle (intermediate between prionognath and labidognath). Pharynx maxillae symmetrical. Maxillary carriers similar in length to combined length of maxillae. Protrusible proboscis present, with papillae in subterminal position, distal ring of papillae absent. Pharynx dorsolateral ciliated folds present.

First segment chaetous (although parapodia very reduced or possibly absent (see Fig. C in plate)); second segment chaetous. First chaetiger with both notochaetae and neurochaetae (notochaetae may be internalised); parapodia similar in length or slightly shorter than subsequent parapodia; parapodia more-or-less laterally directed and free from head; chaetae similar in orientation, length and thickness to other chaetae. Parapodia biramous (best developed in mid and posterior body); parapodial lobes prominent. Notopodial lobes represented by at least one chaetal lobe (posteriorly), or absent (anteriorly). Neuropodial lobes represented by at least one chaetal lobe. Interramal fleshy process present. Interramal fleshy process cirrus-like (similar in size to parapodial lobes; probably function as branchiae). Lateral organs present (as a ciliated patch at ventral base of dorsal cirrus). Dorsal cirri present; more-or-less cirriform. Ventral cirri present; cirriform or tapering.

Chaetae first appear on first segment after peristomium, arranged as a single bundle (or row) of many chaetae (lower bundle - neurochaetae - only). Aciculae present; in both dorsal and ventral positions (=noto- and neuroaciculae). Neuroaciculae distally tapering. Capillary chaetae present, in ventral (neuropodial) position, distally tapered to a point or sharply bent, edge smooth or hirsute-serrate. Capillary chaetae, externally not pseudo-segmented. Capillary chaetae, internally not chambered or hollow. Spines present; with a single distal or subdistal hair (=aristate or hooded); present in most or all chaetigers; in ventral (neuropodial) position only. Hooks absent. Compound chaetae present; in ventral (neuropodial) position; appendage distally tapering to slender tips (=spinigerous); appendage not canaliculated; appendage without hoods or guards; joint, appearance distinctly asymmetrical (=heterogomph); joint effected by ligament(s); shaft tapering slightly or evenly thick from emergence to joint; shaft, internally solid, without distinct core. Compound chaetae present in most or all chaetigers.

Tube absent.

**Literature used for description**. Glasby CJ, Fauchald K (2003). POLiKEY. An information system for polychaete families and higher taxa. Version 2, 5 June 2003. Australian Biological Resources Study, DCCEEW, Canberra, https:[//www.dcceew.gov.au/;](http://www.dcceew.gov.au/%3B) Rouse GW, Pleijel, F, Tilic E (2022) Annelida. Oxford University Press, Oxford, UK.

# Hesionidae Grube, 1850

**Description**. Body segment number: variable (although fixed at 21 segments in Hesionini). Body shape elongate, more-or-less equal width over entire length or widest anteriorly and tapering posteriorly. Body translucent, gut visible (rarely) or opaque, gut usually not visible, pigmentation absent or present, pigmentation reddish-brown base colour with brownish spots, blotches or stripes on dorsal surface; some genera have an iridescent sheen; epidermis more-or-less smooth. Pygidium simple lobe. Pygidial appendages present, one pair of cirri or one pair of cirri and single medial papilla. Discrete head present; complex in shape bearing appendages.

Prostomium rounded to oval, facial tubercle absent or present, eyes on head present (rarely absent), two pairs; head eyes compound, with lenses (often reddish). Prostomial antennae present; paired, lateral, or median and paired laterals; unarticulated. Palps present, or absent (rarely); anteroventral; tapering (usually) sensory type; unarticulated, or biarticulated. Nuchal organs present; paired low projections from posterolateral prostomium; indistinct dorsolateral ciliated patches. Peristomium not visible.

Gut more-or-less straight, lacking side branches. Foregut a muscular axial pharynx. Pharynx jaws absent or present, one pair of lateral jaws. Paired jaws plate-like or fang-like. Pharynx teeth absent or present, as a single tooth. Protrusible proboscis present, smooth, distal ring of papillae present (reduced in some taxa).

First segment tentaculate (tentacular segments fused, or at least not distinguishable dorsally); second segment achaetous (tentacular segments fused, or at least not distinguishable dorsally). Tentacular cirri present; 6–8 pair(s) (rarely 2 pairs); arise over two or more segments (on first 3–5 segments); internal aciculae present in at least some cirri. First chaetiger with neurochaetae only; parapodia similar in length or slightly shorter than subsequent parapodia; parapodia more-or-less laterally directed and free from head; chaetae similar in orientation, length and thickness to other chaetae. Parapodia uniramous, or biramous; parapodial lobes prominent. Notopodial lobes represented by at least one chaetal lobe, or absent (notochaetae may be absent). Neuropodial lobes represented by at least one chaetal lobe. Dorsal cirri present; more-or-less cirriform (with distinct basal cylindrical cirrophores). Ventral cirri present; cirriform or tapering.

Chaetae first appear on third segment after peristomium or fourth segment after peristomium or fifth segment after peristomium, arranged in paired bundles (or rows) of many chaetae or as a single bundle (or row) of many chaetae (lower bundle - neurochaetae - only). Aciculae present; in both dorsal and ventral positions (=noto- and neuroaciculae). Neuroaciculae distally tapering. Capillary chaetae present, in dorsal (notopodial) position, distally tapered to a point, edge smooth or hirsute-serrate. Capillary chaetae, externally not pseudo-segmented. Capillary chaetae, internally not chambered or hollow. Forked chaetae absent, or present (rarely), furcate, tines distinctly unequal in length. Spines absent, or present (rarely); slightly curved and more-or-less smooth. Hooks absent (present in the notopodia of one genus). Compound chaetae present; in ventral (neuropodial) position; appendage distally tapering to slender tips (=spinigerous), or distally curved (=falcate; ie., one distal tooth) (uni- or bidentate); appendage not canaliculated; appendage without hoods or guards; joint, appearance distinctly asymmetrical (=heterogomph); joint effected by ligament(s); shaft tapering slightly or evenly thick from emergence to joint; shaft, internally chambered, with camerated core. Compound chaetae present in most or all chaetigers (neurochaetae only).

Segmental organs are metanephridia; present in most segments of the body. Penis present (rarely) or absent.

Tube absent.

**Literature used for description**. Glasby CJ, Fauchald K (2003). POLiKEY. An information system for polychaete families and higher taxa. Version 2, 5 June 2003. Australian Biological Resources Study, DCCEEW, Canberra, https:[//www.dcceew.gov.au/;](http://www.dcceew.gov.au/%3B) Rouse GW, Pleijel, F, Tilic, E (2022) Annelida. Oxford University Press, Oxford, UK.

# Hirudinidae Whitman, 1886

**Description**. Body segment number: fixed, 34 segments including 2 preoral 'segments' (prostomium and peristomium) and 32 postoral segments (true segments generally obscured by intervening superficial annulations (3–16), giving the impression of many more segments). Secondary annulation present (disk-shaped receptors called sensillae mark the middle annulus of each segment); anterior region uniannulate, or biannulate, or triannulate; mid-body triannulate, or 4-annulate, or 5-annulate (5-annulate segments are typical); posterior uniannulate, or biannulate, or 3-annulate, or 4-annulate. Body shape elongate, more-or-less equal width over entire length (when relaxed; but can be dramatically shortened by contraction of longitudinal muscles during movement), dorsoventrally flattened. Anterior end sucker on ventral surface present (surrounding mouth); not clearly separated from rest of body. Body pigmentation absent or present, dark brown-green-black base colour with lighter longitudinal stripes/blotches; epidermis more-or-less smooth, or papillate. Posterior sucker on ventral surface present; circular; rays absent; anus positioned near posterior end (dorsally, just before posterior sucker).

Eyes on head present, five pairs (usually; arranged in an arc on segments II-VI, with 3rd and 4th pairs separated by one annulus); head eyes simple pigmented cups.

Gut more-or-less straight, lacking side branches or straight with side branches. Foregut a muscular axial pharynx; muscular axial pharynx not rotated (euthylaematous). Pharynx jaws absent or present, leech-type (either soft-muscular or horny) (as soft muscular 'jaws'); two (duognathous) muscular jaws, or three (trignathous) muscular jaws arranged in a triangle (pharyngeal ridge between each jaw in Macrobdellinae); denticles absent or present, one row of teeth (rarely 2 rows). Protrusible proboscis absent (a low fold, 'velum', separates the buccal cavity from the sucker cavity). Crop present. Caeca of midgut (=posterior crop caeca) present (though reduced in number in the non-blood feeding species). Circulatory system absent.

Sexual reproduction hermaphroditic. Clitellum fully encircles body, in region of male and female pores. Male and female pores separated by 3–5 annuli. Clitellum occurs from IX-XIII (usual position for leeches). Gastropores absent (usually; only present on segments XII and XIII in *Philobdella floridiana*). Testes present, many pairs (9 or 10 pairs), one pair per segment. Sperm sac present. Ovaries present, one pair. Egg sacs present; globular. Oviduct single, shared oviduct from egg sac, or separate one for each egg sac. Segmental organs are metanephridia; nephridial pores paired (ventrolateral) or single (ventromedial), present in most segments of the body (between VII-XXIV; 17 pairs). Female gonoduct present, vaginal sac present. Female pores single, median, in segment XI-XII. Male gonoducts present, with atrium present, fused. Male pores single, median, in segment X-XI. Penis present (recurved shape). Prostate gland absent.

**Literature used for description**. Sawyer RT (1986) Leech biology and behaviour. Vol. 1 Anatomy, Physiology, and behaviour. Oxford Science Publications, Oxford UK, 418 pp; Siddall ME, Bely AE, Borda E (2006) Hirudinida Pp. 393–429. In Reproductive Biology and Phylogeny of Annelida. Series Editor BGM Jamieson. Volume 4. Editors G Rouse, F Pleijel. Science Publishers, Enfield, New Hampshire; Borda E, Oceguera-Figueroa A, Siddall ME (2008) On the classification, evolution and biogeography of terrestrial haemadipsoid leeches (Hirudinida: Arhynchobdellida: Hirudiniformes). Molecular Phylogenetics and Evolution 46: 142–154; Phillips AJ, Siddall ME (2009) Poly-paraphyly of Hirudinidae: many lineages of medicinal leeches. BMC Evolutionary Biology 9: 246. doi:10.1186/1471–2148–9-246; Phillips AJ, Arauco-Brown R, Oceguera-Figueroa A, Gomez GP et al. (2010) *Tyrannobdella rex* N. Gen. N. Sp. and the Evolutionary Origins of Mucosal Leech Infestations. PLoS ONE 5(4): e10057. doi:10.1371/journal.pone.0010057; Brusca, RC, Moore, W & Shuster, SM. (eds) (2016) Chapter 14. Phylum Annelida. In: Invertebrates, 3rd Edition. Sinauer Associates, Sunderland, MA; Thorp JH, Lovell LL, Timm T, Martin P, Gelder SR, Govedich FR, Moser WE, Nakano T, Bielecki A, Bain BA, Utevsky S, Gil J, Glasby CJ, Martin D (2019) Phylum Annelida. In: Rogers DC, Thorp JH (Eds) Keys to Palaearctic Fauna: Thorp and Covich’s Freshwater invertebrates - Volume IV. Academic Press, Elsevier, pp. 357–518; Sawyer RT (2019). Description of the Southern swamp leech *Philobdella floridana* (Verrill, 1874)(Annelida: Hirudinea) from the Outer Banks, North Carolina, USA, with a revision of the species. Zootaxa 4544: 189–213.

# Histriobdellidae Claus & Moquin-Tandon, 1884

**Description**. Body segment number: fixed, less than 14 segments (9 to about 13). Body shape elongate, more-or-less equal width over entire length. Body translucent, gut visible. Pygidium deeply cleft forming two large feet (locomotory appendages). Discrete head present; complex in shape bearing appendages (palps, antennae, papillae).

Prostomium rounded to oval. Prostomial antennae present; median and paired laterals; unarticulated. Palps present; anterodorsal; tapering (usually) sensory type; unarticulated. Nuchal organs absent, or present; paired low projections from posterolateral prostomium; indistinct dorsolateral ciliated patches. Peristomium a single ring. Peristomial cirri present, 1 pair (laterally positioned papillae (adhesive organs) are functionally locomotory appendages).

Gut more-or-less straight, lacking side branches. Foregut a muscular ventral pharynx. Pharynx jaws present, multiple jaw elements of different shapes and sizes. Multiple jaws comprising ventral mandibles and dorsal maxillae, 2 pairs of toothed plates plus many small free denticles arranged in longitudinal rows (plates very small and not always toothed; additionally, there is an unpaired dorsal rod). Pharynx maxillae symmetrical. Superior row fewer than 8 pairs. Maxillary carriers shorter than combined length of maxillae. Pharynx dorsolateral ciliated folds present. Circulatory system absent.

First segment chaetous, or achaetous; second segment chaetous. Parapodia absent (although they have lateral projections (cirri), they are structurally very different from parapodia). Dorsal cirri present (uncertain homology, but assumed to be dorsal cirri); more-or-less cirriform. Ventral cirri present; cirriform or tapering (assumed to be cirri).

Chaetae absent.

Segmental organs are protonephridia; several pairs in anterior body. Penis present (also called a copulatory organ; located ventrally between segments 4 and 5), with hardened sheath absent or present (chitinised spinous structure).

**Literature used for description**. Glasby CJ, Fauchald K (2003). POLiKEY. An information system for polychaete families and higher taxa. Version 2, 5 June 2003. Australian Biological Resources Study, DCCEEW, Canberra, https:[//www.dcceew.gov.au/;](http://www.dcceew.gov.au/%3B) Tzetlin A, Budaeva N, Vortsepneva E, Helm C (2020) New insights into the morphology and evolution of the ventral pharynx and jaws in Histriobdellidae (Eunicida, Annelida). Zoological Letters 6: 14, https://doi.org/10.1186/s40851–020–00168–2; Helm C, Vila I, Budaeva N (2021) 7.12.5 Histriobdellidae Claus & Moquin-Tandon, 1884 In: Purschke G, Böggemann M, Westheide W (Eds), Handbook of Zoology, Annelida, Volume 3: Pleistoannelida, Sedentaria III and Errantia I. De Gruyter, Berlin, 452–459; Rouse GW, Pleijel, F, Tilic E (2022) Annelida. Oxford University Press, Oxford, UK.

# Hormogastridae Michaelsen, 1900

**Description**. Body segment number: variable. Body shape elongate, more-or-less equal width over entire length. Dorsal intersegmental furrow present (?), pores on mid-dorsal line present (?). Pygidium simple lobe. Pygidial appendages absent.

Prostomium bluntly conical.

Foregut a muscular dorsal pharynx. Gizzard present (2 or 3, each in a single segment) between segments VI-VIII. Calciferous glands present. Intestinal typhlosole present, formed from all layers of the intestine (multilamellate).

Chaetae first appear on first segment after peristomium (=S2 for oligochaete workers). Crotchet chaetae present, simple-pointed. Genital chaetae present.

Sexual reproduction hermaphroditic. Clitellum thick, multilayered, partially encircles body or fully encircles body, in region of male and female pores. Clitellum occurs from XII to XIV for about 17 segments. Tubercula pubertatis present, paired ridges ventral to clitellum. Gonadal segments bearing genital papillae absent, or present. Testes present, one pair in total or two pairs in total; present in segments X and XI, or XI only. Sperm sac absent. Ovaries present, one pair; present in segments XIII. Segmental organs are metanephridia; both nephridial pores and gonoducts located around clitellum. Spermathecae present, post-testicular or in testicular segments. Spermathecal pores present, located within 1 or 2 segments of male pores; 2–9 pairs; in segment IX-X (in *Ailoscolex*); XIII-XVI in other genera (intersegmental). Female gonoduct present. Female pores one pair, in segment XIV. Male gonoducts present (2 pairs). Male pores one pair or two pairs, in segment XIII to XX or later, opisthoporous (two or more segments following testicular segment). Prostate gland present, more than one pair. Prostate pores in segments XXI to XXIV.

**Literature used for description**. Blakemore, RJ (2006). Introductory Key of the revised families of earthworms of the World. Unpub. March 2006; Jamieson, BGM (2006) Non-leech Clitellata (with contributions by Marco Ferraguti). Pp. 235–392. In Reproductive Biology and Phylogeny of Annelida. Series Editor BGM Jamieson. Volume 4. Editors G Rouse, F Pleijel. Science Publishers, Enfield, New Hampshire; Rota E, de Jong Y (2015) Fauna Europaea: Annelida

- Terrestrial Oligochaeta (Enchytraeidae and Megadrili), Aphanoneura and Polychaeta. Biodiversity Data Journal 3: e5737. doi: 10.3897/BDJ.3.e5737.

# Hrabeiellidae Christoffersen, 2012

**Description**. Body probably with fixed number of segments (16) including 1 preoral 'segment' (peristomium) and 15 postoral segments. Body shape elongate, more-or-less equal width over entire length (but short). Body translucent, gut visible, pigmentation present, pigmentation conspicuous white or green epidermal pigment glands and subepidermal gland cells ('cuticular glands'), up to four rows per segment; glands most dense on head and anal segments. Pygidium simple lobe (funnel-like). Pygidial appendages absent.

Prostomium rounded to oval. Nuchal organs present; paired low projections from posterolateral prostomium; indistinct dorsolateral ciliated patches.

Gut more-or-less straight (posterior intestine sinuous), lacking side branches. Foregut a muscular dorsal pharynx.

Protrusible proboscis absent. Gizzard absent. Calciferous glands absent.

Chaetae two or more (usually 2-3, exceptionally 4) per bundle, first appear on first segment after peristomium, more-or-less in lumbricine arrangement (paired upper and lower bundles of one or a few chaetae each), although pairs lie closely spaced in more ventrolateral position. Chaetae distally bristled, shovel-shaped; present in most chaetigers in ventral (neuropodial) position.

Sexual reproduction hermaphroditic (simultaneous). Testes present, one pair in total; present in segment VI. Ovaries present; present in segments XIII or XIV, ovisacs extending back to last segment. Egg sacs present. Nephridia present in most segments of the body, one pair nephridia in each segment (holonephridia). Male gonoducts present, with atrium present, fused (ventral bulb-like structure). Male pores single, median, in segment VI, prosoporous. Penis present (unpaired). Prostate gland present.

Tube absent.

# Literature used for description. Pizl V, Chalupský J (1984) *Hrabeiella periglandulata* gen. et sp. n. (Annelida) – a curious worm from Czechoslovakia. Vìstník Èeskoslovenské spoleènosti zoologické 48: 291 295; Glasby CJ, Fauchald K (2003). POLiKEY. An information system for polychaete families and higher taxa. Version 2, 5 June 2003. Australian Biological Resources Study, DCCEEW, Canberra, https:[//www.dcceew.gov.au/;](http://www.dcceew.gov.au/%3B) Rota E, Lupetti P (2015) An ultrastructural investigation of Hrabeiella Pizl & Chalupský, 1984 (Annelida). II. The spermatozoon. Tissue and Cell 29(5): 603–609; Purschke G (2019) Hrabeiellidae Christoffersen, 2012, In Purschke G, Böggemann M, Westheide W (Eds), Handbook of Zoology, Annelida, Volume 2: Pleistoannelida, Sedentaria II. De Gruyter, Berlin, 275–284; Rouse GW, Pleijel, F, Tilic E (2022) Annelida. Oxford University Press, Oxford, UK.

# Iospilidae Bergström, 1914

**Description**. Body segment number: variable. Body shape elongate, more-or-less equal width over entire length, regionalization absent (although first few parapodia are uniramous compared to remainder which are biramous). Body translucent, gut visible, pigmentation absent or present, pigmentation may have segmental pigment spots on anterodorsal side of parapodia. Pygidium simple lobe. Pygidial appendages absent. Discrete head present; lobe-like without appendages. Prostomium rounded to oval, eyes on head present, one pair; head eyes compound, with lenses. Palps present; anteroventral; tapering (usually) sensory type (bulbous); unarticulated. Nuchal organs present. Peristomium not visible.

Foregut a muscular axial pharynx. Pharynx jaws absent or present, one pair of lateral jaws.

Paired jaws fang-like. Protrusible proboscis present, distal ring of papillae present.

First segment tentaculate; second segment chaetous and tentaculate. Tentacular cirri present; 2 pair(s); arise over two or more segments (segments appear fused); internal aciculae absent. First chaetiger with neurochaetae only; parapodia similar in length or slightly shorter than subsequent parapodia; parapodia more-or-less laterally directed and free from head; chaetae similar in orientation, length and thickness to other chaetae. Parapodia uniramous and biramous (only anteriormost parapodia are uniramous); parapodial lobes prominent. Notopodial lobes absent (though dorsal cirri are present). Neuropodial lobes represented by at least one chaetal lobe. Dorsal cirri present; flattened and foliaceous. Ventral cirri present; cirriform or tapering.

Chaetae first appear on second segment after peristomium, arranged as a single bundle (or row) of many chaetae (lower bundle - neurochaetae - only). Aciculae present; in ventral position (=neuroaciculae). Neuroaciculae distally tapering. Capillary chaetae absent or present, in ventral (neuropodial) position, distally tapered to a point, edge hirsute-serrate. Capillary chaetae, externally not pseudo-segmented. Capillary chaetae, internally not chambered or hollow. Hooks absent. Compound chaetae present; in ventral (neuropodial) position; appendage distally tapering to slender tips (=spinigerous); appendage not canaliculated; appendage without hoods or guards; joint, appearance distinctly asymmetrical (=heterogomph); joint effected by ligament(s); shaft tapering slightly or evenly thick from emergence to joint; shaft, internally solid, without distinct core. Compound chaetae present in most or all chaetigers.

Tube absent.

**Literature used for description**. Glasby CJ, Fauchald K (2003). POLiKEY. An information system for polychaete families and higher taxa. Version 2, 5 June 2003. Australian Biological Resources Study, DCCEEW, Canberra, https:[//www.dcceew.gov.au/;](http://www.dcceew.gov.au/%3B) Rouse GW, Pleijel, F, Tilic E (2022) Annelida. Oxford University Press, Oxford, UK.

# Iphionidae Kinberg, 1856

**Description**. Body segment number: fixed (for some taxa less than 14 segments) or variable. Body shape ovate to elliptical, dorsoventrally flattened. Body opaque, gut usually not visible, pigmentation present, pigmentation yellowish to orange; more rarely blackish; epidermis more-or-less smooth, or papillate. Body surface with protective covering as scales (elytrae), dorsally. Pygidium simple lobe. Pygidial appendages present, one pair of cirri. Discrete head present; complex in shape bearing appendages.

Prostomium rounded to oval, anteriorly incised (anterior notch), facial tubercle absent, eyes on head absent (rarely) or present, two pairs; head eyes compound, with lenses. Prostomial antennae absent, or present; median one only, or paired, lateral, or median and paired laterals; unarticulated, or consist of basal ceratophore and distal ceratostyle. Palps present; anteroventral; tapering (usually) sensory type; unarticulated. Nuchal organs absent, or present; single antenna-like projection from posterior prostomium. Peristomium not visible.

Foregut a muscular axial pharynx. Pharynx jaws present, two pairs of jaws. Paired jaws fang-like. Protrusible proboscis present, distal ring of papillae present.

First segment chaetous and tentaculate; second segment chaetous. Tentacular cirri present;

2 pair(s); arise on a single segment. First chaetiger without external chaetae, or with notochaetae only, or with neurochaetae only, or with both notochaetae and neurochaetae; parapodia similar in length or slightly shorter than subsequent parapodia; parapodia anteriorly directed and wrapping around head; chaetae similar in orientation, length and thickness to other chaetae. Parapodia biramous; parapodial lobes prominent. Notopodial lobes represented by at least one chaetal lobe. Neuropodial lobes represented by at least one chaetal lobe. Dorsal cirri present (present on non-elytrous segments); more-or-less cirriform. Elytra with a tuberculated pentagonal or hexagonal pattern. Elytra, post segment 7 occur on every other segment from segment 7 to midbody, then present on every segment, or absent, to end of body (13–14 pairs in total). Ventral cirri present; cirriform or tapering. Branchiae absent or present, arise from lateral body or arise from dorsal body, occur on mid-body segments; lateral branchiae digitiform, or branching. Dorsal branchiae simple filaments each arising directly from body wall.

Chaetae first appear on first segment after peristomium or second segment after peristomium, arranged in paired bundles (or rows) of many chaetae or as a single bundle (or row) of many chaetae (lower bundle - neurochaetae - only). Aciculae present; in both dorsal and ventral positions (=noto- and neuroaciculae). Neuroaciculae distally tapering. Capillary chaetae present, in dorsal (notopodial) position, distally tapered to a point, edge smooth or hirsute-serrate or spinose or corn-eared. Capillary chaetae, externally not pseudo-segmented. Capillary chaetae, internally not chambered or hollow. Spines present; slightly curved and more-or-less smooth, or with small teeth or spinelets (proximally); present in most or all chaetigers; in both dorsal and ventral positions. Hooks absent. Compound chaetae absent.

Tube absent, or present; membranous.

**Literature used for description**. Rouse GW, Pleijel, F, Tilic E (2022) Annelida. Oxford University Press, Oxford, UK.

# Kynotidae Jamieson, 1971

**Description**. Body segment number: variable. Secondary annulation absent or present (segments IV to XIII may be biannulate). Body shape elongate, more-or-less equal width over entire length. Dorsal intersegmental furrow present (?), pores on mid-dorsal line present (?).

Prostomium bluntly conical. Foregut a muscular dorsal pharynx.

Chaetae first appear on first segment after peristomium (=S2 for oligochaete workers), in lumbricine arrangement - paired upper and lower bundles of one or a few chaetae each. . Crotchet chaetae present, simple-pointed. Genital chaetae present.

Sexual reproduction hermaphroditic. Clitellum thick, multilayered, partially encircles body or fully encircles body, posterior to male pore(s), posterior to female pore(s). Clitellum occurs from XVIII to XLVII. Tubercula pubertatis absent. Gonadal segments with extensions of the body wall absent, or present as claspers. Testes present, two pairs in total; present in segments X and XI. Ovaries present, one pair; present in segments XIII. Segmental organs are metanephridia; both nephridial pores and gonoducts located around clitellum. Spermathecae present, post-testicular. Spermathecal pores present, located within 1 or 2 segments of male pores; 3 pairs (usually); in segment XIII to XVII. Female gonoduct present. Female pores one pair, in segment XIV. Male gonoducts present, with atrium present. Male pores one pair (in copulatory pouches, associated with paired retractile claspers), in segment XVI, rarely XV, opisthoporous (two or more segments following testicular segment). Prostate gland present, tubular, one pair. Prostate pores in segments XII to XIV; prostate pore and male pore on segment XVII united, discharge through single pore.

**Literature used for description**. Jamieson, BGM (2006) Non-leech Clitellata (with contributions by Marco Ferraguti). Pp. 235–392. In Reproductive Biology and Phylogeny of Annelida. Series Editor BGM Jamieson. Volume 4. Editors G Rouse, F Pleijel. Science Publishers, Enfield, New Hampshire; Misirlioðlu, M, Reynolds, JW, Stojanoviæ, M, Trakiæ, T, Sekuliæ, J, James, SW, Csuzdi, C, Decaëns, T, Lapied, E, Phillips, HRP, Cameron, EK, Brown, GG (2023). Earthworms (Clitellata, Megadrili) of the world: an updated checklist of valid species and families, with notes on their distribution. Zootaxa 5255 (1): 417–438.

# Lacydoniidae Bergström, 1914

**Description**. Body segment number: variable (but relatively few segments). Body shape elongate, more-or-less equal width over entire length. Body translucent, gut visible, pigmentation absent or present, pigmentation posterior body may have pigment spots. Pygidium simple lobe. Pygidial appendages present, one pair of cirri and single medial papilla. Discrete head present; complex in shape bearing appendages.

Prostomium rounded to oval, eyes on head absent or present, one pair or two pairs; head eyes simple pigmented cups, or compound, with lenses. Prostomial antennae present; median and paired laterals (antennae may be very small, meaning the median one is easily overlooked); unarticulated. Palps present (resemble antennae); anteroventral; tapering (usually) sensory type; unarticulated. Nuchal organs present; paired low projections from posterolateral prostomium; indistinct dorsolateral ciliated patches. Peristomium not visible.

Gut more-or-less straight, lacking side branches. Foregut a muscular axial pharynx.

Protrusible proboscis present, smooth, distal ring of papillae present (?).

First segment tentaculate (a few chaetae may bear a small tentacular cirrus); second segment chaetous. Tentacular cirri present; 1 pair(s); arise on a single segment; internal aciculae absent. First chaetiger with neurochaetae only; parapodia similar in length or slightly shorter than subsequent parapodia; parapodia more-or-less laterally directed and free from head; chaetae similar in orientation, length and thickness to other chaetae. Parapodia biramous (from segment 5); parapodial lobes prominent. Notopodial lobes represented by at least one chaetal lobe. Neuropodial lobes represented by at least one chaetal lobe. Dorsal cirri present; flattened and foliaceous (slightly). Ventral cirri present; cirriform or tapering.

Chaetae first appear on first segment after peristomium or second segment after peristomium, arranged in paired bundles (or rows) of many chaetae. Aciculae present; in both dorsal and ventral positions (=noto- and neuroaciculae). Neuroaciculae distally tapering. Capillary chaetae present, in dorsal (notopodial) position, distally tapered to a point, edge smooth or hirsute-serrate. Capillary chaetae, externally not pseudo-segmented. Capillary chaetae, internally not chambered or hollow. Hooks absent. Compound chaetae present; in ventral (neuropodial) position; appendage distally tapering to slender tips (=spinigerous); appendage not canaliculated; appendage without hoods or guards; joint, appearance distinctly asymmetrical (=heterogomph); joint effected by ligament(s); shaft tapering slightly or evenly thick from emergence to joint; shaft, internally solid, without distinct core. Compound chaetae present in most or all chaetigers.

Segmental organs are protonephridia. Tube absent.

**Literature used for description**. Glasby CJ, Fauchald K (2003). POLiKEY. An information system for polychaete families and higher taxa. Version 2, 5 June 2003. Australian Biological Resources Study, DCCEEW, Canberra, https:[//www.dcceew.gov.au/;](http://www.dcceew.gov.au/%3B) Rizzo AE, Magalha~es, WF, Santos CSG (2015) Lacydoniidae Bergström, 1914 (Polychaeta) in the South Atlantic: morphology, three new species and five new records. Journal of the Marine Biological Association of the United Kingdom, DOI: 10.1017/S0025315415001381, pp 1–21; Rizzo AE, Magalhães WF (2022) 7.13.6 Lacydoniidae Bergström, 1914 In: Purschke G, Böggemann, M, Westheide W (Eds) Handbook of Zoology. Annelida. Vol. 4: Pleistoannelida, Errantia II. De Gruyter, Berlin, 368–375; Rouse GW, Pleijel, F, Tilic E (2022) Annelida. Oxford University Press, Oxford, UK.

# Lobatocerebridae Rieger, 1980

**Description**. Body segmentation absent. Body shape elongate, more-or-less equal width over entire length. Body translucent, gut visible, pigmentation present, pigmentation speckled appearance as result of green epidermal glands.

Epidermis more-or-less smooth. Dorsal body surface, appearance under compound microscope ciliated. Anus subterminal (dorsal). Pygidium absent. Elongate proboscis absent. Discrete head absent.

Nuchal organs absent. Peristomium not visible. Gut more-or-less straight, lacking side branches. Foregut without a distinct ventral or axial pharynx. Pharynx jaws absent. Protrusible proboscis absent. Gizzard absent. Calciferous glands absent.

Parapodia absent. Chaetae absent.

Sexual reproduction hermaphroditic. Testes present, single (sac-like, opens in a mid-dorsal gonopore). Segmental organs are protonephridia (3 pairs).

Tube absent.

**Literature used for description**. Kerbl A, Worsaae K (2019) 6.2. Lobatocerebridae Rieger, 1980, In Annelida Volume 1: Annelida Basal Groups and Pleistoannelida, Sedentaria I, Purschke G, Böggemann M, Westheide W (Eds), Handbook of Zoology. Boston: De Gruyter, 201–215; Rouse GW, Pleijel, F, Tilic E (2022) Annelida. Oxford University Press, Oxford, UK.

# Longosomatidae Hartman, 1944

**Description**. Body segment number: variable. Body segments strongly elongate in midbody, elongate body segments with indistinct parapodia, chaetae arise directly from body wall (nearly complete circlets of spines). Body shape elongate, more-or-less equal width over entire length, regionalization present, regionalization comprising three regions (first 6–8 segments short and generally branchiate; following 8–14 segments are very elongated; last segments are short and inflated), regions demarcated by change in chaetal types over body or structural differences in parapodia over body (segment length). Epidermis more-or-less smooth. Pygidium simple lobe. Pygidial appendages absent. Discrete head present; complex in shape bearing appendages.

Prostomium bluntly conical. Palps present; anterodorsal; grooved (usually) feeding type; feeding palp longitudinally grooved. Nuchal organs present; paired low projections from posterolateral prostomium; posterior projections. Peristomium a single ring.

Foregut a muscular ventral pharynx.

First segment chaetous; second segment chaetous. First chaetiger with both notochaetae and neurochaetae; parapodia similar in length or slightly shorter than subsequent parapodia; parapodia more-or-less laterally directed and free from head; chaetae similar in orientation, length and thickness to other chaetae. Parapodia biramous; parapodial lobes prominent. Notopodial lobes represented by at least one chaetal lobe. Neuropodial lobes represented by at least one chaetal lobe. Branchiae present, arise from lateral body, occur first few segments and occur on mid-body segments; lateral branchiae digitiform.

Chaetae first appear on first segment after peristomium, arranged in paired bundles (or rows) of many chaetae. Aciculae absent. Capillary chaetae present, in dorsal (notopodial) position or in ventral (neuropodial) position, distally tapered to a point, edge smooth or hirsute-serrate. Capillary chaetae, externally not pseudo-segmented. Capillary chaetae, internally not chambered or hollow. Spines present; slightly curved and more-or-less smooth, or with a single distal or subdistal hair (=aristate or hooded); present in most or all chaetigers; in ventral (neuropodial) position only. Hooks absent, or present (rarely in the neuropodia of segment 1).

Segmental organs are metanephridia; metanephridia single anterior pair of excretory metanephridia and several more posterior ones for gamete release (excretory ones on segment 1).

Tube present; membranous.

**Literature used for description**. Glasby CJ, Fauchald K (2003). POLiKEY. An information system for polychaete families and higher taxa. Version 2, 5 June 2003. Australian Biological Resources Study, DCCEEW, Canberra, https:[//www.dcceew.gov.au/;](http://www.dcceew.gov.au/%3B) Blake JA, Maciolek NJ (2019) 7.3.1.9 Longosomatidae Hartman, 1944 In: Purschke G, Böggemann M, Westheide W (Eds) Handbook of Zoology, Annelida, Volume 1: Annelida Basal Groups and Pleistoannelida, Sedentaria I. De Gruyter, Berlin, 457–466; Rouse GW, Pleijel, F, Tilic E (2022) Annelida. Oxford University Press, Oxford, UK.

# Lopadorrhynchidae Claparède, 1870

**Description**. Body segment number: fixed or variable, less than 14 segments (rarely). Body shape elongate, more-or-less equal width over entire length, dorsoventrally flattened. Body translucent, gut visible, pigmentation present, pigmentation one species has dark transparent bands and parapodial pigment spots; epidermis more-or-less smooth. Pygidium simple lobe. Pygidial appendages absent or present, one pair of cirri. Discrete head present; complex in shape bearing appendages.

Prostomium rounded to oval, eyes on head absent or present, one pair; head eyes simple pigmented cups, or compound, with lenses. Prostomial antennae present; paired, lateral; unarticulated. Palps present (resemble antennae); anteroventral; tapering (usually) sensory type; unarticulated. Nuchal organs present; paired low projections from posterolateral prostomium; posterolateral ciliated bulbs. Peristomium not visible.

Gut more-or-less straight, lacking side branches. Foregut a muscular axial pharynx. Pharynx jaws present, one pair of lateral jaws. Paired jaws fang-like (stylet type). Protrusible proboscis present, distal ring of papillae present (?).

First segment tentaculate; second segment chaetous, or tentaculate. Tentacular cirri present; 2–3 pair(s); arise on a single segment, or over two or more segments; internal aciculae absent. First chaetiger with neurochaetae only; parapodia similar in length or slightly shorter than subsequent parapodia; parapodia more-or-less laterally directed and free from head; chaetae similar in orientation, length and thickness to other chaetae. Parapodia uniramous. Notopodial lobes absent (though dorsal cirri are present). Neuropodial lobes represented by at least one chaetal lobe. Dorsal cirri present; flattened and foliaceous (usually slender leaf-like, rarely digitate). Ventral cirri present; cirriform or tapering.

Chaetae first appear on first segment after peristomium or second segment after peristomium or third segment after peristomium, arranged as a single bundle (or row) of many chaetae (lower bundle - neurochaetae - only). Aciculae present; in ventral position (=neuroaciculae). Capillary chaetae absent. Spines absent, or present; slightly curved and more-or-less smooth; present in most or all chaetigers (in segments 3–4 or 3–5 in *Lopadorrynchus*); in ventral (neuropodial) position only. Hooks absent. Compound chaetae present; in ventral (neuropodial) position; appendage distally tapering to slender tips (=spinigerous) (may be broad, oar-shaped, subdistally); appendage not canaliculated; appendage without hoods or guards; joint, appearance distinctly asymmetrical (=heterogomph); joint effected by ligament(s); shaft tapering slightly or evenly thick from emergence to joint; shaft, internally solid, without distinct core. Compound chaetae present in most or all chaetigers.

Segmental organs are protonephridia. Tube absent.

**Literature used for description**. Glasby CJ, Fauchald K (2003). POLiKEY. An information system for polychaete families and higher taxa. Version 2, 5 June 2003. Australian Biological Resources Study, DCCEEW, Canberra, https:[//www.dcceew.gov.au/;](http://www.dcceew.gov.au/%3B) Rouse GW, Pleijel, F, Tilic E (2022) Annelida. Oxford University Press, Oxford, UK.

# Lumbricidae Rafinesque-Schmaltz, 1815

**Description**. Body segment number: variable. Secondary annulation absent or present; mid-body triannulate. Body shape elongate, more-or-less equal width over entire length. Body pigmentation absent or present, greenish or brownish base pigment or banded. Bioluminescence absent, or present (only *Eisenia* species). Dorsal intersegmental furrow present, pores on mid-dorsal line present (usually, 1 row). Pygidium simple lobe. Pygidial appendages absent.

Prostomium bluntly conical.

Gut straight with side branches. Foregut a muscular dorsal pharynx. Gizzard present, in segments XVII-XX. Calciferous glands present. Intestinal typhlosole present, formed from all layers of the intestine. Heart bodies present.

Chaetae first appear on first segment after peristomium (dorsal and ventral bundles; =S2 for oligochaete workers), in lumbricine arrangement - paired upper and lower bundles (usually 2 chaetae per bundle) - closely spaced lateral and ventrolateral pairs or arranged in widely spaced lateral and ventrolateral pairs. Crotchet chaetae present, simple-pointed (straight and blunt, hardly protruding from body wall). Genital chaetae present.

Sexual reproduction hermaphroditic. Clitellum thick, multilayered (often long, extending over many segments), partially encircles body, posterior to male pores, in region of female pores (*Eiseniella*) or posterior to female pore(s). Clitellum occurs from XVII to LII. Seminal groove present (extend from male pore to far posterior clitellum). Tubercula pubertatis present (on clitellar segments). Gonadal segments bearing genital papillae absent, or present (*Diporodrilus* for example). Testes present, two pairs in total (rarely 1 pair), one pair per segment; present in segments X and XI. Sperm sac absent, or present. Ovaries present, one pair; present in segments XIII. Segmental organs are metanephridia; both nephridial pores and gonoducts located around clitellum, one pair nephridia in each segment (holonephridia). Spermathecae present, simple, pre-testicular or in testicular segments. Spermathecal pores absent or present (often several pairs), located within 1 or 2 segments of male pores or located well anterior to male pores; 2 pairs (or more); in segment VIII-XI, commonly, but variable. Female gonoduct absent or present. Female pores one pair, in segment XIV (inconspicuous, XXV in *Eiseniella)*. Male gonoducts present. Male pores one pair (conspicuous, often on glandular swellings), in segment XV, rarely XII to XIV, opisthoporous (two or more segments following testicular segment). Prostate gland absent (usually).

**Literature used for description**. Jamieson, BGM (2006) Non-leech Clitellata (with contributions by Marco Ferraguti). Pp. 235–392. In Reproductive Biology and Phylogeny of Annelida. Series Editor BGM Jamieson. Volume 4. Editors G Rouse, F Pleijel. Science Publishers, Enfield, New Hampshire; Timm, T (2009) A guide to the freshwater Oligochaeta and Polychaeta of Northern and Central Europe. Lauterbornia 66: 1–235; Blakemore, RJ (2009) Cosmopolitan earthworms – a global and historical perspective. In: Shain, D.H. (Ed.), Annelids as Model Systems in the Biological Sciences. John Wiley & Sons, New York, pp. 257–283, https://doi.org/10.1002/9780470455203.ch14; Plisko JD, Nxele, TC (2015) An Annotated Key Separating Foreign Earthworm Species from the Indigenous South African taxa (Oligochaeta: Acanthodrilidae, Eudrilidae, Glossoscolecidae, Lumbricidae, Megascolecidae, Microchaetidae, Ocnerodrilidae and Tritogeniidae). African Invertebrates 56:663–708; Thorp JH, Lovell LL, Timm T, Martin P, Gelder SR, Govedich FR, Moser WE, Nakano T, Bielecki A, Bain BA, Utevsky S, Gil J, Glasby CJ, Martin D (2019) Phylum Annelida. In: Rogers DC, Thorp JH (Eds) Keys to Palaearctic Fauna: Thorp and Covich’s Freshwater invertebrates - Volume IV. Academic Press, Elsevier, pp. 357–518; Misirlioðlu, M, Reynolds, JW, Stojanoviæ, M, Trakiæ, T, Sekuliæ, J, James, SW, Csuzdi, C, Decaëns, T, Lapied, E, Phillips, HRP, Cameron, EK, Brown, GG (2023). Earthworms (Clitellata, Megadrili) of the world: an updated checklist of valid species and families, with notes on their distribution. Zootaxa 5255 (1): 417–438; Csuzdi C, James SW, Lapied E (2023) Drilobase. World Earthworm Database. Taxonomy section. <http://taxo.drilobase.org/> (Accessed 28 July 2023).

# Lumbriculidae Claus, 1872

**Description**. Body segment number: variable. Secondary annulation absent or present (usually). Body shape elongate, more-or-less equal width over entire length (small). Body pigmentation absent or present, sometimes with brown bands. Pygidium simple lobe. Pygidial appendages absent.

Prostomium conical, tapering to slender tip (rarely) or bluntly conical, prolobic (prostomium demarcated from peristomium without a tongue) or zygolobic (prostomium not demarcated).

Foregut a muscular dorsal pharynx. Heart bodies present, segment XVIII (and more posteriorly).

Chaetae absent or present, two per bundle, first appear on first segment after peristomium (at least ventral ones; dorsal bundles may start on segment 2), lumbricine arrangement, closely spaced lateral and ventrolateral pairs. Capillary (=hair) chaetae absent. Crotchet chaetae present (rarely absent), simple or bifid (rarely, shorter upper tooth; always with nodulus). Genital chaetae absent, or present (rarely).

Reproduction sexual.

Sexual reproduction hermaphroditic. Clitellum thin, single layered, in region of male pores (male pores on first clitellar segment), and female pores. Clitellum occurs from VIII to X for several segments. Tubercula pubertatis absent. Gonadal segments bearing genital papillae absent, or present (rarely). Testes present, one pair in total or two pairs in total (rarely more pairs), one pair per segment; present in segments (VIII) IX-X (XI). Sperm sac present (extend for many segments posterior to testes). Ovaries present, one pair or two pairs; present in segments (X) XI or XII (XIII). Segmental organs are metanephridia (may be missing in some segments), nephridial pores located posterior to gonoducts. Spermathecae present, post-testicular and pre-testicular. Spermathecal pores present, located within 1 or 2 segments of male pores (in segments immediately before or/and after male pores); variable number pairs; in segment VII-IX or XIII-XVI. Female gonoduct present. Female pores one pair or two pairs, in segment XI-XIII. Male gonoducts present, with atrium present. Male pores one pair or two pairs (rarely more pairs), in segment VI to XI, prosoporous (same segment as corresponding testes) or plesioporous (in segment following testicular segment). Penis absent or present (mature worms of *Stylodrilus* have permanently protruding penes). Prostate gland absent.

**Literature used for description**. Brinkhurst RO and Jamieson, BGM (1971) Aquatic Oligochaeta of the World with contributions by DG Cook, DV Anderson, J van der Land, University of Toronto Press, Toronto, Canada, 860 pp; Jamieson, BGM (2006) Non-leech Clitellata (with contributions by Marco Ferraguti). Pp. 235–392. In Reproductive Biology and Phylogeny of Annelida. Series Editor BGM Jamieson. Volume 4. Editors G Rouse, F Pleijel. Science Publishers, Enfield, New Hampshire; Martin, P, Martinez-Ansemil, E, Pinder, A, Timm, T, Wetzel, MJ (2008) Global diversity of oligochaetous clitellates (‘‘Oligochaeta’’; Clitellata) in freshwater. Hydrobiologia (2008) 595:117–127; Martin P, Kaygorodova I (2008) A new species of Pseudorhynchelmis Hrabě, 1982 (Clitellata: Lumbriculidae) from Lake Baikal, with re-descriptions of P. parva and P. olchonensis. Zootaxa 1938: 21; Timm, T (2009) A guide to the freshwater Oligochaeta and Polychaeta of Northern and Central Europe. Luterbornia 66: 1–235. Pinder, A (2013). Tools for identifying Australian aquatic oligochaetes of the families Phreodrilidae, Lumbriculidae and Capilloventridae (Clitellata: Annelida). Museum Victoria Science Reports 18: 1–20; Thorp JH, Lovell LL, Timm T, Martin P, Gelder SR, Govedich FR, Moser WE, Nakano T, Bielecki A, Bain BA, Utevsky S, Gil J, Glasby CJ, Martin D (2019) Phylum Annelida. In: Rogers DC, Thorp JH (Eds) Keys to Palaearctic Fauna: Thorp and Covich’s Freshwater invertebrates - Volume IV. Academic Press, Elsevier, pp. 357–518.

# Lumbrineridae Schmarda, 1861

**Description**. Body segment number: variable. Body shape elongate, more-or-less equal width over entire length. Epidermis more-or-less smooth (cuticle is iridescent). Pygidium simple lobe. Pygidial appendages absent or present, one pair of cirri or four cirri. Discrete head present; lobe-like without appendages (1–3 very small antennae/papilla may be present in the groove between the prostomium and peristomium).

Prostomium bluntly conical. Prostomial antennae absent, or present; median and paired laterals (papilla-like); unarticulated. Nuchal organs present; paired low projections from posterolateral prostomium; indistinct dorsolateral ciliated patches. Peristomium a double ring.

Gut more-or-less straight, lacking side branches. Foregut a muscular ventral pharynx. Pharynx jaws present, multiple jaw elements of different shapes and sizes. Multiple jaws comprising ventral mandibles and dorsal maxillae, 4–5 (right) and 4–6 (left) toothed plates in a semicircle. Pharynx maxillae symmetrical. Maxillary carriers shorter than combined length of maxillae. Pharynx dorsolateral ciliated folds present.

First segment chaetous; second segment chaetous. First chaetiger with neurochaetae only; parapodia similar in length or slightly shorter than subsequent parapodia; parapodia more-or-less laterally directed and free from head; chaetae similar in orientation, length and thickness to other chaetae. Parapodia uniramous (anteriorly), or biramous; parapodial lobes prominent. Notopodial lobes represented by at least one chaetal lobe, or absent (usually). Neuropodial lobes represented by at least one chaetal lobe. Lateral organs present (ciliated patch at ventral base of dorsal cirrus). Dorsal cirri absent, or present; flattened and foliaceous. Ventral cirri absent (replaced by inflated pads). Branchiae absent (rarely) or present, arise from lateral body (posterior face), occur on mid-body segments; lateral branchiae digitiform (one or more per parapodium).

Chaetae first appear on first segment after peristomium, arranged as a single bundle (or row) of many chaetae (lower bundle - neurochaetae - only). Aciculae present; in both dorsal and ventral positions (=noto- and neuroaciculae) (dorsal one sometimes absent). Capillary chaetae present, in ventral (neuropodial) position, distally tapered to a point, edge smooth (limbate). Capillary chaetae, externally not pseudo-segmented. Capillary chaetae, internally not chambered or hollow. Hooks present; with a distal hood; occur in ventral (neuropodial) position; occur over entire body. Compound chaetae absent, or present; in ventral (neuropodial) position; appendage multidentate; appendage not canaliculated; appendage with a single hood open in front; joint, appearance distinctly asymmetrical (=heterogomph); joint effected by ligament(s); shaft tapering slightly or evenly thick from emergence to joint; shaft, internally solid, without distinct core.

Tube absent, or present; membranous.

**Literature used for description**. Glasby CJ, Fauchald K (2003). POLiKEY. An information system for polychaete families and higher taxa. Version 2, 5 June 2003. Australian Biological Resources Study, DCCEEW, Canberra, https://[www.dcceew.gov.au/;](http://www.dcceew.gov.au/%3B) Oug, E, Borisova, P, Budaeva N (2022) 7.12.6. Lumbrineridae Schmarda, 1861, In: Purschke G, Böggemann M, Westheide W (Eds), Handbook of Zoology, Annelida, Volume 3: Pleistoannelida, Errantia II. De Gruyter, Berlin, 1–35; Rouse GW, Pleijel, F, Tilic E (2022) Annelida. Oxford University Press, Oxford, UK.

# Lutodrilidae McMahan, 1976

**Description**. Body segment number: variable. Body shape elongate, more-or-less equal width over entire length (quadrangular posteriorly). Dorsal intersegmental furrow present (?), pores on mid-dorsal line present (?). Pygidium simple lobe. Pygidial appendages absent.

Prostomium bluntly conical.

Gut straight with side branches. Foregut a muscular dorsal pharynx. Gizzard present (?). Calciferous glands present. Intestinal typhlosole present. Heart bodies present, segments XI-XV.

.

Chaetae first appear on first segment after peristomium (=S2 for oligochaete workers), in lumbricine arrangement - paired upper and lower bundles of 2 chaetae each, - closely spaced lateral and ventrolateral pairs. Crotchet chaetae present, simple-pointed, with jagged transverse ridges below curved portion. Genital chaetae present. Sexual reproduction hermaphroditic. Clitellum thick, multilayered (often long, extending over many segments), fully encircles body, in region of male and female pores. Clitellum occurs from XX to LXXI. Tubercula pubertatis present. Gonadal segments bearing genital papillae absent. Gonadal segments with extensions of the body wall present, as alae (alae on segments VVII–LIII). Testes present, many pairs (9 or 10 pairs), one pair per segment; present in segments XII-XXI. Sperm sac absent. Ovaries present, one pair; present in segment XXIII. Segmental organs are metanephridia; both nephridial pores and gonoducts located around clitellum. Spermathecae present. Spermathecal pores absent (not visible externally), located within 1 or 2 segments of male pores or located well anterior to male pores; 4 pairs (or more); in segment XV to XXVI. Female gonoduct present. Female pores one pair, in segment XXIV. Male gonoducts present. Male pores one pair, in segment XX or later; in intersegmental groove, opisthoporous (two or more segments following testicular segment). Prostate gland absent.

**Literature used for description**. McMahan, ML (1979) Anatomical notes on *Lutrodrilus multivesiculatus* (Annelida: Oligochaeta). Proceedings of the Biological Society of Washington 92(1): 84-97; Jamieson, BGM (2006) Non-leech Clitellata (with contributions by Marco Ferraguti). Pp. 235–392. In Reproductive Biology and Phylogeny of Annelida. Series Editor BGM Jamieson. Volume 4. Editors G Rouse, F Pleijel. Science Publishers, Enfield, New Hampshire; Thorp JH, Lovell LL, Timm T, Martin P, Gelder SR, Govedich FR, Moser WE, Nakano T, Bielecki A, Bain BA, Utevsky S, Gil J, Glasby CJ, Martin D (2019) Phylum Annelida. In: Rogers DC, Thorp JH (Eds) Keys to Palaearctic Fauna: Thorp and Covich’s Freshwater invertebrates - Volume IV. Academic Press, Elsevier, pp. 357–518.

# Magelonidae Cunningham & Ramage, 1888

**Description**. Body segment number: variable. Body shape elongate, more-or-less equal width over entire length, regionalization present (change in chaetae approximating a thorax-abdomen boundary), regionalization comprising two regions, regions demarcated by change in chaetal types over body. Epidermis more-or-less smooth. Pygidium simple lobe. Pygidial appendages present, one pair of cirri. Discrete head present; complex in shape bearing appendages.

Prostomium flattened, shovel-shaped (may be a pair of 'horns' on anterior edge). Palps present; ventral (immediately behind and lateral to the mouth); grooved (usually) feeding type (papillated rather than grooved); feeding palp papillated. Nuchal organs absent. Peristomium not visible.

Foregut without a distinct ventral or axial pharynx. Pharynx dorsolateral ciliated folds present.

First segment achaetous (may comprise two fused achaetous segments); second segment chaetous. First chaetiger with both notochaetae and neurochaetae; parapodia similar in length or slightly shorter than subsequent parapodia; parapodia more-or-less laterally directed and free from head; chaetae similar in orientation, length and thickness to other chaetae. Parapodia biramous; parapodial lobes prominent. Notopodial lobes represented by at least one chaetal lobe (lamellate lobe). Neuropodial lobes represented by at least one chaetal lobe (lamellate lobe). Lateral organs present. Dorsal cirri absent (although tiny dorsal medial lobes on abdominal segments resemble a reduced dorsal cirrus). Ventral cirri absent (although tiny ventral medial lobes on abdominal segments resemble a reduced ventral cirrus).

Chaetae first appear on second segment after peristomium (assuming the achaetous segment is single), arranged in paired bundles (or rows) of many chaetae. Aciculae absent. Capillary chaetae present (in thoracic chaetigers), in dorsal (notopodial) position or in ventral (neuropodial) position, distally tapered to a point and expanded (subdistally), edge smooth. Capillary chaetae, externally not pseudo-segmented. Capillary chaetae, internally not chambered or hollow. Hooks present (uni-, bi-, or tridentate; in abdominal chaetigers); with a distal hood; occur in dorsal (notopodial) position, or occur in ventral (neuropodial) position; occur in anterior body only.

# Segmental organs are protonephridia.

# Tube absent.

**Literature used for description**. Glasby CJ, Fauchald K (2003). POLiKEY. An information system for polychaete families and higher taxa. Version 2, 5 June 2003. Australian Biological Resources Study, DCCEEW, Canberra, https:[//www.dcceew.gov.au/;](http://www.dcceew.gov.au/%3B) Mortimer K (2019) 4.2 Magelonidae Cunningham & Ramage, 1888 In Purschke G, Böggemann M, Westheide W (Eds), Handbook of Zoology, Annelida, Volume 1: Annelida Basal Groups and Pleistoannelida, Sedentaria I. De Gruyter, Berlin, 112–131; Rouse GW, Pleijel, F, Tilic E (2022) Annelida. Oxford University Press, Oxford, UK.

# Maldanidae Malmgren, 1867

**Description**. Body segment number: variable. Body segments strongly elongate in midbody, elongate body segments with distinct (but truncate) notopodia and neuropodial tori. Body shape elongate, more-or-less equal width over entire length. Body pigmentation absent or present, middle segments often banded orange to dark red; epidermis more-or-less smooth. Anus positioned at posterior body (terminal or dorso-terminal). Pygidium simple lobe (may form a funnel or have multiple lobes), or plate-like. Pygidial appendages absent or present, more than four cirri (range in length from simple crenulations to cirri that are longer than pygidial segment; often variable lengths). Discrete head present; complex in shape bearing appendages.

Prostomium narrow, keel- or ridge-shaped (formed from the prostomium and peristomium; a central 'cephalic keel' may be surrounded by a raised 'cephalic rim'), eyes on head present, one pair or numerous, unpaired; head eyes simple pigmented cups. Nuchal organs present; paired low projections from posterolateral prostomium; indistinct dorsolateral ciliated patches (on either side of median cephalic keel). Peristomium a single ring, or a double ring (rarely).

Foregut a non-muscular axial pharynx or a muscular ventral pharynx. Pharynx dorsolateral ciliated folds absent or present.

First segment chaetous. First chaetiger with notochaetae only, or with neurochaetae only, or with both notochaetae and neurochaetae; parapodia similar in length or slightly shorter than subsequent parapodia; parapodia more-or-less laterally directed and free from head; chaetae similar in orientation, length and thickness to other chaetae. Parapodia biramous; parapodial lobes absent or very low. Notopodial lobes represented by at least one chaetal lobe. Neuropodial lobes low ridges (tori) (over much of body). Lateral organs present.

Chaetae first appear on first segment after peristomium, arranged in paired bundles (or rows) of many chaetae. Aciculae absent. Capillary chaetae present, in dorsal (notopodial) position, distally tapered to a point, edge smooth or hirsute-serrate or spinose. Capillary chaetae, externally not pseudo-segmented. Capillary chaetae, internally not chambered or hollow. Spines absent, or present; slightly curved and more-or-less smooth; present only in one or a few anterior chaetigers; in ventral (neuropodial) position only. Hooks present (long handled); with a subdistal beard (usually); occur in ventral (neuropodial) position; occur over entire body (neuropodia). Uncini present; with teeth arranged in transverse series above an enlarged main fang (=crested); in ventral (neuropodial) position throughout; arranged in one row, or arranged in two rows.

Segmental organs are metanephridia; metanephridia several pairs metanephridia anteriorly for excretion, posterior ones for gamete release (4–7 pairs).

Tube present; membranous (often covered with mud, shell or sand).

**Literature used for description**. Glasby CJ, Fauchald K (2003). POLiKEY. An information system for polychaete families and higher taxa. Version 2, 5 June 2003. Australian Biological Resources Study, DCCEEW, Canberra, https:[//www.dcceew.gov.au/;](http://www.dcceew.gov.au/%3B) De Assis JE, Bleidorn C, Christoffersen ML (2021) 7.7.6 Maldanidae Malmgren, 1867 In: Purschke G, Böggemann M, Westheide W (Eds), Handbook of Zoology, Annelida, Volume 3: Pleistoannelida, Sedentaria III and Errantia I. De Gruyter, Berlin, 186–201; Rouse GW, Pleijel, F, Tilic E (2022) Annelida. Oxford University Press, Oxford, UK.

# Megascolecidae Rosa, 1891

**Description**. Body segment number: variable. Body shape elongate, more-or-less equal width over entire length. Body pigmentation absent or present, often dark brown or even black, with lighter coloured clitellum. Bioluminescence absent, or present. Dorsal intersegmental furrow present (usually), pores on mid-dorsal line present. Pygidium simple lobe. Pygidial appendages absent.

Prostomium bluntly conical.

Gut straight with side branches. Foregut a muscular dorsal pharynx. Gizzard present (one to three, rarely vestigial or absent). Calciferous glands absent or present, in segment IX-X (but not restricted to these segments). Intestinal typhlosole present, formed from the inner (epithelial) layer only of the intestine. Caeca of foregut absent (or rare). Caeca of midgut (=posterior crop caeca) absent (or rare). Caeca of hindgut absent (or rare). Heart bodies present, posterior to segment XI.

Chaetae first appear on first segment after peristomium (=S2 for oligochaete workers), in lumbricine arrangement - paired upper and lower bundles of one or a few chaetae each, or in perichaetine arrangement - chaetae more-or-less evenly distributed around perimeter of segment (more than 8 chaetae per segment). Crotchet chaetae present, simple-pointed. Genital chaetae present.

Sexual reproduction hermaphroditic. Clitellum thick, multilayered, partially encircles body or fully encircles body, anterior to male pores, in region of female pores. Clitellum occurs from XII to XX. Seminal groove absent, or present. Tubercula pubertatis present. Gonadal segments bearing genital papillae present. Testes present, two pairs in total (usually), one pair per segment (sometimes enclosed in testis sacs); present in segments X and XI. Ovaries present, one pair; present in segments XIII, rarely XII and XIII. Segmental organs are metanephridia; both nephridial pores and gonoducts located around clitellum, one pair nephridia in each segment (holonephridia) (rarely) or multiple, minute, nephridia in each segment (meronephridia). Spermathecae present, with a basal diverticula (one or more), pre-testicular. Spermathecal pores absent or present (often several pairs), paired (usually), located within 1 or 2 segments of male pores or located well anterior to male pores; variable number pairs (usually 4-5 pairs); in segment VI-IX. Female gonoduct present. Female pores one pair, in segment XIV, midventral. Male gonoducts present. Male pores one pair (rarely single or two pairs), in segment XVIII mostly, rarely XVII to XIX; absent in parthenogenetic species, opisthoporous (two or more segments following testicular segment). Prostate gland present, tubular, one to three pairs. Prostate pores in segments XVII to XIX, or XVIII, ventral; prostate pore and male pore on segment XVIII united, discharge through single pore.

**Literature used for description**. Easton EG (1984) Earthworms (Oligochaeta) from islands of the south-western Pacific, and a note on two species from Papua New Guinea. New Zealand Journal of Zoology 11: 111–128; Dyne GR, Jamieson BGM (2004) Native Earthworms of Australia II (Megascolecidae, Acanthodrilinae). Department of the Environment and Heritage, Australian Government, 200 pp; Jamieson, BGM (2006) Non-leech Clitellata (with contributions by Marco Ferraguti). Pp. 235–392. In Reproductive Biology and Phylogeny of Annelida. Series Editor BGM Jamieson. Volume 4. Editors G. Rouse, F. Pleijel. Science Publishers, Enfield, New Hampshire; Martin, P, Martinez-Ansemil, E., Pinder, A, Timm, T, Wetzel, MJ (2008) Global diversity of oligochaetous clitellates (‘‘Oligochaeta’’; Clitellata) in freshwater. Hydrobiologia (2008) 595:117–127; James SW, Davidson SK (2012) Molecular phylogeny of earthworms (Annelida : Crassiclitellata) based on 28S, 18S and 16S gene sequences. Invertebrate Systematics 26: 213–229; Plisko, JD, Nxele, TC (2015) An annotated key separating foreign earthworm species from the indigenous South Africian taxa (Oligochaeta: Acanthodrilidae, Eudrilidae, Glossoscolecidae, Lumbricidae, Megascolecidae, Microchaetidae, Ocnerodrilidae and Tritogeniidae). African Invertebrates 56: 663–708; Anderson FE, Williams BW, Horn KM, Erséus C, Halanych KM, Santos SR, James SW (2017) Phylogenomic analyses of Crassiclitellata support major Northern and Southern Hemisphere clades and a Pangaean origin for earthworms. BMC Evolutionary Biology 17: 123; Thorp JH, Lovell LL, Timm T, Martin P, Gelder SR, Govedich FR, Moser WE, Nakano T, Bielecki A, Bain BA, Utevsky S, Gil J, Glasby CJ, Martin D (2019) Phylum Annelida. In: Rogers DC, Thorp JH (Eds) Keys to Palaearctic Fauna: Thorp and Covich’s Freshwater invertebrates - Volume IV. Academic Press, Elsevier, pp. 357–518.

# Melinnidae Chamberlin, 1919

**Description**. Body segment number: variable. Body shape widest anteriorly and tapering posteriorly, regionalization present, regionalization comprising two regions (thorax and abdomen), regions demarcated by absence of abdominal notopodia. Body pigmentation absent or present, dorsal body may have speckled pigmentation; epidermis more-or-less smooth. Thoracic lobe-like dorsolateral expansion present (collar-like lobe over the first five segments enclosing the branchial bases). Thoracic ventral glandular areas present; indistinct mid-ventral swelling. Pygidium simple lobe. Pygidial appendages present, one pair of cirri or more than four cirri. Discrete head present; complex in shape bearing appendages.

Prostomium hood-like, covering the tentacles dorsally (tentacles often retracted), eyes on head absent or present, numerous, unpaired; head eyes simple pigmented cups. Buccal tentacles present (several tentacles often retracted into buccal cavity, especially the smaller ones; rarely only one tentacle present); arising inside mouth; grooved. Nuchal organs present; paired low projections from posterolateral prostomium; indistinct dorsolateral ciliated patches. Peristomium a single ring, may carry lobes.

Gut straight except for a large midbody loop. Foregut a muscular ventral pharynx. Heart bodies present (for *Melinna palmata*; associated with the dorsal blood vessel).

First segment achaetous; second segment chaetous (parapodia reduced; neuropodial spines emerge directly from body wall). First chaetiger with notochaetae only; parapodia similar in length or slightly shorter than subsequent parapodia; parapodia more-or-less laterally directed and free from head; chaetae similar in orientation, length and thickness to other chaetae. Parapodia biramous (most of thorax), or uniramous (abdomen, although relictual notopodia often present); parapodial lobes prominent. Notopodial lobes represented by at least one chaetal lobe. Neuropodial lobes low ridges (tori) (after anteriormost segments). Lateral organs present. Branchiae present, arise from dorsal body, occur first few segments (branchiae crowded together so segmental position difficult to determine; appear to be from segments 2 and 3 but actually from segments 2–5). Dorsal branchiae multiple filaments arising from a central stalk, or simple filaments each arising directly from body wall (2–4 pairs of tapering branchiae which vary from smooth to crenulated to pinnate).

Chaetae first appear on second segment after peristomium, present along most of body (anterior ones displaced dorsally), arranged in paired bundles (or rows) of many chaetae. Aciculae absent. Capillary chaetae present (from segment 4; first pairs very small), in dorsal (notopodial) position, distally tapered to a point, edge smooth. Capillary chaetae, externally not pseudo-segmented. Capillary chaetae, internally not chambered or hollow. Spines absent, or present (anterior body only); slightly curved and more-or-less smooth (laterally), or sharply bent (=geniculate) or recurved (dorsally); present only in one or a few anterior chaetigers (distinct grapple-shaped hooks situated dorsally behind bases of branchiae); in ventral (neuropodial) position only. Hooks absent. Uncini present (from segment 6); with teeth in vertical series, teeth usually similar-sized (=pectinate), or with teeth arranged in transverse series above an enlarged main fang (=crested); in ventral (neuropodial) position throughout (shifted dorsally in first few chaetigers); arranged in one row.

Segmental organs are metanephridia; nephridial pores paired (ventrolateral) (segments 4–7; anterior pair for excretion, posterior 3 pairs for both excretion and gamete release), several pairs in anterior body. Tube present; membranous (fine sediment particles bound and lined with mucus; tube often extends above surface of sediment).

**Literature used for description**. Stiller J, Tilic E, Rousset V, Pleijel F and Rouse GW (2020). Spaghetti to a Tree: A Robust Phylogeny for Terebelliformia (Annelida) Based on Transcriptomes, Molecular and Morphological Data. Biology 9, 73; doi:10.3390/biology9040073; Alvestad T, Budaeva N (2021). Key to the species in Norwegian waters from the families Ampharetidae and Melinnidae. Online at https:[//www.artsdatabanken.no/Pages/302041/Key_to_the_species_in;](http://www.artsdatabanken.no/Pages/302041/Key_to_the_species_in%3B) Rouse, Tilic, Pleijel, new Black book; Rouse GW, Pleijel, F, Tilic E (2022) Annelida. Oxford University Press, Oxford, UK.

# Microchaetidae Beddard, 1895 emend. Plisko, 2012

**Description**. Body segment number: variable. Body shape elongate, more-or-less equal width over entire length. Dorsal intersegmental furrow present (?), pores on mid-dorsal line present (?). Pygidium simple lobe. Pygidial appendages absent. Discrete head present; lobe-like without appendages.

Prostomium bluntly conical, prolobic (prostomium demarcated from peristomium without a tongue). Peristomium a single ring.

Foregut a muscular dorsal pharynx. Gizzard present (one), in segment VII (supraoesophageal vessel absent). Calciferous glands present (oesophageal). Intestinal typhlosole present, variable. Heart bodies present, segments IX-XI.

Chaetae first appear on first segment after peristomium (=S2 for oligochaete workers), in lumbricine arrangement - paired upper and lower bundles of two chaetae per bundle in - closely spaced lateral and ventrolateral pairs (sometimes irregularly distanced). . Crotchet chaetae present, simple-pointed. Genital chaetae present.

Sexual reproduction hermaphroditic. Clitellum thick, multilayered, partially encircles body (or slightly encircling body), in region of male and female pores. Clitellum occurs from X to XXXIII, typically, although may occupy up to 44 segments. Tubercula pubertatis present. Gonadal segments bearing genital papillae present. Testes present, one pair in total or two pairs in total, one pair per segment; present in segments X and XI, or X. Sperm sac present. Ovaries present; present in segment XIII. Segmental organs are metanephridia; both nephridial pores and gonoducts located around clitellum, one pair nephridia in each segment (holonephridia) (V- or J-shaped nephridial bladders). Spermathecae present, simple, post-testicular or in testicular segments. Spermathecal pores present, paired, located within 1 or 2 segments of male pores or located well anterior to male pores; variable number pairs (usually 4-5); in segment X or IX-XIII. Female gonoduct present. Female pores one pair, in segment XIV. Male gonoducts present. Male pores single, median or one pair, in segment XV to XIX; in intersegmental furrows; sometimes beginning as early as segment XIII, opisthoporous (two or more segments following testicular segment). Penis absent. Prostate gland absent.

**Literature used for description**. Jamieson, BGM (2006) Non-leech Clitellata (with contributions by Marco Ferraguti). Pp. 235–392. In Reproductive Biology and Phylogeny of Annelida. Series Editor BGM Jamieson. Volume 4. Editors G Rouse, F Pleijel. Science Publishers, Enfield, New Hampshire; Plisko. JD (2012) Notes on the status of the family Microchaetidae. Zoology in the Middle East 58:suppl. 4: 47–58, DOI:10.1080/09397140.2012.10648984. Plisko JD, Nxele, TC (2015) An Annotated Key Separating Foreign Earthworm Species from the Indigenous South African taxa (Oligochaeta: Acanthodrilidae, Eudrilidae, Glossoscolecidae, Lumbricidae, Megascolecidae, Microchaetidae, Ocnerodrilidae and Tritogeniidae). African Invertebrates 56:663–708; Nxele, TC, Plisko, JD, Mwabvu, T, Zishiri, TO (2016) A new family Kazimierzidae for the genus *Kazimierzus*, earlier recorded to the composite Microchaetidae (Annelida, Oligochaeta). African Invertebrates 57(2): 111–117; Misirlioðlu, M, Reynolds, JW, Stojanoviæ, M, Trakiæ, T, Sekuliæ, J, James, SW, Csuzdi, C, Decaëns, T, Lapied, E, Phillips, HRP, Cameron, EK, Brown, GG (2023). Earthworms (Clitellata, Megadrili) of the world: an updated checklist of valid species and families, with notes on their distribution. Zootaxa 5255 (1): 417–438.

# Microphthalmidae Hartmann-Schröder, 1971, emended Salazar-Vallejo et al. 2019

**Description**. Body segment number: variable. Body shape elongate, more-or-less equal width over entire length. Dorsal body surface, appearance under compound microscope smooth. Pygidium membranous (foliose, lobate or convoluted; also called an anal plate). Pygidial appendages present, one pair of cirri. Discrete head present; complex in shape bearing appendages.

Prostomium rounded to oval, eyes on head absent or present, one pair (minute); head eyes simple pigmented cups. Prostomial antennae present; paired, lateral, or median and paired laterals; unarticulated. Palps absent (rarely), or present (resemble antennae); anteroventral; tapering (usually) sensory type; unarticulated. Nuchal organs present; paired low projections from posterolateral prostomium; indistinct dorsolateral ciliated patches. Peristomium not visible.

Gut more-or-less straight, lacking side branches. Foregut a muscular axial pharynx.

Protrusible proboscis present, smooth, distal ring of papillae present (?10).

First segment tentaculate (cirriferous segments well separated); second segment achaetous (cirriferous segments well separated). Tentacular cirri present; 4–6 pair(s); arise over two or more segments (segments 1–3 or 1–2); internal aciculae absent. First chaetiger with both notochaetae and neurochaetae; parapodia similar in length or slightly shorter than subsequent parapodia; parapodia more-or-less laterally directed and free from head; chaetae similar in orientation, length and thickness to other chaetae. Parapodia uniramous, or biramous; parapodial lobes prominent (though notopdial lobe may be reduced). Notopodial lobes absent (though dorsal cirri are present). Neuropodial lobes represented by at least one chaetal lobe. Dorsal cirri present; more-or-less cirriform (with undistinct basal cirrostyles). Ventral cirri present; cirriform or tapering.

Chaetae first appear on third segment after peristomium (*Ştruwella*) or fourth segment after peristomium, arranged in paired bundles (or rows) of many chaetae or as a single bundle (or row) of many chaetae (lower bundle - neurochaetae - only). Aciculae present; in both dorsal and ventral positions (=noto- and neuroaciculae). Neuroaciculae distally tapering. Capillary chaetae present, in dorsal (notopodial) position or in ventral (neuropodial) position (rarely), distally tapered to a point (various shapes from spine-like to sickle-shaped), edge hirsute-serrate. Capillary chaetae, externally not pseudo-segmented. Capillary chaetae, internally not chambered or hollow. Hooks absent. Compound chaetae absent (rarely), or present; in ventral (neuropodial) position; appendage distally curved (=falcate; ie., one distal tooth) (uni- or bidentate); appendage not canaliculated; appendage without hoods or guards; joint, appearance distinctly asymmetrical (=heterogomph); joint effected by ligament(s); shaft tapering slightly or evenly thick from emergence to joint; shaft, internally solid, without distinct core. Compound chaetae present in most or all chaetigers (neurochaetae only).

Segmental organs are protonephridia. Tube absent.

**Literature used for description**. Glasby CJ, Fauchald K (2003). POLiKEY. An information system for polychaete families and higher taxa. Version 2, 5 June 2003. Australian Biological Resources Study, DCCEEW, Canberra, [https://www.dcceew.gov.au/;](http://www.dcceew.gov.au/%3B) Salazar-Vallejo et al. (2019). Phylogeny of Microphthalminae Hartmann-Schröder, 1971, and revision of *Hesionella* Hartman, 1939, and *Struwela* Hartmann-Schröder, 1959 (Annelida, Errantia). PeerJ 7:e7723.; Rouse GW, Pleijel, F, Tilic E (2022) Annelida. Oxford University Press, Oxford, UK

# Moniligastridae Claus, 1880

**Description**. Body segment number: variable. Body shape elongate, more-or-less equal width over entire length. Dorsal intersegmental furrow present (?), pores on mid-dorsal line present (?). Pygidium simple lobe. Pygidial appendages absent.

Foregut a muscular dorsal pharynx. Gizzard present, multiple, posterior to the oesophagus. Heart bodies present.

Chaetae first appear on first segment after peristomium (=S2 for oligochaete workers - those of pre-clitellar segments may be minute), two per bundle, in lumbricine arrangement - paired upper and lower bundles closely spaced lateral and ventrolateral pairs. Crotchet chaetae absent or present (may be absent in anterior segments), simple-pointed. Genital chaetae absent.

Sexual reproduction hermaphroditic. Clitellum thin, multilayered, in region of male and female pores. Clitellum occurs from IX to XIV. Tubercula pubertatis present. Testes present, one pair in total or two pairs in total, one pair per segment; present in segments IX to XII. Sperm sac present. Ovaries present, one pair (in septal chambers); present in segments XI or XIII. Segmental organs are metanephridia; both nephridial pores and gonoducts located around clitellum. Spermathecae present (having unusually long ducts), with a basal diverticula, pre-testicular. Spermathecal pores present, located within 1 or 2 segments of male pores; 1–2 pairs; in segment VIII or VIII and IX. Female gonoduct present (yet to be recognised). Female pores one pair, in segment XII or XIV. Male gonoducts present. Male pores one pair or two pairs, in segment X or XI to XII, plesioporous (in segment following testicular segment). Prostate gland present (encapsulated), lobular, one pair or more than one pair. Prostate pores in segments IX−XII; prostate pore and male pore on segment XVII united, discharge through single pore (combined male and prostate pores).

**Literature used for description**. James SW (2000). An Illustrated Key to the Earthworms of The Samoan Archipelago (Oligochaeta: Glossoscolecidae, Moniligastridae). Technical Report No. 49, 11 pp; Jamieson, BGM (2006) Non-leech Clitellata (with contributions by Marco Ferraguti). Pp. 235–392. In Reproductive Biology and Phylogeny of Annelida. Series Editor BGM Jamieson. Volume 4. Editors G Rouse, F Pleijel. Science Publishers, Enfield, New Hampshire; Misirlioðlu, M, Reynolds, JW, Stojanoviæ, M, Trakiæ, T, Sekuliæ, J, James, SW, Csuzdi, C, Decaëns, T, Lapied, E, Phillips, HRP, Cameron, EK, Brown, GG (2023). Earthworms (Clitellata, Megadrili) of the world: an updated checklist of valid species and families, with notes on their distribution. Zootaxa 5255

(1): 417–438.

# Myzostomida von Graff, 1877

**Description**. Body segmentation absent (but segmentation is indicated by the 5 pairs of parapodia and repetition of internal organs). Body shape circular (usually, with marginal cirri on perimeter; rarely, some species are elongated to match for example pinnules of their host), dorsoventrally flattened. Body pigmentation present, diverse colours that often match the colour pattern of the host; epidermis more-or-less smooth, or thick and rugose, or papillate, or with radial or transverse dorsal ridges. Dorsal body surface, appearance under compound microscope smooth, or ciliated. Body margin cirrate, scalloped or irregular (usually 10 pairs of marginal cirri (alternating with parapodia)). Pygidium simple lobe (anus ventral). Pygidial appendages absent. Elongate proboscis present; retractable and tube-like. Discrete head absent.

Peristomium not visible.

Gut straight with side branches. Foregut a muscular axial pharynx. Protrusible proboscis present, distal ring of papillae present (4 to 30 papillae). Caeca of midgut (=posterior crop caeca) present (several pairs). Circulatory system absent.

Parapodia present. Parapodia uniramous (usually 5 pairs of appendages comprising small lobes with chaetae). Notopodial lobes absent. Neuropodial lobes represented by at least one chaetal lobe. Lateral organs present (sucker or pit-like organ on ventrolateral body).

Arranged as a single bundle (or row) of many chaetae (lower bundle - neurochaetae - only). Aciculae present; in ventral position (=neuroaciculae). Neuroaciculae distally bent at right angles. Capillary chaetae absent. Hooks present; without distal hood, beard or ligament; occur in ventral (neuropodial) position; occur over entire body.

Sexual reproduction hermaphroditic. Testes present, one pair in total. Ovaries present, one pair or two pairs. Segmental organs are protonephridia (5 pairs; possible also a pair of posterior metanephridia); present in most segments of the body. Female gonoduct present. Female pores single, median, in segment near anus. Male gonoducts present. Male pores one pair, in segment midbody (parapodia 3). Penis present (paired in ectoparasites, absent in endoparastites).

Tube absent.

**Literature used for description**. Grygier, MJ (2000), Chapter 2. Class Myzostomida, pp. 297–329 In CJ Glasby et al. (Eds) Fauna of Australia Vol. 4A. Polychaetes & Allies. The Southern Synthesis. ABRS, Canberra; Eeckhaut I, Lanterbecq D (2021) 7.10 Myzostomida In: Purschke G, Böggemann M, Westheide W (Eds), Handbook of Zoology, Annelida, Volume 3: Pleistoannelida, Sedentaria III and Errantia I. De Gruyter, Berlin, 228–265; Rouse GW, Pleijel, F, Tilic E (2022) Annelida. Oxford University Press, Oxford, UK.

# Naididae Ehrenberg, 1831, sensu lato

**Description**. Body segment number: variable (many naidids can reproduce asexually by paratomy, resulting in reproductive individuals bearing a posterior chain of zooids). Secondary annulation absent or present. Body shape elongate, more-or-less equal width over entire length, more-or-less cylindrical (rarely quadrangular). Body pigmentation absent (often pink or red due to colour of blood); epidermis more-or-less smooth, or tuberculate, with bumps of various sizes and arrangements (rarely, e.g., the tubificine *Baikalodrilus* species which have 4 tubercles per segment near the setal bundles). Pygidium simple lobe. Pygidial appendages absent or present (rarely), single medial cirrus and one pair of cirri (when present, two or three ciliated caudal appendages).

Prostomium bluntly conical (minute), with an anterior tentacle-like projection ( ='proboscis'; rarely), zygolobic (prostomium not demarcated), eyes on head absent or present (rarely), one pair; head eyes simple pigmented cups.

Gut absent (in *Olavius* and *Inanidrilus*; rely on bacterial symbionts for food) or present, more-or-less straight, lacking side branches or straight with side branches. Foregut a muscular dorsal pharynx. Heart bodies absent or present.

Branchiae absent or present (eg., *Branchiura sowerbyi, Dero, Branchiodrilus*), arise from lateral body, occur on mid-body segments (*Branchiodrilus*) or occur near posterior end or occur adjacent to terminal segment or pygidium (3 gill appendages or an anal gill chamber); lateral branchiae digitiform.

Chaetae two or more per bundle (rarely a single chaeta up to 10 chaetae per bundle anteriorly); dorsal bundle first appear on first segment after peristomium (rarely), or 4-6^th^ segment after peristomium (rarely, dorsal bundle absent, eg., *Chaetogaster*), ventral bundle appear on first segment after peristomium; , in lumbricine arrangement in closely spaced lateral and ventrolateral pairs or arranged in widely spaced lateral and ventrolateral pairs. Capillary (=hair) chaetae absent or present (dorsally), in dorsal (notopodial) position, distally tapered to a point, edge smooth or hirsute-serrate (rarely). Crotchet chaetae present (usually with nodulus), single-pointed, bifid or distally pectinate (rarely pectinate or palmate). Needle chaetae absent or present (in dorsal bundle), single-pointed or bifid or pectinate. Genital chaetae absent, or present (penial and/or spermathecal chaetae present or lacking).

Reproduction asexual, or sexual. Asexual reproduction by paratomy, or budding, or fragmentation.

Sexual reproduction hermaphroditic. Clitellum thin, single layered, in region of male pores (male pore may be on first clitellar segment), and female pores. Clitellum occurs from IV-XVI. Testes present, one pair in total; present in segments IV-XXI. Sperm sac present (paired or unpaired). Ovaries present, one pair; present in segments V-XXII. Egg sacs present (paired or unpaired; oviducts reduced to funnels only). Segmental organs are protonephridia (rarely), or metanephridia; nephridial pores located anteriorly, gonoducts located around clitellum or both nephridial pores and gonoducts located around clitellum or nephridial pores located posterior to gonoducts. Spermathecae present, post-testicular or in testicular segments. Spermathecal pores present, paired (mostly, rarely single or absent), located within 1 or 2 segments of male pores (usually in the testicular segment preceding or following male pore segment); in segment V-XXIII. Female gonoduct present. Female pores one pair, in segment VII-XXIII. Male gonoducts present, with atrium present. Male pores one pair, in segment V-XXII, plesioporous (in segment following testicular segment). Penis absent or present. Prostate gland absent (diffuse prostate) or present.

**Literature used for description**. Harman WJ (1969) Revision of the Family Opistocystidae (Oligochaeta). Transactions of the American Microscopical Society 88: 472–478; Brinkhurst RO and Jamieson, BGM (1971) Aquatic Oligochaeta of the World with contributions by DG Cook,

DV Anderson, J van der Land, University of Toronto Press, Toronto, Canada, 860 pp; Harman, WJ, Loden MS (1978) A Reevaluation of the Opistocystidae Oligochaeta with descriptions of two new genera. Proceedings of the Biological Society of Washington 91: 453–462; Jamieson, BGM (2006) Non-leech Clitellata (with contributions by Marco Ferraguti). Pp. 235–392. In Reproductive Biology and Phylogeny of Annelida. Series Editor BGM Jamieson. Volume 4. Editors G Rouse, F Pleijel. Science Publishers, Enfield, New Hampshire; Martin, P, Martinez-Ansemil, E., Pinder, A, Timm, T, Wetzel, MJ (2008) Global diversity of oligochaetous clitellates (‘‘Oligochaeta’’; Clitellata) in freshwater. Hydrobiologia (2008) 595:117–127; Timm, T (2009) A guide to the freshwater Oligochaeta and Polychaeta of Northern and Central Europe. Lauterbornia 66: 1–235; Gustavsson, L. M. (2017). Molecular data reveal a tropical freshwater origin of Naidinae (Annelida, Clitellata, Naididae). Molecular Phylogenetics and Evolution 115: 115–127; Thorp JH, Lovell LL, Timm T, Martin P, Gelder SR, Govedich FR, Moser WE, Nakano T, Bielecki A, Bain BA, Utevsky S, Gil J, Glasby CJ, Martin D (2019) Phylum Annelida. In: Rogers DC, Thorp JH (Eds) Keys to Palaearctic Fauna: Thorp and Covich’s Freshwater invertebrates - Volume IV. Academic Press, Elsevier, pp. 357–518; Schmelz,RM, Erséus, C, Martin P, van Haaren T, Timm T (2021) A proposed order-level classification in Oligochaeta (Annelida, Clitellata). Zootaxa 5040 (4): 589–597.

# Naididae, Naidinae Ehrenberg, 1831

**Description**. Body segment number: variable (many naidids can reproduce asexually by paratomy, resulting in reproductive individuals bearing a posterior chain of zooids). Secondary annulation absent or present. Body shape elongate, more-or-less equal width over entire length (small worms). Pygidium simple lobe. Pygidial appendages absent or present (*Aulophorus* species have two ciliated caudal appendages).

Prostomium bluntly conical (minute), with an anterior tentacle-like projection (='proboscis'; rarely, in some genera), zygolobic (prostomium not demarcated), eyes on head absent (rarely) or present, one pair; head eyes simple pigmented cups.

Gut more-or-less straight, lacking side branches. Foregut a muscular dorsal pharynx. Heart bodies present.

Branchiae absent or present (rarely, e.g., *Dero, Branchiodrilus*), arise from lateral body on mid-body segments (*Branchiodrilus*) or occur near posterior end or occur adjacent to terminal segment or pygidium (an anal gill chamber in *Aulophorus*, *Dero*); lateral branchiae digitiform.

Chaetae two or more per bundle (rarely a single chaeta; *Paranais* can have 7 chaetae per bundle); dorsal bundle first appear on first segment after peristomium (rarely), or 4-6^th^ segment after peristomium (rarely, dorsal bundle absent, eg., *Chaetogaster*), ventral bundle appear on first segment after peristomium; in lumbricine arrangement - paired upper and lower bundles of one or a few chaetae each (usually several chaetae) - closely spaced lateral and ventrolateral pairs. Capillary (=hair) chaetae absent or present (dorsally), in dorsal (notopodial) position, distally tapered to a point, edge smooth. Crotchet chaetae present (usually with nodulus), bifid (usually). Needle chaetae present, single-pointed or bifid or pectinate, or absent. Genital chaetae absent, or present.

Reproduction asexual, or sexual. Asexual reproduction by budding, or fragmentation.

Sexual reproduction hermaphroditic. Clitellum thin, single layered, in region of male pores (male pore on first clitellar segment), and female pores. Clitellum occurs from IV-VIII. Testes present, one pair in total; present in segments IV, V. Sperm sac present (usually unpaired). Ovaries present, one pair; present in segments V, VI. Egg sacs present (usually unpaired; oviducts reduced to funnels only). Segmental organs are protonephridia (rarely), or metanephridia; nephridial pores located posterior to gonoducts. Spermathecae present, pre-testicular or in testicular segments. Spermathecal pores present, paired (mostly), located within 1 or 2 segments of male pores (usually in the testicular segment preceding male pores); in segment IV or V. Female gonoduct present. Female pores one pair, in segment VII. Male gonoducts present, with atrium present. Male pores one pair, in segment V or VI, plesioporous (in segment following testicular segment). Penis absent. Prostate gland present.

**Literature used for description**. Brinkhurst RO and Jamieson, BGM (1971) Aquatic Oligochaeta of the World with contributions by DG Cook, DV Anderson, J van der Land, University of Toronto Press, Toronto, Canada, 860 pp; Jamieson, BGM (2006) Non-leech Clitellata (with contributions by Marco Ferraguti). Pp. 235–392. In Reproductive Biology and Phylogeny of Annelida. Series Editor BGM Jamieson. Volume 4. Editors G Rouse, F Pleijel. Science Publishers, Enfield, New Hampshire; Martin, P, Martinez-Ansemil, E., Pinder, A, Timm, T, Wetzel, MJ (2008) Global diversity of oligochaetous clitellates (‘‘Oligochaeta’’; Clitellata) in freshwater. Hydrobiologia (2008) 595:117–127; Timm, T (2009) A guide to the freshwater Oligochaeta and Polychaeta of Northern and Central Europe. Lauterbornia 66: 1–235. Thorp JH, Lovell LL, Timm T, Martin P, Gelder SR, Govedich FR, Moser WE, Nakano T, Bielecki A, Bain BA, Utevsky S, Gil J, Glasby CJ, Martin D (2019) Phylum Annelida. In: Rogers DC, Thorp JH (Eds) Keys to Palaearctic Fauna: Thorp and Covich’s Freshwater invertebrates - Volume IV. Academic Press, Elsevier, pp. 357–518.

# Naididae, Opistocystinae Černosvitov, 1936

**Description**. Body segment number: variable (opistocystids can reproduce asexually by paratomy, resulting in additional budded reproductive segments). Secondary annulation absent or present. Body shape elongate, more-or-less equal width over entire length. Pygidium simple lobe. Pygidial appendages present (three ciliated caudal appendages), single medial one and a pair of longer ventrolateral ones.

Prostomium bluntly conical (minute), anteriorly with an anterior tentacle-like projection (='proboscis'), eyes on head absent.

Foregut a muscular dorsal pharynx.

Branchiae present, occur adjacent to terminal segment or pygidium (3 gill appendages).

Chaetae first appear on first segment after peristomium (=S2 for oligochaete workers), in lumbricine arrangement - paired upper and lower bundles of one or a few chaetae each. Capillary (=hair) chaetae present, in dorsal (notopodial) position, distally tapered to a point, edge smooth or hirsute-serrate. Crotchet chaetae present, bifid. Needle chaetae absent or present (in dorsal bundle), single-pointed.

Reproduction asexual, or sexual. Asexual reproduction by budding.

Sexual reproduction hermaphroditic. Clitellum thin, single layered, in region of male and female pores. Clitellum occurs from XI to XVI. Testes present, one pair in total; present in segments XI to XXI. Ovaries present; present in segments XII to XXII. Oviducts reduced to funnels only. Segmental organs are metanephridia. Spermathecae present, post-testicular. Spermathecal pores present, paired (mostly), located within 1 or 2 segments of male pores (in segment immediately posterior to the one bearing the male and female pores); in segment XII (*Crustipellis*) or XXIII/XXIV (*Opistocysta* and *Trieminentia)*. Female gonoduct present. Female pores one pair, in segment XII or XXII - XXIII. Male gonoducts present, with atrium present (oval). Male pores one pair, in segment XII or XXII, plesioporous (in segment following testicular segment). Penis present (eversible). Prostate gland absent (diffuse prostate covering atria).

**Literature used for description**. Harman WJ (1969) Revision of the Family Opistocystidae (Oligochaeta). Transactions of the American Microscopical Society 88: 472–478; Brinkhurst RO and Jamieson, BGM (1971) Aquatic Oligochaeta of the World with contributions by DG Cook,

DV Anderson, J van der Land, University of Toronto Press, Toronto, Canada, 860 pp; Harman, WJ, Loden MS (1978) A Reevaluation of the Opistocystidae Oligochaeta with descriptions of two new genera. Proceedings of the Biological Society of Washington 91: 453–462; Jamieson, BGM (2006) Non-leech Clitellata (with contributions by Marco Ferraguti). Pp. 235–392. In Reproductive Biology and Phylogeny of Annelida. Series Editor BGM Jamieson. Volume 4. Editors G Rouse, F Pleijel. Science Publishers, Enfield, New Hampshire; Erséus, C, Envall, I, Marchese, M, Gustavsson, L (2010). The systematic position of Opistocystidae (Annelida, Clitellata) revealed by DNA data. Molecular Phylogenetics and Evolution 54: 309–313; Erséus, C.; Envall, I.; De Wit, P.; Gustavsson, L. M. (2017). Molecular data reveal a tropical freshwater origin of Naidinae (Annelida, Clitellata, Naididae). Molecular Phylogenetics and Evolution 115: 115–127.

# Naididae, Pristininae Lastočkin, 1921

**Description**. Body segment number: variable (many pristinids can reproduce asexually by paratomy, resulting in reproductive individuals bearing a posterior chain of zooids). Secondary annulation absent or present. Body shape elongate, more-or-less equal width over entire length (small worms). Pygidium simple lobe. Pygidial appendages absent.

Prostomium bluntly conical (minute), with an anterior tentacle-like projection (='proboscis'; rarely), zygolobic (prostomium not demarcated).

Foregut a muscular dorsal pharynx.

Chaetae first appear on first segment after peristomium (dorsal and ventral bundles; =S2 for oligochaete workers), in lumbricine arrangement - paired upper and lower bundles of one or a few chaetae each - widely spaced lateral and ventrolateral pairs. Capillary (=hair) chaetae present, in dorsal (notopodial) position, distally tapered to a point, edge smooth. Crotchet chaetae present (usually with nodulus), bifid (usually). Needle chaetae present, single-pointed or bifid. Genital chaetae absent, or present.

Reproduction asexual, or sexual. Asexual reproduction by paratomy.

Sexual reproduction hermaphroditic. Clitellum thin, single layered, in region of male and female pores. Clitellum occurs from VI to VIII. Testes present, one pair in total; present in segment VI. Ovaries present, one pair; present in segment VII. Oviducts reduced to funnels only. Segmental organs are metanephridia; both nephridial pores and gonoducts located around clitellum. Spermathecae present, in testicular segments. Spermathecal pores present, paired (mostly), located within 1 or 2 segments of male pores (usually in the testicular segment VI or VII); in segment VI-VII (VII according to Timm 2009). Female gonoduct present. Female pores one pair, in segment VIII. Male gonoducts present, with atrium present. Male pores one pair, in segment VII, plesioporous (in segment following testicular segment). Penis present. Prostate gland absent.

**Literature used for description**. Timm, T (2009) A guide to the freshwater Oligochaeta and Polychaeta of Northern and Central Europe. Lauterbornia 66: 1–235; Schmelz,RM, Erséus, C, Martin P, van Haaren T, Timm T (2021) A proposed order-level classification in Oligochaeta (Annelida, Clitellata). Zootaxa 5040 (4): 589–597.

# Naididae, Tubificinae d'Udekem, 1855

**Description**. Body segment number: variable. Secondary annulation absent or present. Body shape elongate, more-or-less equal width over entire length (long tapering tail region), more-or-less cylindrical (rarely quadrangular). Body pigmentation absent (often pink or red due to blood); epidermis more-or-less smooth, or tuberculate, with bumps of various sizes and arrangements (rarely, eg., *Baikalodrilus* species which have 4 tubercles per segment near the chaetal bundles). Pygidium simple lobe. Pygidial appendages absent.

Prostomium bluntly conical, prolobic (prostomium not demarcated). Eyes absent.

Gut more-or-less straight, lacking side branches or straight with side branches. Foregut a muscular dorsal pharynx. Heart bodies absent or present.

Branchiae usually absent (only present in a single species), arise from dorsal body, occur near posterior end (digitiform).

Chaetae two per bundle or more (up to 10 chaetae per bundle in anterior bundles of *Aulodrilus*); dorsal bundle first appear on first segment after peristomium (mostly), ventral bundle first appear on first segment after peristomium; in lumbricine arrangement - paired upper and lower bundles of one or a few chaetae each (usually several chaetae) - widely spaced lateral and ventrolateral pairs. Capillary (=hair) chaetae absent or present (*Limnodrilus* species lack hair chaetae), in dorsal (notopodial) position, distally tapered to a point, edge smooth or hirsute-serrate (rarely, eg. in *Baikalodrilus*). Crotchet chaetae present (usually with nodulus), bifid or distally pectinate (rarely pectinate or palmate). Genital chaetae absent, or present (penial and/or spermathecal chaetae present or lacking).

Reproduction asexual, or sexual. Asexual reproduction by fragmentation.

Sexual reproduction hermaphroditic. Clitellum thin, single layered, in region of male pores (male pores on first clitellar segment), and female pores. Clitellum occurs from X-XI, usually, range IX-XII. Testes present, one pair in total; present in segment X, usually. Sperm sac present (often). Ovaries present, one pair; present in segment XI, usually. Egg sacs present; oviducts reduced to funnels only. Segmental organs are metanephridia; , nephridial pores located anteriorly, gonoducts located around clitellum (nephridia often poorly developed on one side of the body). Spermathecae present, in testicular segment. Spermathecal pores present, paired (usually, rarely single or absent), located within 1 or 2 segments of male pores (usually in the testicular segment X preceding or following male pore segment). Female gonoduct present. Female pores one pair, in segment XII. Male gonoducts present, with atrium present. Male pores one pair, in segment XI, plesioporous (in segment following testicular segment). Prostate gland present.

**Literature used for description**. Brinkhurst RO and Jamieson, BGM (1971) Aquatic Oligochaeta of the World with contributions by DG Cook, DV Anderson, J van der Land, University of Toronto Press, Toronto, Canada, 860 pp; Jamieson, BGM (2006) Non-leech Clitellata (with contributions by Marco Ferraguti). Pp. 235–392. In Reproductive Biology and Phylogeny of Annelida. Series Editor BGM Jamieson. Volume 4. Editors G Rouse, F Pleijel. Science Publishers, Enfield, New Hampshire; Martin, P, Martinez-Ansemil, E., Pinder, A, Timm, T, Wetzel, MJ (2008) Global diversity of oligochaetous clitellates (‘‘Oligochaeta’’; Clitellata) in freshwater. Hydrobiologia (2008) 595:117–127; Timm, T (2009) A guide to the freshwater Oligochaeta and Polychaeta of Northern and Central Europe. Lauterbornia 66: 1–235; Thorp JH, Lovell LL, Timm T, Martin P, Gelder SR, Govedich FR, Moser WE, Nakano T, Bielecki A, Bain BA, Utevsky S, Gil J, Glasby CJ, Martin D (2019) Phylum Annelida. In: Rogers DC, Thorp JH (Eds) Keys to Palaearctic Fauna: Thorp and Covich’s Freshwater invertebrates - Volume IV. Academic Press, Elsevier, pp. 357–518.

# Narapidae Righi & Varela, 1983

**Description**. Secondary annulation absent or present. Body shape elongate, more-or-less equal width over entire length. Pygidium simple lobe.

Foregut a muscular dorsal pharynx.

Chaetae absent (presence of chaetae indicated as uncertain in type description; considered absent by some authors).

Sexual reproduction hermaphroditic. Clitellum thin, single layered, in region of male and female pores. Clitellum occurs from V to VII. Seminal groove absent. Tubercula pubertatis absent. Testes present, one pair in total; present in segment V. Ovaries present, unpaired; present in segments VII. Segmental organs are metanephridia. Spermathecae present, post-testicular. Spermathecal pores present, paired, located within 1 or 2 segments of male pores; 1 pair; in segment VII. Female gonoduct absent. Male gonoducts present, with atrium present. Male pores one pair, in segment VI, plesioporous (in segment following testicular segment). Penis present. Prostate gland absent.

**Literature used for description**. Righi, G, Varela, ME (1983). *Narapa bonettoi*, gen. nov. sp. nov.(Oligochaeta, Narapidae, fam. nov.) de agua doce da Argentina. Natura Neotropicalis. 1(14): 7–15. Jamieson, BGM (2006) Non-leech Clitellata (with contributions by Marco Ferraguti). Pp. 235–392. In Reproductive Biology and Phylogeny of Annelida. Series Editor BGM Jamieson. Volume 4. Editors G Rouse, F Pleijel. Science Publishers, Enfield, New Hampshire.

# Nephtyidae Grube, 1850

**Description**. Body segment number: variable. Body shape elongate, more-or-less equal width over entire length. Body pigmentation absent or present (occasionally with some dark brown pigment on dorsum); epidermis more-or-less smooth. Pygidium simple lobe. Pygidial appendages present, single medial cirrus. Discrete head present; complex in shape bearing appendages.

Prostomium pentagonal to quadrangular, eyes on head absent or present, one pair or two pairs; head eyes simple pigmented cups. Postcephalic eyes present; subepidermal. Prostomial antennae present; paired, lateral (rarely absent); unarticulated. Palps present (unarticulated; resemble antennae, although rarely the palps are bifid); anteroventral; tapering (usually) sensory type; unarticulated. Nuchal organs present; paired low projections from posterolateral prostomium; indistinct dorsolateral ciliated patches. Peristomium not visible.

Foregut a muscular axial pharynx. Pharynx jaws present, one pair of lateral jaws (rarely with an additional pair of toothed plates). Paired jaws fang-like. Protrusible proboscis present, with papillae in subterminal position, in distinct rows, distal ring of papillae present (rarely absent).

First segment chaetous (dorsal and ventral cirri may be slightly enlarged, resembling tentacles); second segment chaetous. Tentacular cirri present. First chaetiger with both notochaetae and neurochaetae; parapodia similar in length or slightly shorter than subsequent parapodia; parapodia anteriorly directed and wrapping around head; chaetae similar in orientation, length and thickness to other chaetae. Parapodia biramous; parapodial lobes prominent. Notopodial lobes represented by at least one chaetal lobe. Neuropodial lobes represented by at least one chaetal lobe. Interramal fleshy process present. Interramal fleshy process cirrus-like (recurved inward or outward; may be referred to as branchiae or gills). Dorsal cirri present; more-or-less cirriform. Ventral cirri present; cirriform or tapering. Branchiae present, arise from lateral body (attached ventrally on notopodium and extending into interramal space), occur on mid-body segments; lateral branchiae recurved cirrus (rarely papilliform).

Chaetae first appear on first segment after peristomium, arranged in paired bundles (or rows) of many chaetae. Aciculae present; in both dorsal and ventral positions (=noto- and neuroaciculae). Capillary chaetae present, in dorsal (notopodial) position or in ventral (neuropodial) position, distally tapered to a point or sharply bent, edge smooth or hirsute-serrate. Capillary chaetae, externally not pseudo-segmented. Capillary chaetae, internally not chambered or hollow, or distinctly chambered. Forked chaetae absent, or present. lyrate, tines more-or-less equal in length. Hooks absent.

Segmental organs are protonephridia.

Tube absent.

**Literature used for description**. Glasby CJ, Fauchald K (2003). POLiKEY. An information system for polychaete families and higher taxa. Version 2, 5 June 2003. Australian Biological Resources Study, DCCEEW, Canberra, https:[//www.dcceew.gov.au/;](http://www.dcceew.gov.au/%3B) Rava A, Rizzo AE, Lana P (2022) 7.13.8 Nephtyidae Grube, 1850 In: Purschke G, Böggemann, M, Westheide W (Eds) Handbook of Zoology. Annelida. Vol. 4: Pleistoannelida, Errantia II. De Gruyter, Berlin, 380–393; Rouse GW, Pleijel, F, Tilic E (2022) Annelida. Oxford University Press, Oxford, UK.

# Nereididae Blainville, 1818

**Description**. Body segment number: variable. Body shape elongate, more-or-less equal width over entire length. Body pigmentation absent or present, olive-green, brown and orange are common base colours with or without contrasting bright speckles or patches; some species have dark bands stripes on one or more anterior segments; epidermis more-or-less smooth. Pygidium simple lobe. Pygidial appendages present, one pair of cirri. Discrete head present; complex in shape bearing appendages.

Prostomium bluntly conical (sometimes called an inverted t-shape), eyes on head present (rarely absent), two pairs; head eyes compound, with lenses. Prostomial antennae present (rarely absent); paired, lateral; unarticulated. Palps present; anteroventral; tapering (usually) sensory type; biarticulated (robust basal part). Nuchal organs present; paired low projections from posterolateral prostomium; indistinct dorsolateral ciliated patches. Peristomium a single ring, enlarged and fused, referred to in recent literature as a tentacular belt.

Gut more-or-less straight, lacking side branches (single pair of caeca posterior to pharynx). Foregut a muscular axial pharynx. Pharynx jaws present (very rarely absent), one pair of lateral jaws. Paired jaws fang-like (often serrated). Pharynx teeth absent (rarely) or present (=paragnaths), arranged in distinct areas. Protrusible proboscis present, smooth or with papillae in subterminal position, in distinct areas, distal ring of papillae absent.

First segment tentaculate; second segment chaetous (assuming the tentacular cirri are all derived from segment 1, which needs confirmation). Tentacular cirri present; 3–4 pair(s); arise on a single segment (which is fused to peristomium); internal aciculae absent. First chaetiger with neurochaetae only, or with both notochaetae and neurochaetae; parapodia similar in length or slightly shorter than subsequent parapodia; parapodia more-or-less laterally directed and free from head; chaetae similar in orientation, length and thickness to other chaetae. Parapodia uniramous (rarely), or biramous (up to five lobes per parapodium); parapodial lobes prominent. Notopodial lobes represented by at least one chaetal lobe, or absent. Neuropodial lobes represented by at least one chaetal lobe. Dorsal cirri present; more-or-less cirriform, or flattened and foliaceous (rarely; see 'branchiae' for the range of dorsal cirri modifications). Ventral cirri present; cirriform or tapering (see 'branchiae' for the range of ventral cirri modifications). Branchiae absent or present (rarely), arise from lateral body (vascularised parapodial structures), occur first few segments or occur on mid-body segments; lateral branchiae digitiform (modified dorsal, and sometimes also, ventral cirri, which can be expanded and lobe-like, tree-like or pectinate). Chaetae first appear on second segment after peristomium (assuming the tentacular cirri are all derived from segment 1, which needs confirmation), arranged in paired bundles (or rows) of many chaetae or as a single bundle (or row) of many chaetae (lower bundle - neurochaetae - only). Aciculae present; in both dorsal and ventral positions (=noto- and neuroaciculae). Neuroaciculae distally tapering (usually with a squared-off tip; rarely, sharply curved distally). Capillary chaetae absent. Spines absent, or present (rarely in *Simplisetia. Hediste* and *Platynereis*); slightly curved and more-or-less smooth, or sharply bent (=geniculate) or recurved; present in most or all chaetigers; in both dorsal and ventral positions. Genital chaetae absent, or present (rarely, as either hooked-shaped acicula or non-acicular chaetae in extreme anterior or posterior body); hooks absent. Compound chaetae present; in ventral (neuropodial) position, or in dorsal (notopodial) position; appendage distally tapering to slender tips (=spinigerous), or distally curved (=falcate; ie., one distal tooth); appendage not canaliculated; appendage without hoods or guards; joint, appearance more-or-less symmetrical (=homogomph; includes also hemigomph and sesquigomph), or distinctly asymmetrical (=heterogomph); joint effected by ligament(s) (double ligaments); shaft tapering slightly or evenly thick from emergence to joint; shaft, internally chambered, with camerated core. Compound chaetae present in most or all chaetigers.

Sexual reproduction gonochoric (many species undergo epitokous metamorphosis prior to breeding; metamorphosed forms may not be identifiable with this key). Segmental organs are metanephridia.

Tube absent, or present (eg., *Platynereis*); membranous.

**Literature used for description**. Glasby CJ, Fauchald K (2003). POLiKEY. An information system for polychaete families and higher taxa. Version 2, 5 June 2003. Australian Biological Resources Study, DCCEEW, Canberra, https:[//www.dcceew.gov.au/;](http://www.dcceew.gov.au/%3B) Bakken T, Glasby CJ, Santos, CSG, Wilson RS (2022) 7.13.3.3 Nereididae Blainville, 1818, In: Purschke G, Böggemann M, Westheide W (Eds), Handbook of Zoology, Annelida, Volume 3: Pleistoannelida, Errantia II. De Gruyter, Berlin, 259–307; Rouse GW, Pleijel, F, Tilic E (2022) Annelida. Oxford University Press, Oxford, UK.

# Nerillidae Levinsen, 1883

**Description**. Body segment number: fixed, less than 14 segments (7–9 body segments). Secondary annulation present (up to 10 annuli present throughout body). Body shape sausage or grub-shaped. Body translucent, gut visible; epidermis more-or-less smooth. Dorsal body surface, appearance under compound microscope ciliated. Pygidium simple lobe. Pygidial appendages present, one pair of cirri. Discrete head present; complex in shape bearing appendages.

Prostomium rounded to oval, eyes on head absent or present, one pair or two pairs; head eyes simple pigmented cups (red or, less commonly, blue). Prostomial antennae present; paired, lateral, or median and paired laterals; unarticulated, or articulated. Palps present; anteroventral; tapering (usually) sensory type (highly variable in shape); unarticulated. Nuchal organs present; paired low projections from posterolateral prostomium; indistinct dorsolateral ciliated patches. Peristomium not visible.

Gut more-or-less straight, lacking side branches. Foregut a muscular ventral pharynx. Pharynx jaws absent (although fine stylets may be present, observable through the body wall). Pharynx dorsolateral ciliated folds present. First segment chaetous and tentaculate (rarely); second segment chaetous. Tentacular cirri 1 pair(s); arise on a single segment; internal aciculae absent. First chaetiger with neurochaetae only, or with both notochaetae and neurochaetae; parapodia similar in length or slightly shorter than subsequent parapodia; parapodia more-or-less laterally directed and free from head; chaetae similar in orientation, length and thickness to other chaetae. Parapodia biramous (presumably, although uncertain); parapodial lobes absent or very low. Notopodial lobes represented by at least one chaetal lobe. Neuropodial lobes represented by at least one chaetal lobe. Interramal fleshy process present. Interramal fleshy process cirrus-like (the only distinct parapodial lobe). Dorsal cirri absent (cirri present arise from between noto- and neuropodia). Branchiae absent.

Chaetae first appear on first segment after peristomium, arranged in paired bundles (or rows) of many chaetae. Aciculae absent. Capillary chaetae present, distally tapered to a point, edge smooth. Capillary chaetae, externally not pseudo-segmented. Capillary chaetae, internally not chambered or hollow. Hooks absent. Compound chaetae present; appendage distally curved (=falcate; ie., one distal tooth) (long-bladed falcigers); appendage not canaliculated; appendage without hoods or guards; joint, appearance distinctly asymmetrical (=heterogomph); joint effected by ligament(s); shaft tapering slightly or evenly thick from emergence to joint; shaft, internally solid, without distinct core. Compound chaetae present in most or all chaetigers.

Sexual reproduction hermaphroditic, or gonochoric. Testes absent (spermatocytes develop in coelom). Ovaries absent (oocytes develop in coelom). Segmental organs are protonephridia, or metanephridia (rarely). Female gonoduct present. Female pores two pairs, in segment 7, usually. Male gonoducts present (2–3 pairs). Male pores single (median), or one pair, in segment 5 to 7.

Tube absent.

**Literature used for description**. Glasby CJ, Fauchald K (2003). POLiKEY. An information system for polychaete families and higher taxa. Version 2, 5 June 2003. Australian Biological Resources Study, DCCEEW, Canberra, https:[//www.dcceew.gov.au/;](http://www.dcceew.gov.au/%3B) Worsaae K (2020) Errantia incertae sedis: Nerillidae Levinsen, 1883 In: Wesheide W, Purschke G, Böggemann M eds Handbook of Zoology, Annelida, Volume 3 Pleistoannelida, Sedentaria III and Errantia I, Berlin, Boston: De Gruyter, 2021. https://doi.org/10.1515/9783110291704; Worsaae, K, Kerbl, A., Di Domenico, M., Gonzalez, B.C., Bekkouche, N. & Martínez, A. (2021) Interstitial Annelida. Diversity 2021, 13, 77. https://doi.org/10.3390/d13020077; Thorp JH et al. (2019) Phylum Annelida, In Keys to Palaearctic Fauna: Thorp and Covich's Freshwater Invertebrates, Volume IV. Academic Press, Elsevier; Worsaae K (2021) 7.9 Errantia incertae sedis: Nerillidae Levinsen, 1883 In: Purschke G, Böggemann M, Westheide W (Eds), Handbook of Zoology, Annelida, Volume 3: Pleistoannelida, Sedentaria III and Errantia I. De Gruyter, Berlin, 215–228; Rouse GW, Pleijel, F, Tilic E (2022) Annelida. Oxford University Press, Oxford, UK.

# Ocnerodrilidae Beddard, 1891

**Description**. Body segment number: variable. Body shape elongate, more-or-less equal width over entire length. Dorsal intersegmental furrow present (?), pores on mid-dorsal line present (?). Pygidium simple lobe. Pygidial appendages absent.

Prostomium bluntly conical.

Gut more-or-less straight, lacking side branches or straight with side branches. Foregut a muscular dorsal pharynx. Calciferous glands absent or present, in segment IX or X (or segments IX and X). Caeca of foregut present (paired). Heart bodies present, extend to segment XI.

Chaetae first appear on first segment after peristomium (=S2 for oligochaete workers), in lumbricine arrangement - paired upper and lower bundles with 2 chaetae per bundle - closely spaced lateral and ventrolateral pairs or arranged in widely spaced lateral and ventrolateral pairs. Crotchet chaetae present, simple-pointed. Genital chaetae absent, or present.

Sexual reproduction hermaphroditic. Clitellum thick, multilayered, fully encircles body, in region and female pores. Clitellum occurs from XIII to XVIII or XIV to XIX. Seminal groove absent, or present. Tubercula pubertatis present, paired ridges on ventrolateral margins of clitellum. Testes present, one pair in total or two pairs in total, one pair per segment; present in segments X or XI. Ovaries present, one pair; present in segments

XIII. Segmental organs are metanephridia; both nephridial pores and gonoducts located around clitellum, one pair nephridia in each segment (holonephridia). Spermathecae absent or present, simple or with a basal diverticula (rarely), pre-testicular. Spermathecal pores absent or present, located well anterior to male pores; 1–4 pairs; in segment VII-X. Female gonoduct present (often conspicuous). Female pores one pair or two pairs, in segment XIV. Male gonoducts present (inconspicuous or on small protuberances). Male pores one pair, in segment XVII or XVIII, opisthoporous (two or more segments following testicular segment). Prostate gland present, tubular, one pair. Prostate pores in segments XVII to XIX, ventral; prostate pore and male pore on segment XVII united, discharge through single pore.

**Literature used for description**. Jamieson, BGM (2006) Non-leech Clitellata (with contributions by Marco Ferraguti). Pp. 235–392. In Reproductive Biology and Phylogeny of Annelida. Series Editor BGM Jamieson. Volume 4. Editors G Rouse, F Pleijel. Science Publishers, Enfield, New Hampshire; Fragoso C, Rojas P (2009) A New Ocnerodrilid Earthworm genus from Southeastern Mexico (Annelida: Oligochaeta), with a key for the genera Of Ocnerodrilidae. Megadrilogica Volume 13 (9): 141–152.; Plisko JD, Nxele TC (2015) An annotated key separating foreign earthworm species from the indigenous South Africian taxa (Oligochaeta: Acanthodrilidae, Eudrilidae, Glossoscolecidae, Lumbricidae, Megascolecidae, Microchaetidae, Ocnerodrilidae and Tritogeniidae). African Invertebrates 56: 663–708; Thorp JH, Lovell LL, Timm T, Martin P, Gelder SR, Govedich FR, Moser WE, Nakano T, Bielecki A, Bain BA, Utevsky S, Gil J, Glasby CJ, Martin D (2019) Phylum Annelida. In: Rogers DC, Thorp JH (Eds) Keys to Palaearctic Fauna: Thorp and Covich’s Freshwater invertebrates - Volume IV. Academic Press, Elsevier, pp. 357–518; Misirlioðlu, M, Reynolds, JW, Stojanoviæ, M, Trakiæ, T, Sekuliæ, J, James, SW, Csuzdi, C, Decaëns, T, Lapied, E, Phillips, HRP, Cameron, EK, Brown, GG (2023). Earthworms (Clitellata, Megadrili) of the world: an updated checklist of valid species and families, with notes on their distribution. Zootaxa 5255 (1): 417–438.

# Oenonidae Kinberg, 1865

**Description**. Body segment number: variable. Body shape elongate, more-or-less equal width over entire length. Body pigmentation absent or present, yellow, orange or brown; epidermis more-or-less smooth (cuticle is often iridescent). Pygidium simple lobe. Pygidial appendages absent or present, one pair of cirri or four cirri. Discrete head present; lobe-like without appendages (1–3 very small antennae may be present in the groove between the prostomium and peristomium).

Prostomium bluntly conical, eyes on head present, one pair or two pairs (when 2 pairs usually 1 pair larger than the other); head eyes simple pigmented cups. Prostomial antennae absent, or present; median and paired laterals; unarticulated. Nuchal organs present; paired low projections from posterolateral prostomium; indistinct dorsolateral ciliated patches. Peristomium a double ring, or a single ring.

Foregut a muscular ventral pharynx. Pharynx jaws absent (rarely, in parasitic forms) or present, multiple jaw elements of different shapes and sizes. Multiple jaws comprising ventral mandibles and dorsal maxillae (maxillae are less developed or even absent in symbiotic forms), 4–5 pairs of toothed plates in parallel rows. Pharynx maxillae asymmetrical (right maxilla 1 transformed to a base plate; right maxilla 3 lacking) or symmetrical. Maxillary carriers longer than combined length of maxillae. Pharynx dorsolateral ciliated folds present.

First segment chaetous; second segment chaetous. First chaetiger with both notochaetae and neurochaetae; parapodia similar in length or slightly shorter than subsequent parapodia; parapodia more-or-less laterally directed and free from head; chaetae similar in orientation, length and thickness to other chaetae. Parapodia uniramous, or biramous (lobes only in notopodia); parapodial lobes prominent. Notopodial lobes represented by at least one chaetal lobe, or absent. Neuropodial lobes represented by at least one chaetal lobe. Lateral organs present (ciliated patch at ventral base of dorsal cirrus). Dorsal cirri present; flattened and foliaceous. Ventral cirri absent (replaced by inflated pads).

Chaetae first appear on first segment after peristomium, arranged as a single bundle (or row) of many chaetae (lower bundle - neurochaetae - only). Aciculae present; in ventral position (=neuroaciculae) (dorsal ones sometimes present). Neuroaciculae distally tapering. Capillary chaetae present, in ventral (neuropodial) position, distally tapered to a point, edge smooth (limbate) or spinose. Capillary chaetae, externally not pseudo-segmented. Capillary chaetae, internally not chambered or hollow. Hooks absent, or present; with a distal hood; occur in ventral (neuropodial) position; occur over entire body.

Tube absent.

**Literature used for description**. Glasby CJ, Fauchald K (2003). POLiKEY. An information system for polychaete families and higher taxa. Version 2, 5 June 2003. Australian Biological Resources Study, DCCEEW, Canberra, https:[//www.dcceew.gov.au/;](http://www.dcceew.gov.au/%3B) Steiner TM (2022) 7.12.7. Oenonidae Kinberg, 1865, In: Purschke G, Böggemann M, Westheide W (Eds), Handbook of Zoology, Annelida, Volume 3: Pleistoannelida, Errantia II. De Gruyter, Berlin, 35–64; Rouse GW, Pleijel, F, Tilic E (2022) Annelida. Oxford University Press, Oxford, UK.

# Onuphidae Kinberg, 1865

**Description**. Body segment number: variable. Body shape elongate, more-or-less equal width over entire length. Body pigmentation absent or present, brown, black or white stripes, speckles or flecks on anterior body; epidermis more-or-less smooth. Pygidium simple lobe. Pygidial appendages present, one pair of cirri or four cirri. Discrete head present; complex in shape bearing appendages.

Prostomium rounded to oval, anteriorly incised (shallow; with two ventrally inflated pad-like lips (= frontal lips) on either side, which have erroneously been called palps in the old literature), frontal lips present (paired, digitiform processes), eyes on head absent or present, two pairs (anterior pair minute); head eyes simple pigmented cups. Prostomial antennae present; median and paired laterals (one pair of lateral antennae; the other shorter lateral pair are palpal antennae); consist of basal ceratophore and distal ceratostyle. Palps present (resemble antennae); anteroventral; tapering (usually) sensory type; biarticulated (basal palpophore ringed, as in the lateral antennae ceratophores). Nuchal organs present; paired low projections from posterolateral prostomium; indistinct dorsolateral ciliated patches. Peristomium a single ring. Peristomial cirri absent, or present, 1 pair.

Gut more-or-less straight, lacking side branches. Foregut a muscular ventral pharynx. Pharynx jaws present, multiple jaw elements of different shapes and sizes. Multiple jaws comprising ventral mandibles and dorsal maxillae, 4–5 (right) and 4–6 (left) toothed plates in a semicircle. Pharynx maxillae asymmetrical (right maxilla 3 missing; right maxilla 4 larger than left one). Maxillary carriers shorter than combined length of maxillae. Pharynx dorsolateral ciliated folds present.

First segment chaetous; second segment chaetous. First chaetiger with neurochaetae only; parapodia similar in length or slightly shorter than subsequent parapodia, or very elongated (first few anterior parapodia may be several times length of more posterior parapodia); parapodia anteriorly directed and wrapping around head, or more-or-less laterally directed and free from head; chaetae similar in orientation, length and thickness to other chaetae, or slender and elongate, forming cage (or basket) around head. Parapodia uniramous. Notopodial lobes absent (dorsal cirrus only present). Neuropodial lobes represented by at least one chaetal lobe. Lateral organs present (as a ciliated patch at ventral base of dorsal cirrus). Dorsal cirri present; more-or-less cirriform. Ventral cirri present; cirriform or tapering (rarely reduced as a ventral glandular pad). Branchiae present (rarely absent), arise from lateral body (upper edge of dorsal cirri), occur on mid-body segments; lateral branchiae digitiform, or branching.

Chaetae first appear on first segment after peristomium, arranged as a single bundle (or row) of many chaetae (lower bundle - neurochaetae - only). Aciculae present; in ventral position (=neuroaciculae), or in both dorsal and ventral positions (=noto- and neuroaciculae). Neuroaciculae distally tapering. Capillary chaetae present, in ventral (neuropodial) position, distally tapered to a point, edge smooth. Capillary chaetae, externally not pseudo-segmented. Capillary chaetae, internally not chambered or hollow. Comb-like chaetae present. Comb-like chaetae comb comprised of many long tines. Hooks present; with a distal hood (rarely unprotected); occur in ventral (neuropodial) position; occur over entire body (neuropodia only). Compound chaetae present; in ventral (neuropodial) position; appendage distally tapering to slender tips (=spinigerous) (rarely), or distally curved (=falcate; ie., one distal tooth) (in anterior parapodia only), or multidentate; appendage not canaliculated; appendage with paired guards on each side of the crest; joint, appearance distinctly asymmetrical (=heterogomph), or flat or pseudocompound (in anterior chaetigers only); joint effected by ligament(s) (most with an incomplete hinge - pseudocompound); shaft tapering slightly or evenly thick from emergence to joint; shaft, internally solid, without distinct core. Compound chaetae present in a few anterior chaetigers only.

Segmental organs are metanephridia.

Tube present; membranous, or leathery or parchment like, or translucent, chitin-like.

**Literature used for description**. Glasby CJ, Fauchald K (2003). POLiKEY. An information system for polychaete families and higher taxa. Version 2, 5 June 2003. Australian Biological Resources Study, DCCEEW, Canberra, https:[//www.dcceew.gov.au/;](http://www.dcceew.gov.au/%3B) Budaeva N (2021) 7.12.3 Onuphidae Kinberg, 1865 In: Purschke G, Böggemann M, Westheide W (Eds), Handbook of Zoology, Annelida, Volume 3: Pleistoannelida, Sedentaria III and Errantia I. De Gruyter, Berlin, 383–413; Rouse GW, Pleijel, F, Tilic E (2022) Annelida. Oxford University Press, Oxford, UK.

# Opheliidae Malmgren, 1867

**Description**. Body segment number: variable (but between 30 to 60 segments in adults). Secondary annulation absent or present. Body shape elongate, more-or-less equal width over entire length or widest anteriorly and tapering posteriorly (preserved specimens often curved), regionalization absent or present (weak; in some genera), regionalization comprising two regions or three regions (demarked by body constrictions and/or presence of papillae). Body pigmentation absent or present, brown brands, speckles, or flecks on dorsal body; epidermis more-or-less smooth. Ventral groove present (rarely absent). Pygidium simple lobe, or elongate hooded anal tube. Pygidial appendages present, single medial cirrus or one pair of cirri or more than four cirri (cirri/papillae may be present inside anal cavity or on rim of anal tube; thicker pygidial lobes may also be present). Discrete head present; lobe-like without appendages.

Prostomium conical, tapering to slender tip, with an anterior tentacle-like projection (='palpode'), eyes on head absent or present, one pair; head eyes simple pigmented cups. Eyes on trunk absent, or present (may be present over several segments). Trunk eyes on lateral body. Nuchal organs present; paired low projections from posterolateral prostomium; posterior projections. Peristomium not visible, or visible (may not be visible in adults). Peristomium a single ring.

Gut more-or-less straight, lacking side branches. Foregut a non-muscular axial pharynx (?and a muscular ventral pharynx in some taxa). Protrusible proboscis present (bubble-like, lobate or with oral tentacles), distal ring of papillae absent. Pharynx dorsolateral ciliated folds present. Heart bodies absent.

First segment chaetous; second segment chaetous. First chaetiger with both notochaetae and neurochaetae; parapodia similar in length or slightly shorter than subsequent parapodia; parapodia more-or-less laterally directed and free from head; chaetae similar in orientation, length and thickness to other chaetae. Parapodia biramous; parapodial lobes absent or very low. Notopodial lobes represented by at least one chaetal lobe. Neuropodial lobes represented by at least one chaetal lobe. Lateral organs present. Branchiae absent or present, arise from lateral body, occur on mid-body segments (usually from chaetiger 2–10) or occur near posterior end; lateral branchiae digitiform, or branching (rarely).

Chaetae first appear on first segment after peristomium, arranged in paired bundles (or rows) of many chaetae. Aciculae absent. Capillary chaetae present, in dorsal (notopodial) position or in ventral (neuropodial) position, distally tapered to a point, edge smooth or hirsute-serrate or spinose (posterior capillaries of *Euzonus* may be knobbed distally and almost spine-like). Capillary chaetae, externally not pseudo-segmented. Capillary chaetae, internally not chambered or hollow. Hooks absent.

Segmental organs are protonephridia, or metanephridia; metanephridia 3–8 pairs, usually in midbody.

Tube absent.

**Literature used for description**. Blake, J (2000). Family Opheliidae Malmgren, 1867. In JA Blake, B Hilbig, PV Scott (Eds.), Taxonomic atlas of the benthic fauna of the Santa Maria Basin and western Santa Barbara Channel, vol. 7 – The Annelida, Part 4. Polychaeta: Flabelligeridae to Sternaspidae (pp. 145–168). Santa Barbara: Santa Barbara Museum of Natural History; Glasby CJ, Fauchald K (2003). POLiKEY. An information system for polychaete families and higher taxa. Version 2, 5 June 2003. Australian Biological Resources Study, DCCEEW, Canberra, https:[//www.dcceew.gov.au/;](http://www.dcceew.gov.au/%3B) Blake, JA, Maciolek NJ (2019) 7.6.1 Opheliidae Malmgren, 1867 In: Purschke G, Böggemann M, Westheide W (Eds), Handbook of Zoology, Annelida, Volume 2: Pleistoannelida, Sedentaria II. De Gruyter, Berlin, 285–311; Rouse GW, Pleijel, F, Tilic E (2022) Annelida. Oxford University Press, Oxford, UK.

# Orbiniidae Hartman, 1942

**Description**. Body segment number: variable. Body shape elongate, more-or-less equal width over entire length, regionalization present (rarely absent), regionalization comprising two regions, regions demarcated by laterally-directed thoracic parapodia and dorsally-directed midbody and abdominal parapodia. Epidermis more-or-less smooth (rarely papillate at the junction of thorax and abdomen in some taxa). Pygidium simple lobe. Pygidial appendages present, one pair of cirri or four cirri. Discrete head present; lobe-like without appendages.

Prostomium conical, tapering to slender tip or bluntly conical or rounded to oval, eyes on head absent or present, one pair; head eyes simple pigmented cups. Nuchal organs present; paired low projections from posterolateral prostomium; indistinct dorsolateral ciliated patches. Peristomium a single ring, or a double ring (rarely).

Gut more-or-less straight, lacking side branches or straight with side branches. Foregut a non-muscular axial pharynx or a muscular ventral pharynx (eversible part is either lobate or branching). Protrusible proboscis present (fleshy and bilobed or branching). Pharynx dorsolateral ciliated folds absent or present.

First segment chaetous; second segment chaetous. First chaetiger with both notochaetae and neurochaetae; parapodia similar in length or slightly shorter than subsequent parapodia; parapodia more-or-less laterally directed and free from head; chaetae similar in orientation, length and thickness to other chaetae. Parapodia biramous (lobes may be more prominent posteriorly, including the presence of 'subpodal lobes' which resemble ventral cirri); parapodial lobes absent or very low. Notopodial lobes represented by at least one chaetal lobe. Neuropodial lobes represented by at least one chaetal lobe. Interramal fleshy process absent, or present. Interramal fleshy process cirrus-like (probably a non-retractile lateral organ). Lateral organs present. Branchiae present (rarely absent), arise from dorsal body, occur on mid-body segments and occur near posterior end (one pair per segment). Dorsal branchiae simple filaments each arising directly from body wall (often ciliated; cylindrical or flattened).

Chaetae first appear on first segment after peristomium, arranged in paired bundles (or rows) of many chaetae. Aciculae absent, or present; in dorsal position (=notoaciculae) (thorax and abdomen; some taxa), or in ventral position (=neuroaciculae) (abdomen only; some taxa). Capillary chaetae present, in dorsal (notopodial) position or in ventral (neuropodial) position, distally tapered to a point or sharply bent, edge hirsute-serrate or crenulate. Capillary chaetae, externally not pseudo-segmented. Capillary chaetae, internally distinctly chambered (=camerated). Crotchet chaetae absent or present (*Questa* only), bifid or trifid. Forked chaetae absent, or present, lyrate, tines more-or-less equal in length. Spines present; slightly curved and more-or-less smooth, or harpoon or arrow-shaped with reverse barbs, or sharply bent (=geniculate) or recurved (may be smooth or ribbed); present in most or all chaetigers; in both dorsal and ventral positions. Hooks absent, or present (rarely); without distal hood, beard or ligament; occur in ventral (neuropodial) position; occur over entire body (abdomen only).

Clitellum absent (although one genus, *Questa*, bears a superficially similar glandular-like epidermal organ). Segmental organs are metanephridia; metanephridia along most of the body; anterior ones for excretion, posterior ones for gamete release; present in most segments of the body.

Tube absent.

**Literature used for description**. Glasby CJ, Fauchald K (2003). POLiKEY. An information system for polychaete families and higher taxa. Version 2, 5 June 2003. Australian Biological Resources Study, DCCEEW, Canberra, https:[//www.dcceew.gov.au/;](http://www.dcceew.gov.au/%3B) Bleidorn C, Helm C (2019)

7.1.4 Orbiniidae Hartman, 1942 In Purschke G, Böggemann M, Westheide W (Eds), Handbook of Zoology, Annelida, Volume 1: Annelida Basal Groups and Pleistoannelida, Sedentaria I. De Gruyter, Berlin, 251–268; Rouse GW, Pleijel, F, Tilic E (2022) Annelida. Oxford University Press, Oxford, UK.

# Orobdellidae Nakano, Zainudin & Hikida, 2012

**Description**. Body segment number: fixed, 34 segments including 2 preoral 'segments' (prostomium and peristomium) and 32 postoral segments. Secondary annulation present (disk-shaped receptors called sensillae mark the middle annulus of each segment); anterior region uniannulate, or biannulate, or triannulate; mid-body 4-annulate, or 6-annulate, or 8-annulate, or more; posterior uniannulate, or biannulate, or 3-annulate; post-anal region absent. Body shape elongate, more-or-less equal width over entire length (when relaxed; but can be dramatically shortened by contraction of longitudinal muscles during movement), dorsoventrally flattened. Anterior end sucker on ventral surface present; not clearly separated from rest of body. Large mouth on ventral surface of sucker. Epidermis papillate (numerous, minute, one row on each annulus). Posterior sucker on ventral surface present; anus positioned near posterior end (dorsally, just before posterior sucker).

Eyes on head present, one pair or two pairs or three pairs (usually 3 pairs arranged on 2 separate segments; occasionally only one pair on posterior SII); head eyes simple pigmented cups.

Gut straight with side branches. Foregut a muscular axial pharynx; muscular axial pharynx not rotated (euthylaematous) (check, some reports say strepsilaematous). Pharynx jaws absent or present, leech-type (either soft-muscular or horny) (as soft muscular 'jaws'); two (duognathous) muscular jaws, or three (trignathous) muscular jaws arranged in a triangle; denticles absent or present, paired, fine teeth or series of soft teeth. Protrusible proboscis absent. Crop present (tubular acaecate). Caeca of foregut absent. Caeca of midgut (=posterior crop caeca) absent. Caeca of hindgut absent. Circulatory system absent.

Sexual reproduction hermaphroditic. Clitellum fully encircles body, in region of male and female pores. Male and female pores separated by 5–6 annuli. Clitellum occurs from X-XIII (usual position for leeches). Gastropores absent, or present (tubular gastroporal duct; one pore opens to the crop); on segment XIII, anterior part. Testes present, many pairs (about 20), in multiple grape-like clusters per segment (= 2 or 3 in each annulus); present in segments XIX to XXIV. Ovaries present, one pair; present in segment XIII. Egg sacs present; globular. Oviduct single, shared oviduct from egg sac. Segmental organs are metanephridia; nephridial pores paired (ventrolateral), present in most segments of the body (17 pairs). Female gonoduct present (inconspicuous). Female pores single, median, in segment XIII, anterior part. Male gonoducts present, with atrium present (weakly muscularized), bilobed. Male pores single, median, in segment XI, posterior part. Penis absent. Prostate gland absent.

**Literature used for description**. Siddall ME, Bely AE, Borda E (2006) Hirudinida Pp. 393–429. In Reproductive Biology and Phylogeny of Annelida. Series Editor BGM Jamieson. Volume 4. Editors G Rouse, F Pleijel. Science Publishers, Enfield, New Hampshire; Nakano, T., Ramlah, Z. & Hikida, T. (2012). Phylogenetic position of gastrostomobdellid leeches (Hirudinida, Arhynchobdellida, Erpobdelliformes) and a new family for the genus *Orobdella*. —Zoologica Scripta, 41, 177–185; Nakano T, Lai Y-T (2012) A new species of *Orobdella* (Hirudinida, Arhynchobdellida, Orobdellidae) from Taipei, Taiwan. ZooKeys 207: 49–63. doi: 10.3897/zookeys.207.3334; Nakano T (2016) Four new species of the genus *Orobdella* from Shikoku and Awajishima island, Japan (Hirudinida, Arhynchobdellida, Orobdellidae). Zoosystematics and Evolution 91: 79–102; Nakano T, Eto K, Nishikawa K, Hossman MY, Jeratthitikule E (2018) Systematic revision of the Southeast Asian macrophagous leeches, with the description of two new gastrostomobdellid species (Hirudinida: Arhynchobdellida: Erpobdelliformes). Zoological Journal of the Linnean Society 184: 1–30. Thorp JH, Lovell LL, Timm T, Martin P, Gelder SR, Govedich FR, Moser WE, Nakano T, Bielecki A, Bain BA, Utevsky S, Gil J, Glasby CJ, Martin D (2019) Phylum Annelida. In: Rogers DC, Thorp JH (Eds) Keys to Palaearctic Fauna: Thorp and Covich’s Freshwater invertebrates - Volume IV. Academic Press, Elsevier, pp. 357–518; Nakano T, Prozorova L (2019). A new species of *Orobdella* (Hirudinida: Arhynchobdellida: Orobdellidae) from Primorye Territory, Russian Far East. Journal of Natural History. 53 (5–6): 351–364.

# Oweniidae Rioja, 1917

**Description**. Body segment number: variable. Body segments strongly elongate in midbody, elongate body segments with distinct (but truncate) notopodia and neuropodial tori (neuropodia with dense fields of very small hooks). Body shape elongate, more-or-less equal width over entire length, regionalization present (but subtle with the anterior region - thorax - comprising only 1–3 segments), regionalization comprising two regions, regions demarcated by change in chaetal types over body (thorax with notochaetae only). Pygidium simple lobe, or with multiple digitate lobes. Pygidial appendages absent or present, one pair of cirri. Discrete head absent (*Owenia* only has a lobed structure resembling tentacles), or present; lobe-like without appendages, or complex in shape bearing appendages.

Prostomium rounded to oval (in taxa where the prostomium is well-defined), eyes on head absent or present (on peristomium), one pair; head eyes simple pigmented cups. Buccal tentacles absent, or present (as a crown); surrounding perimeter of mouth; ciliated. Palps absent, or present; anterodorsal; grooved (usually) feeding type; feeding palp longitudinally grooved. Nuchal organs absent. Peristomium a single ring.

Gut more-or-less straight, lacking side branches. Foregut a muscular ventral pharynx.

Pharynx dorsolateral ciliated folds present.

First segment chaetous. First chaetiger with notochaetae only; parapodia similar in length or slightly shorter than subsequent parapodia; parapodia more-or-less laterally directed and free from head; chaetae similar in orientation, length and thickness to other chaetae. Parapodia uniramous (first 1–3 chaetigers), or biramous (remaining chaetigers); parapodial lobes absent or very low. Notopodial lobes represented by at least one chaetal lobe. Neuropodial lobes low ridges (tori) (throughout).

Chaetae first appear on first segment after peristomium, arranged in paired bundles (or rows) of many chaetae. Aciculae absent. Capillary chaetae present, in dorsal (notopodial) position, distally tapered to a point, edge smooth or spinose. Capillary chaetae, externally not pseudo-segmented. Capillary chaetae, internally not chambered or hollow. Spines absent, or present; slightly curved and more-or-less smooth; present only in posterior chaetigers; in dorsal (notopodial) position only. Hooks present (long handled); without distal hood, beard or ligament; occur in ventral (neuropodial) position; occur over entire body (neuropodia only).

Sexual reproduction gonochoric.

Tube present; membranous (tight-fitting tube covered with overlapping sediment grains; worms difficult to extract because of their numerous parapodial hooks).

**Literature used for description**. Glasby CJ, Fauchald K (2003). POLiKEY. An information system for polychaete families and higher taxa. Version 2, 5 June 2003. Australian Biological Resources Study, DCCEEW, Canberra, https://[www.dcceew.gov.au/;](http://www.dcceew.gov.au/%3B) Capa M, Parapar J, Hutchings P (2012). Phylogeny of Oweniidae (Polychaeta) based on morphological data and taxonomic revision of Australian fauna. Zoological Journal of the Linnean Society 166: 236–278. doi: 10.1111/j.1096–3642.2012.00850.x.; Capa, M, Parapar J, Hutchings P (2019) 4.1 Oweniidae Rioja, 1917 In Purschke G, Böggemann M, Westheide W (Eds), Handbook of Zoology, Annelida, Volume 1: Annelida Basal Groups and Pleistoannelida, Sedentaria I. De Gruyter, Berlin, 91–112; Rouse GW, Pleijel, F, Tilic E (2022) Annelida. Oxford University Press, Oxford, UK.

# Ozobranchidae Pinto, 1921

**Description**. Body segment number: fixed, 34 segments including 2 preoral 'segments' (prostomium and peristomium) and 32 postoral segments (true segments generally obscured by intervening superficial annulations, giving the impression of many more segments). Secondary annulation present (disk-shaped receptors called sensillae mark the middle annulus of each segment); anterior region biannulate (i.e., trachelostome); mid-body biannulate, or triannulate (ie., urostome somites). Body segments similar dimensions throughout. Body shape elongate, more-or-less equal width over entire length (when relaxed; but can be dramatically shortened by contraction of longitudinal muscles during movement), dorsoventrally flattened or more-or-less cylindrical, regionalization present, regionalization comprising two regions (a narrow anterior trachelosome and much wider posterior urosome, which bears branchiae). Anterior end sucker on ventral surface present (surrounding mouth); not clearly separated from rest of body. Small mouth pore on ventral surface of sucker. Epidermis more-or-less smooth, or papillate. Posterior sucker on ventral surface present; anus positioned near posterior end (dorsally, just before posterior sucker). Elongate proboscis present; retractable and tube-like (protruded through a small mouth pore).

Eyes on head absent or present, one pair (on one annulus); head eyes simple pigmented cups.

Gut straight with side branches. Foregut without a distinct ventral or axial pharynx. Pharynx jaws absent. Protrusible proboscis present, smooth, distal ring of papillae absent. Crop present. Caeca of midgut (=posterior crop caeca) present (5 pairs). Circulatory system present.

Branchiae present, arise from lateral body, occur on mid-body segments; lateral branchiae branching (5–20 pairs starting segment XIII).

Sexual reproduction hermaphroditic. Clitellum fully encircles body, in region of male and female pores. Clitellum occurs from X-XIII (usual position for leeches). Gonadal segments bearing a sperm transfer system in copulatory area present (as bilateral coelomic tubes). Testes present, many pairs, one pair per segment; present in segments XIV to XVII (4 pairs). Sperm sac present. Ovaries present, one pair; present in segments XIV to XVI. Egg sacs present; tubular. Oviduct separate one for each egg sac. Vaginal sac present. Segmental organs are metanephridia; nephridial pores single (ventromedial). Female gonoduct present, vaginal sac present. Female pores single, median, in segment XII. Male gonoducts present, with atrium present, bilobed. Male pores single, median, in segment XI. Penis absent. Prostate gland absent.

**Literature used for description**. Sanjeeva Raj PJ (1954) A Synopsis of the Species of the Genus *Ozobranchus* (De Quatrefages 1852) Hirudinea Annelida. The Journal of the Bombay Natural History Society 52: 473–480; Davies RW (1978) The morphology of *Ozobrancghus margoi* (Apathy) (Hirudinoidea), a parasite of marine turtles. Journal of Parisotology 64: 1092–1096; Siddall ME, Bely AE, Borda E (2006) Hirudinida Pp. 393–429. In Reproductive Biology and Phylogeny of Annelida. Series Editor BGM Jamieson. Volume 4. Editors G Rouse, F Pleijel. Science Publishers, Enfield, New Hampshire; Christofferson ML (2008) A Catalogue of the Piscicolidae, Ozobranchidae, and Arhynchobdellida (Annelida, Clitellata, Hirudinea) from South America. Neotropical Biology and Conservation 3(1):39–48; Brusca, RC, Moore, W & Shuster, SM. (eds) (2016) Chapter 14. Phylum Annelida. In: Invertebrates, 3rd Edition. Sinauer Associates, Sunderland, MA; Thorp JH, Lovell LL, Timm T, Martin P, Gelder SR, Govedich FR, Moser WE, Nakano T, Bielecki A, Bain BA, Utevsky S, Gil J, Glasby CJ, Martin D (2019) Phylum Annelida. In: Rogers DC, Thorp JH (Eds) Keys to Palaearctic Fauna: Thorp and Covich’s Freshwater invertebrates - Volume IV. Academic Press, Elsevier, pp. 357–518; Burreson EM (2020) Marine and estuarine leeches (Hirudinida : Ozobranchidae and Piscicolidae) of Australia and New Zealand with a key to the species. Invertebrate Systematics 34: 235–259; Koo KS, Yun K, Jang Y (2022) *Ozobranchus jantseanus* (Clitellata: Ozobranchidae) from Reeve’s Turtle, *Mauremys reevesii*: New Annelid Fauna in Korea. The Korean Journal of Parisotology 60: 213–215.

# Paralacydoniidae Pettibone, 1963

**Description**. Body segment number: variable. Body shape elongate, more-or-less equal width over entire length. Epidermis more-or-less smooth. Pygidium simple lobe. Pygidial appendages present, one pair of cirri and single medial papilla. Discrete head present; complex in shape bearing appendages.

Prostomium bluntly conical, eyes on head absent or present, one pair; head eyes simple pigmented cups. Prostomial antennae present; paired, lateral; unarticulated. Palps present (resemble antennae); anteroventral; tapering (usually) sensory type; biarticulated. Nuchal organs present; paired low projections from posterolateral prostomium; indistinct dorsolateral ciliated patches. Peristomium not visible.

Foregut a muscular axial pharynx. Protrusible proboscis present, smooth, distal ring of papillae present.

First segment achaetous (uncertain; Wilson (2000) observed that the first segment is chaetigerous); second segment chaetous. Tentacular cirri absent. First chaetiger with neurochaetae only; parapodia similar in length or slightly shorter than subsequent parapodia; parapodia more-or-less laterally directed and free from head; chaetae similar in orientation, length and thickness to other chaetae. Parapodia biramous; parapodial lobes prominent. Notopodial lobes represented by at least one chaetal lobe. Neuropodial lobes represented by at least one chaetal lobe. Interramal fleshy process absent (though interramal cilia present). Dorsal cirri present; more-or-less cirriform. Ventral cirri present; cirriform or tapering.

Chaetae first appear on second segment after peristomium, arranged in paired bundles (or rows) of many chaetae. Aciculae present; in both dorsal and ventral positions (=noto- and neuroaciculae). Capillary chaetae present, in dorsal (notopodial) position, distally tapered to a point, edge hirsute-serrate. Capillary chaetae, externally not pseudo-segmented. Capillary chaetae, internally not chambered or hollow. Hooks absent. Compound chaetae present; in ventral (neuropodial) position; appendage distally tapering to slender tips (=spinigerous); appendage not canaliculated; appendage without hoods or guards; joint, appearance distinctly asymmetrical (=heterogomph); joint effected by ligament(s); shaft tapering slightly or evenly thick from emergence to joint; shaft, internally solid, without distinct core. Compound chaetae present in most or all chaetigers.

Tube absent.

**Literature used for description**. Glasby CJ, Fauchald K (2003). POLiKEY. An information system for polychaete families and higher taxa. Version 2, 5 June 2003. Australian Biological Resources Study, DCCEEW, Canberra, https:[//www.dcceew.gov.au/;](http://www.dcceew.gov.au/%3B) Rizzo AE, Magalhães WF (2022) 7.13.7Paralacydoniidae Pettibone, 1963 In: Purschke G, Böggemann, M, Westheide W (Eds) Handbook of Zoology. Annelida. Vol. 4: Pleistoannelida, Errantia II. De Gruyter, Berlin, 376–380; Rouse GW, Pleijel, F, Tilic E (2022) Annelida. Oxford University Press, Oxford, UK.

# Paraonidae Cerruti, 1909

**Description**. Body segment number: variable. Body shape elongate, more-or-less equal width over entire length (thin, fragile), regionalization present, regionalization comprising three regions (pre-branchial, branchial and postbranchial regions). Epidermis more-or-less smooth. Pygidium simple lobe. Pygidial appendages present, one pair of cirri or more than four cirri or three cirri (typical). Discrete head present; lobe-like without appendages, or complex in shape bearing appendages.

Prostomium bluntly conical, with an anterior tentacle-like projection (='palpode'; not readily apparent in fixed material as it is eversible), eyes on head absent or present, one pair; head eyes simple pigmented cups. Prostomial antennae absent, or present; median one only (may be absent); unarticulated. Nuchal organs present; paired low projections from posterolateral prostomium; indistinct dorsolateral ciliated patches. Peristomium not visible (although a thin annulus resembling a peristomium can sometimes be seen in SEMs).

Gut more-or-less straight, lacking side branches. Foregut a non-muscular axial pharynx (although some taxa were considered to have a muscular ventral pharynx; Strelzov 1979)). Pharynx dorsolateral ciliated folds absent or present.

First segment chaetous; second segment chaetous. First chaetiger with both notochaetae and neurochaetae; parapodia similar in length or slightly shorter than subsequent parapodia; parapodia more-or-less laterally directed and free from head; chaetae similar in orientation, length and thickness to other chaetae. Parapodia biramous; parapodial lobes absent or very low. Notopodial lobes represented by at least one chaetal lobe. Neuropodial lobes represented by at least one chaetal lobe. Lateral organs absent, or present. Branchiae present, arise from dorsal body, occur on mid-body segments (limited region). Dorsal branchiae simple filaments each arising directly from body wall (may be flattened).

Chaetae first appear on first segment after peristomium, arranged in paired bundles (or rows) of many chaetae. Aciculae absent. Capillary chaetae present, in dorsal (notopodial) position or in ventral (neuropodial) position, distally tapered to a point, edge smooth or hirsute-serrate. Capillary chaetae, externally not pseudo-segmented. Capillary chaetae, internally not chambered or hollow. Forked chaetae absent, or present, lyrate, tines distinctly unequal in length. Spines present; slightly curved and more-or-less smooth, or with a single distal or subdistal hair (=aristate or hooded); present in most or all chaetigers; in both dorsal and ventral positions. Hooks absent, or present (rarely); with a distal hood; occur in ventral (neuropodial) position; occur over entire body.

Sexual reproduction gonochoric. Segmental organs are metanephridia; metanephridia restricted to anterior and midbody segments.

Tube absent.

**Literature used for description**. Strelzov VE (1979) Polychaete Worms of the Family Paraonidae Cerrui, 1909: (Polychaeta, Sedentaria). New Delhi, Amerind Publishing Co. Pvt Ltd,, 212 pp; Glasby CJ, Fauchald K (2003). POLiKEY. An information system for polychaete families and higher taxa. Version 2, 5 June 2003. Australian Biological Resources Study, DCCEEW, Canberra, https:[//www.dcceew.gov.au/;](http://www.dcceew.gov.au/%3B) Blake JA (2019) Paraonidae Cerruti, 1909 In Purschke G, Böggemann M, Westheide W (Eds), Handbook of Zoology, Annelida, Volume 1: Annelida Basal Groups and Pleistoannelida, Sedentaria I. De Gruyter, Berlin, 281–308; Rouse GW, Pleijel, F, Tilic E (2022) Annelida. Oxford University Press, Oxford, UK.

# Parergodrilidae Reisinger, 1925

**Description**. Body segment number: fixed, less than 14 segments (8 or 9 chaetigers for *Parergodrilus*, and a max. 10 chaetigers for *Stygocapitella*). Body shape sausage or grub-shaped. Body translucent, gut visible; epidermis more-or-less smooth. Pygidium simple lobe, or bilobed. Pygidial appendages absent. Discrete head present; lobe-like without appendages.

Prostomium rounded to oval. Nuchal organs absent, or present (*Stygocapitella*); paired low projections from posterolateral prostomium; indistinct dorsolateral ciliated patches. Peristomium a single ring.

Gut straight except for a large midbody loop. Foregut a muscular ventral pharynx. Heart bodies present, around the oesophagus.

First segment achaetous; second segment chaetous. First chaetiger with neurochaetae only; parapodia similar in length or slightly shorter than subsequent parapodia; parapodia more-or-less laterally directed and free from head; chaetae similar in orientation, length and thickness to other chaetae. Parapodia absent.

Chaetae first appear on second segment after peristomium, arranged as a single bundle (or row) of many chaetae (each with 2–6 chaetae). Aciculae absent. Capillary chaetae present, in ventral (neuropodial) position, distally tapered to a point or expanded (both types present in a chaetiger), edge smooth. Capillary chaetae, externally not pseudo-segmented. Capillary chaetae, internally not chambered or hollow. Forked chaetae absent, or present, furcate, tines more-or-less equal in length (outer-most ones thicker than inner ones). Genital chaetae present (copulatory chaetae present in posterior end of males of *Parergodrilus*); hooks absent.

Sexual reproduction gonochoric (internal fertilisation). Testes present, one pair in total; present in segment 8 (chaetiger 7). Sperm sac present. Ovaries present, one pair. Egg sacs present. Oviduct separate one for each egg sac. Segmental organs are metanephridia; metanephridia 2 pairs only (*Parergodrilus*) or many distributed along whole body (*Stygocapitella*); nephridial pores paired (ventrolateral). Spermathecae present, paired, spermathecal pores, 1 pair; in segment chaetigers 6–7 or 9–10. Female gonoduct present. Female pores one pair, in segment between chaetigers 6–7 in *Parergodrilus* and 9–10 in *Stygocapitella*. Male gonoducts present, with atrium present. Penis absent or present (=everted atrium; in Parergodrilus). Prostate gland present, one pair. Prostate pores in segment chaetiger 9; prostate pore and male pore on segment XVII united, discharge through single pore.

Tube absent.

**Literature used for description**. Glasby CJ, Fauchald K (2003). POLiKEY. An information system for polychaete families and higher taxa. Version 2, 5 June 2003. Australian Biological Resources Study, DCCEEW, Canberra, https:[//www.dcceew.gov.au/;](http://www.dcceew.gov.au/%3B) Purschke G (2019) 7.1.3 Parergodrilidae Reisinger, 1925 In Purschke G, Böggemann M, Westheide W (Eds), Handbook of Zoology, Annelida, Volume 1: Annelida Basal Groups and Pleistoannelida, Sedentaria I. De Gruyter, Berlin, 237–250; Rouse GW, Pleijel, F, Tilic E (2022) Annelida. Oxford University Press, Oxford, UK.

# Parvidrilidae Erséus, 1999

**Description**. Body segment number: variable. Secondary annulation absent or present. Body shape elongate, more-or-less equal width over entire length (small worm). Pygidium simple lobe. Pygidial appendages absent.

Prostomium bluntly conical, zygolobic (prostomium not demarcated).

Foregut a muscular dorsal pharynx.

Chaetae first appear on second segment after peristomium (both dorsal and ventral positions; =S3 for oligochaete workers), in lumbricine arrangement - paired upper and lower bundles of one or a few chaetae each - closely spaced lateral and ventrolateral pairs. Capillary (=hair) chaetae present, in both dorsal and ventral positions, distally tapered to a point (dorsal and ventral ones may be very long - greater than body width), edge smooth. Crotchet chaetae present (ventral position only), simple or bifid (some lack a nodulus). Needle chaetae present (dorsal position only), single-pointed or bifid.

Sexual reproduction hermaphroditic. Clitellum thin, single layered, partially encircles body, in region of male and female pores. Clitellum occurs from IX to

XIV. Testes present, one pair in total; present in segment XI. Ovaries present; present in segment XII. Segmental organs are metanephridia; both nephridial pores and gonoducts located around clitellum. Spermathecae present, post-testicular. Spermathecal pores present, located within 1 or 2 segments of male pores; 1 pair; in segment XIII. Female gonoduct present. Female pores one pair, in segment XIII. Male gonoducts present. Male pores single, median, in segment XII or XIII (penis present), plesioporous (in segment following testicular segment). Penis present. Prostate gland absent.

**Literature used for description**. Martinez-Ansemil, E, Creuzé des Châtelliers M, Martin P, Sambugar B (2012). The Parvidrilidae - a diversified groundwater family: description of six new species from southern Europe, and clues for its phylogenetic position within Clitellata (Annelida). Zoological Journal of the Linnean Society 166 (3): 530–558; Jamieson, BGM (2006) Non-leech Clitellata (with contributions by Marco Ferraguti). Pp. 235–392. In Reproductive Biology and Phylogeny of Annelida. Series Editor BGM Jamieson. Volume 4. Editors G Rouse, F Pleijel. Science Publishers, Enfield, New Hampshire; Timm, T (2009) A guide to the freshwater Oligochaeta and Polychaeta of Northern and Central Europe. Lauterbornia 66: 1–235; Thorp JH, Lovell LL, Timm T, Martin P, Gelder SR, Govedich FR, Moser WE, Nakano T, Bielecki A, Bain BA, Utevsky S, Gil J, Glasby CJ, Martin D (2019) Phylum Annelida. In: Rogers DC, Thorp JH (Eds) Keys to Palaearctic Fauna: Thorp and Covich’s Freshwater invertebrates - Volume IV. Academic Press, Elsevier, pp. 357–518.

# Pectinariidae Quatrefages, 1866

**Description**. Body segment number: variable. Secondary annulation present. Body shape widest anteriorly and tapering posteriorly, regionalization present, regionalization comprising three regions, regions demarcated by structural differences in parapodia over body (a thorax comprising 5 segments; an abdomen with 12–14 biramous chaetigers; and a posterior scaphe, a short, flattened or frilly, achaetous region). Body translucent, gut visible; epidermis more-or-less smooth. Thoracic ventral glandular areas present; indistinct mid-ventral swelling (=ventral pads); caudal region short, few segments, mostly achaetous, with frilly lobes (= scaphe). Pygidium anal flap or ligule. Pygidial appendages absent or present, more than four cirri. Discrete head absent (semi-circular region bearing a smooth or fringed veil derived from prostomium, peristomium and anterior segments).

Caudal eyes absent, or present (one pair). Buccal tentacles present; arising from one side of mouth (several short ventral tentacles). Nuchal organs present (laying lateral to the mouth). Peristomium not visible.

Gut straight except for a large midbody loop. Foregut a muscular ventral pharynx. Pharynx dorsolateral ciliated folds present. Heart bodies present.

First segment achaetous (not visible as it is incorporated into the head as the operculum); second segment achaetous (not visible as it is incorporated into the head as the operculum). First chaetiger with notochaetae only; parapodia very elongated; parapodia anteriorly directed and wrapping around head (golden paleae); chaetae projecting obliquely, distinctly thicker and more shiny than subsequent ones (=paleae). Parapodia biramous (thorax), or uniramous (abdomen); parapodial lobes prominent. Notopodial lobes represented by at least one chaetal lobe. Neuropodial lobes low ridges (tori) (over much of body). Lateral organs present. Branchiae present, arise from dorsal body, occur first few segments (2 and 3). Dorsal branchiae simple filaments each arising directly from body wall and lamellate (first two pairs digitiform (sometimes called buccal cirri); second two pairs lamellate).

Chaetae first appear on first segment after peristomium (ie, the paleae), arranged in paired bundles (or rows) of many chaetae. Aciculae absent. Capillary chaetae present, in dorsal (notopodial) position, distally tapered to a point, edge smooth or hirsute-serrate. Capillary chaetae, externally not pseudo-segmented. Capillary chaetae, internally not chambered or hollow. Paleate chaetae present, associated with head (fan-shaped group of golden cephalic paleae arising from segment 1). Spines absent, or present; sharply bent (=geniculate) or recurved; present only in posterior chaetigers (in pre-pygidial segments fused to scaphe); in dorsal (notopodial) position only. Hooks absent. Uncini present; with teeth in vertical series, teeth usually similar-sized (=pectinate); in ventral (neuropodial) position throughout; arranged in more than two rows.

Segmental organs are metanephridia; metanephridia several pairs metanephridia anteriorly for excretion, posterior ones for gamete release (4–7 pairs); several pairs in anterior body.

Tube present; close-fitting, uncemented, sand grains forming tubes that are cone or tusk shaped (grains arranged in a single layer); entrance able to be plugged by worm (fan-shaped paleae seals the tube).

**Literature used for description**. Glasby CJ, Fauchald K (2003). POLiKEY. An information system for polychaete families and higher taxa. Version 2, 5 June 2003. Australian Biological Resources Study, DCCEEW, Canberra, https:[//www.dcceew.gov.au/;](http://www.dcceew.gov.au/%3B) Stiller J, Tilic E, Rousset V, Pleijel F and Rouse GW (2020). Spaghetti to a Tree: A Robust Phylogeny for Terebelliformia (Annelida) Based on Transcriptomes, Molecular and Morphological Data. Biology 9, 73; doi:10.3390/biology9040073; Hutchings P, Carrerette O, Nogueira JM de M (2021) 7.7.1 Pectinariidae Quatrefages, 1866 In: Purschke G, Böggemann M, Westheide W (Eds), Handbook of Zoology, Annelida, Volume 3: Pleistoannelida, Sedentaria III and Errantia I. De Gruyter, Berlin, 34–49; Rouse GW, Pleijel, F, Tilic E (2022) Annelida. Oxford University Press, Oxford, UK.

# Phreodrilidae Beddard, 1891

**Description**. Body segment number: variable. Secondary annulation absent or present. Body shape elongate, more-or-less equal width over entire length. Pygidium simple lobe. Pygidial appendages absent.

Prostomium bluntly conical, with an anterior tentacle-like projection (='proboscis'; rarely), zygolobic (prostomium not demarcated).

Foregut a muscular dorsal pharynx.

Branchiae absent or present (rarely), arise from lateral body, occur near posterior end.

Chaetae two or more per bundle (up to 4 per bundle) (dorsally), or two per bundle (ventrally), first appear on second or third segment after peristomium (dorsal bundle) or first segment after peristomium (ventral bundle), in lumbricine arrangement. Capillary (=hair) chaetae absent or present (often with basal support chaetae on either side), in dorsal (notopodial) position, distally tapered to a point, edge smooth (rarely with plumose tips or an annulated shaft). Crotchet chaetae present (usually with nodulus), simple or bifid (usually, but upper tooth rudimentary). Needle chaetae present (accompany the capillary chaetae). Genital chaetae present (spermathecal chaetae present ventrally on XII-XIII).

Sexual reproduction hermaphroditic. Clitellum thin, single layered, in region of male and female pores. Clitellum occurs from XI-XIII. Testes present, one pair in total; present in segments usually XI. Sperm sac present (usually). Ovaries present, one pair; present in segment XII, usually. Egg sacs present (usually). Segmental organs are metanephridia (nephridia usually present on one side of the body only, or a single large nephridium in VII), nephridial pores located anteriorly, gonoducts located around clitellum. Spermathecae present, post-testicular. Spermathecal pores present, located within 1 or 2 segments of male pores (usually in segment preceding or following male pore segment); 1 pair; in segment XIII. Female gonoduct present. Female pores one pair, in segment XIII. Male gonoducts present. Male pores one pair, in segment XI or XII, rarely segments VIII-X, plesioporous (in segment following testicular segment). Penis present (eversible or pendant). Prostate gland absent.

**Literature used for description**. Brinkhurst RO and Jamieson, BGM (1971) Aquatic Oligochaeta of the World with contributions by DG Cook, DV Anderson, J van der Land, University of Toronto Press, Toronto, Canada, 860 pp; Jamieson, BGM (2006) Non-leech Clitellata (with contributions by Marco Ferraguti). Pp. 235–392. In Reproductive Biology and Phylogeny of Annelida. Series Editor BGM Jamieson. Volume 4. Editors G. Rouse, F. Pleijel. Science Publishers, Enfield, New Hampshire; Timm, T (2009) A guide to the freshwater Oligochaeta and Polychaeta of Northern and Central Europe. Lauterbornia 66: 1–235; Pinder, A (2013). Tools for identifying Australian aquatic oligochaetes of the families Phreodrilidae, Lumbriculidae and Capilloventridae (Clitellata: Annelida). Museum Victoria Science Reports 18: 1–20; Thorp JH, Lovell LL, Timm T, Martin P, Gelder SR, Govedich FR, Moser WE, Nakano T, Bielecki A, Bain BA, Utevsky S, Gil J, Glasby CJ, Martin D (2019) Phylum Annelida. In: Rogers DC, Thorp JH (Eds) Keys to Palaearctic Fauna: Thorp and Covich’s Freshwater invertebrates - Volume IV. Academic Press, Elsevier, pp. 357–518.

# Phyllodocidae [Örsted, 1843](https://www.marinespecies.org/aphia.php?p=taxdetails&id=130500) sensu lato

**Description**. Body segment number: variable. Body shape elongate, more-or-less equal width over entire length (often very long and thin). Body pigmentation present, often different hues of yellow, green and blue as base colour, but orange stripes also occur; epidermis more-or-less smooth. Pygidium simple lobe. Pygidial appendages present, one pair of cirri or one pair of cirri and single medial papilla. Discrete head present; complex in shape bearing appendages.

Prostomium bluntly conical, eyes on head present, one pair; head eyes compound, with lenses. Prostomial antennae present; paired, lateral, or median and paired laterals; unarticulated. Palps present (resemble antennae); anteroventral; tapering (usually) sensory type; unarticulated. Nuchal organs present; single antenna-like projection from posterior prostomium and paired low projections from posterolateral prostomium; indistinct dorsolateral ciliated patches, or posterior projections. Peristomium not visible.

Foregut a muscular axial pharynx. Protrusible proboscis present, smooth or with papillae in subterminal position, irregularly arranged or in distinct rows, distal ring of papillae present.

First segment tentaculate; second segment chaetous and tentaculate. Tentacular cirri present; 2–4 pair(s); arise on a single segment, or over two or more segments (segments often fused); internal aciculae absent. First chaetiger with neurochaetae only; parapodia similar in length or slightly shorter than subsequent parapodia; parapodia more-or-less laterally directed and free from head; chaetae similar in orientation, length and thickness to other chaetae. Parapodia uniramous. Notopodial lobes absent (though dorsal cirri are present). Neuropodial lobes represented by at least one chaetal lobe. Dorsal cirri present; flattened and foliaceous. Ventral cirri present; cirriform or tapering, or foliaceous.

Chaetae first appear on second segment after peristomium or third segment after peristomium or fourth segment after peristomium, arranged in paired bundles (or rows) of many chaetae or as a single bundle (or row) of many chaetae (lower bundle - neurochaetae - only). Aciculae present; in ventral position (=neuroaciculae). Neuroaciculae distally tapering. Capillary chaetae absent or present, in dorsal (notopodial) position or in ventral (neuropodial) position, distally tapered to a point, edge smooth. Capillary chaetae, externally not pseudo-segmented. Capillary chaetae, internally not chambered or hollow. Hooks absent. Compound chaetae present; in ventral (neuropodial) position; appendage distally tapering to slender tips (=spinigerous); appendage not canaliculated; appendage without hoods or guards; joint, appearance distinctly asymmetrical (=heterogomph); joint effected by ligament(s); shaft distinctly inflated distally near joint; shaft, internally solid, without distinct core. Compound chaetae present in most or all chaetigers.

Segmental organs are protonephridia. Tube absent.

**Literature used for description**. Glasby CJ, Fauchald K (2003). POLiKEY. An information system for polychaete families and higher taxa. Version 2, 5 June 2003. Australian Biological Resources Study, DCCEEW, Canberra, https:[//www.dcceew.gov.au/;](http://www.dcceew.gov.au/%3B) Rouse GW, Pleijel, F, Tilic E (2022) Annelida. Oxford University Press, Oxford, UK.

# Phyllodocidae, Eteoninae, Alciopini Ehlers, 1864

**Description**. Body segment number: variable. Body shape elongate, more-or-less equal width over entire length. Body translucent, gut visible (except for the largest species which are opaque), pigmentation present, pigmentation often different hues of yellow, green and brown as base colour. Bioluminescence absent, or present (questionably in *Alciopina* and *Rhynchonereella*). Pygidium simple lobe. Pygidial appendages present, one pair of cirri or one pair of cirri and single medial papilla. Discrete head present; complex in shape bearing appendages.

Prostomium rounded to oval, eyes on head present, one pair; head eyes compound, with lenses (large, protruding orange-red eyes, occupying over half dorsal surface of head). Prostomial antennae present; median and paired laterals; unarticulated. Palps present (resemble antennae); anteroventral; tapering (usually) sensory type; unarticulated. Nuchal organs present; paired low projections from posterolateral prostomium; indistinct dorsolateral ciliated patches. Peristomium not visible.

Foregut a muscular axial pharynx. Protrusible proboscis present, smooth or with papillae in subterminal position, irregularly arranged or in distinct rows, distal ring of papillae present.

First segment tentaculate; second segment chaetous and tentaculate. Tentacular cirri present; 3–5 pair(s); arise over two or more segments; internal aciculae absent. First chaetiger with neurochaetae only; parapodia similar in length or slightly shorter than subsequent parapodia; parapodia more-or-less laterally directed and free from head; chaetae similar in orientation, length and thickness to other chaetae. Parapodia uniramous. Notopodial lobes absent (though dorsal cirri are present). Neuropodial lobes represented by at least one chaetal lobe. Dorsal cirri present; flattened and foliaceous. Ventral cirri present; cirriform or tapering.

Chaetae first appear on second segment after peristomium or third segment after peristomium or fourth segment after peristomium or fifth segment after peristomium, arranged as a single bundle (or row) of many chaetae (lower bundle - neurochaetae - only). Aciculae present; in ventral position (=neuroaciculae). Neuroaciculae distally tapering. Capillary chaetae present, in ventral (neuropodial) position, distally tapered to a point or with subdistal spur, edge smooth. Capillary chaetae, externally not pseudo-segmented. Capillary chaetae, internally not chambered or hollow. Hooks absent. Compound chaetae present; in ventral (neuropodial) position; appendage distally tapering to slender tips (=spinigerous); appendage not canaliculated; appendage without hoods or guards; joint, appearance distinctly asymmetrical (=heterogomph); joint effected by ligament(s); shaft tapering slightly or evenly thick from emergence to joint; shaft, internally solid, without distinct core. Compound chaetae present in most or all chaetigers.

Segmental organs are protonephridia. Tube absent.

**Literature used for description**. Glasby CJ, Fauchald K (2003). POLiKEY. An information system for polychaete families and higher taxa. Version 2, 5 June 2003. Australian Biological Resources Study, DCCEEW, Canberra, https:[//www.dcceew.gov.au/](http://www.dcceew.gov.au/) [as Alciopidae]; Rouse GW, Pleijel, F, Tilic E (2022) Annelida. Oxford University Press, Oxford, UK.

# Pilargidae Saint-Joseph, 1899

**Description**. Body segment number: variable. Body shape elongate, more-or-less equal width over entire length, dorsoventrally flattened or more-or-less cylindrical. Body pigmentation absent or present, some pilargids (eg., *Hermundura*, *Pilargis*) have dorsal pigment spots; epidermis more-or-less smooth (cuticle iridescent in some genera), or papillate (verrucae very obvious in some genera). Pygidium simple lobe. Pygidial appendages present, one pair of cirri. Discrete head present; lobe-like without appendages, or complex in shape bearing appendages.

Prostomium rounded to oval, eyes on head absent or present, one pair; head eyes simple pigmented cups. Postcephalic eyes absent, or present; subepidermal (usually one pair on posterior brain lobes). Prostomial antennae absent, or present; paired, lateral, or median and paired laterals; unarticulated. Palps present (may be reduced or fused or papilla-like); anteroventral; tapering (usually) sensory type; unarticulated (occasionally weakly biarticulated, eg., *Synelmis*). Nuchal organs present; paired low projections from posterolateral prostomium; indistinct dorsolateral ciliated patches. Peristomium not visible.

Gut more-or-less straight, lacking side branches or straight with side branches (segmental caecae reported in *Sigambra grubii* (Pettibone, 1966) and *Antonbruunia*). Foregut a muscular axial pharynx. Pharynx jaws absent (although sclerotised structures may resemble jaws; Cabira was incorrectly stated to have jaws (Licher & Westheide 1994)). Protrusible proboscis present, smooth (poorly known sclerotised structures have been reported), distal ring of papillae absent or present.

First segment tentaculate, or achaetous (rarely); second segment chaetous. Tentacular cirri present; 2 pair(s); arise on a single segment; internal aciculae absent. First chaetiger with neurochaetae only; parapodia similar in length or slightly shorter than subsequent parapodia; parapodia more-or-less laterally directed and free from head; chaetae similar in orientation, length and thickness to other chaetae. Parapodia biramous; parapodial lobes absent or very low, or prominent. Notopodial lobes represented by at least one chaetal lobe. Neuropodial lobes represented by at least one chaetal lobe. Dorsal cirri present; more-or-less cirriform (positioned above or below notoacicula). Ventral cirri present; cirriform or tapering.

Chaetae first appear on second segment after peristomium, arranged in paired bundles (or rows) of many chaetae. Aciculae present; in both dorsal and ventral positions (=noto- and neuroaciculae) (several neuroaciculae in *Antonbruunia*). Neuroaciculae distally tapering. Capillary chaetae present, in dorsal (notopodial) position or in ventral (neuropodial) position, distally tapered to a point or expanded (bifid in *Antonbruunia*), edge smooth or hirsute-serrate. Capillary chaetae, externally not pseudo-segmented. Capillary chaetae, internally not chambered or hollow. Spines absent, or present; slightly curved and more-or-less smooth, or sharply bent (=geniculate) or recurved; present in most or all chaetigers; in dorsal (notopodial) position only. Hooks absent, or present (very prominent in *Sigambra*); without distal hood, beard or ligament; occur in dorsal (notopodial) position; occur over entire body.

Tube absent.

**Literature used for description**. Glasby CJ, Fauchald K (2003). POLiKEY. An information system for polychaete families and higher taxa. Version 2, 5 June 2003. Australian Biological Resources Study, DCCEEW, Canberra, https:[//www.dcceew.gov.au/;](http://www.dcceew.gov.au/%3B) Mackie ASY, Oliver, PG, Nygren A (2015) *Antonbruunia sociabilis* sp. nov. (Annelida: Antonbruunidae) associated with the chemosynthetic deep-sea bivalve *Thyasira scotiae* Oliver & Drewery, 2014, and a re-examination of the systematic affinities of Antonbruunidae. Zootaxa 3995 (1): 20–36; Glasby CJ, Salazar-Vallejo SI (2022) 7.13.3.4 Pilargidae Saint-Joseph, 1899, In: Purschke G, Böggemann M, Westheide W (Eds), Handbook of Zoology, Annelida, Volume 3: Pleistoannelida, Errantia II. De Gruyter, Berlin, 308–323; Aguado M (2022) 7.13.12 Antonbruunidae Fauchald, 1977 In: Purschke G, Böggemann, M, Westheide W (Eds) Handbook of Zoology. Annelida. Vol. 4: Pleistoannelida, Errantia II. De Gruyter, Berlin, 394–399; Rouse GW, Pleijel, F, Tilic E (2022) Annelida. Oxford University Press, Oxford; Huč S, Hiley AS, McCowin, MF, Rouse GW (2023) A Mitogenome-Based Phylogeny of Pilargidae (Phyllodocida, 2 Polychaeta, Annelida) and Evaluation of the Position of *Antonbruunia*. Diversity, in press.

# Piscicolidae Johnston, 1865. Revised

**Description**. Body segment number: fixed, 34 segments including 2 preoral 'segments' (prostomium and peristomium) and 32 postoral segments (true segments generally obscured by intervening superficial annulations (3–16), giving the impression of many more segments). Secondary annulation present (disk-shaped receptors called sensillae mark the middle annulus of each segment); mid-body 4-annulate, or 5-annulate, or 6-annulate, or 7-annulate (presence of subannuli may give the appearance of more). Body segments similar dimensions throughout. Body shape elongate, more-or-less equal width over entire length (when relaxed; but can be dramatically shortened by contraction of longitudinal muscles during movement), more-or-less cylindrical, regionalization present, regionalization comprising two regions (usually changing at segment 13 from narrow anterior region (=neck or trachelsome) to wider posterior region (trunk or urosome)). Anterior end sucker on ventral surface present (bell-shaped; distinct from body); clearly separated from rest of body. Small mouth pore on ventral surface of sucker. Body pigmentation absent or present, brown bands, blotches or stripes over a light base colour, or base colour dark brown/black; epidermis more-or-less smooth, or tuberculate, with bumps of various sizes and arrangements; tuberculae arrangement in transverse rows. Posterior sucker on ventral surface present; circular; rays absent, or present (pigmented, with numerous broad radial bands); anus positioned near posterior end (dorsally, just before posterior sucker). Elongate proboscis present; retractable and tube-like (protruded through a small mouth pore).

Eyes on head absent (rarely) or present, two pairs or three pairs or four pairs (usually over 2–4 segments); head eyes simple pigmented cups. Postcephalic eyes absent, or present (on neck); epidermal. Eyes on trunk absent, or present. Trunk eyes on dorsal surface (arranged transversely). Caudal eyes absent, or present (about 10 eyespots sometimes present on posterior sucker just in from its perimeter).

Gut straight with side branches. Foregut without a distinct ventral or axial pharynx. Pharynx jaws absent. Protrusible proboscis present, distal ring of papillae absent. Crop present. Caeca of midgut (=posterior crop caeca) present (2 pairs; free or fused). Caeca of hindgut present (or walls strongly folded). Circulatory system present.

Branchiae absent or present (11–12 pairs gills which have 'pulsatile vesicles' at their bases which function to circulate coelomic fluid through the gills), arise from lateral body, occur on mid-body segments; lateral branchiae digitiform (or leaf-like).

Sexual reproduction hermaphroditic. Clitellum on trachelstome, fully encircles body, in region of male and female pores. Male and female pores separated by variable annuli (number of). Clitellum occurs from X-XIII (usual position for leeches). Gonadal segments bearing a sperm transfer system in copulatory area present (as parenchymal conducting tissue). Testes present (=testisacs), many pairs (5–6 pairs), one pair per segment; present in segments XIII to XVIII. Ovaries present, one pair; present in segments XIII. Egg sacs present; tubular. Oviduct separate one for each egg sac. Segmental organs are metanephridia; nephridial pores paired (ventrolateral) or single (ventromedial). Female gonoduct present. Female pores single, median, in segment XII. Male gonoducts present, with atrium present, bilobed. Male pores single, median, in segment XI. Penis absent. Prostate gland absent.

**Literature used for description**. Siddall ME, Bely AE, Borda E (2006) Hirudinida Pp. 393–429. In Reproductive Biology and Phylogeny of Annelida. Series Editor BGM Jamieson. Volume 4. Editors G Rouse, F Pleijel. Science Publishers, Enfield, New Hampshire; Christofferson ML (2008) A Catalogue of the Piscicolidae, Ozobranchidae, and Arhynchobdellida (Annelida, Clitellata, Hirudinea) from South America. Neotropical Biology and Conservation 3(1):39–48; Bielecki A, Cios S, Cichocka JM, Pakulnicka J (2012) *Piscicola siddalli* n. sp., a Leech Species from the United Kingdom (Clitellata: Hirudinida: Piscicolidae). Comparative Parasitology 79(2): 219–230; Brusca, RC, Moore, W & Shuster, SM. (eds) (2016) Chapter 14. Phylum Annelida. In: Invertebrates, 3rd Edition. Sinauer Associates, Sunderland, MA; Thorp JH, Lovell LL, Timm T, Martin P, Gelder SR, Govedich FR, Moser WE, Nakano T, Bielecki A, Bain BA, Utevsky S, Gil J, Glasby CJ, Martin D (2019) Phylum Annelida. In: Rogers DC, Thorp JH (Eds) Keys to Palaearctic Fauna: Thorp and Covich’s Freshwater invertebrates - Volume IV. Academic Press, Elsevier, pp. 357–518; Burreson EM (2020) Marine and estuarine leeches (Hirudinida : Ozobranchidae and Piscicolidae) of Australia and New Zealand with a key to the species. Invertebrate Systematics 34: 235–259.

# Poecilochaetidae Hannerz, 1956

**Description**. Body segment number: variable. Body shape elongate, more-or-less equal width over entire length. Epidermis more-or-less smooth. Pygidium simple lobe. Pygidial appendages present, three cirri or four cirri. Discrete head present; complex in shape bearing appendages.

Prostomium rounded to oval, facial tubercle present, eyes on head present, two pairs; head eyes simple pigmented cups. Prostomial antennae present; median one only; unarticulated. Palps present; anterodorsal; grooved (usually) feeding type; feeding palp longitudinally grooved. Caruncle absent, or present. Nuchal organs present; projecting considerably from prostomium (three posteriorly projecting digitiform lobes). Peristomium visible. Peristomium a single ring, reduced dorsally.

Foregut a muscular ventral pharynx. Pharynx dorsolateral ciliated folds present.

First segment chaetous and tentaculate (two enlarged neuropodial lobes that resemble tentacles); second segment chaetous. First chaetiger with both notochaetae and neurochaetae; parapodia very elongated; parapodia anteriorly directed and wrapping around head; chaetae slender and elongate, forming cage (or basket) around head (=cephalic cage chaetae). Parapodia biramous; parapodial lobes prominent. Notopodial lobes slender, flask- or spindle-shaped (rarely cirriform). Neuropodial lobes slender, flask- or spindle-shaped (rarely cirriform). Lateral organs absent. Branchiae present (rarely absent), arise from lateral body (posterior face), occur on mid-body segments or occur near posterior end; lateral branchiae digitiform, or branching.

Chaetae first appear on first segment after peristomium, arranged in paired bundles (or rows) of many chaetae. Aciculae absent. Capillary chaetae present (many different kinds), in dorsal (notopodial) position or in ventral (neuropodial) position, distally tapered to a point, edge smooth or corn-eared or hirsute-serrate. Capillary chaetae, externally not pseudo-segmented. Capillary chaetae, internally not chambered or hollow. Spines present (many different kinds); slightly curved and more-or-less smooth, or with small teeth or spinelets, or with a single distal or subdistal hair (=aristate or hooded), or sharply bent (=geniculate) or recurved; present in most or all chaetigers in both dorsal and ventral positions (posterior spines in notopodial position). Hooks absent.

Segmental organs are metanephridia; metanephridia along most of the body; anterior ones for excretion, posterior ones for gamete release.

Tube present; membranous (U-shaped or branching).

**Literature used for description**. Glasby CJ, Fauchald K (2003). POLiKEY. An information system for polychaete families and higher taxa. Version 2, 5 June 2003. Australian Biological Resources Study, DCCEEW, Canberra, https:[//www.dcceew.gov.au;](http://www.dcceew.gov.au/) Blake JA, Maciolek NJ (2019)

7.4.2 Poecilochaetidae Hennerz, 1956 In: Purschke G, Böggemann M, Westheide W (Eds), Handbook of Zoology, Annelida, Volume 2: Pleistoannelida, Sedentaria II. De Gruyter, Berlin, 103–119; Rouse GW, Pleijel F, Tilic E (2022) Annelida. Oxford University Press, Oxford, UK.

# Polygordiidae Czerniavsky, 1881

**Description**. Body segment number: variable, segmentation present (but segments poorly delineated externally (parapodia and chaetae absent) so body appears smooth; segments only inferred through presence of internal septa which can be seen through body wall). Body segments similar dimensions throughout. Body shape elongate, more-or-less equal width over entire length. Body translucent, gut visible; epidermis more-or-less smooth (iridescent cuticle). Ventral groove present (shallow; also may have a pair of ventrolateral grooves). Pygidium simple lobe (may be inflated and be adorned with a ring of subterminal papilla-sized adhesive glands). Pygidial appendages absent or present, one pair of cirri or more than four cirri (up to 15, attached terminally or subterminally). Discrete head present; complex in shape bearing appendages.

Prostomium bluntly conical (posterior head fold). Postcephalic eyes present; epidermal. Prostomial antennae absent (the sensory palps resemble long antennae). Palps present; frontal; tapering (usually) sensory type; unarticulated. Nuchal organs present; paired low projections from posterolateral prostomium; indistinct dorsolateral ciliated patches. Peristomium a single ring, separated from prostomium by a head fold.

Foregut a muscular ventral pharynx (as per Worsaae 2021 who described it as a ventral pharyngeal sac). Protrusible proboscis present. Pharynx dorsolateral ciliated folds present. Parapodia absent. Chaetae absent.

Sexual reproduction gonochoric. Segmental organs are protonephridia. Tube absent.

**Literature used for description**. Glasby CJ, Fauchald K (2003). POLiKEY. An information system for polychaete families and higher taxa. Version 2, 5 June 2003. Australian Biological Resources Study, DCCEEW, Canberra, https:[//www.dcceew.gov.au/;](http://www.dcceew.gov.au/%3B) Worsaae, K, Kerbl, A., Di Domenico, M., Gonzalez, B.C., Bekkouche, N. & Martinez, A. (2021). Interstitial Annelida. Diversity 2021, 13, 77. https://doi.org/10.3390/d13020077; Ramey-Balci PA, Fiege D, Purschke G (2021) 7.11.1 Polygordiidae Czerniavsky, 1881 In: Purschke G, Böggemann M, Westheide W (Eds), Handbook of Zoology, Annelida, Volume 3: Pleistoannelida, Sedentaria III and Errantia I. De Gruyter, Berlin, 266–279; Rouse GW, Pleijel, F, Tilic E (2022) Annelida. Oxford University Press, Oxford, UK.

# Polynoidae Kinberg, 1856

**Description**. Body segment number: fixed or variable, less than 14 segments (rarely). Body shape elongate, more-or-less equal width over entire length or ovate to elliptical, dorsoventrally flattened. Body pigmentation absent (rarely) or present, highly variable pigmentation patterns; often brown or orange elytra with a darker central spot; epidermis more-or-less smooth (rarely papillate ventrally). Bioluminescence absent, or present. Body surface with protective covering as scales (elytrae), dorsally. Pygidium simple lobe. Pygidial appendages present, one pair of cirri. Discrete head present; complex in shape bearing appendages.

Prostomium rounded to oval, anteriorly incised (often with peaks anterolaterally), facial tubercle absent or present, eyes on head present, two pairs; head eyes compound, with lenses. Prostomial antennae present (rarely absent); median and paired laterals (rarely median and, or, lateral antennae absent - mostly in deep-sea species); consist of basal ceratophore and distal ceratostyle. Palps present; anteroventral; tapering (usually) sensory type; unarticulated. Nuchal organs present; paired low projections from posterolateral prostomium; indistinct dorsolateral ciliated patches. Peristomium not visible.

Gut straight with side branches. Foregut a muscular axial pharynx. Pharynx jaws present, two pairs of jaws. Paired jaws fang-like. Protrusible proboscis present, distal ring of papillae present.

First segment chaetous and tentaculate (dorsal and ventral cirri enlarged and elongated); second segment chaetous. Tentacular cirri present; 2–3 pair(s); arise on a single segment. First chaetiger without external chaetae, or with notochaetae only; parapodia similar in length or slightly shorter than subsequent parapodia; parapodia anteriorly directed and wrapping around head; chaetae similar in orientation, length and thickness to other chaetae. Parapodia biramous (usually); parapodial lobes prominent. Notopodial lobes represented by at least one chaetal lobe (poorly developed by comparison to neuropodia). Neuropodial lobes represented by at least one chaetal lobe. Dorsal cirri present (present on non-elytrous segments); more-or-less cirriform. Elytra with papillae, tubercles or smooth. Elytra, post segment 7 occur on every other segment from segment 7 to midbody, then present on every segment, or absent, to end of body. Ventral cirri present; cirriform or tapering. Branchiae absent or present (rarely), arise from lateral body, occur on mid-body segments; lateral branchiae digitiform.

Chaetae arranged in paired bundles (or rows) of many chaetae. Aciculae present; in both dorsal and ventral positions (=noto- and neuroaciculae). Neuroaciculae distally tapering. Capillary chaetae present (though thick and almost spine-like), in dorsal (notopodial) position or in ventral (neuropodial) position, distally tapered to a point, edge spinose or corn-eared. Capillary chaetae, externally not pseudo-segmented. Capillary chaetae, internally not chambered or hollow. Spines present; with small teeth or spinelets, or slightly curved and more-or-less smooth; present in most or all chaetigers; in both dorsal and ventral positions. Hooks absent.

Segmental organs are metanephridia; metanephridia along most of the body; anterior ones for excretion, posterior ones for gamete release;

Tube absent.

**Literature used for description**. Glasby CJ, Fauchald K (2003). POLiKEY. An information system for polychaete families and higher taxa. Version 2, 5 June 2003. Australian Biological Resources Study, DCCEEW, Canberra, https:[//www.dcceew.gov.au/;](http://www.dcceew.gov.au/%3B) Hourdez S (2022) 7.13.1.4. Polynoidae Kinberg, 1856, In: Purschke G, Böggemann M, Westheide W (Eds), Handbook of Zoology, Annelida, Volume 3: Pleistoannelida, Errantia II. De Gruyter, Berlin, 93–113; Rouse GW, Pleijel, F, Tilic E (2022) Annelida. Oxford University Press, Oxford, UK.

# Pontodoridae Bergström, 1914

**Description**. Body segment number: variable. Body shape elongate, more-or-less equal width over entire length. Body translucent, gut visible. Pygidium simple lobe. Pygidial appendages present, one pair of cirri. Discrete head present; complex in shape bearing appendages.

Prostomium rounded to oval, eyes on head present, one pair; head eyes simple pigmented cups. Prostomial antennae present; paired, lateral; unarticulated. Palps present; anteroventral; tapering (usually) sensory type; unarticulated. Nuchal organs present; paired low projections from posterolateral prostomium; indistinct dorsolateral ciliated patches. Peristomium not visible.

Foregut a muscular axial pharynx. Protrusible proboscis present, with papillae in subterminal position (very long), irregularly arranged, distal ring of papillae present. Pharynx proventricle present (?).

First segment tentaculate; second segment chaetous and tentaculate. Tentacular cirri present; 2 pair(s); arise on a single segment; internal aciculae absent. First chaetiger with neurochaetae only; parapodia similar in length or slightly shorter than subsequent parapodia; parapodia more-or-less laterally directed and free from head; chaetae similar in orientation, length and thickness to other chaetae. Parapodia uniramous. Notopodial lobes absent (though dorsal cirri are present). Neuropodial lobes represented by at least one chaetal lobe. Dorsal cirri present; more-or-less cirriform. Ventral cirri present; cirriform or tapering.

Arranged as a single bundle (or row) of many chaetae (lower bundle - neurochaetae - only). Aciculae present; in ventral position (=neuroaciculae). Neuroaciculae distally tapering. Capillary chaetae absent. Hooks absent. Compound chaetae present; in ventral (neuropodial) position; appendage distally tapering to slender tips (=spinigerous); appendage not canaliculated; appendage without hoods or guards; joint, appearance distinctly asymmetrical (=heterogomph); joint effected by ligament(s); shaft tapering slightly or evenly thick from emergence to joint; shaft, internally solid, without distinct core. Compound chaetae present in most or all chaetigers.

Tube absent.

**Literature used for description**. Rouse GW, Pleijel, F, Tilic E (2022) Annelida. Oxford University Press, Oxford, UK.

# Praobdellidae Sawyer, 1986

**Description**. Body segment number: fixed, 34 segments including 2 preoral 'segments' (prostomium and peristomium) and 32 postoral segments (true segments generally obscured by intervening superficial annulations, giving the impression of many more segments). Secondary annulation present (disk-shaped receptors called sensillae mark the middle annulus of each segment); mid-body 5-annulate. Body shape elongate, more-or-less equal width over entire length (when relaxed; but can be dramatically shortened by contraction of longitudinal muscles during movement), dorsoventrally flattened. Anterior end sucker on ventral surface present (surrounding mouth; sulcus on its inner surface); not clearly separated from rest of body. Large mouth on ventral surface of sucker. Body pigmentation absent or present, dorsal surface may be green and lateral margins may be orange coloured (*Limnatis* and *Limnobdella* species); epidermis papillate (weakly). Posterior sucker on ventral surface present; anus positioned near posterior end (dorsally, just before posterior sucker).

Eyes on head present, five pairs (usually; arranged in two separate groups); head eyes simple pigmented cups.

Gut straight with side branches. Foregut a muscular axial pharynx; muscular axial pharynx not rotated (euthylaematous). Pharynx jaws present, leech-type (either soft-muscular or horny) (as soft muscular 'jaws'); two (duognathous) muscular jaws, or three (trignathous) muscular jaws arranged in a triangle; denticles present, two rows of teeth or as cutting plates or paired, fine teeth or series of soft teeth. Protrusible proboscis absent. Caeca of midgut (=posterior crop caeca) present (11 pairs). Caeca of hindgut present (one pair, longer than more anterior ones). Circulatory system absent.

Sexual reproduction hermaphroditic. Clitellum fully encircles body, in region of male and female pores. Male and female pores separated by 5 annuli. Clitellum occurs from X-XIII (usual position for leeches). Testes present, many pairs, one pair per segment. Ovaries present, one pair. Egg sacs present. Segmental organs are metanephridia; nephridial pores single (ventromedial). Female gonoduct present. Female pores single, median, in segment XII. Male gonoducts present. Male pores single, median, in segment XI. Prostate gland absent.

**Literature used for description**. Siddall ME, Bely AE, Borda E (2006) Hirudinida Pp. 393–429. In Reproductive Biology and Phylogeny of Annelida. Series Editor BGM Jamieson. Volume 4. Editors G Rouse, F Pleijel. Science Publishers, Enfield, New Hampshire; Brusca, RC, Moore, W & Shuster, SM. (eds) (2016) Chapter 14. Phylum Annelida. In: Invertebrates, 3rd Edition. Sinauer Associates, Sunderland, MA; Nakano, T, Tomikawa, K, Sakono T, Yoshikawa N (2017) Praobdellidae (Hirudinida: Arhynchobdellida) is not specific only to the mucous-membrane after all: Discovery of a praobdellid leech feeding on the Japanese freshwater crab *Geothelphusa dehaani.* Parasitology International 66: 210–213; Thorp JH, Lovell LL, Timm T, Martin P, Gelder SR, Govedich FR, Moser WE, Nakano T, Bielecki A, Bain BA, Utevsky S, Gil J, Glasby CJ, Martin D (2019) Phylum Annelida. In: Rogers DC, Thorp JH (Eds) Keys to Palaearctic Fauna: Thorp and Covich’s Freshwater invertebrates - Volume IV. Academic Press, Elsevier, pp. 357–518; Utevsky S, Mabrouki Y, Taybi AF, Huseynov M, Manafov A, Morhun H, Shahina O, Utevsky G, Khomenko A, Utevsky A (2022) New records of leeches of the genus *Limnatis* (Hirudinea, Praobdellidae) from the South Caucasus and Central Asia: phylogenetic relationships of Eurasian and African populations. Animal Biodiversity and Conservation 45: 43–52.

# Propappidae Coates, 1986

**Description**. Body segment number: variable. Secondary annulation absent or present. Body shape elongate, more-or-less equal width over entire length (small worms). Pygidium simple lobe. Pygidial appendages absent.

Prostomium bluntly conical with an anterior tentacle-like projection (='proboscis'; when present, short; absent in *Propappus glandulosus*).

Foregut a muscular dorsal pharynx.

Chaetae present, first appear on first segment after peristomium (both dorsal and ventral bundles), in lumbricine arrangement - paired upper and lower bundles of mostly 3 per bundle, widely spaced lateral and ventrolateral pairs. Capillary (=hair) chaetae absent. Crotchet chaetae present, bifid.

Sexual reproduction hermaphroditic. Clitellum thin, single layered, in region of male and female pores. Clitellum occurs from XII for a few segments. Testes present, one pair in total; present in segment XI. Ovaries present, one pair; present in segment XIII. Segmental organs are metanephridia; both nephridial pores and gonoducts located around clitellum. Spermathecae present, pre-testicular. Spermathecal pores present, located well anterior to male pores ; 1 pair; in segment III-IV, in intersegmental groove (IV-V according to Timm 2009). Female gonoduct present. Female pores one pair, in segment XIV. Male gonoducts present. Male pores one pair, in segment XII, plesioporous (in segment following testicular segment). Prostate gland absent.

**Literature used for description**. Jamieson BGM (2006) Non-leech Clitellata (with contributions by Marco Ferraguti). Pp. 235–392. In Reproductive Biology and Phylogeny of Annelida. Series Editor BGM Jamieson. Volume 4. Editors G Rouse, F Pleijel. Science Publishers, Enfield, New Hampshire; Martin, P, Martinez-Ansemil, E., Pinder, A, Timm, T, Wetzel, MJ (2008) Global diversity of oligochaetous clitellates (‘‘Oligochaeta’’; Clitellata) in freshwater. Hydrobiologia (2008) 595:117–127; Timm, T (2009) A guide to the freshwater Oligochaeta and Polychaeta of Northern and Central Europe. Lauterbornia 66: 1–235.

# Protodrilidae Hatschek, 1888

**Description**. Body segment number: variable. Body segments similar dimensions throughout. Body shape elongate, more-or-less equal width over entire length. Body translucent, gut visible, pigmentation absent or present, pigmentation pharynx may be pigmented; epidermis more-or-less smooth. Dorsal body surface, appearance under compound microscope ciliated (midventral ciliary band). Ventral groove present (ciliated). Pygidium bilobed (pygidial lobes with adhesive glands). Discrete head present; complex in shape bearing appendages (also bearing cilia; stiff or flexible; called 'anterior sensory cilia' in Annelida Glossary, [doi.org/10.5281/zenodo.14848165](https://doi.org/10.5281/zenodo.14848165)).

Prostomium bluntly conical, eyes on head absent or present, one pair; head eyes simple pigmented cups. Eyes on trunk absent, or present. Trunk eyes on dorsal surface, or on ventral surface. Palps present; anteroventral (bearing single internal canals); tapering (usually) sensory type; unarticulated. Nuchal organs present; paired low projections from posterolateral prostomium; indistinct dorsolateral ciliated patches. Peristomium a single ring.

Foregut a muscular ventral pharynx. Protrusible proboscis present. Pharynx dorsolateral ciliated folds present.

First segment achaetous; second segment achaetous. Parapodia absent. Chaetae absent.

Sexual reproduction gonochoric. Testes present. Segmental organs are protonephridia. Penis absent.

Tube absent.

**Literature used for description**. Glasby CJ, Fauchald K (2003). POLiKEY. An information system for polychaete families and higher taxa. Version 2, 5 June 2003. Australian Biological Resources Study, DCCEEW, Canberra, https:[//www.dcceew.gov.au/;](http://www.dcceew.gov.au/%3B) Worsaae, K, Kerbl, A., Di Domenico, M., Gonzalez, B.C., Bekkouche, N. & Martinez, A. (2021). Interstitial Annelida. Diversity 2021, 13, 77. https://doi.org/10.3390/d13020077; Martinez A, Purschke G, Worsaae K (2021) 7.11.3 Protodrilidae Hatschek, 1888 In: Purschke G, Böggemann M, Westheide W (Eds), Handbook of Zoology, Annelida, Volume 3: Pleistoannelida, Sedentaria III and Errantia I. De Gruyter, Berlin, 299–337; Rouse GW, Pleijel, F, Tilic E (2022) Annelida. Oxford University Press, Oxford, UK.

# Protodriloididae Purschke & Jouin, 1988

**Description**. Body segment number: variable (segments indistinct). Body segments similar dimensions throughout. Body shape elongate, more-or-less equal width over entire length. Body translucent, gut visible, pigmentation absent or present, pigmentation yellow, orange or green spots (epidermal glands or gut pigmentation). Dorsal body surface, appearance under compound microscope ciliated (dorsal and ventral ciliary bands). Ventral groove present (ciliated). Pygidium bilobed. Discrete head present; complex in shape bearing appendages.

Prostomium bluntly conical. Palps present (tentacle-like anterior projections of prostomium); frontal (bearing single internal canals); tapering (usually) sensory type; unarticulated. Nuchal organs present; paired low projections from posterolateral prostomium; indistinct dorsolateral ciliated patches. Peristomium a single ring.

Foregut a muscular ventral pharynx. Protrusible proboscis present. Pharynx dorsolateral ciliated folds present.

First segment achaetous; second segment achaetous. First chaetiger with neurochaetae only; parapodia similar in length or slightly shorter than subsequent parapodia; parapodia more-or-less laterally directed and free from head; chaetae similar in orientation, length and thickness to other chaetae. Parapodia absent.

Chaetae absent or present, first appear on second segment after peristomium, in lumbricine arrangement - paired upper and lower bundles of one or a few chaetae each (1 per 'bundle'). Capillary chaetae absent. Crotchet chaetae present, bifid. Comb-like chaetae absent. Hooks absent.

Sexual reproduction gonochoric. Segmental organs are protonephridia. Penis absent. Tube absent.

**Literature used for description**. Glasby CJ, Fauchald K (2003). POLiKEY. An information system for polychaete families and higher taxa. Version 2, 5 June 2003. Australian Biological Resources Study, DCCEEW, Canberra, https:[//www.dcceew.gov.au/;](http://www.dcceew.gov.au/%3B) Worsaae, K, Kerbl, A., Di Domenico, M., Gonzalez, B.C., Bekkouche, N. & Martinez, A. (2021) Interstitial Annelida. Diversity 13: 77. https://doi.org/10.3390/d13020077; Martinez A, Worsaae K, Purschke G (2021) 7.11.4 Protodriloididae Purschke & Jouin,1988 In: Purschke G, Böggemann M, Westheide W (Eds), Handbook of Zoology, Annelida, Volume 3: Pleistoannelida, Sedentaria III and Errantia I. De Gruyter, Berlin, 338–353; Rouse GW, Pleijel, F, Tilic E (2022) Annelida. Oxford University Press, Oxford, UK.

# Psammodrilidae Swedmark, 1952

**Description**. Body segment number: variable. Body segments similar dimensions throughout. Body shape elongate, more-or-less equal width over entire length, regionalization present, regionalization comprising two regions, regions demarcated by structural differences in parapodia over body (thorax region of about 6 segments with cirri but no external chaetae; abdomen region of 7–31 chaetigers with uncini). Body translucent, gut visible, pigmentation absent. Dorsal body surface, appearance under compound microscope ciliated (almost entirely). Pygidium simple lobe. Pygidial appendages absent (except for sensory cilia of two different lengths). Discrete head present; lobe-like without appendages (bearing cilia; stiff or flexible; called 'anterior sensory cilia' in Annelida Glossary, [doi.org/10.5281/zenodo.14848165](https://doi.org/10.5281/zenodo.14848165)).

Prostomium rounded to oval, eyes on head absent or present, two pairs; head eyes simple pigmented cups. Nuchal organs absent. Peristomium a double ring (muscular collar region with warty appearance).

Foregut without a distinct ventral or axial pharynx (although a proventricle-type organ maybe present). Pharynx dorsolateral ciliated folds present.

First segment chaetous; second segment chaetous. First chaetiger with notochaetae only; parapodia similar in length or slightly shorter than subsequent parapodia; parapodia more-or-less laterally directed and free from head; chaetae similar in orientation, length and thickness to other chaetae. Parapodia present. Parapodia uniramous. Notopodial lobes represented by at least one chaetal lobe (notopodia only on thoracic segments). Neuropodial lobes low ridges (tori) (neuropodia only on abdomen).

Chaetae first appear on first segment after peristomium, arranged as a single bundle (or row) of many chaetae (notoaciculae only in thorax; neurochaetae in abdomen). Aciculae present; in dorsal position (=notoaciculae) (support thoracic cirri). Capillary chaetae absent. Hooks present (uncini-like); without distal hood, beard or ligament; occur in ventral (neuropodial) position; occur in posterior body only.

Segmental organs are metanephridia; metanephridia several pairs metanephridia anteriorly for excretion, posterior ones for gamete release (4–7 pairs).

# Tube absent, or present.

**Literature used for description**. Glasby CJ, Fauchald K (2003). POLiKEY. An information system for polychaete families and higher taxa. Version 2, 5 June 2003. Australian Biological Resources Study, DCCEEW, Canberra, https:[//www.dcceew.gov.au/;](http://www.dcceew.gov.au/%3B) Worsaae K, Kerbl A, Di Domenico M, Gonzalez BC, Bekkouche N, Martinez A (2021). Interstitial Annelida. Diversity 2021, 13, 77. https://doi.org/10.3390/d13020077; Worsaae K (2019) 5.2 Psammodrilidae Swedmark, 1952 In Purschke G, Böggemann M, Westheide W (Eds), Handbook of Zoology, Annelida, Volume 1: Annelida Basal Groups and Pleistoannelida, Sedentaria I. De Gruyter, Berlin, 143–155; Rouse GW, Pleijel, F, Tilic E (2022) Annelida. Oxford University Press, Oxford, UK: Rouse GW, Pleijel, F, Tilic E (2022) Annelida. Oxford University Press, Oxford, UK.

# Randiellidae Erséus & Strehlow, 1986

**Description**. Body segment number: variable. Secondary annulation absent or present. Body shape elongate, more-or-less equal width over entire length. Pygidium simple lobe. Pygidial appendages absent.

Prostomium bluntly conical, zygolobic (prostomium not demarcated).

Foregut a muscular dorsal pharynx.

Chaetae first appear on first segment after peristomium (both dorsal and ventral bundles; =S2 for oligochaete workers), in lumbricine arrangement - paired upper and lower bundles of one or a few chaetae each (in anterior segments often more than 2 per bundle) - closely spaced lateral and ventrolateral pairs. Capillary (=hair) chaetae absent. Crotchet chaetae present, simple. Genital chaetae present (long fine spine, hair-like, with hollow tip; segment X).

Sexual reproduction hermaphroditic. Clitellum thin, single layered, posterior to male pores, in region of female pores. Clitellum occurs from XII-XIII. Testes present, one pair in total or two pairs in total; present in segments X-XIII, usually X. Ovaries present, one pair or two pairs; present in segments X-XIII, usually XII. Segmental organs are metanephridia; both nephridial pores and gonoducts located around clitellum. Spermathecae present, pre-testicular (all in one segment). Spermathecal pores present, located well anterior to male pores; 1–2 pairs; in segment VII and/or VIII. Female gonoduct absent. Male gonoducts present, with atrium absent. Male pores one pair or two pairs, in segment XI, plesioporous (in segment following testicular segment) or prosoporous (same segment as corresponding testes) (uncertain). Prostate gland absent.

**Literature used for description**. Erséus C, Strehlow DR (1986) Four new interstitial species of marine Oligochaeta representing a new family. Zoologica Scripta 15: 53–60; Erséus C (1997) A record of Randiella from New Caledonia, the first known occurrence of the marine interstitial family Randiellidae (Annelida; Oligochaeta) in the South Pacific Ocean. Journal of Natural History 31: 1745–1750; Jamieson BGM (2006) Non-leech Clitellata (with contributions by Marco Ferraguti). Pp. 235–392. In Reproductive Biology and Phylogeny of Annelida. Series Editor BGM Jamieson. Volume 4. Editors G Rouse, F Pleijel. Science Publishers, Enfield, New Hampshire; Timm T (2012) Life forms in Oligochaeta: a literature review. Zoology in the Middle East, Supplement 4, 58: 71–82.

# Rhinodrilidae Benham, 1890

**Description**. Body segment number: variable. Body shape elongate, more-or-less equal width over entire length. Dorsal intersegmental furrow present, pores on mid-dorsal line absent (scored from James 2012). Pygidium simple lobe. Pygidial appendages absent.

Prostomium bluntly conical. Foregut a muscular dorsal pharynx. Gizzard present, one pair in segment VI. Calciferous glands present (three pairs of extra-mural calciferous glands), in segment VII–XIV. Intestinal typhlosole present. Heart bodies present, segments X and XI.

Chaetae first appear on second segment after peristomium (=S3 for oligochaete workers), in lumbricine arrangement - paired upper and lower bundles of one or a few chaetae each (closely spaced lateral and ventrolateral pairs anteriorly) or in perichaetine arrangement - chaetae more-or-less evenly distributed around perimeter of segment (more than 8 chaetae per segment) (posteriorly). Crotchet chaetae present, simple-pointed. Genital chaetae present (on segments XVIII-XXIII).

Sexual reproduction hermaphroditic. Clitellum partially encircles body, in region of male pores. Clitellum occurs from XIV-XXII. Tubercula pubertatis present, paired ridges on ventrolateral margins of clitellum. Testes present, two pairs in total; present in segments X and XI. Sperm sac absent. Ovaries present; present in segment XIII. Segmental organs are metanephridia; both nephridial pores and gonoducts located around clitellum, one pair nephridia in each segment (holonephridia). Spermathecae present, pre-testicular. Spermathecal pores present, located well anterior to male pores; 3 pairs; in segment VI/VII to VIII/IX; intersegmental. Female gonoduct present. Female pores one pair, in segment XIV. Male gonoducts present (male pores usually very small, behind female pores; if macroscopic, then with copulatory chambers). Male pores one pair or two pairs, in segment XI and XII, plesioporous (in segment following testicular segment). Prostate gland present (?or similar structures).

**Literature used for description**. James SW, Davidson SK (2012) Molecular phylogeny of earthworms (Annelida : Crassiclitellata) based on 28S, 18S and 16S gene sequences. Invertebrate Systematics 26: 213–229; James SW (2012) Re-erection of Rhinodrilidae Benham, 1890, a senior synonym of Pontoscolecidae James, 2012 (Annelida: Clitellata). Zootaxa 3540: 67–68. Hernandez-Garcia, LM, Costa Sousa S, Jovita Pereira N, Rousseau GX, Ferreira C (2023) Additions to earthworms (Annelida, Crassiclitellata) from Aragua and Miranda states, Venezuela. Zootaxa 5255: 171–182; Misirlioðlu, M, Reynolds, JW, Stojanoviæ, M, Trakiæ, T, Sekuliæ, J, James, SW, Csuzdi, C, Decaëns, T, Lapied, E, Phillips, HRP, Cameron, EK, Brown, GG (2023). Earthworms (Clitellata, Megadrili) of the world: an updated checklist of valid species and families, with notes on their distribution. Zootaxa 5255 (1): 417–438.

# Sabellariidae Johnston, 1865

**Description**. Body segment number: variable. Body shape widest anteriorly and tapering posteriorly, regionalization present, regionalization comprising three regions (four regions if the thorax is considered two), regions demarcated by structural differences in parapodia over body and inversion of parapodia (a thorax-parathorax region comprising 4–5 chaetigers; an abdomen with many chaetigers segments; and a posterior cauda of many very short achaetigerous segments). Body pigmentation present, dark red or brown dorsally; epidermis more-or-less smooth. Caudal region an unsegmented tube. Pygidium simple lobe. Pygidial appendages absent. Discrete head present (head is a difficult-to-see lobe in front of the mouth which is surrounded by a massive operculum comprising modified anterior segments); lobe-like without appendages, or complex in shape bearing appendages (may bear a small cirrus).

Prostomium narrow, keel- or ridge-shaped (also called a median organ), with an anterior tentacle-like projection (='palpode'), eyes on head absent or present; head eyes simple pigmented cups. Caudal eyes absent, or present. Buccal tentacles present (also called ‘oral filaments’; rarely absent); lining inner margin of opercular lobes; smooth. Palps present; anteroventral (just in front of the mouth); grooved (usually) feeding type; feeding palp longitudinally grooved. Nuchal organs present; paired low projections from posterolateral prostomium; indistinct dorsolateral ciliated patches, or patches adjacent mid-dorsal hump. Peristomium not visible.

Gut more-or-less straight, lacking side branches. Foregut without a distinct ventral or axial pharynx. Heart bodies present. First segment chaetous (though highly modified forming an operculum); second segment chaetous (also forming the operculum). First chaetiger with both notochaetae and neurochaetae (highly modified into an operculum); parapodia very elongated; parapodia anteriorly directed and wrapping around head; chaetae projecting obliquely, distinctly thicker and more shiny than subsequent ones (=paleae). Parapodia biramous; parapodial lobes prominent. Notopodial lobes represented by at least one chaetal lobe (anteriorly), or low lateral ridges (tori) (posteriorly). Neuropodial lobes low ridges (tori) (anteriorly), or represented by at least one chaetal lobe (posteriorly). Branchiae present, arise from dorsal body, occur on mid-body segments (occur over limited region of parathorax and abdomen). Dorsal branchiae simple filaments each arising directly from body wall.

Chaetae first appear on first segment after peristomium, arranged in paired bundles (or rows) of many chaetae. Aciculae absent. Capillary chaetae present, in dorsal (notopodial) position or in ventral (neuropodial) position, distally tapered to a point or expanded, edge smooth. Capillary chaetae, externally not pseudo-segmented. Capillary chaetae, internally not chambered or hollow. Paleate chaetae present (called 'cephalic paleae'), associated with head (derived from segments 1 and 2). Spines present; slightly curved and more-or-less smooth; present in most or all chaetigers (first pair are often large and hook shaped); in dorsal (notopodial) position only. Hooks absent. Uncini present; with teeth in vertical series, teeth usually similar-sized (=pectinate); in ventral (neuropodial) position on thorax and dorsal (notopodial) position on abdomen; arranged in one row.

Reproduction sexual.

Segmental organs are metanephridia; metanephridia single anterior pair of excretory metanephridia and several more posterior ones for gamete release (excretory ones on segment 1); nephridial pores located anteriorly.

Tube present; cemented sand grains (often reef-forming, although some species are solitary); entrance able to be plugged by worm.

**Literature used for description**. Glasby CJ, Fauchald K (2003). POLiKEY. An information system for polychaete families and higher taxa. Version 2, 5 June 2003. Australian Biological Resources Study, DCCEEW, Canberra, https:[//www.dcceew.gov.au/;](http://www.dcceew.gov.au/%3B) Capa M, Hutchings P (2019) 7.4.5 Sabellariidae Johnston, 1865 In: Purschke G, Böggemann M, Westheide W (Eds), Handbook of Zoology, Annelida, Volume 2: Pleistoannelida, Sedentaria II. De Gruyter, Berlin, 144–163; Rouse GW, Pleijel F, Tilic E (2022) Annelida. Oxford University Press, Oxford, UK.

# Sabellidae Latreille, 1825

**Description**. Body segment number: variable. Body shape elongate, more-or-less equal width over entire length, regionalization present (thoracic uncini in neuropodial position, abdominal uncini notopodial), regionalization comprising two regions (comprising 4–12 thoracic segments (typically) and 3 to over 100 abdominal segments), regions demarcated by inversion of parapodia (and shift in position of faecal groove from dorsal (thoracic) to ventral (abdominal)). Body pigmentation present, radiolar crowns often brightly coloured; body may be speckled with darker pigment; epidermis more-or-less smooth. Thoracic membranes absent, or present. Faecal groove present. Pygidium simple lobe. Pygidial appendages absent. Discrete head absent (as a radiolar crown); radiolar crown present (3 to many pairs of radioles (basally fused dorsally or unfused); paired ventral sacs and parallel lamellae may be present with radioles); internal 'skeleton' present; modified radioles absent. Eyes on head present, numerous, unpaired (on peristomium or radiolar crown); head eyes simple pigmented cups (on peristomium), or compound, with lenses (radiolar crown). Caudal eyes absent, or present. Nuchal organs present (pair of internalized pouches in a dorsal pit above mouth). Peristomium a single ring, collar-like (collar region referred to as ventral lappets).

Foregut without a distinct ventral or axial pharynx. Protrusible proboscis absent. Heart bodies absent.

First segment chaetous. First chaetiger with notochaetae only (called collar chaetae); parapodia similar in length or slightly shorter than subsequent parapodia; parapodia more-or-less laterally directed and free from head; chaetae similar in orientation, length and thickness to other chaetae. Parapodia biramous; parapodial lobes prominent. Notopodial lobes represented by at least one chaetal lobe (anteriorly), or low lateral ridges (tori) (posteriorly). Neuropodial lobes low ridges (tori) (anteriorly), or represented by at least one chaetal lobe (posteriorly).

Chaetae first appear on first segment after peristomium, arranged in paired bundles (or rows) of many chaetae. Aciculae absent. Capillary chaetae present, in dorsal (notopodial) position or in ventral (neuropodial) position, distally tapered to a point or expanded or with subdistal spur, edge smooth or spinose. Capillary chaetae, externally not pseudo-segmented. Capillary chaetae, internally not chambered or hollow. Hooks absent. Uncini present (usually avicular type; i.e., usually short shafts; long shafts when present resemble hooks); with teeth in vertical series, teeth usually similar-sized (=pectinate), or with teeth arranged in transverse series above an enlarged main fang (=crested); in ventral (neuropodial) position on thorax and dorsal (notopodial) position on abdomen; arranged in one row.

Reproduction asexual, or sexual. Asexual reproduction by paratomy.

Sexual reproduction hermaphroditic, or gonochoric. Segmental organs are metanephridia; metanephridia single anterior pair of excretory metanephridia and several more posterior ones for gamete release (excretory ones on segment 1); nephridial pores located anteriorly.

Tube absent (rarely), or present; membranous, or leathery or parchment like.

**Literature used for description**. Fitzhugh K (1989). A systematic revision of the Sabellidae-Caobangidae-Sabellongidae complex (Annelida: Polychaeta). Bulletin of the American Museum of Natural History. 192: 1–104., available online at [http://hdl.handle.net/2246/881;](http://hdl.handle.net/2246/881%3B) Glasby CJ, Fauchald K (**2003**). POLiKEY. An information system for polychaete families and higher taxa. Version 2, 5 June 2003. Australian Biological Resources Study, DCCEEW, Canberra, https:[//www.dcceew.gov.au/;](http://www.dcceew.gov.au/%3B) Capa M, Giangrande A, Nogueira JM de M, Tover-Hernandez MA (2019) 7.4.6 Sabellidae Latreille, 1825 In: Purschke G, Böggemann M, Westheide W (Eds), Handbook of Zoology, Annelida, Volume 2: Pleistoannelida, Sedentaria II. De Gruyter, Berlin, 164–212; Capa M, Kupriyanova E, Nogueira JMdM, Bick A, Tovar-Hernández MA (2021) Fanworms: Yesterday, Today and Tomorrow. Diversity 13: 130, https://doi.org/10.3390/d13030130; Rouse GW, Pleijel F, Tilic E (2022) Annelida. Oxford University Press, Oxford, UK.

# Saccocirridae Czerniavsky, 1881

**Description**. Body segment number: variable. Body segments similar dimensions throughout. Body shape elongate, more-or-less equal width over entire length. Body translucent, gut visible, pigmentation absent or present, pigmentation yellow, orange or green spots (epidermal glands or gut pigmentation). Dorsal body surface, appearance under compound microscope smooth (but covered with adhesive glands visible under SEM). Ventral groove present (ciliated). Pygidium bilobed (with adhesive glands). Discrete head present; complex in shape bearing appendages.

Prostomium bluntly conical, eyes on head present, one pair; head eyes simple pigmented cups. Palps present (tentacle-like); anteroventral (bearing single internal canals); tapering (usually) sensory type; unarticulated. Nuchal organs present; paired low projections from posterolateral prostomium; indistinct dorsolateral ciliated patches. Peristomium a single ring.

Foregut a muscular ventral pharynx or without a distinct ventral or axial pharynx.

Protrusible proboscis present. Pharynx dorsolateral ciliated folds present. First segment chaetous; second segment chaetous. First chaetiger with neurochaetae only; parapodia similar in length or slightly shorter than subsequent parapodia; chaetae similar in orientation, length and thickness to other chaetae. Parapodia uniramous. Notopodial lobes absent. Neuropodial lobes represented by at least one chaetal lobe.

Chaetae first appear on variable, arranged as a single bundle (or row) of many chaetae (lower bundle - neurochaetae - only). Aciculae absent. Capillary chaetae absent. Forked chaetae present (truncate types also present), furcate, tines more-or-less equal in length, or tines distinctly unequal in length. Hooks present (tridentate); without distal hood, beard or ligament.

Sexual reproduction gonochoric. Segmental organs are protonephridia. Penis present. Tube absent.

**Literature used for description**. Worsaae, K, Kerbl, A., Di Domenico, M., Gonzalez, B.C., Bekkouche, N. & Martinez, A. (2021) Interstitial Annelida. Diversity 2021, 13, 77. https://doi.org/10.3390/d13020077; Di Domenico M, Worsaae K, Purschke G (2021) 7.11.2 Saccocirridae Czerniavsky, 1881 In: Purschke G, Böggemann M, Westheide W (Eds), Handbook of Zoology, Annelida, Volume 3: Pleistoannelida, Sedentaria III and Errantia I. De Gruyter, Berlin, 280–298; Rouse GW, Pleijel, F, Tilic E (2022) Annelida. Oxford University Press, Oxford, UK.

# Salifidae Johansson, 1910

**Description**. Body segment number: fixed, 34 segments including 2 preoral 'segments' (prostomium and peristomium) and 32 postoral segments. Secondary annulation present (disk-shaped receptors called sensillae mark the middle annulus of each segment); mid-body 6-annulate. Body shape elongate, more-or-less equal width over entire length (when relaxed; but can be dramatically shortened by contraction of longitudinal muscles during movement), dorsoventrally flattened. Anterior end sucker on ventral surface present; not clearly separated from rest of body. Large mouth on ventral surface of sucker. Posterior sucker on ventral surface present; anus positioned near posterior end (dorsally, just before posterior sucker).

Eyes on head present, four pairs or five pairs (four or more pairs arranged in an arc); head eyes simple pigmented cups.

Gut straight with side branches. Foregut a muscular axial pharynx; muscular axial pharynx ridges of pharynx rotated 60 degrees to the right (strepsilaematous). Pharynx jaws present, leech-type (either soft-muscular or horny) (as soft muscular 'jaws' or pseudognaths); two (duognathous) muscular jaws, or three (trignathous) muscular jaws arranged in a triangle; denticles present, paired, fine teeth or series of soft teeth. Protrusible proboscis absent. Crop present. Caeca of midgut (=posterior crop caeca) present (11 pairs). Circulatory system absent.

Sexual reproduction hermaphroditic. Clitellum fully encircles body, in region of male and female pores. Male and female pores separated by 4–5 annuli. Clitellum occurs from X-XIII (usual position for leeches). Gastropores absent, or present. Testes present, a few pairs, two pairs per segment. Sperm sac present. Ovaries present, one pair. Egg sacs present; globular. Oviduct separate one for each egg sac. Segmental organs are metanephridia. Female gonoduct present. Female pores single, median, in segment XII. Male gonoducts present, with atrium present, bilobed (cornua directed posteriorly; prostate tissue anteriorly). Male pores single, median, in segment XI. Prostate gland absent.

**Literature used for description**. Westergren S, Siddall PE (2004) Two new species of salifid leeches (Arhynchobdellida, Erpobdelliformes, Salifidae) from South Africa and Madagascar. American Museum Novitates, no. 3456: Siddall ME, Bely AE, Borda E (2006) Hirudinida Pp. 393–429. In Reproductive Biology and Phylogeny of Annelida. Series Editor BGM Jamieson. Volume 4. Editors G Rouse, F Pleijel. Science Publishers, Enfield, New Hampshire; OCEGUERA-FIGUEROA A, PHILLIPS AJ, PACHECO-CHAVES B, REEVES WK, SIDDALL, ME (2011) Phylogeny of macrophagous leeches (Hirudinea, Clitellata) based on molecular data and evaluation of the barcoding locus. Zoologica Scripta 40: 194–203; NAKANO T, RAMLAH Z, HIKIDA T (2012) Phylogenetic position of gastrostomobdellid leeches (Hirudinida, Arhynchobdellida, Erpobdelliformes) and a new family for the genus *Orobdella*. Zoologica Scripta 41: 177–185; Thorp JH, Lovell LL, Timm T, Martin P, Gelder SR, Govedich FR, Moser WE, Nakano T, Bielecki A, Bain BA, Utevsky S, Gil J, Glasby CJ, Martin D (2019) Phylum Annelida. In: Rogers DC, Thorp JH (Eds) Keys to Palaearctic Fauna: Thorp and Covich’s Freshwater invertebrates - Volume IV. Academic Press, Elsevier, pp. 357–518.

# Scalibregmatidae, Scalibregmatinae Malmgren, 1867

**Description**. Body segment number: variable. Secondary annulation absent or present (usually); anterior region biannulate; mid-body triannulate. Body shape widest anteriorly and tapering posteriorly or sausage or grub-shaped. Epidermis thick and rugose, or tesselated. Ventral groove present. Pygidium simple lobe. Pygidial appendages absent or present, more than four cirri or as lobes. Discrete head present; complex in shape bearing appendages.

Prostomium t-shaped, wide end anteriorly (antenna-like lateral extensions called frontal horns), , eyes on head absent or present, one pair (often large, bar or triangular shaped, sometimes merged); head eyes simple pigmented cups. Palps present (but referred to in the literature and prostomial horns or ciliated palps). Nuchal organs present; paired low projections from posterolateral prostomium; indistinct dorsolateral ciliated patches. Peristomium a single ring, may be partially subdivided into two rings.

Gut more-or-less straight, lacking side branches. Foregut a non-muscular axial pharynx.

First segment chaetous. First chaetiger with both notochaetae and neurochaetae; parapodia similar in length or slightly shorter than subsequent parapodia; parapodia more-or-less laterally directed and free from head (though notopodial spines in anterior parapodia may be forwardly projecting). Parapodia biramous (lobes may be more prominent posteriorly); parapodial lobes absent or very low, or prominent. Notopodial lobes represented by at least one chaetal lobe. Neuropodial lobes represented by at least one chaetal lobe. Interramal fleshy process absent, or present. Interramal fleshy process papilla-like. Lateral organs present. Dorsal cirri absent, or present (posteriorly); more-or-less cirriform. Ventral cirri absent, or present (posteriorly); cirriform or tapering. Branchiae present, arise from lateral body (at base of parapodia), occur on mid-body segments; lateral branchiae single tuft per parapodium.

Chaetae first appear on first segment after peristomium, arranged in paired bundles (or rows) of many chaetae. Aciculae absent. Capillary chaetae present, in dorsal (notopodial) position or in ventral (neuropodial) position, distally tapered to a point, edge smooth. Capillary chaetae, externally not pseudo-segmented. Capillary chaetae, internally not chambered or hollow. Forked chaetae present, lyrate, tines more-or-less equal in length. Spines absent, or present; slightly curved and more-or-less smooth; present only in one or a few anterior chaetigers; in both dorsal and ventral positions. Hooks absent.

Segmental organs are metanephridia; metanephridia along most of the body; anterior ones for excretion, posterior ones for gamete release (although appears to be uncertainty about whether posterior ones can function as oviducts); several pairs in anterior body.

Tube absent (construct galleries).

**Literature used for description**. Glasby CJ, Fauchald K (2003). POLiKEY. An information system for polychaete families and higher taxa. Version 2, 5 June 2003. Australian Biological Resources Study, DCCEEW, Canberra, https:[//www.dcceew.gov.au/;](http://www.dcceew.gov.au/%3B) Blake, JA (2019) 7.6.3 Scalibregmatidae Malgren, 1867 In: Purschke G, Böggemann M, Westheide W (Eds), Handbook of Zoology, Annelida, Volume 2: Pleistoannelida, Sedentaria II. De Gruyter, Berlin, 312–349; Rouse GW, Pleijel, F, Tilic E (2022) Annelida. Oxford University Press, Oxford, UK.

# Scalibregmatidae, *Travisia* [Johnston, 1840](https://www.marinespecies.org/aphia.php?p=taxdetails&id=129417),

**Description**. Body segment number: variable. Secondary annulation absent or present (usually); anterior region biannulate; mid-body biannulate, or triannulate; posterior biannulate. Body shape widest anteriorly and tapering posteriorly. Epidermis thick and rugose, or papillate (?). Ventral groove present. Pygidium simple lobe. Pygidial appendages absent or present, one pair of cirri or as lobes. Discrete head present; lobe-like without appendages.

Prostomium conical, tapering to slender tip. Palps absent. Nuchal organs present; paired low projections from posterolateral prostomium (slit-like). Peristomium a single ring.

Gut more-or-less straight, lacking side branches. Foregut a non-muscular axial pharynx.

First segment chaetous. First chaetiger with both notochaetae and neurochaetae; parapodia similar in length or slightly shorter than subsequent parapodia; parapodia more-or-less laterally directed and free from head; chaetae similar in orientation, length and thickness to other chaetae. Parapodia biramous; parapodial lobes absent or very low. Notopodial lobes represented by at least one chaetal lobe. Neuropodial lobes represented by at least one chaetal lobe. Interramal fleshy process absent, or present. Interramal fleshy process papilla-like. Lateral organs absent, or present. Branchiae present, arise from lateral body, occur first few segments, occur on mid-body segments, and occur near posterior end (from chaetiger 2 or 3); lateral branchiae digitiform, or branching (bifd or trifid).

Chaetae first appear on first segment after peristomium, arranged in paired bundles (or rows) of many chaetae. Capillary chaetae present, in dorsal (notopodial) position or in ventral (neuropodial) position, distally tapered to a point, edge smooth. Capillary chaetae, externally not pseudo-segmented. Capillary chaetae, internally not chambered or hollow.

Segmental organs are metanephridia; metanephridia along most of the body; anterior ones for excretion, posterior ones for gamete release;

Tube absent (construct galleries).

**Literature used for description**. Blake JA, Maciolek NJ (2019) 7.6.2 Travisiidae Hartmann-Schröder, 1971, new family status, In Purschke G, Böggemann M, Westheide W (Eds), Handbook of Zoology, Annelida, Volume 2: Pleistoannelida, Sedentaria II. De Gruyter, Berlin, 302–311; Rouse GW, Pleijel, F, Tilic E (2022) Annelida. Oxford University Press, Oxford, UK.

# Semiscolecidae Scriban & Autrum, 1934

**Description**. Body segment number: fixed, 34 segments including 2 preoral 'segments' (prostomium and peristomium) and 32 postoral segments. Secondary annulation present (disk-shaped receptors called sensillae mark the middle annulus of each segment); anterior region uniannulate, or biannulate; mid-body 5-annulate; posterior biannulate. Body shape elongate, more-or-less equal width over entire length (when relaxed; but can be dramatically shortened by contraction of longitudinal muscles during movement), dorsoventrally flattened. Anterior end sucker on ventral surface present; not clearly separated from rest of body. Large mouth on ventral surface of sucker. Epidermis papillate. Posterior sucker on ventral surface present; rays absent; anus positioned near posterior end (dorsally, just before posterior sucker).

Eyes on head absent or present, five pairs (on segments II-V and VII); head eyes simple pigmented cups.

Gut more-or-less straight, lacking side branches or straight with side branches. Foregut a muscular axial pharynx; muscular axial pharynx not rotated (euthylaematous). Pharynx jaws present, leech-type (either soft-muscular or horny) (as soft muscular 'jaws'); two (duognathous) muscular jaws, or three (trignathous) muscular jaws arranged in a triangle; denticles absent or present, one row of teeth. Protrusible proboscis absent. Crop present. Caeca of midgut (=posterior crop caeca) present (postcaeca may also be present). Caeca of hindgut absent. Circulatory system absent.

Sexual reproduction hermaphroditic. Clitellum fully encircles body, in region of male and female pores. Male and female pores separated by 5–8 annuli. Clitellum occurs from X-XIII (usual position for leeches). Testes present, many pairs (8 or 9 pairs), one pair per segment; present in segments XIV/XV to XXIII/XXIV. Sperm sac present. Ovaries present, one pair; present in segment XII. Egg sacs present; globular. Oviduct separate one for each egg sac. Vaginal sac present (well developed). Segmental organs are metanephridia; nephridial pores paired (ventrolateral), both nephridial pores and gonoducts located around clitellum. Female gonoduct present, vaginal sac present. Female pores single, median, in segment XII or XIII. Male gonoducts present, with atrium present, fused. Male pores single, median, in segment XI or XII. Penis present (straight on XI), with hardened sheath present (extending from XVI to XIX; with prostate glandular region). Prostate gland absent.

**Literature used for description**. Ringuelet RA (1972a) Nuevos taxia de Hirudineos neotropicos con la redefinicion de Semiscolecidae y la descripcion de Cyclobdellidae fam. nov. y Mesobdellidae fam. nov. Physis 31 (82): 193–201; Siddall ME, Borda E (2004) Leech Collections from Chile Including Two New species of *Helobdella* (Annelida: Hirudinida). American Museum Novitates 3457: 1–18; Siddall ME, Bely AE, Borda E (2006) Hirudinida Pp. 393–429. In Reproductive Biology and Phylogeny of Annelida. Series Editor BGM Jamieson. Volume 4. Editors G Rouse, F Pleijel. Science Publishers, Enfield, New Hampshire; Phillips AJ, Siddall ME (2009) Poly-paraphyly of Hirudinidae: many lineages of medicinal leeches. BMC Evolutionary Biology 9:

246. doi:10.1186/1471–2148–9-246.

# Serpulidae Rafinesque, 1815

**Description**. Body segment number: variable. Body shape elongate, more-or-less equal width over entire length, regionalization present (thoracic uncini in neuropodial position, abdominal uncini notopodial), regionalization comprising two regions (comprising 3–12 thoracic segments (usually 7) and 10 to over 100 abdominal segments), regions demarcated by inversion of parapodia (and shift in position of faecal groove from dorsal (thoracic) to ventral (abdominal), which is difficult to see). Body pigmentation absent or present (usually), radiolar crowns often brightly coloured; epidermis more-or-less smooth. Thoracic membranes present. Faecal groove present. Pygidium bilobed. Discrete head absent (as a radiolar crown comprising many radioles and one or two (rarely more) opercula); radiolar crown present (3 to many pairs of radioles (basally fused dorsally or unfused)); internal 'skeleton' absent; modified radioles present, as a singular opercular peduncle (rarely absent or more than one).

Eyes on head absent or present, numerous, unpaired (on radiolar crown or peristomium); head eyes simple pigmented cups, or compound, with lenses. Nuchal organs absent (internalized). Peristomium a single ring, collar-like.

Foregut without a distinct ventral or axial pharynx. Protrusible proboscis absent. Heart bodies absent.

First segment chaetous. First chaetiger with notochaetae only (called collar chaetae); parapodia similar in length or slightly shorter than subsequent parapodia; parapodia more-or-less laterally directed and free from head; chaetae similar in orientation, length and thickness to other chaetae. Parapodia biramous; parapodial lobes prominent. Notopodial lobes represented by at least one chaetal lobe (anteriorly), or low lateral ridges (tori) (posteriorly). Neuropodial lobes low ridges (tori) (anteriorly), or represented by at least one chaetal lobe (posteriorly).

Chaetae first appear on first segment after peristomium, arranged as a single bundle (or row) of many chaetae (collar chaetae). Aciculae absent. Capillary chaetae present, in dorsal (notopodial) position or in ventral (neuropodial) position, distally tapered to a point or sharply bent or with subdistal spur, edge smooth or hirsute-serrate. Capillary chaetae, externally not pseudo-segmented. Capillary chaetae, internally not chambered or hollow. Comb-like chaetae absent, or present (in abdomen only). Comb-like chaetae comb comprised of many short tines. Hooks absent. Uncini present (avicular type; i.e., short shafts); with teeth in vertical series, teeth usually similar-sized (=pectinate); in ventral (neuropodial) position on thorax and dorsal (notopodial) position on abdomen; arranged in one row.

Reproduction asexual, or sexual. Asexual reproduction by paratomy.

Sexual reproduction hermaphroditic (mostly small forms such as Spirorbinae), or gonochoric. Segmental organs are metanephridia; metanephridia single anterior pair of excretory metanephridia and several more posterior ones for gamete release (excretory ones on segment 1); nephridial pores located anteriorly.

Tube present; hard, composed of calcium carbonate (includes spiral forms, tusk-shaped forms, and irregular-shaped forms; tubes may be embedded in coral and resemble a burrow); entrance able to be plugged by worm (operculum) usually present.

**Literature used for description**. Glasby CJ, Fauchald K (2003). POLiKEY. An information system for polychaete families and higher taxa. Version 2, 5 June 2003. Australian Biological Resources Study, DCCEEW, Canberra, https:[//www.dcceew.gov.au/;](http://www.dcceew.gov.au/%3B) Kupriyanova EK, Rzhavsky AV, ten Hove HA (2019) 7.4.7 Serpulidae Rafinesque, 1815 In: Purschke G, Böggemann M, Westheide W (Eds), Handbook of Zoology, Annelida, Volume 2: Pleistoannelida, Sedentaria II. De Gruyter, Berlin, 213–275; Capa M, Kupriyanova E, Nogueira JMdM, Bick A, Tovar-Hernández MA (2021) Fanworms: Today and Tomorrow. Diversity 13: 130, https://doi.org/10.3390/d13030130; Rouse GW, Pleijel F, Tilic E (2022) Annelida. Oxford University Press, Oxford, UK.

# Siboglinidae Caullery, 1914, sensu lato

**Description**. Body segmentation present (poorly demarked; indicated in posterior body by presence of chaetae rather annuli). Body segments strongly elongate in midbody (an elongated trunk consisting of a single segment), elongate body segments with indistinct parapodia, chaetae (uncini) arising from raised annuli. Body shape elongate, more-or-less equal width over entire length, regionalization present (short single-segment anterior region; an elongate single-segment midsection; a short posterior multisegmented 'opisthosoma'), regionalization comprising three regions, regions demarcated by structural differences in parapodia over body. Epidermis more-or-less smooth, or papillate (referred to as cuticular plaques). Caudal region very short, multisegmented; multisegmented caudal region with rows of uncini on each segment, or four peg-like chaetae in most segments, or long-handled hooks. Pygidium simple lobe. Pygidial appendages absent. Discrete head absent, or present (bearing 1 to over 200 separate thin tentacles, which may be fused to form a crown or plume); complex in shape bearing appendages.

Prostomium bluntly conical (referred to as a cephalic lobe). Buccal tentacles present; arising anteroventrally (mouth absent); smooth, or pinnulated. Nuchal organs absent. Peristomium a single ring, or an expanded glandular ring, or an expanded, elaborately collared ring (often elongate but maybe indistinct).

Gut absent.

First segment achaetous; second segment with a very long region comprising most of the body. Parapodia present (only in far posterior body). Parapodia uniramous. Notopodial lobes low lateral ridges (tori). Neuropodial lobes absent.

Chaetae present, inconspicuous, only present posteriorly. Aciculae absent. Capillary chaetae absent. Hooks absent, or present; without distal hood, beard or ligament; occur in posterior body only. Uncini absent, or present; with multiple teeth arranged in two opposing groups; arranged in one row. Segmental organs are metanephridia; metanephridia single anterior pair of excretory metanephridia and several more posterior ones for gamete release (excretory ones on segment 1); nephridial pores located anteriorly, gonoducts located around clitellum.

Tube present; membranous, or leathery or parchment like (may appear segmented); entrance able to be plugged by worm may be present.

**Literature used for description**. Glasby CJ, Fauchald K (2003). POLiKEY. An information system for polychaete families and higher taxa. Version 2, 5 June 2003. Australian Biological Resources Study, DCCEEW, Canberra, https:[//www.dcceew.gov.au/;](http://www.dcceew.gov.au/%3B) Southward, EC (2000), Chapter 3. Class Pogonophora, pp. 330–351 In CJ Glasby et al. (Eds) Fauna of Australia Vol. 4A. Polychaetes & Allies. The Southern Synthesis. ABRS, Canberra; Brusca, RC, Moore, W & Shuster, SM. (eds) (2016) Chapter 14. Phylum Annelida. In: Invertebrates, 3rd Edition. Sinauer Associates, Sunderland, MA; Rouse GW, Pleijel F, Tilic E (2022) Annelida. Oxford University Press, Oxford, UK.

# Siboglinidae, Frenulata Webb, 1969

**Description**. Body segmentation present (poorly demarked; indicated in posterior body by presence of chaetae rather annuli). Body segments strongly elongate in midbody (an elongated trunk consisting of a single segment), elongate body segments with indistinct parapodia, chaetae (uncini) arising from raised annuli. Body shape elongate, more-or-less equal width over entire length, regionalization present (short single-segment 'anterior gland region'; an elongate single-segment trunk; a short posterior multisegmented 'opisthosoma'), regionalization comprising three regions, regions demarcated by change in chaetal types over body (girdle of chaetae half way along trunk) or structural differences in parapodia over body. Body translucent, gut visible; epidermis more-or-less smooth, or papillate (referred to as cuticular plaques). Caudal region very short, multisegmented; multisegmented caudal region with four peg-like chaetae in most segments. Pygidium simple lobe. Pygidial appendages absent. Discrete head present; complex in shape bearing appendages.

Prostomium bluntly conical (referred to as a cephalic lobe). Buccal tentacles present (one to many; may be partially fused together); arising anteroventrally (mouth absent); pinnulated. Nuchal organs absent. Peristomium an expanded glandular ring (elongate).

Gut absent.

First segment achaetous (an elongated segment bearing a distinct ridge line; called the frenulum); second segment with a very long region comprising most of the body (bearing papillae and divided into two regions by two rings of uncini). Parapodia present (only in far posterior body). Parapodia uniramous. Notopodial lobes low lateral ridges (tori). Neuropodial lobes absent.

Chaetae present, inconspicuous, only present posteriorly. Aciculae absent. Capillary chaetae absent. Hooks absent. Uncini present; with multiple teeth arranged in two opposing groups; arranged in one row.

Segmental organs are metanephridia; metanephridia single anterior pair of excretory metanephridia and several more posterior ones for gamete release (excretory ones in segment 1; gonopores in segment 2); nephridial pores located anteriorly.

Tube present; leathery or parchment like (tubes 3–4 times longer than worm; may appear segmented).

**Literature used for description**. Southward, EC (2000), Chapter 3. Class Pogonophora, pp. 330–351 In CJ Glasby et al. (Eds) Fauna of Australia Vol. 4A. Polychaetes & Allies. The Southern Synthesis. ABRS, Canberra; Brusca, RC, Moore, W & Shuster, SM. (eds) (2016) Chapter 14. Phylum Annelida. In: Invertebrates, 3rd Edition. Sinauer Associates, Sunderland, MA; Rouse GW, Pleijel F, Tilic E (2022) Annelida. Oxford University Press, Oxford, UK.

# Siboglinidae, *Osedax* Rouse, Goffredi & Vrijenhoek, 2004

**Description**. Body segmentation present (poorly demarked; indicated in posterior body by presence of chaetae rather annuli). Body segments strongly elongate in midbody (an elongated trunk consisting of a single segment), elongate body segments with indistinct parapodia, chaetae (uncini) arising from raised annuli. Body shape elongate, more-or-less equal width over entire length, regionalization present (check), regionalization comprising three regions, regions demarcated by structural differences in parapodia over body. Epidermis more-or-less smooth, or papillate (referred to as cuticular plaques). Caudal region unmodified typical segments (but shorter and with reduced parapodia), or very short, multisegmented; multisegmented caudal region with long-handled hooks. Pygidium simple lobe. Pygidial appendages absent. Discrete head absent (bearing 2 or 4 tentacles).

Buccal tentacles present (2–4); arising anteroventrally (mouth absent); pinnulated. Nuchal organs absent. Peristomium a single ring.

Gut absent.

First segment achaetous; second segment with a very long region comprising most of the body (unadorned with papillae or uncini). Parapodia present (only in far posterior body). Parapodia uniramous. Notopodial lobes low lateral ridges (tori). Neuropodial lobes absent.

Chaetae present, inconspicuous, , only present posteriorly. Aciculae absent. Capillary chaetae absent. Hooks present; without distal hood, beard or ligament; occur in posterior body only.

Tube present; membranous (transparent).

**Literature used for description**. Southward, EC (2000), Chapter 3. Class Pogonophora, pp. 330–351 In CJ Glasby et al. (Eds) Fauna of Australia Vol. 4A. Polychaetes & Allies. The Southern Synthesis. ABRS, Canberra; Brusca, RC, Moore, W & Shuster, SM. (eds) (2016) Chapter 14. Phylum Annelida. In: Invertebrates, 3rd Edition. Sinauer Associates, Sunderland, MA; Rouse GW, Pleijel F, Tilic E (2022) Annelida. Oxford University Press, Oxford, UK.

# Siboglinidae, *Sclerolinum* Southward, 1961

**Description**. Body segmentation present (poorly demarked; indicated in posterior body by presence of chaetae rather annuli). Body segments strongly elongate in midbody (an elongated trunk consisting of a single segment), elongate body segments with indistinct parapodia, chaetae (uncini) arising from raised annuli. Body shape elongate, more-or-less equal width over entire length, regionalization present (short single-segment anterior region; an elongate single-segment midsection; a short posterior multisegmented 'opisthosoma'), regionalization comprising three regions, regions demarcated by structural differences in parapodia over body. Epidermis more-or-less smooth, or papillate (referred to as plaque-capped papillae). Caudal region very short, multisegmented; multisegmented caudal region with rows of uncini on each segment. Pygidium simple lobe. Pygidial appendages absent. Discrete head present (usually bearing a single tentacle); complex in shape bearing appendages.

Prostomium bluntly conical (referred to as a cephalic lobe). Buccal tentacles present (one pair); arising anteroventrally (mouth absent); smooth. Nuchal organs absent. Peristomium a single ring.

Gut absent.

First segment achaetous (an elongated segment bearing a few crests; called the frenulum); second segment with a very long region comprising most of the body. Parapodia present (only in far posterior body). Parapodia uniramous. Notopodial lobes low lateral ridges (tori). Neuropodial lobes absent.

Chaetae present, inconspicuous, only present posteriorly. Aciculae absent. Capillary chaetae absent. Hooks absent. Uncini present; with multiple teeth arranged in two opposing groups; arranged in one row.

Tube present; leathery or parchment like (often ringed and two or three times longer than worm).

**Literature used for description**. Southward, EC (2000), Chapter 3. Class Pogonophora, pp. 330–351 In CJ Glasby et al. (Eds) Fauna of Australia Vol. 4A. Polychaetes & Allies. The Southern Synthesis. ABRS, Canberra; Brusca, RC, Moore, W & Shuster, SM. (eds) (2016) Chapter 14. Phylum Annelida. In: Invertebrates, 3rd Edition. Sinauer Associates, Sunderland, MA; Rouse GW, Pleijel F, Tilic E (2022) Annelida. Oxford University Press, Oxford, UK.

# Siboglinidae, Vestimentifera Webb, 1969

**Description**. Body segmentation present (poorly demarked; indicated in posterior body by presence of chaetae rather annuli). Body segments strongly elongate in midbody (an elongated trunk consisting of a single segment), elongate body segments with indistinct parapodia, chaetae (uncini) arising from raised annuli. Body shape elongate, more-or-less equal width over entire length, regionalization present (short single-segment anterior region; an elongate single-segment midsection; a short posterior multisegmented 'opisthosoma'), regionalization comprising three regions, regions demarcated by change in chaetal types over body (no chaetae on trunk region) or structural differences in parapodia over body. Body pigmentation present, bright red palpal plumes; epidermis more-or-less smooth, or papillate (referred to as cuticular plaques). Caudal region very short, multisegmented; multisegmented caudal region with rows of uncini on each segment. Pygidium simple lobe. Pygidial appendages absent. Discrete head absent, or present (absent in adults, presumably obscured by mass of tentacles); complex in shape bearing appendages.

Prostomium bluntly conical (referred to as a cephalic lobe - only visible in juveniles). Buccal tentacles present (many; may be fused to the paired obteraculum (=?dorsal lips); collectively referred to as the papal plume); arising anteroventrally (mouth absent); pinnulated. Nuchal organs absent. Peristomium an expanded, elaborately collared ring (elongate).

Gut absent.

First segment achaetous (very elongated and vascularised; called a vestimentum); second segment with a very long region comprising most of the body (unadorned with papillae or uncini). Parapodia present (only in far posterior body). Parapodia uniramous. Notopodial lobes low lateral ridges (tori). Neuropodial lobes absent.

Chaetae present, inconspicuous, only present posteriorly. Aciculae absent. Capillary chaetae absent. Hooks absent. Uncini present; with multiple teeth arranged in two opposing groups; arranged in one row.

Segmental organs are metanephridia; metanephridia single anterior pair of excretory metanephridia and several more posterior ones for gamete release (excretory nephridia in segment 1, gonopores in segment 2); nephridial pores located anteriorly.

Tube present; leathery or parchment like; entrance able to be plugged by worm (an obturaculum seals the tube).

**Literature used for description**. Southward, EC (2000), Chapter 3. Class Pogonophora, pp. 330–351 In CJ Glasby et al. (Eds) Fauna of Australia Vol. 4A. Polychaetes & Allies. The Southern Synthesis. ABRS, Canberra; Brusca, RC, Moore, W & Shuster, SM. (eds) (2016) Chapter 14. Phylum Annelida. In: Invertebrates, 3rd Edition. Sinauer Associates, Sunderland, MA; Rouse GW, Pleijel F, Tilic E (2022) Annelida. Oxford University Press, Oxford, UK.

# Sigalionidae Kinberg, 1856, sensu lato

**Description**. Body segment number: variable. Body shape elongate, more-or-less equal width over entire length or ovate to elliptical. Body pigmentation absent or present; epidermis more-or-less smooth. Body surface with protective covering as scales (elytrae), dorsally, or absent. Pygidium simple lobe. Pygidial appendages present, one pair of cirri. Discrete head present; complex in shape bearing appendages.

Prostomium rounded to oval, facial tubercle absent or present, eyes on head absent or present, one pair or two pairs; head eyes compound, with lenses. Prostomial antennae present; median and paired laterals (usually; median may be absent or minute); unarticulated (median one may have lateral lobe-shaped auricles at base), or consist of basal ceratophore and distal ceratostyle. Palps present; anteroventral, or lateral; tapering (usually) sensory type; unarticulated (basal palpophore may be present). Nuchal organs absent, or present; paired low projections from posterolateral prostomium; indistinct dorsolateral ciliated patches. Peristomium not visible.

Gut more-or-less straight, lacking side branches. Foregut a muscular axial pharynx. Pharynx jaws present, two pairs of jaws. Paired jaws fang-like. Protrusible proboscis present, distal ring of papillae present.

First segment chaetous and tentaculate (dorsal and/or ventral cirri enlarged and elongated); second segment chaetous. Tentacular cirri present; 1–2 pair(s); arise on a single segment. First chaetiger without external chaetae, or with neurochaetae only, or with both notochaetae and neurochaetae; parapodia similar in length or slightly shorter than subsequent parapodia; parapodia anteriorly directed and wrapping around head; chaetae similar in orientation, length and thickness to other chaetae. Parapodia biramous; parapodial lobes prominent. Notopodial lobes represented by at least one chaetal lobe. Neuropodial lobes represented by at least one chaetal lobe. Dorsal cirri present (very short; present on non-elytrous segments); more-or-less cirriform. Elytra with raised concentric rings, or with papillae, tubercles or smooth. Elytra, post segment 7 occur on every other segment from segment 7 to end of body, or occur on every other segment from segment 7 to midbody, then present on every segment, or absent, to end of body. Ventral cirri present; cirriform or tapering. Branchiae absent or present, arise from lateral body (on elytrophores or dorsal tubercles), occur on mid-body segments or occur near posterior end; lateral branchiae digitiform.

Chaetae first appear on first segment after peristomium or second segment after peristomium (or over next few segments), arranged in paired bundles (or rows) of many chaetae. Aciculae present; in both dorsal and ventral positions (=noto- and neuroaciculae). Capillary chaetae present, in dorsal (notopodial) position or in ventral (neuropodial) position, distally tapered to a point, edge hirsute-serrate or spinose or corn-eared. Silky (feltage) chaetae arising from notopodia of mid-posterior body absent. Hooks absent. Compound chaetae present; in ventral (neuropodial) position; appendage distally tapering to slender tips (=spinigerous), or distally curved (=falcate; ie., one distal tooth); appendage not canaliculated, or canaliculated; appendage without hoods or guards; joint, appearance distinctly asymmetrical (=heterogomph), or flat or pseudocompound; joint effected by ligament(s); shaft tapering slightly or evenly thick from emergence to joint; shaft, internally solid, without distinct core. Compound chaetae present in most or all chaetigers.

Segmental organs are protonephridia, or metanephridia; metanephridia along most of the body; anterior ones for excretion, posterior ones for gamete release.

Tube absent, or present.

**Literature used for description**. Glasby CJ, Fauchald K (2003). POLiKEY. An information system for polychaete families and higher taxa. Version 2, 5 June 2003. Australian Biological Resources Study, DCCEEW, Canberra, https:[//www.dcceew.gov.au/;](http://www.dcceew.gov.au/%3B) Eibye-Jacobsen D, Aungtonya C, Gonzalez BC (2022) 7.13.1.5.1. Sigalionidae Kinberg, 1856, In: Purschke G, Böggemann M, Westheide W (Eds), Handbook of Zoology, Annelida, Volume 3: Pleistoannelida, Errantia II. De Gruyter, Berlin, 113–138.

# Sigalionidae, Pelogeniinae Chamberlin, 1919

**Description**. Body segment number: variable. Body shape elongate, more-or-less equal width over entire length. Body pigmentation absent or present; epidermis more-or-less smooth. Body surface with protective covering as scales (elytrae), dorsally (sand grains often adhering to scales). Pygidium simple lobe. Pygidial appendages present, one pair of cirri. Discrete head present; complex in shape bearing appendages.

Prostomium rounded to oval, facial tubercle absent, eyes on head absent or present, two pairs (often); head eyes compound, with lenses. Prostomial antennae present; median and paired laterals (usually; median may be absent or minute). Palps present; anteroventral; tapering (usually) sensory type; unarticulated (basal palpophore may be present). Nuchal organs present; paired low projections from posterolateral prostomium; indistinct dorsolateral ciliated patches. Peristomium not visible.

Gut more-or-less straight, lacking side branches. Foregut a muscular axial pharynx. Pharynx jaws present, two pairs of jaws. Paired jaws fang-like. Protrusible proboscis present. First segment chaetous and tentaculate; second segment chaetous. Tentacular cirri present; 1–2 pair(s); arise on a single segment. First chaetiger without external chaetae, or with neurochaetae only, or with both notochaetae and neurochaetae; parapodia similar in length or slightly shorter than subsequent parapodia; parapodia anteriorly directed and wrapping around head; chaetae similar in orientation, length and thickness to other chaetae. Parapodia biramous; parapodial lobes prominent. Notopodial lobes represented by at least one chaetal lobe. Neuropodial lobes represented by at least one chaetal lobe. Dorsal cirri absent (if present, only on segment 3). Elytra with papillae, tubercles or smooth. Elytra, post segment 7 occur on every other segment from segment 7 to midbody, then present on every segment, or absent, to end of body. Ventral cirri present; cirriform or tapering. Branchiae present, arise from lateral body, occur on mid-body segments or occur near posterior end; lateral branchiae digitiform.

Chaetae first appear on first segment after peristomium or second segment after peristomium (or over next few segments), arranged in paired bundles (or rows) of many chaetae. Aciculae present; in both dorsal and ventral positions (=noto- and neuroaciculae). Capillary chaetae present, in dorsal (notopodial) position, distally tapered to a point. Silky (feltage) chaetae arising from notopodia of mid-posterior body absent. Hooks absent. Compound chaetae present; in ventral (neuropodial) position; appendage without hoods or guards. Compound chaetae present in most or all chaetigers.

Tube absent.

**Literature used for description**. Eibye-Jacobsen D, Aungtonya C, Gonzalez BC (2022) 7.13.1.5.1. Sigalionidae Kinberg, 1856, In: Purschke G, Böggemann M, Westheide W (Eds), Handbook of Zoology, Annelida, Volume 3: Pleistoannelida, Errantia II. De Gruyter, Berlin, 113–138.

# Sigalionidae, Pholoinae Kinberg, 1858

**Description**. Body segment number: variable. Body shape elongate, more-or-less equal width over entire length or ovate to elliptical. Body pigmentation absent or present; epidermis more-or-less smooth. Body surface with protective covering as scales (elytrae), dorsally. Pygidium simple lobe. Pygidial appendages present, one pair of cirri. Discrete head present; complex in shape bearing appendages.

Prostomium rounded to oval, facial tubercle present, eyes on head present, two pairs (often); head eyes compound, with lenses. Prostomial antennae present; median and paired laterals (usually; median may be absent or minute); consist of basal ceratophore and distal ceratostyle. Palps present; anteroventral; tapering (usually) sensory type; unarticulated (basal palpophore may be present). Nuchal organs absent, or present; paired low projections from posterolateral prostomium; indistinct dorsolateral ciliated patches. Peristomium not visible.

Gut more-or-less straight, lacking side branches. Foregut a muscular axial pharynx. Pharynx jaws present, two pairs of jaws. Paired jaws fang-like. Protrusible proboscis present, distal ring of papillae present.

First segment tentaculate (dorsal and ventral cirri often enlarged and elongated); second segment chaetous. Tentacular cirri present; 2 pair(s); arise on a single segment. First chaetiger without external chaetae, or with neurochaetae only, or with both notochaetae and neurochaetae; parapodia similar in length or slightly shorter than subsequent parapodia; parapodia anteriorly directed and wrapping around head; chaetae similar in orientation, length and thickness to other chaetae. Parapodia biramous; parapodial lobes prominent. Notopodial lobes represented by at least one chaetal lobe. Neuropodial lobes represented by at least one chaetal lobe. Dorsal cirri present (very short; present on non-elytrous segments); more-or-less cirriform. Elytra with raised concentric rings. Elytra, post segment 7 occur on every other segment from segment 7 to end of body. Ventral cirri present; cirriform or tapering. Branchiae absent.

Chaetae first appear on first segment after peristomium or second segment after peristomium (or over next few segments), arranged in paired bundles (or rows) of many chaetae. Aciculae present; in both dorsal and ventral positions (=noto- and neuroaciculae). Capillary chaetae present, in dorsal (notopodial) position, distally tapered to a point, edge spinose. Hooks absent. Compound chaetae present; in ventral (neuropodial) position; appendage distally tapering to slender tips (=spinigerous), or distally curved (=falcate; ie., one distal tooth); appendage not canaliculated; appendage without hoods or guards; joint, appearance distinctly asymmetrical (=heterogomph); joint effected by ligament(s); shaft tapering slightly or evenly thick from emergence to joint; shaft, internally solid, without distinct core. Compound chaetae present in most or all chaetigers.

Segmental organs are metanephridia; metanephridia along most of the body; anterior ones for excretion, posterior ones for gamete release.

Tube absent.

**Literature used for description**. Pettibone M (1992) Contribution to the Polychaete Family Pholoidae Kinberg. Smithsonian Contributions to Zoology 532: i-iii, 24pp; Barnich R; Fiege D (2003). The Aphroditoidea (Annelida: Polychaeta) of the Mediterranean Sea. Abhandlungen der Senckenbergischen Naturforschenden Gesellschaft. 559: 1–167.

# Sigalionidae, Pisioninae Ehlers, 1901

**Description**. Body segment number: variable. Body shape elongate, more-or-less equal width over entire length. Body pigmentation absent or present; epidermis more-or-less smooth. Pygidium simple lobe. Pygidial appendages present, one pair of cirri. Discrete head present; complex in shape bearing appendages.

Prostomium conical, tapering to slender tip (highly reduced in *Pisione*), facial tubercle absent, eyes on head absent or present, two pairs (often); head eyes compound, with lenses. Postcephalic eyes absent, or present; subepidermal; 1 annulus with a pair of eyes. Prostomial antennae present; paired, lateral. Palps present; lateral; tapering (usually) sensory type; unarticulated (basal palpophore may be present). Nuchal organs absent. Peristomium not visible.

Gut more-or-less straight, lacking side branches. Foregut a muscular axial pharynx. Pharynx jaws present, two pairs of jaws. Paired jaws fang-like. Protrusible proboscis present.

First segment chaetous and tentaculate (dorsal and/or ventral cirri enlarged and elongated); second segment chaetous (often reduced compared to subsequent ones). Tentacular cirri present; 1 pair(s); arise on a single segment; internal aciculae present in at least some cirri. First chaetiger without external chaetae; parapodia similar in length or slightly shorter than subsequent parapodia, or very elongated; parapodia anteriorly directed and wrapping around head; chaetae similar in orientation, length and thickness to other chaetae. Parapodia biramous (lobes very reduced, and lacking external chaetae in anterior segments); parapodial lobes absent or very low, or prominent. Notopodial lobes represented by at least one chaetal lobe. Neuropodial lobes represented by at least one chaetal lobe. Dorsal cirri present; small, articulated and flask-shaped. Ventral cirri present; small, articulated and flask-shaped. Branchiae absent.

Chaetae first appear on second segment after peristomium (or over next few segments), arranged as a single bundle (or row) of many chaetae (upper bundle - notochaetae - absent). Aciculae present; in both dorsal and ventral positions (=noto- and neuroaciculae). Capillary chaetae absent. Silky (feltage) chaetae arising from notopodia of mid-posterior body absent. Spines absent, or present (rare); slightly curved and more-or-less smooth. Hooks absent. Compound chaetae present; in ventral (neuropodial) position; appendage multidentate; appendage without hoods or guards. Compound chaetae present in most or all chaetigers.

Segmental organs are protonephridia. Tube absent.

**Literature used for description**. Gonzalez BC, Worsaae K, Eibye-Jacobsen D (2022) 7.13.1.5.1. Sigalionidae, Pisioninae Ehlers, 1901, In: Purschke G, Böggemann M, Westheide W (Eds), Handbook of Zoology, Annelida, Volume 3: Pleistoannelida, Errantia II. De Gruyter, Berlin, 138–151.

# Sigalionidae, Sigalioninae Kinberg, 1856

**Description**. Body segment number: variable. Body shape elongate, more-or-less equal width over entire length. Body pigmentation absent or present, often brilliant white with red highlights on prostomium, scales or mid-ventrum; epidermis more-or-less smooth. Body surface with protective covering as scales (elytrae), dorsally. Pygidium simple lobe. Pygidial appendages present, one pair of cirri. Discrete head present; complex in shape bearing appendages.

Prostomium rounded to oval, facial tubercle absent, eyes on head absent or present, two pairs (often); head eyes compound, with lenses. Prostomial antennae present; median and paired laterals (usually; median may be absent or minute); unarticulated (median one may have lateral lobe-shaped auricles at base). Palps present; anteroventral; tapering (usually) sensory type; unarticulated (basal palpophore may be present). Nuchal organs present; paired low projections from posterolateral prostomium; indistinct dorsolateral ciliated patches. Peristomium not visible.

Gut more-or-less straight, lacking side branches. Foregut a muscular axial pharynx. Pharynx jaws present, two pairs of jaws. Paired jaws fang-like. Protrusible proboscis present.

First segment chaetous and tentaculate (dorsal and/or ventral cirri enlarged and elongated); second segment chaetous. Tentacular cirri present; 1–2 pair(s); arise on a single segment. First chaetiger without external chaetae, or with neurochaetae only, or with both notochaetae and neurochaetae; parapodia similar in length or slightly shorter than subsequent parapodia; parapodia anteriorly directed and wrapping around head; chaetae similar in orientation, length and thickness to other chaetae. Parapodia biramous; parapodial lobes prominent. Notopodial lobes represented by at least one chaetal lobe. Neuropodial lobes represented by at least one chaetal lobe. Dorsal cirri present (very short; present on non-elytrous segments); more-or-less cirriform. Elytra with papillae, tubercles or smooth. Elytra, post segment 7 occur on every other segment from segment 7 to midbody, then present on every segment, or absent, to end of body. Ventral cirri present; cirriform or tapering. Branchiae present, arise from lateral body, occur on mid-body segments or occur near posterior end; lateral branchiae digitiform.

Chaetae first appear on first segment after peristomium or second segment after peristomium (or over next few segments), arranged in paired bundles (or rows) of many chaetae. Aciculae present; in both dorsal and ventral positions (=noto- and neuroaciculae). Capillary chaetae present, in dorsal (notopodial) position, distally tapered to a point, edge hirsute-serrate or spinose or corn-eared. Silky (feltage) chaetae arising from notopodia of mid-posterior body absent. Hooks absent. Compound chaetae present; in ventral (neuropodial) position; appendage distally tapering to slender tips (=spinigerous), or distally curved (=falcate; ie., one distal tooth) (and pseudocompound types); appendage without hoods or guards; joint, appearance distinctly asymmetrical (=heterogomph), or flat or pseudocompound. Compound chaetae present in most or all chaetigers.

Segmental organs are metanephridia; metanephridia along most of the body; anterior ones for excretion, posterior ones for gamete release.

Tube absent.

**Literature used for description**. Eibye-Jacobsen D, Aungtonya C, Gonzalez BC (2022) 7.13.1.5.1. Sigalionidae Kinberg, 1856, In: Purschke G, Böggemann M, Westheide W (Eds), Handbook of Zoology, Annelida, Volume 3: Pleistoannelida, Errantia II. De Gruyter, Berlin, 113–138.

# Sigalionidae, Sthenelanellinae Gonzalez, Martínez, Borda, Iliffe, Eibye-Jacobsen & Worsaae, 2018

**Description**. Body segment number: variable. Body shape elongate, more-or-less equal width over entire length. Epidermis more-or-less smooth. Body surface with protective covering as scales (elytrae), dorsally. Pygidium simple lobe. Pygidial appendages present, one pair of cirri. Discrete head present; complex in shape bearing appendages.

Prostomium rounded to oval, facial tubercle absent, eyes on head absent or present, two pairs (often); head eyes compound, with lenses. Prostomial antennae present; median and paired laterals (usually; median may be absent or minute). Palps present; anteroventral; tapering (usually) sensory type; unarticulated (basal palpophore may be present). Nuchal organs present; paired low projections from posterolateral prostomium; indistinct dorsolateral ciliated patches. Peristomium not visible.

Gut more-or-less straight, lacking side branches. Foregut a muscular axial pharynx. Pharynx jaws present, two pairs of jaws. Paired jaws fang-like. Protrusible proboscis present.

First segment chaetous and tentaculate (dorsal and/or ventral cirri enlarged and elongated); second segment chaetous. Tentacular cirri present; 1–2 pair(s); arise on a single segment. First chaetiger without external chaetae, or with neurochaetae only, or with both notochaetae and neurochaetae; parapodia similar in length or slightly shorter than subsequent parapodia; parapodia anteriorly directed and wrapping around head; chaetae similar in orientation, length and thickness to other chaetae. Parapodia biramous; parapodial lobes prominent. Notopodial lobes represented by at least one chaetal lobe. Neuropodial lobes represented by at least one chaetal lobe. Dorsal cirri present (very short; present on non-elytrous segments); more-or-less cirriform. Elytra with papillae, tubercles or smooth. Elytra, post segment 7 occur on every other segment from segment 7 to midbody, then present on every segment, or absent, to end of body. Ventral cirri present; cirriform or tapering. Branchiae present, arise from lateral body, occur on mid-body segments or occur near posterior end; lateral branchiae digitiform.

Chaetae first appear on first segment after peristomium or second segment after peristomium (or over next few segments), arranged in paired bundles (or rows) of many chaetae. Aciculae present; in both dorsal and ventral positions (=noto- and neuroaciculae). Capillary chaetae present, in dorsal (notopodial) position, distally tapered to a point. Silky (feltage) chaetae arising from notopodia of mid-posterior body absent, or present (produced in notopodia of chaetiger 2). Silky (feltage) chaetae incorporated into tube. Hooks absent. Compound chaetae present; in ventral (neuropodial) position; appendage without hoods or guards. Compound chaetae present in most or all chaetigers.

Tube present; leathery or parchment like (tube constructed with feltage chaetae).

**Literature used for description**. Eibye-Jacobsen D, Aungtonya C, Gonzalez BC (2022) 7.13.1.5.1. Sigalionidae Kinberg, 1856, In: Purschke G, Böggemann M, Westheide W (Eds), Handbook of Zoology, Annelida, Volume 3: Pleistoannelida, Errantia II. De Gruyter, Berlin, 113–138.

# Sipuncula Stephen, 1965, sensu lato

**Description**. Body segmentation absent. Body shape peanut shaped, regionalization present, regionalization comprising two regions, regions demarcated by presence of unsegmented trunk and a narrower, long anterior proboscis (introvert capable of rolling in and out). Introvert shorter than trunk or about equal to trunk or longer than trunk, hooks absent or present, papillae absent or present. Trunk smooth or roughened by papillae or rounded skin bodies, anterior extremity of trunk with hardened or calcareous structures absent or present, posterior trunk with shield absent or present. Body pigmentation absent or present; epidermis more-or-less smooth, or papillate (introvert often covered with epidermal hooks). Anus positioned near anterior end, dorsally, near the introvert-trunk junction. Pygidium absent. Elongate proboscis present; retractable and tube-like (a large introvert). Discrete head absent (represented by tentacles which are only visible if introvert is fully everted).

Postcephalic eyes absent, or present; epidermal. Buccal tentacles present; arising from one side of mouth, or surrounding perimeter of mouth; ciliated. Nuchal organs present; on dorsal side of anteriorly projecting introvert organ. Peristomium not visible.

Gut coiled (descending and ascending branches of the intestine wrap around each other). Foregut without a distinct ventral or axial pharynx. Protrusible proboscis absent. Caeca of foregut absent. Caeca of midgut (=posterior crop caeca) absent. Caeca of hindgut absent, or present.

Chaetae absent.

Segmental organs are metanephridia; several pairs in anterior body.

**Literature used for description**. Edmonds SJ (2000) Chapter 5. Phylum Sipuncula. In Beesley PL, Ross GJB, Glasby CJ (Eds) Fauna of Australia Vol. 4A. Polychaetes & Allies. The Southern Synthesis. ABRS, Canberra. pp. 375–400; Brusca, RC, Moore W, Shuster SM (eds) (2016) Chapter 14. Phylum Annelida. In: Invertebrates, 3rd Edition. Sinauer Associates, Sunderland, MA; Schulze A, Boyle MJ, Kawauchi GY (2019) 6.1. Sipuncula In: Purschke G, Böggemann M, Westheide W (Eds), Handbook of Zoology, Annelida, Volume 1: Annelida Basal Groups and Pleistoannelida, Sedentaria I. De Gruyter, Berlin, pp 177–201; Rouse GW, Pleijel, F, Tilic E (2022) Annelida. Oxford University Press, Oxford, UK.

# Sipuncula, Antillesomatidae Kawauchi, Sharma & Giribet, 2012

**Description**. Body segmentation absent. Body shape peanut shaped, regionalization present, regionalization comprising two regions, regions demarcated by presence of unsegmented trunk and a narrower, long anterior proboscis (introvert capable of rolling in and out). Introvert shorter than trunk, hooks absent (in adults) or present (few in small individuals), papillae absent (distally) or present (proximally). Trunk roughened by papillae or rounded skin bodies (dark brown). Body pigmentation absent or present, orange-brown body; tentacles may be banded; epidermis more-or-less smooth, or papillate (introvert often covered with epidermal hooks). Anus positioned near anterior end, dorsally, near the introvert-trunk junction. Pygidium absent. Elongate proboscis present; retractable and tube-like (a large introvert). Discrete head absent (represented by tentacles which are only visible if introvert is fully everted).

Postcephalic eyes absent, or present; epidermal. Buccal tentacles present; arising from one side of mouth (on oral disc; numerous, 30–200 in adults); ciliated. Nuchal organs present; on dorsal side of anteriorly projecting introvert organ. Peristomium not visible.

Gut coiled (descending and ascending branches of the intestine wrap around each other). Foregut without a distinct ventral or axial pharynx. Protrusible proboscis absent. Caeca of foregut absent. Caeca of midgut (=posterior crop caeca) absent. Caeca of hindgut absent, or present.

Chaetae absent.

Segmental organs are metanephridia; several pairs in anterior body. Tube absent.

**Literature used for description**. Edmonds SJ (2000) Chapter 5. Phylum Sipuncula. In Beesley PL, Ross GJB, Glasby CJ (Eds) Fauna of Australia Vol. 4A. Polychaetes & Allies. The Southern Synthesis. ABRS, Canberra. pp. 375–400; Brusca, RC, Moore W, Shuster SM (eds) (2016) Chapter 14. Phylum Annelida. In: Invertebrates, 3rd Edition. Sinauer Associates, Sunderland, MA; Schulze A, Boyle MJ, Kawauchi GY (2019) 6.1. Sipuncula In: Purschke G, Böggemann M, Westheide W (Eds), Handbook of Zoology, Annelida, Volume 1: Annelida Basal Groups and Pleistoannelida, Sedentaria I. De Gruyter, Berlin, pp 177–201; Rouse GW, Pleijel, F, Tilic E (2022) Annelida. Oxford University Press, Oxford, UK.

# Sipuncula, Aspidosiphonidae Quatrefages, 1865

**Description**. Body segmentation absent. Body shape peanut shaped, regionalization present, regionalization comprising two regions, regions demarcated by presence of unsegmented trunk and a narrower, long anterior proboscis (introvert capable of rolling in and out). Introvert longer than trunk, hooks present, papillae absent. Trunk roughened by papillae or rounded skin bodies, anterior extremity of trunk with hardened or calcareous structures present, posterior trunk with shield present. Body pigmentation absent or present; epidermis more-or-less smooth, or papillate (introvert often covered with epidermal hooks). Anus positioned near anterior end, dorsally, near the introvert-trunk junction (surrounded by thickened tissue). Pygidium absent. Elongate proboscis present; retractable and tube-like (a large introvert). Discrete head absent (represented by tentacles which are only visible if introvert is fully everted).

Postcephalic eyes absent, or present; epidermal. Buccal tentacles present; arising from one side of mouth (encircling nuchal organ on oral disc); ciliated. Nuchal organs present; on dorsal side of anteriorly projecting introvert organ. Peristomium not visible.

Gut coiled (descending and ascending branches of the intestine wrap around each other). Foregut without a distinct ventral or axial pharynx. Protrusible proboscis absent. Caeca of foregut absent. Caeca of midgut (=posterior crop caeca) absent. Caeca of hindgut absent, or present.

Chaetae absent.

Segmental organs are metanephridia; several pairs in anterior body. Tube absent.

**Literature used for description**. Edmonds SJ (2000) Chapter 5. Phylum Sipuncula. In Beesley PL, Ross GJB, Glasby CJ (Eds) Fauna of Australia Vol. 4A. Polychaetes & Allies. The Southern Synthesis. ABRS, Canberra. pp. 375–400; Brusca, RC, Moore W, Shuster SM (eds) (2016) Chapter 14. Phylum Annelida. In: Invertebrates, 3rd Edition. Sinauer Associates, Sunderland, MA; Schulze A, Boyle MJ, Kawauchi GY (2019) 6.1. Sipuncula In: Purschke G, Böggemann M, Westheide W (Eds), Handbook of Zoology, Annelida, Volume 1: Annelida Basal Groups and Pleistoannelida, Sedentaria I. De Gruyter, Berlin, pp 177–201; Rouse GW, Pleijel, F, Tilic E (2022) Annelida. Oxford University Press, Oxford, UK.

# Sipuncula, Golfingiidae Stephen & Edmonds, 1972

**Description**. Body segmentation absent. Body shape peanut shaped, regionalization present, regionalization comprising two regions, regions demarcated by presence of unsegmented trunk and a narrower, long anterior proboscis (introvert capable of rolling in and out). Introvert shorter than trunk or about equal to trunk (trunk no longer than 20 cm), hooks absent or present (simple and not sharply curved when present), papillae absent. Trunk roughened by papillae or rounded skin bodies or smooth (small), anterior extremity of trunk with hardened or calcareous structures absent, posterior trunk with shield absent. Body translucent, gut visible or opaque, gut usually not visible, pigmentation absent or present; epidermis more-or-less smooth, or papillate (introvert often covered with epidermal hooks). Anus positioned near anterior end, dorsally, near the introvert-trunk junction. Pygidium absent. Elongate proboscis present; retractable and tube-like (a large introvert). Discrete head absent (represented by tentacles which are only visible if introvert is fully everted).

Postcephalic eyes absent, or present; epidermal. Buccal tentacles present; surrounding perimeter of mouth (on oral disc; tentacles highly variable in shape, sometimes elaborately branched); ciliated. Nuchal organs present; on dorsal side of anteriorly projecting introvert organ. Peristomium not visible.

Gut coiled (descending and ascending branches of the intestine wrap around each other). Foregut without a distinct ventral or axial pharynx. Protrusible proboscis absent. Caeca of foregut absent. Caeca of midgut (=posterior crop caeca) absent. Caeca of hindgut absent, or present.

Chaetae absent.

# Segmental organs are metanephridia; several pairs in anterior body.

# Tube absent.

**Literature used for description**. Edmonds SJ (2000) Chapter 5. Phylum Sipuncula. In Beesley PL, Ross GJB, Glasby CJ (Eds) Fauna of Australia Vol. 4A. Polychaetes & Allies. The Southern Synthesis. ABRS, Canberra. pp. 375–400; Brusca, RC, Moore W, Shuster SM (eds) (2016) Chapter 14. Phylum Annelida. In: Invertebrates, 3rd Edition. Sinauer Associates, Sunderland, MA; Schulze A, Boyle MJ, Kawauchi GY (2019) 6.1. Sipuncula In: Purschke G, Böggemann M, Westheide W (Eds), Handbook of Zoology, Annelida, Volume 1: Annelida Basal Groups and Pleistoannelida, Sedentaria I. De Gruyter, Berlin, pp 177–201; Rouse GW, Pleijel, F, Tilic E (2022) Annelida. Oxford University Press, Oxford, UK.

# Sipuncula, Phascolosomatidae Stephen & Edmonds, 1972

**Description**. Body segmentation absent. Body shape peanut shaped, regionalization present, regionalization comprising two regions, regions demarcated by presence of unsegmented trunk and a narrower, long anterior proboscis (introvert capable of rolling in and out). Introvert about equal to trunk or longer than trunk, hooks present, papillae absent. Trunk roughened by papillae or rounded skin bodies (more concentrated at anterior and posterior ends), anterior extremity of trunk with hardened or calcareous structures present (horny, hardened or calcareous shield), posterior trunk with shield present (horny, hardened or calcareous shield). Body pigmentation absent or present, introvert may be banded; epidermis more-or-less smooth, or papillate (introvert often covered with epidermal hooks). Anus positioned near anterior end, dorsally, near the introvert-trunk junction. Pygidium absent. Elongate proboscis present; retractable and tube-like (a large introvert). Discrete head absent (represented by tentacles which are only visible if introvert is fully everted).

Postcephalic eyes absent, or present; epidermal. Buccal tentacles present; arising from one side of mouth (short tentacles surrounding nuchal organ in an arc on oral disc); ciliated. Nuchal organs present; on dorsal side of anteriorly projecting introvert organ. Peristomium not visible.

Gut coiled (descending and ascending branches of the intestine wrap around each other). Foregut without a distinct ventral or axial pharynx. Protrusible proboscis absent. Caeca of foregut absent. Caeca of midgut (=posterior crop caeca) absent. Caeca of hindgut absent, or present.

Chaetae absent.

Segmental organs are metanephridia; several pairs in anterior body. Tube absent.

**Literature used for description**. Edmonds SJ (2000) Chapter 5. Phylum Sipuncula. In Beesley PL, Ross GJB, Glasby CJ (Eds) Fauna of Australia Vol. 4A. Polychaetes & Allies. The Southern Synthesis. ABRS, Canberra. pp. 375–400; Brusca, RC, Moore W, Shuster SM (eds) (2016) Chapter 14. Phylum Annelida. In: Invertebrates, 3rd Edition. Sinauer Associates, Sunderland, MA; Schulze A, Boyle MJ, Kawauchi GY (2019) 6.1. Sipuncula In: Purschke G, Böggemann M, Westheide W (Eds), Handbook of Zoology, Annelida, Volume 1: Annelida Basal Groups and Pleistoannelida, Sedentaria I. De Gruyter, Berlin, pp 177–201; Rouse GW, Pleijel, F, Tilic E (2022) Annelida. Oxford University Press, Oxford, UK.

# Sipuncula, Siphonosomatidae Kuwauchi et al., 2012

**Description**. Body segmentation absent. Body shape peanut shaped, regionalization present, regionalization comprising two regions, regions demarcated by presence of unsegmented trunk and a narrower, long anterior proboscis (introvert). Introvert shorter than trunk, hooks absent or present (when present arranged in rings), papillae present (conical shaped). Trunk smooth. Body pigmentation absent or present; epidermis more-or-less smooth, or papillate (introvert often covered with epidermal hooks). Anus positioned near anterior end, dorsally, near the introvert-trunk junction. Pygidium absent. Elongate proboscis present; retractable and tube-like (a large introvert). Discrete head absent (represented by tentacles which are only visible if introvert is fully everted). Postcephalic eyes absent, or present; epidermal. Buccal tentacles present; surrounding perimeter of mouth (on oral disc); ciliated. Nuchal organs present; on dorsal side of anteriorly projecting introvert organ. Peristomium not visible.

Gut coiled (descending and ascending branches of the intestine wrap around each other). Foregut without a distinct ventral or axial pharynx. Protrusible proboscis absent. Caeca of foregut absent. Caeca of midgut (=posterior crop caeca) absent. Caeca of hindgut absent, or present.

Chaetae absent.

Segmental organs are metanephridia; several pairs in anterior body. Tube absent.

**Literature used for description**. Edmonds SJ (2000) Chapter 5. Phylum Sipuncula. In Beesley PL, Ross GJB, Glasby CJ (Eds) Fauna of Australia Vol. 4A. Polychaetes & Allies. The Southern Synthesis. ABRS, Canberra. pp. 375–400; Brusca, RC, Moore W, Shuster SM (eds) (2016) Chapter 14. Phylum Annelida. In: Invertebrates, 3rd Edition. Sinauer Associates, Sunderland, MA; Schulze A, Boyle MJ, Kawauchi GY (2019) 6.1. Sipuncula In: Purschke G, Böggemann M, Westheide W (Eds), Handbook of Zoology, Annelida, Volume 1: Annelida Basal Groups and Pleistoannelida, Sedentaria I. De Gruyter, Berlin, pp 177–201; Rouse GW, Pleijel, F, Tilic E (2022) Annelida. Oxford University Press, Oxford, UK.

# Sipuncula, Sipunculidae Rafinesque, 1814

**Description**. Body segmentation absent. Body shape peanut shaped, regionalization present, regionalization comprising two regions, regions demarcated by presence of unsegmented trunk and a narrower, long anterior proboscis (introvert). Introvert shorter than trunk, hooks absent, papillae present (triangular shaped, arranged irregularly). Trunk roughened by papillae or rounded skin bodies, anterior extremity of trunk with hardened or calcareous structures present (horny, hardened or calcareous shield), posterior trunk with shield present (horny, hardened or calcareous shield). Body translucent, gut visible or opaque, gut usually not visible, pigmentation absent or present; epidermis more-or-less smooth, or papillate (introvert often covered with epidermal hooks and subtriangular papillae; trunk covered with rectangular pillow-like papillae). Anus positioned near anterior end, dorsally, near the introvert-trunk junction. Pygidium absent. Elongate proboscis present; retractable and tube-like (a large introvert). Discrete head absent (represented by tentacles which are only visible if introvert is fully everted).

Postcephalic eyes absent, or present; epidermal. Buccal tentacles present; surrounding perimeter of mouth (on oral disc); ciliated. Nuchal organs present; on dorsal side of anteriorly projecting introvert organ. Peristomium not visible.

Gut coiled (descending and ascending branches of the intestine wrap around each other). Foregut without a distinct ventral or axial pharynx. Protrusible proboscis absent. Caeca of foregut absent. Caeca of midgut (=posterior crop caeca) absent. Caeca of hindgut absent, or present.

Chaetae absent.

Segmental organs are metanephridia; several pairs in anterior body. Tube absent.

**Literature used for description**. Edmonds SJ (2000) Chapter 5. Phylum Sipuncula. In Beesley PL, Ross GJB, Glasby CJ (Eds) Fauna of Australia Vol. 4A. Polychaetes & Allies. The Southern Synthesis. ABRS, Canberra. pp. 375–400; Brusca, RC, Moore W, Shuster SM (eds) (2016) Chapter 14. Phylum Annelida. In: Invertebrates, 3rd Edition. Sinauer Associates, Sunderland, MA; Schulze A, Boyle MJ, Kawauchi GY (2019) 6.1. Sipuncula In Purschke G, Böggemann M, Westheide W (Eds), Handbook of Zoology, Annelida, Volume 1: Annelida Basal Groups and Pleistoannelida, Sedentaria I. De Gruyter, Berlin, pp 177–201; Rouse GW, Pleijel, F, Tilic E (2022) Annelida. Oxford University Press, Oxford, UK.

# Sparganophilidae Michaelsen, 1921

**Description**. Body segment number: variable. Body shape elongate, more-or-less equal width over entire length. Dorsal intersegmental furrow present (?), pores on mid-dorsal line present (?). Pygidium simple lobe. Pygidial appendages absent.

Prostomium bluntly conical.

Gut straight with side branches. Foregut a muscular dorsal pharynx. Gizzard absent or present (in *Komarkiona eatoni*), in segment VI. Calciferous glands absent. Intestinal typhlosole absent. Heart bodies present, segments VII-XI.

Chaetae first appear on first segment after peristomium or second segment after peristomium (=S2 or S3, respectively for oligochaete workers), in lumbricine arrangement - paired upper and lower bundles of one or a few chaetae each - widely spaced lateral and ventrolateral pairs. Crotchet chaetae present, simple-pointed. Genital chaetae absent.

Sexual reproduction hermaphroditic. Clitellum thick, multilayered (often long, extending over many segments), partially encircles body, in region of male pores (covering male pores at least with its anterior edge) or posterior to male pores, posterior to female pores. Clitellum occurs from XV to XIX, usually but ranging from XIV to XXVII. Tubercula pubertatis present (pubertal ridges bearing male pores). Gonadal segments bearing genital papillae absent, or present. Testes present, two pairs in total, one pair per segment; present in segments X and XI. Ovaries present, one pair; present in segment XIII. Egg sacs present. Segmental organs are metanephridia; both nephridial pores and gonoducts located around clitellum. Spermathecae present, pre-testicular. Spermathecal pores present (inconspicuous; often several pairs), paired (dorsolateral in *Komarekiona eatoni*), located within 1 or 2 segments of male pores or located well anterior to male pores; 2–3 pairs (usually); in segment VI-IX (V-VI sometimes). Female gonoduct present (inconspicuous). In segment XIV. Male gonoducts present, with atrium present. Male pores one pair, in segment XIX, XX or later, opisthoporous (two or more segments following testicular segment). Prostate gland absent (or not obvious) or present, tubular, more than one pair. Prostate pores in segments IX to XI most obvious, but smaller ones on either side (for *Komarekiona eatoni*).

**Literature used for description**. Jamieson, BGM (2006) Non-leech Clitellata (with contributions by Marco Ferraguti). Pp. 235–392. In Reproductive Biology and Phylogeny of Annelida. Series Editor BGM Jamieson. Volume 4. Editors G Rouse, F Pleijel. Science Publishers, Enfield, New Hampshire; Timm, T (2009) A guide to the freshwater Oligochaeta and Polychaeta of Northern and Central Europe. Lauterbornia 66: 1–235; Thorp JH, Lovell LL, Timm T, Martin P, Gelder SR, Govedich FR, Moser WE, Nakano T, Bielecki A, Bain BA, Utevsky S, Gil J, Glasby CJ, Martin D (2019) Phylum Annelida. In: Rogers DC, Thorp JH (Eds) Keys to Palaearctic Fauna: Thorp and Covich’s Freshwater invertebrates - Volume IV. Academic Press, Elsevier, pp. 357–518.

# Sphaerodoridae Malmgren, 1867

**Description**. Body segment number: variable. Body shape elongate, more-or-less equal width over entire length or sausage or grub-shaped. Body translucent, gut visible or opaque, gut usually not visible; epidermis papillate, or tuberculate, with bumps of various sizes and arrangements; tuberculae arrangement in transverse rows, or in longitudinal rows (dorsal macrotubercles and ventral microtubercles; papillae are also present dorsally and ventrally). Pygidium simple lobe. Pygidial appendages present, one pair of cirri and single medial papilla (cirri may be inflated). Discrete head present; complex in shape bearing appendages.

Prostomium rounded to oval (very small), eyes on head absent or present, two pairs (anterior and posterior pairs may be fused); head eyes simple pigmented cups. Prostomial antennae present; median and paired laterals; unarticulated. Palps present (may resemble antennae); anteroventral; tapering (usually) sensory type; unarticulated. Nuchal organs present; paired low projections from posterolateral prostomium; indistinct dorsolateral ciliated patches. Peristomium not visible.

Gut more-or-less straight, lacking side branches. Foregut a muscular axial pharynx.

Protrusible proboscis present, distal ring of papillae present. Pharynx proventricle present (?).

First segment tentaculate; second segment chaetous. Tentacular cirri present; 1 pair(s); arise on a single segment; internal aciculae absent. First chaetiger with neurochaetae only; parapodia similar in length or slightly shorter than subsequent parapodia; parapodia more-or-less laterally directed and free from head; chaetae similar in orientation, length and thickness to other chaetae (although one genus, *Sphaerodorum*, has stout, recurved hooks in this chaetiger). Parapodia uniramous. Notopodial lobes absent (though dorsal cirri are present). Neuropodial lobes represented by at least one chaetal lobe. Dorsal cirri present; more-or-less cirriform. Ventral cirri present; cirriform or tapering.

Chaetae first appear on second segment after peristomium, arranged as a single bundle (or row) of many chaetae (lower bundle - neurochaetae - only). Aciculae present; in ventral position (=neuroaciculae). Neuroaciculae distally tapering. Capillary chaetae absent. Spines absent, or present; slightly curved and more-or-less smooth, or with small teeth or spinelets, or sharply bent (=geniculate) or recurved (recurved spines rare, only in chaetiger 1 of one genus); present only in one or a few anterior chaetigers, or present in most or all chaetigers; in ventral (neuropodial) position only. Hooks absent. Compound chaetae absent, or present; in ventral (neuropodial) position; appendage distally curved (=falcate; ie., one distal tooth); appendage not canaliculated; appendage without hoods or guards; joint, appearance distinctly asymmetrical (=heterogomph); joint effected by ligament(s) (double ligaments); shaft tapering slightly or evenly thick from emergence to joint; shaft, internally solid, without distinct core. Compound chaetae present in most or all chaetigers.

Segmental organs are protonephridia; present in most segments of the body. Tube absent.

**Literature used for description**. Fauchald K (1974). Sphaerodoridae (Polychaeta: Errantia) from world-wide areas. Journal of Natural History 8: 257–289; Glasby CJ, Fauchald K (2003). POLiKEY. An information system for polychaete families and higher taxa. Version 2, 5 June 2003. Australian Biological Resources Study, DCCEEW, Canberra, https:[//www.dcceew.gov.au/;](http://www.dcceew.gov.au/%3B) Capa M, Bakken T, Meißner K, Nygren A (2018) Three, two, one! Revision of the long-bodied sphaerodorids (Sphaerodoridae, Annelida) and synonymization of *Ephesiella*, *Ephesiopsis* and *Sphaerodorum*. PeerJ 6(1): e5783. https://doi.org/10.7717/peerj.5783; Capa M, Nygren A, Parapar J, Bakken T, Meißner K, Moreira J (2019) Systematic re-structure and new species of Sphaerodoridae (Annelida) after morphological revision and molecular phylogenetic analyses of the North East Atlantic fauna. ZooKeys 845: 1–97. https://doi.org/10.3897/zookeys.845.32428; Capa M, Bakken T, Purschke G (2022) 7.13.14 Sphaerodoridae Malmgren, 1867 In: Purschke G, Böggemann, M, Westheide W (Eds) Handbook of Zoology. Annelida. Vol. 4: Pleistoannelida, Errantia II. De Gruyter, Berlin, 413–438; Rouse GW, Pleijel F, Tilic E (2022) Annelida. Oxford University Press, Oxford, UK.

# Spintheridae Augener, 1913

**Description**. Body segment number: variable. Body shape ovate to elliptical, dorsoventrally flattened. Body pigmentation present, often brightly coloured to appear cryptic on their hosts; epidermis with radial or transverse dorsal ridges (formed by the notopodia). Pygidium simple lobe. Pygidial appendages present, one pair of cirri. Discrete head present; complex in shape bearing appendages (carrying only a minute median antenna).

Prostomium rounded to oval, eyes on head absent or present, two pairs; head eyes simple pigmented cups. Prostomial antennae present; median one only; unarticulated. Peristomium not visible.

Gut straight with side branches (many segmentally arranged diverticulae). Foregut a muscular ventral pharynx. Protrusible proboscis present (tongue-like), distal ring of papillae absent.

First segment achaetous; second segment chaetous. First chaetiger with both notochaetae and neurochaetae; parapodia similar in length or slightly shorter than subsequent parapodia; parapodia anteriorly directed and wrapping around head; chaetae similar in orientation, length and thickness to other chaetae. Parapodia biramous; parapodial lobes prominent. Notopodial lobes long dorsal ridges. Neuropodial lobes represented by at least one chaetal lobe.

Chaetae first appear on second segment after peristomium, arranged in paired bundles (or rows) of many chaetae. Aciculae present; in ventral position (=neuroaciculae). Capillary chaetae absent. Spines present; slightly curved and more-or-less smooth (bifid or simple distally); present in most or all chaetigers; in dorsal (notopodial) position only (internal and external). Hooks absent. Compound chaetae present; in ventral (neuropodial) position; appendage distally curved (=falcate; ie., one distal tooth); appendage not canaliculated; appendage without hoods or guards; joint, appearance more-or-less symmetrical (=homogomph; includes also hemigomph and sesquigomph); joint effected by fold in external sheath of chaeta; shaft tapering slightly or evenly thick from emergence to joint; shaft, internally solid, without distinct core. Compound chaetae present in most or all chaetigers.

Tube absent.

**Literature used for description**. Glasby CJ, Fauchald K (2003). POLiKEY. An information system for polychaete families and higher taxa. Version 2, 5 June 2003. Australian Biological Resources Study, DCCEEW, Canberra, https:[//www.dcceew.gov.au/;](http://www.dcceew.gov.au/%3B) Böggemann M (2019) 7.2.2 Spintheridae Augener, 1913 In Purschke G, Böggemann M, Westheide W (Eds), Handbook of Zoology, Annelida, Volume 1: Annelida Basal Groups and Pleistoannelida, Sedentaria I. De Gruyter, Berlin, 273–280; Rouse GW, Pleijel F, Tilic E (2022) Annelida. Oxford University Press, Oxford, UK.

# Spionidae Grube, 1850

**Description**. Body segment number: variable. Body shape elongate, more-or-less equal width over entire length. Body pigmentation absent or present, body rarely pigmented but palps may be banded; epidermis more-or-less smooth. Pygidium simple lobe, or with multiple digitate lobes. Pygidial appendages present, more than four cirri. Discrete head present; complex in shape bearing appendages.

Prostomium conical, tapering to slender tip (rarely) or triangular to trapezoidal (narrow end posteriorly) or t-shaped, wide end anteriorly (rarely), eyes on head absent or present, one pair or numerous, unpaired; head eyes simple pigmented cups. Prostomial antennae absent, or present; median one only (also called an occipital antenna; very small); unarticulated. Palps present; anterodorsal; grooved (usually) feeding type (groove is rarely absent, for example *Scolelepis*); feeding palp longitudinally grooved. Caruncle absent, or present. Nuchal organs present; single antenna-like projection from posterior prostomium (as an occipital antenna in many species). Peristomium visible. Peristomium a single ring, collar-like (collar surrounds most of the prostomium; may be wing-like).

Foregut a non-muscular axial pharynx or a muscular ventral pharynx. Pharynx dorsolateral ciliated folds present. Heart bodies present (based on observations on *Sreblospio* only).

First segment chaetous. First chaetiger with neurochaetae only, or with both notochaetae and neurochaetae; parapodia similar in length or slightly shorter than subsequent parapodia; parapodia more-or-less laterally directed and free from head (rarely projecting anteriorly); chaetae similar in orientation, length and thickness to other chaetae. Parapodia biramous; parapodial lobes prominent. Notopodial lobes represented by at least one chaetal lobe. Neuropodial lobes represented by at least one chaetal lobe. Lateral organs present. Branchiae present (rarely absent), arise from lateral body (fused basally to notopodium) or arise from dorsal body, occur first few segments or occur on mid-body segments or occur near posterior end; lateral branchiae digitiform (may be smooth or pinnate). Dorsal branchiae multiple filaments arising from a central stalk, or simple filaments each arising directly from body wall (flattened).

Chaetae first appear on first segment after peristomium or second segment after peristomium, arranged in paired bundles (or rows) of many chaetae. Aciculae absent. Capillary chaetae present, in dorsal (notopodial) position or in ventral (neuropodial) position, distally tapered to a point, edge smooth. Capillary chaetae, externally not pseudo-segmented. Capillary chaetae, internally not chambered or hollow. Spines absent, or present; slightly curved and more-or-less smooth (those in chaetiger 5 of the polydorid genera are robust and may be ornate distally); present in most or all chaetigers, or present only in one or a few anterior chaetigers (chaetiger 5); in both dorsal and ventral positions. Hooks present; without distal hood, beard or ligament, or with a distal hood; occur in dorsal (notopodial) position (rarely), or occur in ventral (neuropodial) position; occur over entire body.

Segmental organs are metanephridia; metanephridia along most of the body; anterior ones for excretion, posterior ones for gamete release;

Tube absent, or present; membranous.

**Literature used for description**. Glasby CJ, Fauchald K (2003). POLiKEY. An information system for polychaete families and higher taxa. Version 2, 5 June 2003. Australian Biological Resources Study, DCCEEW, Canberra, https:[//www.dcceew.gov.au/;](http://www.dcceew.gov.au/%3B) Blake JA, Maciolek NJ, Meißner K (2019) 7.4.1 Spionidae Grube, 1850 In: Purschke G, Böggemann M, Westheide W (Eds), Handbook of Zoology, Annelida, Volume 2: Pleistoannelida, Sedentaria II. De Gruyter, Berlin, 1–103; Rouse GW, Pleijel F, Tilic E (2022) Annelida. Oxford University Press, Oxford, UK.

# Sternaspidae Carus, 1863

**Description**. Body segment number: variable. Body shape peanut shaped, regionalization present (Sternaspidae can appear to be bipartite as the first few segments can be retracted into the second region), regionalization comprising three regions, regions demarcated by structural differences in parapodia over body. Body pigmentation absent; epidermis papillate (over entire body; size of papillae depends on degree of contraction of specimen; sediment often adheres to papillae). Ventrocaudal shield on posterior segments present (ranges from yellow-red-black in colour and covers the entire ventral surface of the posterior-most body region; the most distinctive feature of the family). Pygidium simple lobe. Pygidial appendages absent. Discrete head present (very small and may not be visible if anterior end is in retracted state); lobe-like without appendages; head retractable into anterior segments.

Prostomium bluntly conical, eyes on head absent or present; head eyes simple pigmented cups. Palps absent (possibly rarely present as seen in *Sternaspis spinosus*). Nuchal organs absent. Peristomium a single ring.

Gut straight except for a large midbody loop. Foregut a muscular axial pharynx. Protrusible proboscis present, with papillae in subterminal position.

First segment chaetous; second segment chaetous. First chaetiger with neurochaetae only; parapodia similar in length or slightly shorter than subsequent parapodia; parapodia anteriorly directed and wrapping around head (when prostomium retracted), or more-or-less laterally directed and free from head. Parapodia absent, or present (absent in first two regions, present in posterior region). Parapodia uniramous (small conical lobes that may be either notopodial or neuropodial). Branchiae present, arise from dorsal body, occur near posterior end. Dorsal branchiae simple filaments each arising directly from body wall (filiform, retractile).

Chaetae first appear on first segment after peristomium, arranged as a single bundle (or row) of many chaetae (lower bundle - neurochaetae - only). Aciculae absent. Capillary chaetae present, in dorsal (notopodial) position (capiilaries of the mid- and posterior regions are probably notopodial, but there is some doubt), distally tapered to a point, edge smooth or hirsute-serrate. Capillary chaetae, externally not pseudo-segmented. Capillary chaetae, internally not chambered or hollow. Spines present; slightly curved and more-or-less smooth; present only in one or a few anterior chaetigers (pseudocompound); in ventral (neuropodial) position only (uncertain). Hooks absent.

Reproduction sexual (gonochoric).

Segmental organs are metanephridia; metanephridia single anterior pair of excretory metanephridia and several more posterior ones for gamete release (single pair of excretory ones exit between segments 4 and 5; single pair of gonopores behind exit through the elongate cylindrical genital papillae on segment 6).

Tube absent.

**Literature used for description**. Glasby CJ, Fauchald K (2003). POLiKEY. An information system for polychaete families and higher taxa. Version 2, 5 June 2003. Australian Biological Resources Study, DCCEEW, Canberra, https:[//www.dcceew.gov.au/;](http://www.dcceew.gov.au/%3B) Fiege D (2019) 7.3.1.8 Sternaspidae Carus, 1863 In: Purschke G, Böggemann M, Westheide W (Eds) Handbook of Zoology, Annelida, Volume 1: Annelida Basal Groups and Pleistoannelida, Sedentaria I. De Gruyter, Berlin, 440–456; Rouse GW, Pleijel F, Tilic E (2022) Annelida. Oxford University Press, Oxford, University Press, Oxford, UK.

# Syllidae Grube, 1850

**Description**. Body segment number: variable. Body shape elongate, more-or-less equal width over entire length or sausage or grub-shaped, dorsoventrally flattened or more-or-less cylindrical. Body translucent, gut visible or opaque, gut usually not visible, pigmentation absent or present, pigmentation dorsum and/or dorsal cirri are often brightly coloured and patterned (e.g., transverse stripes); epidermis more-or-less smooth, or papillate, or tuberculate, with bumps of various sizes and arrangements (rarely). Bioluminescence absent, or present (linked to swarming and spawning). Pygidium simple lobe. Pygidial appendages present, one pair of cirri or one pair of cirri and single medial papilla. Discrete head present; complex in shape bearing appendages.

Prostomium pentagonal to quadrangular or rounded to oval, eyes on head present, two pairs or three pairs (posterior third pair usually minute ocular specks); head eyes simple pigmented cups, or compound, with lenses (simple eyespots, when present, the more anterior). Prostomial antennae present; median and paired laterals (median one rarely absent); unarticulated, or articulated. Palps present (may be reduced or fused with each other); anteroventral; tapering (usually) sensory type (triangular shaped); unarticulated. Nuchal organs present; paired low projections from posterolateral prostomium; indistinct dorsolateral ciliated patches, or posterior projections (referred to as nuchal epaulettes). Peristomium not visible.

Gut more-or-less straight, lacking side branches (often a pair of small caeca following the proventricle and ventricle). Foregut a muscular axial pharynx (with a chitinous lining; may be straight or coiled (also called a pharyngeal tube)). Pharynx jaws absent (the fused teeth in some taxa may resemble jaws). Pharynx teeth absent or present, as a single tooth (mediodorsal) or present, as a trepan (fused teeth). Protrusible proboscis present, smooth, distal ring of papillae present. Pharynx proventricle present (a barrel-shaped organ, visible through the body wall, with distinctive radial muscular fibre bands).

First segment tentaculate (=elongated dorsal and ventral cirri; rarely absent); second segment chaetous. Tentacular cirri present (rarely absent); 1–2 pair(s); arise on a single segment; internal aciculae absent. First chaetiger with neurochaetae only; parapodia similar in length or slightly shorter than subsequent parapodia; parapodia more-or-less laterally directed and free from head; chaetae similar in orientation, length and thickness to other chaetae. Parapodia uniramous. Notopodial lobes absent (though dorsal cirri, and sometimes notochaetae, are present). Neuropodial lobes represented by at least one chaetal lobe. Dorsal cirri present; more-or-less cirriform (smooth or distinctly annulated, with anterior ones exhibiting a specific length/orientation pattern). Ventral cirri absent (in *Autolytinae*), or present; cirriform or tapering. Branchiae absent (although branchiae-like structures are rarely present).

Chaetae first appear on second segment after peristomium, arranged as a single bundle (or row) of many chaetae (lower bundle - neurochaetae - only, except in reproductive forms in which simple notochaetae may appear). Aciculae present; in ventral position (=neuroaciculae). Neuroaciculae distally tapering (rarely bent or knob-tipped). Capillary chaetae present, in dorsal (notopodial) position (only in reproductive forms) or in ventral (neuropodial) position, distally tapered to a point (rarely bifid), edge smooth. Capillary chaetae, externally not pseudo-segmented. Capillary chaetae, internally not chambered or hollow. Spines absent, or present; slightly curved and more-or-less smooth (may be bifid distally); present in most or all chaetigers; in ventral (neuropodial) position only. Genital chaetae absent, or present (rarely, as modified aciculae of a single chaetiger); hooks absent. Compound chaetae absent, or present; in ventral (neuropodial) position; appendage distally tapering to slender tips (=spinigerous), or distally curved (=falcate; ie., one distal tooth), or multidentate; appendage not canaliculated; appendage without hoods or guards; joint, appearance distinctly asymmetrical (=heterogomph); joint effected by ligament(s); shaft tapering slightly or evenly thick from emergence to joint; shaft, internally solid, without distinct core. Compound chaetae present in most or all chaetigers.

Reproduction sexual (asexual reproduction is rare).

Sexual reproduction hermaphroditic, or gonochoric (both simulataneous and sequential hemaphroditism have been reported; gonochoric forms often transform their parapodia and eyes dramatically prior to reproduction). Segmental organs are protonephridia, or metanephridia.

Tube absent.

**Literature used for description**. Glasby CJ, Fauchald K (2003). POLiKEY. An information system for polychaete families and higher taxa. Version 2, 5 June 2003. Australian Biological Resources Study, DCCEEW, Canberra, https:[//www.dcceew.gov.au/;](http://www.dcceew.gov.au/%3B) San Martin G, Aguado MT (2022) 7.13.2. Syllidae Grube, 1850, In: Purschke G, Böggemann M, Westheide W (Eds), Handbook of Zoology, Annelida, Volume 3: Pleistoannelida, Errantia II. De Gruyter, Berlin, 152–227; Rouse GW, Pleijel F, Tilic E (2022) Annelida. Oxford University Press, Oxford, University Press, Oxford, UK.

# Syngenodrilidae Smith & Green, 1919

**Description**. Body segment number: variable. Secondary annulation absent or present. Body shape elongate, more-or-less equal width over entire length. Pygidium simple lobe. Pygidial appendages absent.

Prostomium bluntly conical.

Foregut a muscular dorsal pharynx. Gizzard present, in segments VIII and IX. Heart bodies present, segments VI-XI.

Chaetae first appear on first segment after peristomium (=S2 for oligochaete workers). Capillary (=hair) chaetae absent. Crotchet chaetae present (very small). Genital chaetae absent, or present.

# Sexual reproduction hermaphroditic. Clitellum thin, single layered, in region of male and female pores. Clitellum occurs from XI to XXVII. Tubercula pubertatis present, paired ridges on ventrolateral margins of clitellum. Gonadal segments bearing genital papillae absent. Testes present, two pairs in total, one pair per segment (testis sac present); present in segments X and XI. Sperm sac present. Ovaries present, one pair; present in segment XIII. Segmental organs are metanephridia; both nephridial pores and gonoducts located around clitellum. Spermathecae present. Spermathecal pores present, located well anterior to male pores; 2 pairs; in segment VIII-IX. Female gonoduct present. Female pores one pair, in segment XIV. Male gonoducts present. Male pores one pair (lateral), in segment XIII, opisthoporous (two or more segments following testicular segment). Prostate gland present, tubular, 3 pairs. Prostate pores in segments XI to XIII; prostate pore and male pore on segment XVII not united, separate pores.

**Literature used for description**. Smith F, Green BR (1919). Descriptions of new African earthworms: including a new genus of Moniligastridae. Proceedings U.S. National Museum 55(2263): 145–166; Jamieson BGM (1978). Phylogenetic and phenetic systematics of the opisthoporous Oligochaeta. Evolutionary Theory 3: 195–233; Jamieson, BGM (2006) Non-leech Clitellata (with contributions by Marco Ferraguti). Pp. 235–392. In Reproductive Biology and Phylogeny of Annelida. Series Editor BGM Jamieson. Volume 4. Editors G Rouse, F Pleijel. Science Publishers, Enfield, New Hampshire.

# Terebellidae Johnston, 1846, sensu lato

**Description**. Body segment number: variable. Body shape widest anteriorly and tapering posteriorly, regionalization present (rarely absent), regionalization comprising two regions, regions demarcated by absence of abdominal notopodia. Body pigmentation absent or present, bands or spots on the tentacles and other parts of the body; epidermis more-or-less smooth, or thick and rugose (anterodorsally). Bioluminescence absent, or present. Thoracic ventral glandular areas present; distinct mid-ventral shield-shaped swellings. Pygidium simple lobe, or with multiple digitate lobes (rarely; rim may have multiple papillae). Pygidial appendages absent. Discrete head absent (ridge-like area at base of tentacular membrane).

Eyes on head absent or present, numerous, unpaired; head eyes simple pigmented cups. Buccal tentacles present; arising from one side of mouth (but not retractible into mouth); grooved. Nuchal organs absent, or present; paired low projections from posterolateral prostomium; indistinct dorsolateral ciliated patches. Peristomium visible. Peristomium expanded into well-developed upper and lower lips.

Gut straight except for a large midbody loop. Foregut a muscular ventral pharynx. Pharynx dorsolateral ciliated folds absent or present. Heart bodies present.

First segment achaetous (may bear lateral lobes); second segment achaetous (may bear lateral lobes or dorsal branchiae). First chaetiger with notochaetae only; parapodia similar in length or slightly shorter than subsequent parapodia; parapodia more-or-less laterally directed and free from head; chaetae similar in orientation, length and thickness to other chaetae. Parapodia present, or absent (very rarely). Parapodia biramous (thorax), or uniramous (abdomen); parapodial lobes prominent. Notopodial lobes represented by at least one chaetal lobe (maybe absent posteriorly). Neuropodial lobes represented by at least one chaetal lobe, or low ridges (tori). Lateral organs present. Branchiae absent or present, arise from dorsal body, occur first few segments. Dorsal branchiae branching, or multiple filaments arising from a central stalk, or simple filaments each arising directly from body wall.

Chaetae present (very rarely absent), first appear on third segment after peristomium or fourth segment after peristomium, arranged in paired bundles (or rows) of many chaetae. Aciculae absent. Capillary chaetae present (rarely absent), in dorsal (notopodial) position, distally tapered to a point or sharply bent, edge smooth or corn-eared or hirsute-serrate. Capillary chaetae, externally not pseudo-segmented. Capillary chaetae, internally not chambered or hollow. Spines absent, or present (rarely, *Amaeana* only); slightly curved and more-or-less smooth; present in most or all chaetigers (mid and posterior body); in ventral (neuropodial) position only. Hooks absent (rarely present, eg., *Pista indica*). Uncini present, or absent (rarely, some polycirrines); with teeth arranged in transverse series above an enlarged main fang (=crested), or with teeth in vertical series, teeth usually similar-sized (=pectinate) (rarely); in ventral (neuropodial) position throughout; arranged in one row, or arranged in two rows.

Reproduction sexual (gonochoric). Segmental organs are metanephridia; metanephridia several pairs metanephridia anteriorly for excretion, posterior ones for gamete release (4–7 pairs; rarely 0–1 pairs excretory nephridia); several pairs in anterior body.

Tube absent, or present; membranous, or leathery or parchment like.

**Literature used for description**. Glasby CJ, Fauchald K (2003). POLiKEY. An information system for polychaete families and higher taxa. Version 2, 5 June 2003. Australian Biological Resources Study, DCCEEW, Canberra, https:[//www.dcceew.gov.au/;](http://www.dcceew.gov.au/%3B) Stiller J, Tilic E, Rousset V, Pleijel F and Rouse GW (2020). Spaghetti to a Tree: A Robust Phylogeny for Terebelliformia (Annelida) Based on Transcriptomes, Molecular and Morphological Data. Biology 9, 73; doi:10.3390/biology9040073; Hutchings P, Carrerette O, Nogueira JMM, Hourdez S, Lavesque N (2021a) TheTerebelliformia-Recent Developments, Future Directions. Diversity 13: 60. https://doi.org/10.3390/d13020060 [accessed Sep 13 2024]; Hutchings P, Nogueira JM de M, Carrerette O (2021b) 7.7.3 Terebellidae s.l.: Polycirridae Malmgren, 1866, Terebellidae Johnston, 1846, Thelepodidae Hessle, 1917, Trichobranchidae Malmgren, 1866, and Telothelepodidae Nogueira, Fitzhugh & Hutchings, 2013 In: Purschke G, Böggemann M, Westheide W (Eds), Handbook of Zoology, Annelida, Volume 3: Pleistoannelida, Sedentaria III and Errantia I. De Gruyter, Berlin, 68–144; Rouse GW, Pleijel F, Tilic E (2022) Annelida. Oxford University Press, Oxford, UK.

# Terebellidae, Terebellinae, Polycirrini Malmgren, 1866

**Description**. Body segment number: variable. Body shape widest anteriorly and tapering posteriorly, regionalization present, regionalization comprising two regions, regions demarcated by absence of abdominal notopodia. Body translucent, gut visible (*Hauchiella and Polycirrus*) or opaque, gut usually not visible, pigmentation absent or present, pigmentation bands or spots on the tentacles and other parts of the body; epidermis more-or-less smooth, or thick and rugose (anterodorsally). Bioluminescence absent, or present. Thoracic ventral glandular areas present; distinct paired ventrolateral swellings. Pygidium simple lobe. Pygidial appendages absent. Discrete head absent (ridge-like area at base of tentacular membrane).

Eyes on head absent or present, numerous, unpaired; head eyes simple pigmented cups. Buccal tentacles present (sometimes two or three kinds of tentacles, including those with expanded ends); arising from one side of mouth (but not retractable into mouth); grooved. Nuchal organs present; paired low projections from posterolateral prostomium; indistinct dorsolateral ciliated patches. Peristomium visible. Peristomium expanded into well-developed upper and lower lips.

Gut straight except for a large midbody loop. Foregut a muscular ventral pharynx. Pharynx dorsolateral ciliated folds absent or present. Heart bodies present.

First segment achaetous; second segment achaetous. First chaetiger with notochaetae only; parapodia similar in length or slightly shorter than subsequent parapodia; parapodia more-or-less laterally directed and free from head; chaetae similar in orientation, length and thickness to other chaetae. Parapodia present, or absent (very rarely). Parapodia uniramous (represented by notopodia on thorax and neuropodia on abdomen). Notopodial lobes represented by at least one chaetal lobe. Neuropodial lobes represented by at least one chaetal lobe, or low ridges (tori). Lateral organs present. Branchiae absent.

Chaetae absent (only in *Hauchiella*) or present, first appear on third segment after peristomium or fourth segment after peristomium, arranged in paired bundles (or rows) of many chaetae. Aciculae absent. Capillary chaetae present (rarely absent), in dorsal (notopodial) position, distally tapered to a point, edge smooth or corn-eared or hirsute-serrate. Capillary chaetae, externally not pseudo-segmented. Capillary chaetae, internally not chambered or hollow. Spines absent, or present (rarely, *Amaeana* only); slightly curved and more-or-less smooth; present in most or all chaetigers (mid and posterior body); in ventral (neuropodial) position only. Hooks absent. Uncini present, or absent (rarely); with teeth in vertical series, teeth usually similar-sized (=pectinate), or with teeth arranged in transverse series above an enlarged main fang (=crested), or with a single tooth surmounting main fang; in ventral (neuropodial) position throughout; arranged in one row (or singly).

Reproduction sexual (gonochoric). Segmental organs are metanephridia; metanephridia several pairs metanephridia anteriorly for excretion, posterior ones for gamete release (4–7 pairs).

Tube absent (although all *Polycirrus* are able to secrete copious mucus which can encapsulate the worm and resemble a tube).

**Literature used for description**. Stiller J, Tilic E, Rousset V, Pleijel F and Rouse GW (2020). Spaghetti to a Tree: A Robust Phylogeny for Terebelliformia (Annelida) Based on Transcriptomes, Molecular and Morphological Data. Biology 9, 73; doi:10.3390/biology9040073; Hutchings P, Nogueira JM de M, Carrerette O (2021) 7.7.3 Terebellidae s.l.: Polycirridae Malmgren, 1866, Terebellidae Johnston, 1846, Thelepodidae Hessle, 1917, Trichobranchidae Malmgren, 1866, and Telothelepodidae Nogueira, Fitzhugh & Hutchings, 2013 In: Purschke G, Böggemann M, Westheide W (Eds), Handbook of Zoology, Annelida, Volume 3: Pleistoannelida, Sedentaria III and Errantia I. De Gruyter, Berlin, 68–144; Rouse GW, Pleijel F, Tilic E (2022) Annelida. Oxford University Press, Oxford, UK.

# Terebellidae, Terebellinae Johnston, 1846 (excluding Polycirrini)

**Description**. Body segment number: variable. Body shape widest anteriorly and tapering posteriorly, regionalization present, regionalization comprising two regions, regions demarcated by absence of abdominal notopodia. Body pigmentation absent or present, bands or spots on the tentacles and other parts of the body; epidermis more-or-less smooth, or thick and rugose (anterodorsally). Thoracic ventral glandular areas present; distinct mid-ventral shield-shaped swellings. Pygidium simple lobe. Pygidial appendages absent. Discrete head absent (ridge-like area at base of tentacular membrane).

Eyes on head absent or present, numerous, unpaired; head eyes simple pigmented cups. Buccal tentacles present; arising from one side of mouth (but not retractible into mouth); grooved. Nuchal organs absent, or present; paired low projections from posterolateral prostomium; indistinct dorsolateral ciliated patches. Peristomium visible. Peristomium expanded into well-developed upper and lower lips.

Gut straight except for a large midbody loop. Foregut a muscular ventral pharynx. Pharynx dorsolateral ciliated folds absent or present. Heart bodies present.

First segment achaetous (may bear lateral lobes); second segment achaetous (may bear lateral lobes or dorsal branchiae). First chaetiger with notochaetae only; parapodia similar in length or slightly shorter than subsequent parapodia; parapodia more-or-less laterally directed and free from head; chaetae similar in orientation, length and thickness to other chaetae. Parapodia present. Parapodia biramous (thorax), or uniramous (abdomen); parapodial lobes prominent. Notopodial lobes represented by at least one chaetal lobe. Neuropodial lobes low ridges (tori). Lateral organs present. Branchiae present (rarely absent), arise from dorsal body, occur first few segments. Dorsal branchiae branching, or multiple filaments arising from a central stalk.

Chaetae first appear on fourth segment after peristomium, arranged in paired bundles (or rows) of many chaetae. Aciculae absent. Capillary chaetae present (rarely absent), in dorsal (notopodial) position, distally tapered to a point or sharply bent, edge smooth or hirsute-serrate. Capillary chaetae, externally not pseudo-segmented. Capillary chaetae, internally not chambered or hollow. Hooks absent. Uncini present; with teeth in vertical series, teeth usually similar-sized (=pectinate), or with teeth arranged in transverse series above an enlarged main fang (=crested); in ventral (neuropodial) position throughout; arranged in two rows.

Reproduction sexual (gonochoric).

Segmental organs are metanephridia; metanephridia several pairs metanephridia anteriorly for excretion, posterior ones for gamete release (4–7 pairs; rarely 0–1 pairs excretory nephridia).

# Tube absent, or present; membranous.

**Literature used for description**. Stiller J, Tilic E, Rousset V, Pleijel F and Rouse GW (2020). Spaghetti to a Tree: A Robust Phylogeny for Terebelliformia (Annelida) Based on Transcriptomes, Molecular and Morphological Data. Biology 9, 73; doi:10.3390/biology9040073; Hutchings P, Nogueira JM de M, Carrerette O (2021) 7.7.3 Terebellidae s.l.: Polycirridae Malmgren, 1866, Terebellidae Johnston, 1846, Thelepodidae Hessle, 1917, Trichobranchidae Malmgren, 1866, and Telothelepodidae Nogueira, Fitzhugh & Hutchings, 2013 In: Purschke G, Böggemann M, Westheide W (Eds), Handbook of Zoology, Annelida, Volume 3: Pleistoannelida, Sedentaria III and Errantia I. De Gruyter, Berlin, 68–144; Rouse GW, Pleijel F, Tilic E (2022) Annelida. Oxford University Press, Oxford, UK.

# Terebellidae, Thelepodinae Hessle, 1917

**Description**. Body segment number: variable. Body shape widest anteriorly and tapering posteriorly, regionalization present, regionalization comprising two regions, regions demarcated by absence of abdominal notopodia. Epidermis more-or-less smooth. Bioluminescence absent, or present. Thoracic ventral glandular areas present; distinct mid-ventral shield-shaped swellings. Pygidium simple lobe. Pygidial appendages absent. Discrete head absent (ridge-like area at base of tentacular membrane).

Eyes on head absent or present, numerous, unpaired; head eyes simple pigmented cups. Buccal tentacles present; arising from one side of mouth (but not retractable into mouth); grooved. Nuchal organs present; paired low projections from posterolateral prostomium; indistinct dorsolateral ciliated patches. Peristomium visible. Peristomium expanded into well-developed upper and lower lips.

Gut straight except for a large midbody loop. Foregut a muscular ventral pharynx. Pharynx dorsolateral ciliated folds absent or present. Heart bodies present.

First segment achaetous (may bear lateral lobes); second segment achaetous (may bear lateral lobes or dorsal branchiae). First chaetiger with notochaetae only; parapodia similar in length or slightly shorter than subsequent parapodia; parapodia more-or-less laterally directed and free from head; chaetae similar in orientation, length and thickness to other chaetae. Parapodia present. Parapodia biramous (thorax), or uniramous (abdomen); parapodial lobes prominent. Notopodial lobes represented by at least one chaetal lobe. Neuropodial lobes low ridges (tori). Lateral organs present. Branchiae present, arise from dorsal body, occur first few segments. Dorsal branchiae simple filaments each arising directly from body wall.

Chaetae first appear on fourth segment after peristomium, arranged in paired bundles (or rows) of many chaetae. Aciculae absent. Capillary chaetae present (rarely absent), in dorsal (notopodial) position, distally tapered to a point, edge smooth or hirsute-serrate. Capillary chaetae, externally not pseudo-segmented. Capillary chaetae, internally not chambered or hollow. Hooks absent. Uncini present; with teeth arranged in transverse series above an enlarged main fang (=crested); in ventral (neuropodial) position throughout; arranged in one row.

Reproduction sexual (gonochoric).

Segmental organs are metanephridia; metanephridia several pairs metanephridia anteriorly for excretion, posterior ones for gamete release (4–7 pairs).

Tube absent, or present; membranous, or leathery or parchment like.

**Literature used for description**. Stiller J, Tilic E, Rousset V, Pleijel F and Rouse GW (2020). Spaghetti to a Tree: A Robust Phylogeny for Terebelliformia (Annelida) Based on Transcriptomes, Molecular and Morphological Data. Biology 9, 73; doi:10.3390/biology9040073; Hutchings P, Nogueira JM de M, Carrerette O (2021) 7.7.3 Terebellidae s.l.: Polycirridae Malmgren, 1866, Terebellidae Johnston, 1846, Thelepodidae Hessle, 1917, Trichobranchidae Malmgren, 1866, and Telothelepodidae Nogueira, Fitzhugh & Hutchings, 2013 In: Purschke G, Böggemann M, Westheide W (Eds), Handbook of Zoology, Annelida, Volume 3: Pleistoannelida, Sedentaria III and Errantia I. De Gruyter, Berlin, 68–144; Rouse GW, Pleijel F, Tilic E (2022) Annelida. Oxford University Press, Oxford, UK.

# Thalassematidae Forbes & Goodsir, 1841, sensu lato

**Description**. Body segmentation absent. Body shape sausage or grub-shaped, regionalization present, regionalization comprising two regions, regions demarcated by presence of unsegmented trunk and a narrower, long anterior proboscis (ribbon-like proboscis leading to mouth at base). Trunk smooth or roughened by papillae or rounded skin bodies, anterior extremity of trunk with hardened or calcareous structures absent, posterior trunk with shield absent. Body pigmentation present, body often dark green, reddish-brown or yellowish. Pygidium absent. Elongate proboscis present; non-retractable and ribbon-like or spatulate; non-retractable elongated proboscis truncate, or bifid; non-retractable elongated proboscis short and scoop-like, or very long. Discrete head absent (represented by proboscis).

Eyes on head absent or present. Peristomium not visible.

Gut straight except for a large midbody loop. Foregut without a distinct ventral or axial pharynx. Gizzard variable. Calciferous glands absent. Caeca of hindgut present (as paired, branched anal vesicles).

Chaetae absent or present, more than two per bundle or one per bundle, only present anteriorly or present anteriorly and posteriorly. Anal chaetae absent, or present; one ring, or two rings; aciculae absent. Spines absent, or present. Hooks present; without distal hood, beard or ligament; occur in ventral (neuropodial) position; occur in anterior body only.

Segmental organs are metanephridia (usually); metanephridia along most of the body; anterior ones for excretion, posterior ones for gamete release (number of anterior pairs varies from one to over a hundred; posterior funnel-shaped structures on anal sac presumed to be gonoducts).

Tube absent.

**Literature used for description**. Edmonds SJ (2000), Chapter 4. Phylum Echiura. In Beesley PL, Ross GJB, Glasby CJ (Eds) Fauna of Australia Vol. 4A. Polychaetes & Allies. The Southern Synthesis. ABRS, Canberra. pp. 353–374; Brusca, RC, Moore, W & Shuster, SM. (eds) (2016) Chapter 14. Phylum Annelida. In: Invertebrates, 3rd Edition. Sinauer Associates, Sunderland, MA; Dohren J von (2019) 7.6.5 Echiura Stephen, 1965 (=Thalassematidae, Forbes & Goodsir, 1841) In Purschke G, Böggemann M, Westheide W (Eds), Handbook of Zoology, Annelida, Volume 2: Pleistoannelida, Sedentaria II. De Gruyter, Berlin, 404–450.

# Thalassematidae, Bonelliinae Lacaze-Duthiers, 1858

**Description**. Body segmentation absent. Body shape sausage or grub-shaped, regionalization present, regionalization comprising two regions, regions demarcated by presence of unsegmented trunk and a narrower, long anterior proboscis (ribbon-like proboscis leading to mouth at base). Trunk smooth or roughened by papillae or rounded skin bodies, anterior extremity of trunk with hardened or calcareous structures absent, posterior trunk with shield absent. Body pigmentation present, entire body often dark green or reddish-brown. Pygidium absent. Elongate proboscis present; non-retractable and ribbon-like or spatulate; non-retractable elongated proboscis bifid (usually); non-retractable elongated proboscis very long. Discrete head absent (represented by proboscis).

Eyes on head absent or present (on proboscis of females only). Peristomium not visible.

Gut straight except for a large midbody loop. Foregut without a distinct ventral or axial pharynx. Gizzard variable. Calciferous glands absent. Caeca of hindgut present (as paired, branched anal vesicles).

Chaetae absent or present, one per bundle, only present anteriorly (as a pair of stout ventral hooks). Anal chaetae absent, or present; aciculae absent. Hooks present; without distal hood, beard or ligament; occur in ventral (neuropodial) position; occur in anterior body only. Reproduction sexual.

Sexual reproduction gonochoric (males much smaller than females). Segmental organs are protonephridia (in dwarf males of Bonelliinae), or metanephridia; metanephridia along most of the body; anterior ones for excretion, posterior ones for gamete release (general pattern; funnel-shaped structures on anal sac presumed to be gonoducts); nephridial pores paired (ventrolateral) or single (ventromedial), nephridial pores located anteriorly (usually 1–2 pairs).

Tube absent.

**Literature used for description**. Edmonds SJ (2000), Chapter 4. Phylum Echiura. In Beesley PL, Ross GJB, Glasby CJ (Eds) Fauna of Australia Vol. 4A. Polychaetes & Allies. The Southern Synthesis. ABRS, Canberra. pp. 353–374; Brusca, RC, Moore, W & Shuster, SM. (eds) (2016) Chapter 14. Phylum Annelida. In: Invertebrates, 3rd Edition. Sinauer Associates, Sunderland, MA; Dohren J von (2019) 7.6.5 Echiura Stephen, 1965 (=Thalassematidae, Forbes & Goodsir, 1841) In Purschke G, Böggemann M, Westheide W (Eds), Handbook of Zoology, Annelida, Volume 2: Pleistoannelida, Sedentaria II. De Gruyter, Berlin, 404–450; Rouse GW, Pleijel F, Tilic E (2022) Annelida. Oxford University Press, Oxford, UK.

# Thalassematidae, Echiurinae Quatrefages, 1847

**Description**. Body segmentation absent. Body shape sausage or grub-shaped, regionalization present, regionalization comprising two regions, regions demarcated by presence of unsegmented trunk and a narrower, long anterior proboscis (ribbon-like proboscis leading to mouth at base). Trunk smooth or roughened by papillae or rounded skin bodies, anterior extremity of trunk with hardened or calcareous structures absent, posterior trunk with shield absent. Body pigmentation present, yellowish. Pygidium absent. Elongate proboscis present; non-retractable and ribbon-like or spatulate; non-retractable elongated proboscis truncate; non-retractable elongated proboscis short and scoop-like, or very long. Discrete head absent (represented by proboscis).

Peristomium not visible.

Gut straight except for a large midbody loop. Foregut without a distinct ventral or axial pharynx. Gizzard variable. Calciferous glands absent. Caeca of hindgut present (as tubular or sac-like anal vesicles).

Chaetae more than two per bundle or one per bundle, present anteriorly and posteriorly (stout ventral hooks anteriorly; ring of many spines posteriorly). Anal chaetae present; two rings; aciculae absent. Spines present; present only in posterior chaetigers. Hooks present; without distal hood, beard or ligament; occur in ventral (neuropodial) position; occur in anterior body only.

Segmental organs are metanephridia; nephridial pores located anteriorly, several pairs (usually 1–3 pairs).

Tube absent.

**Literature used for description**. Edmonds SJ (2000), Chapter 4. Phylum Echiura. In Beesley PL, Ross GJB, Glasby CJ (Eds) Fauna of Australia Vol. 4A. Polychaetes & Allies. The Southern Synthesis. ABRS, Canberra. pp. 353–374; Brusca, RC, Moore, W & Shuster, SM. (eds) (2016) Chapter 14. Phylum Annelida. In: Invertebrates, 3rd Edition. Sinauer Associates, Sunderland, MA; Dohren J von (2019) 7.6.5 Echiura Stephen, 1965 (=Thalassematidae, Forbes & Goodsir, 1841) In Purschke G, Böggemann M, Westheide W (Eds), Handbook of Zoology, Annelida, Volume 2: Pleistoannelida, Sedentaria II. De Gruyter, Berlin, 404–450; Rouse GW, Pleijel F, Tilic E (2022) Annelida. Oxford University Press, Oxford, UK.

# Thalassematidae, Ikedinae Bock, 1942

**Description**. Body segmentation absent. Body shape sausage or grub-shaped, regionalization present, regionalization comprising two regions, regions demarcated by presence of unsegmented trunk and a narrower, long anterior proboscis (ribbon-like proboscis leading to mouth at base). Trunk smooth or roughened by papillae or rounded skin bodies, anterior extremity of trunk with hardened or calcareous structures absent, posterior trunk with shield absent. Body pigmentation present, yellowish with brown flecks or stripes. Pygidium absent. Elongate proboscis present; non-retractable and ribbon-like or spatulate; non-retractable elongated proboscis very long. Discrete head absent (represented by proboscis).

Peristomium not visible.

Gut straight except for a large midbody loop. Foregut without a distinct ventral or axial pharynx. Gizzard variable. Calciferous glands absent. Caeca of hindgut present (as paired, elongate anal vesicles).

Chaetae one per bundle, only present anteriorly (as a pair of stout ventral hooks). Anal chaetae absent, or present; aciculae absent. Hooks present; without distal hood, beard or ligament; occur in ventral (neuropodial) position; occur in anterior body only.

Segmental organs are metanephridia; nephridial pores single (ventromedial), nephridial pores located anteriorly, more than 200 pairs.

Tube absent.

**Literature used for description**. Edmonds SJ (2000), Chapter 4. Phylum Echiura. In Beesley PL, Ross GJB, Glasby CJ (Eds) Fauna of Australia Vol. 4A. Polychaetes & Allies. The Southern Synthesis. ABRS, Canberra. pp. 353–374; Brusca, RC, Moore, W & Shuster, SM. (eds) (2016) Chapter 14. Phylum Annelida. In: Invertebrates, 3rd Edition. Sinauer Associates, Sunderland, MA; Dohren J von (2019) 7.6.5 Echiura Stephen, 1965 (=Thalassematidae, Forbes & Goodsir, 1841) In Purschke G, Böggemann M, Westheide W (Eds), Handbook of Zoology, Annelida, Volume 2: Pleistoannelida, Sedentaria II. De Gruyter, Berlin, 404–450; Rouse GW, Pleijel F, Tilic E (2022) Annelida. Oxford University Press, Oxford, UK.

# Thalassematidae, Thalassematinae Forbes & Goodsir, 1841

**Description**. Body segmentation absent. Body shape sausage or grub-shaped, regionalization present, regionalization comprising two regions, regions demarcated by presence of unsegmented trunk and a narrower, long anterior proboscis (ribbon-like proboscis leading to mouth at base). Trunk smooth or roughened by papillae or rounded skin bodies, anterior extremity of trunk with hardened or calcareous structures absent, posterior trunk with shield absent. Body pigmentation present, green, reddish or brown. Pygidium absent. Elongate proboscis present; non-retractable and ribbon-like or spatulate; non-retractable elongated proboscis truncate; non-retractable elongated proboscis very long. Discrete head absent (represented by proboscis).

Peristomium not visible.

Gut straight except for a large midbody loop. Foregut without a distinct ventral or axial pharynx. Gizzard variable. Calciferous glands absent. Caeca of hindgut present (as anal vesicles).

Chaetae absent or present, one per bundle, only present anteriorly (as a pair of stout ventral hooks). Anal chaetae absent, or present; aciculae absent. Hooks present; without distal hood, beard or ligament; occur in ventral (neuropodial) position; occur in anterior body only.

Segmental organs are metanephridia; nephridial pores located anteriorly, several pairs (usually 1–7 pairs, rarely up to 20 pairs).

# Tube absent.

**Literature used for description**. Edmonds SJ (2000), Chapter 4. Phylum Echiura. In Beesley PL, Ross GJB, Glasby CJ (Eds) Fauna of Australia Vol. 4A. Polychaetes & Allies. The Southern Synthesis. ABRS, Canberra. pp. 353–374; Brusca, RC, Moore, W & Shuster, SM. (eds) (2016) Chapter 14. Phylum Annelida. In: Invertebrates, 3rd Edition. Sinauer Associates, Sunderland, MA; Dohren J von (2019) 7.6.5 Echiura Stephen, 1965 (=Thalassematidae, Forbes & Goodsir, 1841) In Purschke G, Böggemann M, Westheide W (Eds), Handbook of Zoology, Annelida, Volume 2: Pleistoannelida, Sedentaria II. De Gruyter, Berlin, 404–450 ; Rouse GW, Pleijel F, Tilic E (2022) Annelida. Oxford University Press, Oxford, UK.

**Thalassematidae, Urechinae Monro, 1927**

**Description**. Body segmentation absent. Body shape sausage or grub-shaped, regionalization present, regionalization comprising two regions, regions demarcated by presence of unsegmented trunk and a narrower, long anterior proboscis (ribbon-like proboscis leading to mouth at base). Trunk smooth or roughened by papillae or rounded skin bodies, anterior extremity of trunk with hardened or calcareous structures absent, posterior trunk with shield absent. Body pigmentation absent or present. Pygidium absent. Elongate proboscis present; non-retractable and ribbon-like or spatulate; non-retractable elongated proboscis truncate; non-retractable elongated proboscis short and scoop-like. Discrete head absent (represented by proboscis).

Peristomium not visible.

Gut straight except for a large midbody loop. Foregut without a distinct ventral or axial pharynx. Gizzard variable. Calciferous glands absent. Caeca of hindgut present (as pair of large anal vesicles).

Chaetae present, one per bundle, only present anteriorly (as a pair of stout ventral hooks). Anal chaetae present; one ring; aciculae absent. Hooks present; without distal hood, beard or ligament; occur in ventral (neuropodial) position; occur in anterior body only.

Segmental organs are metanephridia; nephridial pores located anteriorly, several pairs (usually 2–3 pairs).

Tube absent.

**Literature used for description**. Edmonds SJ (2000), Chapter 4. Phylum Echiura. In Beesley PL, Ross GJB, Glasby CJ (Eds) Fauna of Australia Vol. 4A. Polychaetes & Allies. The Southern Synthesis. ABRS, Canberra. pp. 353–374; Brusca, RC, Moore, W & Shuster, SM. (eds) (2016) Chapter 14. Phylum Annelida. In: Invertebrates, 3rd Edition. Sinauer Associates, Sunderland, MA; Dohren J von (2019) 7.6.5 Echiura Stephen, 1965 (=Thalassematidae, Forbes & Goodsir, 1841) In Purschke G, Böggemann M, Westheide W (Eds), Handbook of Zoology, Annelida, Volume 2: Pleistoannelida, Sedentaria II. De Gruyter, Berlin, 404–450; Rouse GW, Pleijel F, Tilic E (2022) Annelida. Oxford University Press, Oxford, UK.

# Tiguassidae Brinkhurst, 1988

**Description**. Body segment number: variable. Secondary annulation absent or present. Body shape elongate, more-or-less equal width over entire length.

Prostomium anteriorly with an anterior tentacle-like projection (='proboscis').

Foregut a muscular dorsal pharynx. Gizzard present (cylindrical), in segments VI-VIII. Heart bodies present, segment X.

Chaetae first appear on first segment after peristomium (=S2 for oligochaete workers), in lumbricine arrangement - paired upper and lower bundles of one or a few chaetae each - closely spaced lateral and ventrolateral pairs. Capillary (=hair) chaetae absent. Crotchet chaetae present.

Sexual reproduction hermaphroditic. Clitellum thin, single layered, in region of male pores (male pores on first clitellar segment), and female pores. Clitellum occurs from XII to XVII. Testes present, one pair in total; present in segment XI. Ovaries present, one pair; present in segments XII. Segmental organs are metanephridia; both nephridial pores and gonoducts located around clitellum. Spermathecae present, pre-testicular. Spermathecal pores present, located within 1 or 2 segments of male pores; 2 pairs; in segment IX and X. Female gonoduct present. Female pores one pair, in segment XIII. Male gonoducts present. Male pores one pair (a vestigial second pair may be present), in segment XII, plesioporous (in segment following testicular segment). Prostate gland absent.

**Literature used for description**. Brinkhurst RO (1988) A taxonomic analysis of the Haplotaxidae (Annelida, Oligochaeta). Canadian Journal of Zoology 66: 2243–2252; Jamieson,

BGM (2006) Non-leech Clitellata (with contributions by Marco Ferraguti). Pp. 235–392. In Reproductive Biology and Phylogeny of Annelida. Series Editor BGM Jamieson. Volume 4. Editors G. Rouse, F. Pleijel. Science Publishers, Enfield, New Hampshire.

# Tomopteridae Grube, 1850

**Description**. Body segment number: variable. Body shape elongate, more-or-less equal width over entire length, regionalization absent (though the posterior end may be quite different from the rest of the body - highly extended with very short segments having rudimentary parapodia). Body translucent, gut visible, pigmentation absent or present (as faint transverse stripes), pigmentation discrete pigmented glands on parapodia and/or transverse body stripes; epidermis more-or-less smooth. Bioluminescence absent, or present. Pygidium simple lobe. Pygidial appendages absent. Discrete head present (head fused with first segments; distinctive pair of long sensory palps and even longer tentacular cirri); complex in shape bearing appendages.

Prostomium rounded to oval (very small). Postcephalic eyes present (one pair, with lenses); subepidermal. Palps present; anteroventral; tapering (usually) sensory type; unarticulated. Nuchal organs present; paired low projections from posterolateral prostomium; posterior projections. Peristomium not visible.

Gut more-or-less straight, lacking side branches. Foregut a muscular axial pharynx.

First segment tentaculate, or achaetous (a single pair of cirri is present in juveniles only); second segment tentaculate (extremely long pair of tentacles, supported by aciculae, that are often much wider than the body of the worm). Tentacular cirri present; 1–2 pair(s); arise on a single segment (very long); internal aciculae present in at least some cirri. First chaetiger with neurochaetae only (internal chaetae); parapodia similar in length or slightly shorter than subsequent parapodia; parapodia more-or-less laterally directed and free from head; chaetae similar in orientation, length and thickness to other chaetae. Parapodia biramous; parapodial lobes prominent. Notopodial lobes elongate, ending in rounded lappet. Neuropodial lobes elongate, ending in rounded lappet. Dorsal cirri present; flattened and foliaceous. Ventral cirri present; foliaceous. Branchiae absent.

Chaetae present (only internal chaetae = aciculae), first appear on second segment after peristomium, arranged in paired bundles (or rows) of many chaetae. Aciculae present (in second segment only); in ventral position (=neuroaciculae). Capillary chaetae absent. Hooks absent.

Segmental organs are metanephridia. Tube absent.

**Literature used for description**. Glasby CJ, Fauchald K (2003). POLiKEY. An information system for polychaete families and higher taxa. Version 2, 5 June 2003. Australian Biological Resources Study, DCCEEW, Canberra, https:[//www.dcceew.gov.au/;](http://www.dcceew.gov.au/%3B) Fernandez-Almo MA (2022)

7.13.13 Tomopteridae Grube, 1850 In: Purschke G, Böggemann, M, Westheide W (Eds) Handbook of Zoology. Annelida. Vol. 4: Pleistoannelida, Errantia II. De Gruyter, Berlin, 400–412; Rouse GW, Pleijel F, Tilic E (2022) Annelida. Oxford University Press, Oxford, UK.

# Trichobranchidae Malmgren, 1866

**Description**. Body segment number: variable. Body shape widest anteriorly and tapering posteriorly, regionalization present, regionalization comprising two regions, regions demarcated by absence of abdominal notopodia. Epidermis more-or-less smooth. Thoracic ventral glandular areas present; indistinct mid-ventral swelling, or distinct mid-ventral shield-shaped swellings. Pygidium simple lobe. Pygidial appendages absent. Discrete head absent (projects as a tentacular membrane).

Eyes on head absent (rarely present). Buccal tentacles present (sometimes two kinds of tentacles - distally expanded and filiform); arising from one side of mouth; grooved. Nuchal organs present; paired low projections from posterolateral prostomium; indistinct dorsolateral ciliated patches. Peristomium visible. Peristomium expanded into well-developed upper and lower lips (lower lip conical in *Artacama* and some *Trichobranchus*).

Gut straight except for a large midbody loop. Foregut a muscular ventral pharynx. Protrusible proboscis absent or present (a conical proboscis is present in some *Trichobranchus*). Pharynx dorsolateral ciliated folds present (?). Heart bodies present.

First segment achaetous (may form a proboscis); second segment achaetous. First chaetiger with notochaetae only; parapodia similar in length or slightly shorter than subsequent parapodia; parapodia more-or-less laterally directed and free from head; chaetae similar in orientation, length and thickness to other chaetae. Parapodia present. Parapodia biramous (thorax), or uniramous (abdomen); parapodial lobes prominent. Notopodial lobes represented by at least one chaetal lobe. Neuropodial lobes low ridges (tori) (over most of body). Lateral organs present. Branchiae absent or present, arise from dorsal body, occur first few segments or occur on mid-body segments (branchiae may be fused forming a single structure). Dorsal branchiae lamellate, or multiple filaments arising from a central stalk, or simple filaments each arising directly from body wall.

Chaetae first appear on third segment after peristomium or fourth segment after peristomium or fifth segment after peristomium or sixth segment after peristomium, arranged in paired bundles (or rows) of many chaetae. Aciculae absent. Capillary chaetae present, in dorsal (notopodial) position, distally tapered to a point, edge smooth (narrowly winged). Capillary chaetae, externally not pseudo-segmented. Capillary chaetae, internally not chambered or hollow. Spines absent, or present; sharply bent (=geniculate) or recurved; present only in one or a few anterior chaetigers; in ventral (neuropodial) position only. Hooks present; without distal hood, beard or ligament; occur in ventral (neuropodial) position; occur in anterior body only. Uncini present (abdominal neuropodia); with teeth arranged in transverse series above an enlarged main fang (=crested); in ventral (neuropodial) position throughout; arranged in one row.

Reproduction sexual (gonochoric).

Segmental organs are metanephridia; metanephridia several pairs metanephridia anteriorly for excretion, posterior ones for gamete release (4–7 pairs); several pairs in anterior body.

Tube present; membranous.

**Literature used for description**. Glasby CJ, Fauchald K (2003). POLiKEY. An information system for polychaete families and higher taxa. Version 2, 5 June 2003. Australian Biological Resources Study, DCCEEW, Canberra, https:[//www.dcceew.gov.au/;](http://www.dcceew.gov.au/%3B) Stiller J, Tilic E, Rousset V, Pleijel F and Rouse GW (2020). Spaghetti to a Tree: A Robust Phylogeny for Terebelliformia (Annelida) Based on Transcriptomes, Molecular and Morphological Data. Biology 9, 73; doi:10.3390/biology9040073; Rouse GW, Pleijel F, Tilic E (2022) Annelida. Oxford University Press, Oxford, UK.

# Tritogeniidae Plisko, 2013

**Description**. Body segment number: variable. Secondary annulation absent or present. Body shape elongate, more-or-less equal width over entire length (compact and short). Pygidium simple lobe. Pygidial appendages absent.

Foregut a muscular dorsal pharynx. Gizzard present (one or two), in segments VI-VII and the second in segment IX.

Chaetae first appear on first segment after peristomium (=S2 for oligochaete workers), in lumbricine arrangement - paired upper and lower bundles of one or a few chaetae each (chaetae minute). Crotchet chaetae present (minute, barely distinguishable).

Sexual reproduction hermaphroditic. Clitellum thick, multilayered, partially encircles body (on some segments; often less developed ventrally), in region of male pores. Clitellum occurs from XIII to XXVIII. Tubercula pubertatis present, paired ridges ventral to clitellum. Gonadal segments bearing genital papillae absent (mostly before clitellum). Testes present, two pairs in total, one pair per segment; present in segments X or XI. Ovaries present; present in segment XIII. Segmental organs are metanephridia; multiple, minute, nephridia in each segment (meronephridia). Spermathecae present, simple, post-testicular or pre-testicular or in testicular segments. Spermathecal pores present; 5 (or more); in segment IX/X to XV-XVII (in segmental furrows). Female gonoduct present. In segment XIV. Male gonoducts present. Male pores variable (difficult to detect), in segment XVIII or XIX in intersegmental furrows, opisthoporous (two or more segments following testicular segment). Prostate gland absent.

**Literature used for description**. Plisko JD (2013). A New Family Tritogeniidae for the genera *Tritogenia* and *Michalakus*, earlier accredited to the composite Microchaetidae (Annelida: Oligochaeta). African Invertebrates 54: 69–92. Plisko JD, Nxele, TC (2015) An Annotated Key Separating Foreign Earthworm Species from the Indigenous South African taxa (Oligochaeta: Acanthodrilidae, Eudrilidae, Glossoscolecidae, Lumbricidae, Megascolecidae, Microchaetidae, Ocnerodrilidae and Tritogeniidae). African Invertebrates 56: 663–708.

# Trochochaetidae Pettibone, 1963

**Description**. Body segment number: variable. Body shape elongate, more-or-less equal width over entire length, regionalization present, regionalization comprising two regions, regions demarcated by structural differences in parapodia over body. Epidermis more-or-less smooth. Pygidium simple lobe. Pygidial appendages present, more than four cirri. Discrete head present; complex in shape bearing appendages.

Prostomium triangular to trapezoidal (narrow end posteriorly). Prostomial antennae absent, or present; median one only; unarticulated. Palps present; anterodorsal; grooved (usually) feeding type; feeding palp longitudinally grooved. Caruncle absent, or present. Nuchal organs present; single antenna-like projection from posterior prostomium (as an occipital antenna). Peristomium visible. Peristomium a single ring, collar-like.

Foregut a non-muscular axial pharynx.

First segment chaetous; second segment chaetous. First chaetiger with both notochaetae and neurochaetae; parapodia very elongated; parapodia anteriorly directed and wrapping around head; chaetae slender and elongate, forming cage (or basket) around head. Parapodia biramous (notopodia of midbody absent); parapodial lobes prominent. Notopodial lobes represented by at least one chaetal lobe. Neuropodial lobes represented by at least one chaetal lobe. Lateral organs present. Branchiae present (rarely absent), arise from lateral body, occur first few segments or occur on mid-body segments; lateral branchiae digitiform.

Chaetae first appear on first segment after peristomium, arranged in paired bundles (or rows) of many chaetae. Aciculae absent. Capillary chaetae present, in dorsal (notopodial) position or in ventral (neuropodial) position, distally tapered to a point, edge smooth or hirsute-serrate. Capillary chaetae, externally not pseudo-segmented. Capillary chaetae, internally not chambered or hollow. Spines present; slightly curved and more-or-less smooth, or with a single distal or subdistal hair (=aristate or hooded); present in most or all chaetigers (those of segment 2/3 very stout, dark coloured); in ventral (neuropodial) position only. Hooks absent. Segmental organs are metanephridia; metanephridia along most of the body; anterior ones for excretion, posterior ones for gamete release.

Tube absent, or present; membranous (long and fragile).

**Literature used for description**. Glasby CJ, Fauchald K (2003). POLiKEY. An information system for polychaete families and higher taxa. Version 2, 5 June 2003. Australian Biological Resources Study, DCCEEW, Canberra, https:[//www.dcceew.gov.au/;](http://www.dcceew.gov.au/%3B) Blake JA, Maciolek NJ (2019) 7.4.3 Trochochaetidae Pettibone, 1963 In: Purschke G, Böggemann M, Westheide W (Eds), Handbook of Zoology, Annelida, Volume 2: Pleistoannelida, Sedentaria II. De Gruyter, Berlin, 120–135; Rouse GW, Pleijel F, Tilic E (2022) Annelida. Oxford University Press, Oxford, UK.

# Tumakidae Righi, 1995

**Description**. Body segment number: variable, segmentation present. Secondary annulation present; anterior region biannulate; mid-body triannulate; posterior 3-annulate. Body shape elongate, more-or-less equal width over entire length. Dorsal intersegmental furrow present, pores on mid-dorsal line present (intersegment of 10/11 in *Tumak amari*). Pygidium simple lobe (arch shaped). Pygidial appendages absent.

Gut more-or-less straight, lacking side branches. Foregut a muscular dorsal pharynx. Gizzard present, in segment XXV-XXX. Calciferous glands present, in segment XX-XXIV. Intestinal typhlosole present, formed from the inner (epithelial) layer only of the intestine (? T-shaped, voluminous, begins on XXXIII-XXXIV in *Tumak anari*). Heart bodies present (at least 5 pairs in *Tumak amari*), segments V-IX.

Chaetae first appear on first segment after peristomium (=S2 for oligochaete workers), in lumbricine arrangement - paired upper and lower bundles of one or a few chaetae each. Crotchet chaetae present, simple-pointed. Genital chaetae absent, or present.

Sexual reproduction hermaphroditic. Clitellum may be poorly defined, thick, multilayered, partially encircles body, in region of male pores. Clitellum occurs from XIV to

XXV. Seminal groove absent. Tubercula pubertatis present (between segments XVII-XX; called puberal bands by Jamieson 2006), paired ridges on ventrolateral margins of clitellum. Gonadal segments bearing genital papillae present (also present prior to the clitellum). Testes present, two pairs in total, one pair per segment; present in segments X or XI. Sperm sac absent. Ovaries present; present in segment XIII. Segmental organs are metanephridia; nephridial pores paired (ventrolateral) (minute), both nephridial pores and gonoducts located around clitellum. Spermathecae present, simple, post-testicular. Spermathecal pores present (minute), paired; 2; in segment XII-XVI; intersegmental grooves. Female gonoduct present. Female pores one pair (minute), in segment XIV. Male gonoducts present (paired). Male pores single, median (minute), in segment XVII-XIX, opisthoporous (two or more segments following testicular segment). Prostate gland absent.

**Literature used for description**. Jamieson, BGM (2006) Non-leech Clitellata (with contributions by Marco Ferraguti). Pp. 235–392. In Reproductive Biology and Phylogeny of Annelida. Series Editor BGM Jamieson. Volume 4. Editors G Rouse, F Pleijel. Science Publishers, Enfield, New Hampshire. Celis LV, Rangel-Ch. O (2015) Dos especies nuevas (Oligochaeta: Tumakidae) de la región Caribe de Colombia. Papeis Avulso de Zoologica 55: 405–414.

# Typhloscolecidae Uljanin, 1878

**Description**. Body segment number: variable. Body shape elongate, more-or-less equal width over entire length (widest mid-anteriorly, tapering posteriorly). Body translucent, gut visible, pigmentation absent or present, pigmented coloured glands on dorsal cirri. Pygidium simple lobe. Pygidial appendages present, one pair of cirri. Discrete head present; complex in shape bearing appendages. Prostomium bluntly conical. Prostomial antennae absent, or present; median one only; unarticulated. Nuchal organs present; paired low projections from posterolateral prostomium; posterior projections (as conspicuous epaulettes). Peristomium not visible.

Gut straight with side branches. Foregut a muscular axial pharynx.

First segment tentaculate (foliaceous); second segment tentaculate. Tentacular cirri present; 2–3 pair(s); arise over two or more segments. First chaetiger with neurochaetae only; parapodia similar in length or slightly shorter than subsequent parapodia; parapodia more-or-less laterally directed and free from head; chaetae similar in orientation, length and thickness to other chaetae. Parapodia uniramous. Notopodial lobes absent (though dorsal cirri are present). Neuropodial lobes represented by at least one chaetal lobe. Dorsal cirri present; flattened and foliaceous. Ventral cirri present; foliaceous.

Chaetae first appear on fifth segment after peristomium or sixth segment after peristomium, arranged as a single bundle (or row) of many chaetae (lower bundle - neurochaetae - only). Aciculae present; in ventral position (=neuroaciculae) (emergent). Capillary chaetae absent. Spines present; slightly curved and more-or-less smooth; present in most or all chaetigers (absent anteriorly); in ventral (neuropodial) position only. Hooks absent.

Reproduction sexual (sequential hermaphroditism). Segmental organs are protonephridia.

Tube absent.

**Literature used for description**. Glasby CJ, Fauchald K (2003). POLiKEY. An information system for polychaete families and higher taxa. Version 2, 5 June 2003. Australian Biological Resources Study, DCCEEW, Canberra, https:[//www.dcceew.gov.au/;](http://www.dcceew.gov.au/%3B) Rouse GW, Pleijel F, Tilic E (2022) Annelida. Oxford University Press, Oxford, UK.

# Uncispionidae Green, 1982

**Description**. Body segment number: variable. Body shape elongate, more-or-less equal width over entire length. Epidermis more-or-less smooth. Pygidium with multiple digitate lobes. Discrete head present; complex in shape bearing appendages.

Prostomium triangular to trapezoidal (narrow end posteriorly). Prostomial antennae present; median one only; unarticulated. Palps present; anterodorsal; grooved (usually) feeding type; feeding palp longitudinally grooved. Caruncle absent, or present. Nuchal organs absent, or present; paired low projections from posterolateral prostomium; indistinct dorsolateral ciliated patches, or posterior projections. Peristomium visible. Peristomium a single ring, collar-like (collar bearing small oral lobes laterally and ventrally).

Foregut a non-muscular axial pharynx.

First segment chaetous; second segment chaetous. First chaetiger with both notochaetae and neurochaetae; parapodia very elongated; parapodia anteriorly directed and wrapping around head; chaetae slender and elongate, forming cage (or basket) around head. Parapodia biramous; parapodial lobes prominent. Notopodial lobes represented by at least one chaetal lobe. Neuropodial lobes represented by at least one chaetal lobe. Branchiae present (rarely absent), arise from lateral body (fused basally to notopodium), occur on mid-body segments; lateral branchiae digitiform.

Chaetae first appear on first segment after peristomium, arranged in paired bundles (or rows) of many chaetae. Aciculae absent. Capillary chaetae present, in dorsal (notopodial) position or in ventral (neuropodial) position, distally tapered to a point, edge corn-eared or hirsute-serrate. Capillary chaetae, externally not pseudo-segmented. Capillary chaetae, internally not chambered or hollow. Spines present; slightly curved and more-or-less smooth; present only in one or a few anterior chaetigers (chaetiger 1); in dorsal (notopodial) position only. Hooks present (posterior-most ones enlarged and modified); with a distal hood; occur in ventral (neuropodial) position; occur in posterior body only.

Segmental organs are metanephridia.

Tube present; membranous.

**Literature used for description**. Glasby CJ, Fauchald K (2003). POLiKEY. An information system for polychaete families and higher taxa. Version 2, 5 June 2003. Australian Biological Resources Study, DCCEEW, Canberra, https:[//www.dcceew.gov.au/;](http://www.dcceew.gov.au/%3B) Blake JA, Maciolek NJ (2019) 7.4.4 Uncispionidae Green, 1982 In: Purschke G, Böggemann M, Westheide W (Eds), Handbook of Zoology, Annelida, Volume 2: Pleistoannelida, Sedentaria II. De Gruyter, Berlin, 136–144; Rouse GW, Pleijel F, Tilic E (2022) Annelida. Oxford University Press, Oxford, UK.

# Xerobdellidae Moore, 1946

**Description**. Body segment number: fixed, 34 segments including 2 preoral 'segments' (prostomium and peristomium) and 32 postoral segments. Secondary annulation present (disk-shaped receptors called sensillae mark the middle annulus of each segment); mid-body 5-annulate (Serge Utevski, pers. obs.). Body shape elongate, more-or-less equal width over entire length (when relaxed; but can be dramatically shortened by contraction of longitudinal muscles during movement), more-or-less cylindrical. Anterior end sucker on ventral surface present (posterolateral sucker bears a pair of short lateral cirri; they have been called sensory palps by leech workers); not clearly separated from rest of body. Large mouth on ventral surface of sucker. Posterior sucker on ventral surface present; circular (auricles absent); rays present (radiating out from central pore); anus positioned near posterior end (dorsally, just before posterior sucker).

Eyes on head present, five pairs or four pairs (on segments II-V and VI/VII); head eyes simple pigmented cups. Palps absent.

Gut straight with side branches. Foregut a muscular axial pharynx; muscular axial pharynx not rotated (euthylaematous). Pharynx jaws present, leech-type (either soft-muscular or horny) (as soft muscular 'jaws'); two (duognathous) muscular jaws, or three (trignathous) muscular jaws arranged in a triangle. Protrusible proboscis absent. Crop present. Caeca of midgut (=posterior crop caeca) present (11 pairs). Circulatory system absent.

Sexual reproduction hermaphroditic. Clitellum fully encircles body, in region of male and female pores. Clitellum occurs from X-XIII (usual position for leeches). Testes present, many pairs. Sperm sac present. Ovaries present, one pair; present in segment XII. Egg sacs present; globular. Oviduct single, shared oviduct from egg sac, or separate one for each egg sac. Segmental organs are metanephridia; metanephridia ventral; nephridial pores paired (ventrolateral) (except for 17th nephridial pair opening via a single medioventral pore). Female gonoduct present. Female pores single, median (in form of a vagina), in segment XII/XIII (in intersegmental furrow). Male gonoducts present, with atrium present, fused. Male pores single, median, in segment XI. Penis present (straight), with hardened sheath present (in XII). Prostate gland absent.

**Literature used for description**. Siddall ME, Borda E (2004) Leech Collections from Chile Including Two New species of *Helobdella* (Annelida: Hirudinida). American Museum Novitates 3457: 1–18; Siddall ME, Bely AE, Borda E (2006) Hirudinida Pp. 393–429. In Reproductive Biology and Phylogeny of Annelida. Series Editor BGM Jamieson. Volume 4. Editors G Rouse, F Pleijel. Science Publishers, Enfield, New Hampshire; Borda E, Oceguera-Figueroa A, Siddall ME (2008) On the classification, evolution and biogeography of terrestrial haemadipsoid leeches (Hirudinida: Arhynchobdellida: Hirudiniformes). Molecular Phylogenetics and Evolution 46 (2008) 142–154.

# Yndolaciidae Støp-Bowitz, 1987

**Description**. Body segment number: variable. Body shape elongate, more-or-less equal width over entire length. Epidermis more-or-less smooth. Pygidium simple lobe. Pygidial appendages absent or present, one pair of cirri. Discrete head present; lobe-like without appendages.

Prostomium pentagonal to quadrangular. Palps absent. Nuchal organs present; paired low projections from posterolateral prostomium; posterolateral ciliated bulbs (elaborate and ramified in some species). Peristomium not visible.

Foregut a muscular axial pharynx (assumption based on affinity with Phyllodocida). Pharynx teeth absent. Protrusible proboscis present, smooth (apart from cilia).First segment tentaculate (2 pairs either on segment1 or spread over a fused segment 1 and 2); second segment chaetous and tentaculate. Tentacular cirri present; 2 pair(s); arise on a single segment, or over two or more segments (uncertain if one or more than one segment); internal aciculae present in at least some cirri. First chaetiger with both notochaetae and neurochaetae; parapodia similar in length or slightly shorter than subsequent parapodia; parapodia more-or-less laterally directed and free from head; chaetae similar in orientation, length and thickness to other chaetae. Parapodia biramous; parapodial lobes prominent. Notopodial lobes represented by at least one chaetal lobe. Neuropodial lobes represented by at least one chaetal lobe. Dorsal cirri present; more-or-less cirriform. Ventral cirri present; cirriform or tapering.

Chaetae first appear on second segment after peristomium or third segment after peristomium (uncertain), arranged as a single bundle (or row) of many chaetae. Aciculae present; in both dorsal and ventral positions (=noto- and neuroaciculae). Neuroaciculae distally tapering. Capillary chaetae absent. Hooks absent. Compound chaetae present; in dorsal (notopodial) position, or in ventral (neuropodial) position; appendage distally tapering to slender tips (=spinigerous).

Tube absent.

**Literature used for description**. Rouse GW, Pleijel F, Tilic E (2022) Annelida. Oxford University Press, Oxford, UK.
