# Supplementary material for: ﻿ANNiKEY Linear – diagnoses, descriptions, and a single-access identification key to Annelida family-level taxa
Source: Zookeys. 2025 Jul 31;1247:217–403. doi: 10.3897/zookeys.1247.137606 (PMC12344570; doi:10.3897/zookeys.1247.137606)
Supplement: ﻿Supplementary material 2 — Annotated morphological character list for Annelida [file zookeys-1247-217_article-137606__-s002.docx]

Supplementary File S2. Morphological character list

(extracted from Glasby CJ, Biriukova O, Martin P, Utevsky S, Wilson RS (2024) ANNiKEY Interactive: a taxonomic information system and a multi-access key to Annelida family-level taxa, [doi.org/10.5281/zenodo.13738486](file:///C:\Users\glasb\Downloads\doi.org\10.5281\zenodo.13738486).

#1-39. Non-morphological characters

#40. Body segment number: <fixed or variable>/

1. fixed/

2. variable <segments added as worm grows; usually numerous - more than about 15 in adults>/

#41. Body segmentation <=metamerism; presence of delimited annuli; interpretation does not align with Rouse & Fauchald 1997>/

1. absent/

2. present/

A segment is a unit which is serially repeated and comprises the body of the worm; often separated internally by septa (see Annelida Glossary). In most polychaetes and oligochaetes segments are often demarcated externally by parapodia (sometimes) and chaetae. Note that how segments have been designated and counted in Annelida differs depending on the group. In polychaetes, Arabic numbers have been used (S1, S2, S3 etc); in leeches and oligochaetes, Roman numerals (SI, SII, SIII ...) have been preferred. In polychaetes segment numbering starts after the presegmental prostomium and peristomium, in oligochaetes the peristomium is counted as the first segment, while in leeches the prostomium and peristomium have been counted as the first two segments. In AnniKey we have maintained the historical numbering sequence of each group in order to facilitate comparisons within each group and with previous studies; however, we have defaulted to Arabic numeral designation for all annelids.

#42. <Body segment fixed at; count only applies to adults, not including rare cases of adjoining juveniles that bud from the tail in asexually reproducing forms>/

1. less than 14 segments/

2. 15 segments/

3. 31 segments including 2 preoral 'segments' (prostomium and peristomium) and 29 postoral segments/

4. 34 segments including 2 preoral 'segments' (prostomium and peristomium) and 32 postoral segments/

Counting body segments is usually straightforward, especially when the segments bear parapodia and or chaetae. When lacking, e.g., in leeches, distinguishing segments is difficult, particularly because the pseudoannulations of leeches resemble the annulations that mark true segments. Leech segment counting follows Sawyer (1986: 54–66), viz. body is comprised of 34 segments (I–XXXIV; ie., counting the preoral prostomium and peristomium as segments): six segments (I–VI) constitute the head, seven (XXVIII–XXXIV) constitute the tail (caudal sucker), and the intervening 21 segments (VII–XXVII) constitute the midbody. Acanthobdellid segment counting is similar (prostomium and peristomium counted as segments).

#43. <Body> secondary annulation <presence of segments within segments>/

1. absent/

2. present/

While true segments are often separated internally by septa, secondary segmentation (=pseudosegments, pseudoannulations or referred to as somites in some leech literature) is only visible on the body surface in leeches and some polychaetes and oligochaetes. Pseudoannulations of polychaetes are often restricted to certain parts of the body, unlike leeches in which they occur throughout the length of the body. Leech workers have developed the following notation for identifying the annulations: the mid-body segment is comprised typically of five external annuli, labeled from anterior to posterior as b1, b2, a2, b5 and b6 (This is derived from the “primitive” condition in which a leech segment is notionally tri-annulate, a1, a2 and a3; in hirudinid leeches the first and third notional annuli are further subdivided into b1 and b2; and b5 and b6, respectively.) The middle annulus, a2, is the centre of the segment, and is defined externally as the sensilla-bearing annulus, and internally by location of the respective ganglion. Thus, for example, XII b2/a2 refers to the furrow between the second and third annuli of segment twelve. See also 'biannulate segment' (bis) in Annelida Glossary.

#44. Anterior region <secondary annulation; number of annuli in segments I-VI; score for leeches only>/

1. uniannulate/

2. biannulate/

3. triannulate/

#45. Mid-body <secondary annulation; number of annuli in segment VII to XXIII; score for leeches only>/

1. uniannulate/

2. biannulate/

3. triannulate/

4. 4-annulate/

5. 5-annulate/

6. 6-annulate/

7. 7-annulate/

8. 8-annulate, or more/

#46. Posterior <secondary annulation; number of annuli in the caudal sucker region XXIV-XXVII; score for leeches only>/

1. uniannulate/

2. biannulate/

3. 3-annulate/

4. 4-annulate/

5. 5-annulate/

#47. Post-anal region <secondary annulation; number of annuli in the postanal region; score for leeches only>/

1. absent/

2. uniannulate/

#48. Body segments <relative size along body>/

1. similar dimensions throughout/

2. strongly elongate in midbody/

Most annelids have similar-sized segments along the body; a few polychaete families have unusually elongated segments in part of the body (usually middle), but otherwise show no body regionalisation.

#49. Elongate body segments <form of parapodia>/

1. with distinct (but truncate) notopodia and neuropodial tori/

2. with indistinct parapodia, chaetae arise directly from body wall/

3. with indistinct parapodia, chaetae (uncini) arising from raised annuli/

Tori and raised annuli (states 1, 3, respectively) are referred to as girdles in Siboglinidae (see Annelid Glossary).

#50. Body shape <dorsal view>/

1. elongate, more or less equal width over entire length <=vermiform>/

2. widest anteriorly and tapering posteriorly <=arenicoliform, sipunculiform>/

3. sausage or grub-shaped/

4. ovate to elliptical/

5. pyriform <=pear-shaped; narrow end anteriorly>/

6. circular/

7. peanut shaped/

Overall body shape, as viewed dorsally, is a useful character but it can be difficult to determine with precision; when unsure, select multiple states.

#51. Body shape <in cross-section>/

1. dorsoventrally flattened <body depth much less then body width>/

2. more-or-less cylindrical <body depth not significantly less than body width>/

Most worms are more or less cylindrical in cross-section (occasionally a worm may be distinctly quadrangular, but this is noted as a variation of the cylindrical type). In a dorsoventrally flattened worm, the body width is many times greater than the body depth.

#52. Body regionalization <presence of two or more different regions of the body>/

1. absent <similar body form throughout; variation in width and thickness not considered different regions>/

2. present/

Body regionalization is typical of many polychaete families; when there are two regions (bipartite) the regions are often referred to as thorax and abdomen; three distinct regions (tripartite) occur in other polychaete families. Note that this character does not include the smaller body changes across first few or last few segments that are typical of many annelids, particularly polychaetes.

#53. Body regionalization comprising <number major regions>/

1. two regions <i.e., bipartite body, e.g., thorax/abdomen, trunk/introvert, trunk/proboscis>/

2. three regions <i.e., tripartite body>/

Body regions are marked by changes in morphology, for example, shifts in the form of the parapodia, body thickness, or body surface texture. In those leeches in which the body is clearly divided into two regions, the anterior region is called the trachelosome and the posterior region, the urosome. See 'bipartite body', 'tripartite body', 'trachelosome' and 'urosome' in the Annelida Glossary.

#54. Body regions demarcated by <often gross differences in parapodial morphology>/

1. absence of abdominal notopodia/

2. laterally-directed thoracic parapodia and dorsally-directed midbody and abdominal parapodia/

3. change in chaetal types over body/

4. structural differences in parapodia over body <not including first few chaetigers>/

5. inversion of parapodia/

6. presence of unsegmented trunk and a narrower, long anterior proboscis <feeding appendage, also referred to as an introvert>/

Body regions are usually demarcated by changes in parapodial morphology; alternatively, body may be demarcated by specialised structures occurring at the anterior or posterior end of the body.

#55. Anterior end sucker on ventral surface <presence; represents modified first four segments, or using leech terminology segments I-VI, of which I and II are the prostomium and peristomium, respectively>/

1. absent/

2. present/

Leech segment counting follows Sawyer (1986: 54–66), viz. body is comprised of 34 segments (I–XXXIV; ie., counting the prostomium and peristomium as segments): six segments (I–VI) constitute the head, seven (XXVIII–XXXIV) constitute the tail (caudal sucker), and the intervening 21 segments (VII–XXVII) constitute the midbody. Therefore, considering the prostomium and peristomium are counted as segments, the anterior sucker is only comprised of 4 segments. Image

#56. <Anterior end sucker; form>/

1. clearly separated from rest of body/

2. not clearly separated from rest of body/

#57. <Anterior end sucker leading to; form of mouth>/

1. small mouth pore on ventral surface of sucker/

2. large mouth on ventral surface of sucker/

#58. Introvert <relative length>/

1. shorter than trunk/

2. about equal to trunk/

3. longer than trunk/

#59. Introvert hooks <on surface, often in circular rows; presence>/

1. absent/

2. present/

Introvert hooks are superficially similar to chaetae but are both arranged differently (often in circular rows) and structurally different (derived from the cuticle).

#60. Introvert papillae <presence>/

1. absent/

2. present/

#61. Trunk <surface appearance>/

1. smooth/

2. roughened by papillae or rounded skin bodies/

Strictly, the trunk is the segmented region of the body between the peristomium and pygidium, i.e., almost all of the body; however, the term as used here is only applied to sipunculans and echiurans to distinguish the body proper from the equally large introvert/proboscis. Trunk papillae (trp) occurring on any part of the trunk should be scored as state 2.

#62. Anterior extremity of trunk with hardened or calcareous structures <shield, cap, cone or knob; presence>/

1. absent/

2. present/

#63. Posterior trunk with shield <presence; hardened cuticular structure>/

1. absent/

2. present/

#64. Body, in life <transparency; light transmission in fixed specimens>/

1. translucent, gut visible/

2. opaque, gut usually not visible/

Some annelids are very thin (small and or dorsoventrally flattened) and light will pass through them, even as fixed specimens, enabling the gut and other internal organs to be readily seen when viewed under a microscope.

#65. Body pigmentation <presence, including base colour (but not white, yellow or pinkish which are common) and spots resulting from coloured epidermal glands>/

1. absent/

2. present/

Epidermal glands (eg) or pigment glands are regularly or irregularly arranged glands in the epidermal layer which may be brightly coloured (see Annelida Glossary). Worms that are white or pinkish are considered unpigmented. Pigmentation usually fades in preserved worms.

#66. Body pigmentation <colour and patterns>/

#67. Epidermis <appearance under stereo microscope; for Sipuncula and Echiura we scored this information under trunk surface>/

1. more-or-less smooth <may be shiny or dull finish>/

2. thick and rugose <sometimes on anterior body only>/

3. papillate <includes verrucate; papillae/verrucae irregularly or uniformly arranged, or restricted to venter and parapodia>/

4. tuberculate, with bumps of various sizes and arrangements/

5. with radial or transverse dorsal ridges/

6. tesselated/

Papillae and tubercles are epidermal sensory organs; their sizes, shapes and arrangement are often of systematic importance. The character concerns the macroscopic surface structures on the body wall, not structures attached to the body wall such as elytrae and parapodia.

#68. Bioluminescence <light-producing ability in living animals; presence>/

1. absent/

2. present/

Scored after Verdes and Gruber (2017)

#69. Tuberculae arrangement <transverse or longitudinal>/

1. in transverse rows/

2. in longitudinal rows/

#70. Dorsal intersegmental furrow <presence>/

1. absent/

2. present/

Intersegmental furrows (yellow) on segments 5–8; they vary from deep to poorly developed. Image source:

#71. Dorsal pores on mid-dorsal line <presence; EARTHWORMS only>/

1. absent/

2. present/

Dorsal pores (yellow) are small apertures in the midline of intersegmental grooves of the mid-anterior region of the body; they are used for respiration in earthworms.

#72. Dorsal body surface, appearance under compound microscope <applies only to meiofauna>/

1. smooth/

2. ciliated/

#73. Body surface with protective covering <gross appearance>/

1. absent/

2. as gelatinous sheath/

3. as scales (elytrae), dorsally/

4. as shield-like spines (paleae), dorsally/

#74. Body margin <form, when viewed from above>/

1. smooth, aside from parapodia/

2. cirrate, scalloped or irregular/

#75. Thoracic membranes <presence>/

1. absent/

2. present/

The thoracic membranes are a prominent membrane continuous with the peristomial collar (=ventral lappets) in Serpulidae, extending over some or all of the thorax and usually free dorsally (see Annelida Glossary). Not to be confused with the similar-looking flap-like vestimentum of some deep-sea Siboglinidae, which is derived from the pre-thoracic peristomium.

#76. Thoracic lobe-like dorsolateral expansion <presence; causing parapodia of first few segments to be displaced dorsally>/

1. absent/

2. present/

#77. Posterior <=caudal> sucker on ventral surface <presence; represents modified segments 26–32>/

1. absent/

2. present/

This character is for leeches only - All leeches possess a posteroventral sucker, formed by the last segments of the leech; it may be reduced in some species. Non-leech clitellates and non-clitellates lack a posteroventral sucker.

#78. Posterior sucker <shape>/

1. elliptical <and prehensile - able to grasp the substrate>/

2. circular/

A few leech taxa bear caudal auricles (see Annelida Glossary) on the fringe of the posterior sucker.

#79. <Posterior sucker> rays <presence>/

1. absent/

2. present/

#80. Anus <position>/

1. positioned at posterior body <either carried by pygidium or simple hole in body wall>/

2. positioned near anterior end, dorsally, near the introvert-trunk junction/

3. positioned near posterior end/

4. subterminal/

The position of the anus defines major annelid groups, for example: polychaetes and oligochaetes - at posterior end; Hirudinidae - near posterior end (just anterior to posterior sucker); sipunculans - near anterior end at base of introvert.

#81. Faecal groove <presence>/

1. absent/

2. present/

A groove on the ventral abdomen of tubiculous polychaetes running from the anus to the lateral midbody along which faecal pellets pass (see Annelida Glossary).

#82. Thoracic ventral glandular areas <presence>/

1. absent/

2. present/

#83. Thoracic ventral glandular areas <form>/

1. indistinct mid-ventral swelling <visibility enhanced by staining with methyl green>/

2. distinct mid-ventral shield-shaped swellings/

3. distinct paired ventrolateral swellings/

A ventral glandular shield is a glandular region, sometimes rugose, present as discrete pads on a few anterior segments or continuous along the ventral surface in some Terebellidae (see Annelida Glossary)

#84. Ventral groove <presence>/

1. absent/

2. present/

A ventral groove is a mid-ventral groove extending over most or all of the body, between two ridges of longitudinal ventral muscles in some Opheliidae (see Annelida Glossary).

#85. Ventrocaudal shield on posterior segments <presence>/

1. absent/

2. present/

A ventrocaudal shield is a bilaterally symmetrical brownish sclerotinised shield located ventrally posterior to segment 13 or 14 in Sternaspidae (see Annelida Glossary).

#86. Caudal region <form; POLYCHAETES ONLY>/

1. unmodified typical segments (but shorter and with reduced parapodia)/

2. an unsegmented tube <called and anal tube>/

3. very short, multisegmented <called an opisthosoma in Siboglinidae>/

4. short, few segments, mostly achaetous, with frilly lobes <called a scaphe in Pectinariidae>/

5. prominent achaetous sacrificial caudal region (at least in *Arenicola* and *Abarenicola*)/

#87. Multisegmented caudal region with <form>/

1. rows of uncini on each segment/

2. four peg-like chaetae in most segments/

3. long-handled hooks/

#88. Pygidium <presence; post-segmental structure marking the extreme posterior end of the body bearing the anus and often anal cirri>/

1. absent <anus a simple hole in body wall>/

2. present/

The presence of a terminal body region, the pygidium, in Annelida is widespread but in those few groups that lack segmentation (sipunculans and echiurans) it is lacking. Although clitellates have a pygidium in some sense, this term is not used among its workers and, especially for leeches, a pygidium in the sense of the terminal ring in polychaetes is not visible.

#89. Pygidium <form; LEECHES EXCLUDED>/

1. simple lobe <note if tapered or funnel-like; or if anus is pointing dorsally, ventrally or posteriorly>/

2. plate-like/

3. bilobed/

4. with multiple digitate lobes/

5. deeply cleft forming two large feet/

6. membranous <often foliose>/

7. anal flap or ligule/

8. elongate hooded anal tube/

State 1: See Annelida Glossary terms 'Anal funnel' and 'pygidial funnel' State 2: See Annelida Glossary terms 'Anal plaque', 'anal plate' State 5: See Annelida Glossary term 'locomotory appendage'.

#90. Pygidial appendages <presence; includes cirri and other obvious caudal appendages, but not papillae or cilia which are often very small and numerous>/

1. absent/

2. present/

#91. Pygidial appendages <form; POLYCHAETES ONLY>/

1. single medial cirrus/

2. one pair of cirri/

3. one pair of cirri and single medial papilla/

4. three cirri/

5. four cirri/

6. more than four cirri/

7. as lobes/

When multiple pygidial cirri are present, they are often referred to as dorsal pygidial cirri and ventral pygidial cirri, depending on their position (see Annelida Glossary).

#92. Elongate proboscis <presence; form - assessable if proboscis is in the non-retracted state>/

1. absent/

2. present/

The proboscis is the eversible part of the pharynx used for feeding (see Annelida Glossary). Most annelids have a proboscis that extends slightly beyond the head. A few annelid groups (sipunculans and echiurans) have an elongated proboscis, which is as long as the rest of the body. NOTE: the elongated proboscis must be everted in order to see its associated characters. #93. <Elongated proboscis; retractability>/

1. retractable and tube-like <polychaetes, sipunculans; some leeches>/

2. non-retractable and ribbon-like or spatulate <echiurans>/

#94. Non-retractable elongated proboscis <form of tip>/

1. truncate/

2. bifid/

#95. Non-retractable elongated proboscis <length>/

1. short and scoop-like/

2. very long <many times longer than trunk>/

#96. Discrete head <presence or absence of a recognisable 'head' rather than a region comprising tentacles, a proboscis or radiolar crown; aligns with Rouse & Fauchald 1997>/

1. absent/

2. present/

A 'head' refers to the prostomium plus peristomium (see Annelida Glossary). A discrete head refers to an externally recognisable anteriormost structure with or without eyes and antennae. A score of 'absent' was given if a head is not externally recognisable, for example in many polychaetes whose prostomium is incorporated into various types of feeding organs. Prostomia are often difficult to see - although presegmental, sometimes they can be surrounded by anterior segments and appear postsegmental. In oligochaetes, heads are a simple lobe with no attached appendages; in leeches the prostomium is part of the anterior sucker (leech workers refer to the prostomium as segment I). Interpretation of this character follows Purschke et al. (2014).

#97. Discrete head <whether simple and lobe-like, adorned with various structures, or sucker-like>/

1. lobe-like without appendages <may have cilia>/

2. complex in shape bearing appendages <papillae, palps, tentacles or antennae>/

3. as a disc-shaped sucker <only leeches; the sucker comprises the prostomium and peristomium>/

asc = anterior sensory cilia (see Annelida Glossary)

#98. Head <retractable into anterior segments or not>/

1. not retractable/

2. retractable into anterior segments/

#99. Radiolar crown <presence>/

1. absent/

2. present/

The feathery radiolar crown is both a feeding appendage and the primary site of respiration in Sabellidae (Tilic et al. 2021).

#100. <Radiolar crown with> internal 'skeleton'/

1. absent/

2. present/

The internal 'skeleton' are actually ladder-like vacuolated cells that support the radioles. This character is difficult to observe, and therefore not used in the linear key. It is output in the descriptions and, especially, the Nexus file, as it is potentially valuable for phylogenetic analysis. Coding of this character follows Fitzhugh (1989) and Capa et al. (2021, Table 1).

#101. <Radiolar crown> modified radioles <presence; form>/

1. absent/

2. present, as a singular opercular peduncle/

3. present, as a pair of ventral filamentous appendages/

The radiolar crown often has one or more modified radioles. For example, an opercular peduncle is a modified branchial pinnule with an operculum at the tip, used to stopper the tube of the worm; the paired ventral filamentous appendages are vascularised appendages located at the ventral margin of each branchial lobe in some Fabriciidae; and dorsal lips are paired ciliated lappets on the dorsal edge of the mouth in Fabriciidae, Sabellidae and Serpulidae (see Annelida Glossary).

#102. Prostomium <basic shape>/

1. conical, tapering to slender tip/

2. bluntly conical <rounded tip>/

3. triangular to trapezoidal (narrow end posteriorly)/

4. pentagonal to quadrangular <sharply angular>/

5. rounded to oval <anteriorly truncate>/

6. narrow, keel- or ridge-shaped/

7. t-shaped, wide end anteriorly <resembling horns>/

8. flattened, shovel-shaped/

9. hood-like, covering the tentacles dorsally/

#103. Prostomium anteriorly <form>/

1. incised/

2. with four identical small cirriform projections/

3. with an anterior tentacle-like projection (called a 'palpode' in polychaetes and 'proboscis' in oligochaetes)/

The small cirriform projections (state 3) have been called prostomial appendages or paired antennae and palps (see Annelida Glossary); the palpode of oligochaetes is referred to as a tentacle or proboscis by oligochaete workers.

#104. <Prostomium posteriorly; shape in relation to peristomium; OLIGOCHAETES ONLY>/

1. epilobic (tongue of prostomium partly divides peristomium)/

2. prolobic (prostomium demarcated from perisomium without a tongue)/

3. tanylobic (tongue completely divides peristomium)/

4. zygolobic (prostomium not demarcated)/

The character is useful only for oligochaetes. Epilobic and tanylobic prostomia have longitudinal grooves, which are lacking in the other two forms. All truly aquatic oligochaetes have zygolobic prostomia; terrestrial oligochaetes have various forms. Image source:

#105. Ommatophores <presence>/

1. absent/

2. present/

Ommatophores are projecting structures supporting the eyes (see Annelida Glossary)

#106. Ommatophores <form>/

1. as low ocular mounds/

2. as stalk-like ocular peduncles/

#107. Facial tubercle <presence; situated anteroventral to the prostomium>/

1. absent/

2. present/

Facial tubercles are raised pillow-like structures anterior to and beneath the prostomium (see Annelida Glossary). The presence of a facial tubercle was used as a family-defining character for Aphroditidae by Hutchings & McRae (1993) and Gonzalez et al. (2018), but Pettibone (1969) was explicit that a facial tubercle was absent in Heteraphrodita. When present, the facial tubercle is usually well-developed and conspicuous but it is less developed in some aphroditids. Hutchings & McRae (1993) used development and ornamentation (the surface may be rugose or papillate) of the facial tubercle to distinguish species within Aphroditidae but were unsure if preservation artefacts are a compounding factor (Robin Wilson pers. comm., June 2021). The small papilliform structure below the median antenna in Polynoidae is also considered a facial tubercle.

#108. Frontal lips <presence; situated anteroventral to the prostomium>/

1. absent/

2. present/

Frontal (or dorsal) lips look like small palps; they are not readily differentiated externally.

#109. Eyes on head <presence; head includes the prostomium, peristomium and anterior segments that are fused with the prostomium and peristomium; also includes feeding appendages>/

1. absent/

2. present/

For the purpose of this dataset we are only considering pigmented eyes; unpigmented photoreceptor organs occur in some polychaete annelids (G. Purschke pers. comm.), but they are invisible without specialised microscopy (TEM) and staining. Eyes on the head are shown in the upper images and lower left image; eyes in other parts of the body are shown in lower middle (parapodial eyes) and lower right (pygidial eyes) images. Most leeches are scored as having eyes on the head, which is considered to be their anterior sucker comprised of six fused segments (I–VI) of which I, II represent the annelid prostomium and peristomium, respectively.

#110. <Head eyes; number>/

1. one pair/

2. two pairs/

3. three pairs/

4. numerous, unpaired <may be arranged in two patches>/

5. five pairs/

6. four pairs/

#111. Head eyes <form; presence of a refractive body or lens>/

1. simple pigmented cups <ocelli>/

2. compound, with lenses/

We take a loose definition of what constitutes a compound eye (true compound eyes probably only occur in some Sabellida; Rouse et al. 2022); any eye that appears to have a clear central area (lens) is considered to be compound.

#112. Postcephalic eyes <presence; includes pigmented subdermal 'brain eyes' in polychaetes>/

1. absent/

2. present/

In this image, examples of postcephalic eyes are shown in the lower images. The upper images show cephalic eyes.

#113. <Postcephalic eyes; position relative to surface>/

1. epidermal/

2. subepidermal <on posterior brain lobes>/

#114. <Number of annuli with postcephalic eyes; number annuli with paired eyespots>/

annuli each with a pair of eyes/

Includes the ‘haemadipsine’ ocular arch of many blood-feeding leeches

#115. Eyes on trunk <presence>/

1. absent/

2. present/

trunk eyes are also referred to as 'lateral segmental eye spots' in the Annelida Glossary

#116. Trunk eyes <position>/

1. on dorsal surface/

2. on ventral surface/

3. on lateral body/

#117. Caudal eyes <includes pygidium; posterior sucker etc; presence>/

1. absent/

2. present/

#118. Prostomial antennae <presence; aligns with Rouse & Fauchald 1997>/

1. absent/

2. present/

#119. Prostomial antennae <position; aligns with Rouse & Fauchald 1997>/

1. median one only/

2. paired, lateral/

3. median and paired laterals/

#120. Prostomial antennae <form>/

1. unarticulated <smooth>/

2. articulated/

3. consist of basal ceratophore and distal ceratostyle/

#121. Buccal tentacles <presence; does not include the feathery radioles of branchial crowns or frilly membranes around mouth>/

1. absent/

2. present/

'Buccal tentacle' refers to the multiple tentacle-like feeding appendages of polychaetes and sipunculans; also called oral filaments (see Annelida Glossary). Although they are innervated in the same way as paired palps, they have only recently been referred to as palps in the literature. Caution: does not include the feathery radioles of branchial crowns or frilly membranes around mouth.

#122. Buccal tentacles <position in relation to mouth; oral disc in sipunculans>/

1. arising inside mouth/

2. arising from one side of mouth <may be on a distinct membrane, oral disc, or encircling nuchal organ as in some sipunculans>/

3. surrounding perimeter of mouth/

4. arising anteroventrally (mouth absent)/

5. lining inner margin of opercular lobes/

#123. Buccal tentacles <form>/

1. smooth/

2. pinnulated/

3. grooved/

4. ciliated/

We have identified four main types of buccal tentacles. Other variations occur including a grooved type with expanded tips (Polycirinni) and short vs long grooved tentacles (eg. *Loimia* species), but these other types largely represent subfamily variation.

#124. Palps <presence>/

1. absent/

2. present/

Palps are paired structures located on the prostomium or peristomium (see Annelida Glossary) and, rarely, may appear to come from the first few segments. Following most literature, we treat buccal tentacles (=oral filaments) separately, even though they have been shown to be innervated in the same way and thus homologous.

#125. Palps <location>/

1. anterodorsal/

2. anteroventral/

3. frontal/

4. lateral/

5. ventral/

The images show two of the most common locations of palps: anterodorsal palps are located dorsally on the posterior part of the prostomium and resemble the more centrally positioned antennae; anteroventral palps are located anterior part of the prostomium and are thicker than the antennae.

#126. Palps <basic type>/

1. grooved (usually) feeding type/

2. tapering (usually) sensory type/

Sensory palps are tapering structures located anteroventrally, more or less round in cross-section, frequently covered with cilia. Feeding (or grooved) palps are located dorsally, and have a longitudinal groove lined by cilia.

#127. Feeding palp <appearance>/

1. longitudinally grooved/

2. papillated/

3. not grooved or papillated/

#128. <Sensory palp; appearance>/

1. unarticulated/

2. bi-articulated/

3. multiple articulations/

Sensory palps include 'palpal antenna', the lateral-most pair of appendages located on the prostomium in Eunicidae, Onuphidae and related families; they are very similar in form to true antennae but are innervated differently (see Annelida Glossary). Biarticulated palps, may not articulate in a functional sense but they are divided into two parts, a basal palpophore and a distal palpostyle.

#129. Caruncle <presence>/

1. absent/

2. present/

A caruncle is a raised fleshy projection extending posteriorly from the prostomium for one to a few segments (see Annelida Glossary).

#130. Nuchal organs <presence; aligns with Rouse et al. 2022>/

1. absent/

2. present/

Nuchal organs are chemosensory structures of polychaetes situated posterolaterally on the prostomium. They take various forms including folds or flaps (nuchal fold/flap), papillate (nuchal papilla or occipital papilla) (see Annelida Glossary), or simple paired low projections, which are difficult to see under light microscopy.

#131. Nuchal organs <general location and form>/

1. single antenna-like projection from posterior prostomium <also called nuchal antenna, nuchal papilla or occipital antenna>/

2. paired low projections from posterolateral prostomium/

3. projecting considerably from prostomium <either three lobes or adjacent a mediodorsal caruncle>/

4. on dorsal side of anteriorly projecting introvert organ <unpaired; two to four-lobed cushion>/

5. unpaired caruncle or nuchal fold/

The various shapes and locations of nuchal organs include a single small antenna-like projection on the posterior prostomium, which has been referred to under different names in polychaete, for example, occipital antenna (Spionidae) and nuchal papilla (Phyllodocidae). They are unlikely to be homologous structures, as evidenced in some Phllodocidae which can have both typical paired dorsolateral nuchal papillae and a nuchal papilla.

#132. <Paired nuchal organs; form; may be barely visible or obvious posterior projections from the head>/

1. indistinct dorsolateral ciliated patches <not clearly visible with light microscope>/

2. posterior projections/

3. posterolateral ciliated bulbs/

4. club-shaped/

5. patches adjacent mid-dorsal hump <median organ>/

#133. Peristomium <visibility in adults>/

1. not visible <in adults; either because it is reduced or incorporated into a head structure>/

2. visible/

The peristomium is the pre-segmental region behind the prostomium that includes the mouth; it is referred to as segment 1 (or segment I) in oligochaetes and segment 2 (or metamere II) in leeches. The peristomium is extremely variable in form ranging from a simple ring, ring with collars, and lips, and it may be fused with anterior segments or reduced to an incomplete ring in polychaetes. In leeches the peristomium is always incorporated into the anterior sucker, and thus not visible.

#134. Peristomium <form; sometimes called segment 1 - see notes; aligns with Rouse & Fauchald 1997>/

1. a single ring (no collars)/

2. a single ring, collar-like/

3. a double ring/

4. an expanded glandular ring <frenulum>/

5. an expanded, elaborately collared ring <vestimentum>/

6. expanded into well-developed upper and lower lips/

The peristomium is highly variable in Annelida. Three common forms of peristomia are shown in blue shading in the first figure. Some annelids lack a peristomium or, as in the top left image, the peristomium has disappeared by adulthood. A single ring with or without collars and lobes (states 1, 2) is found in many polychaete families; a double-ring peristomium (state 3) is referred to as a 'biannulate peristomium' (see Annelida Glossary; note Aberrantidae may have a triannulate peristomium, but it is coded here as biannulate). In Terebellidae and Trichobranchidae the peristomium is expanded into feeding organs, the upper and lower lips (state 6). Other expanded types of peristomia include the glandular and collared rings of deep-sea Siboglinidae (states 4, 5); the bridle is coloured yellow (see Annelida Glossary).

#135. Peristomial cirri <presence>/

1. absent/

2. present, 1 pair/

Peristomial cirri are paired, elongated cirri on the peristomium of polychaetes (see Annelida Glossary).

#136. Gut <presence>/

1. absent/

2. present/

#137. Gut <basic form>/

1. more-or-less straight, lacking side branches/

2. straight with side branches <blind-ending lateral prostrusions; includes multiple folds, caecae or diverticulae>/

3. straight except for a large midbody loop/

4. coiled/

#138. Foregut <type of pharyngeal pad (stomodaeum)>/

1. a non-muscular axial pharynx/

2. a muscular axial pharynx/

3. a muscular ventral pharynx/

4. a muscular dorsal pharynx/

5. without a distinct ventral or axial pharynx/

This character is included for the annelid specialist. The type of foregut is established through histological sectioning and examination of musculature. A muscular foregut is called a pharynx (see Annelida Glossary). Coding of Rouse and Fauchald (1997) for most taxa and Rouse et al. (for leeches).

#139. Muscular axial pharynx <form; LEECHES ONLY>/

1. not rotated (euthylaematous)/

2. ridges of pharynx rotated 60 degrees to the right (strepsilaematous)/

Applies only to leeches

#140. <Pharynx> extends from <segmental extent; LEECHES only>/

#141. Pharynx jaws <presence; includes the muscular ridges of some leeches; see Notes>/

1. absent <agnathous>/

2. present <gnathous>/

3. present, leech-type (either soft-muscular or horny)/

Jaws are present only in some polychaetes and leeches. Although some leeches lack true horny jaws, instead of having soft muscular jaws, their placement in the buccal cavity is the same as horny jaws. Also, because soft muscular jaws, like horny jaws may have associated teeth-like structures, we are regarding all leeches as having jaws.

#142. <Pharynx jaws; form; more or less follows coding of Rouse and Fauchald (1997) but we treat axial and ventral jaws as one character, not two; also the single tooth or fused teeth of Syllidae are not considered jaws as do Rouse and Fauchald>/

1. one pair of lateral jaws/

2. two pairs of jaws <dorsal and ventral positions>/

3. multiple jaw elements of different shapes and sizes/

4. one pair of dorsal-ventral jaws/

Multiple jaw elements (state 3) comprise both macrognaths and micrognaths (see Annelida Glossary). The image in the upper left shows a polychaete lacking jaws.

#143. <Leech-type jaws: form and number of muscular-type jaws>/

1. two (duognathous) muscular jaws/

2. three (trignathous) muscular jaws arranged in a triangle/

#144. <Leech-type jaws with> denticles <presence; includes stylets and other teeth-like structures>/

1. absent <astichodont>/

2. present/

Many leeches have mouthpart structures referred to in the literature as jaws, or soft muscular jaws. We do not know how they relate to polychaete jaws, but are treating them here as homologous.

#145. <Leech-type jaws; form of denticles; including pseudognaths, cutting plates and stylets>/

1. one row of teeth <monostichodont; found in blood-feeding leeches>/

2. two rows of teeth <distichodont; typical of predaceous leeches>/

3. as cutting plates/

4. paired, fine teeth <stylets>/

5. series of soft teeth <pseudognaths>/

Teeth form and arrangement in leeches varies considerably; in general, single rows of teeth (monostichodont) are found in blood-feeding leeches and two rows of teeth (distichodont) are typical of predaceous leeches.

#146. Paired jaws <form>/

1. plate-like/

2. fang-like <includes any piercing type including stylets>/

3. fang-like, with a basal support <carrier>/

#147. Multiple jaws <basic types>/

1. comprising ventral mandibles and dorsal maxillae/

2. comprising many dorsal jaw pieces (micrognaths) arranged in ring and usually pair ventral toothed plates (macrognaths)/

#148. Multiple jaws <form of jaw elements>/

1. 4–5 pairs of toothed plates in parallel rows <i.e. prionognath arrangement>/

2. 4–5 (right) and 4–6 (left) toothed plates in a semicircle <i.e. labidognath arrangement>/

3. 2 pairs of toothed plates plus many small free denticles arranged in longitudinal rows <i.e. ctenognath arrangement>/

#149. Pharynx maxillae <symmetry>/

1. asymmetrical/

2. symmetrical/

#150. Superior row <of maxillae; number of free small denticles>/

1. more than 8 pairs/

2. fewer than 8 pairs/

#151. Superior base plates <of maxillae; presence, fusion with each other>/

1. present, fused to each other posteriorly/

2. present, free of each other/

3. absent/

#152. Superior base plates <fusion of superior base plates and maxillary carrier>/

1. fused with maxillary carrier, K-shaped/

2. fused with maxillary carrier, not K-shaped/

3. not fused with maxillary carrier/

#153. Maxillary carriers <relative length>/

1. shorter than combined length of maxillae/

2. longer than combined length of maxillae/

3. similar in length to combined length of maxillae/

#154. Pharynx teeth <presence and type of tooth-like armature>/

1. absent/

2. present, as a single tooth <usually hyaline>/

3. present, as a trepan (fused teeth)/

4. present (=paragnaths), arranged in distinct areas/

The states, 'with a single tooth' and 'with a trepan' are shown on the same image, as they may occur together. More often one or the other will be present. In Nereididae the teeth are called paragnaths (see Annelida Glossary).

1. absent/

2. present/

The proboscis (or buccal cavity) is the eversible part of the muscular foregut (=pharynx) (see Annelida Glossary). It may be protrusible or not; it is a useful character for leeches and polychaetes, but not for oligochaetes

#156. <Proboscis; eversible part of pharynx; surface form>/

1. smooth/

2. with papillae in subterminal position/

3. with transverse ridges of thick cuticular lamellae/

4. with hardened (sclerotised) papillae of different structures <proboscideal organs>/

In addition to jaws, some groups of leeches have structures anterior to them that appear to be similar to polychaete denticles - these are treated here as proboscis features, although whether they are homologous or not is unknown. In polychaetes, proboscideal papillae may be subterminal, located on the barrel of the pharynx, or terminal (a ring of terminal or oral ring papillae) (see Annelida Glossary)

#157. <Proboscis; eversible part of pharynx; papillae arrangement>/

1. irregularly arranged/

2. in distinct rows/

3. in distinct areas/

Proboscideal papillae are best observed when the proboscis is everted as shown in the images; distinguishing between 'distinct rows' and 'distinct areas' may be difficult - in the latter, the areas are usually separated by non-papillate areas so it is actually more obvious than shown in the image on right where the areas abut each other.

#158. <Proboscis; eversible part of pharynx> distal ring of papillae <presence>/

1. absent/

2. present/

#159. Pharynx proventricle <presence>/

1. absent/

2. present/

The proventricle is a highly muscularised part of the posterior foregut (see Annelida Glossary). When present, it is usually visible through the body wall. It is only present in two families of polychaetes (Syllidae and Sphaerodoridae) and muscle differences between them suggest that they are not homologous.

#160. Pharynx dorsolateral ciliated folds <presence>/

1. absent/

2. present/

#161. Crop <presence; LEECHES ONLY>/

1. absent/

2. present/

The crop is an enlargement of the gut used to store food. It immediately precedes the gizzard (if present)

#162. Gizzard <presence; OLIGOCHAETES and LEECHES ONLY>/

1. absent/

2. present/

The gizzard is a thick-walled muscular region of the posterior oesophagus between the oseophagus (or crop, if present) and the intestine of earthworms and some leeches involved in the grinding of food and soil particles.

#163. <Gizzard location> segment/

#164. Calciferous glands <presence>/

1. absent/

2. present/

Calciferous glands are found in certain earthworms; they are thought to excrete calcium by secreting granules of calcium carbonate that are transformed into calcite crystals in the intestine to aid digestion, although other ideas have been proposed. Coding is incomplete for this character.

#165. <Calciferous glands> in segment <location>/

#166. Intestinal typhlosole <presence; oligochaetes only>/

1. absent/

2. present/

#167. <Intestinal typhlosole; form; oligochaetes only>/

1. formed from all layers of the intestine/

2. formed from the inner (epithelial) layer only of the intestine/

As the form of the typhosole is strongly influenced by worm size (Marchan et al. 2016), we have refrained from including further characters, such as number of its lamellae.

#168. Caeca of foregut <form; presence of foregut lateral expansions = caeca or diverticula; SCORE FOR CLITELLATES AND SIPUNCULA>/

1. absent/

2. present/

Caeca (also called an oesophageal caeca or oesphageal glands; see Annelida Glossary) are paired blind sacs, or diverticula, arising from the gut after the posterior foregut.

#169. Caeca of midgut (=posterior crop caeca) <form; presence of midgut lateral expansions = caeca or diverticula; SCORE FOR CLITELLATES AND SIPUNCULA>/

1. absent/

2. present/

Caeca (also called an oesophageal caeca or oesphageal glands; see Annelida Glossary) are paired blind sacs, or diverticula, arising from the gut after the posterior foregut. The crop of leeches is considered here to be part of the mid gut.

#170. Caeca of hindgut <form; presence of hindgut lateral expansions = caeca or diverticula; SCORE FOR CLITELLATES AND SIPUNCULA>/

1. absent/

2. present/

Caeca (also called an oesophageal caeca or oesphageal glands; see Annelida Glossary) are paired blind sacs, or diverticula, arising from the gut after the posterior foregut.

#171. Circulatory system <presence>/

1. absent/

2. present/

Most annelids have a circulatory system with dorsal and ventral longitudinal vessels, but some leeches have lost their vessels and instead have a system of coelomic sinuses to circulate haemoglobin

#172. Heart bodies <presence>/

1. absent/

2. present/

The heart body (=dorsoventral heart) is an enlarged lateral blood vessel connecting the main dorsal and ventral vessels in the anterior body that is observed to pulsate. In fabriciid polychaetes similar structures are found in the main radioles of the radiolar crown, which is both a respiratory and feeding organ.

#173. <Heart body; segmental position; oligochaetes only>/

#174. First segment <appearance of first true annulus; score for all taxa except LEECHES>/

1. chaetous <with parapodia and/or chaetae>/

2. tentaculate <usually much shorter than following segments>/

3. achaetous <ie., lacking parapodia, chaetae or tentacular cirri; but may bear lateral lobes or dorsal gills>/

A segment is a serially repeated unit that comprises the body of a worm, which is often separated internally by septa (see Annelida Glossary). Unfortunately, how they have been designated and counted in Annelida differs depending on the group. In polychaetes, Arabic numbers have mostly (see exception below) been used (S1, S2, S3 etc.); in leeches and oligochaetes, Roman numerals (SI, SII, SIII ...) have been preferred. In polychaetes segment numbering starts after the presegmental prostomium and peristomium, in oligochaetes the peristomium is counted as the first segment while in leeches the prostomium and peristomium have been counted as the first two segments. In the leech literature segments including the prostomium and peristomium have been referred to as 'somites'. In AnniKey, for most characters we have maintained the historical numbering sequence of each group in order to facilitate comparisons with previous studies, ie., Roman numerals for oligochaetes and leeches, Arabic numerals for polychaetes. It should be mentioned that some polychaete authors have used a combined system of Roman numerals and Arabic numerals either to distinguish segments from chaetigers (eg. Reuscher, Fiege and Wehe (2009) Ampharetidae), or segments from annuli (Wells (1959) - Arenicolidae) but this has not been implemented here. Practical tip: the first segment in polychaetes and oligochaetes is usually a complete ring around the body, in contrast to the parasegmental peristomium, which is often incomplete.

#175. Second segment <appearance of second annulus, which may bear parapodia, cirri or chaetae; POLYCHAETES ONLY>/

1. chaetous <with parapodia or chaetae; may be reduced in size>/

2. tentaculate/

3. achaetous <lacking parapodia and/or chaetae>/

4. with a very long region comprising most of the body <largely achaetous; may bear a few rows of uncini>/

#176. Tentacular cirri <presence; tentacular cirri are cephalised cirri that resemble parapodial cirri except they surround the head and are longer; aligns with Rouse & Fauchald 1997>/

1. absent/

2. present/

Tentacular cirri are dorsal and/or ventral cirri of parapodia that have been modified through cephalisation of anterior segments (see Annelida Glossary). They are called anterior dorsal (ventral) cirri in Rouse and Pleijel (2001) and Rouse et al. (2022). Not to be confused with peristomial cirri which is found on the peristomium. Practical tip: tentacular cirri are often more elongate than the dorsal and ventral cirri of more posterior parapodia.

#177. Tentacular cirri <number pairs>/

pair(s)/

Many families of polychaetes have paired dorsal and ventral cirri on parapodia of cephalised segments; often more elongate than those of more posterior parapodia; see 'ventral tentacular cirri' and 'dorsal tentacular cirri' in Annelida Glossary

#178. <Tentacular cirri> arise <segment number>/

1. on a single segment <segment 1 or 2>/

2. over two or more segments <segments 1–5>/

#179. <Tentacular cirri> internal aciculae <presence>/

1. absent/

2. present in at least some cirri/

The dorsal tentacular cirri are coloured yellow (see Annelida Glossary) and the internal aciculae are the rod-like structures below.

#180. First chaetiger <general appearance; POLYCHAETES ONLY>/

1. without external chaetae <aciculae present>/

2. with notochaetae only/

3. with neurochaetae only/

4. with both notochaetae and neurochaetae/

A chaetiger is a segment bearing chaetae; they are present only in polychaetes and oligochaetes, although oligochaete workers don't use this term (see Annelida Glossary).

#181. First chaetiger parapodia <relative length; POLYCHAETES ONLY>/

1. similar in length or slightly shorter than subsequent ones/

2. very elongated/

#182. First chaetiger parapodia <orientation; POLYCHAETES ONLY>/

1. anteriorly directed and wrapping around head/

2. more-or-less laterally directed and free from head/

#183. First chaetiger chaetae <form of chaetae; POLYCHAETES ONLY>/

1. similar in orientation, length and thickness to other chaetae/

2. projecting obliquely, distinctly thicker and more shiny than subsequent ones (=paleae)/

3. slender and elongate, forming cage (or basket) around head/

Paleae are called 'cephalic paleae' in the Annelida Glossary. Cage-like chaetae are called 'cephalic cage chaetae' in the Annelida Glossary.

#184. Parapodia <lateral body appendages; including low ridge-like structures; presence over at least some of body>/

1. absent/

2. present/

Parapodia are characteristic of polychaetes. They mostly comprise one or two fleshy lateral projections from a body segment, usually bearing chaetae; however, sometimes the chaetae emerge directly from the body wall. Clitellates lack parapodia and have very few chaetae (see Annelida Glossary).

#185. Parapodia <form, generally>/

1. uniramous <one-branched>/

2. biramous <two-branched>/

Uniramous parapodia have only one lobe present, usually the neuropodium. Biramous parapodium have two branches: notopodium (dorsal; in red)) and neuropodium (ventral) (see Annelida Glossary). Note that we consider parapodia uniramous, even though they might have remnants of notopodia (eg. a few notochaetae and/or a dorsal cirrus) provided that notopodial lobe is absent. Note that some authors refer to this as semi-biramous.

#186. <Biramous or uniramous> parapodial lobes <form of lobes>/

1. absent or very low/

2. prominent/

Biramous parapodia may have poorly developed parapodial lobes or well-developed lobes; top image shows a uniramous parapodium for comparison.

#187. Notopodial lobes <form>/

1. represented by at least one chaetal lobe <not modified>/

2. elongate, ending in rounded lappet/

3. large, fusiform adorned with sensory hairs/

4. slender, flask- or spindle-shaped/

5. low lateral ridges (tori) <only occur posteriorly>/

6. long dorsal ridges/

7. absent <though dorsal cirrus may be present>/

#188. Neuropodial lobes <form>/

1. represented by at least one chaetal lobe <not modified>/

2. elongate, ending in rounded lappet/

3. large, fusiform adorned with sensory hairs/

4. slender, flask- or spindle-shaped/

5. low ridges (tori) <may occur only in part of body>/

6. absent/

A torus is the development of a parapodium (usually neuropodium) into a raised ridge-like structure, often with hooks or uncini (see Annelida Glossary).

#189. Interramal fleshy process <presence>/

1. absent/

2. present/

Also referred to as an interramal branchia, interramal cirrus or interramal papilla (see Annelida Glossary); likely to have different functions including as respiratory organs and lateral organs (sensory receptors).

#190. Interramal fleshy process <form>/

1. cirrus-like/

2. papilla-like/

#191. Lateral organs <presence>/

1. absent/

2. present/

Lateral organs are lateral papilla-like sensory organs; they are also referred to as an interramal papilla (see Annelida Glossary).

#192. Dorsal cirri <presence; includes both cirriform and flattened types>/

1. absent/

2. present/

Dorsal cirri are slender cirriform to leaf-like structures that lie just above the parapodia. Scale-like elytrae are a modified type of dorsal cirrus (see Annelida Glossary for more information on elytrae). Dorsal cirri may be confused with simple branchiae - compare images shown in branchial characters.

#193. Dorsal cirri <form>/

1. more-or-less cirriform <may be smooth, wrinkled or annulated>/

2. flattened and foliaceous/

3. small, articulated and flask-shaped/

Dorsal cirri are slender cirriform to leaf-like structures that lie just above the parapodia; they are often absent in worms that do not have well-developed parapodia (image upper left); they may have a swollen base called a cirrophore - not shown (see Annelida Glossary).

#194. Elytra <structure>/

1. with raised concentric rings/

2. with lateral pouches/

3. with a tuberculated pentagonal or hexagonal pattern/

4. with papillae, tubercles or smooth/

#195. Elytra, post segment 7 <arrangement>/

1. occur on every other segment from segment 7 to end of body/

2. occur on every other segment from segment 7 to midbody, then present on every segment, or absent, to end of body/

In the alternate arrangement (left image and anterior end of right image), there are two pairs parapodia per elyral segment; in the one-to-one arrangement (right image, posterior end) there is one pair of parapodia per one elytral segment.

#196. Ventral cirri <presence; includes both cirriform and flattened types>/

1. absent/

2. present/

The ventral cirrus is normally situated on the lower edge of the neuropodial lobe (see Annelida Glossary).

#197. Ventral cirri <presence; form>/

1. cirriform or tapering/

2. foliaceous/

3. small, articulated and flask-shaped/

#198. Branchiae <presence; here taken to mean any outgrowths or extensions of the coelomic system from the body wall; may contain a loop of blood vessels (so are usually red) and bands or tufts of cilia; includes the feathered/pectinate and digitiform branchiae of polychaetes and the pulsatile vesicles of marine leeches>/

1. absent/

2. present/

Branchiae (=gills; respiratory organs) are common in many polychaetes but are less common in clitellates (oligochaetes and leeches); terrestrial clitellates lack branchiae. Branchiae that occur on or just dorsal to the parapodia may be difficult to distinguish from parapodial cirri. The easiest way to distinguish them is by observing a living specimen - branchiae will usually appear red (rarely green) due to the circulating blood. Branchiae often have a more complex shape including basal stalks, branching, or flattened and leaf-like. The pulsatile vesicles on lateral body margins of marine leeches (Piscicolidae) are not strictly branchiae but function as such.

#199. <Branchiae position; segmental position - dorsal or lateral>/

1. arise from lateral body <from parapodia, if present>/

2. arise from dorsal body/

#200. <Branchiae distribution along body>/

1. occur first few segments/

2. occur on mid-body segments/

3. occur near posterior end/

4. occur adjacent to terminal segment or pygidium <= perianal gills>/

#201. Lateral branchiae <form>/

1. digitiform <may be one or more pairs per segment/parapodium>/

2. branching/

3. single tuft per parapodium/

4. several tufts per parapodium/

5. recurved cirrus/

Lateral branchiae (also called parapodial branchiae, see Annelida Glossary) may be confused with dorsal cirri in polychaetes. Lateral branchiae are usually more elaborate - branched or tufted - but may also be simple. Simple types may be smooth, pinnate or have various other surfaces. Identification tip: Simple parapodial branchia resemble dorsal cirri; however, in live specimens the vascularised branchiae will be coloured red (rarely green), and the dorsal cirri will typically take the colour of the rest of the parapodia. Note: small parapodial 'digitiform' branchiae are sometimes referred to as 'ctenidia' (see Annelida Glossary).

#202. Dorsal branchiae <basic form>/

1. branching <often from a central stalk>/

2. lamellate <often from a central stalk>/

3. multiple filaments arising from a central stalk/

4. simple filaments each arising directly from body wall/

Dorsal branchiae take a variety of forms. They arise immediately dorsal to the parapodia or more centrally on the dorsal body surface, either singly, one on each side of the segment, or in a cluster united by central stalk. Filamentous types may be smooth, pinnate or otherwise (eg., pinnate branchia, Annelida Glossary).

#203. Chaetae <presence; does not include internalised aciculae>/

1. absent/

2. present <anywhere on body>/

Chaetae (or setae, as oligochaete taxonomists say) are characteristic epidermal features of most polychaetes (absent in a few; see image) and oligochaetes (also rarely absent); they are lacking in leeches, Branchiobdellidae and sipunculans.

#204. Chaetae <number per bundle, fascicle or row; SCORED ONLY FOR OLIGOCHAETES>/

1. more than two per bundle/

2. one per bundle/

3. two per bundle/

#205. Chaetae first appear on <first segmental occurrence; dorsal and ventral bundles are scored separately for oligochaetes>/

1. first segment after peristomium/

2. second segment after peristomium/

3. third segment after peristomium/

4. fourth segment after peristomium/

5. fifth segment after peristomium/

6. sixth segment after peristomium/

States are expressed in this way to make the data comparable across all Annelida (counting system for polychaetes, oligochaetes and leeches differs)

#206. Dorsal bundle chaetae first appear on <first segmental occurrence; OLIGOCHAETES ONLY>/

1. first segment after peristomium/

2. second segment after peristomium/

3. third segment after peristomium/

4. fourth segment after peristomium/

5. fifth segment after peristomium/

6. sixth segment after peristomium/

#207. Ventral bundle chaetae first appear on <first segmental occurrence; OLIGOCHAETES ONLY>/

1. first segment after peristomium/

2. second segment after peristomium/

3. third segment after peristomium/

#208. Chaetae <brittleness; composition>/

1. chitinous with scleroprotein coating, flexible/

2. calcareous, brittle/

#209. Chaetae <distribution along body; all annelids>/

1. present along most of body/

2. only present anteriorly/

3. present anteriorly and posteriorly/

4. only present posteriorly/

#210. <Chaetae> arranged <in each segment>/

1. in lumbricine arrangement - paired upper and lower bundles of one or a few chaetae each <typical of oligochaetes - specify number if possible; pairs within a bundle can be closely spaced or as far apart as between bundles>/

2. in perichaetine arrangement - chaetae more or less evenly distributed around perimeter of segment (more than 8 chaetae per segment) <typical of oligochaetes>/

3. in paired bundles (or rows) of many chaetae <typical of polychaetes>/

4. as a single bundle (or row) of many chaetae <typical of polychaetes>/

#211. <Lumbricine> chaetal bundles arranged <details of chaetal spacing within a segment>/

1. in closely spaced lateral and ventrolateral pairs/

2. in widely spaced lateral and ventrolateral pairs <or lateral and ventrolateral pairs not discernable>/

See 'perichaetine-' and 'lumbricine-chaetal arrangement', Annelida Glossary.

#212. Anal chaetae <presence>/

1. absent/

2. present/

Chaetae that surround the anus are present only in echiuran annelids; polychaetes only have chaetae present on true segments and they are usually reduced in size and number on the segments leading up to the anus-bearing pygidium.

#213. Anal chaetae <arrangement; number rings>/

1. one ring/

2. two rings/

#214. Aciculae <presence>/

1. absent/

2. present/

Aciculae are internal supportive chaeta (sometimes slightly projecting) of the parapodia of polychaetes; usually one, or at most a few, per parapodial lobe, often darker and more stout than external chaetae.

#215. Aciculae <position>/

1. in dorsal position (=notoaciculae)/

2. in ventral position (=neuroaciculae)/

3. in both dorsal and ventral positions (=noto- and neuroaciculae)/

#216. Neuroaciculae <form of tip>/

1. distally tapering <tip may be blunt or sharp>/

2. distally axehead-shaped/

3. distally bent at right angles/

#217. Capillary (or hair) chaetae <presence>/

1. absent/

2. present/

Capillary is a general term referring to a long, slender, hair-like chaetae tapering to a fine point; we have taken a rather broad view of what constitutes a capillary chaetae, and included forms having a variety of overall thicknesses (expanded hairs) and surface ornamentation (serrated hairs). The common feature of all capillaries is that they have a slender smooth shaft and taper to a point. Coding based largely on Merz and Woodin (2006).

#218. <Capillary chaetae; position within a segment>/

1. in dorsal (notopodial) position/

2. in ventral (neuropodial) position/

3. in both dorsal and ventral positions/

Capillaries (or hair) chaetae are common in aquatic oligochaetes and polychaetes. Presence of hair chaetae only in the dorsal position is the most usual state for oligochaetes, whereas polychaetes are more variable.

#219. <Capillary chaetae> distally <type, form>/

1. tapered to a point/

2. sharply bent/

3. expanded <includes bilimbate, knife shaped, unilimbate and whiplike bilimbate>/

4. with subdistal spur/

5. knife-shaped/

Capillaries may be serrated or smooth (see Annelida Glossary); sharply bent capillaries are smooth and bent at various angles; distally expanded capillaries include a variety of forms that are both symmetrical (bilimbate chaetae and whip-like bilimbate chaetae and asymmetrical (unilimbate chaetae); capillaries with a subdistal spur are also called bayonet chaetae (see Annelida Glossary); knife-shaped capillaries are a rare, special type of distally expanded capillary.

#220. <Capillary chaetae> edge <form of edge>/

1. smooth/

2. hirsute-serrate <hairs arranged on one or both (feathered) sides of capillary; sometimes called pectinate>/

3. spinose <very short hairs often covering the greater part of the circumference of the chaetae>/

4. corn-eared <spines arranged in whorls completely around chaeta>/

5. crenulate <lamellate-type serrations; includes subuluncini>/

A capillary chaeta is often slender and long, tapering to a fine point; in oligochaetes capillary chaetae are always smooth. In polychaetes they take a variety of forms. Annikey includes pectinate chaetae as a type of capillary chaetae (see Annelida Glossary).

#221. Capillary chaetae, externally <external structure; POLYCHAETES ONLY>/

1. not pseudo-segmented/

2. pseudo-segmented/

#222. Capillary chaetae, internally <internal structure; POLYCHAETES ONLY>/

1. distinctly chambered/

2. hollow (tubular)/

3. not chambered or hollow/

Chambered capillary chaetae are referred to as 'barred capillary chaetae' in the Annelida Glossary.

#223. Crotchet chaetae <also called sigmoid chaetae; gently S shaped curve usually with a swelling (nodulus) on the shaft; presence>/

1. absent/

2. present/

Crotchet chaetae are also called sigmoid chaetae, bifid crotchet chaetae or simple crotchet chaeta (see Annelida Glossary).

#224. <Crotchet chaetae; basic forms>/

1. simple-pointed <tapered to a single point>/

2. bifid/

3. distally pectinate <i.e., serrated>/

4. trifid/

Crotchet chaetae are typlical of oligochaetes. Of the different forms, the bifid type is most common, simple crotchets (not shown) are tapered to a single point, and pectinate crotchets (not shown) are distally serrated.

#225. Forked chaetae <presence>/

1. absent/

2. present/

Forked chaetae are chaeta that are distally bifurcated into two tines of equal or unequal in length; in some taxa smaller/finer teeth may be present in the bottom of the 'U' of the fork resembling an eating fork (see image here). Note that the similar needle chaetae in oligochate also may be forked distally, but differ in being very fine, and have a swollen nodulus mid-chaeta. See Annelida Glosssary for the range of forked chaetae.

#226. Forked chaetae <form; type>/

1. furcate/

2. ringent/

3. lyrate/

#227. Forked chaetae <length of tines>/

1. tines more or less equal in length/

2. tines distinctly unequal in length/

#228. Needle chaetae <presence; very thin emergent spines, often accompanying hair chaetae; OLIGOCHAETES ONLY>/

1. absent/

2. present/

Needle chaetae, or needle crotchets (see Annelida Glossary) refer to oligochaete chaetae that are very fine and needle-like, bearing a complex morphology of the tips; they project distinctly from the chaetal sacs (unlike the similarly fine support chaetae which never emerge beyond the chaetal sacs); also, unlike crotchets they do not have a bulge (nodulus) midway along the shaft.

#229. <Needle chaetae; form of tip>/

1. single pointed/

2. bifid <record whether teeth equal or unequal>/

3. pectinate/

#230. Support chaetae <presence; OLIGOCHAETES ONLY>/

1. absent/

2. present/

Support chaetae are very fine and tapered to a simple tip and never emerge beyond the chaetal sacs; they are always in a pair on either side of a normal chaeta.

#231. Comb-like chaetae <presence>/

1. absent/

2. present/

A simple chaeta with a comb-like arrangement of terminal teeth; includes flat trumpet chaetae (see Annelid Glossary).

#232. Comb-like chaetae <form>/

1. comb comprised of many long tines <true comb chaetae>/

2. comb comprised of many short tines <=flat trumpet chaetae>/

Short-tine combs are also called flat-trumpet chaetae (see Annelid Glossary).

#233. Silky (feltage) chaetae arising from notopodia of mid-posterior body <extremely thin, long silk-like chaetae; presence>/

1. absent/

2. present/

Silk chaetae are fine glistening silk-like chaetal fibres secreted by spinning glands in two families of Aphroditiformia polychaetes (see Annelida Glossary).

#234. Silky (feltage) chaetae <utilisation>/

1. incorporated into tube/

2. forming a felt cover on dorsal surface/

#235. Paleate chaetae <presence>/

1. absent/

2. present/

Paleate chaetae is a general term used here to refer to both opercula and parapodial paleae. Although both are similar externally, being broadly flattened with a metallic sheen, they appear to be differently constructed internally, and thus, likely of different origins. Parapodial paleae include those of Chrysopetalidae, referred to as camerate chaetae, because of their chamberlike internal structure (camerate paleae are closer structurally to camerate spines also present in Chrysopetalidae)

#236. Paleate chaetae <occurrence>/

1. associated with head <opercula paleae>/

2. associated with notopodia <parapodial paleae>/

#237. Parapodial paleae <arrangement>/

1. arranged in linear fans across dorsum/

2. arranged in rosettes across dorsum/

3. arranged in two lateral bunches on segment 2/

#238. Spines <presence; thickened chaetae, often slightly curved, tapering to a rounded point, and/or ornamented; mostly equivalent to simple falcate hooks of Merz & Woodin 2006>/

1. absent/

2. present/

Spines include a range of thickened chaetae, which are often slightly curved and taper to a rounded point; they may be ornamented. The different types of spines identified here include the camerate spines of Chrysopetalidae (which are actually allied to camerate paleae of the same family). Spines are called 'acicular spines' (acs) in the Annelida Glossary. Spine-like genital chaetae are not included here; they are often restricted to one or two segments and only appear at the time of reproduction.

#239. Spines <form>/

1. slightly curved and more-or-less smooth <includes sabre-chaetae>/

2. with small teeth or spinelets/

3. harpoon or arrow-shaped with reverse barbs/

4. with a single distal or subdistal hair (=aristate or hooded)/

5. sharply bent (=geniculate) or recurved/

6. plume-shaped, slightly curved, flattened distally/

Spines are robust chaetae, straight or gently curved, with a variety of different tips; see 'spines', 'modified spines' and 'recurved spines' (Annelida Glossary).

#240. Spines <distribution along body>/

1. present in most or all chaetigers/

2. present only in one or a few anterior chaetigers/

3. present only in posterior chaetigers/

#241. Spines <position within a segment>/

1. in dorsal (notopodial) position only/

2. in ventral (neuropodial) position only/

3. in both dorsal and ventral positions/

#242. Genital chaetae <presence; only on one or two segments - highly modified and different from regular chaetae; mostly in OLIGOCHAETA, rarely POLYCHAETA>/

1. absent/

2. present/

Genital chaetae are modified chaetae on genital segments that appear prior to reproduction; they include penial chaetae, which occur at the male or prostatic pores, and spermathecal chaetae (spc), which are associated with spermathecae (see Annelida Glossary). They are often non-emergent and can only be viewed by mounting a specimen on a slide and using transmitted light.

#243. Hooks <presence; equivalent to simple dentate hooks of Merz & Woodin 2006>/

1. absent/

2. present/

Hooks are similar in appearance to acicular uncini and the two terms have been confused in the literature; they are also similar to curved spines. For this dataset, hooks are recognised by being deeply embedded in the body wall and often occurring singly or in small clusters in anterior parapodia, whereas acicular uncini are often arranged in rows in posterior parapodia. Hooks differ from curved spines in the degree of distal curvature - hooks are bent almost at right angles whereas spines are straight to gently curved. See Annelida Glossary for a more complete description.

#244. Hooks <covering or appendage distally>/

1. without distal hood, beard or ligament/

2. with a subdistal beard/

3. with a distal hood/

4. with a subdental ligament/

#245. Hooks <position within a segment>/

1. occur in dorsal (notopodial) position/

2. occur in ventral (neuropodial) position/

Hooks in the ventral position are often called 'subacicular hooks' (see Annelida Glossary).

#246. Hooks <distribution along body>/

1. occur over entire body/

2. occur in anterior body only/

3. occur in posterior body only/

#247. Uncini <presence>/

1. absent/

2. present/

Uncini are compact, hooked-shaped chaetae that are usually arranged in rows (shown in yellow) on the posterior body. They come in two basic forms: those with a long shaft (called acicular uncini) and those having a flat base, i.e., without a shaft (avicular uncini). These two types are not differentiated in this dataset as it may be difficult to see the shaft, which is embedded in the body wall of the worm. The two types of uncini are differentiated and explained in more detail in the Annelida Glossary.

#248. Uncini <form distally - arrangement of teeth>/

1. with teeth in vertical series, teeth usually similar-sized (=pectinate)/

2. with teeth arranged in transverse series above an enlarged main fang (=crested)/

3. with a single tooth surmounting main fang/

4. with multiple teeth arranged in two opposing groups/

#249. Uncini <position within a segment>/

1. in ventral (neuropodial) position throughout/

2. in ventral (neuropodial) position on thorax and dorsal (notopodial) position on abdomen/

#250. Uncini <arrangement within each parapodium>/

1. arranged in one row/

2. arranged in two rows/

3. arranged in more than two rows/

#251. Compound chaetae <presence>/

1. absent/

2. present/

#252. Compound chaetae <position within a segment>/

1. in dorsal (notopodial) position/

2. in ventral (neuropodial) position/

#253. Compound chaetae appendage <form>/

1. distally tapering to slender tips (=spinigerous)/

2. distally curved (=falcate; ie., one distal tooth)/

3. multidentate/

Distally curved (falcate) types often have fine 'teeth' along the length of the appendage, but differ from distally toothed forms (no image) which have two or more heavy teeth at the tip of the appendage.

#254. Compound chaetae appendage <internal form>/

1. not canaliculated/

2. canaliculated/

#255. Compound chaetae appendage <presence of distal covering>/

1. without hoods or guards/

2. with a single hood open in front/

3. with paired guards on each side of the crest/

#256. Compound chaetae joint, appearance <symmetry>/

1. more-or-less symmetrical (=homogomph; includes also hemigomph and sesquigomph)/

2. distinctly asymmetrical (=heterogomph)/

3. flat or pseudocompound/

#257. Compound chaetae joint <attachment type>/

1. effected by ligament(s) <single or double>/

2. effected by fold in external sheath of chaeta/

#258. Compound chaetae shaft <form near joint>/

1. tapering slightly or evenly thick from emergence to joint/

2. distinctly inflated distally near joint/

#259. Compound chaetae shaft, internally <form internally>/

1. solid, without distinct core <may be grainy>/

2. chambered, with camerated core/

#260. Compound chaetae <distribution along body>/

1. present in a few anterior chaetigers only/

2. present in most or all chaetigers/

#261. Reproduction <sexual or asexual>/

1. asexual/

2. sexual/

#262. Asexual reproduction by <method>/

1. paratomy <producing a chain of zooids>/

2. budding <body axes of adult and new individuals not aligned>/

3. fragmentation/

#263. Sexual reproduction <separate male and female or hermaphroditic>/

1. hermaphroditic/

2. gonochoric <separate males and females>/

Citellates are hermaphroditic whilst polychaetes are usually gonochoric and rarely hermaphroditic.

#264. Clitellum <presence; only present in sexually mature specimens>/

1. absent/

2. present <in mature specimens>/

The clitellum is a sleave-like glandular thickening of a few mid-body segments bearing reproductive organs. Its presence is a defining feature of clitellates (oligochaetes and leeches, shown in separate images) and its form and position on the body are important for identification; unfortunately, it is only present in sexually mature individuals, so immature forms are often not identifiable. Further, in leeches it may be barely discernable even in reproductive individuals.

#265. <Clitellum structure>/

1. thin, single-layered/

2. thick, multilayered/

3. thin, multilayered/

Thickness is measured in the region of the male pores; the clitellum is one cell thick in all microdriles including Haplotaxidae and Alluroididae; in all other oligochaetes comprising the Crasiclitellata the clitellum comprises more than one layer of cells and is thicker, except for Moniligastridae which is multilayered but rather thin (Jamieson 2006).

#266. <Clitellum shape when fully developed in mature specimens; oligochaetes only>/

1. partially encircles body <saddle-shaped clitellum>/

2. fully encircles body <annular clitellum>/

The clitellum when fully developed may be either saddle-shaped or annular (completely encircling the segment) (see Annelida Glossary).

#267. <Clitellum situated; position in relation to male pores>/

1. in region of male pore(s) <covering or partially covering them>/

2. posterior to male pore(s)/

3. anterior to male pore(s)/

#268. <Clitellum situated; position in relation to female pores>/

1. in region of female pore(s) <covering or partially covering them>/

2. posterior to female pore(s)/

#269. Male and female pores separated by <number of annuli, or pseudosegments; Leeches ONLY>/

annuli/

#270. Clitellum occurs from <segments>/

#271. Seminal groove <presence on clitellum>/

1. absent/

2. present/

A seminal groove is a longitudinal, midventral groove extending posteriorly from the genital field and connecting prostate pores and male pores (see Annelida Glossary)

#272. Tubercula pubertatis <=external ridges or tubercles on clitellum for mating; presence, in sexually mature specimens only; presence>/

1. absent/

2. present/

Tubercula pubertatis are paired glandular swellings located near the ventro-lateral margins of the saddle-shaped clitellum (earthworms only) that may function to keep worms together during mating (see Annelida Glossary).

#273. <Tubercula pubertatis form>/

1. paired ridges on the ventrolateral margins of the clitellum/

2. paired ridges ventral to the clitellum/

3. discontinuous paired tubercules ventral to the clitellum/

Image source:

#274. Gonadal segments bearing genital papillae <presence>/

1. absent/

2. present/

The presence of papillae on the ventral surface of the gonadal segments is part of a broader ornamentation referred to as the genital field.

#275. Gonadal segments with extensions of the body wall <presence and form>/

1. absent/

2. present, as alae <lateral keel-like structures>/

3. present as claspers/

#276. Gonadal segments bearing a sperm transfer system in copulatory area <some LEECHES only>/

1. absent/

2. present/

Some leeches have an external modification of the clitellum in the copulatory area that allows the passage of sperm from male to female reproductive tissue

#277. <Gonadal segments bearing> gastropores <external slits and pores between the crop caeca and the ventral body wall in Leeches only>/

1. absent/

2. present/

Gastropores are extensions from the crop caeca to the ventral body wall exiting externally as pores and slits in segments XII and XIII. They presumably function in copulation.

#278. <Gonadal segments> gastropores on segments <segmental occurrence>/

#279. Testes <presence of discrete testes>/

1. absent/

2. present/

Testes, or testisacs when sperm-producing tissue is enveloped by a coelomic cavity, are the organs producing sperm. They are apparently absent in most non-clitellate Annelida. In oligochaetes, testes are generally located in the segment (sometimes across two segments) immediately anterior to the segment with the ovaries and with the male pores, while in leeches there tend to be a few pairs to many pairs (see Annelida Glossary).

#280. <Testes, number; CLITELLATA only; observed by dissection; segmental position often difficult to tell, especially in leeches>/

1. one pair in total <either segment 10 (proandric arrangement) or 11 (metandric arrangement) in oligochaetes>/

2. two pairs in total <segments 10 and 11 in oligochaetes; holandric arrangement>/

3. many pairs <i.e., more than a few; leeches and a few oligochaetes>/

4. single/

5. a few pairs/

#281. <Testes; segmental arrangement when more than one pair are present>/

1. one pair per segment <most leeches and oligochaetes>/

2. in multiple grape-like clusters per segment/

3. two pairs per segment <tetrad arrangement>/

#282. <Testes> present in segments <segmental occurrence>/

#283. Sperm sac <presence>/

1. absent/

2. present/

Sperm sacs are pouches on the opposite side of the prostate septa where the later stages of spermatogenesis occur, and also serve as sperm reservoirs

#284. Ovaries <presence>/

1. absent/

2. present/

Discrete ovaries are absent in most non-clitellate annelids; in clitellates, one pair of ovaries are typically present in the segment with the male pores, which is the segment after the one with the testes (see Annelida Glossary).

#285. <Ovaries, number>/

1. one pair <most oligochaetes and leeches>/

2. two pairs <rare among oligochaetes>/

3. unpaired/

Leeches always have a single pair of ovaries whilst oligochaetes are more variable.

#286. <Ovaries> present in segments <segmental occurrence>/

#287. Egg sacs <form; presence of distinct ovisac enveloping ovaries; only present in clitellates and *Hrabeiella*>/

1. absent/

2. present <present mostly in clitellates and *Hrabeiella*>/

Ovary architecture is similar in all clitellates.

#288. Egg sacs <form>/

1. globular <=spherical>/

2. tubular/

#289. Oviduct <form>/

1. single, shared oviduct from egg sac <=ovisacs>/

2. separate one for each egg sac <=ovisacs>/

#290. Vaginal sac <presence; and segment range; leeches ONLY>/

1. absent/

2. present/

#291. Segmental organs are <type; coded based on Rouse et al. 2022>/

1. protonephridia <in most segments serving only for excretion>/

2. metanephridia <in many segments function for excretion and reproduction>/

Segmental organs, or simply nephridia, are ducts that serve for both excretion/osmoregulation and gamete release (Bartolomaeus 1999). Most segments of Annelida contain nephridia that either are protonephridia (closed at the coelom) or metanephridia (open at the coelom via a nephrostome). Metanephridia occur in different segments along the body. Those in infertile anterior segments with complete septa only function in excretion and, therefore, can be strictly called nephridia. Posterior ones, in segments with incomplete septa often also function as gonoducts (hence preference for the term 'segmental organ', which suggests both a secretory and reproductive function. Although we have coded Clitellata as having metanephridia (following Rouse et al. 2022), some authors consider coelomoducts and metanephridia to be separate structures, especially in earthworms (Crassiclitellata); in microdriles metanephridia have been referred to as 'open exonephric holonephridia' (Goodrich 1945; Brinkhurst and Jamieson 1971; Jamieson & Ferraguti 2006); in leeches metanephridia are referred to simply as 'nephridia'. Consequently, all nephridal characters have been downweighted in importance for clitellate families in this dataset.

#292. Metanephridia <number of pairs>/

#293. Metanephridia <position with a segment; leeches only>/

1. ventral/

2. lateral/

#294. Metanephridia <distribution; coded based on Rouse et al. 2022; CODING FORMULATED FOR POLYCHAETES>/

1. along most of the body; anterior ones for excretion, posterior ones for gamete release/

2. several pairs metanephridia anteriorly for excretion, posterior ones for gamete release/

3. single anterior pair of excretory metanephridia and several more posterior ones for gamete release/

4. restricted to posterior thorax and anterior abdomen/

5. restricted to anterior and midbody segments/

#295. <Metanephridia> exiting via pore <basic form of excretory nephridia and reproductive coelomoducts>/

1. separate from gonoduct <= plectonephridia; typical of clitellates and some polychaetes; ciliated organs present or absent>/

2. in common with gonoduct <= typical metanephridia>/

Nephridia of polychaetes are usually of the combined form (ie., coelomoducts and nephridia exit through a common pore), which is referred to as metanephridia (see Annelida Glossary); those of clitellates are always separate.

#296. Nephridial pores <opening; =nephropores or nephridiopores; arrangement per segment; may be indicated externally by presence of pore only or papilla pore>/

1. paired (ventrolateral)/

2. single (ventromedial)/

3. dorsal/

4. lateral/

Nephridial pores are also called nephropores

#297. <Nephridial pores, distribution in relation to gonoducts; distribution as recognised by exterior pores, or through dissection; OLIGOCHAETES only>/

1. nephridial pores located anteriorly, gonoducts located around clitellum/

2. both nephridial pores and gonoducts located around clitellum/

3. nephridial pores located posterior to gonoducts/

Nephridial pores (nephridiopores) of earthworms are situated laterally immediately behind intersegmental grooves; they are usually difficult to see, and even more so in microdriles, so nephridial characters are down-weighted in these groups

#298. <Nephridia, distribution along body; distribution; as recognised by exterior pores or through dissection; POLYCHAETES and LEECHES>/

1. present in most segments of the body/

2. several pairs in anterior body/

#299. <Nephridia; number in each segment; oligochaetes only>/

1. one pair nephridia in each segment (holonephridia)/

2. multiple, minute, nephridia in each segment (meronephridia)/

3. mixed holonephridia and meronephridia/

The metanephridia of oligochaetes can be divided into holonephridia (=holonephric; larger, one pair per segment) and meronephridia (=meronephric; smaller, more than one pair in each segment).

#300. Spermathecae <presence of internal spermathecae>/

1. absent/

2. present/

Spermathecae (or seminal receptacles) are sacs for sperm storage in the female reproductive tract which are transferred by mating (see Annelida Glossary); rare in polychaetes, common in oligochaetes and Branchiobellidae, questionably present in some leeches. External spermatophores (present in some oligochaetes, leeches and branchiobdellids) are not considered here.

#301. <Spermathecae; form; citellates only>/

1. simple/

2. with a basal diverticula/

Diverticulated spermatheca usually have a single branch (diverticulum) arising from the bulbous ampulla or the duct of the spermathecal pore (spo), while simple spermatheca lack this side branch (see Annelida Glossary)

#302. <Spermathecae; location relative to testicular segments; only relevant for hermaphrodites like most oligochaetes>/

1. post-testicular/

2. pre-testicular/

3. in testicular segments/

#303. Spermathecal pores <presence; scored only for clitellates>/

1. absent/

2. present/

The spermathecal pore is a distinct glandular protuberance typically anterior to the clitellum in the female tract of oligochaetes that receives packets of sperm (spermatophores) (see Annelida Glossary).

#304. <Spermathecal pores; paired or unpaired>/

1. unpaired <single, midventral>/

2. paired/

There are usually one or more pairs of spermathecal pores per segment; rarely do they occur singly on the midventral line

#305. <Spermathecal pores; location relative to male pores>/

1. located within 1 or 2 segments of male pores <on or near clitellum>/

2. located well anterior to male pores <the prosothecous condition>/

#306. Spermathecal pores <number> pairs/

#307. <Spermathecal pores> in segment/

#308. Female gonoducts <presence>/

1. absent/

2. present <notes if conspicuous or inconspicuous>/

The female gonoduct is a duct leading from the ovary to the female pore (gonopore) in clitellates (see Annelida Glossary); it (they) are located either anterior to the clitellum or in clitellar segments.

#309. Vaginal sac <=vaginal caecum; presence>/

1. absent/

2. present/

#310. Female pores <paired or single>/

1. one pair <usual oligochaete condition>/

2. two pairs/

3. single, median <usual leech condition>/

Female paired pores of oligochaetes are always located in the segment behind the corresponding ovaries; they are usually inconspicuous; the leech pores are always single.

#311. <Female pores> in segment/

We score this character for both clitellates (oligochaetes and hirudinids) using that groups traditional Roman numeral notation and non-clitellates (Arabic numerals)

#312. Male gonoducts <=seminal vesicles or ejaculatory ducts in LEECHES; presence>/

1. absent/

2. present/

The male gonoduct (=vas deferentia or sperm duct) is a duct leading from the area of sperm production in the coelom to the male pore (gonopore) in clitellates (see Annelida Glossary); it (they) are located anterior to the clitellum.

#313. <Male gonoducts distally> with atrium <presence>/

1. absent/

2. present/

#314. <Male atrium; form>/

1. bilobed/

2. fused/

The atrium is a muscular distal chamber of the male gonoducts; it may be lined with prostate cells (as in many microdriles) but they are not true prostate as a separate organ as in some earthworms. The atrium either terminates in the penis or opens to the exterior by a pore.

#315. Male pores <paired or single; only scored for clitellates>/

1. single, median <the normally paired male ducts join to form single mid-ventral external pore>/

2. one pair <paired male ducts remain separated>/

3. two pairs <two pairs of male ducts>/

4. three pairs/

5. four pairs/

The male pore (mpo) is the external opening of the male gonoduct (=vas deferentia or sperm duct) in clitellates. Leeches have a single male pore whilst oligochaetes have more than one.

#316. <Male pores> in segment <of the vasa deferentia; segmental location>/

We score this character for both clitellates (oligochaetes and hirudinids) using that groups traditional Roman numeral notation and non-clitellates (Arabic numerals)

#317. <Male pores, position in relation to testis; score for clitellates, EXCLUDING LEECHES>/

1. prosoporous (same segment as corresponding testes)/

2. plesioporous (in segment following testicular segment)/

3. opisthoporous (two or more segments following testicular segment)/

This is a useful character to separate oligochaetes and leeches.

#318. Penis <presence; eversible structure that may be covered by a hardened sheath>/

1. absent/

2. present/

An annelid penis is mainly found in clitellates in the vicinity of the clitellum; it is usually difficult to see. It may be soft or surrounded by a hardened penis sheath (see Annelida Glossary).

#319. <Penis> with hardened sheath <presence>/

1. absent/

2. present/

A penis sheath is a hardened (chitinised) structure enclosing the penis in some annelids, mostly clitellates (see Annelida Glossary).

#320. Prostate gland <presence>/

1. absent/

2. present/

Prostate glands produce fluid for the transport of sperm; extend to the exterior through male pores (see Annelida Glossary). This character refers to the presence of a distinct gland, and does not count prostate tissue that might be associated with male ducts (=diffuse prostates).

#321. <Prostate gland; form>/

1. tubular <central lumen and no side branches>/

2. lobular <=racemose; branching ductlets with no central lumen>/

A racemore prostate is also referred to as a lobular prostate; a tubular prostrate (tpr) resembles folded tubes (see Annelida Glossary). Intermediate forms may exist but, for simplicity, are not coded here.

#322. <Prostate gland; number pairs>/

1. one pair/

2. more than one pair/

#323. Prostate pores in segment <location>/

The prostate pore is the external opening connecting the prostate gland in oligochaetes (see Annelida Glossary) Practical identification tips: Identification of sexual anatomy and external openings (pores) is important taxonomic information in Clitellata but it is difficult to obtain. Identifying these structures is best done by interpreting their anatomy relative to one another and by recognising characteristic patterns. [add more about these patterns].

#324. Relationship between prostate pore and male pore on segment XVII <united or not>/

1. united, discharge through single pore/

2. not united, separate pores/

#325. Tube <presence>/

1. absent/

2. present/

#326. Tube <type>/

1. membranous <mucus based and often covered with sediment particles>/

2. leathery or parchment like/

3. hard, composed of calcium carbonate <straight, curved or spiral>/

4. close-fitting, uncemented, sand grains forming tubes that are cone or tusk shaped/

5. cemented sand grains/

6. translucent, chitin-like <may be partially covered with sediment particles>/

Our concept of a tube precludes a simple mucous sheath, which many polychaetes can generate, particularly when under stress.

#327. <Tube> entrance able to be plugged by worm <presence; of a part of the worm that closes the tube>/

1. absent/

2. present/

A plug in the form of a specialised chaetae (left and right) or an operculum (centre) effectively seals the opening of the tube.

#328-333 Non-morphological characters

**References**

Annelida Glossary: Glossary of terms for identification of the major groups of annelid worms, [doi.org/10.5281/zenodo.14848165](https://doi.org/10.5281/zenodo.14848165).

Bartolomaeus T (1999) Structure, function and development of segmental organs in Annelida. Hydrobiologia 402: 1 –37.

Brinkhurst RO, Jamieson BGM (1971) Aquatic Oligochaeta of the World with contributions by D.G. Cook, D.V. Anderson, J. van der Land. University of Toronto Press, Toronto, Canada, 860 pp

Capa M, Kupriyanova E, Nogueira JMdM, Bick A, Tovar-Hernández MA (2021) Fanworms:Yesterday, Today and Tomorrow. Diversity 13: 130, https://doi.org/10.3390/d13030130.]

Fitzhugh K (1989). A systematic revision of the Sabellidae-Caobangidae-Sabellongidae complex (Annelida: Polychaeta). Bulletin of the American Museum of Natural History. 192: 1–104., available online at <http://hdl.handle.net/2246/881>

Goodrich ES (1945) The study of nephridia and genital ducts since 1895. Quarterly Journal of Journal of Microscopical Science 86: 113-301.

Gonzalez BC, Martínez A, Borda E, Iliffe TM, Eibye-Jacobsen D, Worsaae K (2018) Phylogeny and systematics of Aphroditiformia. Cladistics 34: 225–259. doi: 10.1111/cla.12202

Hutchings PA, McRae J (1993) The Aphroditidae (Polychaeta) from Australia, together with a redescription of the Aphroditidae collected during the Siboga Expedition. Records of the Australian Museum 45(3): 279–363.

Jamieson BGM (2006) Non-leech Clitellata (with contributions by Marco Ferraguti) In Reproductive Biology and Phylogeny of Annelida. Series Editor BGM Jamieson. Volume 4. Editors G Rouse, F Pleijel. Science Publishers, Enfield, New Hampshire, 235–392.

Marchan DF, Novo M, Fernandez, R, De Sosa I (2016) Evaluating evolutionary pressures and phylogenetic signal in earthworms: a case study – the number of typhlosole lamellae in Hormogastridae (Annelida, Oligochaeta). Zoological Journal of the Linnean Society 178: 4–14

Merz RA, Woodin SA (2006) Polychaete chaetae: function, fossils and phylogeny. Integrative & Comparative Biology 46: 481–496

Pettibone MH (1969) Revision of the aphroditoid polychaetes of the family Eulepethidae Chamberlin (=Eulepidinae Darboux, =Pareulepidae Hartman). Smithsonian Contributions to Zoology 41: 1–44., available online at http://si-pddr.si.edu/dspace/handle/10088/5690

Purschke G, Bleidorn C and Struck T (2014) Systematics, evolution and phylogeny of Annelida – a morphological perspective. Memoirs of Museum Victoria 71: 247–269.

[Reuscher M, Fiege D, Wehe T (2009) Four new species of Ampharetidae (Annelida: Polychaeta) from Pacific hot vents and cold seeps, with a key and synoptic table of characters for all genera. Zootaxa 2191: 1-40](https://www.marinespecies.org/aphia.php?p=sourcedetails&id=141909)

Rouse GW, Fauchald K (1997) Cladistics and polychaetes. Zoological Scripta 26: 139–204

Rouse GW, Pleijel F, Tilic E (2022) Annelida. Oxford University Press, Oxford, UK, 432 pp

Sawyer RT (1986) Leech biology and behaviour. Vol. 1 Anatomy, Physiology, and Behaviour. Oxford Science Publications, Oxford UK, 418 pp.

Tilic E, Rouse GW, Bartolomaeus T (2021) Comparative ultrastructure of the radiolar crown in Sabellida (Annelida). Zoomorphology 140: 27–45, <https://doi.org/10.1007/s00435-020-00509-x>

Verdes A, Gruber DF (2017) Glowing worms: Biological, chemical, and functional diversity of bioluminescent annelids. Integrative and Comparative Biology 57: 18–32.]

Wells GP (1959) The genera of Arenicolidae (Polychaeta). Proceedings of the Zoological Society of London 133: 301–314.
